# PyrAtes: Modular Organic Salts with Large Stokes Shifts for Fluo‐rescence Microscopy

**DOI:** 10.1002/anie.202318127

**Published:** 2024-04-03

**Authors:** Iakovos Saridakis, Margaux Riomet, Oliver J. V. Belleza, Guilhem Coussanes, Nadja K. Singer, Nina Kastner, Yi Xiao, Elliot Smith, Veronica Tona, Aurélien de la Torre, Eric F. Lopes, Pedro A. Sánchez‐Murcia, Leticia González, Harald H. Sitte, Nuno Maulide

**Affiliations:** ^1^ Institute of Organic Chemistry University of Vienna Währinger Strasse 38 1090 Vienna Austria; ^2^ Centre of Physiology and Pharmacology, Institute of Pharmacology Medical University of Vienna Schwarzspanierstraße 17A 1090 Vienna Austria; ^3^ Institute of Theoretical Chemistry University of Vienna Währinger Strasse 17 1090 Vienna Austria; ^4^ Vienna Doctoral School in Chemistry (DoSChem) University of Vienna Währinger Strasse 42 1090 Vienna Austria; ^5^ CeMM Research Center for Molecular Medicine of the Austrian Academy of Sciences Lazarettgasse 14 1090 Vienna Austria; ^6^ Hourani Center for Applied Scientific Research Al-Ahliyya Amman University 19328 Amman Jordan; ^7^ Center for Addiction Research and Science - AddRess Medical University Vienna Währinger Strasse 13 A 1090 Vienna Austria

**Keywords:** Fluorescence, Dyes, Stokes shift, Imidazo[1,2-*a*]pyridinium, Fluorophore

## Abstract

The deployment of small‐molecule fluorescent agents plays an ever‐growing role in medicine and drug development. Herein, we complement the portfolio of powerful fluorophores, reporting the serendipitous discovery and development of a novel class with an imidazo[1,2‐a]pyridinium triflate core, which we term *PyrAtes*. These fluorophores are synthesized in a single step from readily available materials (>60 examples) and display Stokes shifts as large as 240 nm, while also reaching NIR−I emissions at λ_max_ as long as 720 nm. Computational studies allow the development of a platform for the prediction of λ_max_ and λ_Em_. Furthermore, we demonstrate the compatibility of these novel fluorophores with live cell imaging in HEK293 cells, suggesting PyrAtes as potent intracellular markers.

## Graphical abstract

We report here the discovery of a powerful novel fluorophore class with an imidazo[1,2‐*a*]pyridinium core, termed PyrAtes. The synthesis was achieved in a single step, demonstrating a wide array of substrates with Stokes shifts as large as 240 nm, while also reaching NIR−I emissions at λ_max_ up to 720 nm. These novel fluorophores were successfully applied in live cell imaging in HEK293 cells, suggesting PyrAtes as potent intracellular markers.

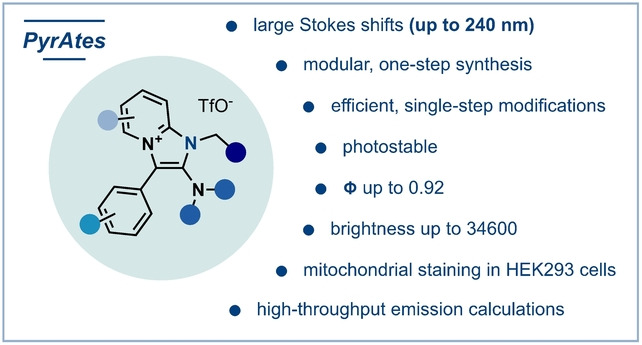

Almost two centuries after the serendipitous discovery of the fluorescent properties of the alkaloid quinine by Herschel^[1]^ and the coining of the term “fluorescence” by Stokes,^[2]^ the deployment of small fluorescent agents has become essential for medicine and drug development.[^3^, ^4^, ^5^, ^6^, ^7^, ^8^, ^9^] In particular, the advent of the (super‐resolved) fluorescence microscopy era[^10^, ^11^, ^12^, ^13^, ^14^, ^15^] and bioorthogonal technologies[^16^, ^17^, ^18^, ^19^] has revolutionized our understanding of biological function, enabling the visualization of architectures and dynamics within tissues, cells, and organelles.[^20^, ^21^]

The design and synthesis of powerful small‐molecule fluorescent reagents goes hand‐in‐hand with the remarkable advances in fluorescence microscopy.^[22]^ However, most of the chemical matter in this area is clustered around well‐established, state‐of‐the‐art fluorophores:[^23^, ^24^, ^25^] Rhodamines, fluoresceins, coumarins, cyanines and (boron‐dipyrromethenes) BODIPYs are the most utilized fluorescent probes, a consequence of their distinct advantages (Figure 1).

**Figure 1:**
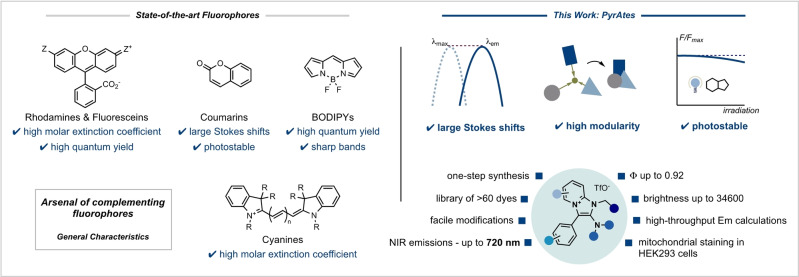
State‐of‐the‐art fluorescent dye families and PyrAtes as a new complementary fluorophore family.

Nevertheless, the continued development of novel families of fluorophores, particularly with complementary properties to those available, remains a sought endeavor.^[26]^

Among the various features of a fluorophore (e.g., fluorescence brightness—the product of fluorescence quantum yield and molar extinction coefficient), its value and applicability in fluorescence microscopy are highly dependent on its Stokes shift (i.e., the difference in energy between the emission and absorption maxima).^[27]^ The use of a dye with a moderate to narrow Stokes shift in fluorescence imaging typically has negative consequences. Indeed, a significant overlap between absorption and emission bands (self‐absorption) results in reduced signal‐to‐noise ratios, thus hampering image resolution.[^28^, ^29^] Alternatively, this overlap mandates excitation at wavelengths shorter than the λ_max_, resulting in (a) usually lower absorptivity, (b) potential tissue damage (e.g., in in‐vivo studies) and (c) detrimental effect on the signal‐to‐noise ratio through possible auto‐fluorescence of cells. Circumventing this outcome, technologies have been devised involving energy transfer to a second fluorophore, either through‐space (Förster resonance energy transfer, FRET)[^30^, ^31^] or through‐bond.[^32^, ^33^] Nevertheless, the design and synthetic efforts of probes employed in such techniques are rather demanding.

Among contemporary trends in dye synthesis, a stride towards higher synthetic modularity has emerged in recent years. Indeed, poor synthetic accessibility usually results in costly protocols, slow library enrichment or the need to develop special, tailor‐made procedures dedicated to specific challenges. For instance, recent studies have developed tailor‐made BODIPYs with large Stokes shifts, surpassing the narrow Stokes shifts of most members of that family.[^34^, ^35^] However, a *general* family of photostable fluorophores, with large Stokes shifts and a unified modular synthesis remains elusive.

We noted that intrinsic modularity of multi‐component reactions (MCR) would serve as the best match for the needs of small‐molecule fluorescent probes.[^36^, ^37^, ^38^] Herein, we report an unprecedented MCR platform for the construction of novel small‐molecule fluorescent dyes from readily accessible feedstock materials, computational studies of their properties, as well as imaging in live cells.

Our research group recently reported the α‐amination of amides via electrophilic activation involving alkyl azides as aminating reagents.[^39^, ^40^] During these studies, we serendipitously discovered that when benzyl amide **1 a** was engaged in the protocol in the presence of 2‐fluoropyridine **2 a** and azide **3 a**, imidazo[1,2‐*a*] **pyr** idinium trifl **ate** (henceforth termed *PyrAte*) salt **4 a** was obtained in 30 % yield, in lieu of the anticipated amination adduct (Scheme 1; see Supporting Information for a detailed postulated reaction mechanism). Strikingly, when a solution of the salt was subjected to UV irradiation, intense fluorescence was observed (λ_max_=295 nm, λ_Em_=430 nm, Φ=0.49, ϵ=7200, in methanol).[^41^, ^42^, ^43^, ^44^, ^45^, ^46^] Prompted by these preliminary results, we carried out extensive reaction optimization (see Supporting Information for details), increasing the yield to 79 % (isolated 72 %).

**Scheme 1:**
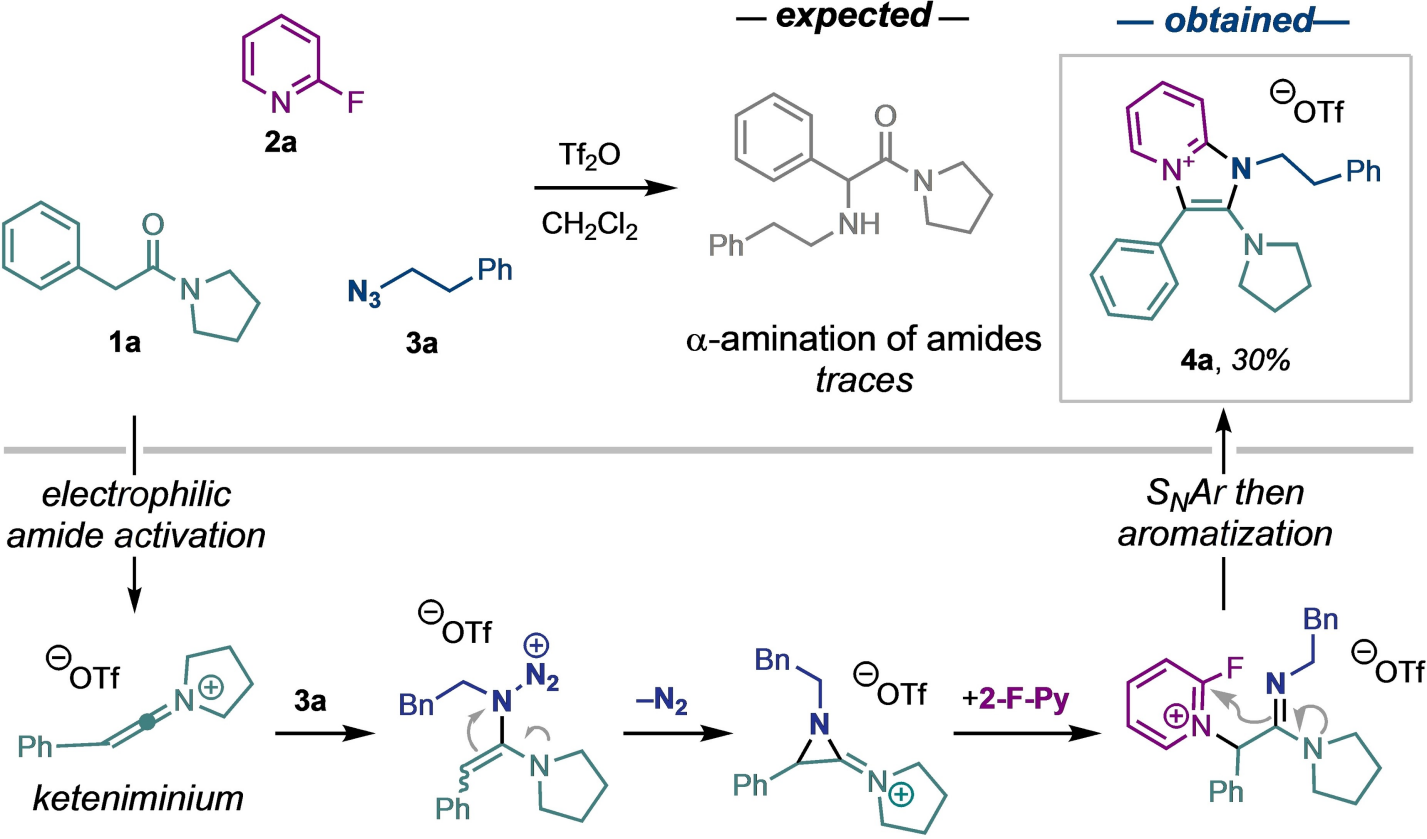
The serendipitous discovery of PyrAtes during our studies on α‐amination of amides (top) and postulated mechanism (bottom, see Supporting Information for details).

The promising photophysical properties of **4 a** inspired us explore the scope and limitations of this multi‐component transformation. The robustness of the method is reflected by the easily accessible large library of PyrAtes summarized in Figure 2A. The protocol is general and provides the fluorescent salts in a single operation while tolerating functionalities such as nitro (**4 b**), ethers (**4 d**), halides (**4 f**), a second azide handle (**4 n**), olefins (**4 l**), or esters (**4 v**). Interestingly, a curiosity‐driven exploration resulted in the development of a subclass of our fluorescent dyes: When the azide (a nitrenoid) was replaced by lutidine *N*‐oxide (LNO, an oxenoid), a new type of oxazolopyridinium salt was obtained instead. This subclass of PyrAtes, termed OxoPyrAtes, also exhibits fluorescence (Figure 2A). Moreover, when selected OxoPyrAtes were treated with Lawesson's reagent (see Supporting Information for details), the corresponding sulfur‐containing fluorescent salts were obtained as a second subclass of PyrAtes, which we term ThioPyrAtes (Figure 2B). The optical and photophysical properties of a selection of PyrAtes, OxoPyrAtes and ThioPyrAtes are summarized in Table 1 (see Supporting Information for details). To estimate the applicability of our dyes in aqueous media, the properties of representative dyes were measured—apart from methanol—in PBS (phosphate‐buffered saline)/DMSO 99 : 1 (v/v). Our findings occasionally showed a quantum yield drop *vs* in methanol (cf. **4 e** or **6 a**), however, counterbalanced with a small (**4 e**; +6 nm) or significant (**4 i**; +22 nm) enhancement of the Stokes shift.

**Figure 2:**
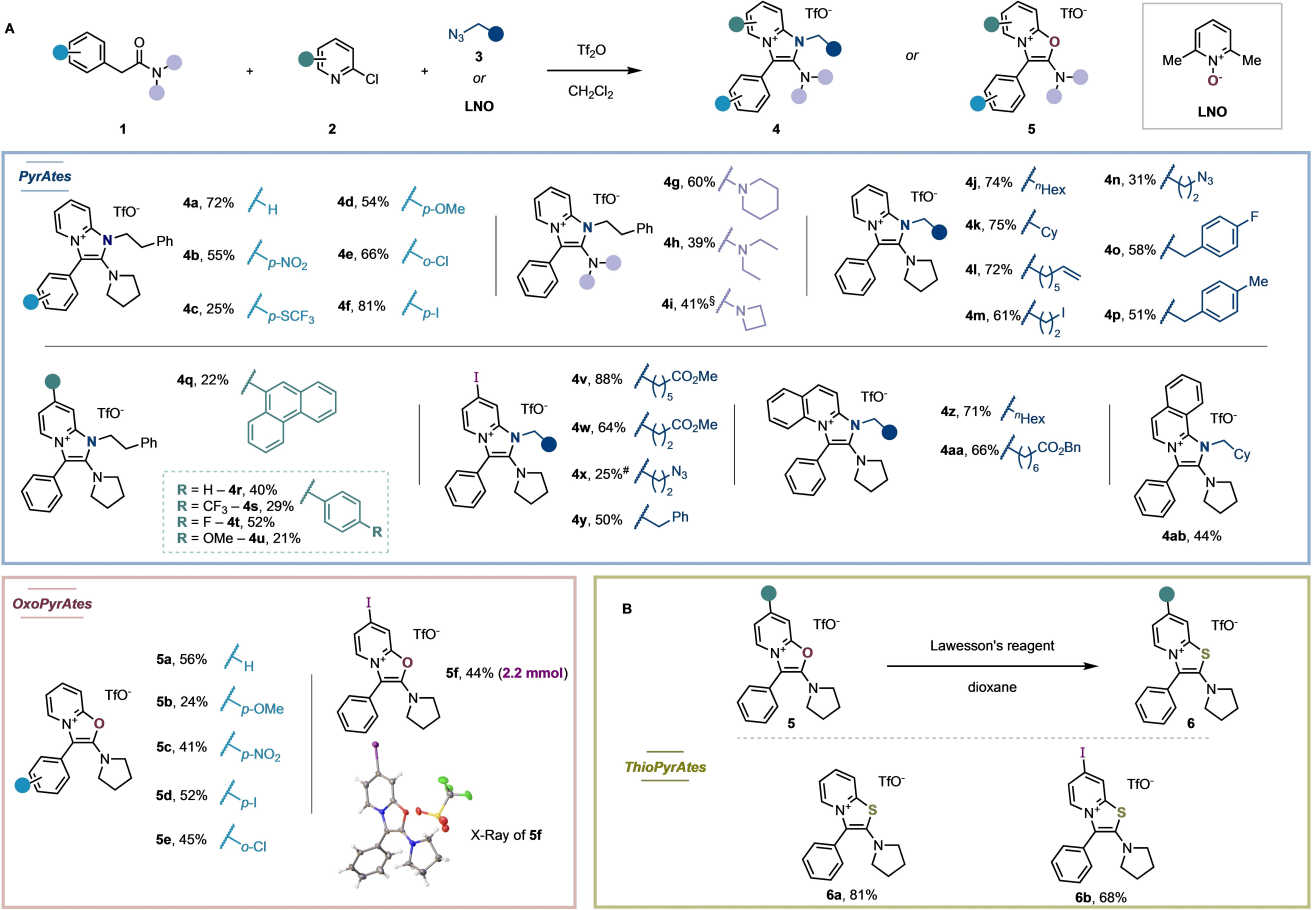
Scope of the multi‐component fluorophore synthesis. Synthesis of (A) PyrAtes and OxoPyrAtes and (B) ThioPyrAtes. Conditions: A) reactions were performed on a 0.2 mmol scale. Amide (1.0 equiv.), 2‐chloropyridine (5.0 equiv.), Tf_2_O (2.0 equiv.), azide (2.0 equiv.), CH_2_Cl_2_ (0.1 M), 0 °C to 20 °C, 16 h. For OxoPyrAtes: LNO (1.5 equiv.) was used instead of the azide. B) OxoPyrAtes (1.0 equiv.), Lawesson's reagent (0.55 equiv.), 1,4‐dioxane (0.1 M), 80 °C, 16 h. Tf_2_O: Triflic anhydride, LNO: lutidine‐*N*‐oxide. ^#^Yield after semi‐purification, the substrate was used as such at the next cross‐coupling step towards **7 e**. ^§^Trifluoroacetic acid (TFA) salt instead of triflate. See Supporting Information for details.^[79]^

**Table 1:** Photophysical properties of selected PyrAtes.^[a]^

cmpd	λ_max_ (nm)	λ_Em_ (nm)	Stokes shift (nm)	ϵ at λ_max_ (M^−1^ cm^−1^)	Quantum yield, Φ	Brightness at λ_max_ (M^−1^ cm^−1^)
**4 a**	295	430	135	7200	0.49	3600
**4 e**	293	412	125	5300	0.68	3600
4 e*	287	418	131	6610	0.13	890
**4 i**	331	436	105	5300	0.62	3300
4 i*	318	445	127	6310	0.68	4300
**4 t**	342	458	115	12900	***0.92***	11900
**4 u**	***350***	454	104	***16400***	0.76	***12400***
**4 z**	323	***480***	***157***	7400	0.53	3900
**5 a**	357	450	93	12000	***0.22***	***2600***
5 a*	357	450	93	7100	0.21	1500
**5 b**	***358***	***479***	***121***	***14200***	0.13	1900
**6 a**	374	489	115	19300	0.19	3700
6 a*	375	487	112	21200	0.065	1400

[a] Measurements in methanol or PBS/DMSO 99 : 1 v/v (*) (See Supporting Information for details). Highest values of the properties per PyrAtes class are highlighted.

Despite the satisfying properties of the described PyrAtes, we naturally targeted emissions at longer wavelengths to unlock application of these probes in living cells with fluorescence microscopy. The preliminary data gathered on compounds **4 r**–**4 u** hinted at the influence of the substituent's electronics, at the position‐4 of the pyridinium moiety, on the hypso‐/bathochromic properties of our fluorophores. This was further supported by computational studies of HOMO–LUMO gaps of selected substrates (see Supporting Information for details). Additionally, it is well known that extended π‐systems between “push‐pull” moieties can lower HOMO–LUMO gaps of chromophores.[^47^, ^48^] We therefore conducted post‐synthetic cross‐couplings on iodo‐PyrAtes **4 w**–**4 y**, iodo‐OxoPyrAte **5 f**, and iodo‐ThioPyrAte **6 b** with a series of aryl and vinyl boronic esters (Figure 3). Electron‐withdrawing or strong electron‐donating aromatic systems were introduced with spacers consisting of either zero (e.g., **7 a** or **7 d**), one (e.g., **7 i** or **9 b**), two (e.g., **7 k**) or three double bonds (**7 o**). Notably, the introduction of a julolidine moiety as the electron‐donating moiety afforded the reddest‐emitting fluorophores **7 l** and **8 i** in the NIR−I region, elevating the maxima of emission wavelengths up to 685 nm and 720 nm, respectively. The superiority of julolidine over *N*,*N*‐diethylaniline (e.g., **7 a** vs **7 b**) is commonly attributed to the “locking” of the C_Ar_−N bond rotation, resulting in better overlap between the lone pair at nitrogen and the π* orbital of the aromatic ring. Optical and photophysical properties of selected salts are summarized in Table 2 (see Supporting Information for details). Again, a decrease in the average brightness was observed in aqueous media, although with greater enhancement of the Stokes shift, ranging from +5 nm (**7 o**) to +81 nm (**8 e**).

**Figure 3:**
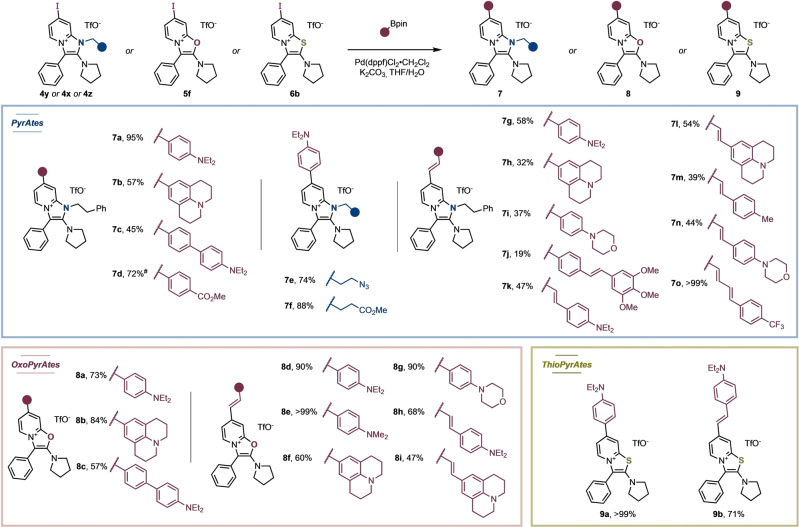
One‐step post‐synthetic modifications of PyrAtes. OxoPyrAtes and ThioPyrAtes with extended π‐conjugated systems. Conditions: Iodo‐PyrAte (1.0 equiv.), boronic ester (1.1 equiv.), Pd(dppf)Cl_2_ (0.05 equiv.) and K_2_CO_3_ (2.0 equiv.) in THF (0.1 M) at 23 °C for 16 h. ^#^Was obtained as a mixture of triflate and TFA salts. See Supporting Information for details.

**Table 2:** Photophysical properties of selected PyrAtes with extended π‐systems.^[a]^

cmpd	λ_max_ (nm)	λ_Em_ (nm)	Stokes shift (nm)	ϵ at λ_max_ (M^−1^ cm^−1^)	Quantum yield, Φ	Brightness at λ_max_ (M^−1^ cm^−1^)
**7 a**	406	493	87	33400	0.84	28000
7 a*	393	500	107	20600	0.24	4900
**7 b**	415	520	105	19900	0.36	7000
**7 c**	397	624	***227***	***42400***	0.20	8500
**7 f**	406	493	87	38840	***0.89***	***34600***
**7 l**	***470***	***685***	215	31200	0.18	5500
**7 m**	390	498	108	25200	0.14	3500
7 m*	393	519	121	38500	0.44	16900
**7 n**	418	636	218	28100	0.15	4200
**7 o**	397	532	135	41600	0.37	15500
7 o*	411	551	140	22500	0.16	3500
**8 a**	433	508	75	41500	***0.66***	***27000***
8 a*	423	513	90	18100	0.18	3200
**8 c**	422	680	***240***	***47800***	n.d.	n.d.
**8 e**	455	598	143	36300	0.17	6000
8 e*	461	685	224	46100	0.087	4000
**8 i**	***497***	***720***	223	42400	0.10	4300
**9 a**	448	522	74	39700	0.52	20500
**9 b**	483	615	132	38900	0.34	13300
9 b*	463	618	155	51200	0.083	4300

[a] Measurements in methanol or PBS/DMSO 99 : 1 v/v (*) (See Supporting Information for details). Highest values of the photophysical properties on PyrAtes and OxoPyrAtes are highlighted. Φ for **8 c** is not measured due to lack of matching standard.

One of the most significant findings of this study is that the synthesis of PyrAtes is a straightforward process and delivers fluorophores with large Stokes shifts (typically >135 nm). Figure 4A outlines a brief comparison of Stokes shifts of representative PyrAtes (bottom) with selected known fluorophores (top).[^28^, ^29^, ^49^, ^50^, ^51^, ^52^, ^53^, ^54^, ^55^, ^56^, ^57^, ^58^]

**Figure 4:**
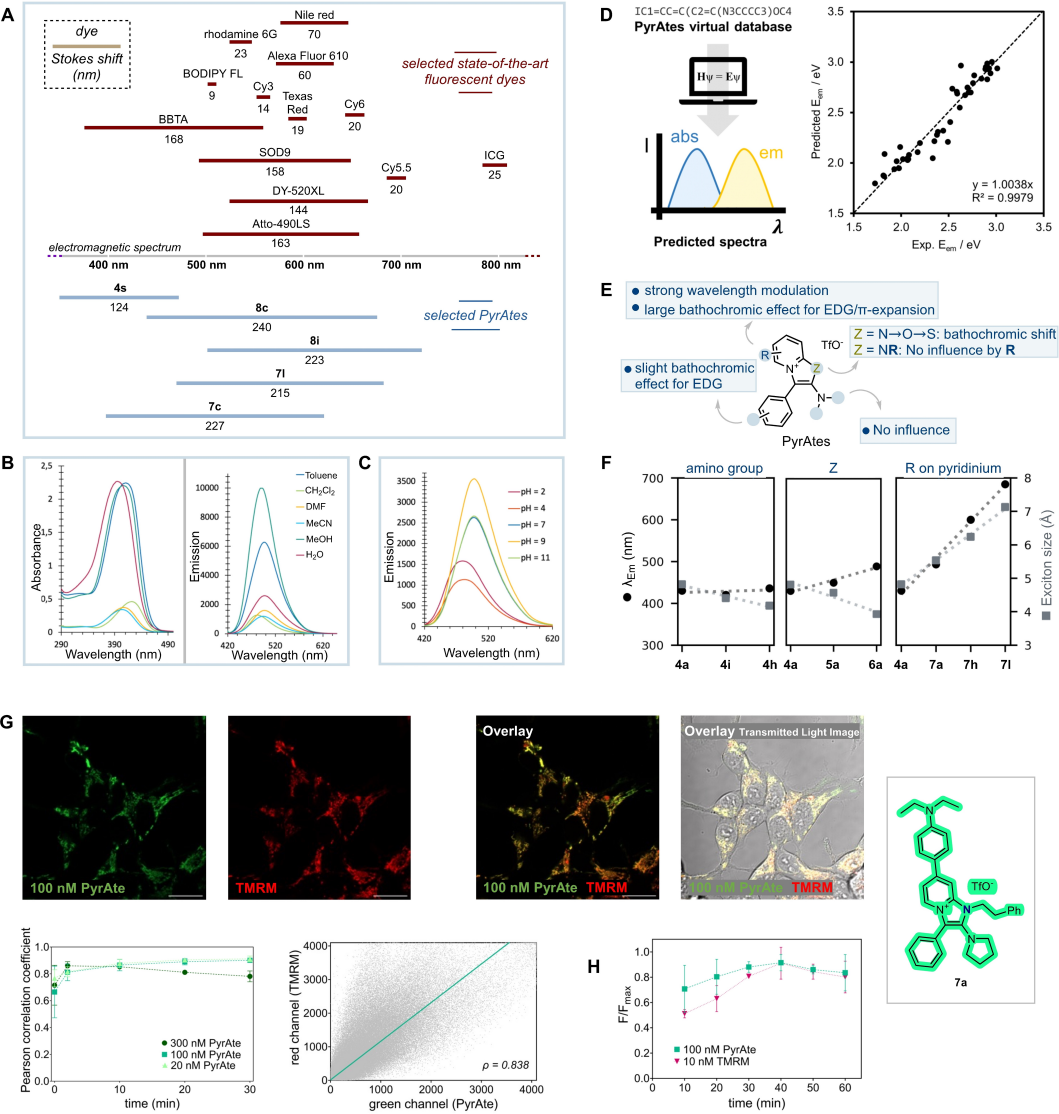
(A) Stokes shift comparison of our PyrAtes with known fluorophores. (B) Solvatochromic effect studies on PyrAte **7 a**. (C) pH sensitivity of PyrAte **7 a**. (D) Scheme of the semi‐automatic computational workflow to predict absorption and emission maxima using simple SMILES descriptors. The regression (*n*=44 compounds) of the predicted emission maxima vs experimental values (eV) is shown as an example. (E,F) Structure‐property relationship established experimentally (E) and theoretically via the calculation of exciton sizes (F). (G) PyrAte **7 a** (100 nM, green) internalization and co‐localization with the mitochondrial marker tetramethylrhodamine (TMRM, red) after 20 minutes (t20) in HEK293 cells. Fluorophores were excited at a wavelength of 407 nm (PyrAte) or 560 nm (TMRM). Co‐localization was evaluated with the Pearson's correlation coefficient (ρ), yielding a strong correlation with ρ=0.838.^[68]^ An overview of Pearson's correlation coefficients for three different concentrations (20 nM light green, 100 nM green, 300 nM dark green) over several time points reaching from 0–30 minutes shows strong or very strong correlation for all tested conditions from 2 minutes onward (*n*=3). Scale bar is 20 μm. (H) PyrAte **7 a** and TMRM bleaching over time (*n*=2–4, ±SD). Parental HEK293 cells were incubated with 100 nM PyrAte and 10 nM TMRM and excited every minute for 15 seconds at a wavelength of 407.5 nm (PyrAte) or 560.2 nm (TMRM). Mean integrated intensity (fluorescence, F) was measured and plotted every ten minutes as a fraction of the maximum intensity (F/F_max_).

Change at the ground‐ and excited‐state energy profiles of certain fluorescent probes in response to environment (e.g., solvent[^59^, ^60^, ^61^] or pH^[62]^) has been an attractive means in proteomics for the study of protein‐protein interactions (PPIs) or protein folding,^[63]^ as well as in the study of lipid dynamics of cellular membranes, for diagnostics in medicine,[^62^, ^64^] and for selective organelle imaging.^[65]^ As a canonical probe, we examined the solvatochromic behavior of PyrAte **7 a** (Figure 4B). As shown in the spectra below, **7 a** shows a significant sensitivity towards solvents. In particular, aprotic polar solvents tend to diminish the absorbance capacity of the dye more than 4‐fold, with a small negative solvatochromism (−27 nm).[^66^, ^67^] Regarding emission intensity, a similar trend is observed, however with a negligible effect on the λ_Em_.

On the other hand, as expected from the structural nature of **7 a** (pKa of *N*,*N*‐diethylaniline conjugate acid ≈6.6), pH appeared to have a more profound effect. The acidity increase of aqueous buffers was accompanied by a hypsochromic effect, presumably due to decreased photoinduced intramolecular charge transfer onto the pyridinium ring (Figure 4C). As such, while pH values of 7 or above retain the photophysical properties of **7 a** (λ_max_=395 nm, λ_Em_=497 nm), pH 4 and 2 cause a significant blue‐shift with λ_max(pH 4)_=361 nm/λ_Em(pH 4)_=483 nm, and λ_max(pH 2)_=356 nm/λ_Em(pH 2)_=480 nm, respectively (see Supporting Information for details).

Furthermore, we implemented a semi‐automatic computational workflow for the prediction of absorption and emission maxima using simple 1D descriptors as input data (i.e., SMILES strings, see Supporting Information for details, Figure 4D left). We trained a regression model with a set of 9 molecules and predicted experimental emission values of 44 compounds. Figure 4D (right) shows the excellent prediction ability of the model for emission maxima (R^2^=0.998).

Next, we analyzed the molecular drivers that control the emission maxima in this chemical series (Figure 4E&F). Experimentally, it was found that the major modulations of fluorescence emission wavelength were obtained by modification of the 4‐position of the pyridinium ring. On the contrary, the *N*,*N*‐dialkylamino and aromatic residues on the amide partner have little to no influence on the optical properties. Finally, a bathochromic shift can be observed by variation of the heteroatom Z of the heterocycle (NR<O<S, Figure 4E).

The effect of these substitutions on the emission maxima can also be quantified leveraging the one‐electron transition density matrix[^69^, ^70^, ^71^] and calculating the size of the electron‐hole pair (exciton size) created upon light absorption and relaxation to the first excited state for these series (see Supporting Information for details).[^72^, ^73^] The electron‐hole pair model has been shown as a versatile tool to understand the effect of chemical fragments on electronic transitions upon light absorption.[^74^, ^75^] Figure 4F illustrates the evolution of experimental emission maxima (nm) and the calculated exciton size (Å) within the different PyrAte series. The substitution of the amino moiety originating from the amide (**4 a** (pyrrolidine)→**4 h** (NEt_2_)→**4 i** (azetidine)) leads to no significant change in the exciton size or the emission maxima, as found experimentally. A change in the heteroatom Z involves a slight decrease of the exciton size with an inversely proportional small redshift of the emission maximum (**4 a** (N−(CH_2_)_2_−Ph)→**5 a** (O)→**6 a** (S)). In contrast, there is a strong increase in both emission maximum and exciton size when the pyridinium moiety is modified within the series **4 a**→**7 a**→**7 h**→**7 l**, in vivid agreement with experimental observations. Importantly, both emission maximum and exciton size changes correlate with the extension of the conjugated system in the chromophore, red‐shifting the emission maxima.

Furthermore, to demonstrate the potential of PyrAtes in fluorescent labeling of biological structures, we applied PyrAte **7 a** in HEK293 cells. This compound was rapidly internalized by the cells, resulting in a bright, punctate staining pattern with minimal cytoplasmic background equilibrated after 20 minutes (Figure 4G).

The ample cell permeability observed enables the use of PyrAtes in targeted fluorescent labeling of intracellular structures. The morphology of the staining appears similar to those seen from known mitochondrial markers such as tetramethylrhodamine (TMRM) and MitoTracker®.[^76^, ^77^, ^78^] It would also be reasonable to expect that the compound gets internalized into the mitochondria, owing to its positive charge. To show that **7 a** labels the mitochondria, we used TMRM as a co‐stain in live cell imaging of HEK293 cells. Indeed, the co‐localization displayed in Figure 4G clearly demonstrates that **7 a** accumulates in the same cellular structures with a similar morphology, thereby confirming its activity as a mitochondrial marker. We also show similar staining of OxoPyrAte **8 a** (see SI). To be suitable for live imaging applications in biological samples, however, it is important to ensure that the compound is non‐toxic (see SI). In Figure 4H, we show that PyrAtes can be imaged in cells using confocal microscopy with no apparent cellular damage and minimal photo‐bleaching throughout 60 minutes of continuous laser irradiation, highlighting the excellent photostability of PyrAtes.

In summary, we developed a family of ionic dyes termed *PyrAtes* with the potential to complement state‐of‐the‐art fluorophores in fluorescence microscopy. PyrAtes exhibit large Stokes shifts (up to 240 nm), photostability as well as synthetic modularity. This allowed us to easily build a broad library of dyes emitting at as long λ_Em_ as 720 nm. Moreover, we implemented a semi‐automatic computational λ_Em_ prediction workflow, enabling us to efficiently expand the library. Furthermore, we demonstrated the applicability of our fluorophores in fluorescence microscopy, by staining mitochondria of HEK293 cells in vitro. Ongoing studies in our laboratories aim to control the mitochondrial accumulation of PyrAtes, thus enabling selective marking of molecules of interest and staining of certain specimens.

## Conflict of interests

N.M., G.C., M.R., I.S., V.T. and E.S. are inventors on a patent on the synthesis of fluorescent salts (PCT/EP2022/057779).

## Supporting information

As a service to our authors and readers, this journal provides supporting information supplied by the authors. Such materials are peer reviewed and may be re‐organized for online delivery, but are not copy‐edited or typeset. Technical support issues arising from supporting information (other than missing files) should be addressed to the authors.

Supporting Information

## Data Availability

The data that support the findings of this study are available in the supplementary material of this article.
