# Supplementary material for: PyrAtes: Modular Organic Salts with Large Stokes Shifts for Fluo‐rescence Microscopy
Source: Angew Chem Int Ed Engl. 2024 Apr 3;63(19):e202318127. doi: 10.1002/anie.202318127 (PMC11497256; doi:10.1002/anie.202318127)
Supplement: Supplementary file 1 — Supporting Information [file ANIE-63-e202318127-s001.pdf]

Angewandte Chemie
Eine Zeitschrift der Gesellschaft Deutscher Chemiker

Supporting Information

PyrAtes: Modular Organic Salts with Large Stokes Shifts for Fluorescence Microscopy

*I. Saridakis, M. Riomet, V. Tona, A. de la Torre, E. F. Lopes, P. A. Sánchez-Murcia, L. González, H. H. Sitte, N. Maulide**

Supporting Information

PyrAtes: Modular Organic Salts with Large Stokes Shifts for Fluorescence Microscopy

Iakovos Saridakis,[§,a,d] Margaux Riomet,[§,a] Oliver J. V. Belleza,[§,b] Guilhem Coussanes,[a] Nadja K. Singer,[c,d] Nina Kastner,[b] Yi Xiao,[a,d,e] Elliot Smith,[#,a] Veronica Tona,[#,a] Aurélien de la Torre,[a] Eric F. Lopes,[a] Pedro A. Sánchez-Murcia,[c] Leticia González,[c] Harald H. Sitte,[b,f,g] and Nuno Maulide[*,a,e]

Email: nuno.maulide@univie.ac.at Homepage: http://maulide.univie.ac.at

[a]Institute of Organic Chemistry, University of Vienna, 1090 Vienna, Austria; [b] Centre of Physiology and Pharmacology, Institute of Pharmacology, Medical University of Vienna, Vienna, Austria; [c]Institute of Theoretical Chemistry, University of Vienna, 1090 Vienna, Austria; [d]Vienna Doctoral School in Chemistry (DoSChem), University of Vienna, 1090 Vienna, Austria; [e]CeMM Research Center for Molecular Medicine of the Austrian Academy of Sciences, 1090 Vienna, Austria; [f]Hourani Center for Applied Scientific Research, Al-Ahliyya Amman University, Amman, Jordan; [g]Center for Addiction Research and Science - AddRess, Medical University Vienna, Vienna, Austria [§]I.S, M.R and O.B contributed equally. [#]E.S and V.T contributed equally

Contents

1. General Information 4
2. Photophysical Properties of PyrAtes 6
3. Reaction Optimizations....9
   3.1. Optimization for PyrAtes 9
   3.2. Optimization for OxoPyrAtes....10
4. Reaction mechanisms 11
   4.1. Synthesis of PyrAtes....11
   4.2. Synthesis of OxoPyrAtes 11
   4.3. Synthesis of ThioPyrAtes 12
5. Experimental procedures 13
   5.1. General Procedures 13
      5.1.1. General Procedure A — Synthesis of benzyl carboxamides....13
      5.1.2. General Procedure B — Synthesis of 2-chloropyridines 13
      5.1.3. General Procedure C — Synthesis of azides....14
      5.1.4. General Procedure D — Boron-Wittig Olefination....14
      5.1.5. General Procedure E — Synthesis of PyrAtes 15
      5.1.6. General Procedure F — Synthesis of OxoPyrAtes 15
      5.1.7. General Procedure G — Synthesis of ThioPyraAtes....16
      5.1.8. General Procedure H — Suzuki-Miyaura Cross-Couplings....16
   5.2. Characterization Data 17
      5.2.1. Amides 17
      5.2.2. 2-Chloropyridines 19
      5.2.3. Azides....21
      5.2.4. Boronic esters....23
      5.2.5. PyrAtes, OxoPyrAtes and ThioPyrAtes 32
6. Solvatochromism and pH studies 75
   6.1. Solvatochromic effect on 7a....75
   6.2. pH Effect on 7a 76
7. X-Ray Analysis of 5f....77
8. Internalization and colocalization studies in HEK293 cells....81
9. Cytotoxicity studies....82
10. Resistance to oxidation....83
11. Computational Studies 84

11.1. Computational Methods....84

11.2. XYZ-coordinates of the optimized S0 and S1 minima of compounds 4a, 5a, 6a, 7a, 8a, 9a, 7g, 8d, and 9b 91

12. NMR Spectra....103

13. References 211

1. General Information

- ***Chemical synthesis***

Unless otherwise stated, all glassware/reaction vessels were flame-dried before use and all reactions were performed under argon atmosphere. Trifluoromethanesulfonic anhydride (Tf_2O) was distilled over P_4O_{10} prior to use, and stored under argon atmosphere at 4 °C. All other reagents were used as received from commercial suppliers and stored under argon atmosphere, unless otherwise stated. The progress of reactions was monitored by thin layer chromatography (TLC) performed on aluminium plates (0.2 mm thickness) coated with silica gel F_{254} or with ^1H NMR spectroscopy. TLC chromatograms were visualized by fluorescence quenching with UV irradiation at 254 nm (or 366 nm) and/or by staining using either potassium permanganate or phosphomolybdic acid. Flash column chromatography was performed using silica gel 60 (230–400 mesh, Merck and co.).

All ^1H, ^{13}C, and ^{19}F NMR spectra were recorded using a Bruker AV-400, AV-500, AV-600 or AV-700 spectrometer at 300 K. Chemical shifts are given in parts per million (ppm, δ), referenced to the solvent peak of $CDCl_3$, defined at δ = 7.26 ppm (^1H NMR) and δ = 77.16 ppm (^{13}C NMR) or DMSO-d_6, defined at δ = 3.33 ppm (^1H NMR) and δ = 39.52 (^{13}C NMR). Coupling constants (*J*) are quoted in Hz. ^1H or ^{13}C NMR splitting patterns were designated as singlet (s), doublet (d), triplet (t), quartet (q), pentet (p). Splitting patterns that could not be interpreted or easily visualized were designated as multiplet (m) or broad (br). Protons and carbons were assigned whenever unambiguously possible.

IR (infrared) spectra were recorded using a Perkin-Elmer Spectrum 100 FT-IR spectrometer. Wavenumbers (ν_{max}) are reported in cm^{-1}. Mass spectra were obtained using a Finnigan MAT 8200 (70 eV) or MAT 8400 (70 eV) using electrospray ionization (ESI). High resolution mass spectra were recorded on a Bruker APEX III FT-MS (7 T magnet).

- ***Photophysical Properties***

Ultraviolet absorbance of PyrAtes was measured on a Thermo Fisher Scientific G10S spectrophotometer at concentrations of 1 µM, 5 µM, 10 µM and 20 µM in MeOH. Fluorescence was recorder on a SHIMADZU RF-6000 spectrofluorometer at concentration of 1 µM in MeOH. The fluorescence quantum yield is calculated relatively to a known standard by the spectrofluorometer. The selected standard must absorb at the desired absorption (around the λ_{max} of the sample). The compound's measured fluorescence must be included within the standard's emission range. The available standards with which we measured the quantum yields of PyrAtes are: Quinine sulfate (QS), with Φ_f = 0.59 in 0.105 M $HClO_4$; Coumarin 153 (C153), with Φ_f = 0.53 in EtOH; Rhodamin 6G (R6G), with Φ_f = 0.91 in EtOH.[1] The refractive index correction for measurements in different solvents was taken into account (methanol, 1.326; aqueous media, 1.33).

- *Cell culture and confocal microscopy*

Human embryonic kidney 293 cells (HEK293 cells) were cultured in Dulbecco's modified Eagle's medium (DMEM, Capricorn Scientific GmbH, Ebsdorfergrund, Germany) with high glucose (4.5 g/L) and L-glutamine (584 mg/L), supplemented with 10% fetal bovine serum (FBS, Bio&Sell, Feucht, Germany), 100 units/mL penicillin and 100 µg/mL streptomycin (Sigma Aldrich, St Louis, USA). The cells were cultured in a humidified atmosphere (37 °C, 5% CO_2). For microscopy experiments, cells were seeded at 2.5 x 10^4 cells/0.25 mL into poly-D-lysine-coated 8-well polymer coverslip (ibidi GmbH, Gräfelfing, Germany) 24 hours prior to the experiment. Prior to imaging, cell culture medium was replaced with pre-warmed Krebs-HEPES buffer (KHB; composition: 10 mM HEPES, 120 mM NaCl, 3 mM KCl, 2 mM $CaCl_2·2H_2O$, 2 mM $MgCl_2·6H_2O$, 20 mM D-glucose, pH adjusted to 7.3). HEK293 cells were imaged using a Nikon A1R laser scanning confocal microscope equipped with a GaAsP detector, using a 60× oil-immersion objective. PyrAtes dissolved in DMSO were diluted in KHB (0.1% v/v) to make the desired final concentration (20 nM, 100 nM, 300 nM), and were added to each well containing the cells. For co-localization experiments, tetramethylrhodamine methylester (TMRM) was added to the cells at a final concentration of 10 nM, 10 minutes prior to addition of PyrAte compounds. PyrAtes were excited using a 407.5 nm laser, and its fluorescent signals collected using 525/50 nm emission filters; TMRM was excited using a 560.2 nm laser, and fluorescence was collected using 595/50 nm emission filters. Images were captured at specific time points after addition of the compounds. Images were post-processed and analyzed using ImageJ. Co-localization was evaluated with the Pearson correlation coefficient (ρ) using the JaCoP PlugIn.[2]

- *Cell viability assays*

HEK293 cells were seeded at a density of 3.6 x 104 cells/0.2 mL into poly-D-lysine coated 96-well plates 24 hours prior to the experiment. The culture medium was replaced with test compounds diluted in standard DMEM to achieve the final concentrations indicated. After one hour of incubation at 37 °C, the solutions were aspirated and replaced with AquaBluer™ reagent (MultiTarget Pharmaceuticals LLC, United States) according to manufacturer’s protocol. After four hours of incubation at 37 °C, fluorescence (Ex. 544 nm, Em. 595 nm) was measured using a Victor3 multilabel plate reader (PerkinElmer). Data were analyzed using Microsoft Excel 2016 and GraphPad Prism v7.0, with % cell viability calculated relative to the vehicle control conditions (0.1% DMSO), after making background corrections.

2. Photophysical Properties of PyrAtes

Table S1. *Photophysical properties of PyrAtes in MeOH or aqueous media (PBS:DMSO 99:1 v/v). Compounds* ***4b****,* ***4f****,* ***4m****,* ***4n****,* ***4v*****–*****4y****,* ***5c****,* ***5f****,* ***6b****, and* ***7e*** *are non-fluorescent salts. Internal standards were used for the measurement of quantum yield: QS, quinine sulfate; C153, coumarin 153; R6G, rhodamine 6G. The excitation wavelength of the quantum yield standard is noted.*

cmpd	λ_{max} (nm)	λ_{Em} (nm)	Stokes shift (nm)	ε at λ_{max} (M^{-1} cm^{-1})	Quantum yield	Standard, $\lambda_{exc(standard)}$	Brightness at λ_{max} (x10^3 M^{-1} cm^{-1})
Measurements in MeOH							
4a	295	430	135	7200	0.49	QS, 300 nm	3.6
4c	286	427	141	7647	0.18	QS, 300 nm	1.3
4d	300	448	148	5000	0.53	QS, 300 nm	2.6
4e	293	412	125	5300	0.68	QS, 300 nm	3.6
4g	295	422	127	7200	0.69	QS, 300 nm	5.0
4h	295	420	125	6000	0.53	QS, 300 nm	3.2
4i	331	436	105	5300	0.62	QS, 325 nm	3.3
4j	295	424	129	5300	0.55	QS, 300 nm	2.9
4k	295	427	131	5400	0.56	QS, 300 nm	3.0
4l	295	427	131	3300	0.51	QS, 300 nm	1.7
4o	295	427	131	5300	0.62	QS, 300 nm	3.3
4p	295	429	133	4800	0.56	QS, 300 nm	2.7
4q	343	464	121	16300	0.69	QS, 350 nm	11.2
4r	340	460	120	8400	0.86	QS, 350 nm	7.3
4s	350	474	124	10800	0.87	QS, 350 nm	9.4
4t	342	458	115	12900	0.92	QS, 350 nm	11.9
4u	350	454	104	16400	0.76	QS, 350 nm	12.4
4z	323	480	157	7400	0.53	QS, 325 nm	3.9
4aa	325	477	152	18200	0.38	QS, 325 nm	6.9
4ab	307	472	165	7500	0.20	QS, 300 nm	1.5
5a	357	450	93	12000	0.22	QS, 350 nm	2.6
5b	358	479	121	14200	0.13	QS, 350 nm	1.9
5d	356	451	95	17300	0.55	QS, 350 nm	9.4

**5e**	351	427	76	14600	0.19	QS, 350 nm	2.9
**6a**	374	489	115	19300	0.19	C153, 375 nm	3.7
**7a**	406	493	87	33400	0.84	C153, 400 nm	28.0
**7b**	415	520	105	19900	0.36	C153, 425 nm	7.0
**7c**	397	624	227	42400	0.20	R6G, 425 nm	8.5
**7d**	354	490	136	15800	0.79	QS, 350 nm	12.5
**7f**	406	493	87	38840	0.89	C153, 400 nm	34.6
**7g**	436	571	125	22700	0.11	C153, 425 nm	2.6
**7h**	455	600	145	26100	0.20	R6G, 450 nm	5.2
**7i**	401	565	164	19800	0.12	C153, 400 nm	2.4
**7j**	365	543	178	22600	-	*No standard match*	-
**7k**	455	644	189	36500	0.14	R6G, 450 nm	5.1
**7l**	470	685	215	31200	0.18	R6G, 475 nm	5.5
**7m**	390	498	108	25200	0.14	C153 400 nm	3.5
**7n**	418	636	218	28100	0.15	R6G, 425 nm	4.2
**7o**	397	532	135	41600	0.37	C153 400 nm	15.5
**8a**	433	508	75	41500	0.66	C153 425 nm	27.0
**8b**	445	528	83	33200	0.08	C153 450 nm	2.8
**8c**	422	680	240	47800	-	*No standard match*	-
**8d**	468	600	132	37900	0.14	R6G, 475 nm	5.2
**8e**	455	598	143	36300	0.17	R6G, 450 nm	6.0
**8f**	488	628	140	30500	0.08	R6G, 475 nm	2.5
**8g**	430	596	166	20300	0.18	R6G, 425 nm	3.7
**8h**	483	682	199	34800	0.17	R6G, 475 nm	5.9
**8i**	497	720	223	42400	0.10	R6G, 500 nm	4.3
**9a**	448	522	74	39700	0.52	C153, 450 nm	20.5
**9b**	483	615	132	38900	0.34	R6G, 500 nm	13.3
Measurements in aqueous media (PBS/DMSO 99:1 v/v)							
**4e**	287	418	131	6610	0.13	QS, 280 nm	0.89
**4i**	318	445	127	6310	0.68	QS, 325 nm	4.3
**4aa**	325	489	164	21100	0.41	QS, 325 nm	8.7
**5a**	357	450	93	7100	0.21	QS, 350 nm	1.5

**6a**	375	487	112	21200	0.065	QS, 375 nm	1.4
**7a**	393	500	107	20600	0.24	QS, 375 nm	4.9
**7f**	393	504	111	37700	0.27	C153, 400 nm	10.2
**7m**	393	519	121	38500	0.44	C153, 400 nm	16.9
**7o**	411	551	140	22500	0.16	C153, 400 nm	3.5
**8a**	423	513	90	18100	0.18	C153, 425 nm	3.2
**8e**	461	685	224	46100	0.087	R6G, 450 nm	4.0
**9a**	434	526	92	45400	0.039	C153, 450 nm	1.8
**9b**	463	618	155	51200	0.083	R6G, 475 nm	4.3

3. Reaction Optimizations

3.1. Optimization for PyrAtes

*Table S2. Reaction optimization for the synthesis of PyrAte **4a**. Reactions conducted on a 0.1 mmol scale. NMR yields obtained with mesitylene as an internal standard.*

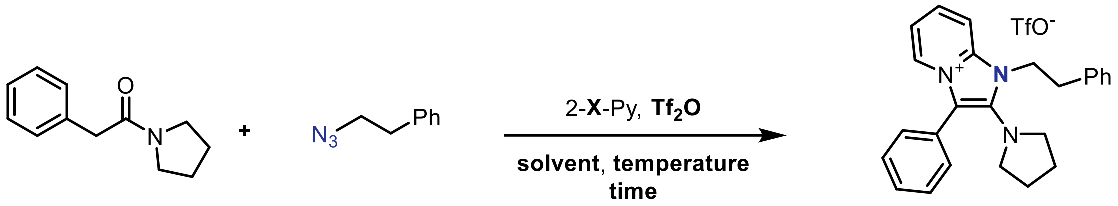

1a **3a** (2.0 equiv.) **4a**

Entry	Solvent	X	Py (equiv.)	Reaction Time (h)	Tf_2O (equiv.)	Temp.	NMR Yield
1	DCM	F	2	0.5	2.0	0 °C to 23 °C	30%
2	$CHCl_3$	F	2	0.5	2.0	0 °C to 23 °C	<10%
3	CH_3NO_2	F	2	0.5	2.0	0 °C to 23 °C	15%
4	DCM	F	3	0.5	1.2	0 °C to 23 °C	24%
5	DCM	F	5	0.5	1.2	0 °C to 23 °C	44%
6	DCM	F	5	1.0	1.2	0 °C to 23 °C	39%
7	DCM	F	5	0.5	1.2	0 °C to 40 °C	16%
8	DCM	F	5	4.0	1.2	0 °C to 23 °C	25%
9	DCM	F	5	0.5	2.0	0 °C to 23 °C	55%
**10**	**DCM**	**Cl**	**5**	**0.5**	**2.0**	0 °C to 23 °C	**79%**
11	DCM	Cl	4	0.5	2.0	0 °C to 23 °C	61%
12	DCM	Cl	3	0.5	2.0	0 °C to 23 °C	55%
13	DCM	Br	5	0.5	2.0	0 °C to 23 °C	45%
14	DCM	Br	3	0.5	2.0	0 °C to 23 °C	36%
15	DCM	I	5	0.5	2.0	0 °C to 23 °C	21%
16	DCM	I	3	0.5	2.0	0 °C to 23 °C	26%

3.2. Optimization for OxoPyrAtes

Table S3. Reaction optimization for the synthesis of OxoPyrAte ***5a****. Reactions conducted on a 0.1 mmol scale. Isolated yields obtained after column chromatography on silica gel (cf.* ***General Procedure F****).*

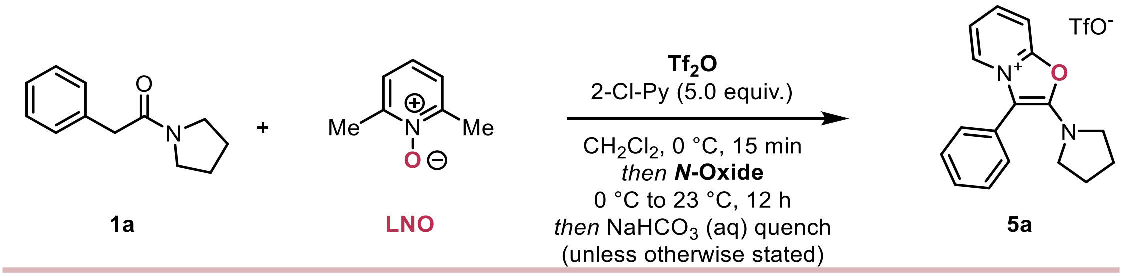

Entry	*N*-oxide	*N*-oxide (equiv.)	Tf_2O (equiv.)	Elaboration	isolated Yield
1	LNO	2.0	2.0	Column chromatography (CC)	35%
2	LNO	2.0	2.0	Et_2O wash, then CC	46%
3	LNO	2.0	2.0	HCl 0.1 M work-up	15%
4	LNO	1.0	2.0	Et_2O wash, then CC	21%
5	LNO	1.5	2.0	Et_2O wash, then CC	50%
6	LNO	1.1	2.0	Et_2O wash, then CC	45%
7	LNO	1.1	1.1	Et_2O wash, then CC	37%
**8**	**LNO**	**1.5**	**2.0**	**CC, then Et_2O wash**	**56%**
9	4-nitropyridine *N*-oxide	1.5	2.0	Et_2O wash, then CC	-
10	LNO (quench after 5 min)	1.5	2.0	CC, then Et_2O wash	17%

4. Reaction mechanisms

4.1. Synthesis of PyrAtes

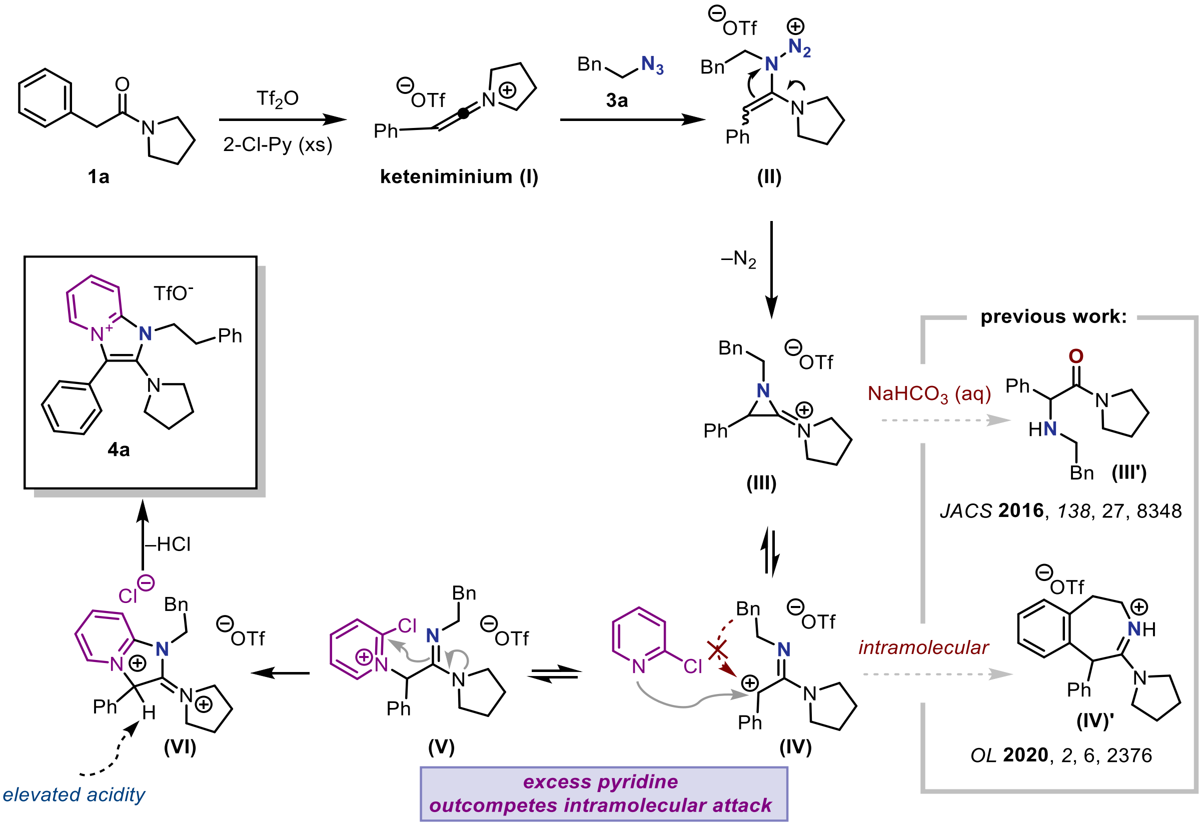

Scheme S1. Plausible mechanism for the synthesis of PyrAtes, highlighting the necessity for excess pyridine base and the challenges that might occur according to literature precedent.

4.2. Synthesis of OxoPyrAtes

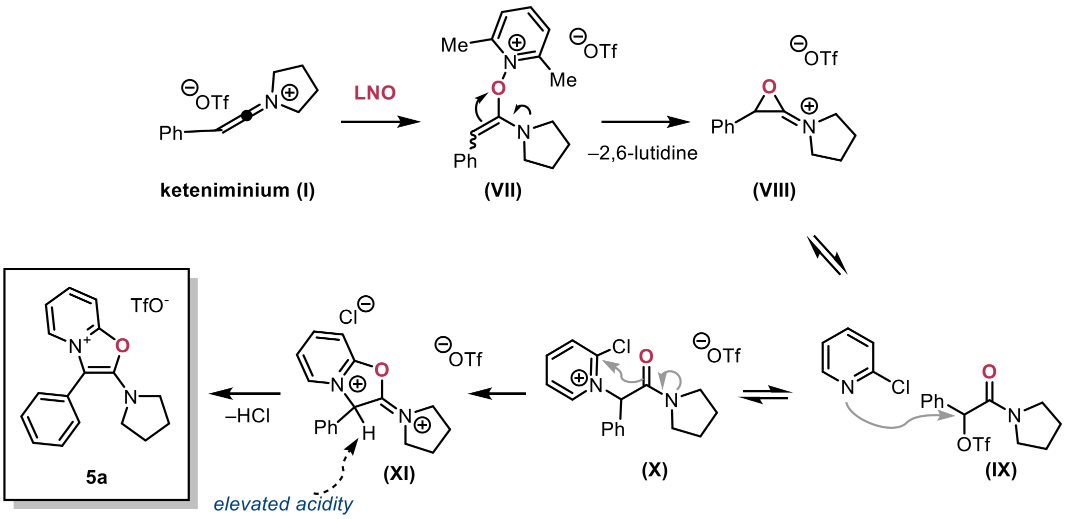

Scheme S2. Plausible mechanism for the synthesis of OxoPyrAtes.[3]

4.3. Synthesis of ThioPyrAtes

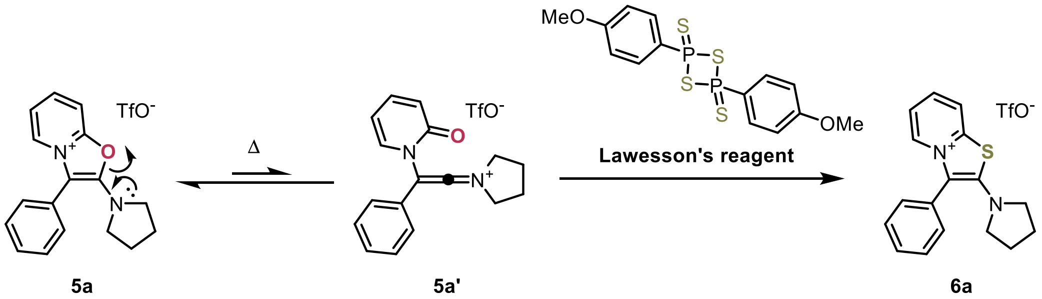

Scheme S3. Mechanistic scenario for the synthesis of ThioPyrAtes from OxoPyrates with Lawesson's reagent.

5. Experimental procedures

5.1. General Procedures

5.1.1. General Procedure A — Synthesis of benzyl carboxamides

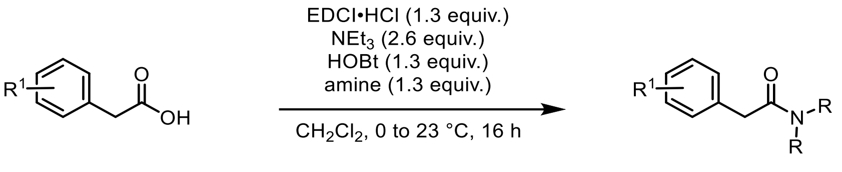

1

Carboxylic acid (1.0 equiv.) was dissolved in dry CH_2Cl_2 (0.1 M) and added to a flame-dried Schlenk tube. Then, 1-(3-dimethylaminopropyl)-3-ethylcarbodiimide hydrochloride (EDCI•HCl, 1.3 equiv.), triethylamine (2.6 equiv.), 1-hydroxybenzotriazole hydrate (HOBt, 1.3 equiv.) and the secondary amine R_2NH (1.3 equiv.) were added at 0 °C. The reaction mixture was slowly allowed to warm to room temperature and stirred for 16 h. The reaction mixture was subsequently diluted with water and extracted with CH_2Cl_2. The collected organic layers were washed successively with saturated aqueous solution of sodium bicarbonate and then brine, dried over anhydrous magnesium sulfate, filtered, and concentrated under reduced pressure. The residue was purified by flash column chromatography on silica gel (heptane/ethyl acetate) to afford the desired amide **1**.

5.1.2. General Procedure B — Synthesis of 2-chloropyridines

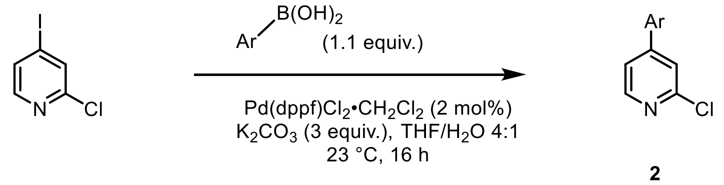

2

2-Chloro-4-iodopyridine (1.0 equiv.), the corresponding aryl boronic acid (1.1 equiv.), [1,1′-bis(diphenylphosphino)ferrocene]dichloropalladium(II) complex with dichloromethane (0.02 equiv.) and K_2CO_3 (3.0 equiv.) were added in a Schlenk tube under argon atmosphere and dissolved in THF (0.1 M), followed by water addition. The mixture was stirred at room temperature for 16 h, before being diluted and extracted with CH_2Cl_2. The organic phase was dried over magnesium sulfate, concentrated and the remaining mixture was purified by column chromatography (silica gel, EtOAc in heptanes 10%) to afford the desired 2-chloropyridine **2**.

5.1.3. General Procedure C — Synthesis of azides

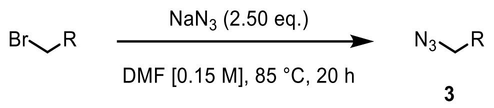

NaN_3 (2.5 equiv.) was added to the stirred solution of alkyl bromide (1.0 equiv.) in DMF at room temperature, and the mixture was subsequently heated to 80 °C for 20 h. Then, the mixture was cooled to room temperature and the salt was filtered off. The filter cake was washed wish Et_2O, the filtrate was diluted with water, extracted with Et_2O (3 times) and the collected organic phases were washed with brine (6 times), dried over anhydrous magnesium sulfate and concentrated to give a crude oil. Purification by column chromatography (silica gel, EtOAc in heptanes) afforded the desired azide **3**.

5.1.4. General Procedure D — Boron-Wittig Olefination

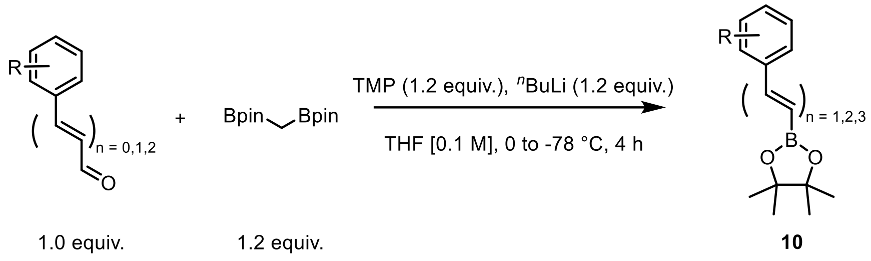

Freshly distilled 2,2,6,6-tetramethylpiperidine (TMP, 1.2 equiv.) was dissolved in THF (0.1 M). The resulting solution was cooled to 0 °C and a solution of *n*-butyllithium (^{n}BuLi, 1.6 M in hexanes, 1.2 equiv.) was added. After 5 min, a solution of bis(4,4,5,5-tetramethyl1,3,2-dioxaborolan-2-yl)methane (1.2 equiv.) in THF (0.1 M) was added. After 5 min, the reaction mixture was cooled to –78 °C and a solution of the aldehyde (1.0 equiv.) in THF (0.1 M) was charged. After 4 hours at –78 °C, the reaction was stopped with addition of a saturated aqueous solution of ammonium chloride and extracted with ethyl acetate. The resulting organic layer was washed with brine, dried over anhydrous magnesium sulfate, filtered, and the filtrate was concentrated *in vacuo*. The crude material was purified directly by column chromatography (SiO_2, EtOAc in heptanes 5% to 20%).

5.1.5. General Procedure E — Synthesis of PyrAtes

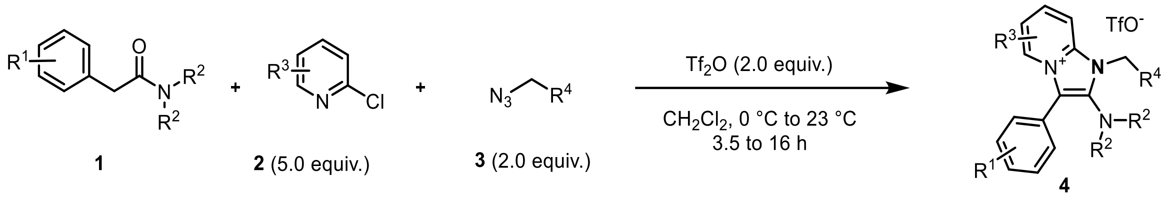

1 **2** (5.0 equiv.) **3** (2.0 equiv.) **4**

Triflic anhydride (2.0 equiv.) was added dropwise to a mixture of the corresponding amide **1** (1.0 equiv., typical scale: 0.2 mmol) and 2-chloropyridine derivative **2** (5.0 equiv.) in CH_2Cl_2 at 0 °C. After 15 min, the azide **3** (2.0 equiv.) was added and the reaction mixture was allowed to reach room temperature. After stirring for 3.5–16 h, $NaHCO_3$ (aq.) was added to the reaction mixture, which was then extracted with CH_2Cl_2. The organic phase was dried over anhydrous magnesium sulfate, concentrated and the resulting brown oil was purified by column chromatography (silica gel, DMA in CH_2Cl_2 0% to 50%) affording the corresponding PyrAte **4**.

5.1.6. General Procedure F — Synthesis of OxoPyrAtes

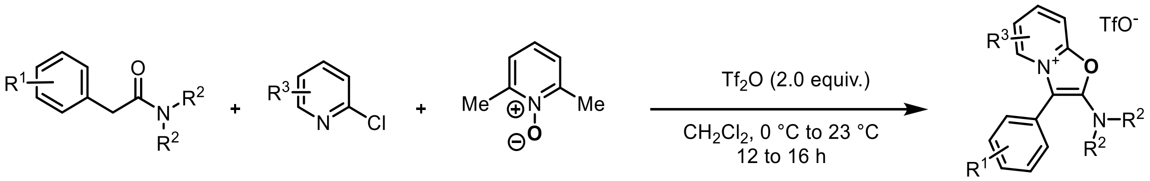

1 **2** (5.0 equiv.) **LNO** (1.5 equiv.) **5**

Triflic anhydride (2 equiv.) was added dropwise to a mixture of the corresponding amide **1** (1 equiv. , typical scale: 0.2 mmol) and 2-chloropyridine derivative **2** (5 equiv.) in CH_2Cl_2 (0.1 M) under argon at 0 °C. The mixture was stirred for 15 minutes, after which lutidine-*N*-oxide (1.5 equiv.) was added and the reaction allowed to warm to room temperature. After 12 to 16 h, the reaction was quenched with a saturated aqueous solution of sodium bicarbonate and the reaction stirred further for 1 h. The mixture was then diluted with CH_2Cl_2, washed once with saturated aqueous solution of sodium bicarbonate. The organic layer was then separated, dried over magnesium sulfate and concentrate under reduced pressure. The product was purified by column chromatography (silica gel, DMA [CH_2Cl_2/MeOH/NH_4OH 90:10:1] in CH_2Cl_2 0% to 100%) and then triturated in Et_2O (in the cases of contaminant LNO-based byproducts) to afford the pure salt **5**.

5.1.7. General Procedure G — Synthesis of ThioPyraAtes

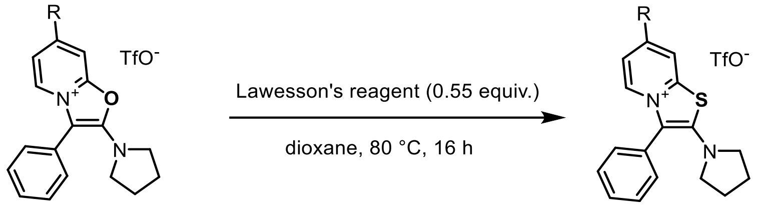

5 6

In a flame-dried Schlenk tube, Oxo-PyrAte **5** (1.0 equiv.) and Lawesson's reagent (0.55 eq.) were dissolved in anhydrous dioxane (0.1 M). The reaction mixture was stirred at 80 °C for 16 h. Then, the solvent was removed under reduced pressure and the resulting mixture was purified by column chromatography (silica gel, DMA [CH_2Cl_2/MeOH/NH_4OH 90:10:1] in CH_2Cl_2 0% to 100%) to afford the desired ThioPyrAte **6**.

5.1.8. General Procedure H — Suzuki-Miyaura Cross-Couplings

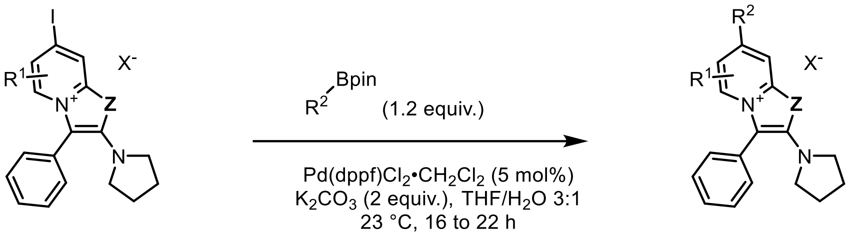

The corresponding pinacol boronic ester (1.2 equiv.), [1,1′-bis(diphenylphosphino)ferrocene]dichloropalladium(II) complex with dichloromethane (0.05 equiv.) and K_2CO_3 (2.0 equiv.) were added in a Schlenk tube under argon atmosphere. Iodo-PyrAte **4w**, **4x**, **4y**, **5f**, or **6b** (1.0 equiv.) dissolved in THF (0.1 M) was added to the mixture, followed by water (0.033 M). The mixture was stirred at room temperature for 16 to 22 h, before being diluted and extracted with CH_2Cl_2. The organic phase was dried over magnesium sulfate, concentrated and the remaining mixture was purified by column chromatography (silica gel, DMA [CH_2Cl_2/MeOH/NH_4OH 90:10:1] in CH_2Cl_2 0% to 60%) to afford the desired cross-coupling product **7**, **8**, or **9**.

5.2. Characterization Data

5.2.1. Amides

2-Phenyl-1-(pyrrolidin-1-yl)ethan-1-one (1a)

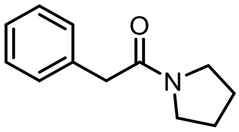

Prepared according to **General Procedure A**. Data are in accordance with literature.[4]

Yield: 1.64 g, 52%.

2-(4-Nitrophenyl)-1-(pyrrolidin-1-yl)ethan-1-one (1b)

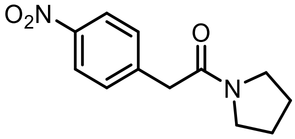

Prepared according to **General Procedure A**. Data are in accordance with literature.[5]

Yield: 926 mg, 79%.

1-(Pyrrolidin-1-yl)-2-(4-((trifluoromethyl)thio)phenyl)ethan-1-one (1c)

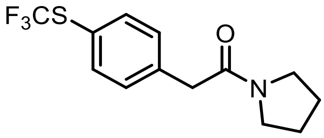

Yield: 419 mg, 48%, colourless oil.

^{1}H NMR (600 MHz, $CDCl_3$): δ 7.60 (d, *J* = 8.1 Hz, 2H), 7.34 (d, *J* = 8.1 Hz, 2H), 3.67 (s, 2H), 3.50 (t, *J* = 5.5 Hz, 2H), 3.45 (t, *J* = 6.8 Hz, 2H), 1.95 (p, *J* = 6.8 Hz, 2H), 1.86 (p, *J* = 6.8 Hz, 2H) ppm.

^{13}C NMR (151 MHz, $CDCl_3$): δ 168.7, 138.4, 136.7 (2C), 130.4 (2C), 129.72 (q, *J* = 308.0 Hz), 122.7 (d, *J* = 2.0 Hz), 47.1, 46.2, 41.8, 26.3, 24.5 ppm.

^{19}F NMR (565 MHz, $CDCl_3$): δ –42.8 ppm.

IR (neat) ν_{max}: 1634, 1434, 1410, 1111, 1080, 510 cm^{-1}.

HRMS (ESI⁺): calculated for $[M+Na]^+$ ($C_{13}H_{14}F_3NOSNa^+$) requires m/z 312.0640, found m/z 312.0640.

2-(4-Methoxyphenyl)-1-(pyrrolidin-1-yl)ethan-1-one (1d)

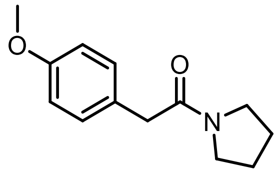

Prepared according to **General Procedure A**. Data are in accordance with literature.[6]

Yield: 758 mg, 69%.

2-(2-Chlorophenyl)-1-(pyrrolidin-1-yl)ethan-1-one (1e)

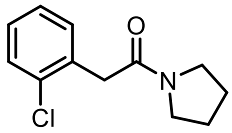

Prepared according to **General Procedure A**. Data are in accordance with literature.[7]

Yield: 890 mg, 80%.

2-(4-Iodophenyl)-1-(pyrrolidin-1-yl)ethan-1-one (1f)

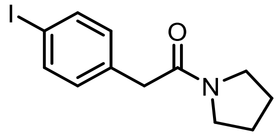

Prepared according to **General Procedure A**. Data are in accordance with literature.[8]

Yield: 2.94 g, 93%.

2-Phenyl-1-(piperidin-1-yl)ethan-1-one (1g)

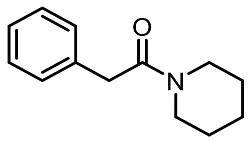

Prepared according to **General Procedure A**. Data are in accordance with literature.[9]

Yield: 358 mg, 82%.

N,N-Diethyl-2-phenylacetamide (1h)

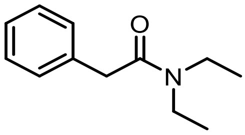

Prepared according to **General Procedure A**. Data are in accordance with literature.[10]

Yield: 330 mg, 86%.

1-(Azetidin-1-yl)-2-phenylethan-1-one (1i)

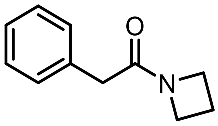

Prepared according to **General Procedure A**. Data are in accordance with literature.[11]

Yield: 505 mg, 29%.

5.2.2. 2-Chloropyridines

2-Chloro-4-(phenanthren-9-yl)pyridine (2b)

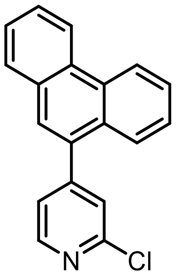

Prepared according to **General Procedure B**.

Yield: 580 mg, 80%, white solid.

^{1}H NMR (600 MHz, $CDCl_3$): δ 8.80 (d, *J* = 8.3 Hz, 1H), 8.74 (d, *J* = 8.3 Hz, 1H), 8.54 (d, *J* = 5.0 Hz, 1H), 7.92 (d, *J* = 7.7 Hz, 1H), 7.81 (d, *J* = 8.2 Hz, 1H), 7.76 – 7.70 (m, 2H), 7.68 (s, 1H), 7.68 – 7.64 (m, 1H), 7.61 – 7.58 (m, 1H), 7.56 (d, *J* = 0.6 Hz, 1H), 7.44 (dd, *J* = 5.0, 1.4 Hz, 1H) ppm.

^{13}C NMR (151 MHz, $CDCl_3$): δ 152.1, 152.0, 149.7, 134.8, 131.0, 130.8, 130.6, 129.6, 129.1, 128.2, 127.7, 127.4, 127.2, 127.2, 126.0, 125.5, 124.1, 123.4, 122.8 ppm.

IR (neat) ν_{max}: 3060, 1711, 1586, 1529, 1494, 1467, 1449, 1420, 1389, 1362, 1235, 1220, 1143, 1119, 1084, 963, 885, 843, 788, 766, 748, 724, 645 cm^{-1}.

HRMS (ESI$^+$): calculated for $[M+H]^+$ ($C_{19}H_{13}ClN^+$) requires m/z 290.0732, found m/z 290.0723.

2-Chloro-4-phenylpyridine (2c)

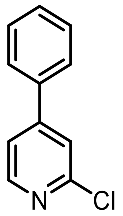

Prepared according to **General Procedure B**.

Yield: 356 mg, 75%, white crystalline.

^{1}H NMR (600 MHz, $CDCl_3$): δ 8.43 (d, *J* = 5.2 Hz, 1H), 7.64 – 7.59 (m, 2H), 7.55 (d, *J* = 1.0 Hz, 1H), 7.52 – 7.46 (m, 3H), 7.44 (dd, *J* = 5.2, 1.6 Hz, 1H) ppm.

^{13}C NMR (151 MHz, $CDCl_3$): δ 152.3, 151.7, 150.2, 137.0, 129.8, 129.4 (2C), 127.2 (2C), 122.2, 120.7 ppm.

IR (neat) ν_{max}: 3058, 1717, 1604, 1587, 1535, 1502, 1460, 1446, 1374, 1225, 1130, 1088, 1052, 988, 878, 844, 804, 760 cm^{-1}.

HRMS (ESI$^+$): calculated for $[M+H]^+$ ($C_{11}H_9ClN^+$) requires m/z 190.0418, found m/z 190.0414.

2-Chloro-4-(4-(trifluoromethyl)phenyl)pyridine (2d)

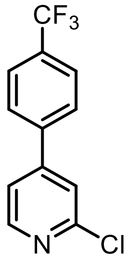

Prepared according to **General Procedure B**. Data are in accordance with literature.[12]

Yield: 652 mg, >99%.

2-chloro-4-(4-fluorophenyl)pyridine (2e)

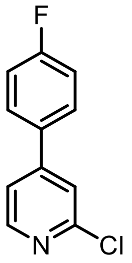

Prepared according to **General Procedure B**. Data are in accordance with literature.[13]

Yield: 390 mg, 75%.

2-Chloro-4-(4-methoxyphenyl)pyridine (2f)

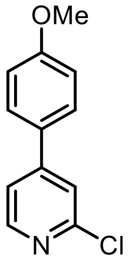

Prepared according to **General Procedure B**. Data are in accordance with literature.[12]

Yield: 437 mg, 80%.

5.2.3. Azides

(2-Azidoethyl)benzene (3a)

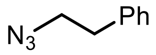

Prepared according to **General Procedure C**. Data are in accordance with literature.[11]

Yield: 2.66 g, 90%.

8-Azidooct-1-ene (3b)

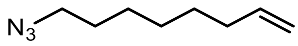

Prepared according to **General Procedure C**. Data are in accordance with literature.[14]

Yield: 3.8 g, 95%.

1,3-Diazidopropane (3c)

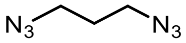

Prepared according to **General Procedure C**. Data are in accordance with literature.[15]

Yield: 262 mg, >99%.

1-(2-Azidoethyl)-4-fluorobenzene (3d)

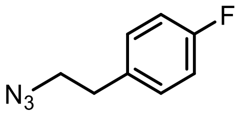

Prepared according to **General Procedure C**. Data are in accordance with literature.[16]

Yield: 275 mg, 83%.

1-(2-Azidoethyl)-4-methylbenzene (3e)

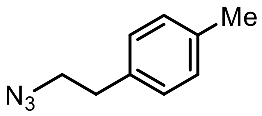

Prepared according to **General Procedure C**. Data are in accordance with literature.[16]

Yield: 300 mg, 93%.

Methyl 7-azidoheptanoate (3f)

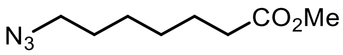

Prepared according to **General Procedure C**. Data are in accordance with literature.[17]

Yield: 1.75 g, 95%.

Methyl 4-azidobutanoate (3g)

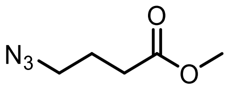

Prepared according to **General Procedure C**. Data are in accordance with literature.[18]

Yield: 1.4 g, 92%.

Benzyl 8-azidooctanoate (3h)

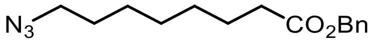

Prepared according to **General Procedure C**. Data are in accordance with literature.[19]

Yield: 490 mg, 35%.

5.2.4. Boronic esters

***N,N*-Diethyl-4'-(4,4,5,5-tetramethyl-1,3,2-dioxaborolan-2-yl)-[1,1'-biphenyl]-4-amine (10a)**

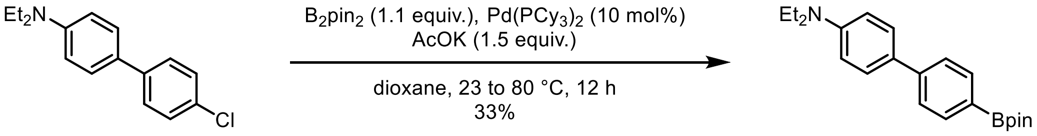

A 25 mL flask assembled a magnetic stirring bar, a septum inlet and a condenser were charged with bis(tricyclohexylphosphine)palladium (67 mg, 0.10 mmol, 0.10 equiv.), and flushed with nitrogen. Dioxane (6 mL) was added, and the resulting mixture was then stirred for 30 min at room temperature. Bis(pinacolato)diboron (0.279 g, 1.1 mmol), KOAc (0.147 g, 1.5 mmol), and aryl chloride (260 mg, 1.0 mmol) were added successively. After being stirred at 80 °C for 16 h, the reaction mixture was treated with water (5 mL) at room temperature. The product was extracted with benzene, washed with brine, and dried over anhydrous magnesium sulfate. Column chromatography afforded the desired product as a white solid (117 mg, 33%).

Yield: 117 mg, 33%, white solid.

^{1}H NMR (600 MHz, $CDCl_3$): δ 7.83 (d, *J* = 8.2 Hz, 2H), 7.57 (d, *J* = 8.2 Hz, 2H), 7.52 (d, *J* = 8.9 Hz, 2H), 6.74 (d, *J* = 8.9 Hz, 2H), 3.40 (q, *J* = 7.1 Hz, 4H), 1.36 (s, 12H), 1.20 (t, *J* = 7.1 Hz, 6H) ppm.

^{13}C NMR (151 MHz, $CDCl_3$): δ 147.5, 144.1, 135.3 (2C), 128.2 (2C), 127.8, 125.4 (2C), 112.0 (2C), 83.8 (2C), 44.6 (2C), 25.0 (4C), 12.8 (2C) ppm. CB carbon is not detected.

IR (neat) $ν_{max}$: 2973, 2929, 1601, 1533, 1504, 1466, 1397, 1355, 1318, 1295, 1267, 1200, 1142, 1092, 1029, 1016, 962, 859 cm^{-1}.

HRMS (ESI$^+$): calculated for $[M+H]^+$ ($C_{22}H_{31}BNO_2^+$) requires m/z 352.2443, found m/z 352.2451.

(*E*)-9-(2-(4,4,5,5-Tetramethyl-1,3,2-dioxaborolan-2-yl)vinyl)-2,3,6,7-tetrahydro-1H,5H-pyrido[3,2,1-ij]quinoline (10b)

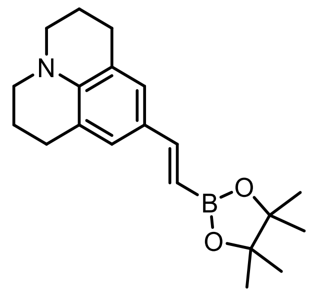

Prepared according to **General Procedure D** using commercially available 9-julolidinecarboxaldehyde.

Yield: 160 mg, 98%, light yellow oil. *Fast decomposition. Must be used immediately*.

^{1}H NMR (400 MHz, $CDCl_3$): δ 7.23 (d, *J* = 18.3 Hz, 1H), 6.94 (s, 2H), 5.82 (d, *J* = 18.3 Hz, 1H), 3.20 – 3.11 (m, 4H), 2.72 (t, *J* = 6.4 Hz, 4H), 1.99 – 1.89 (m, 4H), 1.29 (s, 12H) ppm.

(*E*)-*N*,*N*-Diethyl-4-(2-(4,4,5,5-tetramethyl-1,3,2-dioxaborolan-2-yl)vinyl)aniline (10c)

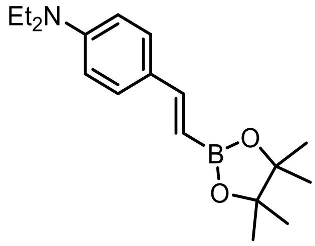

Prepared according to **General Procedure D** using commercially available 4-diethylaminobenzaldehyde.

Yield: 76 mg, 50%, yellow powder.

^{1}H NMR (700 MHz, $CDCl_3$): δ 7.37 (d, *J* = 8.8 Hz, 2H), 7.32 (d, *J* = 18.3 Hz, 1H), 6.61 (d, *J* = 8.9 Hz, 2H), 5.88 (d, *J* = 18.3 Hz, 1H), 3.37 (q, *J* = 7.1 Hz, 4H), 1.30 (s, 12H), 1.17 (t, *J* = 7.1 Hz, 6H) ppm.

^{13}C NMR (176 MHz, $CDCl_3$): δ 150.0, 148.5, 128.8 (2C), 125.1, 111.3 (2C), 83.1 (2C), 44.5 (2C), 25.0 (4C), 12.98 (2C) ppm. CB carbon is not detected.

IR (neat) v_{max}: 2975, 2929, 1600, 1519, 1429, 1377, 1351, 1316, 1269, 1227, 1197, 1184, 1142 cm^{-1}.

HRMS (ESI$^+$): calculated for $[M+H]^+$ ($C_{18}H_{29}BNO_2^+$) requires m/z 302.2286, found m/z 302.2293.

(*E*)-4-(4-(2-(4,4,5,5-Tetramethyl-1,3,2-dioxaborolan-2-yl)vinyl)phenyl)morpholine (10d)

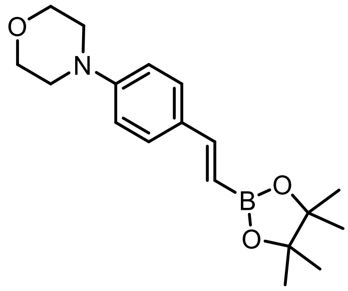

Prepared according to **General Procedure D** using commercially available 4-morpholinobenzaldehyde.

Yield: 126 mg, 80%, yellow-brown powder.

^{1}H NMR (700 MHz, $CDCl_3$): δ 7.42 (d, *J* = 8.8 Hz, 2H), 7.33 (d, *J* = 18.4 Hz, 1H), 6.85 (d, *J* = 8.8 Hz, 2H), 5.99 (d, *J* = 18.4 Hz, 1H), 3.88 – 3.83 (m, 4H), 3.22 – 3.17 (m, 4H), 1.31 (s, 12H) ppm.

^{13}C NMR (176 MHz, $CDCl_3$): δ 151.7, 149.4, 129.3, 128.4 (2C), 115.1 (2C), 83.3 (2C), 66.9 (2C), 48.8 (2C), 25.0 (4C) ppm. CB carbon is not detected.

IR (neat) ν_{max}: 2975, 1612, 1515, 1390, 1360, 1345, 1312, 1292, 1264, 1220, 1213, 1192, 1145, 1118, 1004 cm^{-1}.

HRMS (ESI$^+$): calculated for $[M+H]^+$ ($C_{18}H_{27}BNO_3^+$) requires m/z 316.2079, found m/z 316.2084.

9-((1*E*,3*E*)-4-(4,4,5,5-Tetramethyl-1,3,2-dioxaborolan-2-yl)buta-1,3-dien-1-yl)-2,3,6,7-tetrahydro-1H,5H-pyrido[3,2,1-ij]quinoline (10e)

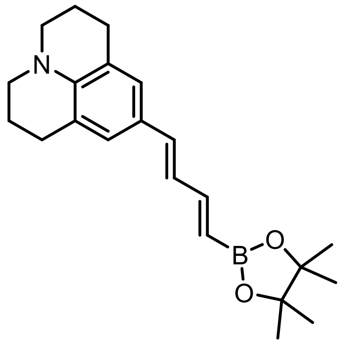

Prepared according to **General Procedure D** using (*E*)-3-(2,3,6,7-tetrahydro-1H,5H-pyrido[3,2,1-ij]quinolin-9-yl)acrylaldehyde which was synthesised based on literature.[20]

Yield: 160 mg, 62%, pale yellow oil. *The product decomposes readily.*

^{1}H NMR (600 MHz, $CDCl_3$): δ 7.15 (dd, *J* = 17.5, 10.3 Hz, 1H), 6.89 (s, 2H), 6.63 (dd, *J* = 15.4, 10.4 Hz, 1H), 6.54 (d, *J* = 15.4 Hz, 1H), 5.50 (d, *J* = 17.5 Hz, 1H), 3.20 – 3.14 (m, 4H), 2.73 (t, *J* = 6.4 Hz, 4H), 2.00 – 1.90 (m, 4H), 1.28 (s, 12H) ppm.

HRMS (ESI$^+$): calculated for $[M+H]^+$ ($C_{22}H_{31}BNO_2^+$) requires m/z 352.2442, found m/z 352.2438.

N,*N*-Diethyl-4-((1*E*,3*E*)-4-(4,4,5,5-tetramethyl-1,3,2-dioxaborolan-2-yl)buta-1,3-dien-1-yl)aniline (10f)

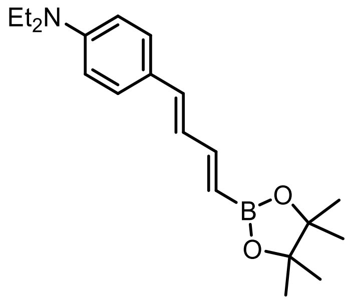

Prepared according to **General Procedure D** using (*E*)-3-(4-(diethylamino)phenyl)acrylaldehyde which was synthesised based on literature.[21]

Yield: 119 mg, 73%, yellow powder.

^{1}H NMR (700 MHz, $CDCl_3$): δ 7.30 (d, *J* = 8.9 Hz, 2H), 7.17 (dd, *J* = 17.5, 9.6 Hz, 1H), 6.67 – 6.60 (m, 4H), 5.52 (d, *J* = 17.5 Hz, 1H), 3.36 (q, *J* = 7.1 Hz, 4H), 1.28 (s, 12H), 1.16 (t, *J* = 7.1 Hz, 6H) ppm.

^{13}C NMR (176 MHz, $CDCl_3$): δ 151.1, 147.9, 137.0, 128.5 (2C), 126.0, 124.1, 111.6 (2C), 83.1 (2C), 44.5 (2C), 24.9(2C), 24.8(2C), 12.8 (2C) ppm. CB carbon not detected.

IR (neat) ν_{max}: 2975, 1590, 1518, 1388, 1354, 1318, 1266, 1192, 1141 cm^{-1}.

HRMS (ESI$^+$): calculated for $[M+H]^+$ ($C_{20}H_{31}BNO_2^+$) requires m/z 328.2443, found m/z 328.2453.

4-(4-((1*E*,3*E*)-4-(4,4,5,5-Tetramethyl-1,3,2-dioxaborolan-2-yl)buta-1,3-dien-1-yl)phenyl)morpholine (10g)

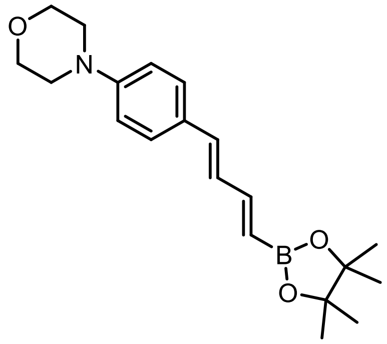

Prepared according to **General Procedure D** using (*E*)-3-(4-morpholinophenyl)acrylaldehyde which was synthesised based on literature.[22]

Yield: 85 mg, 50%, yellow-brown powder.

^{1}H NMR (600 MHz, $CDCl_3$): δ 7.35 (d, *J* = 8.7 Hz, 2H), 7.16 (dd, *J* = 17.5, 10.4 Hz, 1H), 6.85 (d, *J* = 8.8 Hz, 2H), 6.72 (dd, *J* = 15.5, 10.4 Hz, 1H), 6.63 (d, *J* = 15.5 Hz, 1H), 5.59 (d, *J* = 17.5 Hz, 1H), 3.86 (dd, *J* = 10.0, 5.2 Hz, 4H), 3.19 (dd, *J* = 10.0, 5.1 Hz, 4H), 1.29 (s, 12H) ppm.

^{13}C NMR (176 MHz, $CDCl_3$): δ 151.2, 150.5, 136.2, 128.6, 128.2, 128.1 (2C), 115.4 (2C), 83.3 (2C), 66.9 (2C), 48.9 (2C), 24.9 (4C) ppm. CB carbon is not detected.

IR (neat) ν_{max}: 2976, 1738, 1716, 1599, 1366, 1316, 1264, 1226, 1218, 1146, 1122, 1014 cm^{-1}.

HRMS (ESI$^+$): calculated for $[M+Na]^+$ ($C_{20}H_{29}BNO_3Na^+$) requires m/z 342.2235, found m/z 342.2244.

4,4,5,5-Tetramethyl-2-((1*E*,3*E*)-4-(p-tolyl)buta-1,3-dien-1-yl)-1,3,2-dioxaborolane (10h)

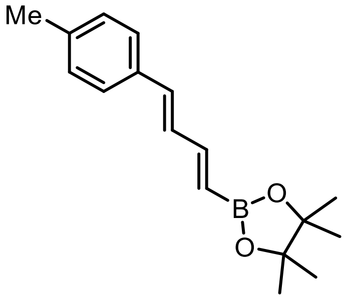

Prepared according to **General Procedure D** using (*E*)-3-(p-tolyl)acrylaldehyde which was synthesised based on literature.[23]

Yield: 131 mg, 48%, yellow solid. Mixture of (*E,E*) and (*E,Z*) isomers in a ratio of 4.8:1. For simplicity, only the major is described below.

^{1}H NMR (600 MHz, CDCl$_3$): δ 7.33 (d, *J* = 8.1 Hz, 2H), 7.17 (dd, *J* = 17.7, 10.6 Hz, 1H), 7.13 (d, *J* = 8.0 Hz, 2H), 6.81 (ddd, *J* = 15.5, 10.4, 0.5 Hz, 1H), 6.67 (d, *J* = 15.6 Hz, 1H), 5.64 (d, *J* = 17.6 Hz, 1H) ppm.

^{13}C NMR (151 MHz, CDCl$_3$): δ 150.2, 138.4, 136.3, 134.2, 129.8, 129.5 (2C), 127.0 (2C), 83.3 (2C), 24.9 (4C), 21.4 ppm. CB carbon is not detected.

IR (neat) ν_{max}: 2978, 2925, 1717, 1626, 1600, 1388, 1359, 1322, 1290, 1260, 1217, 1144, 1128 cm^{-1}.

HRMS (ESI$^+$): calculated for $[M+Na]^+$ ($C_{17}H_{23}BO_2Na^+$) requires m/z 293.1684, found m/z 293.1689.

4,4,5,5-Tetramethyl-2-((1*E*,3*E*,5*E*)-6-(4-(trifluoromethyl)phenyl)hexa-1,3,5-trien-1-yl)-1,3,2-dioxaborolane (10i)

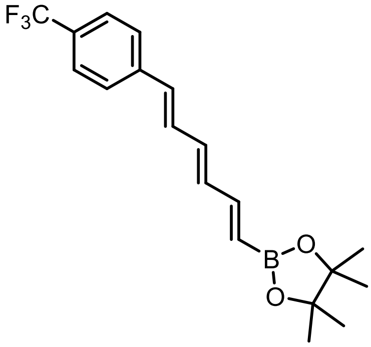

Prepared according to **General Procedure D** using (2*E*,4*E*)-5-(4-(trifluoromethyl)phenyl)penta-2,4-dienalwhich was synthesised based on literature.[24]

Yield: 80 mg, 46%, pale yellow solid.

^{1}H NMR (600 MHz, $CDCl_3$): δ 7.56 (d, *J* = 8.3 Hz, 2H), 7.49 (d, *J* = 8.3 Hz, 2H), 7.13 – 7.05 (m, 1H), 6.90 (dd, *J* = 15.6, 10.1 Hz, 1H), 6.64 (d, *J* = 15.6 Hz, 1H), 6.50 (qd, *J* = 14.9, 10.1 Hz, 2H), 5.66 (d, *J* = 17.6 Hz, 1H), 1.29 (s, 12H) ppm.

^{13}C NMR (151 MHz, $CDCl_3$): δ 149.2, 140.7, 136.5, 135.8, 132.8, 131.2, 129.5 (q, *J* = 32.4 Hz), 126.7 (2C), 125.8 (q, *J* = 3.8 Hz, 2C), 124.3 (q, *J* = 271.9 Hz), 83.5 (2C), 24.9 (4C) ppm. CB carbon is not detected.

^{19}F NMR (565 MHz, $CDCl_3$): δ –62.5 ppm.

IR (neat) ν_{max}: 2980, 1738, 1615, 1588, 1389, 1365, 1323, 1268, 1234, 1217, 1165, 1143 cm^{-1}.

HRMS (ESI$^+$): calculated for $[M+H]^+$ ($C_{19}H_{23}BF_3O_2^+$) requires m/z 351.1738, found m/z 351.1743.

(*E*)-*N*,*N*-Dimethyl-4-(2-(4,4,5,5-tetramethyl-1,3,2-dioxaborolan-2-yl)vinyl)aniline (10j)

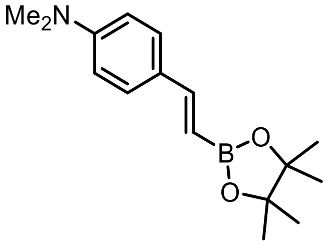

Prepared according to **General Procedure D**. Data are in accordance with literature.[25]

Synthesis of boronic acid pinacol ester 10k

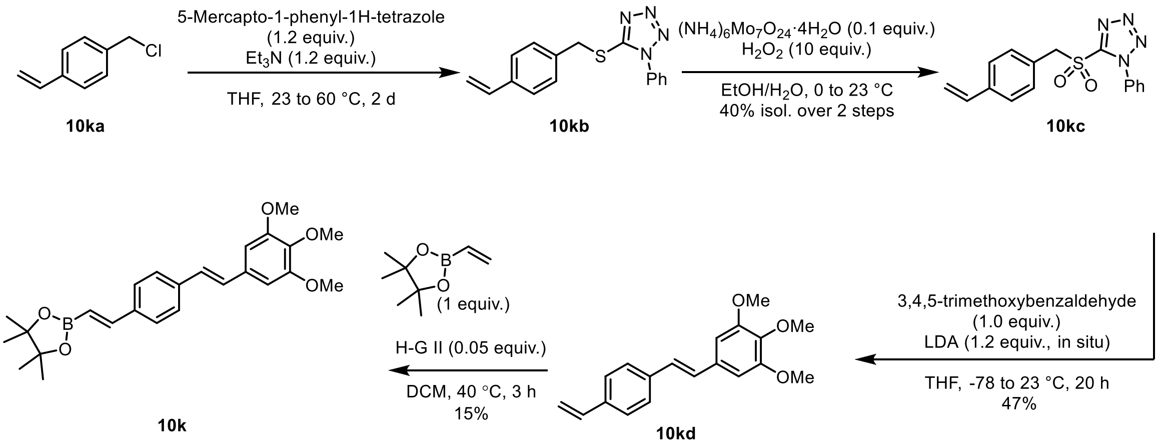

1-Phenyl-5-((4-vinylbenzyl)thio)-1*H*-tetrazole (10kb)

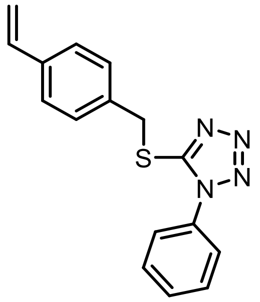

<u>*Representative procedure*</u>: In a flame-dried round bottom flask and under argon atmosphere, 5-mercapto-1-phenyl-1*H*-tetrazole (6.42 g, 36 mmol, 1.2 equiv.), triethylamine (5.02 mL, 36 mmol, 1.2 equiv.) and 4-(chloromethyl)styrene **10ka** (90% w/v) (4.7 mL, 30 mmol, 1.0 equiv.) were dissolved in 150 mL THF at room temperature. The mixture was heated to 60 °C and stirred for 48 h before the addition of saturated aqueous solution of ammonium chloride which was followed by extractions with ethyl acetate (350 mL) and washing of the collected organic phases with brine. The organic layer was dried over anhydrous magnesium sulfate, filtered and concentrated delivering the desired product **10kb** quantitatively, which was used to the next step without further purification.

^{1}H NMR (700 MHz, $CDCl_3$): δ 7.55 – 7.50 (m, 5H), 7.41 – 7.34 (m, 4H), 6.68 (dd, *J* = 17.6, 10.9 Hz, 1H), 5.78 – 5.69 (m, 1H), 5.26 (d, *J* = 10.9 Hz, 1H), 4.62 (s, 2H) ppm.
^{13}C NMR (176 MHz, $CDCl_3$): δ 154.0, 137.7, 136.3, 134.9, 133.8, 130.3, 129.9 (2C), 129.6 (2C), 126.8 (2C), 124.0 (2C), 114.7, 37.6 ppm.
IR (neat) ν_{max}: 3019, 2924, 1737, 1629, 1596, 1498, 1461, 1056, 1041, 1015, 912, 847 cm^{-1}.

HRMS (ESI$^+$): exact mass calculated for $[M+Na]^+$ ($C_{16}H_{14}N_4SNa^+$) requires *m/z* 317.0831, found *m/z* 317.0822.

1-Phenyl-5-((4-vinylbenzyl)sulfonyl)-1*H*-tetrazole (10kc)

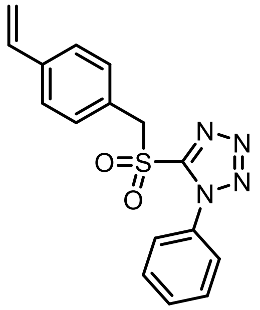

Representative procedure: A 500 mL round bottom flask was charged with sulfide **10kb** (8.83 g, 30 mmol, 1.0 equiv.) and EtOH (300 mL). The solution was cooled to 0 °C and ammonium molybdate tetrahydrate (3.7 g, 3 mmol, 0.1 equiv.) in 35% H_2O_2 (30 mL, 300 mmol, 10 equiv.) was added. The reaction was warmed to room temperature and stirred for 15 h. The reaction mixture was diluted with H_2O (150 mL), the phases were separated and the aqueous phase was extracted with CH_2Cl_2 (3 x 150 mL). The combined organic layers were dried over anhydrous magnesium sulfate, filtrated, and concentrated under reduced pressure. The residue was purified by flash chromatography (silica gel, EtOAc in heptanes 35%) to afford the desired product **10kc**.

Yield: 3.93 g, 40.1% over two steps, light brown powder.

^{1}H NMR (600 MHz, CDCl$_3$): δ 7.56 (t, *J* = 7.5 Hz, 1H), 7.48 (t, *J* = 7.7 Hz, 2H), 7.37 (d, *J* = 8.0 Hz, 2H), 7.32 (d, *J* = 8.1 Hz, 2H), 7.28 (d, *J* = 7.9 Hz, 2H), 6.69 (dd, *J* = 17.6, 10.9 Hz, 1H), 5.78 (d, *J* = 17.6 Hz, 1H), 5.33 (d, *J* = 10.9 Hz, 1H), 4.91 (s, 2H) ppm.

^{13}C NMR (151 MHz, CDCl$_3$): δ 153.1, 139.3, 135.9, 132.9, 131.9 (2C), 131.5, 129.5 (2C), 127.0 (2C), 125.4 (2C), 124.0, 115.9, 62.3 ppm.

IR (neat) ν_{max}: 3066, 3009, 2981, 2919, 1732, 1630, 1595, 1496, 1343, 1154, 759, 687, 619, 516 cm^{-1}.

HRMS (ESI$^+$): exact mass calculated for $[M+Na]^+$ ($C_{16}H_{14}N_4O_2SNa^+$) requires *m/z* 349.0730, found *m/z* 349.0731.

(*E*)-1,2,3-Trimethoxy-5-(4-vinylstyryl)benzene (10kd)

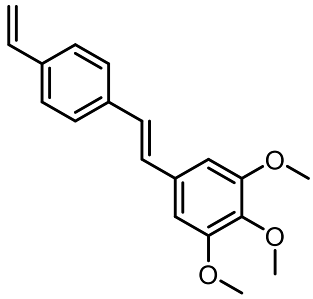

A solution of sulfone **10kc** (898mg, 2.75 mmol, 1.2 equiv.) in THF (0.1 M) was slowly added to a solution of LDA [prepared *in situ* with *n*-BuLi (2.5 M in hexanes, 1.2 mL, 3.0 mmol, 1.2 equiv.) and diisopropylamine (0.425 mL, 3.0 mmol, 1.2 equiv.)] in THF at –78 °C. The mixture was stirred for 30 min at –78 °C. Then, a solution of 3,4,5-trimethoxybenzaldehyde (501 mg, 2.5 mmol, 1.0 equiv.) in THF (0.1 M) was slowly added and the mixture was stirred for another 2 h at –78 °C. The reaction was quenched by saturated aqueous solution of ammonium chloride and extracted with Et_2O (3 times). The combined organic fractions were washed with brine, dried over anhydrous magnesium sulfate,

filtered and concentrated under reduced pressure. The resulting residue was purified by flash column chromatography (silica gel, EtOAc in heptanes 0% to 16%) to afford the desired product **10kd**.

Yield: 345 mg, 47%, pale yellow solid.

^{1}H NMR (600 MHz, CDCl$_3$): δ 7.47 (d, *J* = 8.3 Hz, 2H), 7.41 (d, *J* = 8.2 Hz, 2H), 7.04 (d, *J* = 16.2 Hz, 1H), 6.99 (d, *J* = 16.2 Hz, 1H), 6.78 – 6.68 (m, 3H), 5.77 (d, *J* = 17.5 Hz, 1H), 5.26 (d, *J* = 11.2 Hz, 1H), 3.92 (s, 6H), 3.87 (s, 3H) ppm.

^{13}C NMR (151 MHz, CDCl$_3$): δ 153.6 (2C), 138.2, 137.0, 136.9, 136.6, 133.2, 128.7, 127.9, 126.7 (4C), 113.9, 103.7 (2C), 61.1, 56.3 (2C) ppm.

IR (neat) $ν_{max}$: 3020, 3000, 2958, 2934, 2837, 1732, 1601, 1510, 1453, 1418, 1321, 1185, 1126 cm^{-1}.

HRMS (ESI$^+$): exact mass calculated for $[M+Na]^+$ ($C_{19}H_{20}O_3Na^+$) requires *m/z* 319.1305, found *m/z* 319.1307.

4,4,5,5-Tetramethyl-2-((*E*)-4-((*E*)-3,4,5-trimethoxystyryl)styryl)-1,3,2-dioxaborolane (10k)

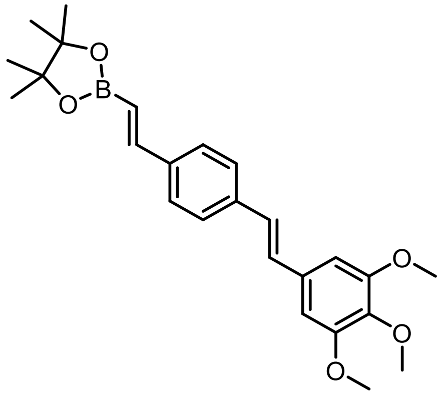

<u>*Representative procedure*</u>: To a solution of olefin **10kd** (148 mg, 0.5 mmol, 1.0 equiv.) in CH_2Cl_2 (0.6 M), was added 4,4,5,5-tetramethyl-2-vinyl-1,3,2-dioxaborolane (85 µL, 0.5 mmol, 1.0 equiv.). The mixture was then added to a suspension of Hoveyda-Grubbs second generation catalyst (15.7 mg, 0.025 mmol, 0.05 equiv.) in CH_2Cl_2 (0.01 M) under argon. The mixture was heated to reflux for 3 h, cooled to room temperature and concentrated under reduced pressure. The residue was purified by column chromatography (silica gel, EtOAc in heptanes 5% to 45%) to afford the desired boronic acid pinacol ester **10k**.

Yield: 32.5 mg,15%, yellow oil.

^{1}H NMR (600 MHz, CDCl$_3$): δ 7.48 (s, 4H), 7.39 (d, *J* = 18.4 Hz, 1H), 7.05 (d, *J* = 16.2 Hz, 1H), 6.99 (d, *J* = 16.2 Hz, 1H), 6.74 (s, 2H), 6.18 (d, *J* = 18.4 Hz, 1H), 3.92 (s, 6H), 3.87 (s, 3H), 1.32 (s, 12H) ppm.

^{13}C NMR (151 MHz, CDCl$_3$): δ 153.6 (2C), 149.1, 138.2, 138.0, 136.9, 133.1, 129.2, 127.8, 127.6 (2C), 126.8 (2C), 116.4, 103.8 (2C), 83.5 (2C), 61.1, 56.3 (2C), 25.0 (4C) ppm.

IR (neat) $ν_{max}$: 2977, 2935, 1622, 1580, 1510, 1419, 1348, 1108, 823 cm^{-1}.

HRMS (ESI$^+$): exact mass calculated for $[M+Na]^+$ ($C_{25}H_{31}BO_5Na^+$) requires *m/z* 445.2157, found *m/z* 445.2163.

5.2.5. PyrAtes, OxoPyrAtes and ThioPyrAtes

1-Phenethyl-3-phenyl-2-(pyrrolidin-1-yl)-1H-imidazo[1,2-a]pyridin-4-ium trifluoromethanesulfonate (4a)

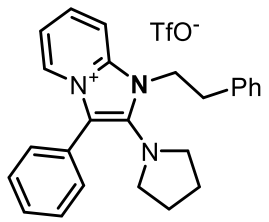

Prepared according to **General Procedure E**.

Yield: 123 mg, 72%, orange solid.

^{1}H NMR (600 MHz, CDCl$_3$): δ 7.84 (d, *J* = 6.8 Hz, 1H), 7.81 (d, *J* = 9.1 Hz, 1H), 7.73 - 7.70 (m, 1H), 7.61 - 7.59 (m, 3H), 7.46 - 7.44 (m, 2H), 7.22 - 7.15 (m, 4H), 7.04 (d, *J* = 6.9 Hz, 2H), 4.76 (t, *J* = 6.9 Hz, 2H), 3.21 (t, *J* = 6.9 Hz, 2H), 3.10 - 3.08 (m, 4H), 1.83 - 1.81 (m, 4H) ppm.

^{13}C NMR (151 MHz, CDCl$_3$): δ 141.0, 137.0, 132.3, 131.8 (2C), 131.2, 130.1 (2C), 128.90 (2C), 128.88 (2C), 127.4, 125.0, 124.6, 122.0, 117.4, 114.2, 111.6, 52.3 (2C), 46.5, 35.0, 25.8 (2C) ppm. Triflate carbon was not detectable by NMR spectroscopy.

^{19}F NMR (565 MHz, CDCl$_3$): δ –78.3 ppm.

IR (neat) ν_{max}: 3060, 2971, 2874, 1647, 1612, 1524, 1266, 1148, 1030, 703, 636 cm^{-1}.

HRMS (ESI$^+$): calculated for $[M–TfO^-]^+$ ($C_{25}H_{26}N_3^+$) requires m/z 368.2121, found m/z 368.2120.

3-(4-Nitrophenyl)-1-phenethyl-2-(pyrrolidin-1-yl)-1H-imidazo[1,2-a]pyridin-4-ium trifluoromethanesulfonate (4b)

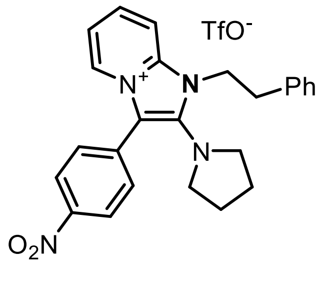

Prepared according to **General Procedure E**.

Yield: 154 mg, 55%, brown solid.

^{1}H NMR (600 MHz, $CDCl_3$): δ 8.38 (d, *J* = 8.7 Hz, 1H), 7.97 (d, *J* = 6.8 Hz, 1H), 7.76 (d, *J* = 8.7 Hz, 1H), 7.72 – 7.68 (m, 1H), 7.65 (d, *J* = 9.1 Hz, 1H), 7.27 – 7.24 (m, 1H), 7.20 (t, *J* = 7.3 Hz, 1H), 7.17 – 7.14 (m, 1H), 7.04 (d, *J* = 7.0 Hz, 1H), 4.62 (t, *J* = 7.0 Hz, 1H), 3.17 (t, *J* = 7.0 Hz, 1H), 3.08 (t, *J* = 6.5 Hz, 2H), 1.85 – 1.81 (m, 2H) ppm.

^{13}C NMR (151 MHz, $CDCl_3$): δ 149.0, 141.3, 136.8, 136.7, 133.1 (2C), 132.9, 131.8, 129.0 (2C), 128.8 (2C), 127.4, 125.0, 124.8 (2C), 122.95 (q, *J* = 323.6 Hz), 118.0, 111.6, 110.9, 52.1 (2C), 46.5, 34.9, 25.8 (2C) ppm.

^{19}F NMR (565 MHz, $CDCl_3$): δ –78.3 ppm.

IR (neat) $ν_{max}$: 1644, 1518, 1347, 1258, 1147, 872, 752, 635, 516 cm^{-1}.

HRMS (ESI$^+$): calculated for $[M–TfO^-]^+$ ($C_{25}H_{25}N_4O_2^+$) requires m/z 413.1973, found m/z 413.1974.

1-Phenethyl-2-(pyrrolidin-1-yl)-3-(4-((trifluoromethyl)thio)phenyl)-1H-imidazo[1,2-a]pyridin-4-ium trifluoromethanesulfonate (4c)

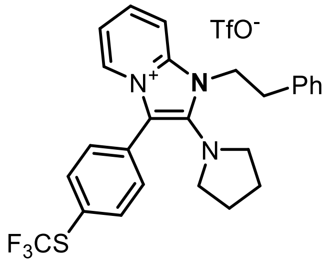

Prepared according to **General Procedure E**.

Yield: 30 mg, 25%, brown oil.

^{1}H NMR (400 MHz, $CDCl_3$): δ 7.90 – 7.82 (m, 3H), 7.75 – 7.66 (m, 2H), 7.60 (d, *J* = 8.2 Hz, 2H), 7.25 – 7.15 (m, 4H), 7.08 – 7.02 (m, 2H), 4.72 (t, *J* = 6.9 Hz, 2H), 3.21 (t, *J* = 6.9 Hz, 2H), 3.15 – 3.09 (m, 4H), 1.88 – 1.80 (m, 4H) ppm.

^{13}C NMR (100 MHz, $CDCl_3$): δ 141.4, 137.1 (2C), 136.9, 133.0 (2C), 132.4, 129.4 (q, *J* = 309.6 Hz), 129.0 (2C), 128.9 (2C), 128.3, 127.8, 127.5, 124.7, 122.4, 117.6, 112.6, 111.4, 52.6 (2C), 46.6, 35.1, 25.9 (2C) ppm. Triflate carbon was not detectable by NMR spectroscopy.

^{19}F NMR (376 MHz, $CDCl_3$): δ –41.8, –78.4 ppm.

IR (neat) $ν_{max}$: 1720, 1651, 1276, 1259, 1224, 1159, 1119, 1083, 1029, 638 cm^{-1}.

HRMS (ESI$^+$): calculated for $[M–TfO^-]^+$ ($C_{26}H_{25}F_3N_3S^+$) requires m/z 468.1716, found m/z 468.1722.

3-(4-Methoxyphenyl)-1-phenethyl-2-(pyrrolidin-1-yl)-1H-imidazo[1,2-a]pyridin-4-ium trifluoromethanesulfonate (4d)

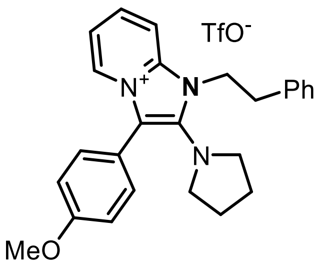

Prepared according to **General Procedure E**.

Yield: 89 mg, 54%, brown oil.

^{1}H NMR (400 MHz, $CDCl_3$): δ 7.85 (d, *J* = 6.8 Hz, 1H), 7.80 (d, *J* = 9.1 Hz, 1H), 7.71 (dd, *J* = 8.6, 7.5 Hz, 1H), 7.32 (d, *J* = 8.6 Hz, 2H), 7.22 – 7.13 (m, 4H), 7.09 (d, *J* = 8.7 Hz, 2H), 7.03 (d, *J* = 7.8 Hz, 2H), 4.71 (t, *J* = 6.8 Hz, 2H), 3.89 (s, 3H), 3.18 (t, *J* = 6.8 Hz, 2H), 3.05 (dd, *J* = 8.6, 4.0 Hz, 4H), 1.85 – 1.77 (m, 4H) ppm.

^{13}C NMR (151 MHz, $CDCl_3$): δ 161.6, 140.8, 136.9, 136.3, 133.1 (2C), 132.4, 128.9 (2C), 128.8 (2C), 127.3, 124.7, 117.5, 116.2, 115.5 (2C), 114.2, 111.3, 55.7, 52.3 (2C), 46.3, 35.0, 25.8 (2C) ppm. Triflate carbon was not detectable by NMR spectroscopy.

^{19}F NMR (565 MHz, $CDCl_3$): δ –78.2 ppm.

IR (neat) ν_{max}: 3507 (broad), 2962, 1645, 1597, 1515, 1453, 1254, 1150, 1030, 751, 637 cm^{-1}.

HRMS (ESI$^+$): calculated for $[M–TfO^-]^+$ ($C_{26}H_{28}N_3O^+$) requires m/z 398.2227, found m/z 398.2229.

3-(2-Chlorophenyl)-1-phenethyl-2-(pyrrolidin-1-yl)-1H-imidazo[1,2-a]pyridin-4-ium trifluoromethanesulfonate (4e)

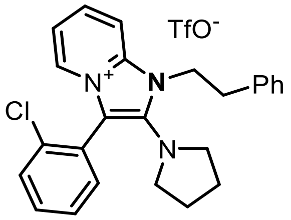

Prepared according to **General Procedure E**.

Yield: 73 mg, 66%, brown oil.

^{1}H NMR (400 MHz, $CDCl_3$): δ 7.96 (d, *J* = 9.1 Hz, 1H), 7.72 – 7.66 (m, 1H), 7.65 – 7.48 (m, 5H), 7.20 – 7.16 (m, 1H), 7.15 – 7.09 (m, 3H), 7.01 – 6.97 (m, 2H), 4.93 (dt, *J* = 13.4, 6.7 Hz, 1H), 4.82 (dt, *J* = 14.9, 6.2 Hz, 1H), 3.23 – 3.16 (m, 2H), 3.14 – 3.04 (m, 4H), 1.85 – 1.76 (m, 4H) ppm.

^{13}C NMR (151 MHz, $CDCl_3$): δ 141.6, 136.9, 136.8, 136.2, 135.1, 133.1, 132.1, 130.5, 129.0 (2C), 128.8 (2C), 128.4, 127.2, 124.43, 124.38, 117.4, 111.8, 110.0, 51.6 (2C), 47.0, 34.8, 25.8 (2C) ppm. Triflate carbon was not detectable by NMR spectroscopy.

^{19}F NMR (376 MHz, CDCl$_3$): δ –78.3 ppm.

IR (neat) ν_{max}: 3405 (broad), 2967, 1646, 1586, 1521, 1275, 1153, 1055, 750, 638 cm^{-1}.

HRMS (ESI$^+$): calculated for [M–TfO$^-$]$^+$ ($C_{25}H_{25}ClN_3^+$) requires m/z 402.1732, found m/z 402.1750.

3-(4-Iodophenyl)-1-phenethyl-2-(pyrrolidin-1-yl)-1H-imidazo[1,2-a]pyridin-4-ium trifluoromethanesulfonate (4f)

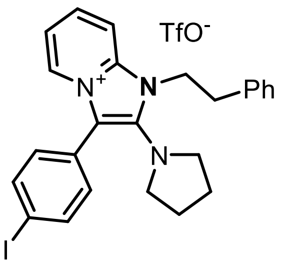

Prepared according to **General Procedure E**.

Yield: 778 mg, 81%, red solid.

^{1}H NMR (600 MHz, CDCl$_3$): δ 7.95 (d, *J* = 8.3 Hz, 2H), 7.84 (d, *J* = 6.8 Hz, 1H), 7.74 – 7.66 (m, 2H), 7.24– 7.16 (m, 6H), 7.05 – 7.00 (m, 2H), 4.69 (t, *J* = 6.9 Hz, 2H), 3.18 (t, *J* = 6.8 Hz, 2H), 3.10 – 3.06 (m, 4H), 1.89 – 1.81 (m, 4H) ppm.

^{13}C NMR (151 MHz, CDCl$_3$): δ 141.0, 139.4 (2C), 136.7, 136.6, 133.3 (2C), 132.5, 129.0 (2C), 128.8 (2C), 127.5, 124.6, 124.4, 117.6, 113.0, 111.2, 98.0, 52.3 (2C), 46.4, 35.0, 25.8 (2C) ppm. Triflate carbon was not detectable by NMR spectroscopy.

^{19}F NMR (565 MHz, CDCl$_3$): δ –78.36 ppm.

IR (neat) ν_{max}: 1736, 1645, 1522, 1489 cm^{-1}.

HRMS (ESI$^+$): calculated for [M–TfO$^-$]$^+$ ($C_{25}H_{25}IN^{+}$) requires m/z 494.1088, found m/z 494.1088.

1-Phenethyl-3-phenyl-2-(piperidin-1-yl)-1H-imidazo[1,2-a]pyridin-4-ium trifluoromethanesulfonate (4g)

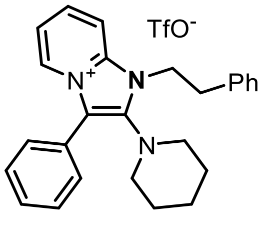

Prepared according to **General Procedure E**.

Yield: 96 mg, 60%, brown solid.

^{1}H NMR (700 MHz, CDCl$_3$): δ 7.93 (d, *J* = 9.1 Hz, 1H), 7.91 (d, *J* = 6.8 Hz, 1H), 7.83 – 7.80 (m, 1H), 7.61 – 7.60 (m, 3H), 7.44 – 7.43 (m, 2H), 7.27 – 7.25 (m, 1H), 7.22 – 7.17 (m, 3H), 7.00 (d, *J* = 7.1 Hz, 2H),

4.68 (t, J = 6.9 Hz, 2H), 3.21 (t, J = 6.9 Hz, 2H), 2.73 – 2.72 (m, 4H), 1.57 – 1.54 (m, 4H), 1.49 – 1.43 (m, 2H) ppm.

^{13}C NMR (175 MHz, CDCl$_3$): δ 142.6, 136.9, 136.6, 133.3, 131.7 (2C), 131.3, 130.0 (2C), 128.91 (2C), 128.90 (2C), 127.3, 125.2, 124.3, 117.6, 117.2, 112.1, 53.3 (2C), 45.5, 35.2, 26.2 (2C), 23.5 ppm. Triflate carbon was not detectable by NMR spectroscopy.

^{19}F NMR (565 MHz, CDCl$_3$): δ –78.3 ppm.

IR (neat) ν_{max}: 3020, 2938, 2850, 1649, 1525, 1150, 1030, 703, 637 cm^{-1}.

HRMS (ESI$^+$): calculated for [M–TfO$^-$]$^+$ ($C_{26}H_{28}N_3^+$) requires m/z 382.2278, found m/z 382.2279.

2-(Diethylamino)-1-phenethyl-3-phenyl-1H-imidazo[1,2-a]pyridin-4-ium trifluoromethanesulfonate (4h)

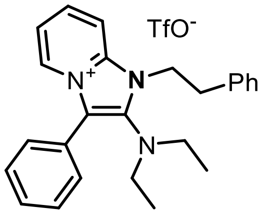

Prepared according to **General Procedure E**.

Yield: 61 mg, 39%, red oil.

^{1}H NMR (600 MHz, CDCl$_3$): δ 7.90 (d, J = 6.8 Hz, 1H), 7.80 – 7.76 (m, 2H), 7.62 – 7.61 (m, 3H), 7.49 – 7.48 (m, 2H), 7.25 – 7.19 (m, 4H), 7.09 (d, J = 7.1 Hz, 2H), 4.67 (t, J = 7.2 Hz, 2H), 3.19 (t, J = 7.2 Hz, 2H), 2.93 (q, J = 7.1 Hz, 4H), 1.04 (t, J = 7.1 Hz, 6H) ppm.

^{13}C NMR (176 MHz, CDCl$_3$): δ 141.0, 136.8 (2C), 133.2, 131.4, 131.4 (2C), 130.1 (2C), 129.1 (2C), 128.9 (2C), 127.4, 125.1, 124.3, 120.8 (q, J = 320.6 Hz, OTf), 118.2, 117.6, 112.0, 47.4 (2C), 45.6, 35.3, 13.4 (2C) ppm.

^{19}F NMR (565 MHz, CDCl$_3$): δ –78.3 ppm.

IR (neat) ν_{max}: 3061, 2972, 2873, 1647, 1525, 1266, 1150, 703, 636 cm^{-1}.

HRMS (ESI$^+$): calculated for [M–TfO$^-$]$^+$ ($C_{25}H_{28}N_3^+$) requires m/z 370.2278, found m/z 370.2278.

2-(Azetidin-1-yl)-1-phenethyl-3-phenyl-1H-imidazo[1,2-a]pyridin-4-ium trifluoroacetate (4i)

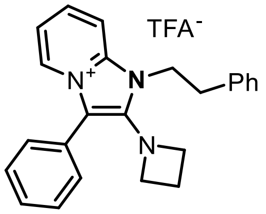

Prepared according to **General Procedure E**.

Yield: 38 mg, 41%, brown oil.

^{1}H NMR (600 MHz, CDCl$_3$): δ 7.76 (d, *J* = 9.1 Hz, 1H), 7.73 (d, *J* = 6.7 Hz, 1H), 7.59 – 7.56 (m, 3H), 7.55 – 7.51 (m, 1H), 7.46 – 7.43 (m, 2H), 7.14 – 7.11 (m, 2H), 7.10 – 7.05 (m, 4H), 4.72 (t, *J* = 6.8 Hz, 2H), 3.96 (t, *J* = 7.5 Hz, 4H), 3.19 (t, *J* = 6.7 Hz, 2H), 2.33 – 2.26 (m, 2H) ppm.

^{13}C NMR (151 MHz, CDCl$_3$): δ 141.3, 136.6, 136.4, 132.3 (2C), 131.2, 131.1, 129.7 (2C), 128.9 (2C), 128.8 (2C), 127.4, 124.6, 123.6, 117.3, 110.9, 107.6, 55.5 (2C), 46.6, 34.2, 18.2 ppm. Triflate carbon was not detectable by NMR spectroscopy.

^{19}F NMR (565 MHz, CDCl$_3$): δ –74.8 ppm.

IR (neat) ν_{max}: 1715, 1688, 1540, 1198, 1119, 751 cm^{-1}.

HRMS (ESI$^+$): calculated for [M–TfO$^-$]$^+$ ($C_{24}H_{24}N_3^+$) requires m/z 354.1965, found m/z 354.1966.

1-Heptyl-3-phenyl-2-(pyrrolidin-1-yl)-1H-imidazo[1,2-a]pyridin-4-ium trifluoromethanesulfonate (4j)

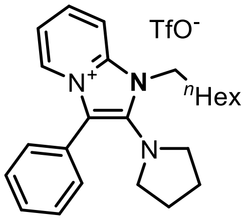

Prepared according to **General Procedure E**.

Yield: 114 mg, 74%, red oil.

^{1}H NMR (700 MHz, CDCl$_3$): δ 7.97 – 7.94 (m, 1H), 7.87 – 7.85 (m, 1H), 7.62 – 7.60 (m, 3H), 7.51 – 7.50 (m, 2H), 7.29 (t, *J* = 6.9 Hz, 1H), 4.42 – 4.40 (m, 2H), 3.20 – 3.18 (m, 4H), 1.92 – 1.88 (m, 1H), 1.87 – 1.85 (m, 4H), 1.44 – 1.40 (m, 2H), 1.38 – 1.34 (m, 2H), 1.30 – 1.28 (m, 4H), 0.87 (t, *J* = 6.9 Hz, 3H) ppm.

^{13}C NMR (175 MHz, CDCl$_3$): δ 140.7, 136.2, 132.8, 131.6 (2C), 131.2, 130.1 (2C), 125.0, 124.9, 117.6, 114.8, 111.2, 52.5 (2C), 44.8, 31.7, 29.2, 28.9, 26.9, 25.9 (2C), 22.7, 14.2 ppm. Triflate carbon was not detectable by NMR spectroscopy.

^{19}F NMR (659 MHz, CDCl$_3$): δ –78.3 ppm.

IR (neat) ν_{max}: 3062, 2928, 2858, 1647, 1526, 1267, 1150, 1031, 637 cm^{-1}.

HRMS (ESI$^+$): calculated for [M–TfO$^-$]$^+$ ($C_{18}H_{19}N_3^+$) requires m/z 362.2591, found m/z 362.2589.

1-(Cyclohexylmethyl)-3-phenyl-2-(pyrrolidin-1-yl)-1H-imidazo[1,2-a]pyridin-4-ium trifluoromethanesulfonate (4k)

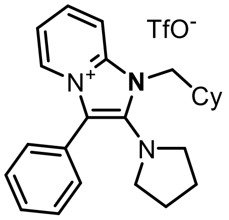

Prepared according to **General Procedure E**.

Yield: 115 mg, 75%, red solid.

^{1}H NMR (700 MHz, CDCl$_3$): δ 7.97 (d, *J* = 6.8 Hz, 1H), 7.95 (d, *J* = 9.1 Hz, 1H), 7.86 – 7.84 (m, 1H), 7.62 – 7.60 (m, 3H), 7.50 – 7.49 (m, 2H), 7.30 (t, *J* = 6.9 Hz, 1H), 4.26 (d, *J* = 7.4 Hz, 2H), 3.18 – 3.16 (m, 4H), 1.92 – 1.88 (m, 1H), 1.85 – 1.83 (m, 4H), 1.74 – 1.73 (m, 2H), 1.66 (s, 1H), 1.61 (d, *J* = 12.7 Hz, 1H), 1.24 – 1.19 (m, 3H), 1.12 – 1.07 (m, 2H) ppm.

^{13}C NMR (175 MHz, CDCl$_3$): δ 141.2, 136.5, 132.8, 131.5 (2C), 131.2, 130.1 (2C), 125.0, 124.7, 120.9 (q, J = 320.9 Hz, OTf), 117.7, 115.3, 111.6, 52.6 (2C), 50.6, 37.9, 30.8 (2C), 26.0, 25.9 (2C), 25.6 (2C) ppm.

^{19}F NMR (659 MHz, CDCl$_3$): δ –78.3 ppm.

IR (neat) ν_{max}: 3061, 2927, 2854, 1646, 1524, 1266, 1149, 1030, 704, 637 cm^{-1}.

HRMS (ESI$^+$): calculated for [M–TfO$^-$]$^+$ ($C_{24}H_{30}N_3^+$) requires m/z 360.2434, found m/z 360.2439.

1-(Oct-7-en-1-yl)-3-phenyl-2-(pyrrolidin-1-yl)-1H-imidazo[1,2-a]pyridin-4-ium trifluoromethanesulfonate (4l)

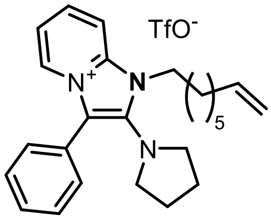

Prepared according to **General Procedure E**.

Yield: 112 mg, 72%, red oil.

^{1}H NMR (600 MHz, CDCl$_3$): δ 7.98 – 7.95 (m, 2H), 7.87 – 7.85 (m, 1H), 7.62 – 7.61 (m, 3H), 7.53 – 7.51 (m, 2H), 7.30 – 7.26 (m, 1H), 5.82 – 5.75 (m, 1H), 4.99 (d, *J* = 17.1 Hz, 1H), 4.94 (d, *J* = 10.1 Hz, 1H), 4.45 – 4.42 (m, 2H), 3.21 – 3.19 (m, 4H), 2.05 – 2.03 (m, 2H), 1.93 – 1.86 (m, 6H), 1.46 – 1.40 (m, 6H) ppm.

^{13}C NMR (151 MHz, CDCl$_3$): δ 140.8, 138.9, 136.3, 132.7, 131.7 (2C), 131.2, 130.1 (2C), 125.0, 124.9, 117.5, 114.7 (2C), 111.3, 52.6 (2C), 44.8, 33.8, 29.2, 28.8, 28.7, 26.8, 25.9 (2C) ppm. Triflate carbon was not detectable by NMR spectroscopy.

IR (neat) ν_{max}: 3076, 2929, 2858, 1646, 1525, 1266, 1149, 1030, 704, 637 cm^{-1}.

HRMS (ESI$^+$): calculated for [M–TfO$^-$]$^+$ ($C_{25}H_{32}N_3^+$) requires m/z 374.2591, found m/z 374.2587.

1-(3-Iodopropyl)-3-phenyl-2-(pyrrolidin-1-yl)-1H-imidazo[1,2-a]pyridin-4-ium trifluoromethanesulfonate (4m)

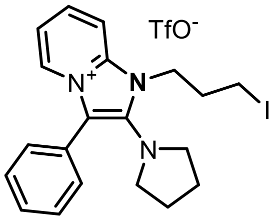

Prepared according to **General Procedure E**.

Yield: 390 mg, 61%, brown solid.

^{1}H NMR (600 MHz, CDCl$_3$): δ 8.18 (d, *J* = 9.1 Hz, 1H), 7.98 (d, *J* = 6.8 Hz, 1H), 7.88 – 7.85 (m, 1H), 7.61 – 7.60 (m, 3H), 7.50 – 7.49 (m, 2H), 7.31 (t, *J* = 6.9 Hz, 1H), 4.57 – 4.52 (m, 2H), 3.34 (t, *J* = 6.1 Hz, 2H), 3.22 – 3.20 (m, 4H), 2.46 – 2.42 (m, 2H), 1.89 – 1.87 (m, 4H) ppm.

^{13}C NMR (151 MHz, CDCl$_3$): δ 140.7, 136.4, 133.1, 131.5 (2C), 131.3, 130.1 (2C), 125.0, 124.6, 117.8, 115.1, 111.4, 52.6 (2C), 44.9, 32.1, 25.8 (2C), 2.7 ppm. Triflate carbon was not detectable by NMR spectroscopy.

^{19}F NMR (565 MHz, CDCl$_3$): δ –78.3 ppm.

IR (neat) ν_{max}: 3079, 2927, 2857, 1649, 1595, 1265, 1148, 1029, 704, 635 cm^{-1}.

HRMS (ESI$^+$): calculated for [M–TfO$^-$]$^+$ ($C_{20}H_{23}IN_3^+$) requires m/z 432.0931, found m/z 432.0931.

1-(3-Azidopropyl)-3-phenyl-2-(pyrrolidin-1-yl)-1H-imidazo[1,2-a]pyridin-4-ium trifluoromethanesulfonate (4n)

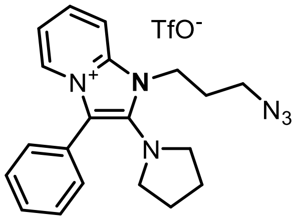

Prepared according to **General Procedure E**.

Yield: 30 mg, 31%, brown solid.

^{1}H NMR (700 MHz, CDCl$_3$): δ 8.16 (d, *J* = 9.1 Hz, 1H), 7.95 (d, *J* = 6.8 Hz, 1H), 7.89 – 7.85 (m, 1H), 7.62 (dd, *J* = 9.4, 6.1 Hz, 3H), 7.54 – 7.50 (m, 2H), 7.30 – 7.26 (m, 1H), 4.58 – 4.54 (m, 2H), 3.61 – 3.57 (m, 2H), 3.22 (t, *J* = 6.6 Hz, 4H), 2.21 (td, *J* = 12.3, 6.5 Hz, 2H), 1.90 – 1.86 (m, 4H) ppm.

^{13}C NMR (176 MHz, CDCl$_3$): δ 140.9, 136.6, 133.0, 131.7 (2C), 131.3, 130.1 (2C), 124.9 (2C), 120.9 (q, *J* = 320 Hz, OTf), 117.7, 114.8, 111.4, 52.6 (2C), 48.7, 42.3, 28.3, 25.9 (2C) ppm.

^{19}F NMR (659 MHz, CDCl$_3$): δ –78.3 ppm.

HRMS (ESI$^+$): calculated for [M–TfO$^-$]$^+$ ($C_{20}H_{23}N_6^+$) requires m/z 347.1979, found m/z 347.1978.

1-(4-Fluorophenethyl)-3-phenyl-2-(pyrrolidin-1-yl)-1H-imidazo[1,2-a]pyridin-4-ium trifluoromethanesulfonate (4o)

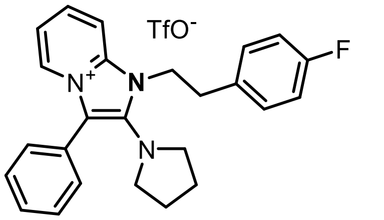

Prepared according to **General Procedure E**.

Yield: 93 mg, 58%, red solid.

^{1}H NMR (700 MHz, CDCl$_3$): δ 7.90 (d, *J* = 9.1 Hz, 1H), 7.85 (d, *J* = 6.6 Hz, 1H), 7.75 (t, *J* = 8.0 Hz, 1H), 7.61 – 7.60 (m, 3H), 7.46 – 7.45 (m, 2H), 7.20 (t, *J* = 6.9 Hz, 1H), 7.08 – 7.06 (m, 2H), 6.92 – 6.90 (m, 2H), 4.73 (t, *J* = 6.8 Hz, 2H), 3.20 (t, *J* = 6.8 Hz, 2H), 3.10 – 3.11 (m, 4H), 1.85 – 1.83 (m, 4H) ppm.

^{13}C NMR (175 MHz, CDCl$_3$): δ 162.1 (d, *J* = 246.1 Hz), 140.9, 136.5, 132.7 (d, *J* = 3.3 Hz), 132.5, 131.7 (2C), 131.3, 130.5 (d, *J* = 7.9 Hz, 2C), 130.1 (2C), 124.9, 124.6, 117.5, 115.7 (d, *J* = 21.4 Hz, 2C), 114.3, 111.7, 52.4 (2C), 46.4, 34.2, 25.8 (2C) ppm. Triflate carbon was not detectable by NMR spectroscopy.

^{19}F NMR (659 MHz, CDCl$_3$): δ –78.3, –115.2 ppm.

IR (neat) ν_{max}: 3052, 2920, 2876, 1647, 1510, 1222, 1151, 1030, 703, 637 cm^{-1}.

HRMS (ESI$^+$): calculated for [M–TfO$^-$]$^+$ ($C_{25}H_{25}FN_3^+$) requires m/z 386.2027, found m/z 386.2031.

1-(4-Methylphenethyl)-3-phenyl-2-(pyrrolidin-1-yl)-1H-imidazo[1,2-a]pyridin-4-ium trifluoromethanesulfonate (4p)

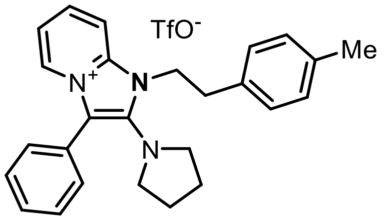

Prepared according to **General Procedure E**.

Yield: 80 mg, 51%, brown solid.

^{1}H NMR (600 MHz, CDCl$_3$): δ 7.85 (d, *J* = 6.8 Hz, 1H), 7.78 (d, *J* = 9.1 Hz, 1H), 7.73 – 7.70 (m, 1H), 7.61 – 7.60 (m, 3H), 7.46 – 7.45 (m, 2H), 7.19 (t, *J* = 6.9 Hz, 1H), 7.02 (d, *J* = 7.7 Hz, 2H), 6.94 (d, *J* = 7.8 Hz, 2H), 4.70 (t, *J* = 7.0 Hz, 2H), 3.16 (t, *J* = 6.9 Hz, 2H), 3.11 – 3.09 (m, 4H), 2.26 (s, 3H), 1.84 – 1.81 (m, 4H) ppm.

^{13}C NMR (151 MHz, CDCl$_3$): δ 140.9, 137.0, 136.5, 133.8, 132.2, 131.7 (2C), 131.2, 130.0 (2C), 129.5 (2C), 128.7 (2C), 125.0, 124.6, 117.4, 114.2, 111.6, 52.3 (2C), 46.5, 34.6, 25.8 (2C), 21.1 ppm. Triflate carbon was not detectable by NMR spectroscopy.

^{19}F NMR (565 MHz, CDCl$_3$): δ –78.3 ppm.

IR (neat) ν_{max}: 3060, 2940, 2874, 1647, 1522, 1267, 1149, 1030, 704, 637 cm^{-1}.

HRMS (ESI$^+$): calculated for $[M–TfO^-]^+$ ($C_{26}H_{28}N_3^+$) requires m/z 382.2278, found m/z 382.2277.

7-(Phenanthren-9-yl)-1-phenethyl-3-phenyl-2-(pyrrolidin-1-yl)-1H-imidazo[1,2-a]pyridin-4-ium trifluoromethanesulfonate (4q)

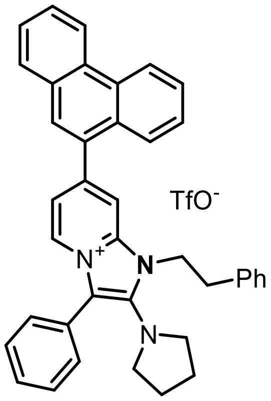

Prepared according to **General Procedure E**.

Yield: 15 mg, 22%, orange solid.

^{1}H NMR (600 MHz, $CDCl_3$): δ 8.77 (d, *J* = 8.3 Hz, 1H), 8.71 (d, *J* = 8.3 Hz, 1H), 8.00 (d, *J* = 7.8 Hz, 1H), 7.92 (d, *J* = 6.9 Hz, 1H), 7.76 – 7.69 (m, 4H), 7.66 (d, *J* = 7.4 Hz, 1H), 7.62 – 7.58 (m, 6H), 7.54 (s, 1H), 7.28 (dd, *J* = 6.9, 0.9 Hz, 1H), 7.19 – 7.17 (m, 2H), 7.14 (t, *J* = 7.2 Hz, 1H), 7.05 (d, *J* = 7.1 Hz, 2H), 4.75 (t, *J* = 6.6 Hz, 2H), 3.22 – 3.19 (m, 6H), 1.86 – 1.84 (m, 4H) ppm.

^{13}C NMR (151 MHz, $CDCl_3$): δ 145.4, 141.2, 137.2, 136.8, 134.1, 132.0 (2C), 131.1, 131.0, 130.8, 130.7, 130.0 (2C), 129.5, 129.4, 129.0 (3C), 128.9 (2C), 127.9, 127.4 (3C), 127.3, 126.1, 125.2, 124.0, 123.3, 122.7, 119.7, 114.4, 110.9, 52.5 (2C), 46.5, 35.2, 25.9 (2C) ppm. Triflate carbon was not detectable by NMR spectroscopy.

^{19}F NMR (565 MHz, $CDCl_3$): δ –78.3 ppm.

IR (neat) ν_{max}: 1650, 1612, 1533, 1269, 1223, 1150, 1030, 753, 702, 636 cm^{-1}.

HRMS (ESI$^+$): calculated for $[M–TfO^-]^+$ ($C_{39}H_{34}N_3^+$) requires m/z 544.2747, found m/z 544.2747.

1-Phenethyl-3,7-diphenyl-2-(pyrrolidin-1-yl)-1H-imidazo[1,2-a]pyridin-4-ium trifluoromethanesulfonate (4r)

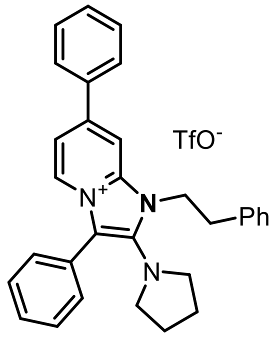

Prepared according to **General Procedure E**.

Yield: 24 mg, 40%.

^{1}H NMR (600 MHz, CDCl$_3$): δ 7.87 (d, *J* = 7.1 Hz, 1H), 7.76 (s, 1H), 7.67 (d, *J* = 7.3 Hz, 2H), 7.61 – 7.60 (m, 3H), 7.50 (t, *J* = 7.4 Hz, 2H), 7.47 – 7.45 (m, 2H), 7.41 (dd, *J* = 7.1, 1.5 Hz, 1H), 7.17 (t, *J* = 7.5 Hz, 2H), 7.10 (t, *J* = 7.4 Hz, 1H), 7.04 (d, *J* = 7.2 Hz, 2H), 4.82 (t, *J* = 6.6 Hz, 2H), 3.21 (t, *J* = 6.5 Hz, 2H), 3.09 – 3.07 (m, 4H), 1.83 – 1.81 (m, 4H) ppm.

^{13}C NMR (151 MHz, CDCl$_3$): δ 145.1, 141.1, 137.3, 137.0, 136.0, 131.6 (2C), 131.2, 130.2, 130.0 (2C), 129.5 (2C), 128.9 (2C), 128.8 (2C), 127.5 (2C), 127.3, 125.0, 124.6, 116.7, 113.8, 107.7, 52.3 (2C), 46.5, 35.1, 25.8 (2C) ppm. Triflate carbon was not detectable by NMR spectroscopy.

^{19}F NMR (565 MHz, CDCl$_3$): δ –78.3 ppm.

IR (neat) ν_{max}: 1650, 1592, 1537, 1499, 1465 ,1263, 1223, 1151, 1030, 764, 719, 658 cm^{-1}.

HRMS (ESI$^+$): calculated for [M–TfO$^-$]$^+$ ($C_{31}H_{30}N_3^+$) requires m/z 444.2434, found m/z 444.2433.

1-Phenethyl-3-phenyl-2-(pyrrolidin-1-yl)-7-(4-(trifluoromethyl)phenyl)-1H-imidazo[1,2-a]pyridin-4-ium trifluoromethanesulfonate (4s)

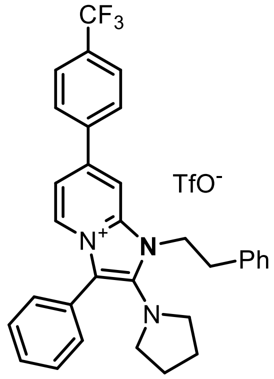

Prepared according to **General Procedure E**.

Yield: 19 mg, 29%.

^{1}H NMR (600 MHz, CDCl$_3$): δ 7.89 – 7.87 (m, 2H), 7.82 (d, *J* = 8.2 Hz, 2H), 7.73 (d, *J* = 8.2 Hz, 2H), 7.62-7.60 (m, 3H), 7.47 – 7.44 (m, 2H), 7.40 (d, *J* = 8.2 Hz, 1H), 7.15 (t, *J* = 7.5 Hz, 2H), 7.08 (t, *J* = 7.4 Hz,

1H), 7.04 (d, *J* = 7.2 Hz, 2H), 4.86 (t, *J* = 6.5 Hz, 2H), 3.20 (t, *J* = 6.5 Hz, 2H), 3.10 – 3.08 (m, 4H), 1.84 – 1.81 (m, 4H) ppm.

^{13}C NMR (151 MHz, CDCl$_3$): δ 143.1, 141.4, 139.4, 137.2, 136.8, 131.8 (q, *J* = 98.2 Hz), 131.6 (2C), 131.3, 130.1 (2C), 129.0 (2C), 128.8 (2C), 128.0 (2C), 127.4, 126.4 (q, *J* = 2.5 Hz, 2C), 124.9, 124.7, 123.9 (d, *J* = 272.2 Hz, CCF$_3$), 120.9 (q, *J* = 320.7 Hz, OTf), 116.5, 113.8, 108.7, 52.3 (2C), 46.7, 35.1, 25.8 (2C) ppm.

^{19}F NMR (565 MHz, CDCl$_3$): δ –62.7, –78.3 ppm.

IR (neat) ν_{max}: 1650, 1592, 1537, 1325, 1260, 1223, 1154, 1120, 1070, 1030, 702, 637 cm^{-1}.

HRMS (ESI$^+$): calculated for [M–TfO$^-$]$^+$ ($C_{32}H_{29}F_3N_3^+$) requires m/z 512.2308, found m/z 512.2308.

7-(4-Fluorophenyl)-1-phenethyl-3-phenyl-2-(pyrrolidin-1-yl)-1H-imidazo[1,2-a]pyridin-4-ium trifluoromethanesulfonate (4t)

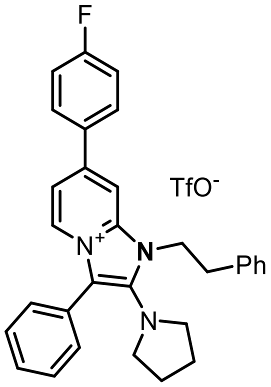

Prepared according to **General Procedure E**.

Yield: 32 mg, 52%.

^{1}H NMR (600 MHz, CDCl$_3$): δ 7.85 (d, *J* = 7.1 Hz, 1H), 7.80 (s, 1H), 7.71 – 7.69 (m, 2H), 7.62 – 7.60 (m, 3H), 7.43 – 7.39 (m, 3H), 7.16 – 7.10 (m, 4H), 7.06 (t, *J* = 7.4 Hz, 1H), 7.02 (d, *J* = 7.1 Hz, 2H), 4.82 (t, *J* = 6.5 Hz, 2H), 3.19 (t, *J* = 6.5 Hz, 2H), 3.06 – 3.05 (m, 4H), 1.83 – 1.81 (m, 4H) ppm.

^{13}C NMR (151 MHz, CDCl$_3$): δ 163.9 (d, *J* = 251.0 Hz), 143.8, 141.0, 137.2, 136.9, 131.5 (2C), 131.9 (d, *J* = 3.1 Hz), 131.2, 130.1 (2C), 129.6 (d, *J* = 8.6 Hz. 2C), 128.9 (2C), 128.7 (2C), 127.3, 124.8, 124.6, 116.5, 116.4 (d, *J* = 22.1 Hz, 2C), 113.8, 107.6, 52.2 (2C), 46.4, 35.1, 25.8 (2C) ppm. Triflate carbon was not detectable by NMR spectroscopy.

^{19}F NMR (565 MHz, CDCl$_3$): δ –78.3, –110.5 ppm.

IR (neat) ν_{max}: 2366, 1650, 1594, 1537, 1445, 1261, 1153, 1058, 842, 75, 609 cm^{-1}.

HRMS (ESI$^+$): calculated for [M–TfO$^-$]$^+$ ($C_{31}H_{29}FN_3^+$) requires m/z 462.2340, found m/z 462.2338.

7-(4-Methoxyphenyl)-1-phenethyl-3-phenyl-2-(pyrrolidin-1-yl)-1H-imidazo[1,2-a]pyridin-4-ium trifluoromethanesulfonate (4u)

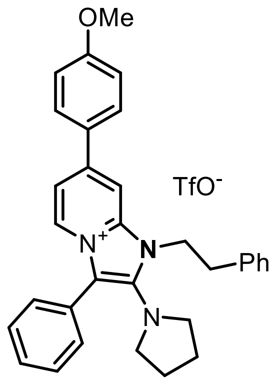

Prepared according to **General Procedure E**.

Yield: 13 mg, 21%.

^{1}H NMR (600 MHz, CDCl$_3$): δ 7.81 (d, *J* = 7.1 Hz, 1H), 7.78 (s, 1H), 7.68 (d, *J* = 8.8 Hz, 2H), 7.61 – 7.59 (m, 3H), 7.44 – 7.43 (m, 2H), 7.38 (dd, *J* = 7.1, 1.6 Hz, 1H), 7.19 – 7.16 (m, 2H), 7.10 (t, *J* = 7.4 Hz, 1H), 7.05 – 7.02 (m, 4H), 4.82 (t, *J* = 6.5 Hz, 2H), 3.88 (s, 3H), 3.22 (t, *J* = 6.5 Hz, 2H), 3.06 – 3.03 (m, 4H), 1.82 – 1.80 (m, 4H) ppm.

^{13}C NMR (151 MHz, CDCl$_3$): δ 161.6, 144.9, 140.9, 137.3, 137.2, 131.6 (2C), 131.1, 130.0 (2C), 129.0 (4C), 128.8 (2C), 128.0, 127.3, 125.1, 124.4, 116.2, 115.0 (2C), 113.7, 106.6, 55.7, 52.3 (2C), 46.3, 35.1, 25.8 (2C) ppm. Triflate carbon was not detectable by NMR spectroscopy.

^{19}F NMR (565 MHz, CDCl$_3$): δ –78.3 ppm.

IR (neat) ν_{max}: 1650, 1606 ,1536, 1467, 1261, 1182, 1151, 1030, 837, 705, 637 cm^{-1}.

HRMS (ESI$^+$): calculated for [M–TfO$^-$]$^+$ ($C_{32}H_{32}N_3O^+$) requires m/z 474.2540, found m/z 474.2539.

7-Iodo-1-(7-methoxy-7-oxoheptyl)-3-phenyl-2-(pyrrolidin-1-yl)-1H-imidazo[1,2-a]pyridin-4-ium trifluoromethanesulfonate (4v)

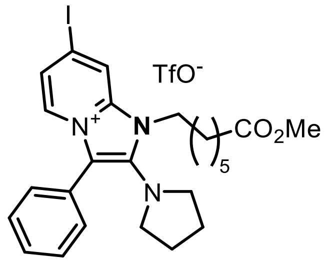

Prepared according to **General Procedure E**.

Yield: 299 mg, 88%, brown oil.

^{1}H NMR (400 MHz, CDCl$_3$): δ 8.17 – 8.15 (m, 1H), 7.66 (d, *J* = 7.1 Hz, 1H), 7.62 – 7.57 (m, 3H), 7.56 – 7.51 (m, 2H), 7.48 (dd, *J* = 7.1, 1.5 Hz, 1H), 4.36 (t, *J* = 7.9 Hz, 2H), 3.67 (s, 3H), 3.23 – 3.17 (m, 4H), 2.33 (t, *J* = 7.3 Hz, 2H), 1.94 – 1.80 (m, 6H), 1.68 – 1.61 (m, 2H), 1.50 – 1.39 (m, 4H) ppm.

^{13}C NMR (100 MHz, CDCl$_3$): δ 174.2, 140.8, 136.2, 131.8 (2C), 131.3, 130.1 (2C), 126.6, 125.0, 124.7, 120.8 (q, *J* = 320.8 Hz, OTf), 119.3, 114.9, 97.5, 52.5 (2C), 51.7, 44.9, 33.9, 28.9, 28.6, 26.6, 25.9 (2C), 24.7 ppm.

^{19}F NMR (565 MHz, CDCl$_3$): δ –78.2 ppm.

IR (neat) ν_{max}: 2950, 2860, 1730, 1636, 1274, 1028, 635 cm^{-1}.

HRMS (ESI$^+$): calculated for [M–TfO$^-$]$^+$ ($C_{25}H_{31}IN_3O_2^+$) requires m/z 532.1455, found m/z 532.1469.

7-Iodo-1-(4-methoxy-4-oxobutyl)-3-phenyl-2-(pyrrolidin-1-yl)-1*H*-imidazo[1,2-a]pyridin-4-ium trifluoromethanesulfonate (4w)

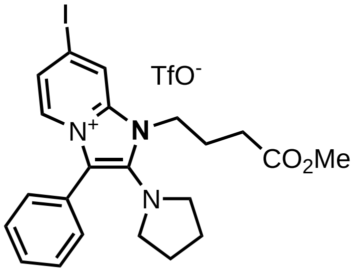

Prepared according to **General Procedure E**.

Yield: 410 mg, 64%, brown powder.

^{1}H NMR (600 MHz, CDCl$_3$): δ 8.36 (d, *J* = 0.7 Hz, 1H), 7.68 (d, *J* = 7.1 Hz, 1H), 7.57 (dd, *J* = 4.1, 2.3 Hz, 3H), 7.55 – 7.51 (m, 2H), 7.49 (d, *J* = 7.0 Hz, 1H), 4.48 – 4.35 (m, 2H), 3.66 (s, 3H), 3.18 (dd, *J* = 7.7, 5.5 Hz, 4H), 2.53 (t, *J* = 6.5 Hz, 2H), 2.20 – 2.12 (m, 2H), 1.88 – 1.77 (m, 4H) ppm.

^{13}C NMR (151 MHz, CDCl$_3$): δ 173.2, 140.8, 136.3, 131.7 (2C), 131.3, 130.0 (2C), 126.7, 124.9, 124.6, 120.8 (q, *J* = 321.0 Hz, OTf), 119.5, 114.8, 98.0, 52.4 (2C), 52.0, 43.9, 30.4, 25.8 (2C), 24.0 ppm.

^{19}F NMR (565 MHz, CDCl$_3$): δ –78.2 ppm.

IR (neat) ν_{max}: 1730, 1638, 1444, 1261, 1223, 1152, 1030, 637 cm^{-1}.

HRMS (ESI$^+$): exact mass calculated for [M–TfO$^-$]$^+$ ($C_{22}H_{25}IN_3O_2^+$) requires *m/z* 490.0986, found *m/z* 490.0987.

1-(3-Azidopropyl)-7-iodo-3-phenyl-2-(pyrrolidin-1-yl)-1H-imidazo[1,2-a]pyridin-4-ium trifluoromethanesulfonate (4x)

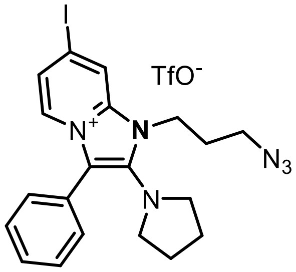

Prepared according to **General Procedure E**.

Yield: 31 mg, 25%. The title compound was used directly at the next step after semi-purification with column chromatography.

^{1}H NMR (700 MHz, CDCl$_3$): δ 8.36 (s, 1H), 7.64 (d, *J* = 7.1 Hz, 1H), 7.62 – 7.59 (m, 3H), 7.55 – 7.53 (m, 2H), 7.49 (dd, *J* = 7.1, 1.4 Hz, 1H), 4.49 (t, *J* = 7.3 Hz, 2H), 3.63 – 3.51 (m, 2H), 3.23 (t, *J* = 6.6 Hz, 4H), 2.23 – 2.17 (m, 2H), 1.88 – 1.80 (m, 4H) ppm.

7-Iodo-1-phenethyl-3-phenyl-2-(pyrrolidin-1-yl)-1H-imidazo[1,2-a]pyridin-4-ium trifluoromethanesulfonate (4y)

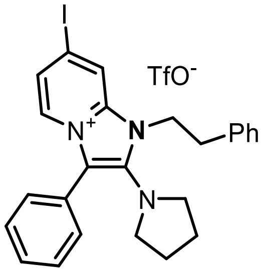

Prepared according to **General Procedure E**.

Yield: 325 mg, 50%, brown solid.

^{1}H NMR (600 MHz, CDCl$_3$): δ 7.71 (s, 1H), 7.59 –7.58 (m, 3H), 7.53 (t, *J* = 7.0 Hz, 1H), 7.49 – 7.47 (m, 2H), 7.35 (dd, *J* = 7.0, 1.3 Hz, 1H), 7.25 – 7.21 (m, 3H), 7.06 (d, *J* = 6.9 Hz, 2H), 4.67 (t, *J* = 6.6 Hz, 2H), 3.18 (t, *J* = 6.6 Hz, 2H), 3.15 – 3.13 (m, 4H), 1.83 – 1.81 (m, 4H) ppm.

^{13}C NMR (151 MHz, CDCl$_3$): δ 140.8, 137.0, 136.4, 131.8 (2C), 131.3, 130.0 (2C), 129.0 (2C), 128.9 (2C), 127.7, 126.3, 124.7, 124.5, 119.8, 114.3, 97.0, 52.4 (2C), 46.9, 35.2, 25.9 (2C) ppm. Triflate carbon was not detectable by NMR spectroscopy.

^{19}F NMR (565 MHz, CDCl$_3$): δ –78.3 ppm.

IR (neat) ν_{max}: 2362, 1642, 1589, 1530, 1429, 1272, 1223, 1153, 1029, 703, 637 cm^{-1}.

HRMS (ESI$^+$): calculated for $[M–TfO^-]^+$ ($C_{25}H_{25}IN_3^+$) requires m/z 494.1088, found m/z 494.1095.

3-Heptyl-1-phenyl-2-(pyrrolidin-1-yl)-3H-imidazo[1,2-a]quinolin-10-ium trifluoromethanesulfonate (4z)

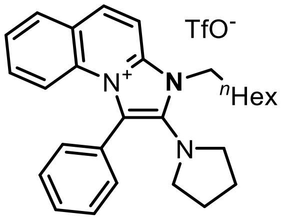

Prepared according to **General Procedure E**.

Yield: 80 mg, 71%, brown oil.

^{1}H NMR (600 MHz, CDCl$_3$): δ 8.24 (d, *J* = 9.5 Hz, 1H), 8.01 (d, *J* = 9.5 Hz, 1H), 7.98 (dd, *J* = 8.0, 1.0 Hz, 1H), 7.73 – 7.69 (m, 1H), 7.66 (t, *J* = 7.5 Hz, 2H), 7.64 – 7.60 (m, 2H), 7.56 (dd, *J* = 11.2, 3.9 Hz, 1H), 7.39 (ddd, *J* = 8.7, 7.2, 1.4 Hz, 1H), 7.27 (d, *J* = 7.7 Hz, 1H), 4.53 – 4.49 (m, 2H), 3.11 (t, *J* = 6.5 Hz, 4H), 1.92 (dt, *J* = 15.3, 7.7 Hz, 2H), 1.76 – 1.72 (m, 4H), 1.48 – 1.43 (m, 2H), 1.41 – 1.35 (m, 2H), 1.31 – 1.27 (m, 4H), 0.88 (t, *J* = 6.9 Hz, 3H) ppm.

^{13}C NMR (151 MHz, CDCl$_3$): δ 140.4, 136.0, 135.1, 132.8, 132.1 (2C), 131.6, 131.1, 130.8, 130.2 (2C), 128.1, 127.3, 125.2, 121.0, 120.9 (q, *J* = 320.8 Hz, OTf), 117.3, 109.4, 53.2 (2C), 44.5, 31.7, 29.9, 28.9, 26.9, 25.9 (2C), 22.7, 14.2 ppm.

^{19}F NMR (565 MHz, CDCl$_3$): δ –78.3 ppm.

IR (neat) $ν_{max}$: 2954, 2358, 1609, 1264, 1223, 1030, 637 cm^{-1}.

HRMS (ESI$^+$): calculated for [M–TfO$^-$]$^+$ ($C_{28}H_{34}N_3^+$) requires m/z 412.2748, found m/z 412.2748.

3-(8-(Benzyloxy)-8-oxooctyl)-1-phenyl-2-(pyrrolidin-1-yl)-3H-imidazo[1,2-a]quinolin-10-ium trifluoromethanesulfonate (4aa)

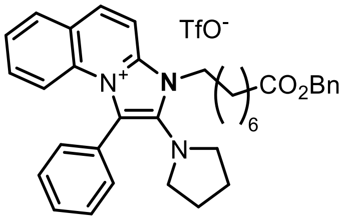

Prepared according to **General Procedure E**.

Yield: 92 mg, 66%, brown oil.

^{1}H NMR (600 MHz, CDCl$_3$): δ 8.24 (d, *J* = 9.5 Hz, 1H), 8.02 (d, *J* = 9.5 Hz, 1H), 7.98 (dd, *J* = 8.0, 1.3 Hz, 1H), 7.73 – 7.69 (m, 1H), 7.66 (t, *J* = 7.6 Hz, 2H), 7.63 – 7.60 (m, 2H), 7.58 – 7.54 (m, 1H), 7.39 (ddd, *J* = 8.8, 7.2, 1.5 Hz, 1H), 7.35 – 7.30 (m, 5H), 7.27 (d, *J* = 7.7 Hz, 1H), 5.09 (s, 2H), 4.53 – 4.46 (m, 2H), 3.10 (t, *J* = 6.5 Hz, 4H), 2.35 (t, *J* = 7.4 Hz, 2H), 1.91 (dt, *J* = 15.3, 7.7 Hz, 2H), 1.75 – 1.72 (m, 4H), 1.64 (dt, *J* = 14.9, 7.5 Hz, 2H), 1.46 (dt, *J* = 15.0, 6.9 Hz, 2H), 1.42 – 1.37 (m, 2H), 1.37 – 1.31 (m, 2H) ppm.

^{13}C NMR (151 MHz, CDCl$_3$): δ 173.7, 140.4, 136.2, 136.0, 135.1, 132.8, 132.1 (2C), 131.6, 131.1, 130.8, 130.2 (2C), 128.7 (2C), 128.3 (3C), 128.1, 127.3, 125.2, 121.0, 120.8 (q, *J* = 320.6 Hz, OTf), 117.3, 109.5, 66.2, 53.2 (2C), 44.4, 34.3, 29.8, 29.0, 28.9, 26.7, 25.9 (2C), 24.8 ppm.

^{19}F NMR (565 MHz, CDCl$_3$): δ –78.3 ppm.

IR (neat) $ν_{max}$: 2934, 1732, 1630, 1264, 1151, 1030, 637 cm^{-1}.

HRMS (ESI$^+$): calculated for [M–TfO$^-$]$^+$ ($C_{36}H_{40}N_3O_2^+$) requires m/z 546.3115, found m/z 546.3110.

1-(Cyclohexylmethyl)-3-phenyl-2-(pyrrolidin-1-yl)-1H-imidazo[2,1-a]isoquinolin-4-ium trifluoromethanesulfonate (4ab)

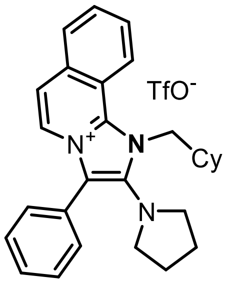

Prepared according to **General Procedure E**.

Yield: 49 mg, 44%, red powder.

^{1}H NMR (400 MHz, CDCl$_3$): δ 8.47 (d, *J* = 8.4 Hz, 1H), 8.03 – 7.97 (m, 1H), 7.96 – 7.90 (m, 1H), 7.88 – 7.82 (m, 1H), 7.78 (d, *J* = 7.2 Hz, 1H), 7.66 – 7.62 (m, 3H), 7.59 (d, *J* = 7.3 Hz, 1H), 7.57 – 7.54 (m, 2H), 4.66 (d, *J* = 7.2 Hz, 2H), 3.18 (t, *J* = 6.5 Hz, 4H), 1.98 (brs, 1H), 1.87 – 1.80 (m, 4H), 1.78 – 1.61 (m, 6H), 1.22 – 1.13 (m, 4H) ppm.

^{13}C NMR (151 MHz, CDCl$_3$): δ 141.2, 133.4, 132.7, 131.8, 131.6 (2C), 131.4, 130.5, 130.2 (2C), 129.2, 124.6, 123.9, 120.8, 120.8 (q, *J* = 320.8 Hz, OTf), 119.4, 118.5, 117.9, 53.5 (2C), 51.3, 37.2, 30.6 (2C), 26.0, 25.9 (2C), 25.6 (2C) ppm.

^{19}F NMR (565 MHz, CDCl$_3$): δ –78.3 ppm.

IR (neat) $ν_{max}$: 2926, 1522, 1264, 1148, 1030, 636 cm^{-1}.

HRMS (ESI$^+$): calculated for [M–TfO$^-$]$^+$ ($C_{28}H_{32}N_3^+$) requires m/z 410.2591, found m/z 410.2590.

3-Phenyl-2-(pyrrolidin-1-yl)oxazolo[3,2-a]pyridin-4-ium trifluoromethanesulfonate (5a)

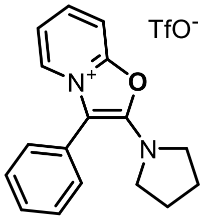

Prepared according to **General Procedure F**.

Yield: 46 mg, 56%, beige solid.

^{1}H NMR (600 MHz, $CDCl_3$): δ 8.03 (d, *J* = 6.4 Hz, 1H), 7.96 (t, *J* = 8.1 Hz, 1H), 7.86 (d, *J* = 8.7 Hz, 1H), 7.65 – 7.62 (m, 2H), 7.59 – 7.53 (m, 4H), 3.42 (app t, *J* = 6.7 Hz, 4H), 1.99 – 1.96 (m, 4H) ppm.

^{13}C NMR (151 MHz, $CDCl_3$): δ 151.4, 146.9, 135.1, 132.6 (2C), 131.1, 129.7 (2C), 125.9, 122.7, 121.6, 110.1, 99.6, 49.1 (2C), 25.5 (2C) ppm. Triflate carbon was not detectable by NMR spectroscopy.

^{19}F NMR (565 MHz, $CDCl_3$): δ –78.3 ppm.

IR (neat) $ν_{max}$: 1665, 1510, 1453, 1265, 1206, 1030, 637 cm^{-1}.

HRMS (ESI$^+$): calculated for $[M–TfO^-]^+$ ($C_{17}H_{17}N_2O^+$) requires m/z 265.1335, found m/z 265.1338.

3-(4-Methoxyphenyl)-2-(pyrrolidin-1-yl)oxazolo[3,2-a]pyridin-4-ium trifluoromethanesulfonate (5b)

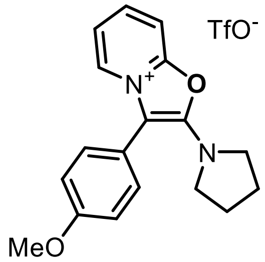

Prepared according to **General Procedure F**.

Yield: 21 mg, 24%, greenish oil.

^{1}H NMR (600 MHz, $CDCl_3$): δ 7.99 (d, *J* = 6.4 Hz, 1H), 7.98 – 7.90 (m, 1H), 7.84 (d, *J* = 8.7 Hz, 1H), 7.59 – 7.51 (m, 3H), 7.08 (d, *J* = 8.7 Hz, 2H), 3.89 (s, 3H), 3.42 (dd, *J* = 7.8, 5.6 Hz, 4H), 2.02 – 1.95 (m, 4H) ppm.

^{13}C NMR (151 MHz, $CDCl_3$): δ 161.8, 151.5, 146.8, 134.7, 134.3 (2C), 126.0, 121.5, 115.2 (2C), 114.2, 110.0, 99.5, 55.7, 49.0 (2C), 25.5 (2C) ppm. Triflate carbon was not detectable by NMR spectroscopy.

^{19}F NMR (665 MHz, $CDCl_3$): δ –78.3 ppm.

IR (neat) $ν_{max}$: 2975, 2878, 2841, 1660, 1628, 1608, 1570, 1524, 1495, 1452, 1376, 1352, 1273, 1251, 1223, 1206, 1179, 1148, 1112, 1030, 964, 914, 897, 861, 842, 763, 758, 735, 671, 637 cm^{-1}.

HRMS (ESI$^+$): calculated for $[M–TfO^-]^+$ ($C_{18}H_{19}N_2O_2^+$) requires *m/z* 295.1441, found *m/z* 295.1445.

3-(4-Nitrophenyl)-2-(pyrrolidin-1-yl)oxazolo[3,2-a]pyridin-4-ium trifluoromethanesulfonate (5c)

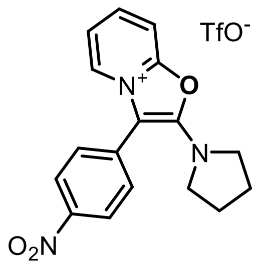

Prepared according to **General Procedure F**.

Yield: 38 mg, 41%, brown oil.

^{1}H NMR (600 MHz, CDCl$_3$): δ 8.42 – 8.35 (m, 2H), 8.20 (d, *J* = 6.5 Hz, 1H), 8.00 – 7.96 (m, 1H), 7.96 – 7.90 (m, 2H), 7.83 (d, *J* = 8.7 Hz, 1H), 7.58 (dd, *J* = 10.3, 3.6 Hz, 1H), 3.44 (t, *J* = 6.7 Hz, 4H), 2.05 – 1.97 (m, 4H) ppm.

^{13}C NMR (151 MHz, CDCl$_3$): δ 151.9, 148.9, 147.3, 135.7, 133.4 (2C), 129.8, 126.2, 124.6 (2C), 121.8, 110.1, 97.9, 49.6 (2C), 25.5 (2C) ppm. Triflate carbon was not detectable by NMR spectroscopy.

^{19}F NMR (565 MHz, CDCl$_3$): δ –78.4 ppm.

IR (neat) $ν_{max}$: 3085, 2925, 2880, 2358, 1765, 1738, 1660, 1630, 1597, 1513, 1493, 1453, 1349, 1323, 1275, 1260, 1223, 1205, 1153, 1106, 1030, 965, 900, 868, 853, 771, 736, 701, 681, 637 cm^{-1}.

HRMS (ESI$^+$): calculated for [M–TfO$^-$]$^+$ ($C_{17}H_{16}N_3O_3^+$) requires *m/z* 310.1186, found *m/z* 310.1190.

3-(4-Iodophenyl)-2-(pyrrolidin-1-yl)oxazolo[3,2-a]pyridin-4-ium trifluoromethanesulfonate (5d)

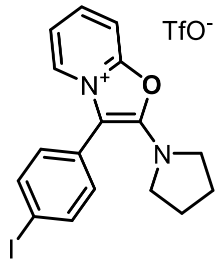

Prepared according to **General Procedure F**.

Yield: 56 mg, 52%, brown solid.

^{1}H NMR (600 MHz, CDCl$_3$): δ 8.02 (d, *J* = 6.4 Hz, 1H), 7.96 – 7.90 (m, 3H), 7.82 (d, *J* = 8.7 Hz, 1H), 7.54 – 7.51 (m, 1H), 7.45 – 7.41 (m, 2H), 3.43 (dd, *J* = 7.8, 5.6 Hz, 4H), 2.03 – 1.97 (m, 4H) ppm.

^{13}C NMR (151 MHz, CDCl$_3$): δ 151.6, 147.0, 139.0 (2C), 135.0, 134.2 (2C), 125.8, 122.4, 121.5, 120.8 (q, *J* = 320.8 Hz, OTf), 110.1, 98.7, 97.8, 49.2 (2C), 25.5 (2C) ppm.

^{19}F NMR (565 MHz, CDCl$_3$): δ –78.3 ppm.

IR (neat) $ν_{max}$: 2878, 1660, 1504, 1258, 1148, 1028, 636 cm^{-1}.

HRMS (ESI$^+$): calculated for [M–TfO$^-$]$^+$ ($C_{17}H_{16}IN_2O^+$) requires m/z 391.0302, found m/z 391.0321.

3-(2-Chlorophenyl)-2-(pyrrolidin-1-yl)oxazolo[3,2-a]pyridin-4-ium trifluoromethanesulfonate (5e)

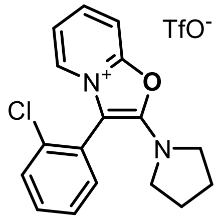

Prepared according to **General Procedure F**.

Yield: 40 mg, 45%, yellow oil.

^{1}H NMR (700 MHz, $CDCl_3$): δ 7.99 (t, *J* = 8.0 Hz, 1H), 7.91 (dd, *J* = 20.7, 7.8 Hz, 2H), 7.79 (d, *J* = 6.3 Hz, 1H), 7.58 (dt, *J* = 8.0, 4.6 Hz, 3H), 7.52 (dd, *J* = 10.7, 4.1 Hz, 1H), 3.47 – 3.40 (m, 4H), 2.02 – 1.97 (m, 4H) ppm.

^{13}C NMR (176 MHz, $CDCl_3$): δ 151.9, 147.1, 137.2, 136.3, 135.2, 133.1, 130.0, 128.3, 126.3, 122.0, 121.6, 110.2, 96.9, 48.8 (2C), 25.5 (2C) ppm. Triflate carbon was not detectable by NMR spectroscopy.

^{19}F NMR (659 MHz, $CDCl_3$): δ –78.3 ppm.

IR (neat) ν_{max}: 3084, 2977, 2925, 2878, 1658, 1629, 1590, 1534, 1506, 1485, 1453, 1424, 1376, 1352, 1325, 1275, 1255, 1222, 1205, 1142, 1072, 1057, 1029, 898, 859, 828, 757, 738, 727, 669, 635 cm^{-1}.

HRMS (ESI$^+$): calculated for $[M–TfO^-]^+$ ($C_{17}H_{16}ClN_2O^+$) requires *m/z* 299.0946, found *m/z* 299.0949.

7-Iodo-3-phenyl-2-(pyrrolidin-1-yl)oxazolo[3,2-a]pyridin-4-ium trifluoromethanesulfonate (5f)

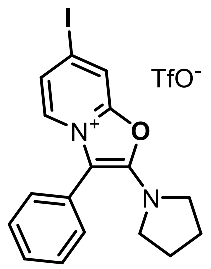

5h, 44%

Prepared according to **General Procedure F**.

Yield: 1.2 g, 44%, light brown solid.

^{1}H NMR (600 MHz, DMSO-d_6): δ 8.74 (d, *J* = 1.0 Hz, 1H), 8.00 (d, *J* = 6.8 Hz, 1H), 7.91 (dd, *J* = 6.8, 1.5 Hz, 1H), 7.58 (s, 5H), 3.26 (t, *J* = 6.5 Hz, 4H), 1.86 (t, *J* = 6.6 Hz, 4H) ppm.

^{13}C NMR (151 MHz, DMSO-d_6): δ 150.1, 146.4, 132.0 (2C), 130.6, 130.2, 129.3 (2C), 126.5, 122.8, 120.7 (q, *J* = 322.3 Hz, OTf), 118.1, 102.3, 99.4, 48.7 (2C), 25.0 (2C) ppm.

^{19}F NMR (565 MHz, DMSO-d_6): δ –77.8 ppm.

IR (neat) ν_{max}: 2362, 1662, 1456, 1261, 1029, 636 cm^{-1}.

HRMS (ESI$^+$): exact mass calculated for $[M–TfO^-]^+$ ($C_{17}H_{16}IN_2O^+$) requires *m/z* 391.0302, found *m/z* 391.0300.

3-Phenyl-2-(pyrrolidin-1-yl)thiazolo[3,2-a]pyridin-4-ium trifluoromethanesulfonate (6a)

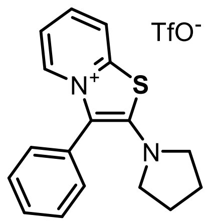

Prepared according to **General Procedure G**.

Yield: 35 mg, 81%, yellow solid.

^{1}H NMR (400 MHz, CDCl$_3$): δ 8.33 (d, *J* = 8.5 Hz, 1H), 8.02 (d, *J* = 6.8 Hz, 1H), 7.79 (dd, *J* = 8.4, 7.6 Hz, 1H), 7.63 – 7.58 (m, 3H), 7.57 – 7.51 (m, 3H), 3.20 – 3.14 (m, 4H), 2.03 – 1.82 (m, 4H) ppm.

^{13}C NMR (151 MHz, CDCl$_3$): δ 147.0, 142.6, 132.9 (2C), 131.5, 131.2, 130.1 (2C), 129.4, 126.7, 123.2, 122.8, 120.9 (q, *J* = 320.9 Hz, OTf), 113.5, 52.9 (2C), 25.9 (2C) ppm.

^{19}F NMR (565 MHz, CDCl$_3$): δ –78.2 ppm.

IR (neat) $ν_{max}$: 3059, 2957, 2877, 1672, 1652, 1613, 1570, 1541, 1495, 1262, 1029, 636 cm^{-1}.

HRMS (ESI$^+$): calculated for [M–TfO$^-$]$^+$ ($C_{17}H_{17}N_2S^+$) requires m/z 281.1107, found m/z 281.1099.

7-Iodo-3-phenyl-2-(pyrrolidin-1-yl)thiazolo[3,2-a]pyridin-4-ium trifluoromethanesulfonate (6b)

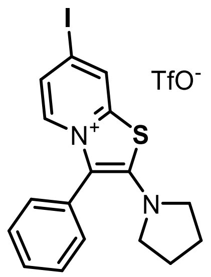

Prepared according to **General Procedure G**.

Yield: 110 mg, 68%, yellow solid.

^{1}H NMR (600 MHz, CDCl$_3$): δ 9.03 (d, *J* = 1.8 Hz, 1H), 7.94 (dd, *J* = 7.0, 1.9 Hz, 1H), 7.85 (d, *J* = 7.1 Hz, 1H), 7.68 – 7.60 (m, 7H), 3.11 (s, 5H), 1.87 (s, 5H) ppm.

^{13}C NMR (151 MHz, CDCl$_3$): δ 145.8, 142.4, 132.8 (2C), 131.2, 131.0, 130.6, 130.2, 129.5, 126.8 (2C), 113.4, 98.3, 52.3 (2C), 25.4 (2C) ppm. Triflate carbon was not detectable by NMR spectroscopy.

^{19}F NMR (565 MHz, CDCl$_3$): δ –77.8 ppm.

IR (neat) $ν_{max}$: 2958, 2851, 1596, 1566, 1500, 1445, 1252, 1118, 1081, 1047, 1029, 912, 888, 872 cm^{-1}.

HRMS (ESI$^+$): calculated for [M–TfO$^-$]$^+$ ($C_{17}H_{16}IN_2S^+$) requires m/z 407.0074, found m/z 407.0081.

7-(4-(Diethylamino)phenyl)-1-phenethyl-3-phenyl-2-(pyrrolidin-1-yl)-1H-imidazo[1,2-a]pyridin-4-ium trifluoromethanesulfonate (7a)

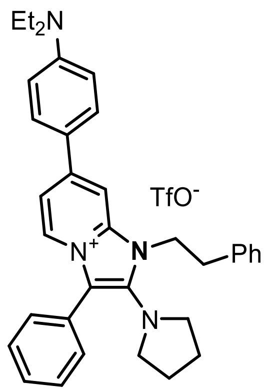

Prepared according to **General Procedure H**.

Yield: 33 mg, 95%.

^{1}H NMR (700 MHz, CDCl$_3$): δ 7.76 (t, *J* = 7.1 Hz, 1H), 7.71 (s, 1H), 7.64 (d, *J* = 8.8 Hz, 2H), 7.60 – 7.59 (m, 3H), 7.43 – 7.41 (m, 2H), 7.39 (dd, *J* = 7.2, 1.5 Hz, 1H), 7.12 (t, *J* = 7.4 Hz, 1H), 7.05 (d, *J* = 7.9 Hz, 2H), 6.76 (d, *J* = 8.9 Hz, 2H), 4.79 (t, *J* = 6.6 Hz, 2H), 3.44 (q, *J* = 7.1 Hz, 4H), 3.23 (t, *J* = 6.6 Hz, 2H), 3.02 – 3.00 (m, 4H), 1.80 – 1.79 (m, 4H), 1.22 (t, *J* = 7.1 Hz, 6H) ppm.

^{13}C NMR (175 MHz, CDCl$_3$): δ 149.6, 145.6, 140.5, 137.6, 137.5, 131.6 (2C), 131.0, 130.0 (2C), 129.0 (2C), 128.8 (4C), 127.3, 125.3, 124.3, 121.3, 115.5, 113.7, 112.0, 104.1, 52.3 (2C), 46.2, 44.6 (2C), 35.1, 25.7 (2C), 12.7 (2C) ppm. Triflate carbon was not detectable by NMR spectroscopy.

^{19}F NMR (659 MHz, CDCl$_3$): δ –78.2 ppm.

IR (neat) ν_{max}: 2972, 1649, 1599, 1536, 1407, 1355, 1262, 1213, 1030, 703, 637 cm^{-1}.

HRMS (ESI$^+$): calculated for [M–TfO$^-$]$^+$ ($C_{35}H_{39}N_4^+$) requires m/z 515.3169, found m/z 515.3167.

1-Phenethyl-3-phenyl-2-(pyrrolidin-1-yl)-7-(2,3,6,7-tetrahydro-1H,5H-pyrido[3,2,1-ij]quinolin-9-yl)-1H-imidazo[1,2-a]pyridin-4-ium trifluoromethanesulfonate (7b)

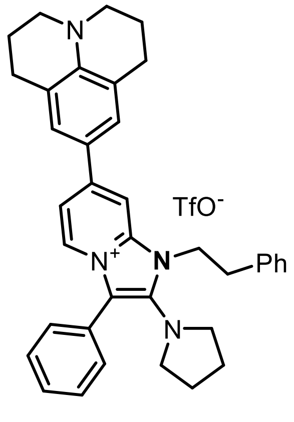

Prepared according to **General Procedure H**.

Yield: 37 mg, 57%, brown solid.

^{1}H NMR (600 MHz, d_6-DMSO): δ 7.99 (s, 1H), 7.69 – 7.58 (m, 5H), 7.57 – 7.54 (m, 2H), 7.34 (s, 2H), 7.25 (app t, *J* = 7.5 Hz, 2H), 7.16 (dd, *J* = 15.4, 7.4 Hz, 3H), 4.70 (t, *J* = 6.7 Hz, 2H), 3.27 – 3.22 (m, 4H), 3.14 (t, *J* = 6.6 Hz, 2H), 2.99 (s, 4H), 2.78 (t, *J* = 6.1 Hz, 4H), 1.94 – 1.88 (m, 4H), 1.74 (s, 4H) ppm.

^{13}C NMR (151 MHz, d_6-DMSO): δ 144.4, 144.1, 139.6, 137.5, 137.0, 131.4 (2C), 130.6, 129.6 (2C), 129.0 (2C), 128.4 (2C), 126.8, 125.6 (2C), 125.1 (2C), 121.1 (2C), 120.4, 114.7, 113.6, 102.7, 51.4 (2C), 49.2 (2C), 44.7, 34.4, 27.3 (2C), 25.2 (2C), 21.2 (2C) ppm. Triflate carbon was not detectable by NMR spectroscopy.

^{19}F NMR (565 MHz, d_6-DMSO): δ –77.8 ppm.

IR (neat) ν_{max}: 1739, 1720, 1592, 1365, 1327, 1226, 1217 cm^{-1}.

HRMS (ESI^{+}): calculated for [M–TfO^{-}]$^{+}$ ($C_{37}H_{39}N_4^{+}$) requires m/z 539.3170, found m/z 539.3161.

7-(4'-(Diethylamino)-[1,1'-biphenyl]-4-yl)-1-phenethyl-3-phenyl-2-(pyrrolidin-1-yl)-1H-imidazo[1,2-a]pyridin-4-ium trifluoromethanesulfonate (7c)

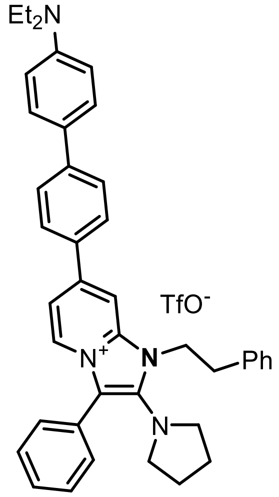

Prepared according to **General Procedure H**.

Yield: 33 mg, 45%, dark orange powder.

^{1}H NMR (600 MHz, d_6-DMSO): δ 8.35 (s, 1H), 8.16 (d, *J* = 7.1 Hz, 1H), 7.95 (d, *J* = 8.5 Hz, 2H), 7.81 (d, *J* = 8.5 Hz, 2H), 7.75 (dd, *J* = 7.2, 1.5 Hz, 1H), 7.69 – 7.65 (m, 3H), 7.63 (d, *J* = 8.9 Hz, 2H), 7.61 – 7.58 (m, 2H), 7.28 – 7.24 (m, 2H), 7.20 (d, *J* = 7.1 Hz, 2H), 7.15 (t, *J* = 7.2 Hz, 1H), 6.78 (d, *J* = 8.9 Hz, 2H), 4.79 (t, *J* = 6.8 Hz, 2H), 3.40 (dd, *J* = 14.1, 7.0 Hz, 5H), 3.17 (t, *J* = 6.9 Hz, 2H), 3.05 (t, *J* = 6.4 Hz, 4H), 1.77 (t, *J* = 6.4 Hz, 4H), 1.13 (t, *J* = 7.0 Hz, 5H) ppm.

^{13}C NMR (151 MHz, d_6-DMSO): δ 147.4, 143.0, 142.0, 140.3, 137.4, 136.5, 132.5, 131.5 (2C), 130.7, 129.6 (2C), 129.0 (2C), 128.4 (2C), 127.8 (2C), 127.6 (2C), 126.8, 125.8 (2C), 125.5, 125.0, 124.9, 115.6, 113.6, 111.8 (2C), 106.0, 51.4 (2C), 45.1, 43.7(2C), 34.3, 25.3 (2C), 12.5 (2C) ppm. Triflate carbon was not detectable by NMR spectroscopy.

^{19}F NMR (565 MHz, d_6-DMSO): δ –77.8 ppm.

IR (neat) ν_{max}: 2970, 1738, 1716, 1648, 1596, 1535, 1515, 1498, 1464, 1421, 1401, 1356, 1268, 1219 cm^{-1}.

HRMS (ESI$^+$): calculated for $[M–TfO^-]^+$ ($C_{41}H_{43}N_4^+$) requires m/z 591.3483, found m/z 591.3477.

7-(4-(Methoxycarbonyl)phenyl)-1-phenethyl-3-phenyl-2-(pyrrolidin-1-yl)-1H-imidazo[1,2-a]pyridin-4-ium trifluoromethanesulfonate and trifluoroacetate (7d)

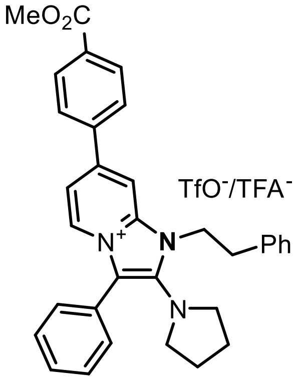

Prepared according to **General Procedure H**. The product was isolated by reverse-phase column chromatography (H_2O/MeCN + 0.1% TFA, 95/5 to 0/100) resulting in partial anion exchange to trifluoroacetate.

Yield: 49 mg, 72%, brown oil.

^{1}H NMR (600 MHz, $CDCl_3$): δ 8.21 – 8.12 (m, 3H), 7.92 – 7.84 (m, 3H), 7.65 – 7.59 (m, 3H), 7.54 – 7.48 (m, 2H), 7.44 (d, *J* = 5.5 Hz, 1H), 7.17 (t, *J* = 7.4 Hz, 2H), 7.13 – 7.06 (m, 3H), 5.08 (brs, 2H), 3.95 (s, 3H), 3.26 (brs, 2H), 3.18 – 3.08 (m, 4H), 1.88 – 1.79 (m, 4H) ppm.

^{13}C NMR (151 MHz, $CDCl_3$): δ 166.6, 143.4, 141.6, 140.0, 137.4, 137.0, 131.9 (2C), 131.5, 131.3, 130.7 (2C), 130.1 (2C), 129.1 (2C), 128.8 (2C), 127.9 (2C), 127.4, 125.0, 124.6, 116.7, 113.7, 109.4, 52.5 (3C), 47.8, 35.4, 25.9 (2C) ppm. Triflate carbon was not detectable by NMR spectroscopy.

^{19}F NMR (565 MHz, $CDCl_3$): δ –75.0 (TFA^-), –78.3 (TfO^-) ppm.

IR (neat) ν_{max}: 1715, 1687, 1647, 1588, 1535, 1434, 1279, 1192, 1109, 770, 731, 700 cm^{-1}.

HRMS (ESI$^+$): calculated for $[M–TfO^-]^+$ ($C_{33}H_{32}N_3O_2^+$) requires m/z 502.2490, found m/z 502.2485.

1-(3-Azidopropyl)-7-(4-(diethylamino)phenyl)-3-phenyl-2-(pyrrolidin-1-yl)-1H-imidazo[1,2-a]pyridin-4-ium trifluoromethanesulfonate (7e)

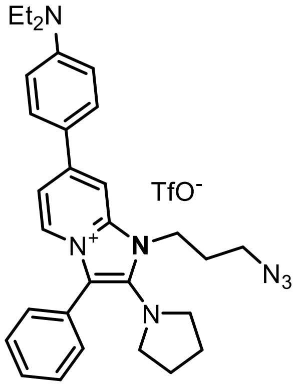

Prepared according to **General Procedure H**.

Yield: 54 mg, 74%.

^{1}H NMR (600 MHz, CDCl$_3$): δ 8.17 (s, 1H), 7.84 (d, *J* = 7.2 Hz, 1H), 7.81 (d, *J* = 9.0 Hz, 2H), 7.65 – 7.59 (m, 3H), 7.55 – 7.50 (m, 2H), 7.46 (dd, *J* = 7.2, 1.6 Hz, 1H), 6.78 (d, *J* = 9.0 Hz, 2H), 4.67 (t, *J* = 7.3 Hz, 2H), 3.68 – 3.64 (m, 2H), 3.43 (q, *J* = 7.1 Hz, 4H), 3.25 – 3.18 (m, 4H), 2.28 – 2.21 (m, 2H), 1.91 – 1.87 (m, 4H), 1.21 (t, *J* = 7.1 Hz, 6H) ppm.

^{13}C NMR (151 MHz, CDCl$_3$): δ 149.7, 146.3, 140.3, 137.8, 131.6 (2C), 131.1, 130.0 (2C), 129.0 (2C), 125.3, 124.4, 121.2, 115.6, 114.2, 112.1 (2C), 103.8, 52.7 (2C), 49.0, 44.7 (2C), 42.1, 28.5, 25.9 (2C), 12.7 (2C) ppm. Triflate carbon was not detectable by NMR spectroscopy.

^{19}F NMR (565 MHz, CDCl$_3$): δ –78.2 ppm.

HRMS (ESI$^+$): calculated for [M–TfO$^-$]$^+$ ($C_{30}H_{36}N_7^+$) requires m/z 494.3027, found m/z 494.3022.

7-(4-(Diethylamino)phenyl)-1-(4-methoxy-4-oxobutyl)-3-phenyl-2-(pyrrolidin-1-yl)-1H-imidazo[1,2-a]pyridin-4-ium trifluoromethanesulfonate (7f)

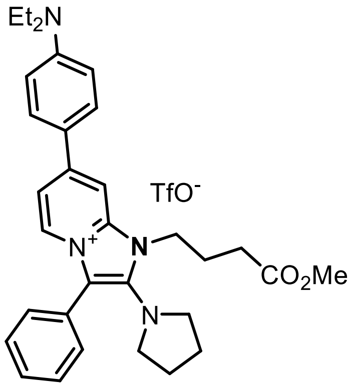

Prepared according to **General Procedure H**.

Yield: 180 mg, 88%, brown oil.

^{1}H NMR (600 MHz, CDCl$_3$): δ 8.16 (s, 1H), 7.88 (d, *J* = 7.2 Hz, 1H), 7.80 (d, *J* = 8.9 Hz, 2H), 7.65 – 7.60 (m, 3H), 7.52 (d, *J* = 7.6 Hz, 2H), 7.49 (d, *J* = 7.0 Hz, 1H), 6.78 (d, *J* = 8.9 Hz, 2H), 4.66 – 4.56 (m, 2H), 3.69 (s, 3H), 3.44 (q, *J* = 7.0 Hz, 4H), 3.21 (t, *J* = 6.4 Hz, 4H), 2.65 (t, *J* = 6.6 Hz, 2H), 2.29 – 2.21 (m, 2H), 1.88 (t, *J* = 6.7 Hz, 4H), 1.22 (t, *J* = 7.1 Hz, 6H) ppm.

^{13}C NMR (151 MHz, CDCl$_3$): δ 173.5, 149.6, 146.0, 140.3, 137.6, 131.6 (2C), 131.0, 130.0 (2C), 128.9 (2C), 125.2, 124.5, 121.3, 115.7, 114.1, 112.0 (2C), 103.8, 52.6 (2C), 51.9, 44.6 (2C), 43.6, 30.6, 25.8 (2C), 24.2, 12.7 ppm. Triflate carbon was not detectable by NMR spectroscopy.

^{19}F NMR (565 MHz, CDCl$_3$): δ –78.2 ppm.

IR (neat) ν_{max}: 2972, 2933, 2872, 1733, 1645, 1536, 1467, 1446, 1438, 1408, 1376, 1356, 1315, 1263, 1212, 1156, 1115, 1078, 1031, 808, 637 cm^{-1}.

HRMS (ESI$^+$): exact mass calculated for [M–TfO$^-$]$^+$ ($C_{32}H_{39}N_4O_2^+$) requires *m/z* 511.3068, found *m/z* 511.3087.

(*E*)-7-(4-(Diethylamino)styryl)-1-phenethyl-3-phenyl-2-(pyrrolidin-1-yl)-1H-imidazo[1,2-a]pyridin-4-ium trifluoromethanesulfonate (7g)

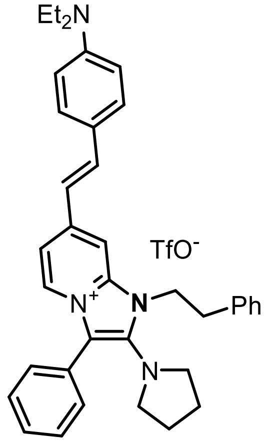

Prepared according to **General Procedure H**.

Yield: 20 mg, 58%, red powder. As a mixture of double bond isomers E/Z 1.00:0.15. For simplicity, only the major isomer is described.

^{1}H NMR (600 MHz, CDCl$_3$): δ 7.69 (t, *J* = 7.1 Hz, 1H), 7.65 (s, 1H), 7.59 – 7.55 (m, 3H), 7.49 (d, *J* = 8.8 Hz, 2H), 7.40 – 7.38 (m, 2H), 7.31 – 7.28 (m, 2H), 7.20 (t, *J* = 7.0 Hz, 2H), 7.14 – 7.13 (m, 1H), 7.05 (d, *J* = 7.9 Hz, 2H), 6.92 (d, *J* = 16.1 Hz, 1H), 6.66 (d, *J* = 8.9 Hz, 2H), 4.72 (t, *J* = 6.6 Hz, 2H), 3.40 (q, *J* = 7.1 Hz, 4H), 3.22 (t, *J* = 6.6 Hz, 2H), 2.98 – 2.97 (m, 4H), 1.79 – 1.77 (m, 4H), 1.19 (t, *J* = 7.1 Hz, 6H) ppm.

^{13}C NMR (151 MHz, CDCl$_3$): δ 149.0, 143.8, 140.4, 137.5, 137.4, 136.9, 131.5 (2C), 131.0, 130.0 (2C), 129.7 (2C), 129.1 (2C), 128.8 (2C), 127.3, 125.2, 124.0, 122.8, 118.5, 115.0, 114.2, 111.6 (2C), 105.8, 52.3 (2C), 46.2, 44.6 (2C), 35.2, 25.7 (2C), 12.8 (2C) ppm. Triflate carbon was not detectable by NMR spectroscopy.

^{19}F NMR (565 MHz, $CDCl_3$): δ –78.1 ppm.

IR (neat) ν_{max}: 2973, 2363, 1650, 1592, 1524, 1374, 1266, 1189, 1153, 1013, 621 cm^{-1}.

HRMS (ESI$^+$): calculated for $[M–TfO^-]^+$ ($C_{37}H_{41}N_4^+$) requires m/z 541.3326, found m/z 541.3332.

(*E*)-1-Phenethyl-3-phenyl-2-(pyrrolidin-1-yl)-7-(2-(2,3,6,7-tetrahydro-1H,5H-pyrido[3,2,1-ij]quinolin-9-yl)vinyl)-1H-imidazo[1,2-a]pyridin-4-ium trifluoromethanesulfonate (7h)

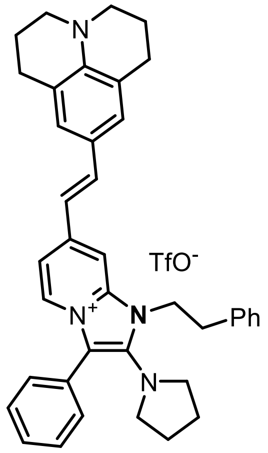

Prepared according to **General Procedure H**.

Yield: 16 mg, 32%, orange solid.

^{1}H NMR (600 MHz, d_6-DMSO): δ 8.04 (d, *J* = 7.1 Hz, 1H), 7.95 (s, 1H), 7.64 (d, *J* = 6.6 Hz, 3H), 7.57 – 7.52 (m, 3H), 7.45 (d, *J* = 16.1 Hz, 1H), 7.30 – 7.25 (m, 2H), 7.23 – 7.18 (m, 3H), 7.08 (s, 2H), 7.02 (d, *J* = 16.2 Hz, 1H), 4.59 (t, *J* = 7.0 Hz, 2H), 3.23 – 3.19 (m, 4H), 3.15 (t, *J* = 7.1 Hz, 2H), 2.99 (m, 4H), 2.71 (t, *J* = 6.2 Hz, 4H), 1.91 – 1.86 (m, 4H), 1.75 (t, *J* = 6.2 Hz, 4H) ppm.

^{13}C NMR (151 MHz, d_6-DMSO): δ 143.9, 142.8, 139.7, 137.3, 136.7, 136.2, 131.4 (2C), 130.5, 129.6 (2C), 129.0 (2C), 128.5 (2C), 126.8, 126.5 (2C), 125.1 (2C), 122.3, 120.8 (2C), 118.2, 114.8, 114.0, 104.6, 51.4 (2C), 49.2 (2C), 44.9, 34.2, 27.2 (2C), 25.2 (2C), 21.2 (2C) ppm. Triflate carbon was not detectable by NMR spectroscopy.

^{19}F NMR (565 MHz, d_6-DMSO): δ –77.8 ppm.

IR (neat) ν_{max}: 1648, 1592, 1506, 1311, 701 cm^{-1}.

HRMS (ESI$^+$): calculated for $[M–TfO^-]^+$ ($C_{39}H_{41}N_4^+$) requires m/z 565.3326, found m/z 565.3328.

(*E*)-7-(4-Morpholinostyryl)-1-phenethyl-3-phenyl-2-(pyrrolidin-1-yl)-1H-imidazo[1,2-a]pyridin-4-ium trifluoromethanesulfonate (7i)

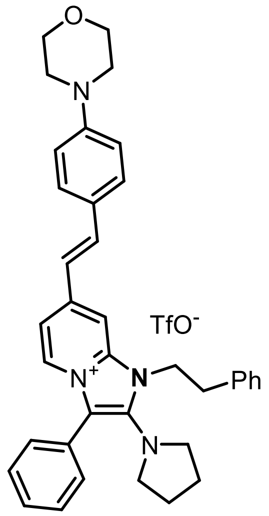

Prepared according to **General Procedure H**.

Yield: 13 mg, 37%, dark orange solid. As a mixture of double bond isomers E/Z 2:1. For simplicity, only the major isomer is described.

^{1}H NMR (600 MHz, $CDCl_3$): δ 7.86 (s, 1H), 7.72 (d, *J* = 7.1 Hz, 2H), 7.59 – 7.55 (m, 3H) 7.50 (s, 1H), 7.39 – 7.38 (m, 2H), 7.35 – 7.34 (m, 1H), 7.20 – 7.18 (m, 2H), 7.13 (t, *J* = 7.4 Hz, 1H), 7.07 (d, *J* = 6.1 Hz, 2H), 7.05 – 7.01 (m, 1H), 6.88 (d, *J* = 8.7 Hz, 2H), 6.82 – 6.81 (m, 1H), 4.76 (t, *J* = 6.6 Hz, 2H), 3.86 – 3.84 (m, 4H), 3.76 – 3.74 (m, 2H), 3.23 – 3.22 (m, 4H), 3.10 – 3.08 (m, 2H), 2.98 – 2.97 (m, 4H), 1.79 – 1.76 (m, 4H) ppm.

^{13}C NMR (151 MHz, $CDCl_3$): δ 152.0, 143.2, 140.6, 137.3, 136.6, 136.2, 131.8, 131.5 (2C), 131.1, 130.5, 130.0 (2C), 129.9, 129.2 (2C), 129.1 (2C), 129.0, 128.9, 128.8 (2C), 127.3, 127.0, 125.1, 124.0, 121.0, 118.3, 115.2, 115.1 (2C), 115.0, 114.2, 109.5, 106.8, 66.8 (2C), 52.2 (2C), 48.4 (2C), 46.3, 35.2, 25.7 (2C) ppm. Triflate carbon was not detectable by NMR spectroscopy.

^{19}F NMR (565 MHz, $CDCl_3$): δ –78.1 ppm.

IR (neat) ν_{max}: 2967, 2343, 1650, 1597, 1536, 1263, 1235, 1151, 1121, 1030, 928, 702, 637 cm^{-1}.

HRMS (ESI$^+$): calculated for $[M–TfO^-]^+$ ($C_{37}H_{39}N_4O^+$) requires m/z 555.3118, found m/z 555.3128.

1-Phenethyl-3-phenyl-2-(pyrrolidin-1-yl)-7-((*E*)-4-((*E*)-3,4,5-trimethoxystyryl)styryl)-1H-imidazo[1,2-a]pyridin-4-ium trifluoromethanesulfonate (7j)

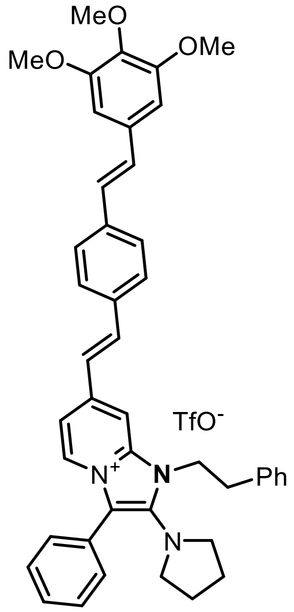

Prepared according to **General Procedure H**.

Yield: 10 mg, 19%, yellow powder.

^{1}H NMR (600 MHz, $CDCl_3$): δ 8.06 (s, 1H), 7.73 (d, *J* = 7.1 Hz, 1H), 7.65 (d, *J* = 8.3 Hz, 2H), 7.62 – 7.58 (m, 3H), 7.52 (d, *J* = 8.4 Hz, 2H), 7.48 (d, *J* = 16.3 Hz, 1H), 7.44 – 7.39 (m, 2H), 7.38 (dd, *J* = 7.2, 1.3 Hz, 1H), 7.24 – 7.19 (m, 3H), 7.14 (t, *J* = 7.4 Hz, 1H), 7.12 – 7.06 (m, 3H), 7.00 (d, *J* = 16.2 Hz, 1H), 6.76 (s, 2H), 4.85 (t, *J* = 6.6 Hz, 2H), 3.94 (s, 6H), 3.88 (s, 3H), 3.25 (t, *J* = 6.7 Hz, 2H), 3.02 (t, *J* = 6.5 Hz, 4H), 1.82 – 1.77 (m, 4H) ppm.

^{13}C NMR (151 MHz, $CDCl_3$): δ 153.6 (2C), 142.4, 140.9, 138.4 (2C), 137.3, 137.2, 135.8, 135.1, 133.0, 131.6 (2C), 131.1, 130.0 (2C), 129.7, 129.1 (2C), 128.8 (2C), 128.3 (2C), 127.6, 127.3, 127.0 (2C), 125.1, 124.0 (2C), 115.4, 114.2, 108.0, 103.9 (2C), 61.2, 56.3 (2C), 52.3 (2C), 46.6, 35.2, 25.8 (2C) ppm. Triflate carbon was not detectable by NMR spectroscopy.

^{19}F NMR (565 MHz, $CDCl_3$): δ –78.1 ppm.

IR (neat) ν_{max}: 3361, 3322, 3213, 3196, 2957, 2922, 2853, 1713, 1701, 1659, 1506, 1465, 1424, 1277, 1260, 1226, 1031, 967, 888, 722, 638 cm^{-1}.

HRMS (ESI$^+$): exact mass calculated for $[M–TfO^-]^+$ ($C_{44}H_{44}N_3O_3^+$) requires *m/z* 662.3377, found *m/z* 662.3377.

7-((1*E*,3*E*)-4-(4-(Diethylamino)phenyl)buta-1,3-dien-1-yl)-1-phenethyl-3-phenyl-2-(pyrrolidin-1-yl)-1H-imidazo[1,2-a]pyridin-4-ium trifluoromethanesulfonate (7k)

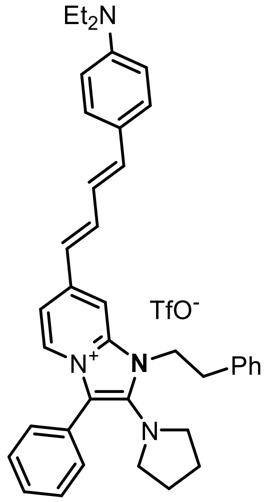

Prepared according to **General Procedure H**.

Yield: 17 mg, 47%, dark red solid. As a mixture of three double bond isomers 4:1:0.7. For simplicity, only the major (*E,E*) isomer is described.

^{1}H NMR (600 MHz, $CDCl_3$): δ 7.68 – 7.67 (m, 2H), 7.58 – 7.55 (m, 3H) 7.50 – 7.49 (m, 1H), 7.39 – 7.37 (m, 2H), 7.35 (d, *J* = 8.9 Hz, 2H), 7.24 – 7.23 (m, 2H), 7.21 – 7.19 (m, 2H), 7.15 – 7.13 (m, 1H), 7.06 (d, *J* = 7.0 Hz, 2H), 6.83 (d, *J* = 15.3 Hz, 1H), 6.63 (d, *J* = 8.9 Hz, 2H), 6.56 (d, *J* = 15.3 Hz, 1H), 4.76 (t, *J* = 6.5 Hz, 2H), 3.39 (q, *J* = 7.1 Hz, 4H), 3.22 (t, *J* = 6.5 Hz, 2H), 2.98 – 2.97 (m, 4H), 1.79 – 1.77 (m, 4H), 1.18 (t, *J* = 7.1 Hz, 6H) ppm.

^{13}C NMR (151 MHz, $CDCl_3$): δ 148.4, 143.2, 140.6, 139.4, 138.2, 137.4 (2C), 131.5 (2C), 131.0, 130.0 (2C), 129.1 (2C), 129.0 (2C), 128.8 (2C), 127.3, 125.2, 124.6, 124.0, 123.9, 123.0, 115.1, 111.7, 111.6 (2C), 106.2, 52.3 (2C), 46.4, 44.6 (2C), 35.2, 25.7 (2C), 12.8 (2C) ppm. Triflate carbon was not detectable by NMR spectroscopy.

^{19}F NMR (565 MHz, $CDCl_3$): δ –78.1 ppm.

IR (neat) ν_{max}: 2632, 1650, 1585, 1521, 1403, 1355, 1269, 1190, 1148, 1031, 637 cm^{-1}.

HRMS (ESI$^+$): calculated for $[M–TfO^-]^+$ ($C_{39}H_{43}N_4^+$) requires m/z 567.3482, found m/z 567.3489.

1-Phenethyl-3-phenyl-2-(pyrrolidin-1-yl)-7-((1*E*,3*E*)-4-(2,3,6,7-tetrahydro-1H,5H-pyrido[3,2,1-ij]quinolin-9-yl)buta-1,3-dien-1-yl)-1H-imidazo[1,2-a]pyridin-4-ium trifluoromethanesulfonate (7l)

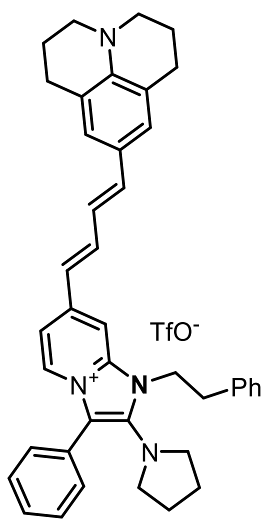

Prepared according to **General Procedure H**.

Yield: 12 mg, 54%, red solid. As a mixture of three double bond isomers 1:0.15:0.12. For simplicity, only major isomer (*E,E*) is described.

^{1}H NMR (600 MHz, d$_6$-DMSO): δ 8.02 (d, *J* = 7.2 Hz, 1H), 7.93 (s, 1H), 7.67 – 7.63 (m, 3H), 7.57 – 7.53 (m, 2H), 7.48 (d, *J* = 7.1 Hz, 1H), 7.43 (dd, *J* = 15.4, 10.7 Hz, 1H), 7.28 – 7.25 (m, 2H), 7.21 – 7.17 (m, 3H), 6.96 (s, 2H), 6.85 (dd, *J* = 15.2, 10.6 Hz, 1H), 6.75 (d, *J* = 15.3 Hz, 1H), 6.62 (d, *J* = 15.4 Hz, 1H), 4.62 (t, *J* = 6.9 Hz, 2H), 3.20 – 3.15 (m, 4H), 3.14 (t, *J* = 7.0 Hz, 2H), 2.99 (t, *J* = 6.4 Hz, 4H), 2.68 (t, *J* = 6.3 Hz, 4H), 1.89 – 1.85 (m, 4H), 1.76 – 1.74 (m, 4H) ppm.

^{13}C NMR (151 MHz, d$_6$-DMSO): δ 143.4, 142.0, 139.9, 138.8, 137.6, 137.4, 136.6, 131.4 (2C), 130.6, 129.6 (2C), 129.0 (2C), 128.4 (2C), 126.8 (2C), 126.0 (2C), 124.6, 123.1, 122.6, 120.8 (2C), 117.8, 114.9, 114.0, 105.1, 51.4 (2C), 49.3 (2C), 44.9, 34.3, 27.2 (2C), 25.2 (2C), 21.3 (2C) ppm. Triflate carbon was not detectable by NMR spectroscopy.

^{19}F NMR (565 MHz, d$_6$-DMSO): δ –77.8 ppm.

IR (neat) ν_{max}: 1646, 1507, 1462, 1309, 1181, 700 cm^{-1}.

HRMS (ESI$^+$): calculated for $[M–TfO^-]^+$ ($C_{41}H_{43}N^+$) requires m/z 591.3483, found m/z 591.3482.

1-Phenethyl-3-phenyl-2-(pyrrolidin-1-yl)-7-((1*E*,3*E*)-4-(p-tolyl)buta-1,3-dien-1-yl)-1H-imidazo[1,2-a]pyridin-4-ium trifluoromethanesulfonate (7m)

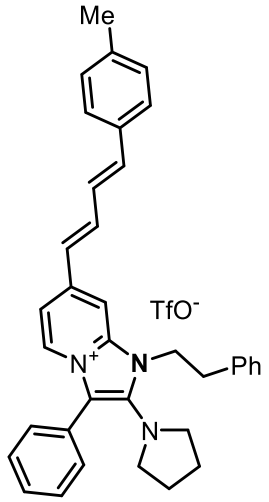

Prepared according to **General Procedure H**.

Yield: 13 mg, 39%, orange-yellow solid. As a mixture of double bond isomers 5:1. For simplicity, only major isomer (*E,E*) is described.

^{1}H NMR (600 MHz, $CDCl_3$): δ 7.85 (s, 1H), 7.70 (d, *J* = 7.1 Hz, 1H), 7.59 – 7.57 (m, 4H), 7.39 – 7.34 (m, *J* = 15.6, 8.4, 5.4 Hz, 4H), 7.29 – 7.27 (m, *J* = 7.1, 1.5 Hz, 1H), 7.21 – 7.18 (m, *J* = 7.4 Hz, 2H), 7.16 – 7.12 (m, 3H), 7.06 (d, *J* = 7.1 Hz, 2H), 6.92 – 6.90 (m, 2H), 6.68 (d, *J* = 15.5 Hz, 1H), 4.79 (t, *J* = 6.6 Hz, 2H), 3.21 (t, *J* = 6.6 Hz, 2H), 3.00 – 2.96 (m, *J* = 6.5 Hz, 4H), 2.35 (s, 3H), 1.79 – 1.76 (m, 4H) ppm.

^{13}C NMR (151 MHz, $CDCl_3$): δ 142.4, 140.8, 138.9, 138.3, 137.3, 137.2 (2C), 134.0, 131.5 (2C), 131.1, 130.0 (2C), 129.6 (2C), 129.1 (2C), 128.8 (2C), 127.3 (2C), 127.1 (3C), 125.0, 124.0, 115.3, 114.2, 107.2, 52.2 (2C), 46.6, 35.2, 25.7 (2C), 25.0 ppm. Triflate carbon was not detectable by NMR spectroscopy.

^{19}F NMR (565 MHz, $CDCl_3$): δ –78.1 ppm.

IR (neat) ν_{max}: 2988, 2329, 1649, 1618, 1596, 1536, 1469, 1263, 1223, 1151, 1030, 753, 702, 637 cm^{-1}.

HRMS (ESI$^+$): calculated for $[M–TfO^-]^+$ ($C_{36}H_{36}N_3^+$) requires m/z 510.2904, found m/z 510.2909.

7-((1*E*,3*E*)-4-(4-Morpholinophenyl)buta-1,3-dien-1-yl)-1-phenethyl-3-phenyl-2-(pyrrolidin-1-yl)-1H-imidazo[1,2-a]pyridin-4-ium trifluoromethanesulfonate (7n)

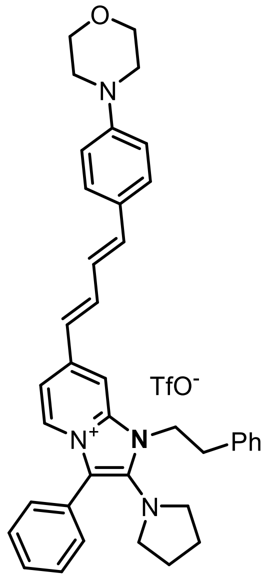

Prepared according to **General Procedure H**.

Yield: 16 mg, 44%, red powder. As a mixture of three double bond isomers 1.0:0.5:0.25. For simplicity, only major isomer (*E,E*) is described.

^{1}H NMR (600 MHz, CDCl$_3$): δ 7.78 (s, 1H), 7.69 (d, *J* = 7.1 Hz, 1H), 7.58 – 7.55 (m, 3H) 7.50 – 7.49 (m, 1H), 7.41 – 7.37 (m, 3H), 7.32 – 7.26 (m, 2H), 7.21 – 7.19 (m, 1H), 7.16 – 7.11 (m, 1H), 7.06 – 7.03 (m, 3H), 6.87 –6.83 (m, 4H), 6.63 (d, *J* = 15.4 Hz, 1H), 4.77 (t, *J* = 6.6 Hz, 2H), 3.86 – 3.85 (m, 4H), 3.84 – 3.82 (m, 2H), 3.22 – 3.20 (m, 4H), 3.19 – 3.17 (m, 2H), 2.98 – 2.96 (m, 4H), 1.79 –1.77 (m, 4H) ppm.

^{13}C NMR (151 MHz, CDCl$_3$): δ 151.5, 142.8, 140.7, 138.3, 137.6, 137.3 (2C), 131.8, 131.5 (2C), 131.1, 130.0 (2C), 129.1 (2C), 128.8 (2C), 128.5 (2C), 128.2, 127.3, 126.1, 125.3, 125.1, 124.0, 115.2 (2C), 114.2, 106.8, 66.9 (2C), 52.2 (2C), 48.7 (2C), 46.4, 35.2, 25.8 (2C) ppm. Triflate carbon was not detectable by NMR spectroscopy.

^{19}F NMR (565 MHz, CDCl$_3$): δ –78.1 ppm.

IR (neat) ν_{max}: 2362, 1649, 1592, 1537, 1469, 1263, 1150, 1121, 1052, 927, 637 cm^{-1}.

HRMS (ESI$^+$): calculated for [M–TfO$^-$]$^+$ ($C_{39}H_{41}N_4O^+$) requires m/z 581.3275, found m/z 581.3284.

1-Phenethyl-3-phenyl-2-(pyrrolidin-1-yl)-7-((1*E*,3*E*,5*E*)-6-(4-(trifluoromethyl)phenyl)hexa-1,3,5-trien-1-yl)-1H-imidazo[1,2-a]pyridin-4-ium trifluoromethanesulfonate (7o)

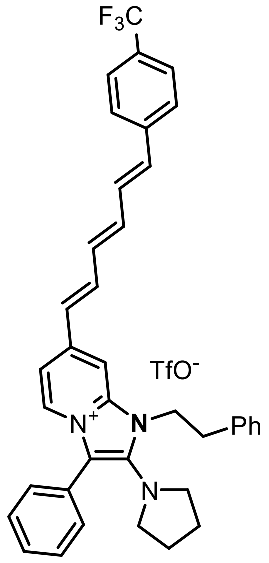

Prepared according to **General Procedure H**.

Yield: 46 mg, quantit, orange-brown solid. As a mixture of three double bond isomers 1.0:0.1:0.1. For simplicity, only major isomer (*E,E,E*) is described.

^{1}H NMR (700 MHz, CDCl$_3$): δ 7.89 (s, 1H), 7.69 (d, *J* = 7.1 Hz, 1H), 7.59 – 7.55 (m, 6H), 7.51 (d, *J* = 8.2 Hz, 2H), 7.39 – 7.37 (m, 2H), 7.31 (dd, *J* = 15.4, 10.9 Hz, 1H), 7.23 (dd, *J* = 7.2, 1.4 Hz, 1H), 7.20 –7.18 (m, 2H), 7.14 –7.12 (m, 1H), 7.06 (d, *J* = 7.0 Hz, 1H), 6.96 (dd, *J* = 15.4, 10.9 Hz, 1H), 6.81 (dd, *J* = 14.6, 10.9 Hz, 1H), 6.72 (d, *J* = 15.4 Hz, 1H), 6.67 (d, *J* = 15.4 Hz, 1H), 6.59 (dd, *J* = 15.4, 10.9 Hz, 1H), 4.80 (t, *J* = 6.6 Hz, 2H), 3.86 – 3.85 (m, 4H), 3.84 – 3.82 (m, 2H), 3.22 (t, *J* = 6.6 Hz, 2H), 3.00 – 2.98 (m, 4H), 1.79 – 1.77 (m, 4H) ppm.

^{13}C NMR (175 MHz, CDCl$_3$): δ 142.1, 141.0, 140.6, 138.1, 137.3, 137.2, 136.6, 133.8 (2C), 131.5 (2C), 131.2, 131.1, 130.0 (2C), 129.6 (q, *J* = 32.3 Hz), 129.1 (2C), 128.8 (2C), 128.4, 127.3, 126.9 (2C), 125.8 (q, *J* = 3.7 Hz, 2C), 125.0, 124.0, 122.7 (q, *J* = 272.5 Hz), 115.5, 114.1, 107.4, 52.2 (2C), 46.5, 35.2, 25.8 (2C) ppm. Triflate carbon was not detectable by NMR spectroscopy.

^{19}F NMR (659 MHz, CDCl$_3$): δ –62.5, –78.1 ppm.

IR (neat) ν_{max}: 2362, 1647, 1613, 1594, 1471, 1324, 1262, 1159, 1120, 1066, 1031, 702, 622 cm^{-1}.

HRMS (ESI$^+$): calculated for [M–TfO$^-$]$^+$ ($C_{38}H_{35}F_3N_3^+$) requires m/z 590.2778, found m/z 590.2777.

7-(4-(Diethylamino)phenyl)-3-phenyl-2-(pyrrolidin-1-yl)oxazolo[3,2-a]pyridin-4-ium trifluoromethanesulfonate (8a)

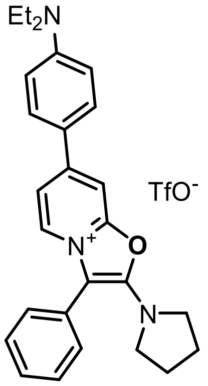

Prepared according to General Procedure H.

Yield: 41 mg, 73%, yellow solid.

^{1}H NMR (600 MHz, d_6-DMSO): δ 8.43 (s, 1H), 8.17 (d, *J* = 7.0 Hz, 1H), 7.91 (d, *J* = 5.7 Hz, 1H), 7.85 (d, *J* = 9.0 Hz, 2H), 7.67 – 7.59 (m, 5H), 6.80 (d, *J* = 9.0 Hz, 2H), 3.44 (q, *J* = 7.0 Hz, 4H), 3.34 – 3.30 (m, 4H), 1.93 – 1.90 (m, 4H), 1.13 (t, *J* = 7.0 Hz, 6H) ppm.

^{13}C NMR (151 MHz, d_6-DMSO): δ 150.0, 149.4, 147.9, 146.9, 132.1 (2C), 130.3, 129.2 (2C), 128.7 (2C), 126.3, 123.3, 119.8, 117.0, 111.7 (2C), 102.3, 98.7, 48.7 (2C), 43.9 (2C), 25.0 (2C), 12.5 (2C) ppm. Triflate carbon was not detectable by NMR spectroscopy.

^{19}F NMR (565 MHz, d_6-DMSO): δ –77.8 ppm.

IR (neat) ν_{max}: 1659, 1596, 1465, 1265, 1155, 1030, 637 cm^{-1}.

HRMS (ESI$^+$): calculated for $[M–TfO^-]^+$ ($C_{27}H_{30}N_3O^+$) requires m/z 412.2384, found m/z 412.2380.

3-Phenyl-2-(pyrrolidin-1-yl)-7-(2,3,6,7-tetrahydro-1H,5H-pyrido[3,2,1-ij]quinolin-9-yl)oxazolo[3,2-a]pyridin-4-ium trifluoromethanesulfonate (8b)

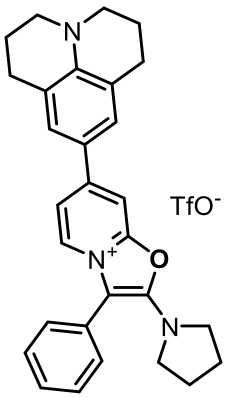

Prepared according to General Procedure H.

Yield: 49 mg, 84%, red-brown solid.

^{1}H NMR (600 MHz, d$_6$-DMSO): δ 8.36 (s, 1H), 8.13 (d, *J* = 7.0 Hz, 1H), 7.86 (dd, *J* = 7.0, 1.2 Hz, 1H), 7.67 – 7.58 (m, 6H), 7.46 (s, 2H), 3.31 (t, *J* = 6.2 Hz, 4H), 3.26 – 3.23 (m, 4H), 2.75 (t, *J* = 6.1 Hz, 4H), 1.93 – 1.86 (m, 9H) ppm.

^{13}C NMR (151 MHz, d$_6$-DMSO): δ 149.8, 147.9, 147.2, 145.0, 132.0 (2C), 130.2, 129.2 (2C), 126.1, 125.7 (2C), 123.3, 121.2 (2C), 119.2, 116.7, 101.8, 98.6, 49.2 (2C), 48.6 (2C), 27.2 (2C), 24.9 (2C), 21.0 (2C) ppm. Triflate carbon was not detectable by NMR spectroscopy.

^{19}F NMR (565 MHz, d$_6$-DMSO): δ –77.8 ppm.

IR (neat) ν_{max}: 1737, 1715, 1662, 1364, 1275, 1260, 1224, 1155, 1030 cm^{-1}.

HRMS (ESI$^+$): calculated for [M–TfO$^-$]$^+$ ($C_{29}H_{30}N_3O^+$) requires m/z 436.2384, found m/z 436.2379.

7-(4'-(Diethylamino)-[1,1'-biphenyl]-4-yl)-3-phenyl-2-(pyrrolidin-1-yl)oxazolo[3,2-a]pyridin-4-ium trifluoromethanesulfonate (8c)

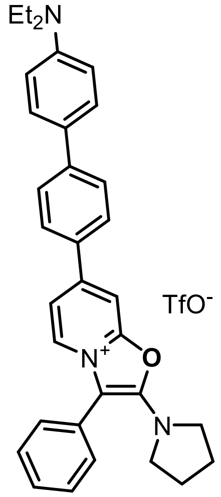

Prepared according to **General Procedure H**.

Yield: 36 mg, 57%, red powder.

^{1}H NMR (600 MHz, d$_6$-DMSO): δ 8.65 – 8.59 (m, 1H), 8.31 (d, *J* = 6.6 Hz, 1H), 8.05 (d, *J* = 6.7 Hz, 1H), 8.00 (d, *J* = 8.0 Hz, 2H), 7.80 (d, *J* = 7.9 Hz, 2H), 7.70 – 7.59 (m, 7H), 6.76 (d, *J* = 8.8 Hz, 2H), 3.39 – 3.36 (m, *J* = 6.9 Hz, 4H), 3.34 (s, 4H), 1.92 (s, 4H), 1.12 (t, *J* = 7.0 Hz, 6H) ppm.

^{13}C NMR (151 MHz, d$_6$-DMSO): δ 150.6, 147.6 (2C), 145.9, 142.6, 132.2 (2C), 131.6, 130.6, 129.3 (2C), 127.8 (2C), 127.7 (2C), 126.6, 126.0 (2C), 124.6, 123.2, 118.6, 111.9 (2C), 105.1, 99.1, 48.8 (2C), 43.8 (2C), 25.0 (2C), 12.6 (2C) ppm. Triflate carbon was not detectable by NMR spectroscopy.

^{19}F NMR (565 MHz, d$_6$-DMSO): δ –77.8 ppm.

IR (neat) ν_{max}: 2971, 1715, 1659, 1612, 1593, 1537, 1510, 1465, 1403, 1374, 1354, 1268, 1223 cm^{-1}.

HRMS (ESI$^+$): calculated for [M–TfO$^-$]$^+$ ($C_{33}H_{34}N_3O^+$) requires m/z 488.2697, found m/z 488.2688.

(*E*)-7-(4-(Diethylamino)styryl)-3-phenyl-2-(pyrrolidin-1-yl)oxazolo[3,2-a]pyridin-4-ium trifluoromethanesulfonate (8d)

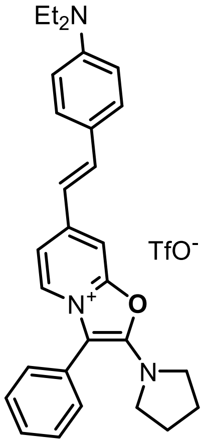

Yield: 53 mg, 90%, orange powder.

^{1}H NMR (600 MHz, d$_6$-DMSO): δ 8.22 (d, *J* = 1.4 Hz, 1H), 8.19 (d, *J* = 6.9 Hz, 1H), 7.72 (dd, *J* = 7.0, 1.6 Hz, 1H), 7.66 (d, *J* = 16.2 Hz, 1H), 7.64 – 7.61 (m, 5H), 7.50 (d, *J* = 9.0 Hz, 2H), 7.12 (d, *J* = 16.2 Hz, 1H), 6.72 (d, *J* = 9.0 Hz, 2H), 3.40 (q, *J* = 7.0 Hz, 4H), 3.33 – 3.30 (m, 4H), 1.93 – 1.90 (m, 4H), 1.12 (t, *J* = 7.0 Hz, 6H) ppm.

^{13}C NMR (151 MHz, d$_6$-DMSO): δ 150.0, 148.7, 147.5, 146.0, 137.4, 132.0 (2C), 130.3, 129.6 (2C), 129.2 (2C), 126.2, 123.2, 122.2, 118.4, 117.9, 111.4 (2C), 103.1, 99.1, 48.6 (2C), 43.8 (2C), 25.0 (2C), 12.5 (2C) ppm. Triflate carbon was not detectable by NMR spectroscopy.

^{19}F NMR (565 MHz, d$_6$-DMSO): δ –77.8 ppm.

IR (neat) ν_{max}: 2971, 1658, 1585, 1521, 1469, 1266, 1030, 705 cm^{-1}.

HRMS (ESI$^+$): calculated for [M–TfO$^-$]$^+$ ($C_{29}H_{32}N_3^+$) requires m/z 438.2540, found m/z 438.2540.

(*E*)-7-(4-(Dimethylamino)styryl)-3-phenyl-2-(pyrrolidin-1-yl)oxazolo[3,2-a]pyridin-4-ium trifluoromethanesulfonate (8e)

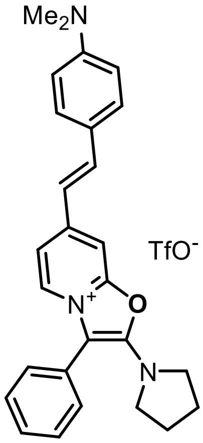

Prepared according to **General Procedure H**.

Yield: 60 mg, quantit., red powder. Product was obtained as a mixture of olefin isomers *E*/*Z* 1:0.8.

^{1}H NMR (600 MHz, CDCl$_3$): δ 7.89 (d, *J* = 6.7 Hz, 1H for minor), 7.76 (d, *J* = 6.0 Hz, 1H for major and 1H for minor), 7.72 – 7.66 (m, 3H for minor), 7.64 (d, *J* = 6.1 Hz, 2H for major), 7.56 (dt, *J* = 13.5, 6.1 Hz, 6H for major), 7.49 (d, *J* = 8.8 Hz, 1H for major and 1H for minor), 7.44 – 7.36 (m, 1H for major and 2H for minor), 7.23 (d, *J* = 8.5 Hz, 2H for minor), 6.91 (d, *J* = 16.0 Hz, 1H for major), 6.85 (d, *J* = 11.9 Hz, 1H for minor), 6.70 (d, *J* = 8.8 Hz, 2H for major), 6.64 (d, *J* = 8.6 Hz, 2H for minor), 6.32 (d, *J* = 11.9 Hz, 1H for minor), 3.42 (d, *J* = 5.8 Hz, 4H for each), 3.04 (s, 3H for each), 2.99 (s, 3H for each), 1.98 (d, *J* = 7.8 Hz, 4H for each) ppm.

^{13}C NMR (151 MHz, CDCl$_3$) for major isomer: δ 151.7, 150.9, 146.5, 146.0, 138.2, 132.5 (2C), 130.7 (2C), 129.7 (2C), 129.7 (2C), 125.2, 123.1, 121.1, 118.7, 112.2 (2C), 104.2, 99.4, 49.2 (2C), 40.3 (2C), 25.6 (2C) ppm. Triflate carbon was not detectable by NMR spectroscopy.

^{19}F NMR (565 MHz, CDCl$_3$): δ –78.2 ppm.

IR (neat) ν_{max}: 1659, 1587, 1524, 1505, 1483, 1469, 1444, 1366, 1353, 1274, 1262, 1222, 1190, 1161, 1119, 1030, 636 cm^{-1}.

HRMS (ESI$^+$): calculated for $[M–TfO^-]^+$ ($C_{27}H_{28}N_3O^+$) requires m/z 410.2227, found m/z 410.2240.

(*E*)-3-Phenyl-2-(pyrrolidin-1-yl)-7-(2-(2,3,6,7-tetrahydro-1H,5H-pyrido[3,2,1-ij]quinolin-9-yl)vinyl)oxazolo[3,2-a]pyridin-4-ium trifluoromethanesulfonate (8f)

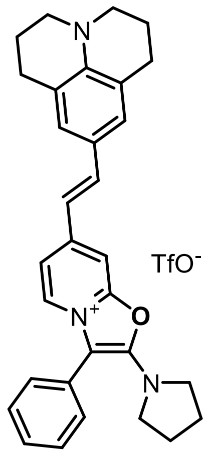

Prepared according to **General Procedure H**.

Yield: 37 mg, 60%, red powder.

^{1}H NMR (600 MHz, d$_6$-DMSO): δ 8.19 – 8.14 (m, 2H), 7.68 (dd, *J* = 7.1, 1.4 Hz, 1H), 7.64 – 7.60 (m, 5H), 7.56 (d, *J* = 16.1 Hz, 1H), 7.09 – 7.04 (m, 3H), 3.32 – 3.30 (m, 4H), 3.24 – 3.2 (m, 4H), 2.70 (t, *J* = 6.3 Hz, 4H), 1.92 – 1.89 (m, 4H), 1.89 – 1.85 (m, 4H) ppm.

^{13}C NMR (151 MHz, d$_6$-DMSO): δ 149.9, 147.5, 146.1, 144.2, 137.7, 132.0 (2C), 130.2, 129.2 (2C), 126.9 (2C), 126.2, 123.2, 122.0, 120.7 (2C), 118.2, 117.4, 102.8, 99.1, 49.2 (2C), 48.6 (2C), 27.2 (2C), 24.9 (2C), 21.1 (2C) ppm. Triflate carbon was not detectable by NMR spectroscopy.

^{19}F NMR (565 MHz, d$_6$-DMSO): δ –77.8 ppm.

IR (neat) ν_{max}: 2358, 1659, 1516, 1309, 1262, 1182, 1030, 636 cm^{-1}.

HRMS (ESI$^+$): calculated for [M–TfO$^-$]$^+$ ($C_{31}H_{32}N_3O^+$) requires m/z 462.2540, found m/z 462.2538.

(*E*)-7-(4-Morpholinostyryl)-3-phenyl-2-(pyrrolidin-1-yl)oxazolo[3,2-a]pyridin-4-ium trifluoromethanesulfonate (8g)

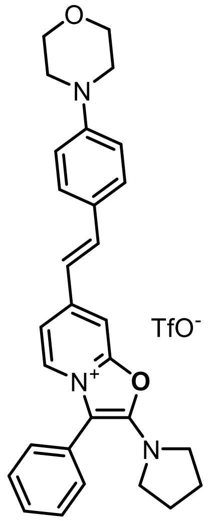

Prepared according to **General Procedure H**.

Yield: 54 mg, 90%, orange oil. Product was obtained as a mixture of olefin isomers *E*/*Z* 1:0.17.

^{1}H NMR (600 MHz, CDCl$_3$): δ 7.86 (d, *J* = 6.8 Hz, 1H for major), 7.81 (s, 1H for major), 7.75 (d, *J* = 6.8 Hz, 1H for minor), 7.68 – 7.61 (m, 1H for major and 3H for minor), 7.59 – 7.48 (m, 7H for major and 3H for minor), 7.38 (d, *J* = 16.1 Hz, 1H and 3H for minor), 7.33 (d, *J* = 6.3 Hz, 1H for minor), 7.23 (d, *J* = 8.6 Hz, 2H for minor), 6.96 (d, *J* = 16.1 Hz, 1H for major), 6.86 (d, *J* = 8.8 Hz, 2H for major), 6.84 – 6.81 (m, 3H for minor), 6.40 (d, *J* = 12.0 Hz, 1H for minor), 3.88 – 3.83 (m, 4H for major), 3.83 – 3.80 (m, 4H for minor), 3.38 (d, *J* = 6.3 Hz, 4H for each), 3.26 – 3.21 (m, 4H for major), 3.21 – 3.18 (m, 4H for minor), 1.96 (t, *J* = 6.6 Hz, 4H for each) ppm.

^{13}C NMR (151 MHz, CDCl$_3$, major): δ 152.2, 150.8, 147.5, 145.9, 137.3, 132.3 (2C), 130.8, 129.6 (2C), 129.5 (2C), 126.5, 125.2, 122.9, 120.2, 119.1, 114.9 (2C), 104.7, 99.3, 66.8 (2C), 49.1 (2C), 48.2 (2C), 25.5 (2C) ppm. Triflate carbon was not detectable by NMR spectroscopy.

^{19}F NMR (565 MHz, CDCl$_3$): δ –78.2 ppm.

IR (neat) ν_{max}: 1660, 1595, 1517, 1470, 1447, 1381, 1350, 1276, 1262, 1228, 1166, 1149, 1121, 1030, 927, 736, 707, 637 cm^{-1}.

HRMS (ESI⁺): calculated for $[M–TfO^-]^+$ ($C_{29}H_{30}N_3O_2^+$) requires m/z 452.2333, found m/z 452.2347.

7-((1*E*,3*E*)-4-(4-(Diethylamino)phenyl)buta-1,3-dien-1-yl)-3-phenyl-2-(pyrrolidin-1-yl)oxazolo[3,2-a]pyridin-4-ium trifluoromethanesulfonate (8h)

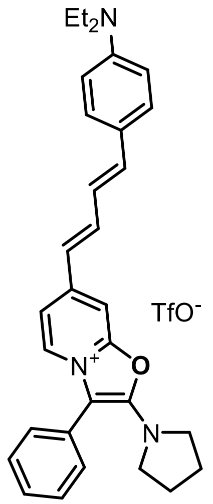

Prepared according to **General Procedure H**.

Yield: 42 mg, 68%, red oil. Product was obtained as a mixture of olefin isomers 1:0.1. Only the major one is assigned.

^{1}H NMR (600 MHz, $CDCl_3$): δ 7.89 (d, *J* = 6.6 Hz, 1H), 7.71 (s, 1H), 7.62 – 7.52 (m, 6H), 7.34 (d, *J* = 8.7 Hz, 2H), 7.29 – 7.25 (m, 1H), 6.82 (d, *J* = 15.3 Hz, 1H), 6.73 (dd, *J* = 15.2, 10.6 Hz, 1H), 6.62 (d, *J* = 8.7 Hz, 2H), 6.53 (d, *J* = 15.2 Hz, 1H), 3.46 – 3.32 (m, 8H), 1.94 (t, *J* = 6.5 Hz, 4H), 1.18 (t, *J* = 7.0 Hz, 6H) ppm.

^{13}C NMR (151 MHz, $CDCl_3$): δ 150.7, 148.6, 147.6, 146.0, 140.4, 139.4, 132.4 (2C), 130.8, 129.6 (2C), 129.2 (2C), 125.2, 123.9, 123.7, 123.0, 122.6, 118.8, 111.6 (2C), 104.3, 99.3, 49.1 (2C), 44.6 (2C), 25.5 (2C), 12.8 (2C) ppm. Triflate carbon was not detectable by NMR spectroscopy.

^{19}F NMR (565 MHz, $CDCl_3$): δ –78.2 ppm.

IR (neat) ν_{max}: 1738, 1723, 1699, 1659, 1579, 1520, 1470, 1403, 1376, 1351, 1267, 1232, 1213, 1204, 1191, 1145, 1115, 1076, 1031, 1007, 869, 816, 802, 707 cm^{-1}.

HRMS (ESI⁺): calculated for $[M–TfO^-]^+$ ($C_{31}H_{34}N_3O^+$) requires m/z 464.2696, found m/z 464.2706.

3-Phenyl-2-(pyrrolidin-1-yl)-7-((1*E*,3*E*)-4-(2,3,6,7-tetrahydro-1H,5H-pyrido[3,2,1-ij]quinolin-9-yl)buta-1,3-dien-1-yl)oxazolo[3,2-a]pyridin-4-ium trifluoromethanesulfonate (8i)

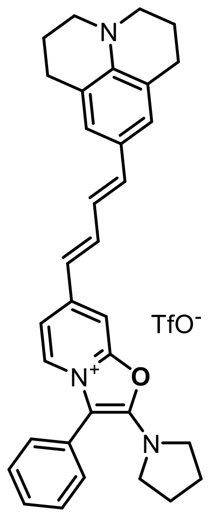

Prepared according to **General Procedure H**.

Yield: 9 mg, 47%, red powder.

^{1}H NMR (600 MHz, d_6-DMSO): δ 8.20 (s, 1H), 8.16 (d, *J* = 6.8 Hz, 1H), 7.66 (d, *J* = 7.0 Hz, 1H), 7.65 – 7.61 (m, 5H), 7.54 (dd, *J* = 15.3, 10.7 Hz, 1H), 6.96 (s, 2H), 6.85 (dd, *J* = 15.2, 10.7 Hz, 1H), 6.75 (d, *J* = 15.3 Hz, 1H), 6.67 (d, *J* = 15.3 Hz, 1H), 3.32 – 3.29 (m, 4H), 3.21 – 3.16 (m, 4H), 2.68 (t, *J* = 6.2 Hz, 4H), 1.91 (t, *J* = 6.5 Hz, 4H), 1.89 – 1.84 (m, 4H) ppm.

^{13}C NMR (151 MHz, d_6-DMSO): δ 150.1, 147.3, 145.2, 143.6, 139.7, 138.9, 132.0 (2C), 130.3, 129.2 (2C), 126.2 (2C), 126.1, 123.8, 123.2, 122.9, 122.4, 120.8 (2C), 118.3, 103.5, 99.2, 49.2 (2C), 48.6 (2C), 27.1 (2C), 24.9 (2C), 21.2 (2C) ppm. Triflate carbon was not detectable by NMR spectroscopy.

^{19}F NMR (565 MHz, d_6-DMSO): δ –77.8 ppm.

IR (neat) ν_{max}: 1657, 1574, 1463, 1262, 1030, 637 cm^{-1}.

HRMS (ESI^{+}): calculated for $[M–TfO^{-}]^{+}$ ($C_{33}H_{34}N_3O^{+}$) requires m/z 488.2697, found m/z 488.2696.

7-(4-(Diethylamino)phenyl)-3-phenyl-2-(pyrrolidin-1-yl)thiazolo[3,2-a]pyridin-4-ium trifluoromethanesulfonate (9a)

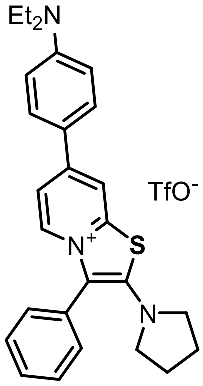

Prepared according to **General Procedure H**.

Yield: 52 mg, >99%, orange solid.

^{1}H NMR (600 MHz, d$_6$-DMSO): δ 8.82 (s, 1H), 8.01 (d, *J* = 7.2 Hz, 1H), 7.96 – 7.92 (m, 1H), 7.77 (d, *J* = 8.9 Hz, 2H), 7.69 – 7.61 (m, 7H), 6.83 (d, *J* = 9.0 Hz, 2H), 3.44 (dd, *J* = 13.9, 6.9 Hz, 5H), 3.16 – 3.11 (m, 5H), 1.88 (s, 5H), 1.14 (t, *J* = 7.0 Hz, 6H) ppm.

^{13}C NMR (151 MHz, d$_6$-DMSO): δ 149.3, 144.2, 142.8, 142.5, 132.9 (2C), 130.8, 130.1, 129.4 (2C), 128.4 (2C), 127.3, 119.5, 118.3, 115.5, 112.4, 111.8 (2C), 52.2 (2C), 43.8 (2C), 25.4 (2C), 12.5 (2C) ppm. Triflate carbon was not detectable by NMR spectroscopy.

^{19}F NMR (565 MHz, d$_6$-DMSO): δ –77.8 ppm.

IR (neat) ν_{max}: 2971, 1714, 1592, 1533, 1516, 1492, 1459, 1400, 1354, 1319, 1258, 1219, 1153, 1093, 1030 cm^{-1}.

HRMS (ESI$^+$): calculated for [M–TfO$^-$]$^+$ ($C_{27}H_{30}N_3S^+$) requires m/z 428.2155, found m/z 428.2150.

(*E*)-7-(4-(Diethylamino)styryl)-3-phenyl-2-(pyrrolidin-1-yl)thiazolo[3,2-a]pyridin-4-ium trifluoromethanesulfonate (9b)

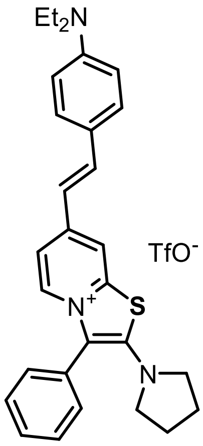

Prepared according to **General Procedure H**.

Yield: 34 mg, 71%, brown powder. As a mixture of double bond isomers E/Z 4:1. For simplicity, only the major isomer is described.

^{1}H NMR (600 MHz, $CDCl_3$): δ 8.46 (s, 1H), 7.81 (d, *J* = 7.1 Hz, 1H), 7.62 – 7.57 (m, 2H), 7.54 (dd, *J* = 6.5, 3.0 Hz, 1H), 7.46 (dt, *J* = 16.0, 6.8 Hz, 3H), 6.84 (d, *J* = 16.1 Hz, 1H), 6.65 (d, *J* = 8.9 Hz, 1H), 3.41 (q, *J* = 7.1 Hz, 2H), 3.17 (t, *J* = 6.6 Hz, 2H), 1.93 (dd, *J* = 8.1, 5.1 Hz, 2H), 1.20 (t, *J* = 7.0 Hz, 3H) ppm.

^{13}C NMR (151 MHz, $CDCl_3$): δ 149.3, 145.5, 143.3, 142.5, 138.2, 133.0 (2C), 131.2, 130.0 (2C), 129.9 (2C), 128.7, 127.3, 122.7, 119.2, 118.1, 117.2, 112.8, 111.6 (2C), 52.8 (2C), 44.6 (2C), 26.0 (2C), 12.8 (2C) ppm. Triflate carbon was not detectable by NMR spectroscopy.

^{19}F NMR (565 MHz, $CDCl_3$): δ –78.1 ppm.

IR (neat) ν_{max}: 1716, 1581, 1522, 1359, 1268, 1223, 734 cm^{-1}.

HRMS (ESI^{+}): calculated for $[M–TfO^{-}]^{+}$ ($C_{29}H_{32}N_3S^{+}$) requires m/z 454.2312, found m/z 454.2305.

6. Solvatochromism and pH studies

Note: UV absorbance of PyrAtes was measured on a Thermo Fisher Scientific G10S spectrophotometer at concentrations of 1 µM, 5 µM, 10 µM and 20 µM in the designated solvent. Fluorescence was recorder on a SHIMADZU RF-6000 spectrofluorometer at concentration of 1 µM in the same solvent. For the evaluation of pH effect, in-house-made buffer aqueous solutions were used.

6.1. Solvatochromic effect on 7a

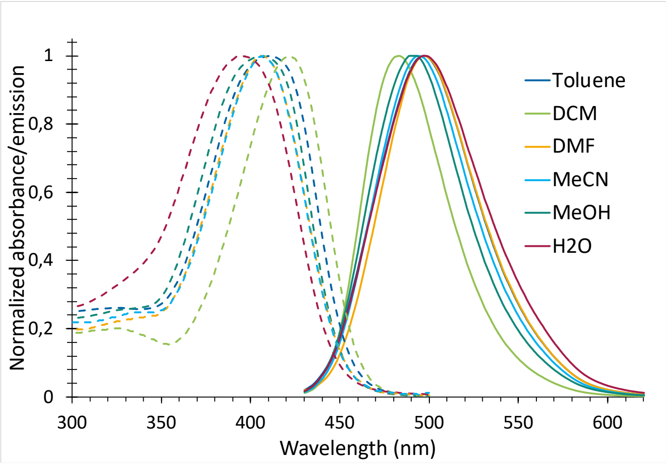

*Figure S1. Normalized absorption and emission of **7a** in various solvents for evaluation of its solvatochromism effect.*

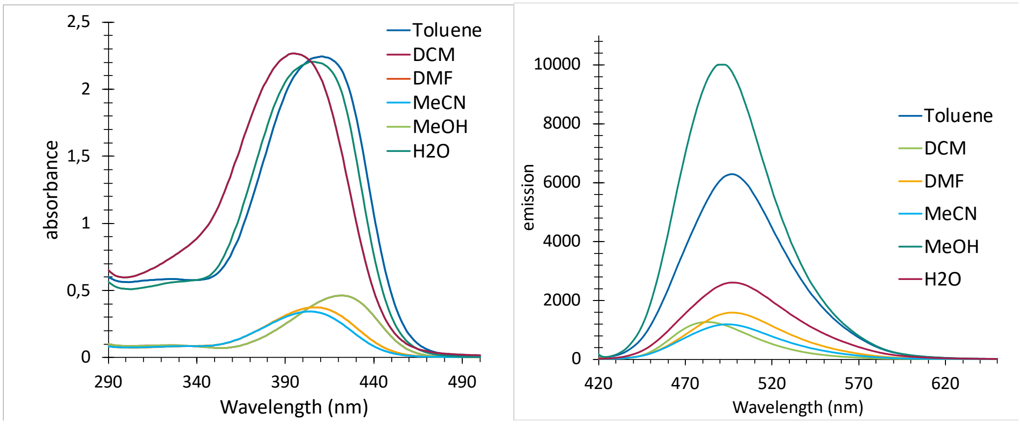

*Figure S2. Absolute absorption and emission profile of **7a** in response to various solvents for the study of its solvatochromic effect.*

6.2. pH Effect on 7a

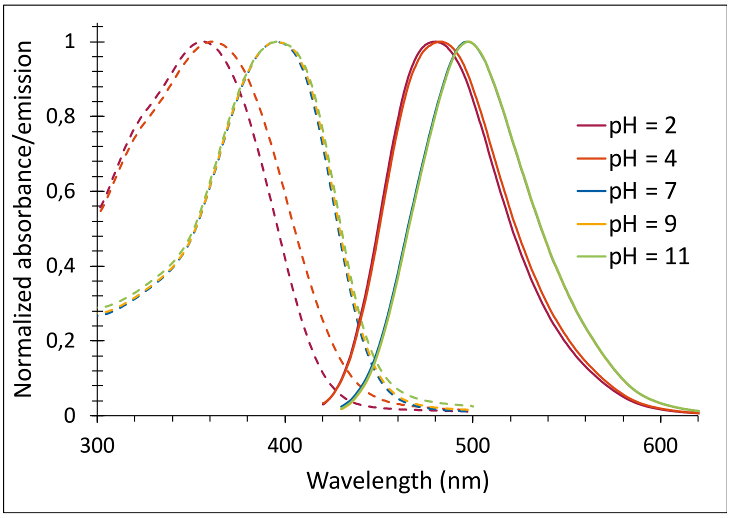

Figure S3. *The effect of pH on PyrAte **7a**. Normalized values of absorption and emission.*

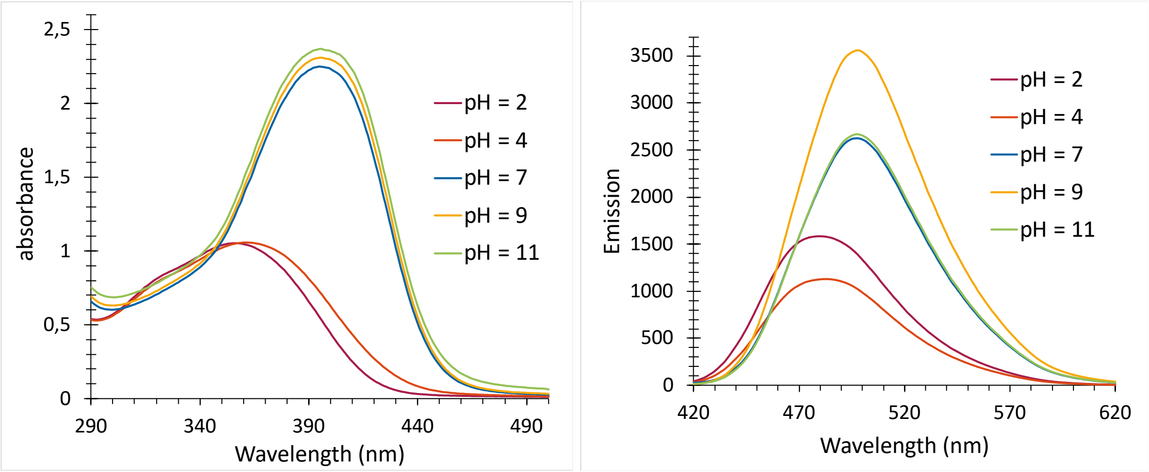

Figure S4. *Absolute absorption and emission profile of **7a** versus different pH values.*

7. X-Ray Analysis of 5f

The X-ray intensity data were measured on Bruker D8 Venture diffractometer equipped with multilayer monochromator, Cu K/α INCOATEC micro focus sealed tube and Oxford cooling systems. The structure was solved by *Direct methods.* Non-hydrogen atoms were refined with *anisotropic displacement parameters*. Hydrogen atoms were inserted at calculated positions and refined with riding model. The following software was used: *Bruker SAINT software package (Bruker SAINT v8.40B Copyright© 2018 Bruker AXS)* using a narrow-frame algorithm for frame integration, *SADABS* (*Sheldrick, G. M. (1996), SADABS*) for absorption correction, *OLEX2*[26] for structure solution, refinement, molecular diagrams and graphical user-interface, *Shelxle*[27] for refinement and graphical user-interface *SHELXS-2015* (*Sheldrick, G. M. (2015), SHELXS v 2016/4 University of Göttingen, Germany*) for structure solution, *SHELXL-2015 (Sheldrick, G. M. (2015), SHELXL v 2016/4 University of Göttingen, Germany*) for refinement, *Platon*[28] for symmetry check. Experimental data and CCDC-Codes Experimental data (Available online: http://www.ccdc.cam.ac.uk/conts/retrieving.html) can be found in Table S4. Crystal data, data collection parameters, and structure refinement details are given in Table S5. Asymmetric Unit visualized in Figure S5. Figures S6 and S7 support information about the data quality.

Table S4. *Experimental parameter and CCDC-Code.*

Sample	Machine	Source	Temp. (K)	Detector Distance (nm)	Time/ Frame (s)	#Frames	Frame width (°)	CCDC
1	Bruker D8	Cu	100	37	15	1649	0.5	2290600

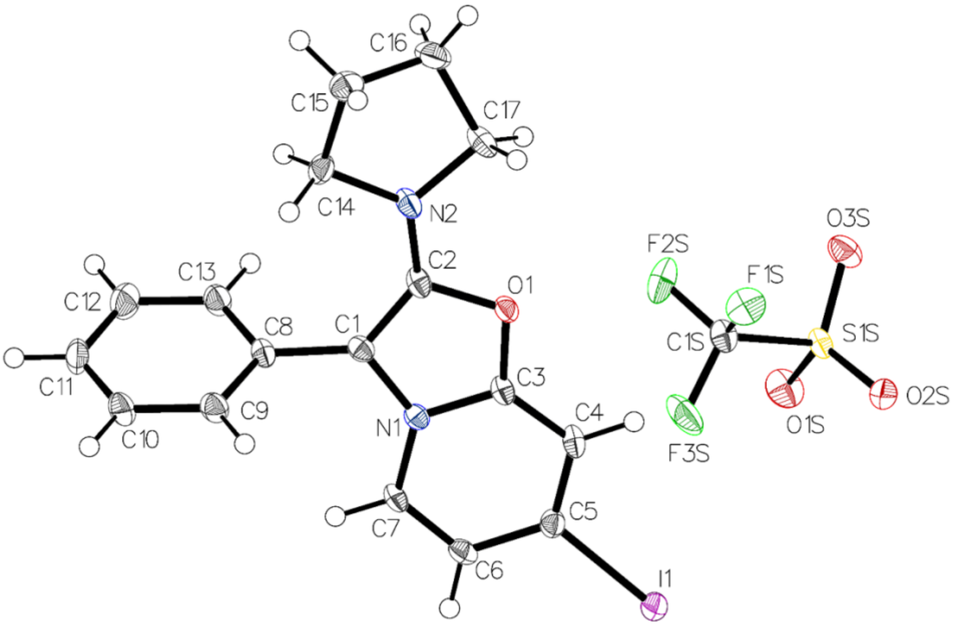

Figure S5 Asymmetric Unit of drawn with 50% displacement ellipsoid. The bond precision for C-C single bonds is 0.0056 Å. The packing is unobtrusive.

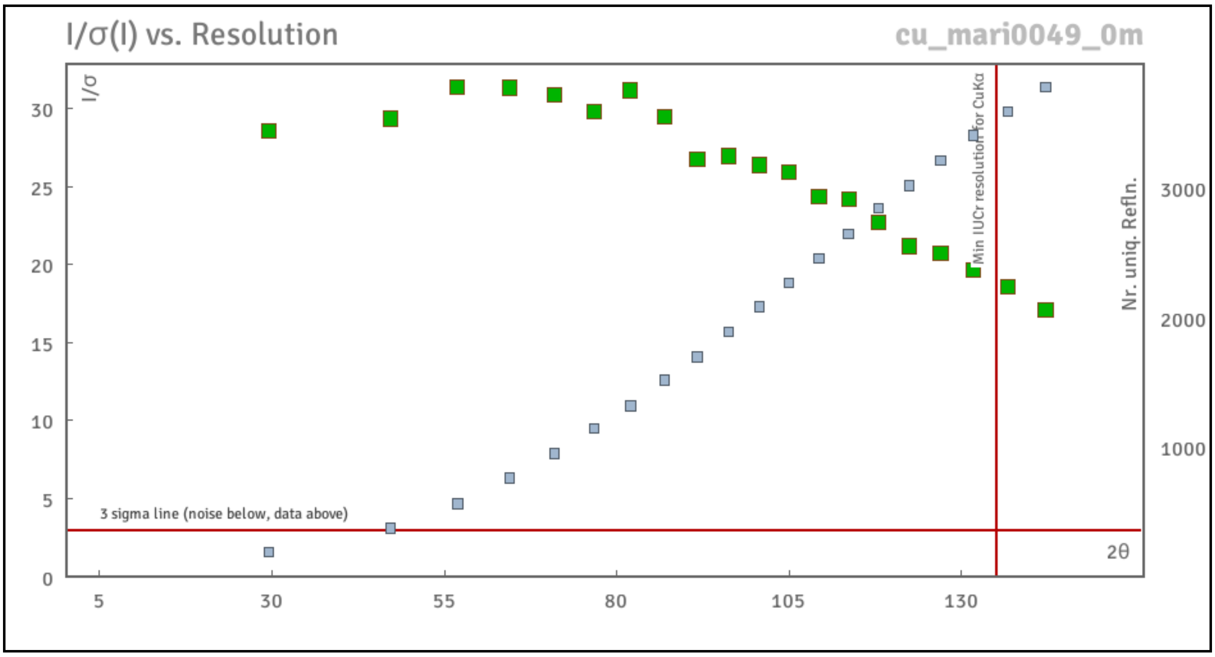

Figure S6. *Data quality I: All data are above the "three sigma" line along the min IUCR definition.*

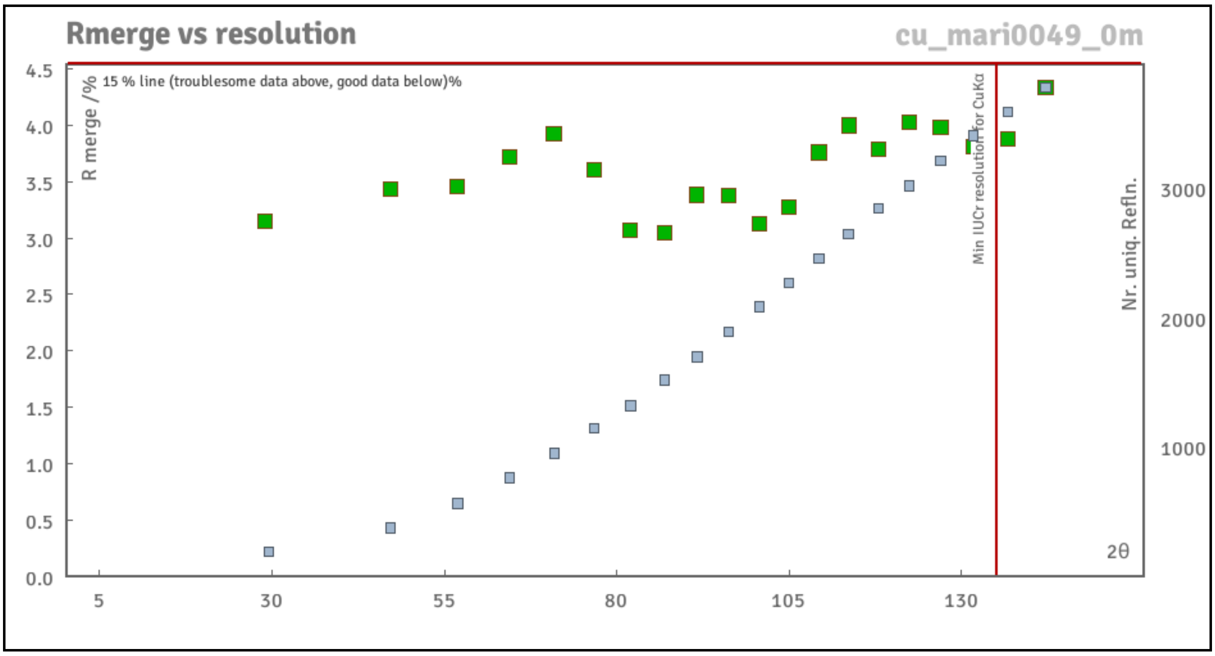

Figure S7. *Data quality II: All data are below the recommendation 15% Rmerge line along the min IUCR definition.*

Table S5. Sample and crystal data, Data collection and structure refinement

Identification code	cu_mari0049_0m
Empirical formula	$C_{18}H_{16}N_2O_4F_3SI$
Formula weight	540.29
Temperature/K	100.00
Crystal system	monoclinic
Space group	$P2_1/c$
a/Å	9.2259(3)
b/Å	10.4310(3)
c/Å	21.1643(7)
α/°	90
β/°	100.5890(10)
γ/°	90
Volume/Å^3	2002.07(11)
Z	4

ρ_{calc}g/cm^3	1.792
μ/mm^{-1}	14.056
F(000)	1064.0
Crystal size/mm^3	0.347 × 0.141 × 0.054
Radiation	CuKα (λ = 1.54178)
2Θ range for data collection/°	8.5 to 145.184
Index ranges	-11 ≤ h ≤ 11, -11 ≤ k ≤ 12, -26 ≤ l ≤ 25
Reflections collected	12383
Independent reflections	3807 [R_{int} = 0.0350, R_{sigma} = 0.0329]
Data/restraints/parameters	3807/0/262
Goodness-of-fit on F^2	1.102
Final R indexes [I>=2σ (I)]	R_1 = 0.0428, wR_2 = 0.1391
Final R indexes [all data]	R_1 = 0.0453, wR_2 = 0.1427
Largest diff. peak/hole / e Å^{-3}	1.44/-2.30

8. Internalization and colocalization studies in HEK293 cells

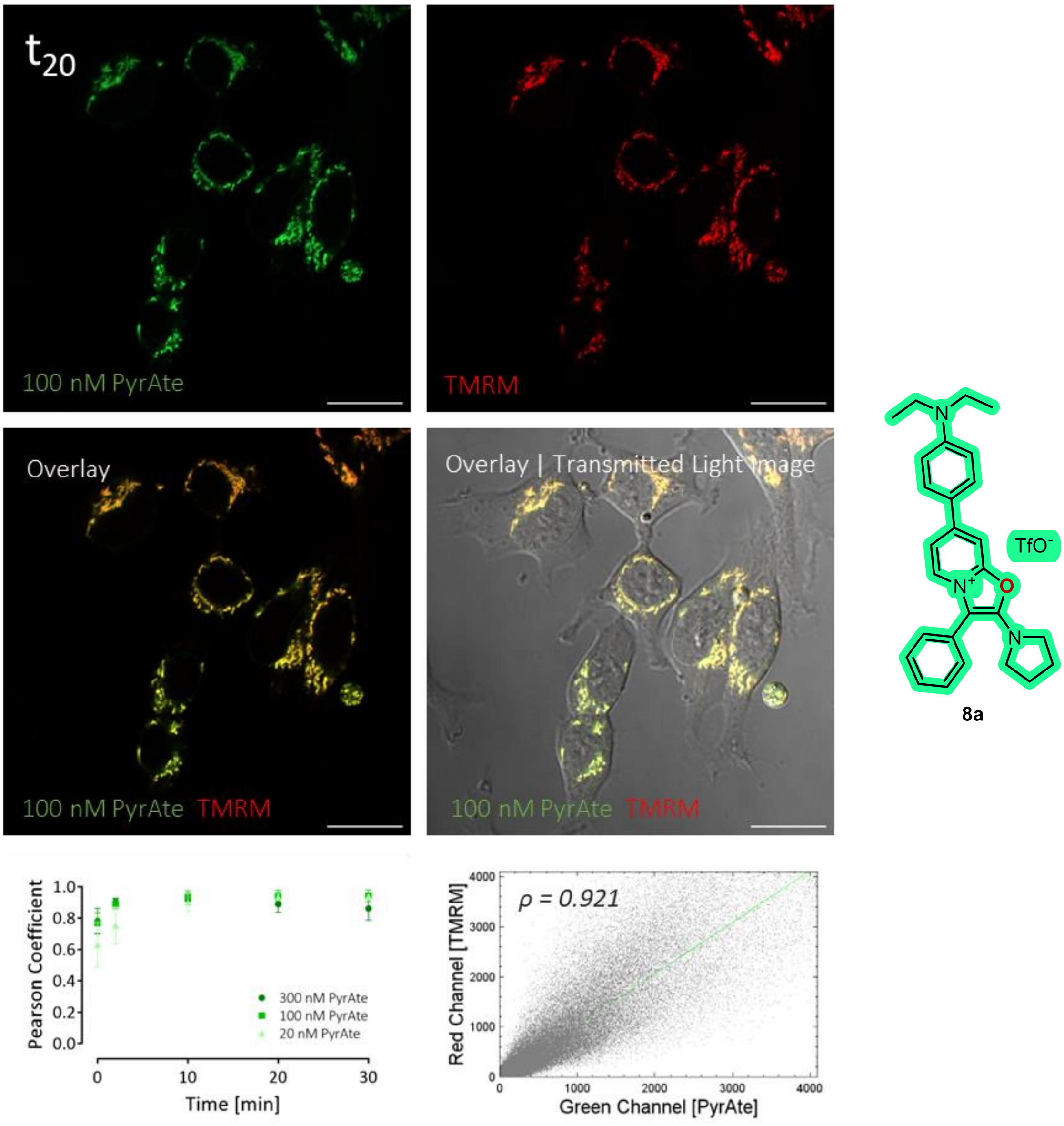

Figure S8. *PyrAte compound **8a** (100 nM, green) internalization and co-localization with the mitochondrial marker tetramethylrhodamine (TMRM, red) after 20 minutes (t20) in HEK293 cells. Flurophores were excited at a wavelength of 407.5 nm (PyrAte) or 560.2 nm (TMRM). Co-localization was evaluated with the Pearson's correlation coefficient (ρ), yielding a strong correlation with ρ=0.921 [N1]. An overview of Pearson's correlation coefficient for three different concentrations (20 nM light green, 100 nM green, and 300 nM dark green) over several time points reaching from 0-30 minutes shows strong or very strong correlation for all tested conditions from 2 minutes onward (n=3). Scale bar is 20 µm.*

9. Cytotoxicity studies

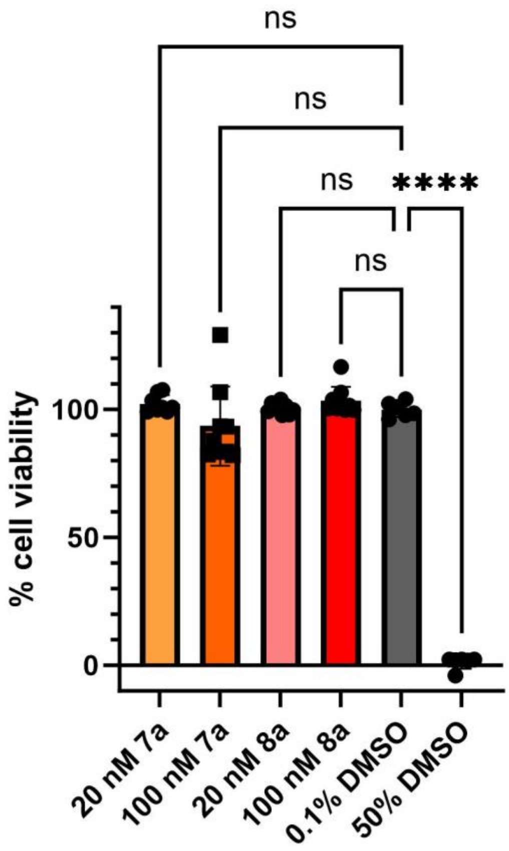

Figure S9. *Viability of HEK293 cells after 60 min incubation of PyrAtes* ***7a*** *and* ***8a*** *(20 nM and 100 nM concentrations) tested using AquaBluer™ cytotoxicity assay. Data shown as mean with individual values, standard deviation and **** P < 0.0001 as determined using one-way ANOVA (N = 3, performed in triplicates). Overall there is a non-significant difference in the % cell viability of cells treated with PyrAtes: 102 ± 3.4, 93.5 ± 15.5, 100 ± 2.1, and 103.5 ± 5.4 for 20 nM* ***7a****, 100 nM* ***7a****, 20 nM* ***8a****, and 100 nM* ***8a****, respectively; in comparison to the control vehicle condition 0.1% DMSO with 100 ± 3.0 % cell viability. This confirms that cells remain viable throughout the duration of imaging using the optimized conditions. DMSO (50% v/v in DMEM) was used as control: 1.15 ± 2.5.*

10. Resistance to oxidation

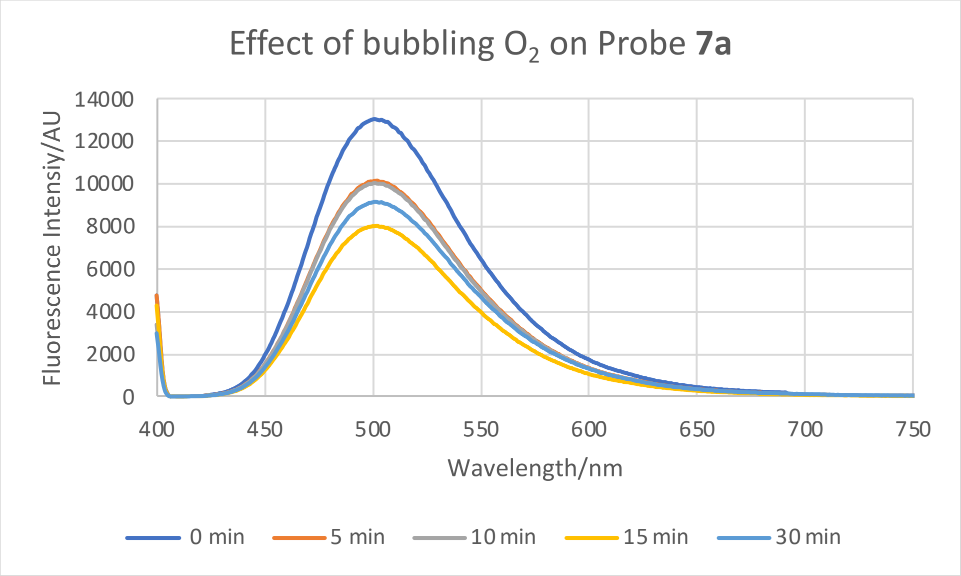

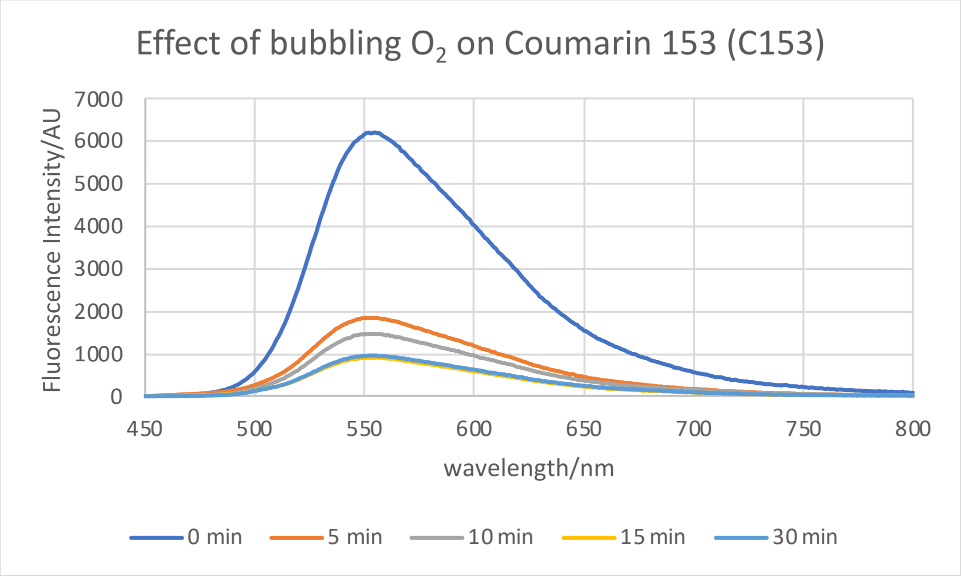

Figure S10. *Effect of bubbling O2 on PyrAte* ***7a*** *(top), in comparison to coumarin 153 (bottom). Oxygen gas was bubbled through a 5 µM solution of the probe in 99:1 (v/v) PBS:DMSO in a round-bottom flask. Aliquots are taken out from the flask at 0, 5, 10, 15 and 30 minutes. UV-Vis absorption spectra are immediately measured at 5 µM concentration to identify maximum absorption wavelength* λ_{max}*. The aliquot is then diluted to 1 µM for measurement of fluorescence, excited at the identified* λ_{max}*. Both* ***7a*** *and coumarin 153 have their fluorescence intensity quenched by molecular oxygen. While* ***7a*** *shows a steady and slow erosion of fluorescence intensity, coumarin 153 is quenched rather quickly—fluorescence intensity decreases to less than a third of the non-oxygenated solution within 5 min.*

11. Computational Studies

11.1. Computational Methods

In order to obtain a model for predicting the emission energies, the following computational procedure is followed. First, a conformational sampling with Schrödinger's MacroModel v12.4[29] is performed for the compounds **4a**, **5a**, **6a**, **7a**, **8a**, **9a**, **7g**, **8d**, and **9b**. Subsequently, the compounds are optimized on the first electronic excited state S_1 at the TDA-B3LYP-D3/def2-SVP,def2-TZVP(for sulfur containing molecules)@IEFPCM(methanol)[30–35] level of theory with 5 states using Gaussian 16[36] (Cartesian coordinates of the mentioned compounds can be found in Section 7.2). Using the resulting S_1 optimized geometries, an ADC(2)/def2-SVP,def2-TZVP (for sulfur containing molecules)@COSMO(methanol,ε=32.613)[37–39] single point calculation including 20 states is carried out using Turbomole 7.0,[40] to obtain reference electronic excited state values. Figure S11A shows that the calculated maximum emission energies and the experimental energies (see also Table S6) are in reasonable agreement, justifying the suitability of both ADC(2) and TDA-DFT calculations. Further, we investigated the correlation of the S_1 HOMO LUMO gap with the experimental emission energy, E_{em} (Figure S11B). The nice correlation (R^2 = 0.972) incited us to compare the S_1 HOMO-LUMO-gap with the one obtained at the ground state S_0 equilibrium geometry (obtained at the B3LYP-D3/def2-SVP,def2-TZVP(for sulfur containing molecules)@IEFPCM(methanol) level of theory, for the Cartesian coordinates see Section 7.2). In view of the also excellent linear correlation (R^2 = 0.904) found (Figure S11C), we designed the protocol sketched in Figure S11D for high-throughput prediction of the E_{em} of novel compounds. As an initial test, the absorption spectra of 44 compounds were explicitly calculated at the TD B3LYP D3/def2 SVP@IEFPCM(methanol) level of theory with 30 states using Gaussian 16 using the ground state geometry. The resulting energies were compared to those obtained from the fast screening approach (Figure S11D) and available experimental values (Figure 4 and Table S7). The excellent correlation enabled us to use this high-throughput scheme for discovery of new PyrAte compounds with optimal absorption and emission properties.

Table S6. *Experimental and calculated (TDA-B3LYP-D3 and ADC(2)) maximum emission energies.*
**basis set: def2-TZVP*

Compound	E_{em} / eV Exp.	Calc. TDA-DFT	Calc. ADC(2)
**4a**	2.88	2.77	3.08
**5a**	2.76	2.51	2.92
**6a***	2.54	2.33	2.28
**7a**	2.51	2.71	2.71
**8a**	2.44	2.57	2.48
**9a***	2.25	2.51	2.27
**7g**	2.17	2.33	2.37
**8d**	2.07	2.24	2.23
**9b***	2.02	2.20	2.06

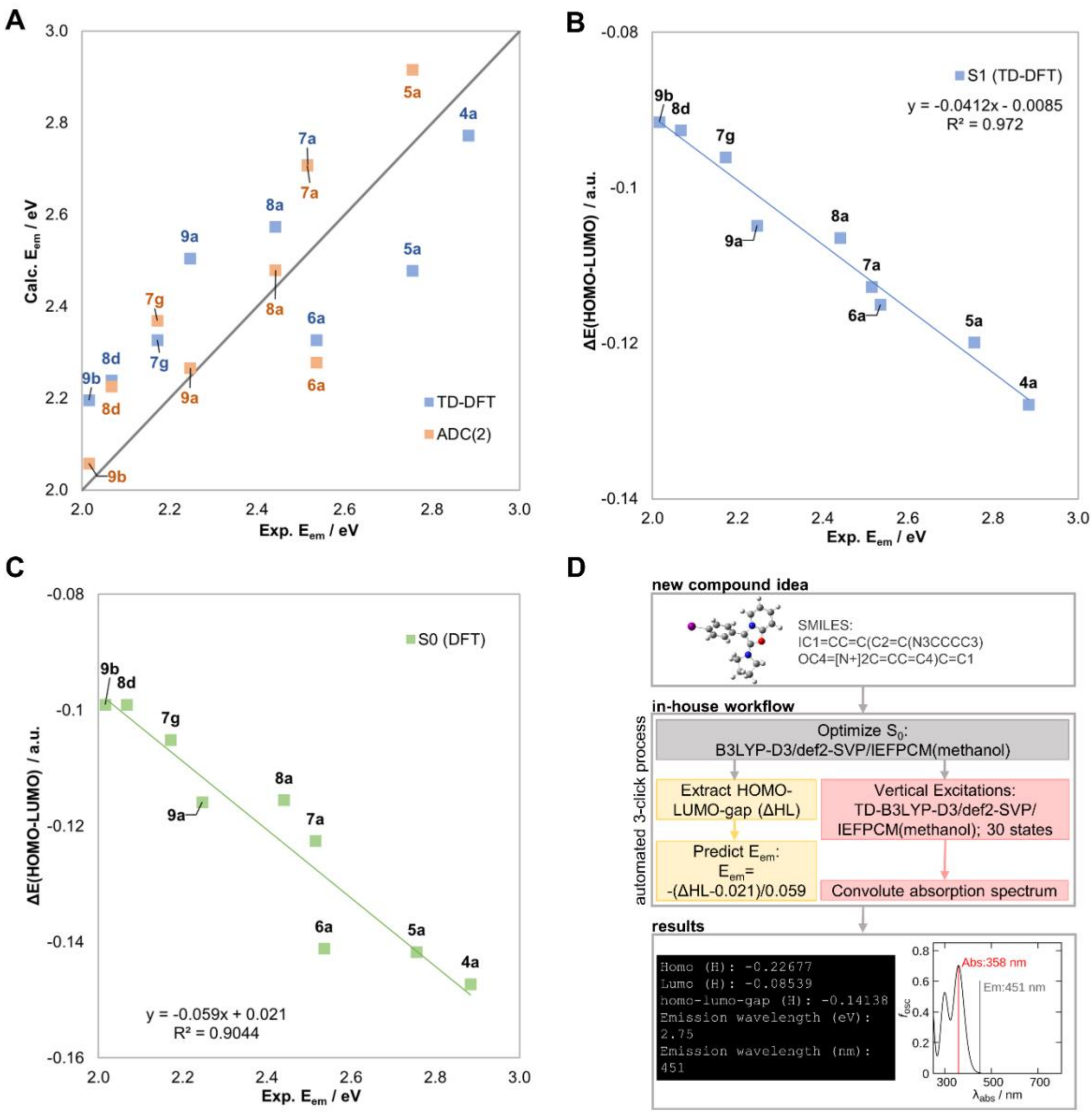

Figure S11. A) Calculated maximum emission energies against experimental values. Level of theory: TDA-B3LYP-D3/def2-SVP@IEFPCM(methanol) and ADC(2)/def2-SVP@COSMO(methanol); def2-TZVP for sulfur-containing compounds. B) S_1 HOMO-LUMO-gap against experimental emission energy. Level of theory: TDA-B3LYP-D3/def2-SVP@IEFPCM(methanol); def2-TZVP for sulfur-containing compounds. C) S_0 HOMO-LUMO-gap against experimental emission energy. Level of theory: B3LYP-D3/def2-SVP@IEFPCM(methanol); def2-TZVP for sulfur-containing compounds. D) Computational workflow for the emission and absorption wavelength prediction of novel PyrAte compounds.

For selected compounds **4a**, **7a**, **7h**, **7l**, **5a**, **6a**, **4h**, **4i** we show the Natural Transition Orbitals (NTOs)[41,42] of emission in Figure S12A,D,F. NTOs summarize in a one-electron representation the changes of electron density upon (de)excitation. During emission, the electron is vertically deexcited from the S_1 minimum to the S_0* (= S_0 electronic state at the S_1 minimum geometry) leaving behind a "hole" at the S_1. The S_1-NTO therefore represents the electron density "hole" that is left by the electron, while the S_0*-NTO represents the electron density at the S_0 state after the "electron" is absorbed. For the investigated molecules, the S_1 NTO nicely correlates with the LUMO and the S_0* NTO with the HOMO frontier orbitals.

An alternative way of representing electron density changes is to use electron-hole-correlation plots[43,44] (checkered plots in Figures S12C,E,G). For this analysis, the molecule is divided into fragments –in this case, four labelled by f1 to f4 in Figure S12B. This fragmentation scheme allows to easily inspect the effect of substitution at any of the moieties. The checkered plots are arranged such that they numerically depict the location of the "hole" and the "electron" at each of the fragments. Such electron-hole-correlation plots enable a straightforward and systematic assessment of the influence of substitution, upon varying fragments f4 (C), f3 (E), and f2 (G). When the pyridinium moiety (f4) is modified within the series **4a** → **7a** → **7h** → **7l**, the plots reveal the "hole" moving from f3 to a mixture of f3/f4 and the "electron" moving from f2 to f4 (Figure S12C). In the case of the heteroatom Z substitution (f3, **4a** (N-$(CH_2)_2$-Ph) → **5a** (O) → **6a** (S)) the "hole" stays mainly located on f3, while the "electron" switches from f2/f3 to f3 back to f2/f3 (Figure S12E). When the amino group (f2) is substituted (**4a** (pyrrolidine) → **4h** (NEt_2) → **4i** (azetidine)), the "hole" is again at the f3 fragment, while the "electron" spreads out more and more from f2/f3 to f1/f2/f3 (Figure S12G). Moreover, exciton sizes[45,46] can be calculated, as the root-mean-square of the electron-hole separation, revealing the degree of orbital distribution on the molecular scaffold. Both, the exciton size (Å) and the experimental maximum emission wavelength (nm) are plotted for selected compounds (Figures 4 and S12C,E,G). The impact of the ligand substitution in terms of a strong (f4), small inverse (f3) and no (f2) correlation is partially discussed in the main text.

For Z, the decrease of the exciton size looks inversely proportional to the maximum of the emission wavelength as can be seen in the series **4a** (Z=NR_2) → **5a** (Z=O) → **6a** (Z=S). The change of the heteroatom on Z is the reason why the exciton size changes in the series. From the molecular orbital picture, the sulfur atom can expand the octet rule and can use the 3d orbitals for electronic excitations, whereas oxygen and nitrogen (2nd period elements) cannot do that. As a consequence, the molecular orbital overlapping and electronic delocalization may change from **4a**/**5a** to **6a**. This can be barely seen on the NTOs we plot in Figure S12. Thus, and to quantify this effect, we divided f3 into two virtual fragments (f3a and f3b) to check the effect of Z on the electron delocalization (see Figure S13).

Looking at the hole-electron matrices, it can be seen that whereas for **4a** and **5a** the hole locates mostly on f3a and the electron on f3b (out-diagonal elements, darker color), in **6a** the relative population on f3b increases for both hole and electron delocalization. Indeed, it also increases the local excitations within f3a (diagonal element). Thus, electron and hole are closer now. This means that the extra orbitals in the sulfur atom in **6a** helps to a better overlapping between molecular orbitals and a reduction of the delocalization of hole-electron. Finally, a calculation of the total delocalization (DEL value in the TheoDORE program suite),[47] as the number of fragments where hole-electron are distributed, confirms how the exciton size is reduced when moving from **4a**(2.02)/**5a**(2.02) to **6a** (1.76).

All results in Figures 4, S12 and S13 are obtained from the analysis of wavefunctions obtained at the TDA-B3LYP-D3/def2-SVP (def2-TZVP for sulfur containing molecules)@IEFPCM(methanol) level of theory using the TheoDORE program suite.[47]

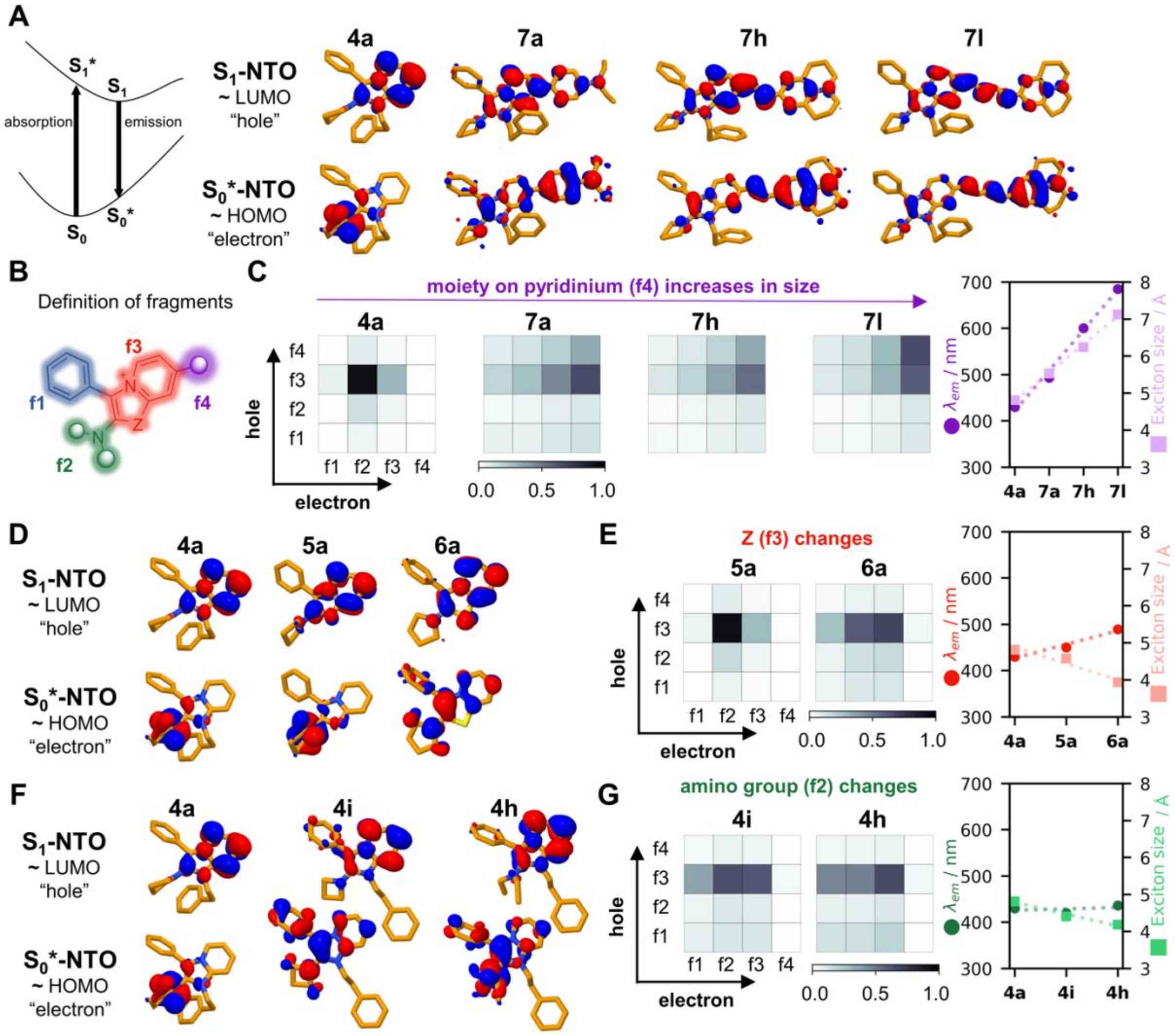

*Figure S12. A,D,F) Schematic energy diagram of absorption and emission and natural transition orbitals (NTOs) involved in emission of compounds **4a**, **7a**, **7h**, **7l** (A), **5a**, **6a** (D), and **4h**, **4i** (F). B) Definition of fragments (f1 to f4) employed to systematically investigate the influence of substitution on the maximum emission wavelength (nm). Also shown are the electron-hole-correlation plots obtained when fragments f4 (C), f3 (E), and f2 (G) are varied as well as calculated exciton sizes (Å) versus experimental emission wavelengths for selected compounds compared with reference **4a**.*

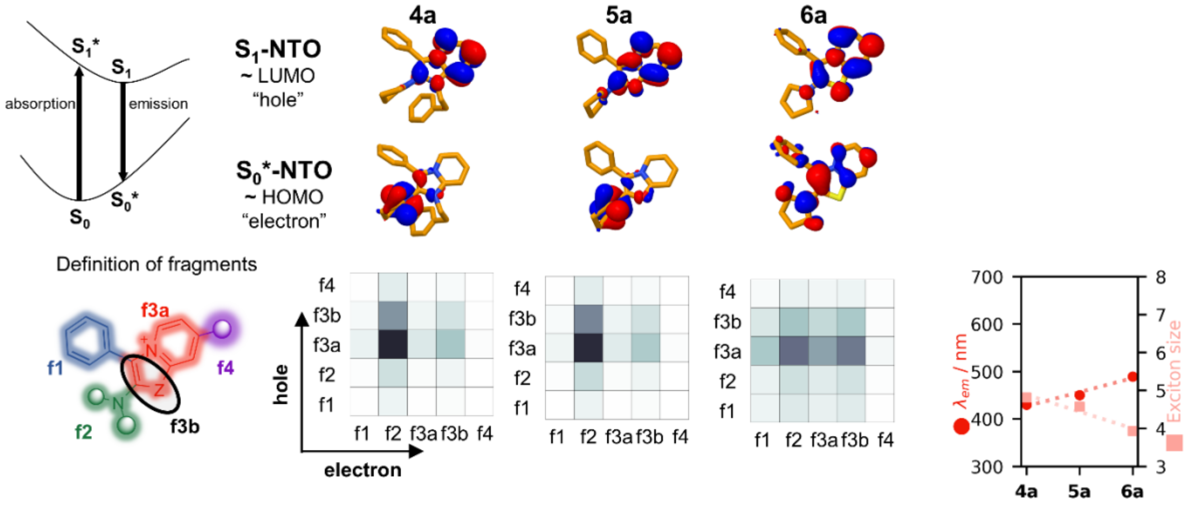

Figure S13. Extension of Figure S12, where the fragments (f1-f4) are further refined for compounds **4a**, **5a**, and **6a**. As before, the NTOs, the electron-hole-correlation plots, and the calculated exciton sizes (Å) versus experimental emission wavelengths are shown.

Table S7. Experimental and calculated (predicted) maximum emission energies and their associated absolute errors (in eV).

NAME	SMILES	Calc. E_{em} / eV	Exp. E_{em} / eV	absolute error / eV
4k	[N+]12=C(C=CC=C2)N(CC3CCCCC3)C(N4CCCC4)=C1C5=CC=CC=C5	415	427	-2.8
4j	CCCCCCCN1C(N2CCCC2)=C(C3=CC=CC=C3)[N+]4=C1C=CC=C4	422	424	-0.5
4l	C=CCCCCCCN1C(N2CCCC2)=C(C3=CC=CC=C3)[N+]4=C1C=CC=C4	423	427	-0.9
4o	FC(C=C1)=CC=C1CCN2C(N3CCCC3)=C(C4=CC=CC=C4)[N+]5=C2C=CC=C5	420	427	-1.6
4p	CC(C=C1)=CC=C1CCN2C(N3CCCC3)=C(C4=CC=CC=C4)[N+]5=C2C=CC=C5	420	429	-2.1
4g	[N+]12=C(C=CC=C2)N(CCC3=CC=CC=C3)C(N4CCCCC4)=C1C5=CC=CC=C5	429	422	1.7
4h	CCN(CC)C1=C(C2=CC=CC=C2)[N+]3=C(C=CC=C3)N1CCC4=CC=CC=C4	413	420	-1.7
4r	[N+]12=C(C=C(C3=CC=CC=C3)C=C2)N(CCC4=CC=CC=C4)C(N5CCCC5)=C1C6=CC=CC=C6	451	460	-2.0
4s	FC(F)(F)C(C=C1)=CC=C1C(C=C2)=CC3=[N+]2C(C4=CC=CC=C4)=C(N5CCCC5)N3CCC6=CC=CC=C6	486	474	2.5
4u	COC(C=C1)=CC=C1C(C=C2)=CC3=[N+]2C(C4=CC=CC=C4)=C(N5CCCC5)N3CCC6=CC=CC=C6	459	454	1.1
4q	[N+]12=C(C=C(C3=CC(C=CC=C4)=C4C5=C3C=CC=C5)C=C2)N(CCC6=CC=CC=C6)C(N7CCCC7)=C1C8=CC=CC=C8	464	464	0.0
4t	FC(C=C1)=CC=C1C(C=C2)=CC3=[N+]2C(C4=CC=CC=C4)=C(N5CCCC5)N3CCC6=CC=CC=C6	459	458	0.2
7a	CCN(CC)C(C=C1)=CC=C1C(C=C2)=CC3=[N+]2C(C4=CC=CC=C4)=C(N5CCCC5)N3CCC6=CC=CC=C6	515	493	4.5

7o	FC(F)(F)C(C=C1)=CC=C1/C=C/C=C/C=C/C(C=C2)=CC3=[N+]2C(C4=CC=CC=C4)=C(N5CCCC5)N3CCC6=CC=CC=C6	605	532	13.7
7m	CC(C=C1)=CC=C1/C=C/C=C/C(C=C2)=CC3=[N+]2C(C4=CC=CC=C4)=C(N5CCCC5)N3CCC6=CC=CC=C6	561	498	12.7
7g	CCN(CC)C(C=C1)=CC=C1/C=C/C(C=C2)=CC3=[N+]2C(C4=CC=CC=C4)=C(N5CCCC5)N3CCC6=CC=CC=C6	588	571	3.0
7i	[N+]12=C(C=C(/C=C/C3=CC=C(N4CCOCC4)C=C3)C=C2)N(CCC5=CC=CC=C5)C(N6CCCC6)=C1C7=CC=CC=C7	564	565	-0.2
7k	CCN(CC)C(C=C1)=CC=C1/C=C/C=C/C(C=C2)=CC3=[N+]2C(C4=CC=CC=C4)=C(N5CCCC5)N3CCC6=CC=CC=C6	639	644	-0.8
7n	[N+]12=C(C=C(/C=C/C=C/C3=CC=C(N4CCOCC4)C=C3)C=C2)N(CCC5=CC=CC=C5)C(N6CCCC6)=C1C7=CC=CC=C7	614	636	-3.5
8e	CN(C)C(C=C1)=CC=C1/C=C/C(C=C2)=CC3=[N+]2C(C4=CC=CC=C4)=C(N5CCCC5)O3	608	598	1.7
8g	[N+]12=C(C=C(/C=C/C3=CC=C(N4CCOCC4)C=C3)C=C2)OC(N5CCCC5)=C1C6=CC=CC=C6	596	596	0.0
8h	CCN(CC)C(C=C1)=CC=C1/C=C/C=C/C(C=C2)=CC3=[N+]2C(C4=CC=CC=C4)=C(N5CCCC5)O3	663	682	-2.8
5b	COC(C=C1)=CC=C1C2=C(N3CCCC3)OC4=[N+]2C=CC=C4	461	479	-3.8
5e	ClC(C=CC=C1)=C1C2=C(N3CCCC3)OC4=[N+]2C=CC=C4	438	427	2.6
4ab	[N+]12=C(C(C=CC=C3)=C3C=C2)N(CC4CCCCC4)C(N5CCCC5)=C1C6=CC=CC=C6	418	472	-11.4
4z	CCCCCCCN1C(N2CCCC2)=C(C3=CC=CC=C3)[N+]4=C1C=CC5=C4C=CC=C5	458	480	-4.6
8a	CCN(CC)C(C=C1)=CC=C1C(C=C2)=CC3=[N+]2C(C4=CC=CC=C4)=C(N5CCCC5)O3	534	508	5.1
8d	CCN(CC)C(C=C1)=CC=C1/C=C/C(C=C2)=CC3=[N+]2C(C4=CC=CC=C4)=C(N5CCCC5)O3	611	600	1.8
5a	[N+]12=C(C=CC=C2)OC(N3CCCC3)=C1C4=CC=CC=C4	444	450	-1.3
4i	[N+]12=C(C=CC=C2)N(CCC3=CC=CC=C3)C(N4CCC4)=C1C5=CC=CC=C5	437	436	0.2
8f	[N+]12=C(C=C(/C=C/C3=CC4=C(N(CCC4)CCC5)C5=C3)C=C2)OC(N6CCCC6)=C1C7=CC=CC=C7	636	628	1.3
7h	[N+]12=C(C=C(/C=C/C3=CC4=C(N(CCC4)CCC5)C5=C3)C=C2)N(CCC6=CC=CC=C6)C(N7CCCC7)=C1C8=CC=CC=C8	605	600	0.8
8i	[N+]12=C(C=C(/C=C/C=C/C3=CC4=C(N(CCC5)CCC4)C5=C3)C=C2)OC(N6CCCC6)=C1C7=CC=CC=C7	689	720	-4.3
7l	[N+]12=C(C=C(/C=C/C=C/C3=CC4=C(N(CCC5)CCC4)C5=C3)C=C2)N(CCC6=CC=CC=C6)C(N7CCCC7)=C1C8=CC=CC=C8	660	685	-3.6
4d	COC(C=C1)=CC=C1C2=C(N3CCCC3)N(CCC4=CC=CC=C4)C5=[N+]2C=CC=C5	432	448	-3.6
4e	ClC(C=CC=C1)=C1C2=C(N3CCCC3)N(CCC4=CC=CC=C4)C5=[N+]2C=CC=C5	422	412	2.4
4a	[N+]12=C(C=CC=C2)N(CCC3=CC=CC=C3)C(N4CCCC4)=C1C5=CC=CC=C5	423	430	-1.6

**8c**	CCN(CC)C(C=C1)=CC=C1C(C=C2)=CC=C2C(C=C3)=CC4=[N+]3C(C5=CC=CC=C5)=C(N6CCCC6)O4	593	680	-12.8
**7c**	CCN(CC)C(C=C1)=CC=C1C(C=C2)=CC=C2C(C=C3)=CC4=[N+]3C(C5=CC=CC=C5)=C(N6CCCC6)N4CCC7=CC=CC=C7	574	624	-8.0
**6a**	[N+]12=C(C=CC=C2)SC(N3CCCC3)=C1C4=CC=CC=C4	453	489	-7.4
**9a**	CCN(CC)C(C=C1)=CC=C1C(C=C2)=CC3=[N+]2C(C4=CC=CC=C4)=C(N5CCCC5)S3	544	522	4.2
**9b**	CCN(CC)C1=CC=C(/C=C/C(C=C2)=CC3=[N+]2C(C4=CC=CC=C4)=C(N5CCCC5)S3)C=C1	608	615	-1.1
**7b**	C1(C2=C(N3CCCC3)N(CCC4=CC=CC=C4)C5=[N+]2C=CC(C6=CC7=C8C(CCCN8CCC7)=C6)=C5)=CC=CC=C1	537	520	3.3
**8b**	C1(C2=C(N3CCCC3)OC4=[N+]2C=CC(C5=CC6=C7C(CCCN7CCC6)=C5)=C4)=CC=CC=C1	559	528	5.9

11.2. XYZ-coordinates of the optimized S0 and S1 minima of compounds 4a, 5a, 6a, 7a, 8a, 9a, 7g, 8d, and 9b

4a

S_0:

C 0.53806 2.31187 -
0.61958
C 2.17970 -0.90118
0.01377
C -2.66617 1.01869
0.94087
N -0.46541 1.48639 -
1.03944
C 0.63096 3.70780 -
0.57702
C 2.82521 -1.64282 -
0.99060
C -0.58138 -2.23179 -
0.50345
C -3.72156 0.10625
0.78495
N 1.57076 1.52089 -
0.17748
C 1.79930 4.26921 -
0.08406
C 3.71720 -2.66359 -
0.65056
C -1.75378 -3.08544 -
0.98440
C -3.76114 -1.07517
1.53174
N -0.84536 -0.91779 -
1.12220
C 2.85778 3.43898
0.34930
C 3.98341 -2.94286
0.69439
C -2.00895 -2.53106 -
2.39060
C -2.74231 -1.36300
2.44681
C 2.73310 2.07031
0.29440
C 3.35462 -2.20070
1.70074
C -1.86385 -1.02001 -
2.18361
C -1.69017 -0.45698
2.61755
C 2.45585 -1.18584
1.36322
C -1.65647 0.72542
1.87211
C 1.23158 0.17496 -
0.33781
C -0.05948 0.15401 -
0.86721
C -1.82427 2.00259 -
1.25125
C -2.57135 2.25300
0.07334
H -0.19811 4.32334 -
0.92223
H 2.61595 -1.41949 -
2.03921
H -0.52333 -2.12819
0.58940
H 0.37138 -2.65360 -
0.86079
H -4.51979 0.32234
0.06902
H 1.90030 5.35430 -
0.03416
H 4.20767 -3.24070 -
1.43802
H -2.63199 -2.91804 -
0.34113
H -1.51522 -4.15835 -
0.97244
H -4.58982 -1.77493
1.39733
H 3.78487 3.86760
0.73076
H 4.68170 -3.74062
0.95915
H -2.99482 -2.79604 -
2.79846
H -1.23877 -2.89526 -
3.09003
H -2.76941 -2.28941
3.02562
H 3.50485 1.36859
0.60675
H 3.55783 -2.41886
2.75185
H -1.54601 -0.48485 -
3.09144
H -2.82646 -0.60142 -
1.84512
H -0.89130 -0.67133
3.33188
H 1.94961 -0.61691
2.14690
H -0.83109 1.42926
2.01326
H -2.37200 1.29961 -
1.88041
H -1.74076 2.93390 -
1.82794
H -2.06360 3.06209
0.62155
H -3.57456 2.62367 -
0.18905

4a

S_1:

C 0.48131 2.30597 -
0.35824
C 2.32762 -0.89467 -
0.28895
C -2.83765 1.07590
1.04504
N -0.48723 1.44587 -
0.83860
C 0.44386 3.66290 -
0.11477
C 2.55817 -1.82759 -
1.31568
C -1.14789 -1.90166 -
0.22959
C -3.87858 0.13818
0.93908
N 1.62886 1.54271 -
0.15161
C 1.64936 4.29545
0.34474
C 3.39230 -2.92738 -
1.09757
C -2.03713 -2.87453 -
0.99921
C -3.96727 -0.93649
1.83046
N -0.65825 -0.98313 -
1.23455
C 2.80105 3.52176
0.52317
C 4.01572 -3.10021
0.14203
C -1.39403 -2.89576 -
2.39730
C -3.01055 -1.09178
2.84107
C 2.82712 2.15738
0.27953
C 3.79908 -2.17055
1.16638
C -0.92432 -1.45108 -
2.58385
C -1.96809 -0.16407
2.95615
C 2.95818 -1.07634
0.95715
C -1.88424 0.91098
2.06493
C 1.38655 0.21962 -
0.48814
C 0.08831 0.17370 -
0.92271
C -1.84609 1.85035 -
1.17130
C -2.70794 2.20870
0.05609
H -0.46957 4.22586 -
0.29221
H 2.09988 -1.68261 -
2.29556
H -1.60366 -1.34387
0.60003
H -0.24462 -2.38869
0.19597
H -4.62740 0.25177
0.15028

H 1.66195 5.36773 0.53578
H 3.56244 -3.64468 -1.90370
H -3.06147 -2.47241 -1.04148
H -2.07364 -3.86196 -0.52298
H -4.78379 -1.65625 1.73421
H 3.73011 3.99013 0.85606
H 4.67155 -3.95759 0.31070
H -2.08398 -3.20784 -3.19071
H -0.52075 -3.56605 -2.41203
H -3.07686 -1.93262 3.53555
H 3.69954 1.51740 0.35972
H 4.28035 -2.30371 2.13810
H -0.04010 -1.28297 -3.21563
H -1.72309 -0.80151 -2.99584
H -1.21691 -0.27855 3.74141
H 2.77507 -0.36621 1.76629
H -1.06976 1.63437 2.16109
H -2.31820 1.03197 -1.72978
H -1.78806 2.71533 -1.85230
H -2.27726 3.08995 0.55420
H -3.69699 2.50766 -0.32396

5a S_0:

C 5.14089 -2.97897 0.59448
C 6.11580 -1.79246 -2.73136
O 4.59991 -1.75531 0.64834
C 5.15941 -3.94917 1.58183
C 7.03780 -0.75602 -2.96850
C 3.62171 0.79655 0.39744
N 5.71773 -3.15511 -0.62964
C 5.80516 -5.14642 1.27719
C 7.57048 -0.56367 -4.24557
C 3.36497 2.18120 -0.20521
N 4.31522 0.08906 -0.69630
C 6.40948 -5.32180 0.01709
C 7.20749 -1.41604 -5.29359
C 3.19165 1.88887 -1.70203
C 6.36328 -4.31959 -0.93173
C 6.30342 -2.45974 -5.06334
C 4.25213 0.81654 -1.97440
C 5.75452 -2.64479 -3.79280
C 5.54545 -1.98080 -1.38853
C 4.82916 -1.13019 -0.55688
H 4.68229 -3.75400 2.54180
H 7.33993 -0.10651 -2.14427
H 4.24579 0.81824 1.30294
H 2.68122 0.27423 0.64397
H 5.84336 -5.94573 2.01857
H 8.28013 0.24861 -4.41920
H 4.24114 2.82961 -0.04292
H 2.49158 2.67083 0.24740
H 6.92468 -6.25035 -0.23012
H 7.62995 -1.26851 -6.29038
H 3.32857 2.77408 -2.33858
H 2.18675 1.48045 -1.89657
H 6.81234 -4.38532 -1.92131
H 6.01461 -3.12533 -5.88012
H 3.98824 0.13321 -2.79466
H 5.23233 1.26456 -2.20899
H 5.02817 -3.44368 -3.62445

5a S_1:

C 5.18055 -3.04361 0.67242
C 6.33210 -1.80059 -2.59409
O 4.00153 -2.56350 0.18243
C 5.40713 -3.64323 1.87440
C 7.50356 -1.03009 -2.46811
C 3.10152 0.08621 -1.39468
N 6.17030 -2.84515 -0.28955
C 6.73702 -4.10498 2.14375
C 8.16410 -0.57079 -3.60849
C 2.01738 0.54972 -2.36855
N 3.27271 -1.32312 -1.68859
C 7.72319 -3.94109 1.15861
C 7.66373 -0.86948 -4.88155
C 1.18787 -0.72572 -2.60471
C 7.47056 -3.33022 -0.05547
C 6.50109 -1.63558 -5.01261
C 2.23633 -1.83964 -2.55556
C 5.83901 -2.10463 -3.87543
C 5.60285 -2.24193 -1.39738
C 4.27514 -2.11644 -1.09647
H 4.58427 -3.76268 2.57774
H 7.88626 -0.77722 -1.47707
H 4.07955 0.58970 -1.45187
H 2.78590 0.14108 -0.33095
H 6.96535 -4.59128 3.09051
H 9.07038 0.02980 -3.50257
H 2.48835 0.88840 -3.30421
H 1.42681 1.37916 -1.96050
H 8.73655 -4.30862 1.33294
H 8.18321 -0.50692 -5.77166
H 0.64308 -0.71797 -3.55658
H 0.46136 -0.87479 -1.79126
H 8.19167 -3.19792 -0.85614

H 6.11210 -1.87948 -6.00375
H 1.91111 -2.82669 -2.19179
H 2.71445 -2.01658 -3.54214
H 4.95228 -2.73267 -3.98112

6a S_0:

C 5.10516 -3.19050 0.81420
C 6.01619 -1.78595 -2.50856
S 4.28900 -1.70727 1.18457
C 5.25633 -4.30250 1.64302
C 7.15191 -0.97434 -2.66988
C 3.56793 1.05293 0.22683
N 5.63485 -3.16365 -0.45392
C 5.95680 -5.40286 1.16650
C 7.70900 -0.78555 -3.93798
C 3.42486 2.37339 -0.53450
N 4.25749 0.17580 -0.73408
C 6.50021 -5.36203 -0.13162
C 7.14499 -1.41592 -5.05183
C 3.29711 1.91897 -1.99377
C 6.33551 -4.24649 -0.92178
C 6.02112 -2.23639 -4.89759
C 4.29229 0.75879 -2.08884
C 5.45706 -2.41976 -3.63319
C 5.42276 -1.96904 -1.16290
C 4.69365 -1.05255 -0.41369
H 4.82356 -4.28529 2.64384
H 7.59086 -0.48636 -1.79681
H 4.15329 1.15407 1.15536
H 2.58119 0.62587 0.48741
H 6.08516 -6.28591 1.79387
H 8.58673 -0.14573 -4.05520
H 4.33374 2.98208 -0.40208
H 2.56666 2.96174 -0.18161
H 7.06074 -6.20647 -0.53390
H 7.58129 -1.26818 -6.04270
H 3.52057 2.71357 -2.71929
H 2.27586 1.55455 -2.19078
H 6.73651 -4.15789 -1.92941
H 5.57715 -2.72777 -5.76648
H 4.01350 0.00659 -2.83912
H 5.31004 1.11043 -2.32603
H 4.57059 -3.04726 -3.51373

6a S_1:

C 4.81343 -3.40059 0.70042
C 6.19092 -1.64600 -2.28590
S 3.97318 -1.90889 1.10391
C 4.73457 -4.60428 1.34095
C 6.98798 -0.50373 -2.38652
C 4.63501 1.35686 -0.27879
N 5.65261 -3.21615 -0.41523
C 5.54520 -5.67453 0.92473
C 7.53544 -0.13797 -3.61098
C 4.07354 2.35845 -1.28455
N 4.21276 0.06937 -0.78852
C 6.44479 -5.45306 -0.13421
C 7.29502 -0.91185 -4.74033
C 2.79373 1.66766 -1.78375
C 6.51557 -4.25878 -0.78584
C 6.50701 -2.05669 -4.64410
C 3.17477 0.18888 -1.79533
C 5.95654 -2.42417 -3.42470
C 5.53835 -1.98774 -1.01054
C 4.69723 -1.16353 -0.32170
H 4.05941 -4.70685 2.17898
H 7.19614 0.08591 -1.50408
H 5.71293 1.35940 -0.12495
H 4.17323 1.46953 0.71391
H 5.50009 -6.62660 1.42898
H 8.15338 0.74767 -3.67860
H 4.78496 2.49001 -2.10076
H 3.88530 3.32686 -0.82942
H 7.12059 -6.23613 -0.44991
H 7.72101 -0.62692 -5.69350
H 2.48222 2.01186 -2.76631
H 1.97270 1.82899 -1.08447
H 7.20579 -4.04060 -1.58176
H 6.31477 -2.65939 -5.52204
H 2.37821 -0.52465 -1.58099
H 3.63087 -0.12365 -2.74538
H 5.33243 -3.30553 -3.35441

7a S_0:

C 0.87832 0.10162 -0.77010
C 3.77932 -1.93118 0.19319
C 0.94663 2.72277 1.03389

N	1.87739	0.96475	-1.12618
C	-0.50830	0.21627	-0.85609
C	3.72970	-2.42220	1.51151
C	5.42485	0.81394	0.00773
C	-0.40238	3.10620	1.11204
N	1.46847	-1.01560	-0.23234
C	-1.32106	-0.81574	-0.37413
C	4.60538	-3.42916	1.92410
C	6.46688	1.82971	-0.46245
C	-1.25665	2.52956	2.05610
N	4.28503	1.07167	-0.89095
C	-0.66485	-1.97990	0.13424
C	5.54754	-3.95059	1.02999
C	6.22103	1.87278	-1.97578
C	-0.77473	1.55438	2.93710
C	0.70034	-2.06653	0.19371
C	5.60764	-3.46515	-0.28099
C	4.69118	1.86776	-2.06687
C	0.57022	1.17539	2.87943
C	4.72437	-2.46730	-0.70046
C	1.42386	1.76043	1.93824
C	2.86444	-0.86605	-0.24844
C	3.11141	0.39128	-0.78311
C	1.63895	2.39093	-1.36754
C	1.82814	3.22777	-0.08597
H	-0.93661	1.11433	-1.29351
H	3.01274	-1.99888	2.21931
H	5.81342	-0.21052	-0.11065
H	5.11859	0.93845	1.05807
H	-0.79124	3.85327	0.41376
C	-2.78607	-0.70029	-0.37085
N	-7.02603	-0.34246	-0.34253
C	-3.42638	0.55723	-0.32693
C	-4.80762	0.68326	-0.31112
C	-5.65864	-0.45815	-0.35233
C	-5.01109	-1.72544	-0.40052
C	-3.62740	-1.83272	-0.40063
C	-7.89933	-1.51124	-0.26129
C	-7.69122	0.95680	-0.41141
C	-7.90495	1.60698	0.95739
C	-8.23882	-2.11565	-1.62561
H	-7.44316	-2.26813	0.39499
H	-8.66258	0.80617	-0.90809
H	-8.91583	-2.97681	-1.50912
H	-8.82427	-1.19947	0.24879
H	-7.12358	1.62748	-1.07447
H	-8.51219	0.95745	1.60781
H	-8.42903	2.56997	0.85013
H	-2.82897	1.46846	-0.25593
H	-5.23002	1.68411	-0.23942
H	-6.94535	1.79164	1.46470
H	-5.59604	-2.64181	-0.45901
H	-3.19447	-2.83359	-0.46296
H	-7.33116	-2.45889	-2.14577
H	-8.73722	-1.37286	-2.26867
H	4.55966	-3.79987	2.95085
H	7.48930	1.52900	-0.19301
H	6.27015	2.81791	-0.01487
H	-2.30508	2.83496	2.09882
H	-1.24232	-2.81866	0.52080
H	6.23595	-4.73412	1.35581
H	6.63249	0.96750	-2.45136
H	6.66213	2.74991	-2.47041
H	-1.44518	1.09446	3.66720
H	1.23457	-2.93098	0.58349
H	6.34124	-3.86945	-0.98252
H	4.31098	2.90163	-1.99936
H	4.30907	1.42004	-2.99732
H	0.95774	0.41852	3.56614
H	4.76303	-2.09288	-1.72592
H	2.47181	1.45121	1.89403
H	0.61487	2.49378	-1.74639
H	2.29953	2.73707	-2.16919
H	2.88803	3.19164	0.20882
H	1.59065	4.27308	-0.33785

7a S_1:

C	0.86374	0.03974	-0.82388
C	3.79158	-1.95063	0.16292
C	0.94877	2.67166	1.01976
N	1.87916	0.91380	-1.17807
C	-0.50252	0.17049	-0.89827
C	3.62417	-2.59519	1.40914
C	5.37777	0.87701	0.06544
C	-0.39324	3.07640	1.10791
N	1.46894	-1.08431	-0.29213
C	-1.33620	-0.90730	-0.42190
C	4.53378	-3.56494	1.83175
C	6.38966	1.93389	-0.38485
C	-1.24815	2.51854	2.06207
N	4.28730	1.01641	-0.91791
C	-0.67883	-2.07907	0.03299
C	5.62830	-3.90670	1.02742
C	6.21365	1.93440	-1.90820
C	-0.77328	1.54175	2.94534
C	0.69053	-2.18878	0.08988
C	5.80650	-3.27107	-0.20849
C	4.69238	1.85286	-2.06194

C 0.56456 1.14024 2.87653
C 4.89642 -2.30686 -0.64105
C 1.41825 1.70568 1.92356
C 2.85555 -0.92413 -0.28982
C 3.10739 0.34472 -0.82352
C 1.63253 2.33991 -1.38980
C 1.82986 3.16864 -0.10366
H -0.92992 1.07043 -1.33007
H 2.79213 -2.31211 2.05732
H 5.83146 -0.12605 0.02734
H 4.99037 1.03157 1.08457
H -0.77619 3.82592 0.40892
C -2.78979 -0.76177 -0.40484
N -7.02242 -0.30157 -0.31146
C -3.40895 0.52012 -0.42641
C -4.78445 0.67836 -0.39576
C -5.65961 -0.44666 -0.33938
C -5.04370 -1.73718 -0.32006
C -3.66899 -1.87897 -0.35657
C -7.92260 -1.44169 -0.14424
C -7.66498 1.00442 -0.44872
C -7.83914 1.73164 0.88843
C -8.30578 -2.10431 -1.47126
H -7.46734 -2.17227 0.53967
H -8.64813 0.83801 -0.91458
H -9.00269 -2.93663 -1.28740
H -8.82636 -1.07262 0.36419
H -7.09391 1.62180 -1.15711
H -8.44601 1.13254 1.58484
H -8.34854 2.69446 0.72803
H -2.79008 1.41821 -0.42337
H -5.18590 1.69032 -0.39292
H -6.86576 1.92781 1.36296
H -5.65383 -2.63857 -0.30186
H -3.26037 -2.89067 -0.37075
H -7.41815 -2.50102 -1.98694
H -8.79899 -1.38366 -2.14173
H 4.39447 -4.04903 2.80145
H 7.41156 1.68959 -0.06180
H 6.12530 2.92073 0.02933
H -2.29160 2.83998 2.11130
H -1.25558 -2.93734 0.37954
H 6.34068 -4.66413 1.36285
H 6.68823 1.04182 -2.34754
H 6.63162 2.82403 -2.40043
H -1.44365 1.09762 3.68539
H 1.22447 -3.07587 0.41509
H 6.65491 -3.53667 -0.84386
H 4.26071 2.86674 -1.99403
H 4.36036 1.40068 -3.00918
H 0.94627 0.38084 3.56379
H 5.02609 -1.82740 -1.61376
H 2.46033 1.37829 1.87146
H 0.60349 2.44308 -1.75467
H 2.27919 2.70662 -2.19499
H 2.89012 3.12730 0.19020
H 1.60030 4.21730 -0.35046

8a S_0:

C 5.14740 -3.26756 0.43205
C 6.08984 -1.82037 -2.80058
O 4.64379 -2.03498 0.60313
C 5.16570 -4.30186 1.34178
C 6.99040 -0.75252 -2.97610
C 3.72765 0.54766 0.59196
N 5.68924 -3.35334 -0.81572
C 5.77031 -5.52114 0.96665
C 7.50535 -0.46205 -4.24182
C 3.44404 1.97070 0.10136
N 4.37311 -0.07729 -0.57740
C 6.33641 -5.57887 -0.34151
C 7.14879 -1.24740 -5.34314
C 3.20367 1.78374 -1.40311
C 6.29554 -4.51197 -1.20764
C 6.26805 -2.32260 -5.17584
C 4.25103 0.73613 -1.79675
C 5.73574 -2.60460 -3.91620
C 5.53691 -2.10979 -1.47006
C 4.86473 -1.31708 -0.55387
H 4.73388 -4.12955 2.32580
H 7.29210 -0.15725 -2.11172
H 4.38944 0.50810 1.46988
H 2.79820 0.00730 0.84263
C 5.81581 -6.66887 1.87781
N 5.98204 -9.97161 4.56795
C 4.93444 -6.78532 2.97473
C 4.96872 -7.86597 3.84329
C 5.91869 -8.91762 3.68827
C 6.79632 -8.80635 2.57029
C 6.74114 -7.72390 1.70758
C 6.82674 -11.12814 4.26939
C 4.95844 -10.06981 5.62044
C 6.20218 -12.10396 3.26896
C 5.15470 -11.19286 6.63165
H 5.99263 -11.60801 2.30842
H 4.94427 -9.11648 6.17340
H 7.05125 -11.64747 5.20820
H 7.80149 -10.77365 3.90457

H 4.35624 -11.11991 7.38556
H 3.95842 -10.17291 5.15622
H 5.25343 -12.50858 3.65564
H 4.16779 -6.02660 3.14483
H 4.23768 -7.89167 4.64809
H 6.88290 -12.94879 3.07840
H 7.53967 -9.57575 2.37195
H 7.46305 -7.69490 0.88931
H 6.11809 -11.10971 7.15791
H 5.08712 -12.19238 6.17693
H 8.19794 0.37391 -4.36515
H 4.32657 2.60907 0.26931
H 2.59193 2.42468 0.62614
H 6.80565 -6.49263 -0.70256
H 7.55793 -1.02361 -6.33126
H 3.31093 2.71217 -1.98123
H 2.19102 1.38648 -1.58016
H 6.71832 -4.52799 -2.21065
H 5.98353 -2.93703 -6.03331
H 3.94964 0.10991 -2.64955
H 5.21809 1.20403 -2.04772
H 5.02554 -3.42656 -3.79831

8a S_1:

C 5.21983 -3.26742 0.42983
C 6.18919 -1.75979 -2.76921
O 4.67811 -2.03138 0.59210
C 5.21606 -4.29348 1.32381
C 6.91047 -0.56338 -2.98597
C 3.63391 0.47162 0.55937
N 5.81216 -3.31846 -0.81023
C 5.89011 -5.52371 0.95718
C 7.40570 -0.25534 -4.25194
C 3.20488 1.85256 0.05651
N 4.35927 -0.09381 -0.59406
C 6.54770 -5.52672 -0.30932
C 7.21054 -1.13859 -5.32193
C 3.01967 1.63455 -1.45089
C 6.52930 -4.46181 -1.17509
C 6.51313 -2.33638 -5.11573
C 4.18853 0.71209 -1.81181
C 6.00472 -2.64774 -3.85580
C 5.65915 -2.07400 -1.44950
C 4.92356 -1.30300 -0.54645
H 4.72798 -4.13664 2.28213
H 7.10325 0.10753 -2.14701
H 4.28376 0.49991 1.44705
H 2.76682 -0.16885 0.79838
C 5.90479 -6.67038 1.85473
N 5.97087 -10.01146 4.52177
C 5.21758 -6.66285 3.10255
C 5.22725 -7.74547 3.96748
C 5.94035 -8.94159 3.65891
C 6.62045 -8.96184 2.40094
C 6.60075 -7.87531 1.54776
C 6.61704 -11.26243 4.13089
C 5.16476 -9.95289 5.75030
C 5.73233 -12.15653 3.25390
C 5.29762 -11.13774 6.69782
H 5.44793 -11.64156 2.32357
H 5.44338 -9.03696 6.29898
H 6.90869 -11.80153 5.03903
H 7.56018 -11.03167 3.61550
H 4.65259 -10.94798 7.56887
H 4.10056 -9.83062 5.47100
H 4.80968 -12.43901 3.78376
H 4.64758 -5.78583 3.40904
H 4.67074 -7.65510 4.89782
H 6.27443 -13.07783 2.98897
H 7.17156 -9.84523 2.08493
H 7.14602 -7.96898 0.60823
H 6.32644 -11.26023 7.06853
H 4.96886 -12.08429 6.24417
H 7.96260 0.67261 -4.40199
H 4.00577 2.58665 0.24052
H 2.29570 2.20746 0.56114
H 7.11122 -6.39986 -0.63620
H 7.60393 -0.89655 -6.31197
H 3.03758 2.56640 -2.03301
H 2.06335 1.12436 -1.64962
H 7.04499 -4.43604 -2.13064
H 6.35339 -3.02703 -5.94718
H 3.99686 0.06020 -2.67660
H 5.10381 1.29068 -2.02188
H 5.43223 -3.56672 -3.71423

9a S_0:

C 4.97429 -3.21205 0.82458
C 5.93789 -1.87225 -2.49765
S 4.29108 -1.67266 1.20531

C 5.05703 -4.31425
1.65103
C 7.07482 -1.08226 -
2.68281
C 3.77851 1.13467
0.26550
N 5.47828 -3.22213 -
0.44713
C 5.65949 -5.49178
1.20090
C 7.62685 -0.93132 -
3.94989
C 3.63249 2.43373 -
0.52435
N 4.36301 0.20417 -
0.71167
C 6.17894 -5.46429 -
0.11622
C 7.05620 -1.57900 -
5.04027
C 3.37739 1.94341 -
1.95293
C 6.08779 -4.35593 -
0.90451
C 5.92980 -2.37816 -
4.86211
C 4.31052 0.74029 -
2.08361
C 5.37159 -2.52229 -
3.59880
C 5.35000 -2.01157 -
1.15092
C 4.71247 -1.05293 -
0.39587
H 4.66640 -4.22925
2.65403
H 7.51898 -0.58244 -
1.83173
H 4.42758 1.23883
1.13710
H 2.80263 0.76481
0.60203
C 5.75310 -6.68302
2.04226
N 6.07492 -10.10038
4.53751
C 4.84920 -6.92393
3.08736
C 4.93126 -8.04548
3.88788
C 5.95569 -9.00836
3.71183
C 6.85306 -8.77278
2.64088
C 6.74941 -7.65285
1.84385
C 6.94064 -11.21427
4.14974
C 5.02145 -10.34183
5.53482
C 6.32481 -12.12389
3.08682
C 5.31864 -11.44655
6.53856
H 6.10044 -11.56583
2.17652
H 4.87714 -9.41715
6.09517
H 7.17464 -11.78998
5.04055
H 7.89534 -10.81814
3.80753
H 4.51393 -11.46056
7.27490
H 4.07076 -10.55758
5.02986
H 5.39664 -12.56971
3.44928
H 4.02915 -6.23931
3.26222
H 4.17816 -8.17663
4.64784
H 7.01437 -12.93126
2.83317
H 7.64844 -9.46923
2.42828
H 7.48618 -7.52183
1.06217
H 6.25501 -11.26707
7.06936
H 5.35992 -12.43454
6.07985
H 8.50311 -0.31031 -
4.08357
H 4.56336 3.00248 -
0.48016
H 2.83174 3.05905 -
0.13381
H 6.64609 -6.33821 -
0.54370
H 7.48670 -1.46221 -
6.02649
H 3.58126 2.69983 -
2.70896
H 2.33906 1.62309 -
2.06088
H 6.47321 -4.31654 -
1.90973
H 5.48052 -2.88072 -
5.70883
H 3.94073 -0.01744 -
2.77257
H 5.30817 1.03970 -
2.41254
H 4.48627 -3.13071 -
3.46308

9a S_1:

C 5.19521 -3.15019
0.89586
C 6.01683 -1.82775 -
2.47928
S 4.56846 -1.57073
1.30628
C 5.23548 -4.25212
1.69361
C 6.88612 -0.78381 -
2.82342
C 3.95336 1.19223
0.27596
N 5.71694 -3.15229 -
0.39269
C 5.87462 -5.44833
1.24856
C 7.33424 -0.64379 -
4.12925
C 3.52975 2.38818 -
0.57424
N 4.44481 0.21702 -
0.70927
C 6.52477 -5.36465 -
0.01773
C 6.92905 -1.54538 -
5.11004
C 3.09438 1.73810 -
1.89070
C 6.45823 -4.26119 -
0.80336
C 6.07245 -2.59260 -
4.77621
C 4.13099 0.63356 -
2.08646
C 5.62028 -2.73676 -
3.47351
C 5.53667 -1.96375 -
1.10375
C 4.89553 -0.99666 -
0.34424
H 4.82167 -4.17374
2.68802
H 7.21563 -0.09249 -
2.05902
H 4.73184 1.43034
1.00271
H 3.09699 0.76982
0.81621
C 5.89004 -6.64799
2.05916
N 5.94912 -10.14400
4.47470
C 4.99797 -6.83142
3.14423
C 5.00486 -7.96565
3.92403
C 5.92233 -9.02385
3.68781
C 6.80896 -8.85250
2.58906
C 6.79005 -7.71538
1.81800
C 6.80455 -11.27588
4.12525
C 4.92319 -10.30512
5.51734
C 6.19367 -12.17980
3.05123
C 5.09280 -11.50571
6.43453
H 6.00787 -11.62334
2.13190

H 4.92759 -9.40344 6.13360
H 6.99699 -11.84957 5.02643
H 7.77426 -10.90006 3.80312
H 4.28767 -11.47674 7.16964
H 3.93794 -10.35419 5.03721
H 5.24758 -12.60206 3.39253
H 4.25623 -6.07658 3.36523
H 4.28190 -8.03425 4.72086
H 6.87636 -13.00126 2.82737
H 7.52309 -9.62014 2.33723
H 7.50831 -7.64618 1.01318
H 6.03827 -11.47484 6.97700
H 5.01960 -12.45490 5.90422
H 8.00766 0.16553 -4.38005
H 4.38242 3.04889 -0.74147
H 2.74024 2.96467 -0.09594
H 7.09547 -6.19693 -0.40132
H 7.27966 -1.43448 -6.12787
H 3.08077 2.43314 -2.72863
H 2.09830 1.30297 -1.78792
H 6.96020 -4.17599 -1.75119
H 5.75002 -3.29345 -5.53535
H 3.77043 -0.21918 -2.66014
H 5.02536 1.01878 -2.58200
H 4.94047 -3.54088 -3.22316

7g S_0:

C 1.67903 -0.04138 -0.75876
C 4.81107 1.78069 -0.14423
C 1.24414 -2.27234 1.46366
N 2.56984 -1.06926 -0.88982
C 0.29166 -0.01390 -0.86428
C 4.79635 2.60073 0.99986
C 5.32251 -2.35654 -1.63397
C 1.73648 -1.20197 2.22683
N 2.39684 1.08868 -0.43847
C -0.39516 1.18282 -0.61865
C 5.80577 3.54415 1.20595
C 6.84062 -2.47738 -1.46505
C 0.86610 -0.37247 2.94215
N 4.93976 -1.41582 -0.56101
C 0.39361 2.35006 -0.36372
C 6.84776 3.67269 0.28069
C 7.02104 -2.32246 0.05046
C -0.51193 -0.60786 2.91001
C 1.75820 2.29130 -0.27924
C 6.87536 2.85578 -0.85527
C 6.04713 -1.19035 0.38606
C -1.01303 -1.68491 2.16947
C 5.86066 1.92074 -1.07139
C -0.14068 -2.50902 1.45327
C 3.76381 0.77355 -0.37193
C 3.85961 -0.58642 -0.62586
C 2.17596 -2.47988 -0.87133
C 2.16531 -3.05778 0.55852
H -0.23611 -0.93223 -1.10753
H 4.00148 2.48467 1.74059
H 5.01995 -1.97166 -2.62006
H 4.84956 -3.34404 -1.49060
H 2.81185 -1.00269 2.24511
C -2.69883 0.22538 -0.53401
C -4.14694 0.25366 -0.47119
N -8.39449 0.16778 -0.24377
C -1.84434 1.28309 -0.58941
C -4.86205 -0.95667 -0.32353
C -6.24649 -0.99876 -0.24908
C -7.02496 0.19170 -0.31728
C -6.30363 1.41450 -0.46747
C -4.92123 1.43608 -0.54239
C -9.20147 1.37281 -0.42624
C -9.13145 -1.06620 0.02124
C -9.42237 2.15989 0.86726
C -9.45737 -1.86506 -1.24256
H -8.46646 2.50519 1.29063
H -2.26831 -0.78106 -0.49986
H -10.06341 -0.79076 0.53926
H -2.24238 2.30134 -0.55992
H -10.17155 1.06117 -0.84402
H -8.74281 2.01235 -1.19558
H -8.56944 -1.68530 0.73697
H -4.30595 -1.89746 -0.27010
H -6.72952 -1.96959 -0.15127
H -10.03046 -2.77113 -0.98946
H -6.83577 2.36327 -0.51404
H -4.42813 2.40424 -0.65675
H -9.92224 1.53483 1.62426
H -10.05498 3.04191 0.67938
H -8.53913 -2.17305 -1.76614
H -10.05999 -1.26255 -1.94073
H 5.78463 4.17318 2.09903
H 7.34620 -1.65168 -1.99228
H 7.22949 -3.42641 -1.86123
H 1.26663 0.46395 3.52052

H	-0.09361	3.31425	-0.21210
H	7.63935	4.40744	0.44635
H	8.05228	-2.08261	0.34643
H	6.72391	-3.25055	0.56609
H	-1.19448	0.04479	3.45975
H	2.39046	3.15468	-0.08102
H	7.68716	2.95151	-1.58025
H	5.67510	-1.21850	1.42232
H	6.53052	-0.21288	0.22644
H	-2.08873	-1.87612	2.14087
H	5.87599	1.28757	-1.96138
H	-0.54095	-3.33730	0.86101
H	2.85357	-3.04644	-1.51854
H	1.17978	-2.55168	-1.32569
H	1.84677	-4.10950	0.48464
H	3.19448	-3.05044	0.94890

7g S_1:

C	2.08678	0.00078	-0.37389
C	5.54508	1.01886	0.34991
C	1.49428	-2.37753	1.69114
N	2.75014	-1.17236	-0.68795
C	0.74862	0.31638	-0.40679
C	5.67732	1.71805	1.56871
C	5.05065	-3.02642	-1.58748
C	2.33701	-1.59720	2.49794
N	3.04543	0.90247	0.05508
C	0.32541	1.61904	0.03202
C	6.86439	2.38387	1.87749
C	6.48003	-3.57023	-1.50503
C	1.80745	-0.71386	3.44474
N	5.01199	-2.02258	-0.50784
C	1.35631	2.54546	0.38923
C	7.94102	2.35949	0.98241
C	6.77469	-3.49750	-0.00186
C	0.42225	-0.60485	3.60364
C	2.68004	2.20418	0.39082
C	7.82214	1.66543	-0.22794
C	6.17972	-2.14157	0.38476
C	-0.42808	-1.39017	2.81615
C	6.63428	1.00564	-0.54617
C	0.10543	-2.26738	1.86837
C	4.30935	0.30793	-0.01660
C	4.11441	-1.00391	-0.42370
C	2.05118	-2.45562	-0.77083
C	2.06335	-3.21744	0.57019
H	0.03141	-0.42841	-0.73963
H	4.85387	1.71532	2.28642
H	4.80423	-2.56554	-2.55645
H	4.33312	-3.84464	-1.40267
H	3.42091	-1.66963	2.37416
C	-2.14235	1.17617	-0.10062
C	-3.52653	1.55880	0.02038
N	-7.65711	2.52999	0.35252
C	-1.04843	1.99356	0.13361
C	-4.55097	0.60597	-0.24584
C	-5.89460	0.91083	-0.14118
C	-6.32952	2.21747	0.24489
C	-5.30632	3.18061	0.51199
C	-3.96671	2.86050	0.40313
C	-8.11378	3.88870	0.64343
C	-8.70786	1.52642	0.18421
C	-8.20078	4.18584	2.14326
C	-9.15171	1.35862	-1.27185
H	-7.21457	4.09480	2.62337
H	-1.96968	0.13550	-0.38884
H	-9.56263	1.84124	0.80209
H	-1.22634	3.02752	0.44431
H	-9.10515	4.00670	0.18013
H	-7.45755	4.61300	0.13942
H	-8.37099	0.56699	0.60266
H	-4.26400	-0.40455	-0.54928
H	-6.62142	0.13441	-0.37378
H	-9.95740	0.61086	-1.33673
H	-5.57059	4.18996	0.82322
H	-3.23404	3.63788	0.62709
H	-8.89056	3.48747	2.64225
H	-8.57205	5.20996	2.30388
H	-8.31586	1.02452	-1.90518
H	-9.53095	2.30918	-1.67824
H	6.95402	2.91511	2.82813
H	7.16916	-2.91307	-2.06026
H	6.56099	-4.58398	-1.92243
H	2.48028	-0.10730	4.05640
H	1.08875	3.56410	0.67800
H	8.87042	2.87872	1.22816
H	7.84544	-3.56149	0.23841
H	6.25441	-4.31170	0.52901
H	0.00577	0.08827	4.33882
H	3.48751	2.88697	0.64122
H	8.65672	1.64603	-0.93298
H	5.86277	-2.07329	1.43710
H	6.91233	-1.33930	0.20288
H	-1.51182	-1.31134	2.93433
H	6.53684	0.47981	-1.49852
H	-0.56571	-2.86434	1.24378

H	2.49131	-3.06150	-1.57077
H	1.01997	-2.24625	-1.07963
H	1.47717	-4.13947	0.42944
H	3.09624	-3.51897	0.80233

8d S_0:

C	5.00922	-3.35998	0.19974
C	6.15705	-1.66298	-2.84130
O	4.49945	-2.13971	0.43734
C	4.95454	-4.46465	1.01682
C	7.06956	-0.59151	-2.87813
C	3.59588	0.44313	0.57840
N	5.63009	-3.34823	-1.01674
C	5.57389	-5.65740	0.57405
C	7.65951	-0.20583	-4.08432
C	3.34619	1.90283	0.18680
N	4.31201	-0.09081	-0.59481
C	6.23039	-5.61149	-0.69306
C	7.36642	-0.89871	-5.26364
C	3.20043	1.83841	-1.33984
C	6.25531	-4.47587	-1.46674
C	6.47403	-1.97698	-5.23445
C	4.26889	0.81875	-1.74982
C	5.86731	-2.35370	-4.03470
C	5.52456	-2.05367	-1.57359
C	4.79732	-1.33259	-0.64058
H	4.43903	-4.37714	1.97173
H	7.32155	-0.06884	-1.95305
H	4.20158	0.32907	1.48969
H	2.65157	-0.10918	0.72673
C	4.97341	-7.09722	2.53187
C	4.95522	-8.31467	3.31395
N	4.82675	-11.81119	5.73789
C	5.57748	-6.89407	1.32661
C	4.25295	-8.34761	4.54120
C	4.19131	-9.48384	5.33261
C	4.84999	-10.68794	4.94959
C	5.55214	-10.65768	3.70585
C	5.60155	-9.51437	2.92835
C	5.45486	-13.05832	5.29910
C	4.04932	-11.84756	6.97722
C	4.62741	-13.87026	4.29778
C	2.54831	-12.08164	6.78036
H	4.40282	-13.28283	3.39433
H	4.42793	-6.26311	2.98660
H	4.21755	-10.91427	7.53782
H	6.11794	-7.71864	0.85422
H	5.64214	-13.66260	6.19867
H	6.44831	-12.83576	4.87830
H	4.46886	-12.64938	7.60214
H	3.73354	-7.44632	4.87973
H	3.62298	-9.43490	6.25944
H	2.03071	-12.05856	7.75252
H	6.06628	-11.54563	3.34295
H	6.15853	-9.55705	1.98973
H	3.67222	-14.19127	4.73941
H	5.18194	-14.77112	3.99043
H	2.35668	-13.05981	6.31449
H	2.10281	-11.30673	6.13788
H	8.36079	0.63186	-4.09986
H	4.21776	2.51957	0.45998
H	2.46371	2.31930	0.69215
H	6.73388	-6.50114	-1.07371
H	7.83421	-0.60055	-6.20500
H	3.34522	2.80938	-1.83377
H	2.20034	1.46308	-1.61114
H	6.75111	-4.40785	-2.43352
H	6.23891	-2.51933	-6.15332
H	4.02119	0.26502	-2.66756
H	5.25063	1.29882	-1.90090
H	5.14888	-3.17696	-4.02405

8d S_1:

C	5.03406	-3.34972	0.22241
C	6.20272	-1.66607	-2.82201
O	4.53220	-2.10993	0.44648
C	4.95607	-4.43499	1.04280
C	7.01692	-0.51577	-2.89920
C	3.61693	0.45056	0.55525
N	5.67372	-3.34186	-0.99532
C	5.59316	-5.65751	0.60863
C	7.58889	-0.13314	-4.11288
C	3.29479	1.88780	0.13671
N	4.35024	-0.07769	-0.60983
C	6.28746	-5.60916	-0.64258
C	7.37753	-0.89945	-5.26533
C	3.14927	1.78449	-1.38728
C	6.33745	-4.48582	-1.42542
C	6.58594	-2.05309	-5.19709
C	4.26902	0.81340	-1.77634
C	5.99998	-2.43445	-3.99014

C	5.59046	-2.05244	-1.55257
C	4.85175	-1.31570	-0.62968
H	4.43405	-4.33977	1.99282
H	7.21548	0.06291	-1.99521
H	4.23215	0.37893	1.46485
H	2.70201	-0.14539	0.72002
C	4.92630	-7.05762	2.58391
C	4.91207	-8.28125	3.34755
N	4.79460	-11.81337	5.72451
C	5.56558	-6.86362	1.37198
C	4.18892	-8.34363	4.57167
C	4.13602	-9.48747	5.34584
C	4.82021	-10.68066	4.95401
C	5.54372	-10.62553	3.72092
C	5.58643	-9.47480	2.95759
C	5.45122	-13.04612	5.28472
C	4.01105	-11.87494	6.95995
C	4.65147	-13.85178	4.25405
C	2.51552	-12.13398	6.74370
H	4.44336	-13.25480	3.35339
H	4.36830	-6.22676	3.02494
H	4.15847	-10.94314	7.52749
H	6.11075	-7.70268	0.92968
H	5.62387	-13.65890	6.18048
H	6.44835	-12.80152	4.88645
H	4.44015	-12.67691	7.57684
H	3.65146	-7.45452	4.91243
H	3.55691	-9.45984	6.26687
H	1.99452	-12.13103	7.71392
H	6.07995	-11.50047	3.35848
H	6.15927	-9.49760	2.02882
H	3.69115	-14.18876	4.67081
H	5.22613	-14.74128	3.95196
H	2.34626	-13.11025	6.26636
H	2.06289	-11.35831	6.10769
H	8.21600	0.76056	-4.15597
H	4.13472	2.55275	0.39524
H	2.39344	2.26863	0.63667
H	6.81002	-6.49947	-0.99827
H	7.83100	-0.60093	-6.21343
H	3.24506	2.75060	-1.90220
H	2.16987	1.35253	-1.64922
H	6.87107	-4.41708	-2.36989
H	6.41338	-2.65296	-6.09388
H	4.06100	0.23354	-2.68761
H	5.22496	1.34337	-1.92570
H	5.35668	-3.31638	-3.95494

9b

S_0:

C	4.05808	-7.63341	0.58785
C	4.29315	-3.94572	0.24221
S	2.35224	-7.46347	0.81222
C	4.78736	-8.80018	0.63098
C	4.78529	-3.48241	-0.98207
C	0.03586	-5.55919	1.00929
N	4.64459	-6.41528	0.36196
C	6.17512	-8.77204	0.43709
C	5.27314	-2.18739	-1.09747
C	-0.90061	-4.35293	0.99703
N	1.33858	-4.98357	0.64362
C	6.75023	-7.49434	0.21975
C	5.26987	-1.33936	0.00706
C	-0.29674	-3.45803	-0.08953
C	5.99860	-6.35817	0.18848
C	4.78421	-1.79320	1.22866
C	1.20861	-3.60938	0.12688
C	4.30416	-3.09256	1.34813
C	3.76584	-5.31766	0.37726
C	2.46622	-5.71150	0.60101
H	4.25833	-9.72286	0.81688
H	4.77237	-4.13613	-1.84519
H	0.08794	-6.04666	1.98463
H	-0.26997	-6.30426	0.26522
C	6.61866	-11.21639	0.63325
C	7.43506	-12.40492	0.65209
N	9.68309	-15.99423	0.69221
C	7.02561	-9.93779	0.45047
C	8.83396	-12.40639	0.48973
C	9.57094	-13.56776	0.51734
C	8.95649	-14.83574	0.70167
C	7.55117	-14.83311	0.88109
C	6.82950	-13.65863	0.84981
C	11.14030	-15.97545	0.56781
C	9.04648	-17.28728	0.94004
C	11.88020	-15.64520	1.86535
C	8.78611	-17.58938	2.41690
H	11.69276	-16.40369	2.62554
H	5.55959	-11.40374	0.78270
H	9.69816	-18.05293	0.52008
H	8.07854	-9.73351	0.29466
H	11.42472	-15.27265	-0.21735
H	11.44522	-16.96001	0.21450
H	8.11438	-17.34159	0.37470
H	9.36309	-11.47353	0.34317
H	10.64022	-13.49340	0.39629
H	8.26673	-18.54466	2.51575
H	7.01787	-15.75566	1.04826

H	5.75519	-13.70747	0.98762
H	11.56654	-14.68005	2.26472
H	12.95562	-15.60514	1.68111
H	8.16843	-16.81638	2.87544
H	9.72128	-17.65093	2.97367
H	5.64628	-1.83649	-2.05090
H	-0.86874	-3.84764	1.96430
H	-1.93110	-4.63997	0.79580
H	7.81716	-7.40020	0.07417
H	5.64464	-0.32814	-0.08556
H	-0.61555	-2.41956	-0.01650
H	-0.57048	-3.83103	-1.07863
H	6.41720	-5.37817	0.03007
H	4.78075	-1.13790	2.09001
H	1.78814	-3.49239	-0.78762
H	1.58284	-2.89100	0.85962
H	3.92897	-3.44971	2.29855

9b S_1:

C	4.07579	-7.62258	0.72613
C	4.30410	-3.93913	0.27191
S	2.36492	-7.42567	0.99853
C	4.78036	-8.78752	0.76458
C	4.96852	-3.58409	-0.91016
C	0.07278	-5.48631	1.14553
N	4.67087	-6.39844	0.47509
C	6.19522	-8.77771	0.55295
C	5.42751	-2.28809	-1.09540
C	-0.89147	-4.31714	0.95858
N	1.36817	-4.94133	0.71415
C	6.78433	-7.49358	0.36418
C	5.23026	-1.32694	-0.10632
C	-0.28855	-3.56167	-0.22932
C	6.05469	-6.35042	0.33476
C	4.57495	-1.67127	1.07182
C	1.21382	-3.64267	0.03648
C	4.11847	-2.96906	1.26329
C	3.80089	-5.30362	0.47662
C	2.49306	-5.68061	0.72390
H	4.24859	-9.70395	0.97153
H	5.10671	-4.32331	-1.68882
H	0.12692	-5.84031	2.17667
H	-0.20774	-6.33226	0.50547
C	6.56058	-11.24656	0.70151
C	7.38604	-12.41803	0.69129
N	9.67888	-15.98006	0.64582
C	6.99642	-9.94837	0.54454
C	8.79484	-12.40127	0.52226
C	9.54285	-13.55242	0.51684
C	8.94194	-14.83322	0.67553
C	7.53069	-14.85474	0.85831
C	6.79578	-13.69476	0.86097
C	11.13489	-15.94591	0.50043
C	9.05992	-17.28860	0.86312
C	11.88272	-15.63635	1.80003
C	8.81023	-17.62084	2.33663
H	11.71108	-16.41342	2.54432
H	5.50140	-11.42943	0.84231
H	9.72333	-18.03341	0.42656
H	8.05626	-9.77451	0.39493
H	11.39966	-15.22105	-0.27053
H	11.44214	-16.92029	0.12419
H	8.12694	-17.34032	0.30040
H	9.31218	-11.45992	0.39629
H	10.61040	-13.46446	0.39057
H	8.30882	-18.58731	2.41243
H	7.01185	-15.78966	0.99963
H	5.72312	-13.75916	1.00025
H	11.56467	-14.68250	2.22134
H	12.95440	-15.58379	1.60029
H	8.17889	-16.86834	2.80960
H	9.74766	-17.67730	2.88913
H	5.93147	-2.02444	-2.01625
H	-0.89021	-3.68589	1.84922
H	-1.91045	-4.65654	0.78202
H	7.85645	-7.41032	0.24483
H	5.58633	-0.31565	-0.25413
H	-0.63238	-2.53115	-0.30227
H	-0.53096	-4.07163	-1.16396
H	6.48729	-5.37312	0.21104
H	4.42416	-0.93064	1.84653
H	1.82191	-3.61669	-0.86688
H	1.54431	-2.83045	0.68828
H	3.61885	-3.23930	2.1843

12. NMR Spectra

1-(Pyrrolidin-1-yl)-2-(4-((trifluoromethyl)thio)phenyl)ethan-1-one (1c)

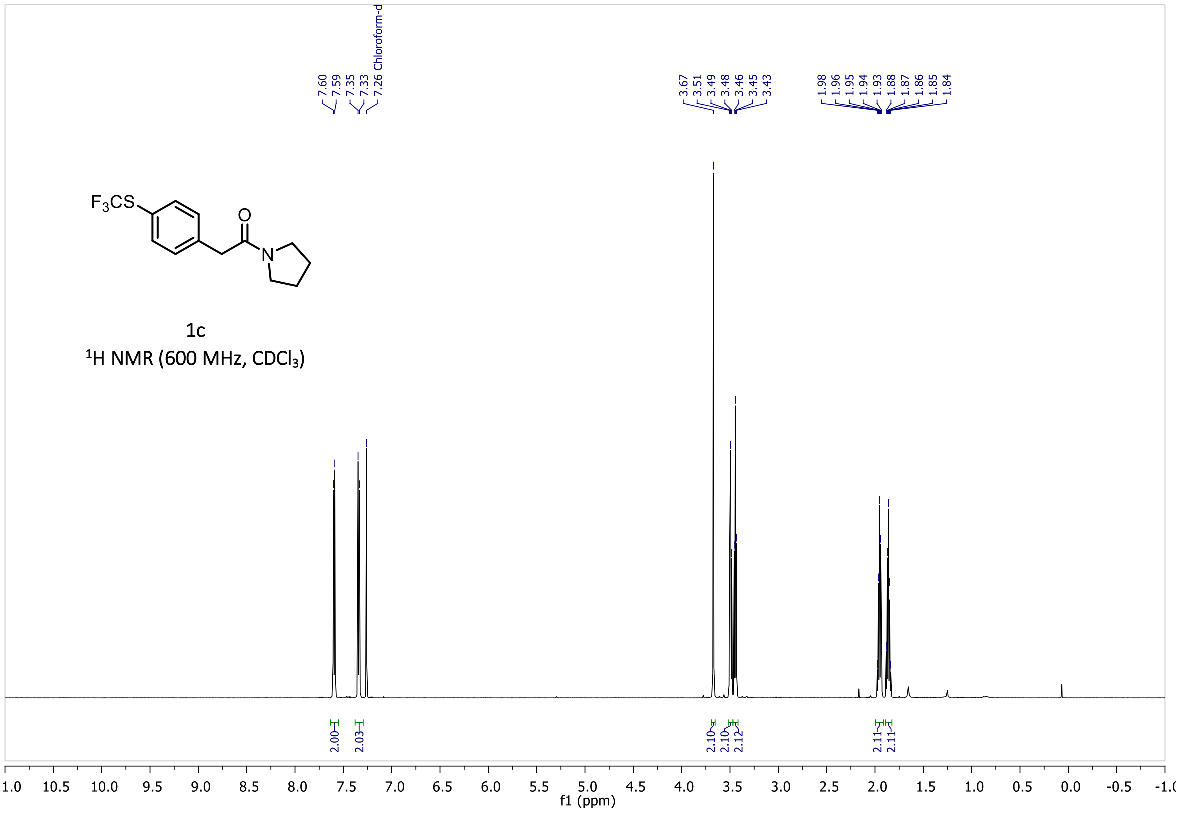

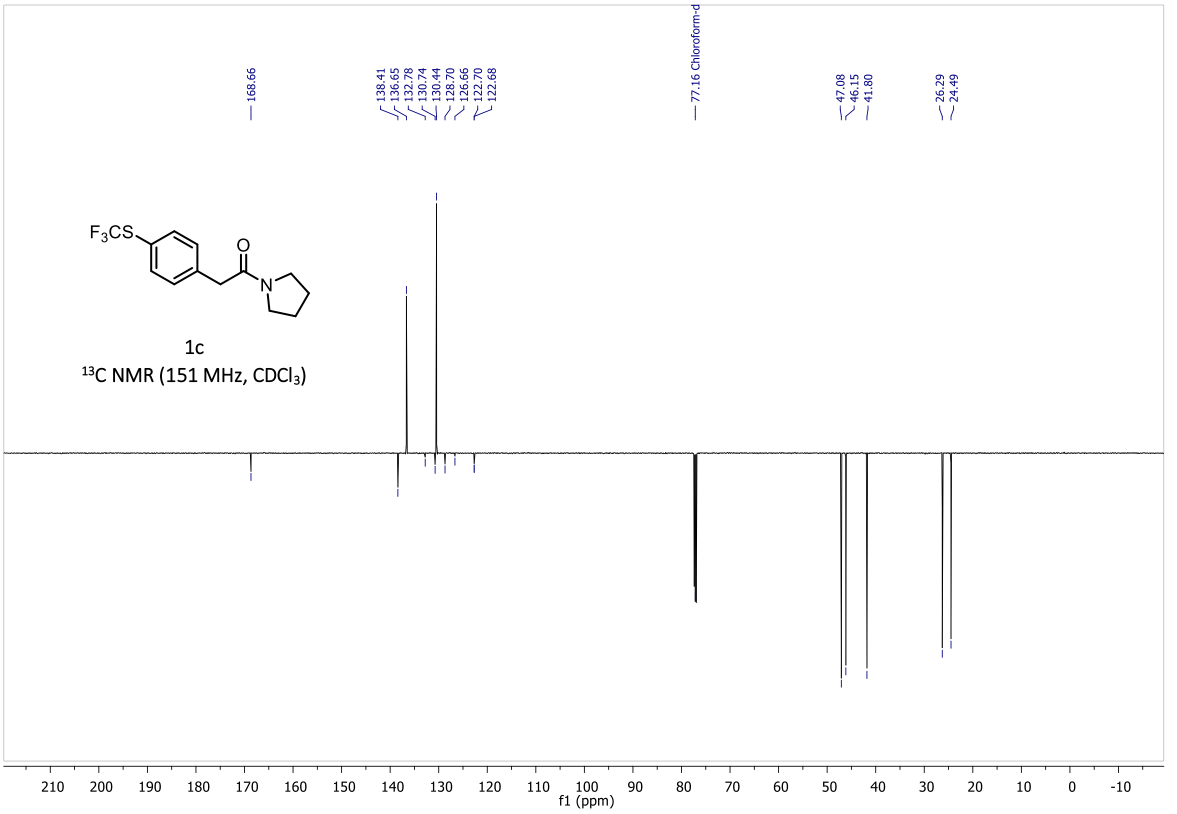

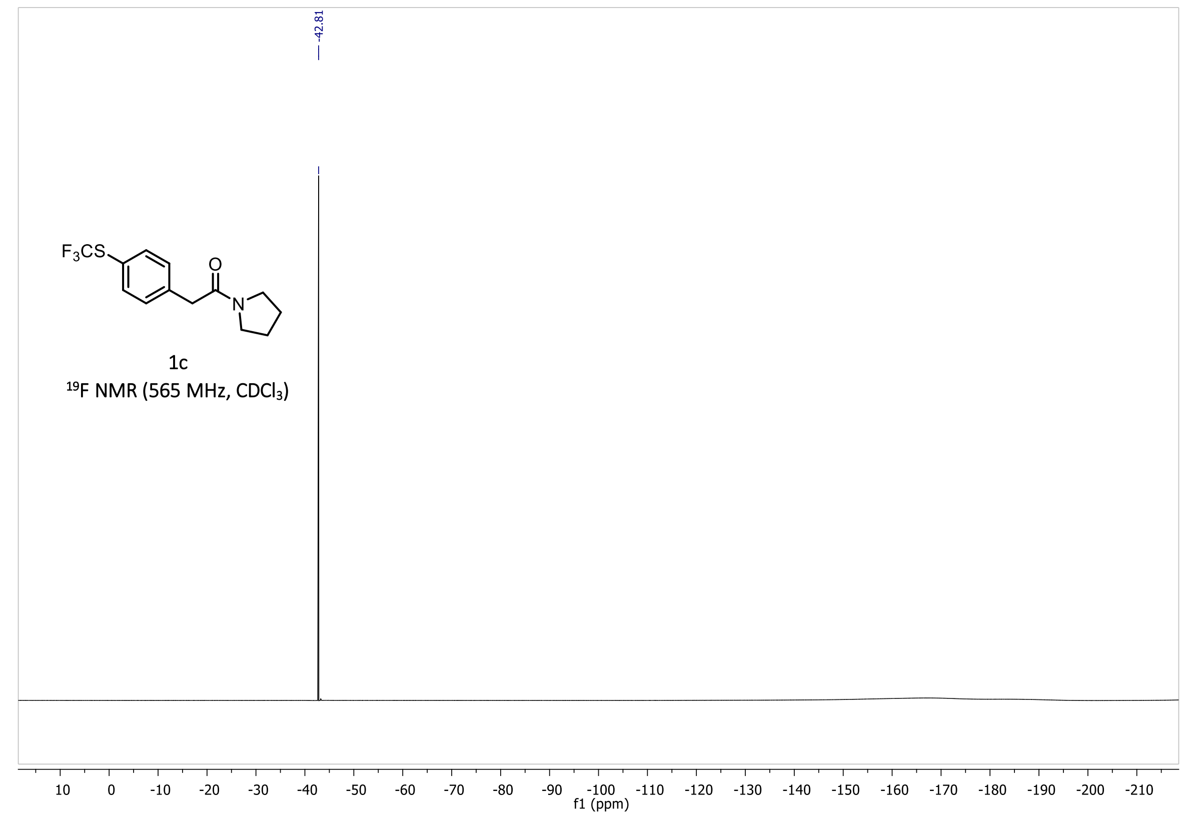

2-Chloro-4-(phenanthren-9-yl)pyridine (2b)

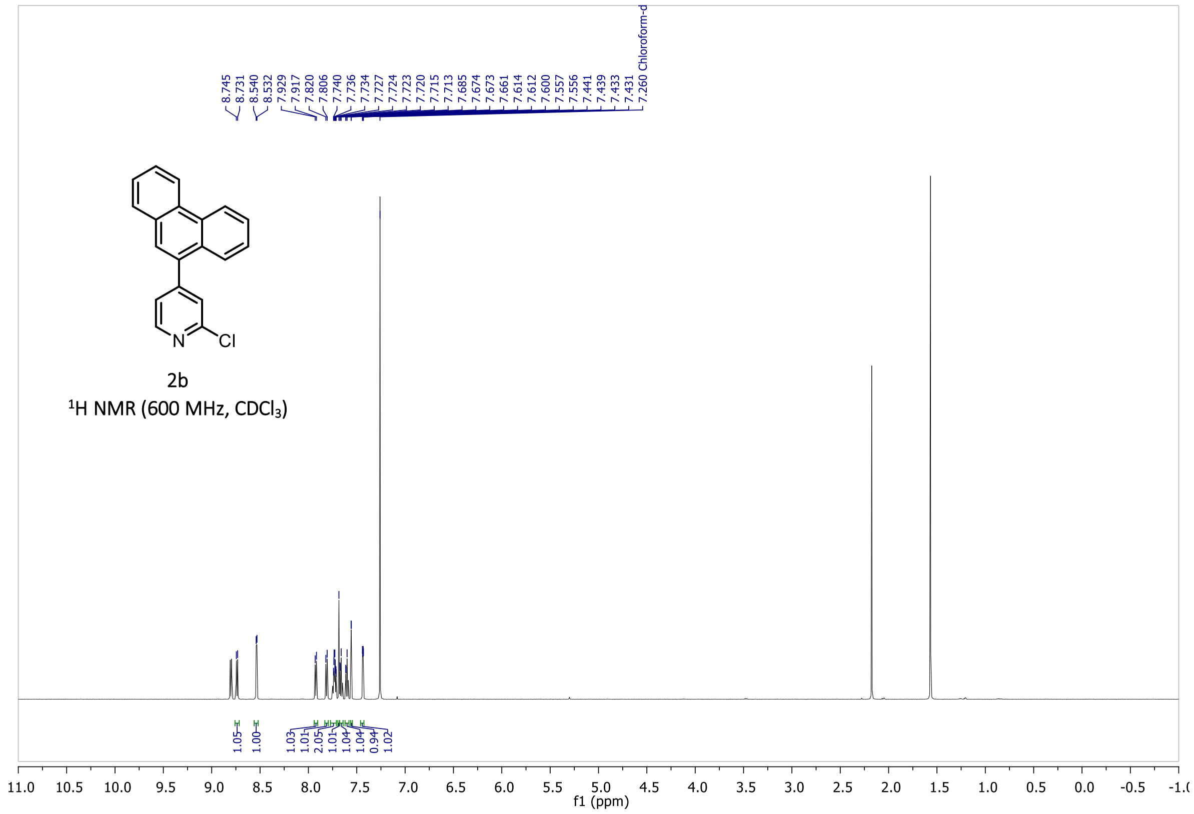

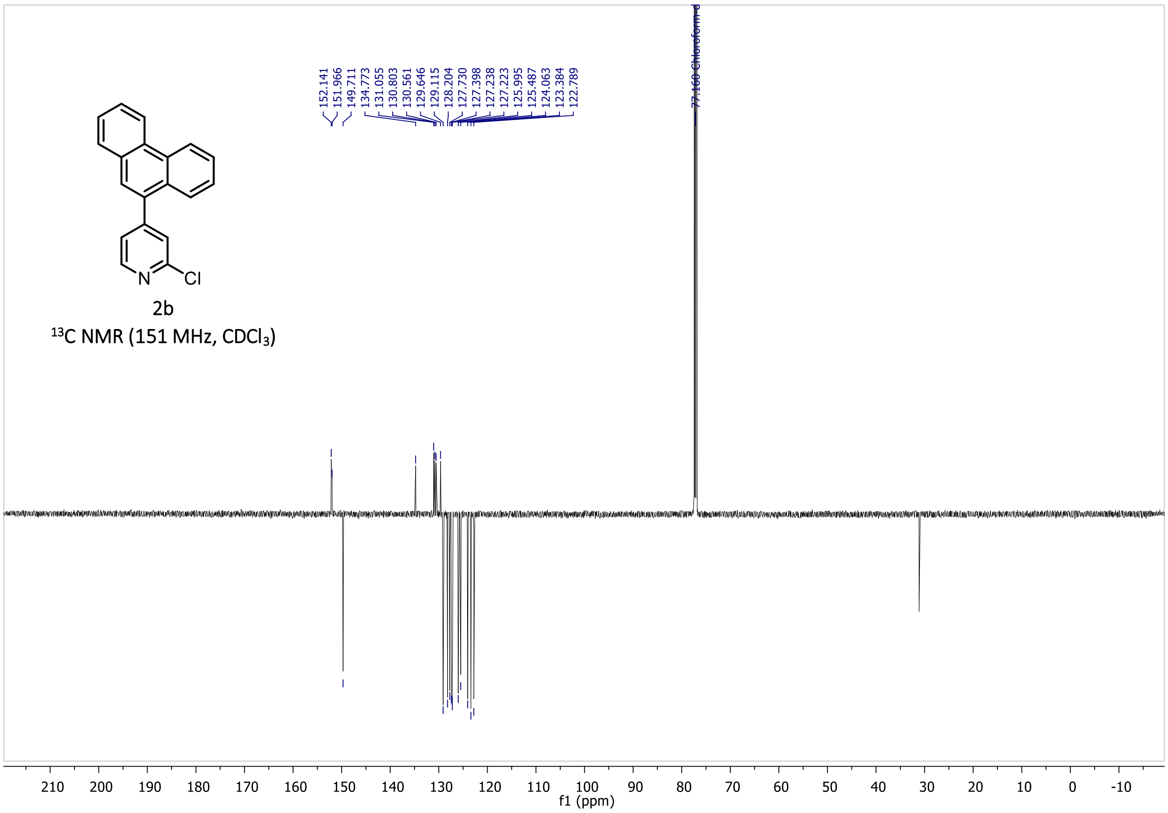

2-Chloro-4-phenylpyridine (2c)

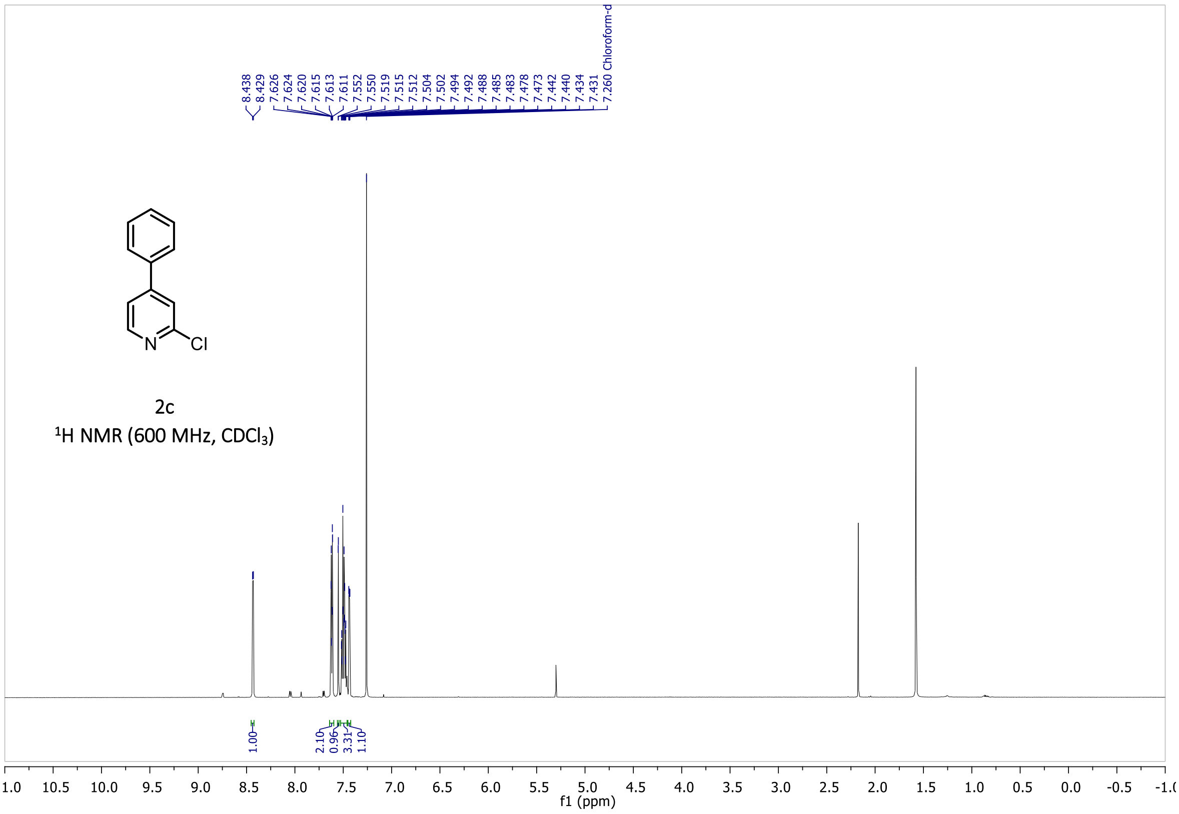

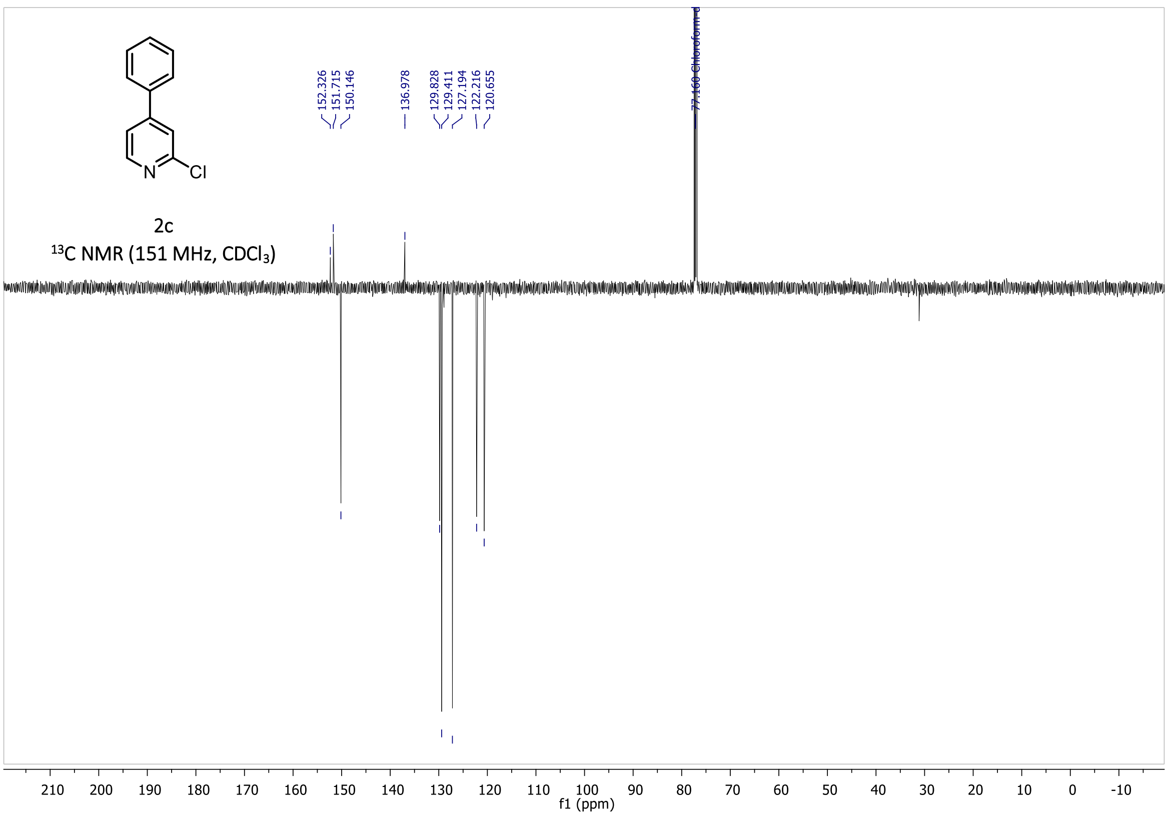

N,N-Diethyl-4'-(4,4,5,5-tetramethyl-1,3,2-dioxaborolan-2-yl)-[1,1'-biphenyl]-4-amine (10a)

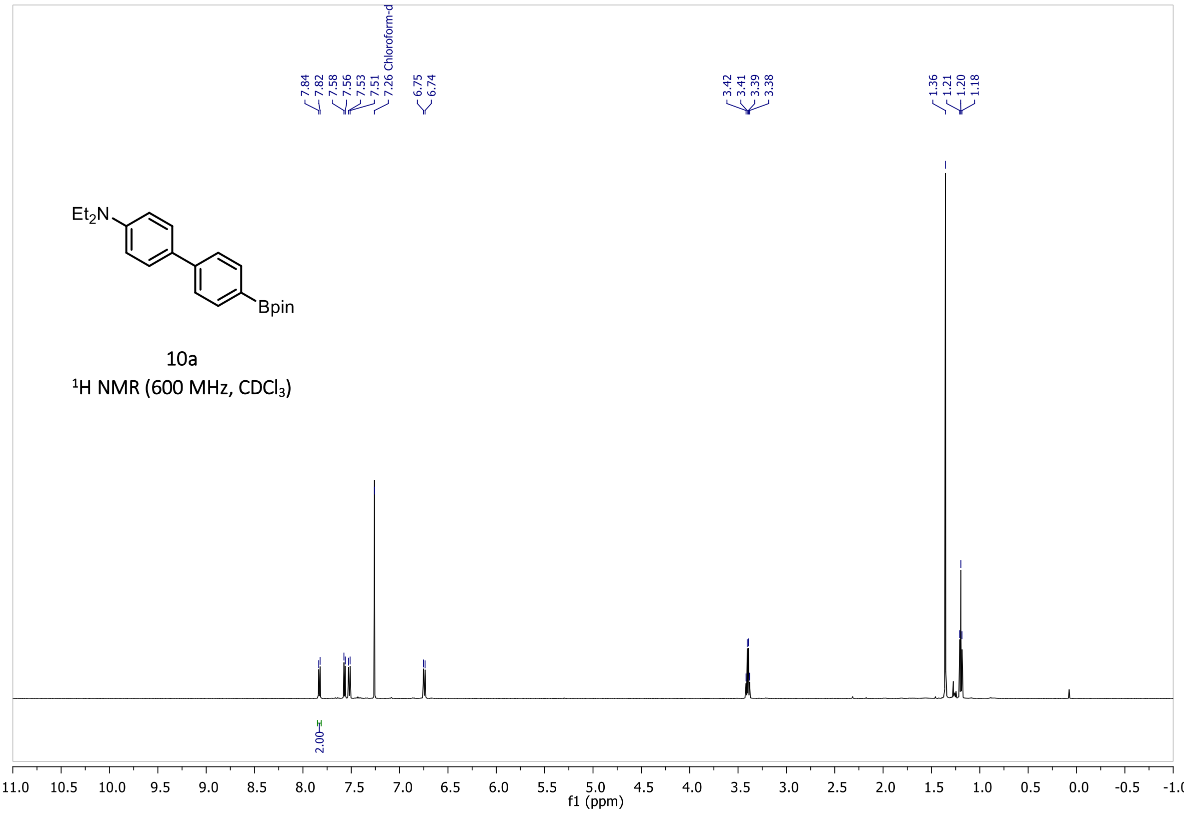

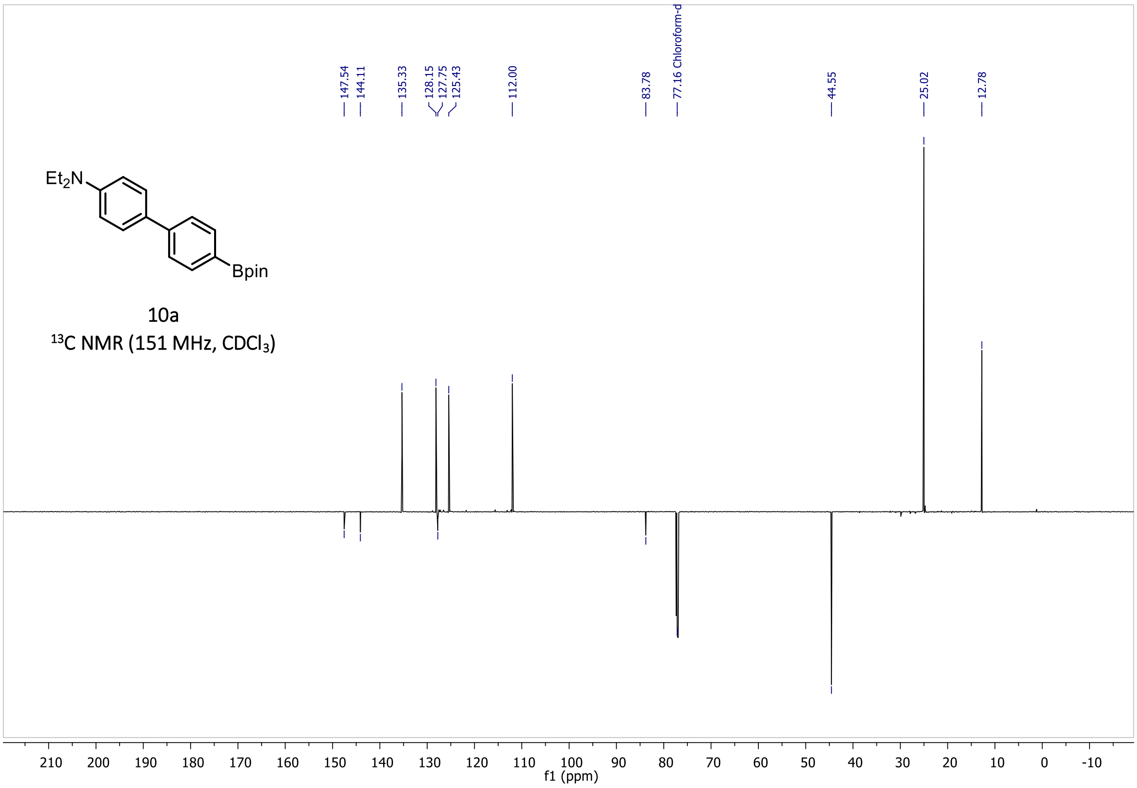

(*E*)-9-(2-(4,4,5,5-Tetramethyl-1,3,2-dioxaborolan-2-yl)vinyl)-2,3,6,7-tetrahydro-1H,5H-pyrido[3,2,1-ij]quinoline (10b)

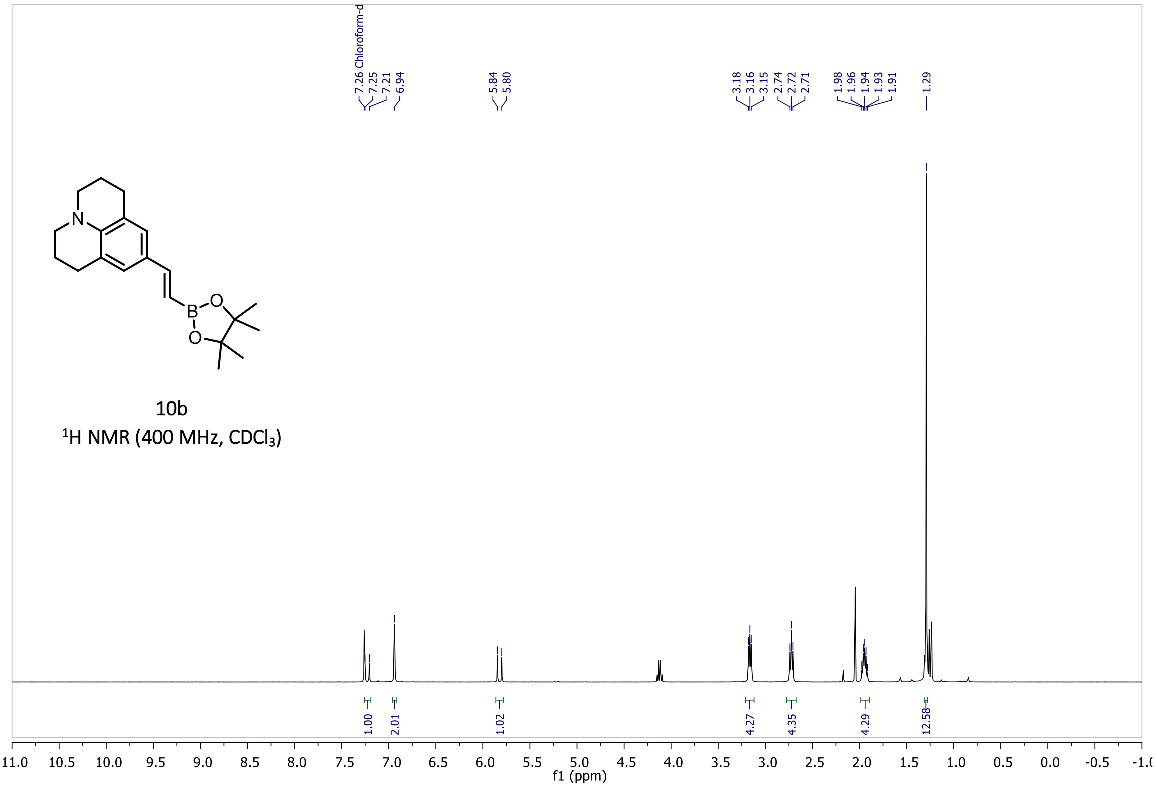

(*E*)-*N*,*N*-Diethyl-4-(2-(4,4,5,5-tetramethyl-1,3,2-dioxaborolan-2-yl)vinyl)aniline (10c)

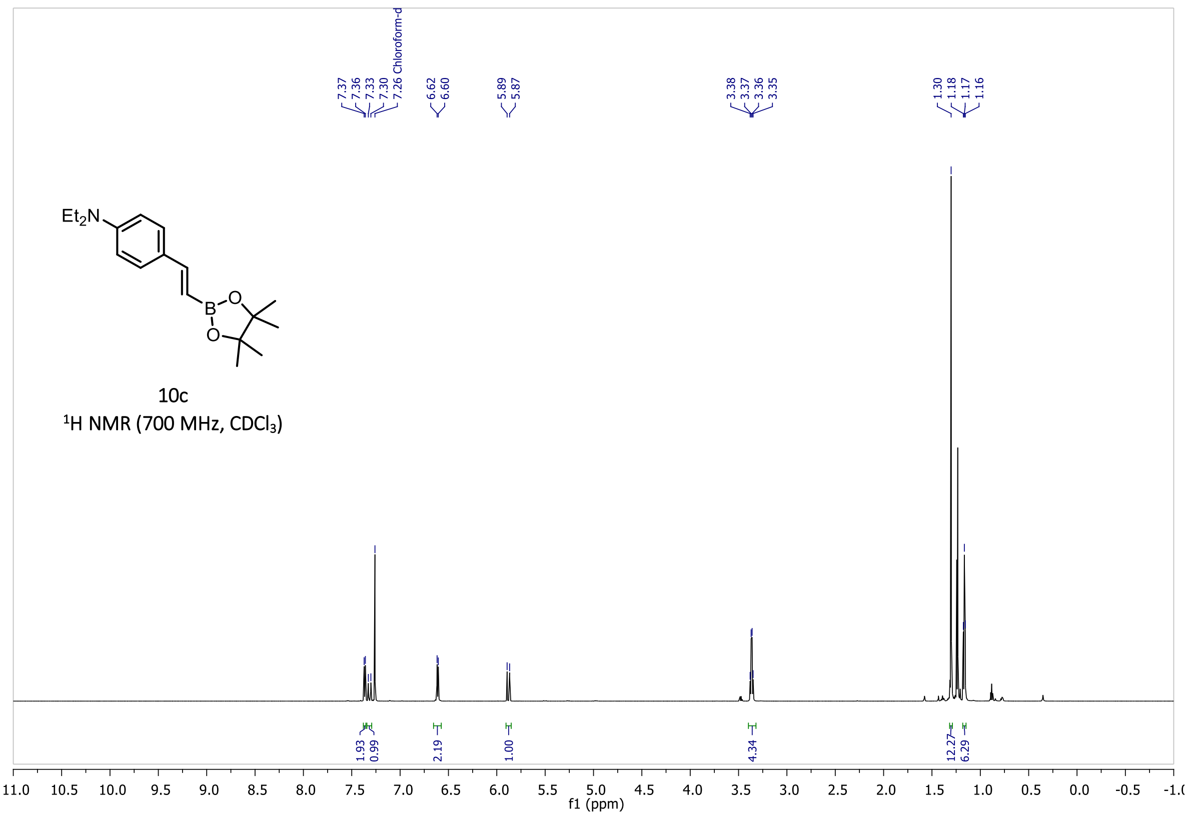

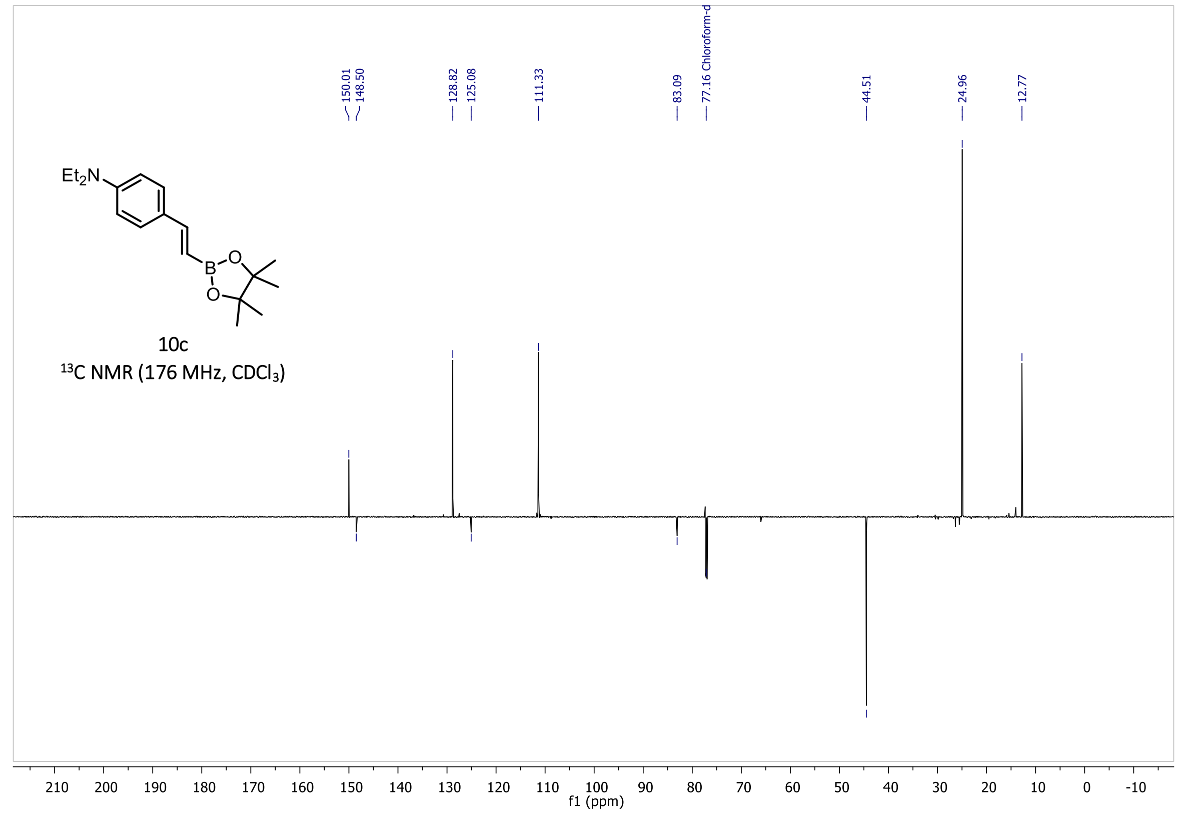

(*E*)-4-(4-(2-(4,4,5,5-Tetramethyl-1,3,2-dioxaborolan-2-yl)vinyl)phenyl)morpholine (10d)

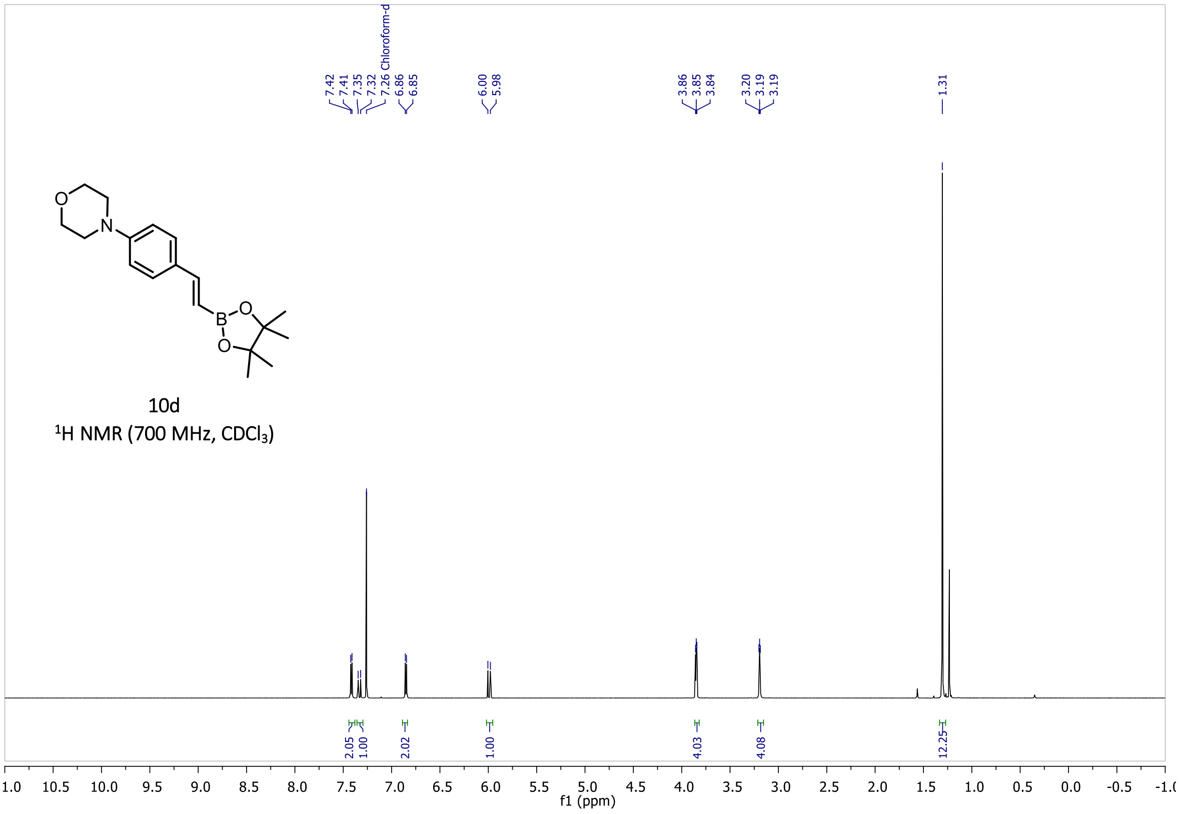

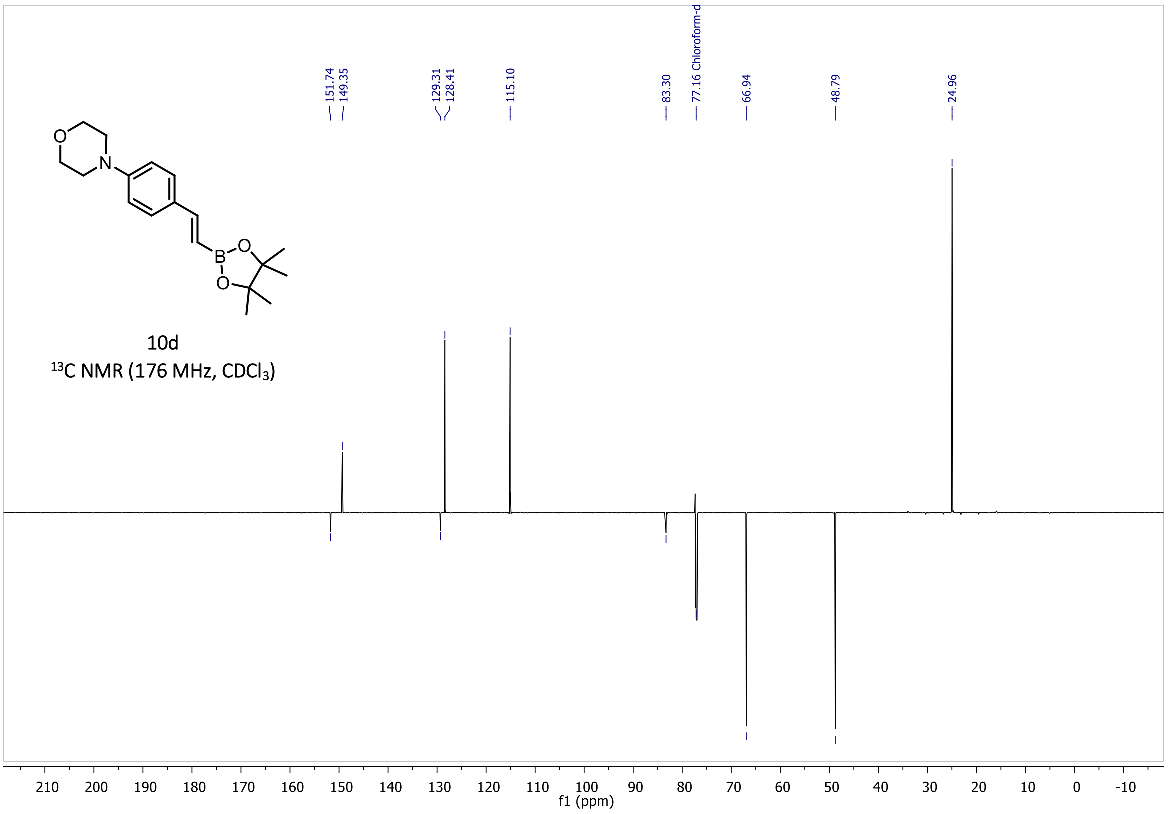

9-((1*E*,3*E*)-4-(4,4,5,5-Tetramethyl-1,3,2-dioxaborolan-2-yl)buta-1,3-dien-1-yl)-2,3,6,7-tetrahydro-1H,5H-pyrido[3,2,1-ij]quinoline (10e)

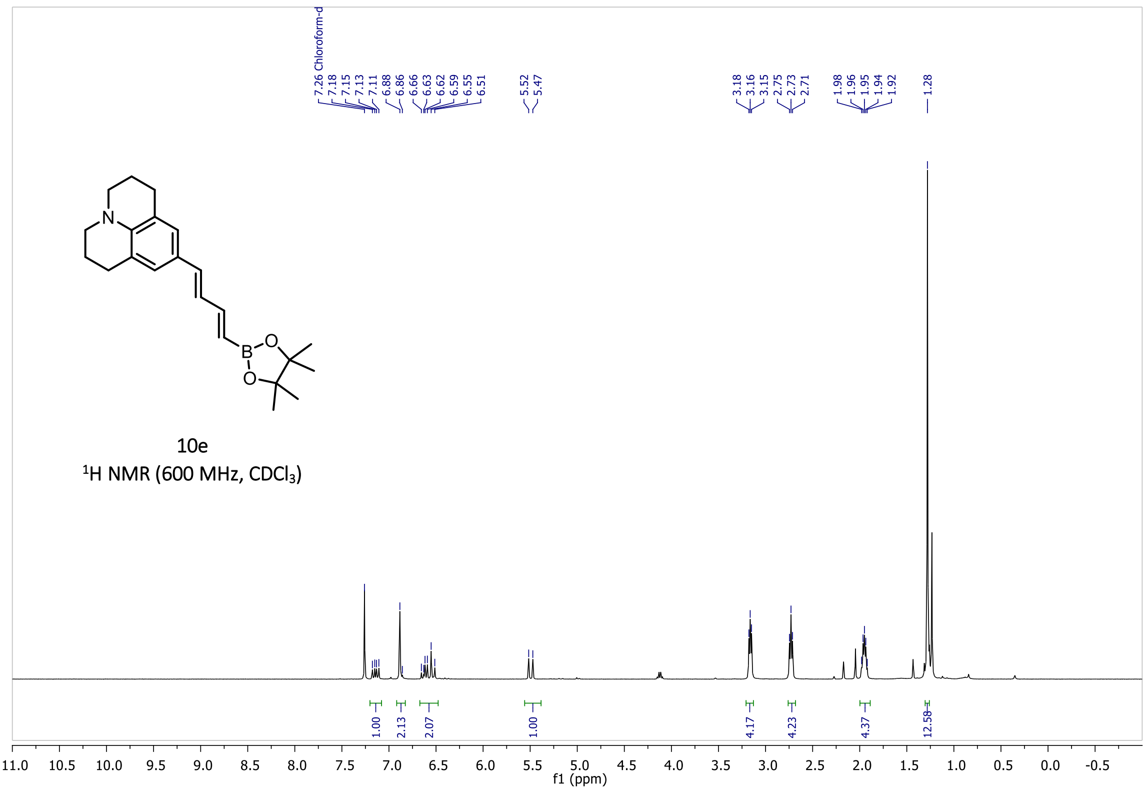

N,*N*-Diethyl-4-((1*E*,3*E*)-4-(4,4,5,5-tetramethyl-1,3,2-dioxaborolan-2-yl)buta-1,3-dien-1-yl)aniline (10f)

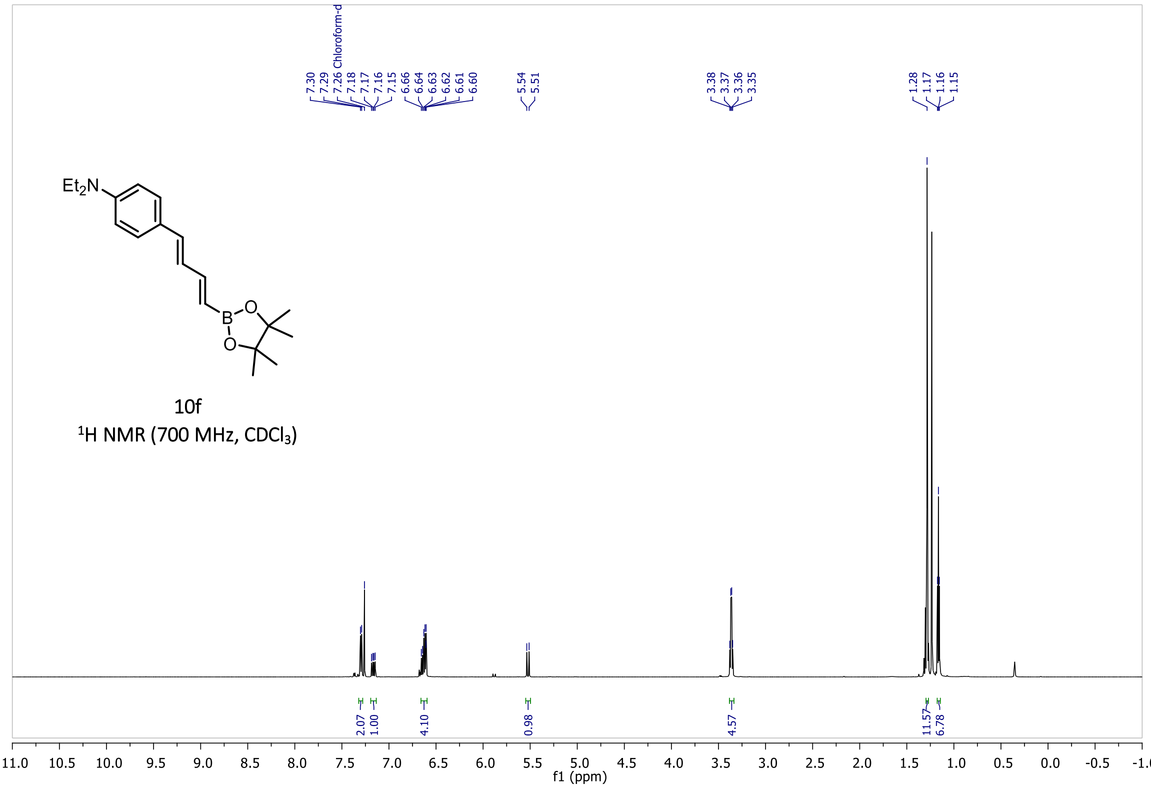

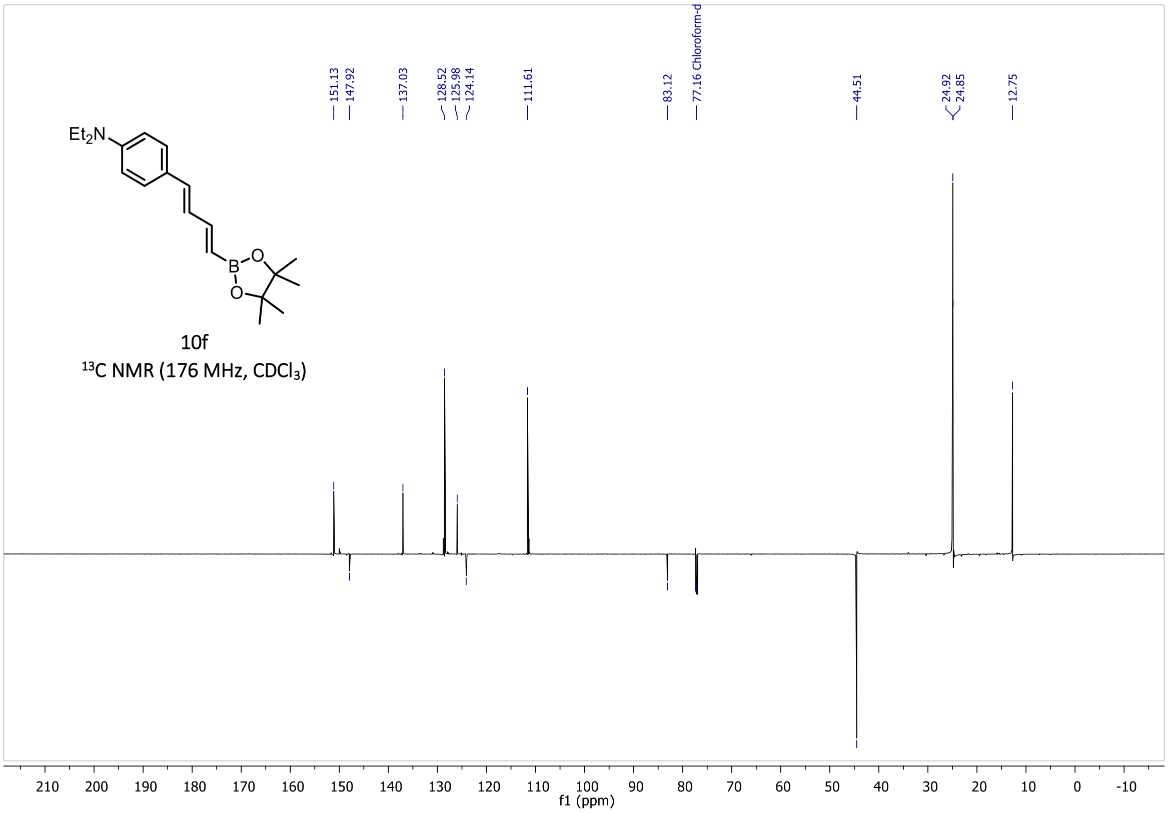

4-(4-((1*E*,3*E*)-4-(4,4,5,5-Tetramethyl-1,3,2-dioxaborolan-2-yl)buta-1,3-dien-1-yl)phenyl)morpholine (10g)

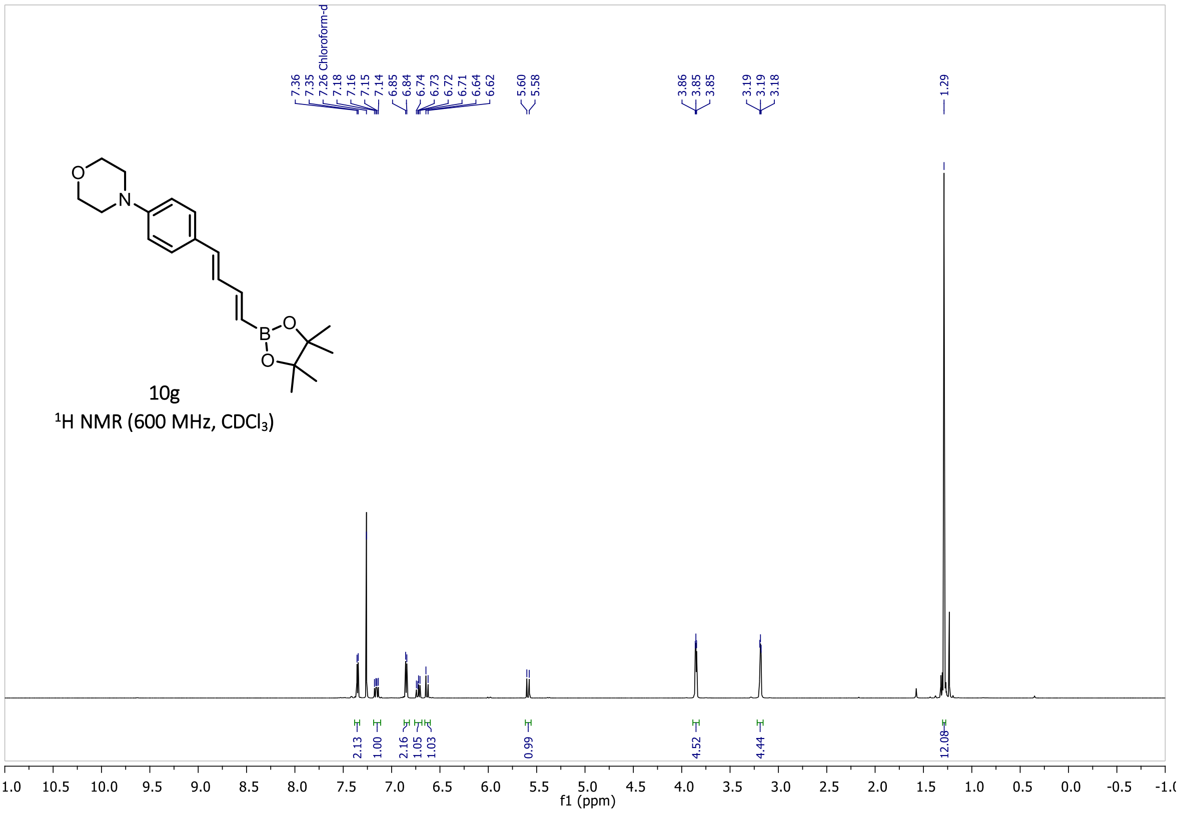

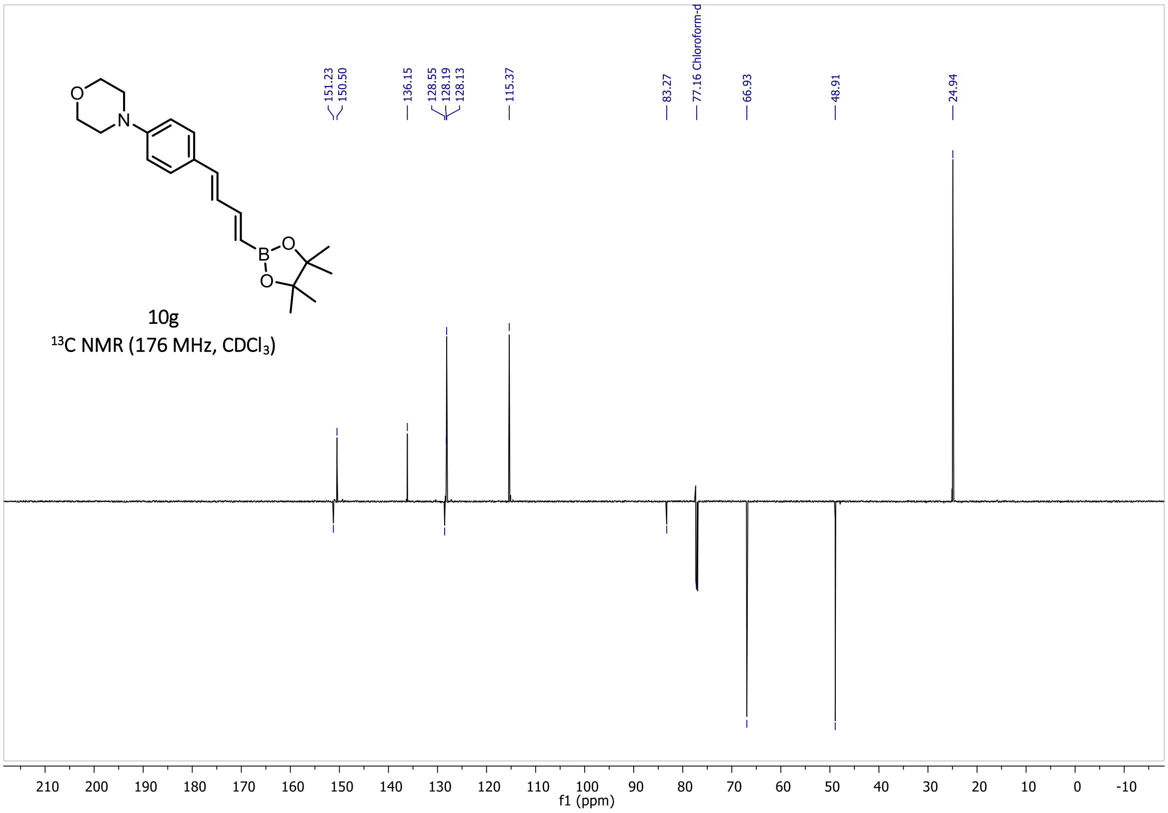

4,4,5,5-Tetramethyl-2-((1*E*,3*E*)-4-(p-tolyl)buta-1,3-dien-1-yl)-1,3,2-dioxaborolane (10h)

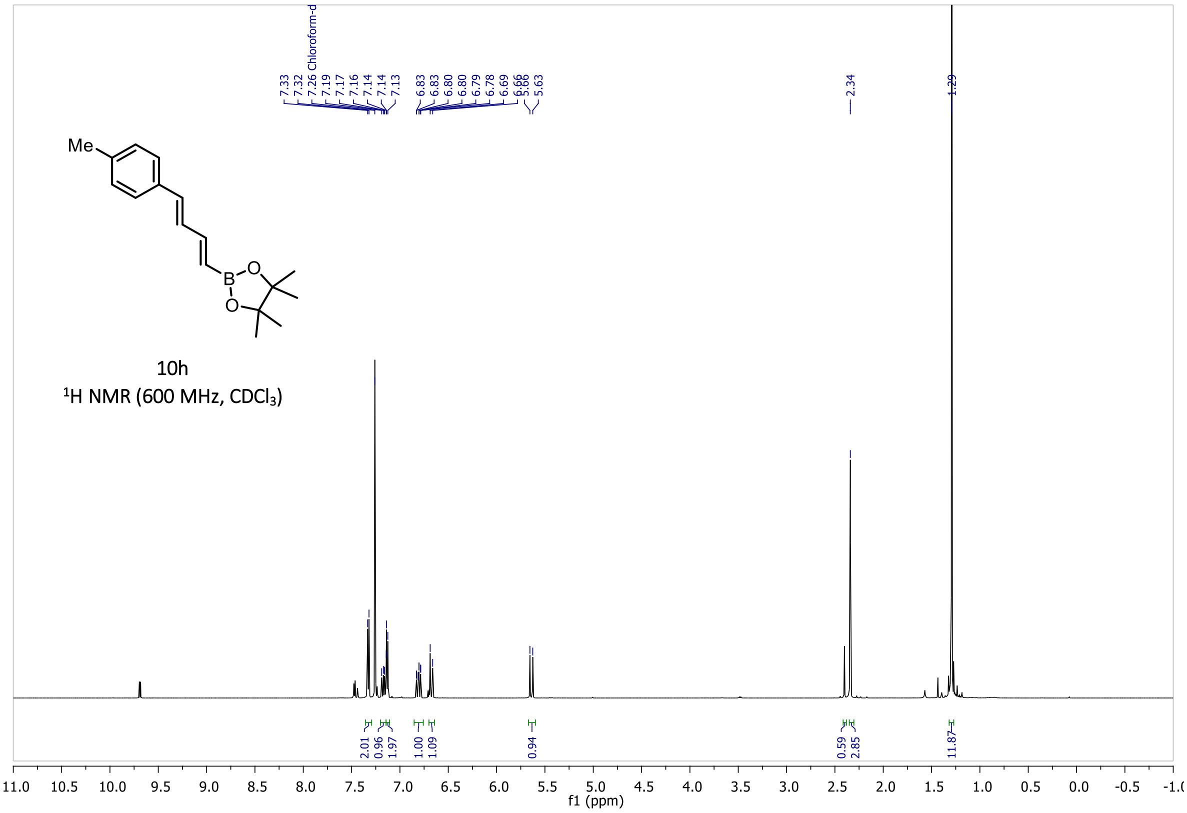

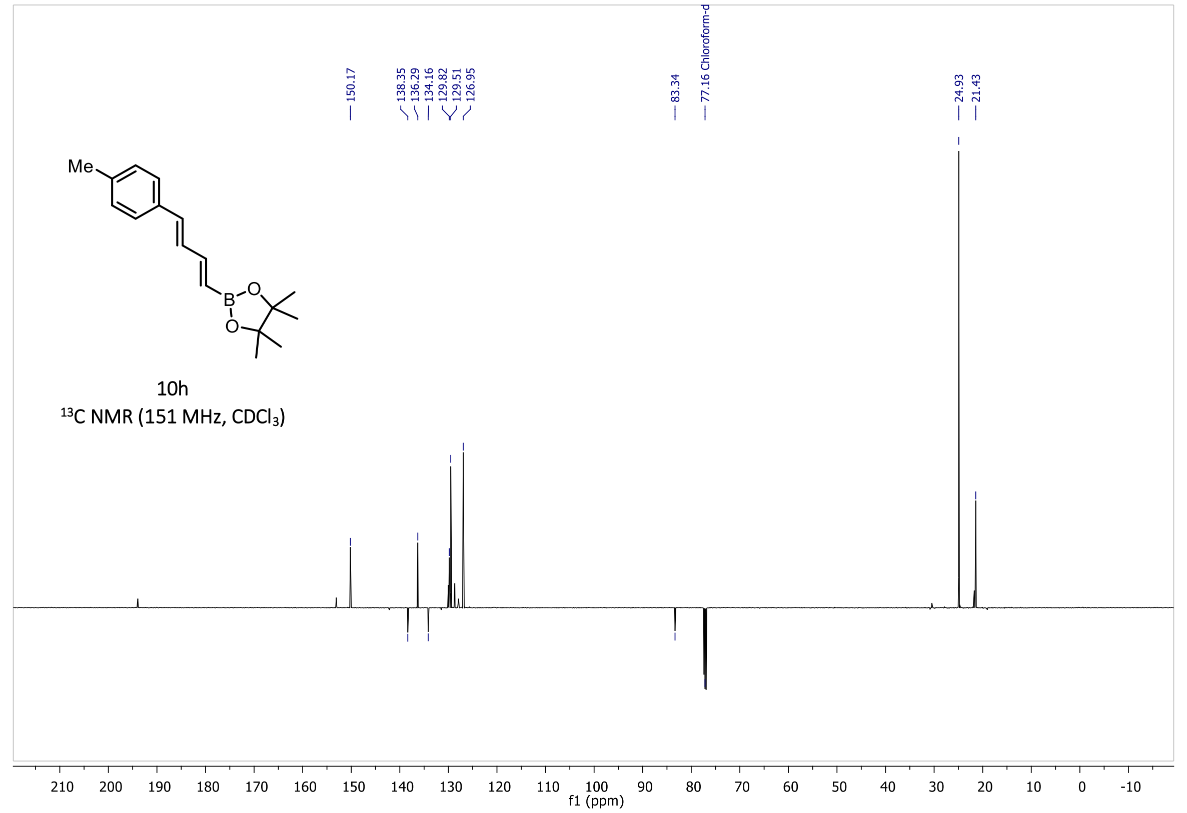

4,4,5,5-Tetramethyl-2-((1*E*,3*E*,5*E*)-6-(4-(trifluoromethyl)phenyl)hexa-1,3,5-trien-1-yl)-1,3,2-dioxaborolane (10i)

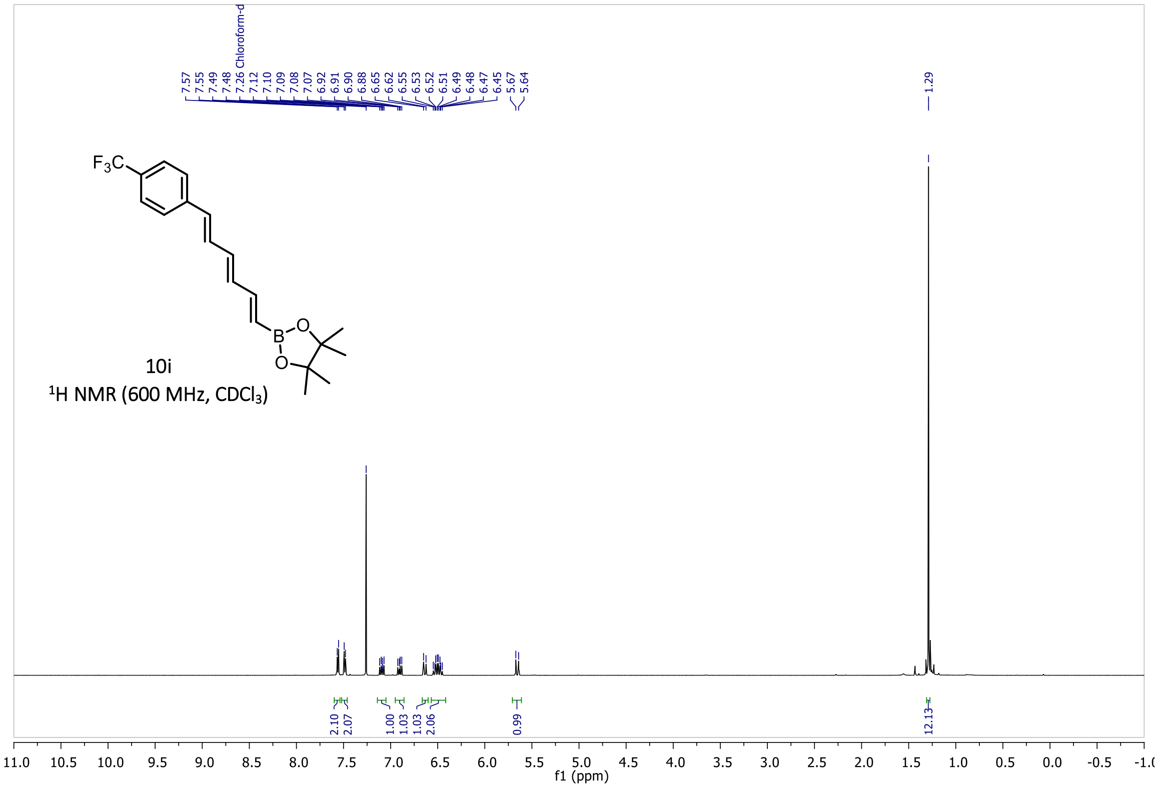

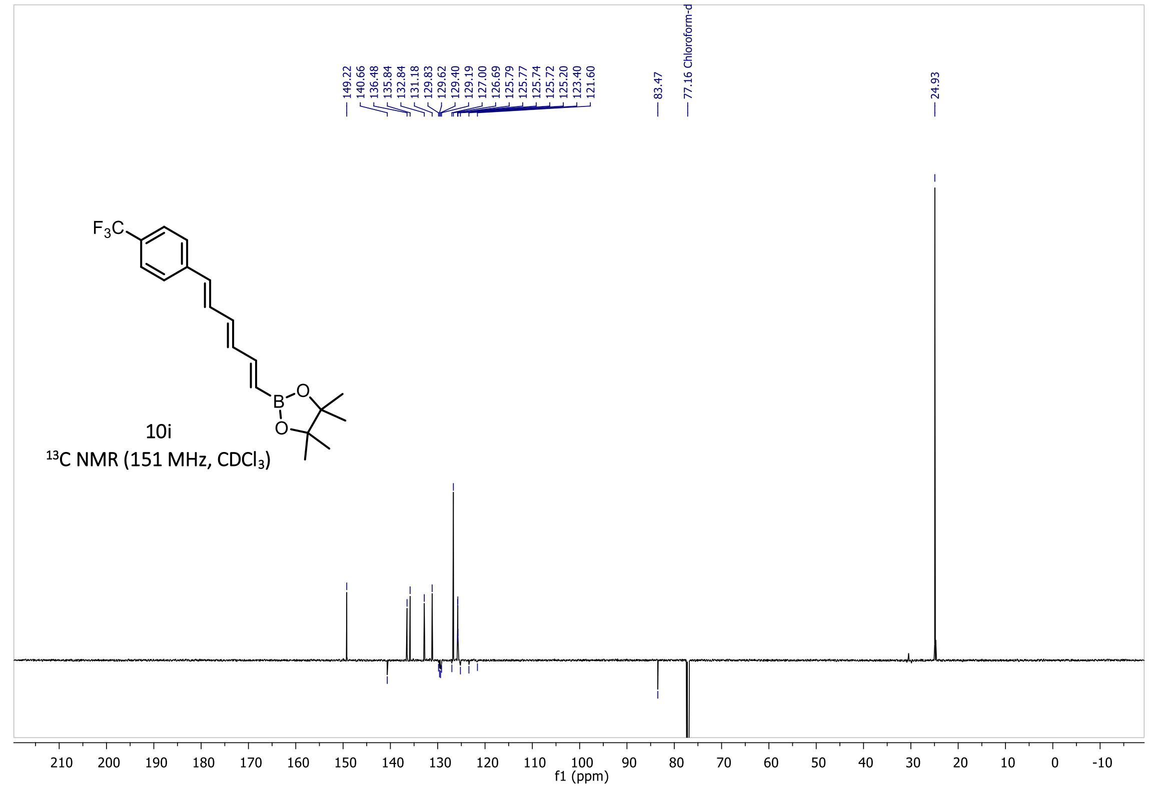

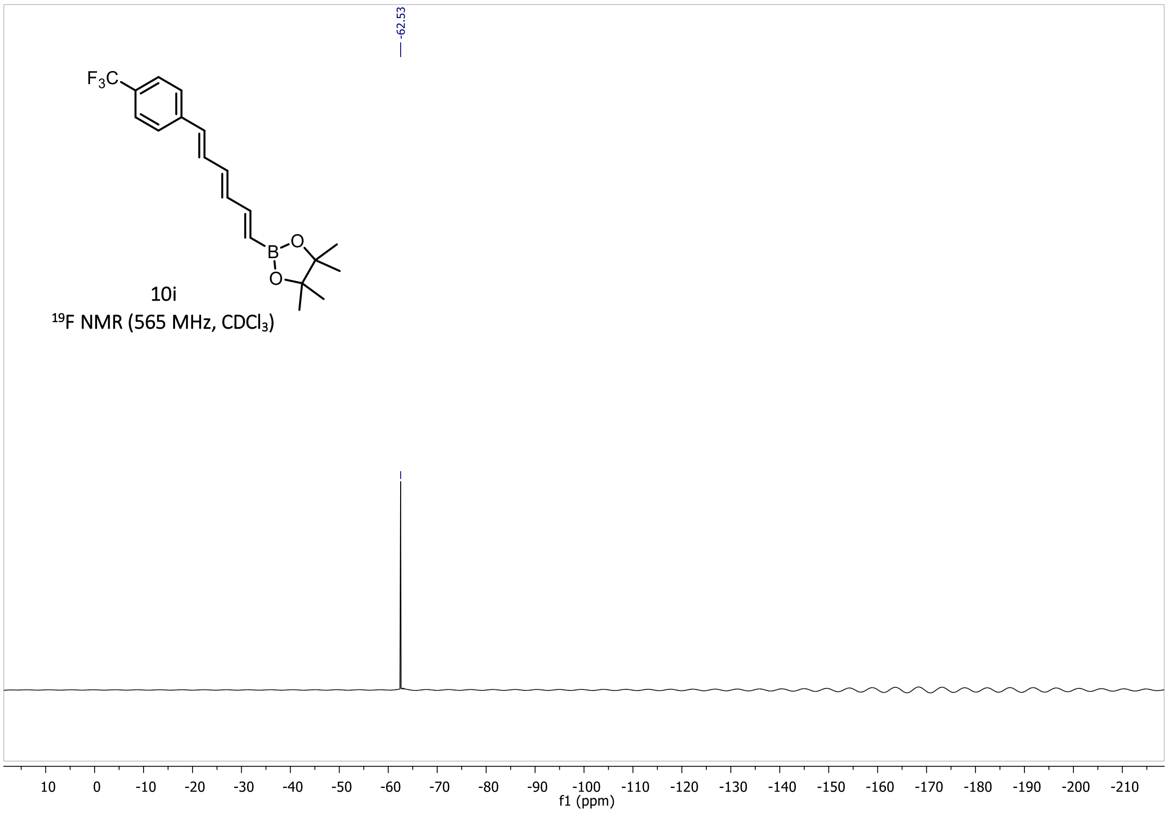

1-Phenyl-5-((4-vinylbenzyl)thio)-1*H*-tetrazole (10kb)

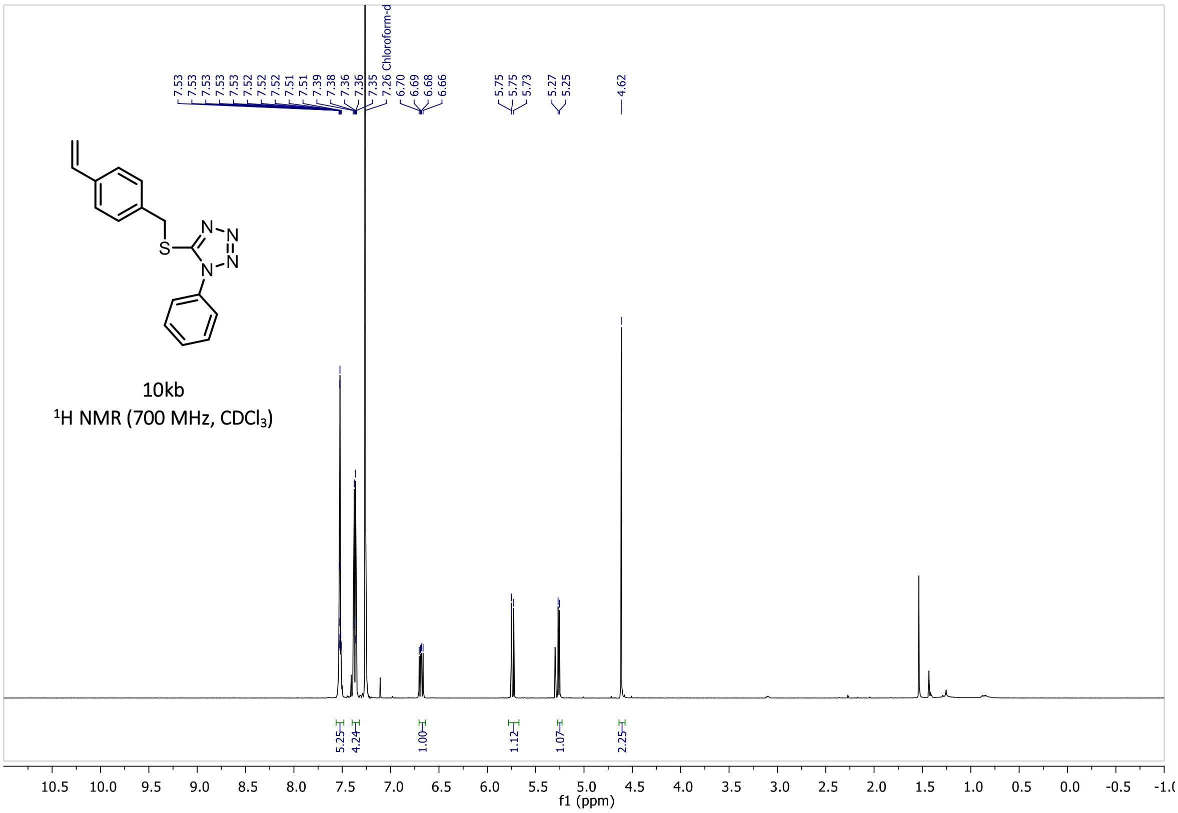

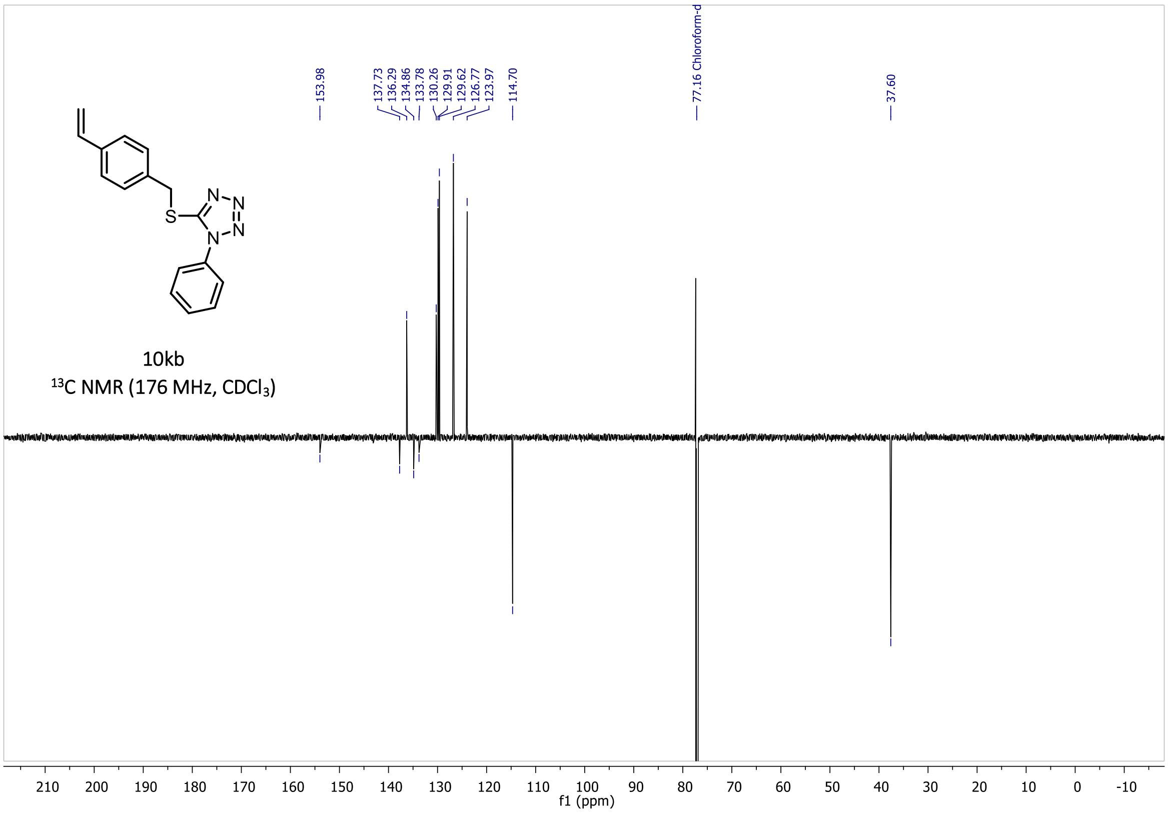

1-Phenyl-5-((4-vinylbenzyl)sulfonyl)-1*H*-tetrazole (10kc)

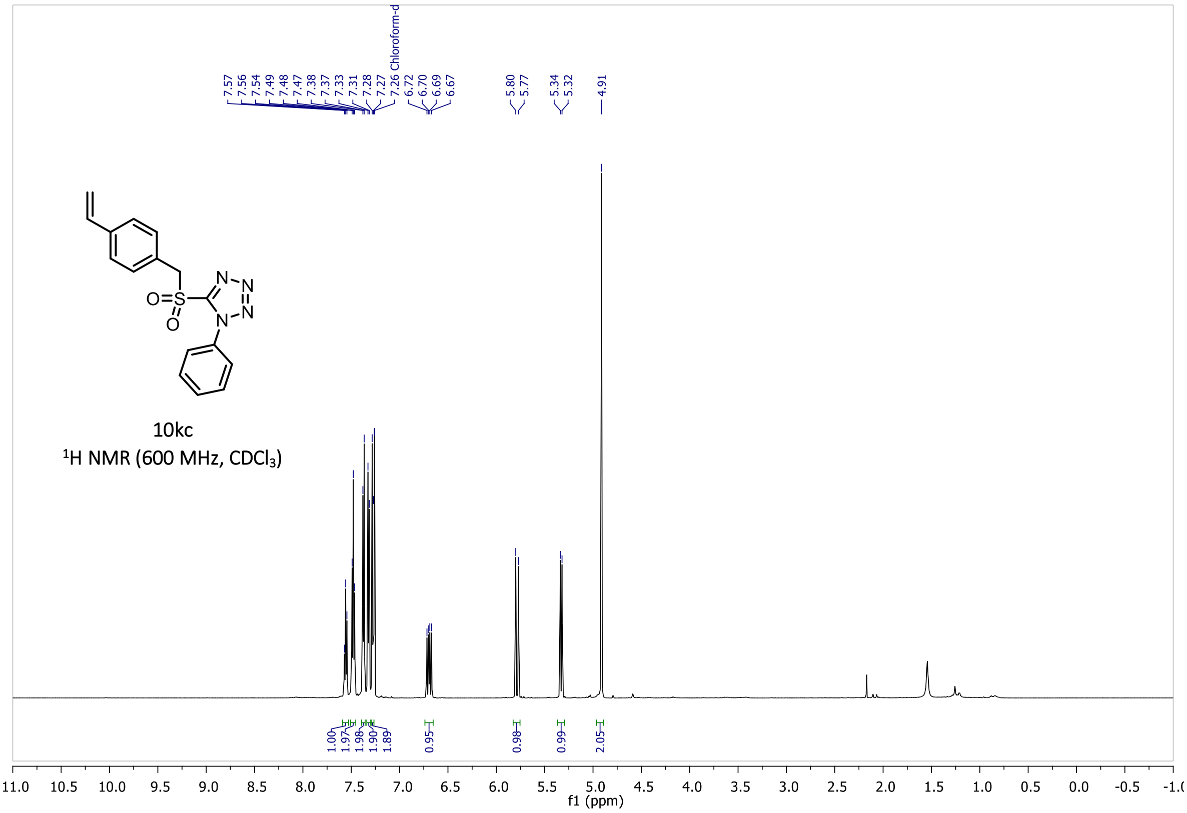

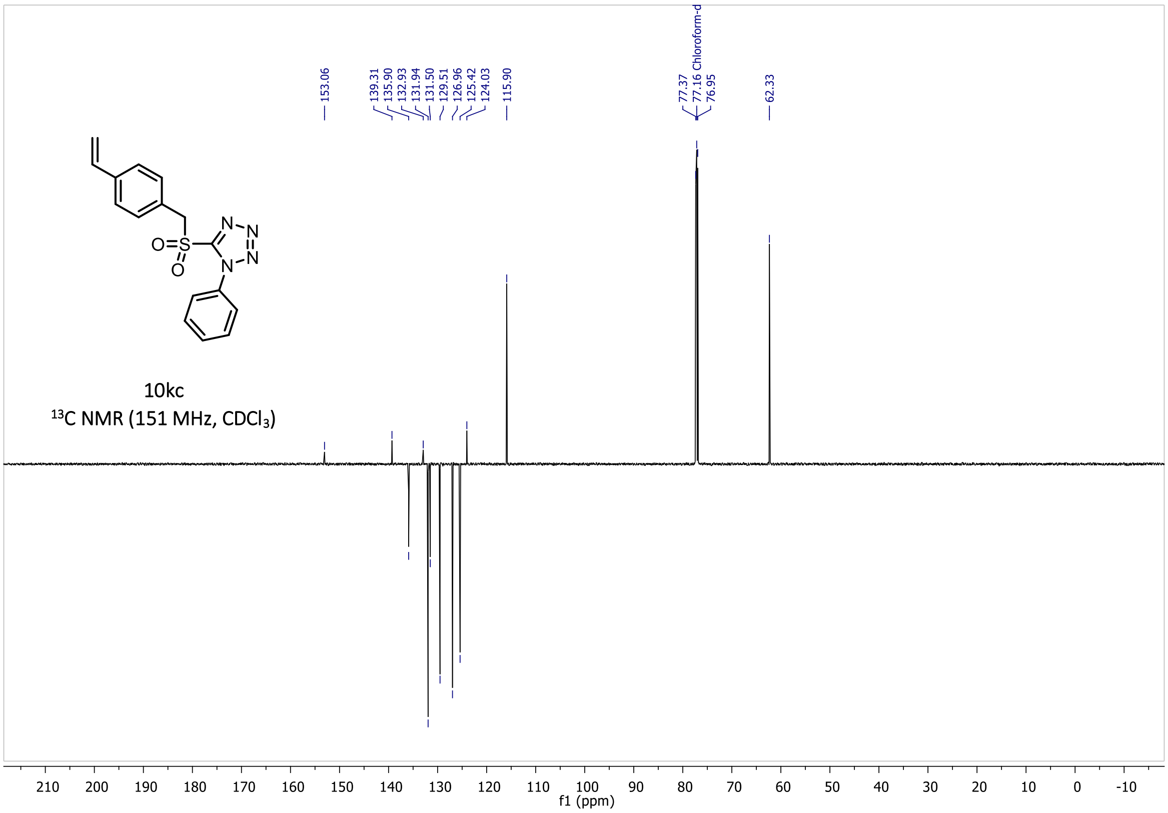

(*E*)-1,2,3-Trimethoxy-5-(4-vinylstyryl)benzene (10kd)

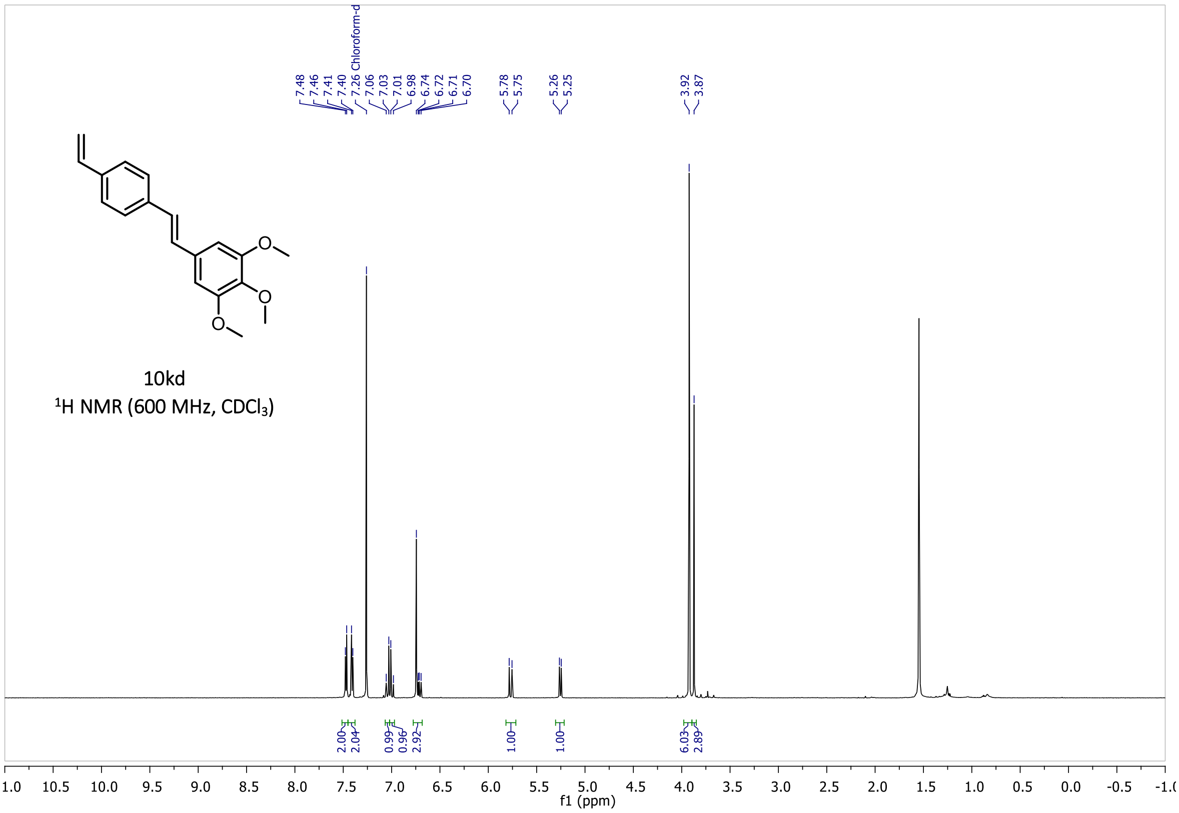

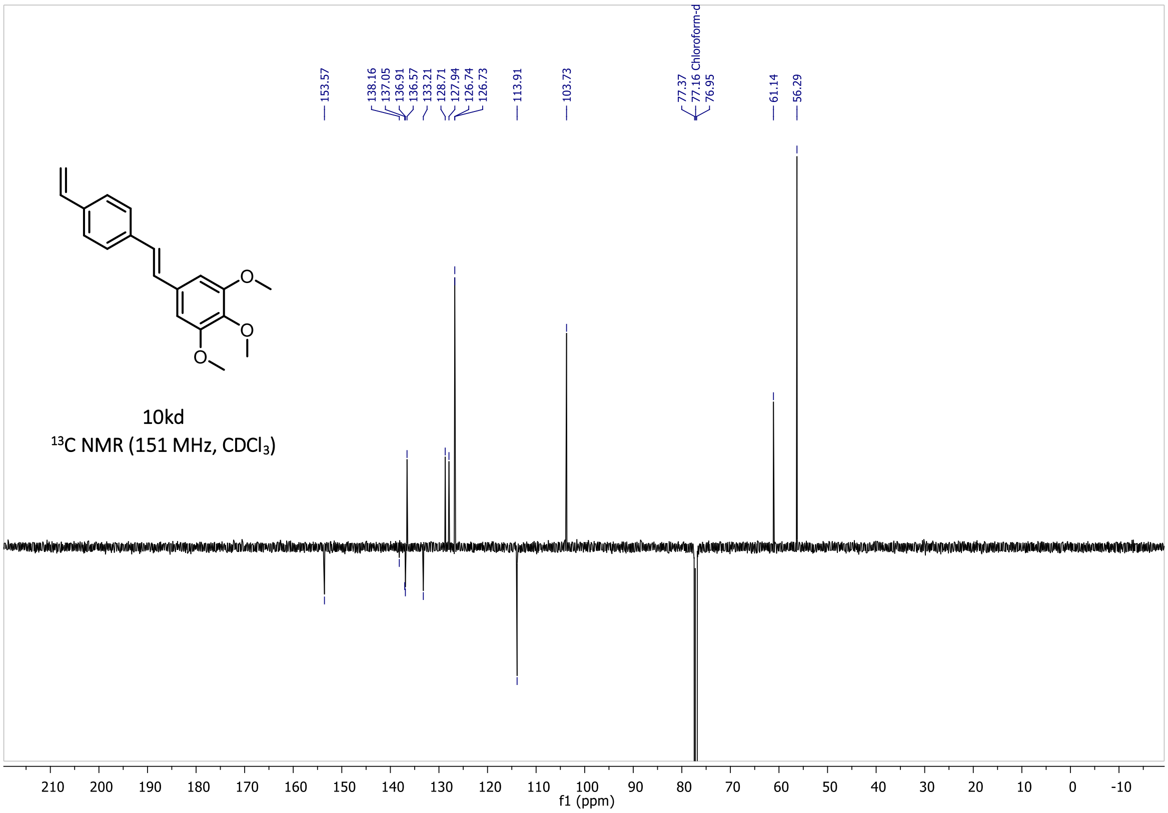

4,4,5,5-Tetramethyl-2-((*E*)-4-((*E*)-3,4,5-trimethoxystyryl)styryl)-1,3,2-dioxaborolane (10k)

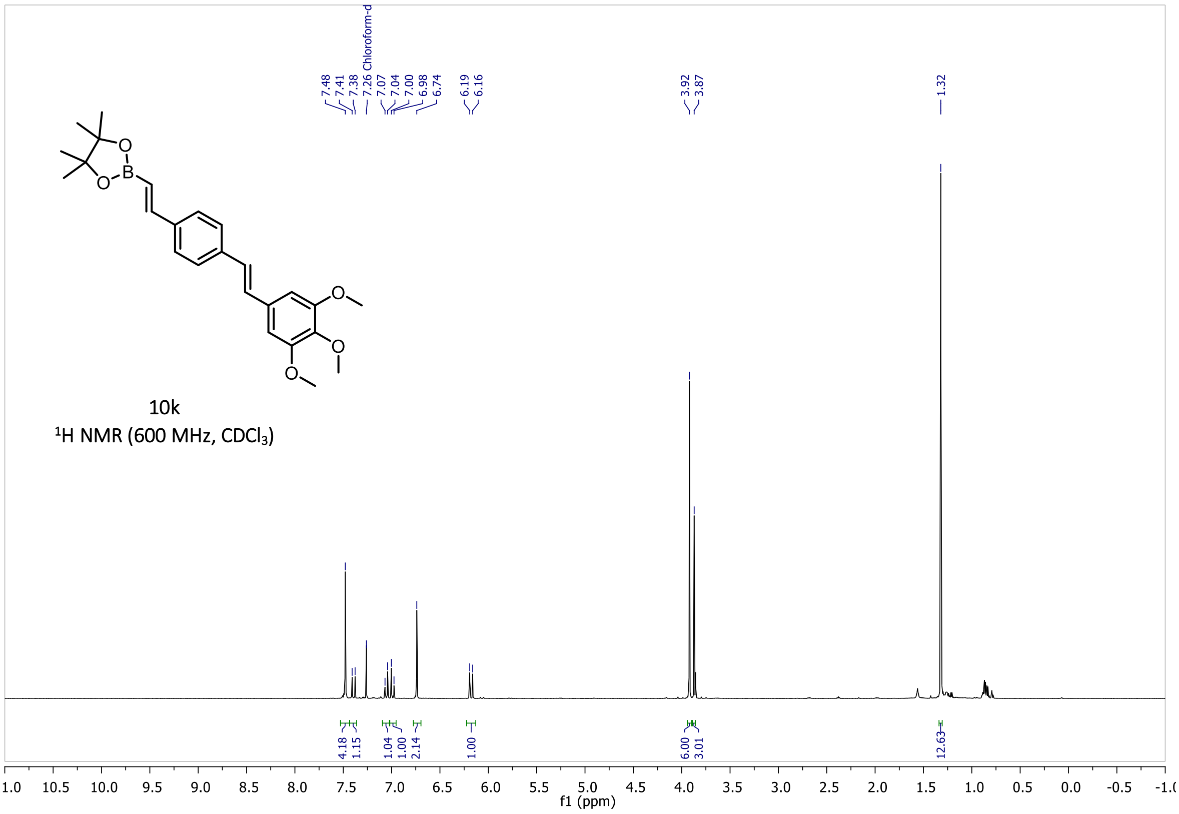

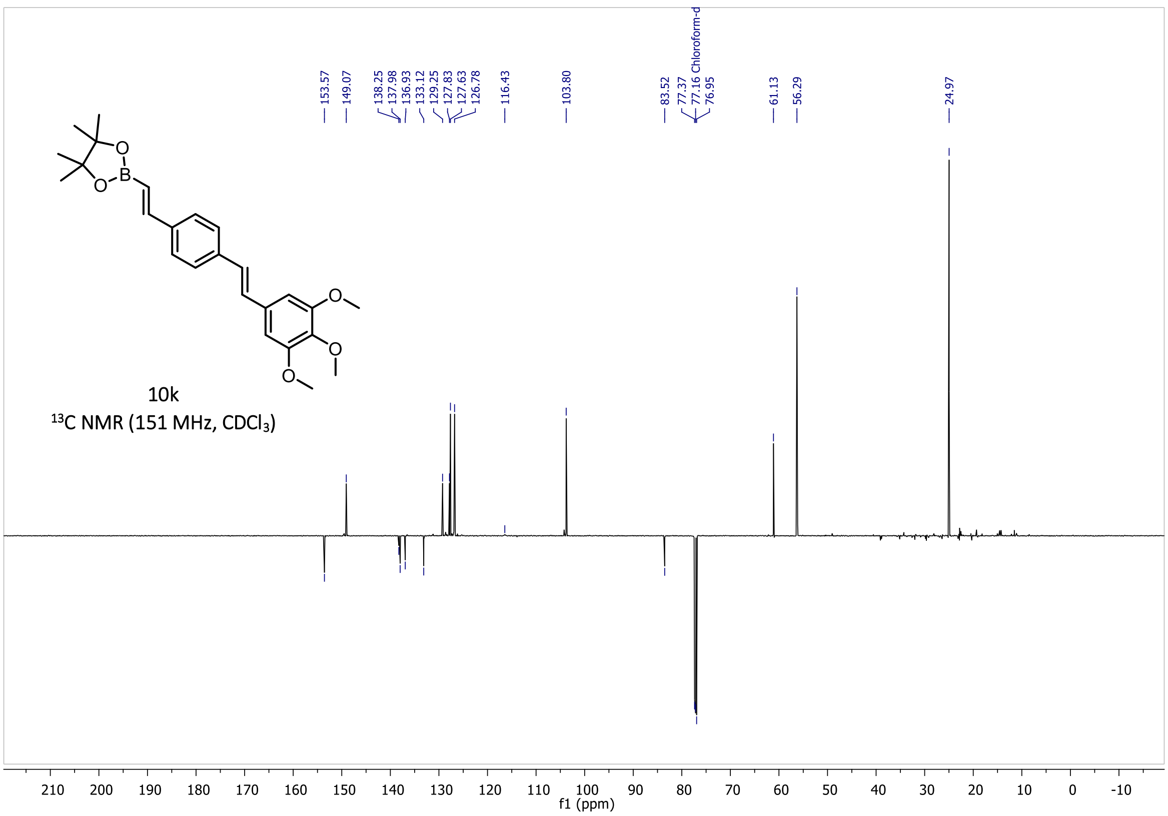

1-Phenethyl-3-phenyl-2-(pyrrolidin-1-yl)-1H-imidazo[1,2-a]pyridin-4-ium trifluoromethanesulfonate (4a)

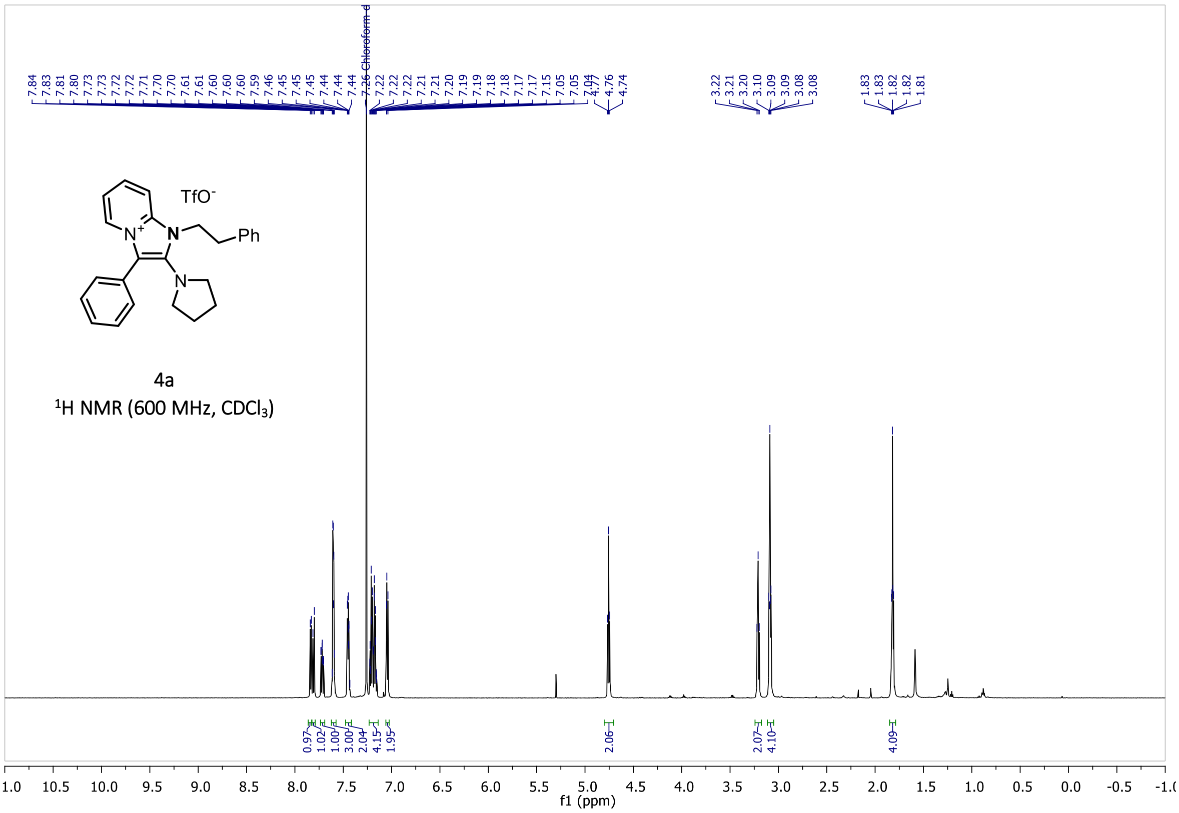

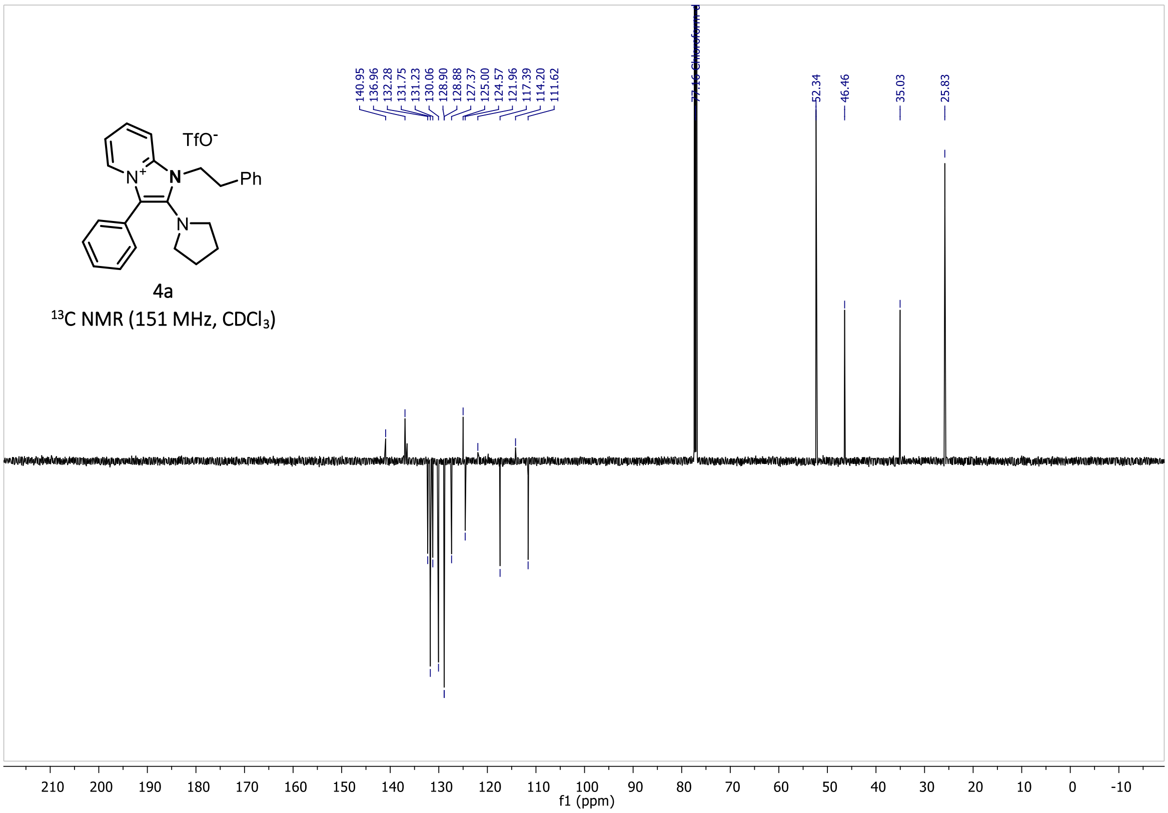
140.95
136.96
132.28
131.75
131.23
130.06
128.90
128.88
127.37
125.00
124.57
121.96
117.39
114.20
111.62
52.34
46.46
35.03
25.83
TfO⁻
N+
N
Ph
N
4a
^{13}C NMR (151 MHz, $CDCl_3$)
f1 (ppm)

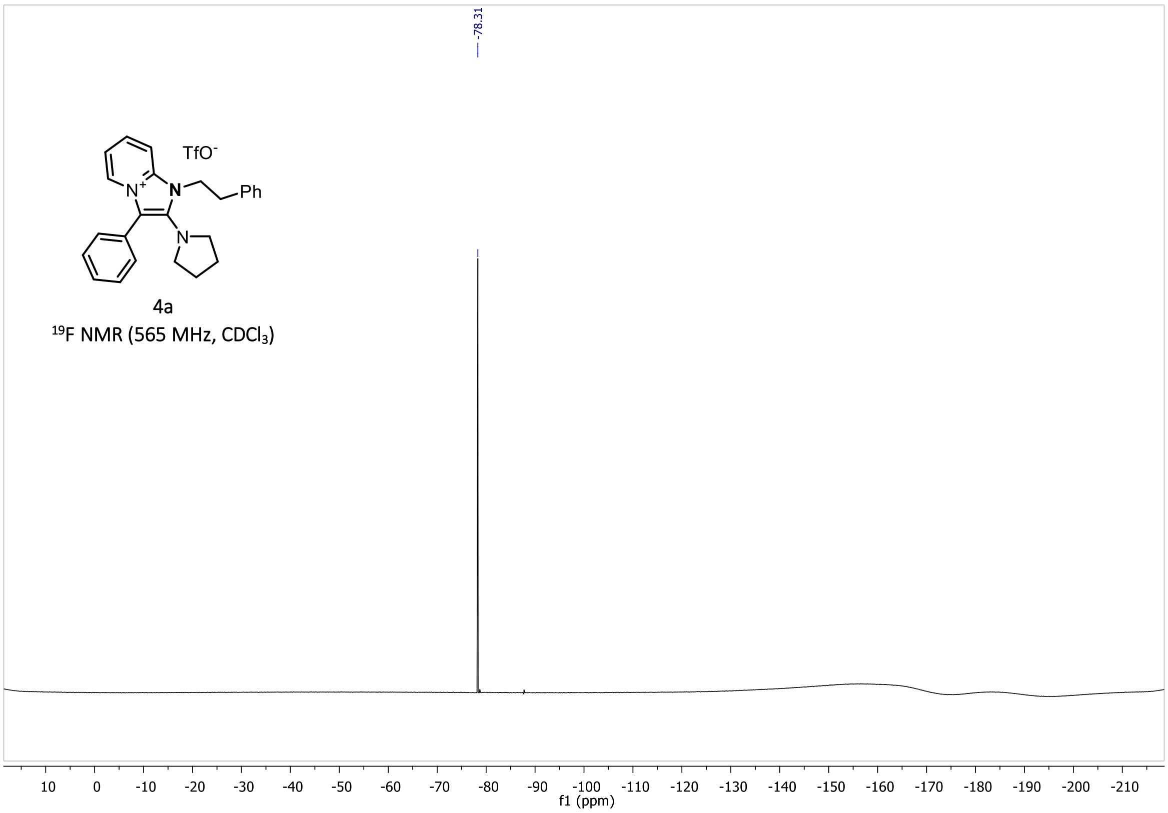
-78.31
TfO⁻
N+
N
Ph
N
4a
^{19}F NMR (565 MHz, $CDCl_3$)
f1 (ppm)

3-(4-Nitrophenyl)-1-phenethyl-2-(pyrrolidin-1-yl)-1H-imidazo[1,2-a]pyridin-4-ium trifluoromethanesulfonate (4b)

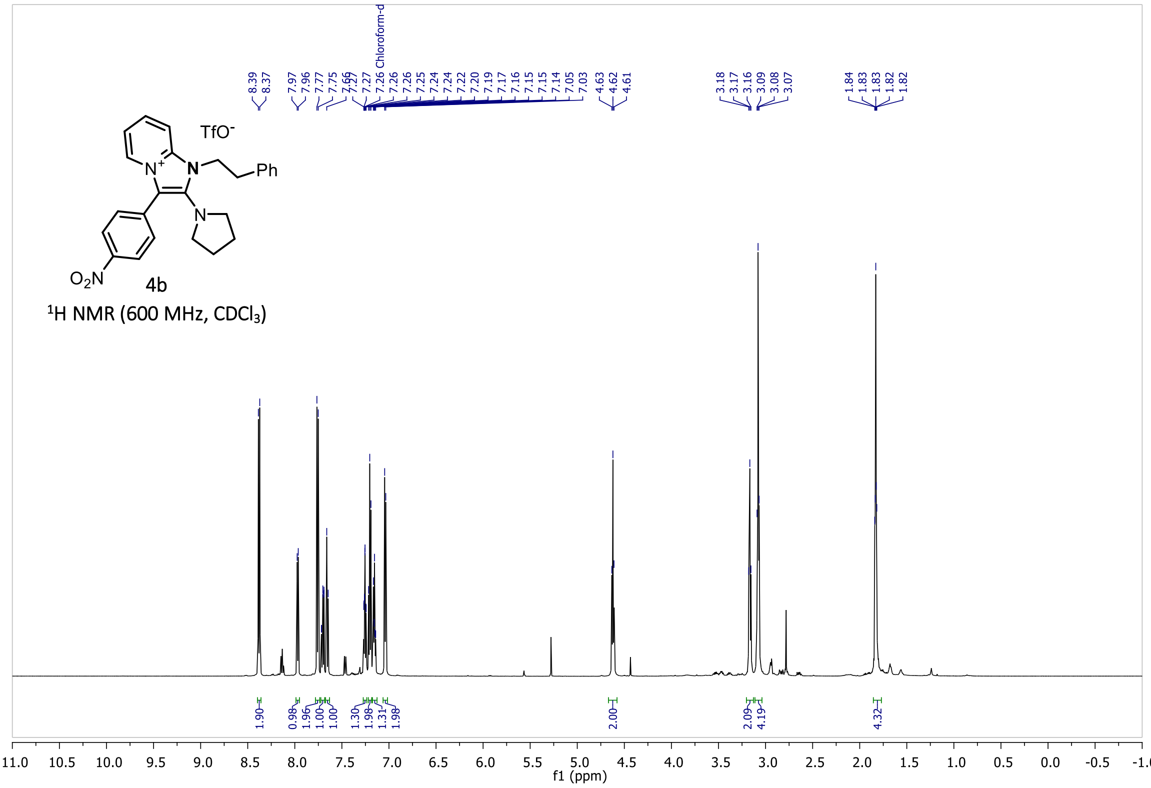

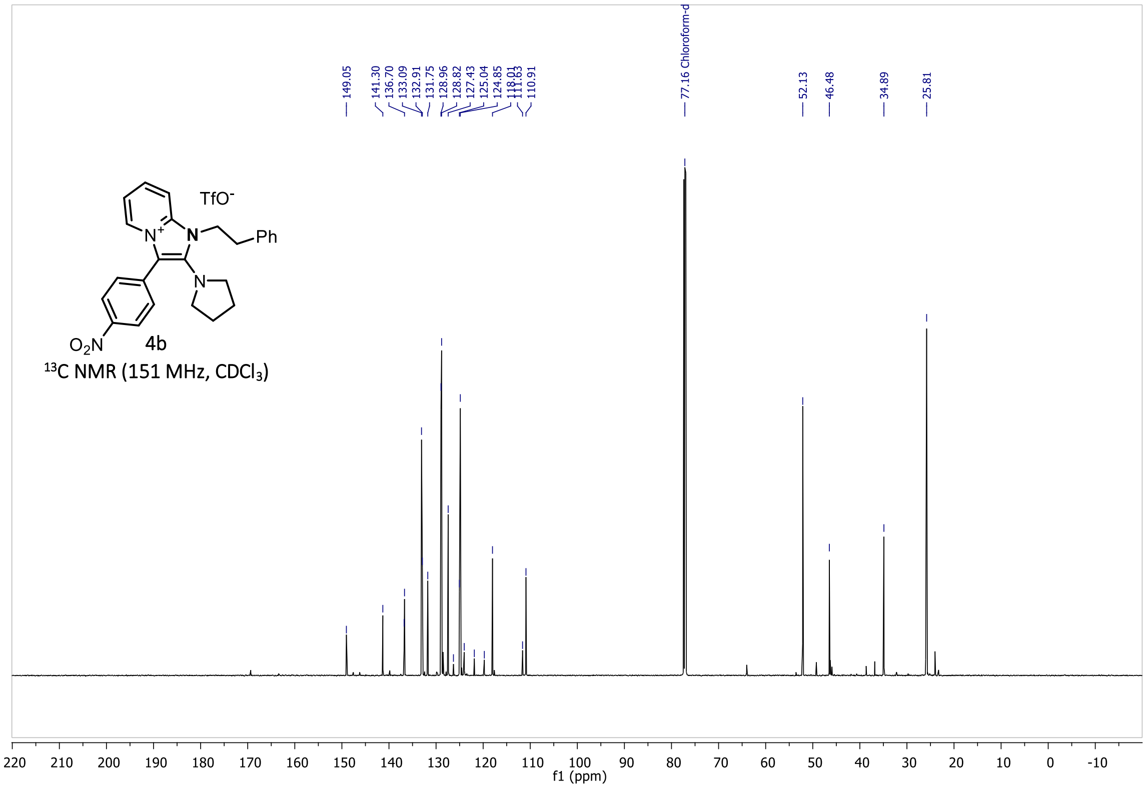

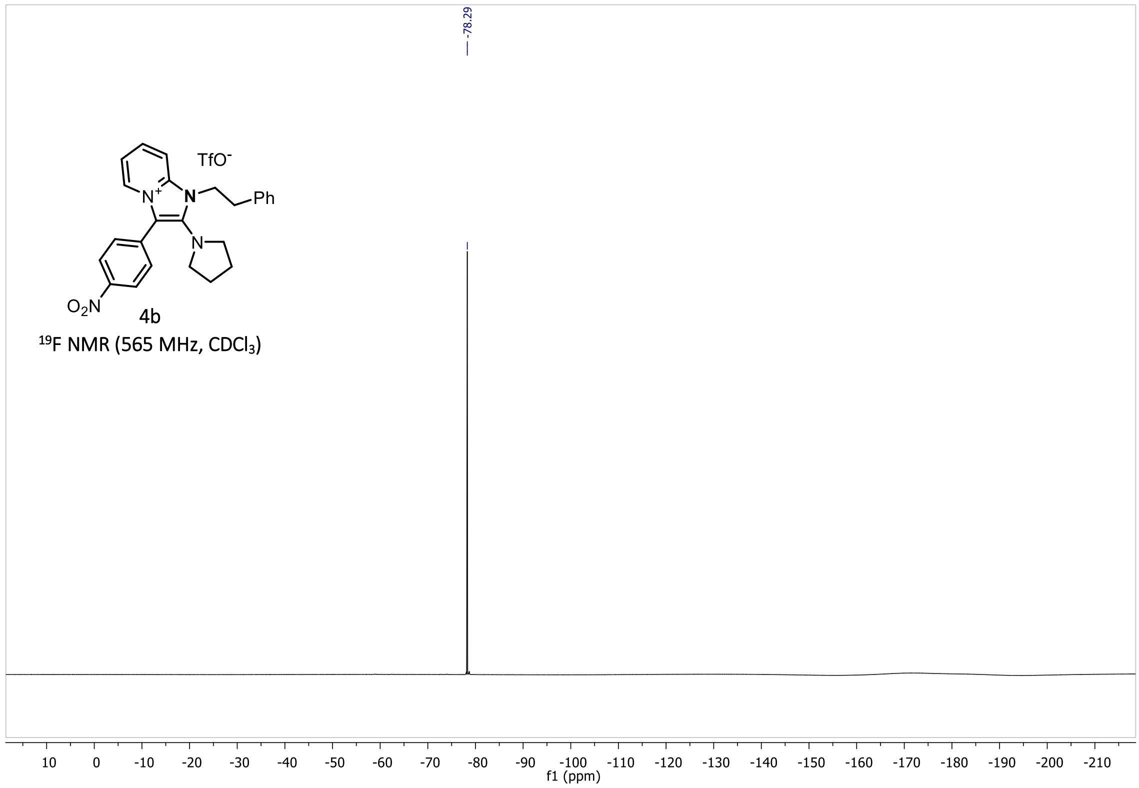

1-Phenethyl-2-(pyrrolidin-1-yl)-3-(4-((trifluoromethyl)thio)phenyl)-1H-imidazo[1,2-a]pyridin-4-ium trifluoromethanesulfonate (4c)

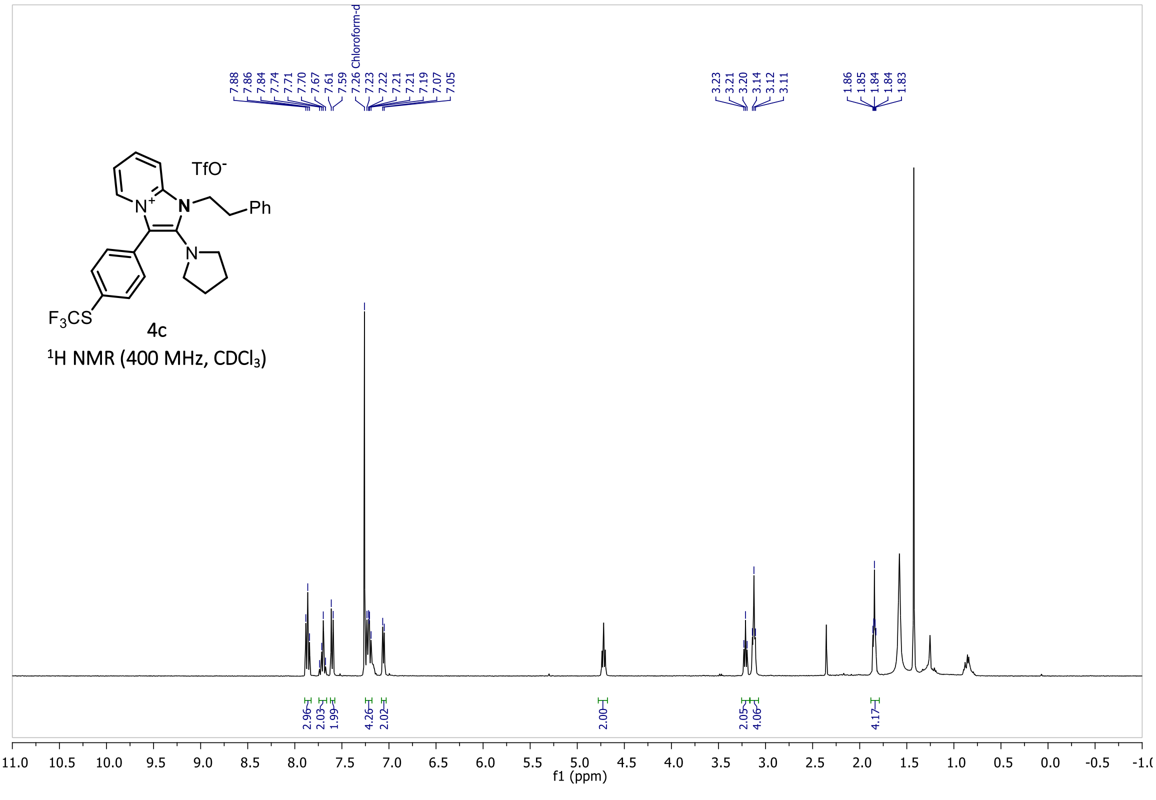

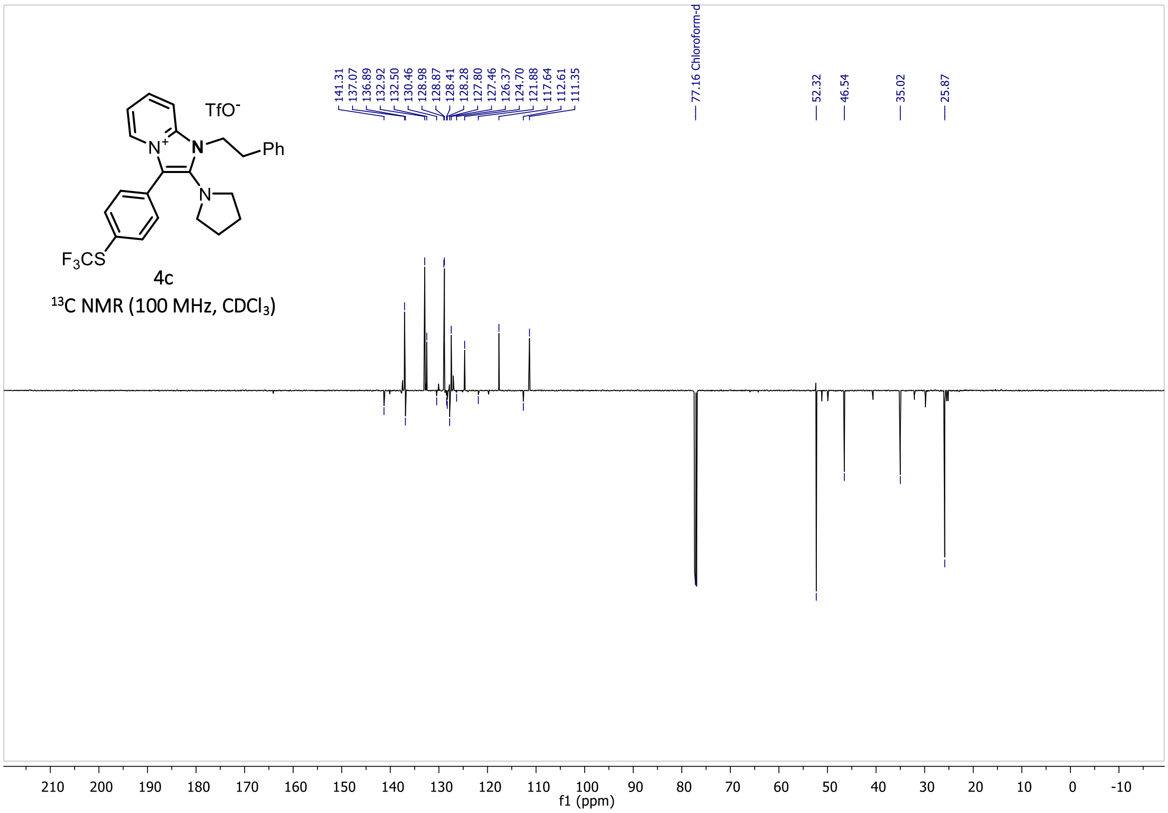

141.31
137.07
136.89
132.92
132.50
130.46
128.98
128.87
128.41
128.28
127.80
127.46
126.37
124.70
121.88
117.64
112.61
111.35
77.16 Chloroform-d
52.32
46.54
35.02
25.87
TfO-
Ph
N+
N
N
F3CS
4c
13C NMR (100 MHz, CDCl3)
f1 (ppm)

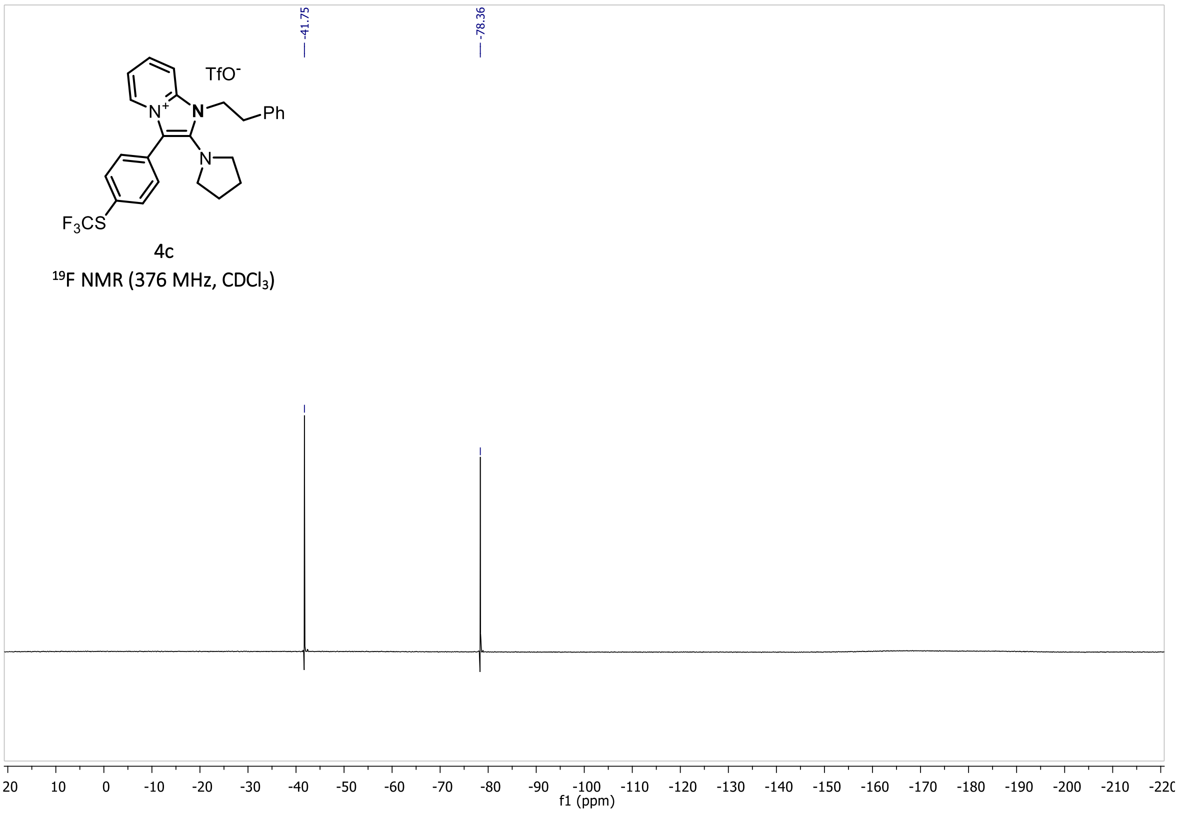

-41.75
-78.36
TfO-
Ph
N+
N
N
F3CS
4c
19F NMR (376 MHz, CDCl3)
f1 (ppm)

3-(4-Methoxyphenyl)-1-phenethyl-2-(pyrrolidin-1-yl)-1H-imidazo[1,2-a]pyridin-4-ium trifluoromethanesulfonate (4d)

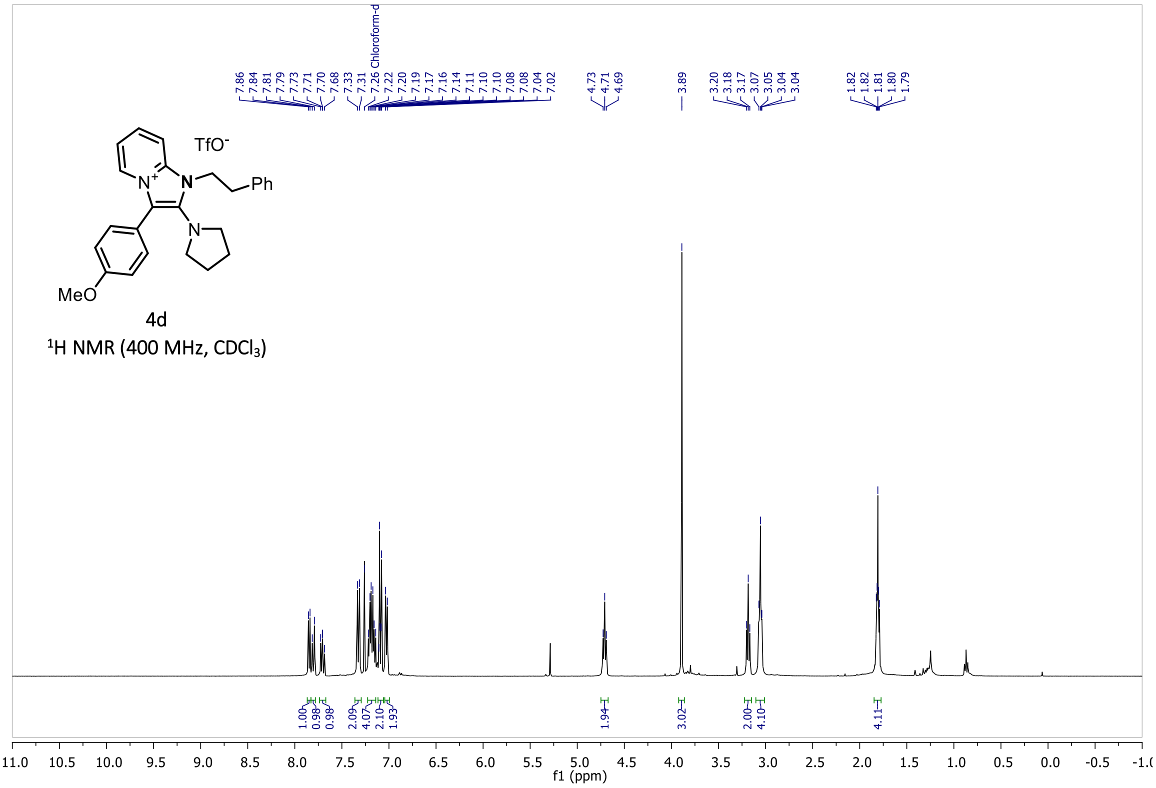

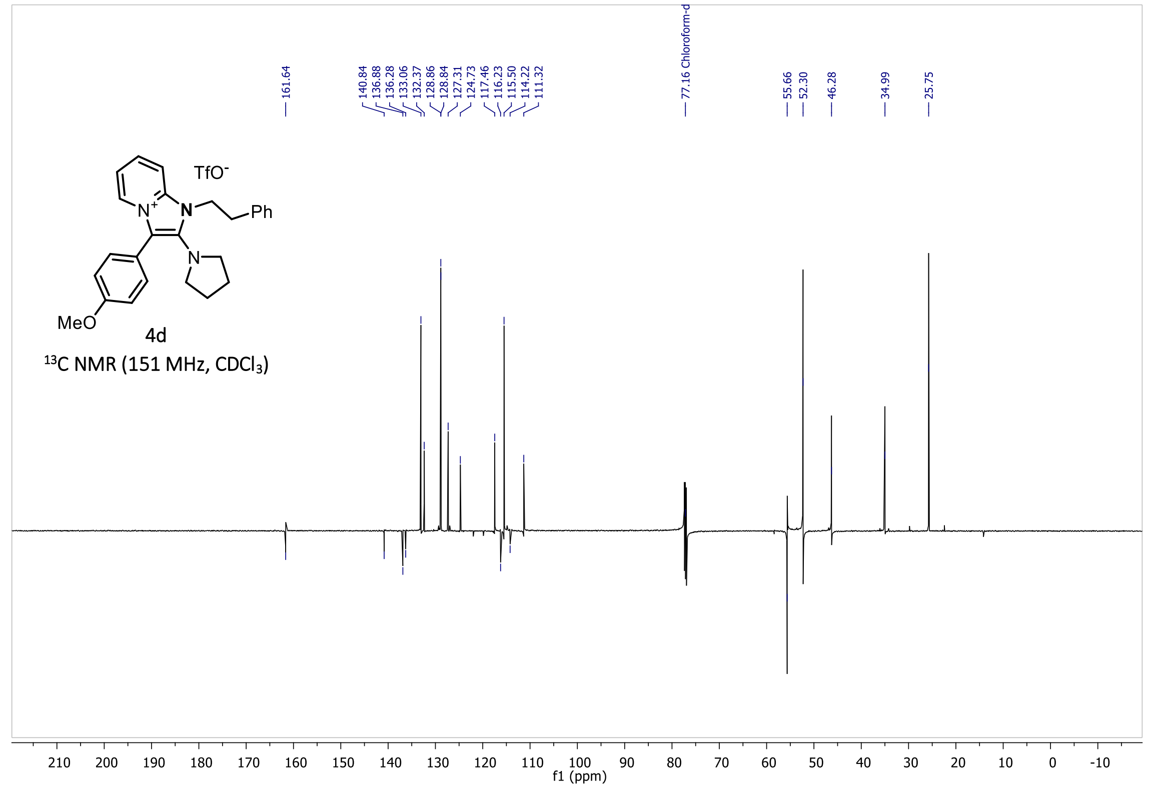

3-(2-Chlorophenyl)-1-phenethyl-2-(pyrrolidin-1-yl)-1H-imidazo[1,2-a]pyridin-4-ium trifluoromethanesulfonate (4e)

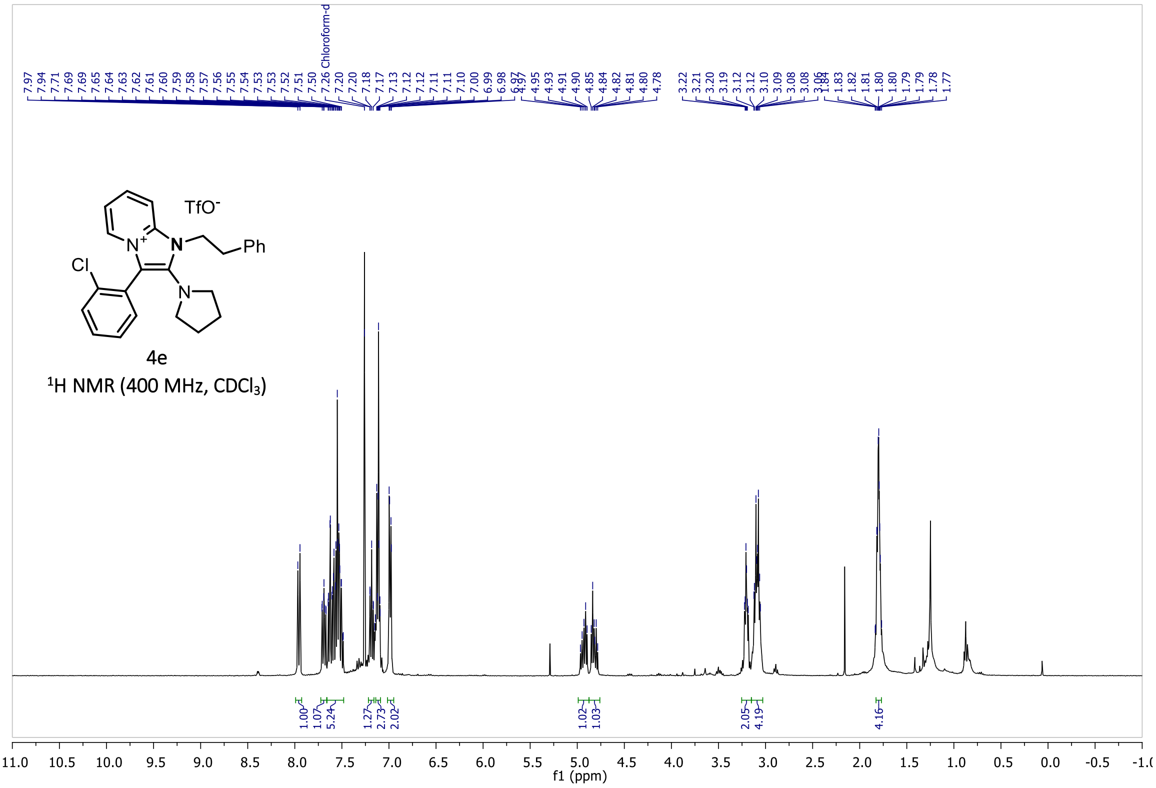

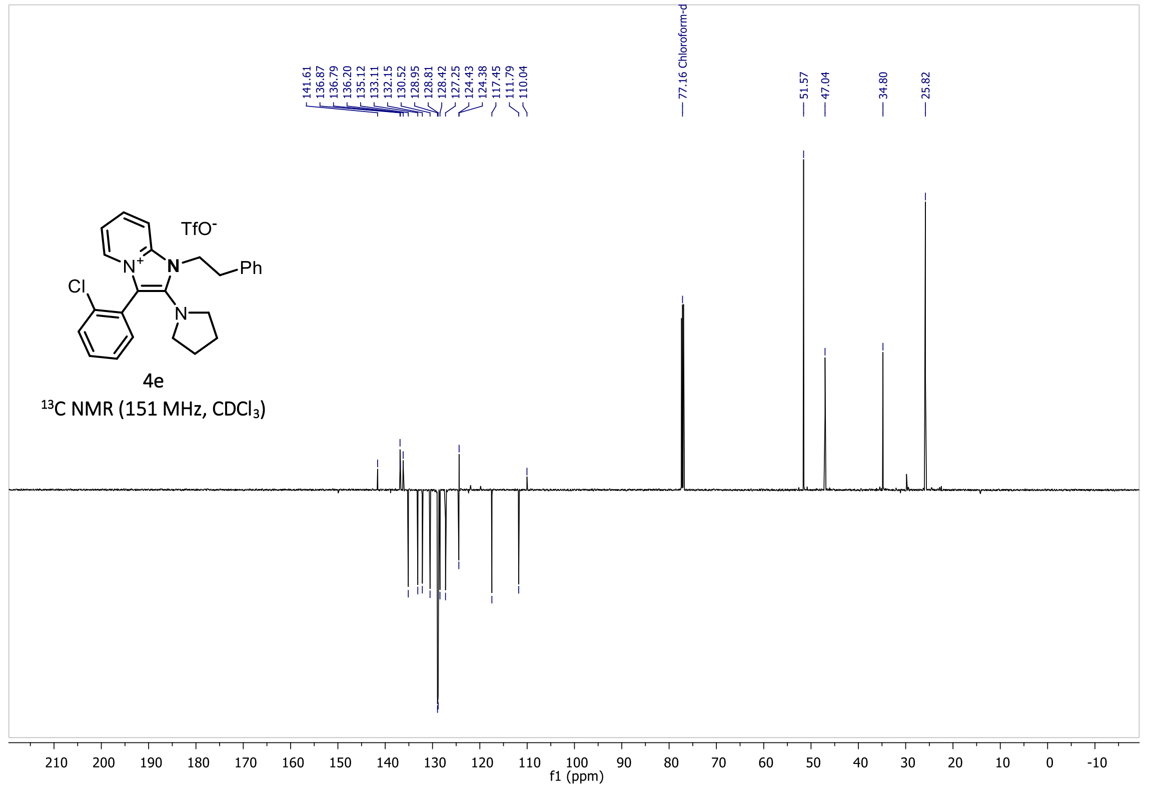

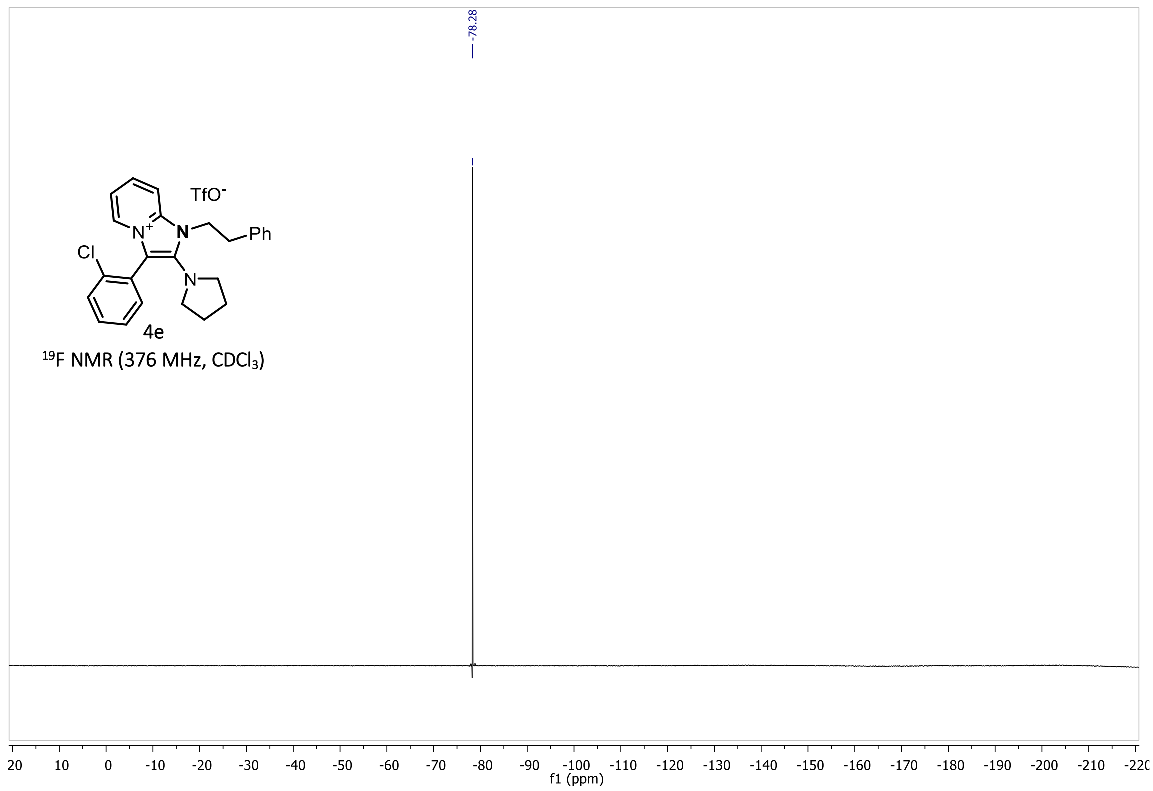

3-(4-Iodophenyl)-1-phenethyl-2-(pyrrolidin-1-yl)-1H-imidazo[1,2-a]pyridin-4-ium trifluoromethanesulfonate (4f)

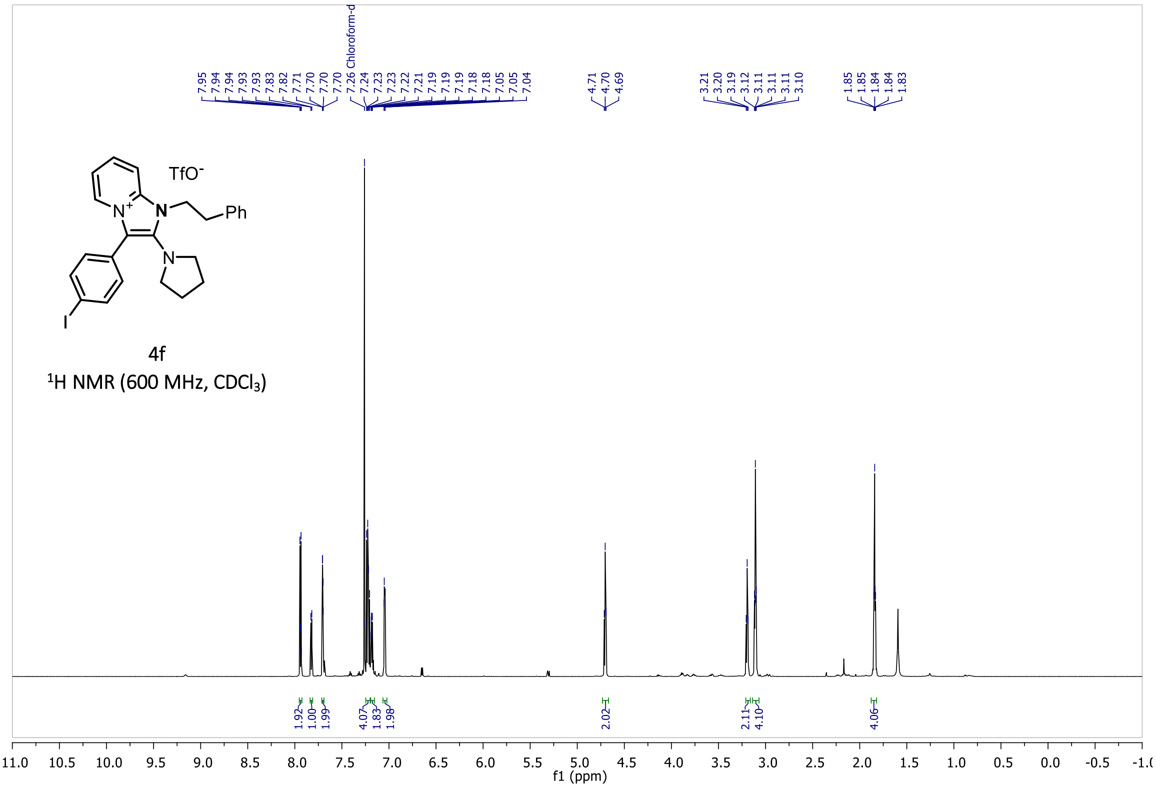

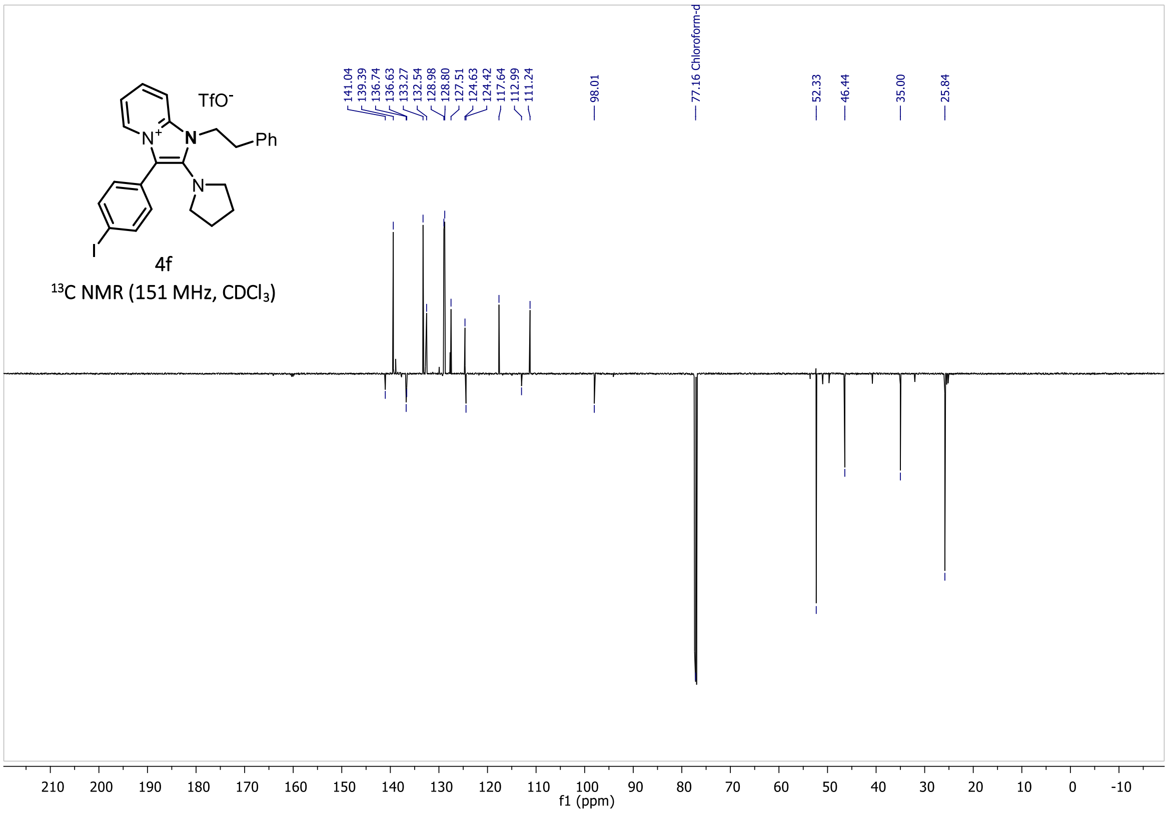
TfO⁻
Ph
N
N
I
4f
13C NMR (151 MHz, CDCl3)
141.04
139.39
136.74
136.63
133.27
132.54
128.98
128.80
127.51
124.63
124.42
117.64
112.99
111.24
98.01
77.16 Chloroform-d
52.33
46.44
35.00
25.84
f1 (ppm)

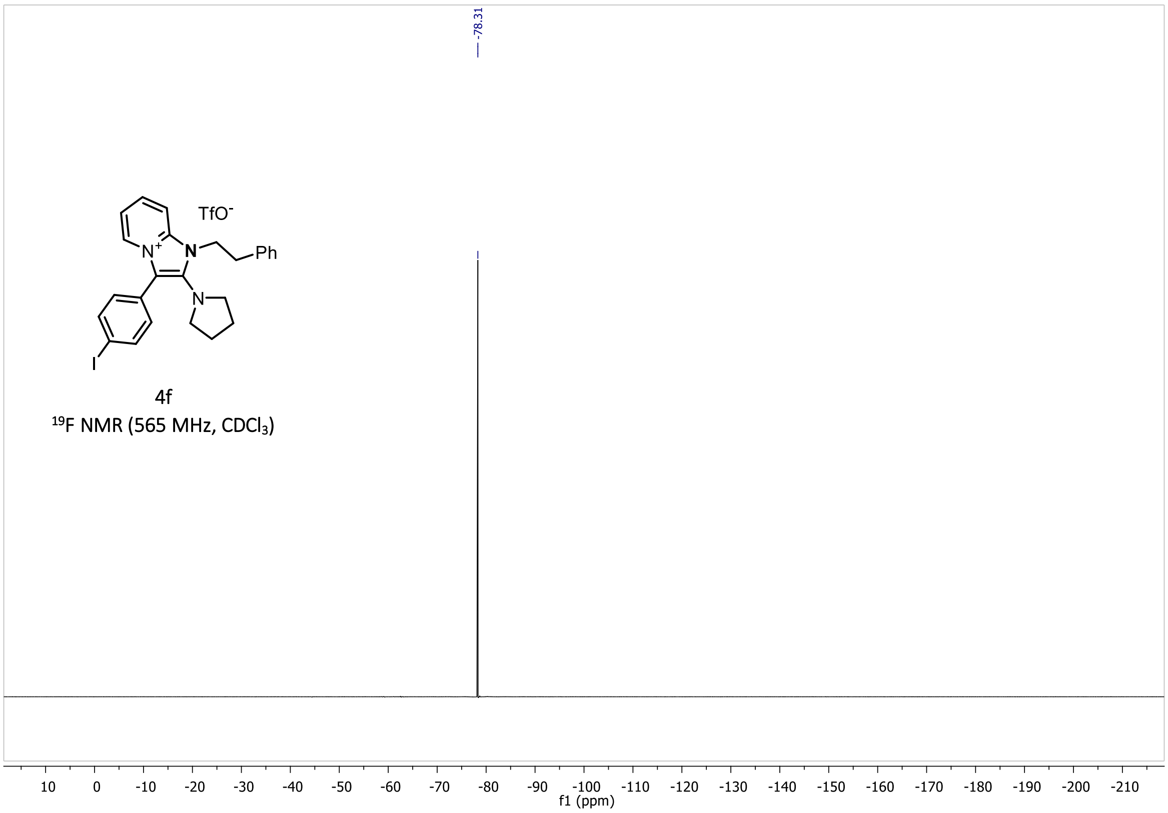
-78.31
TfO⁻
Ph
N
N
I
4f
19F NMR (565 MHz, CDCl3)
f1 (ppm)

1-Phenethyl-3-phenyl-2-(piperidin-1-yl)-1H-imidazo[1,2-a]pyridin-4-ium trifluoromethanesulfonate (4g)

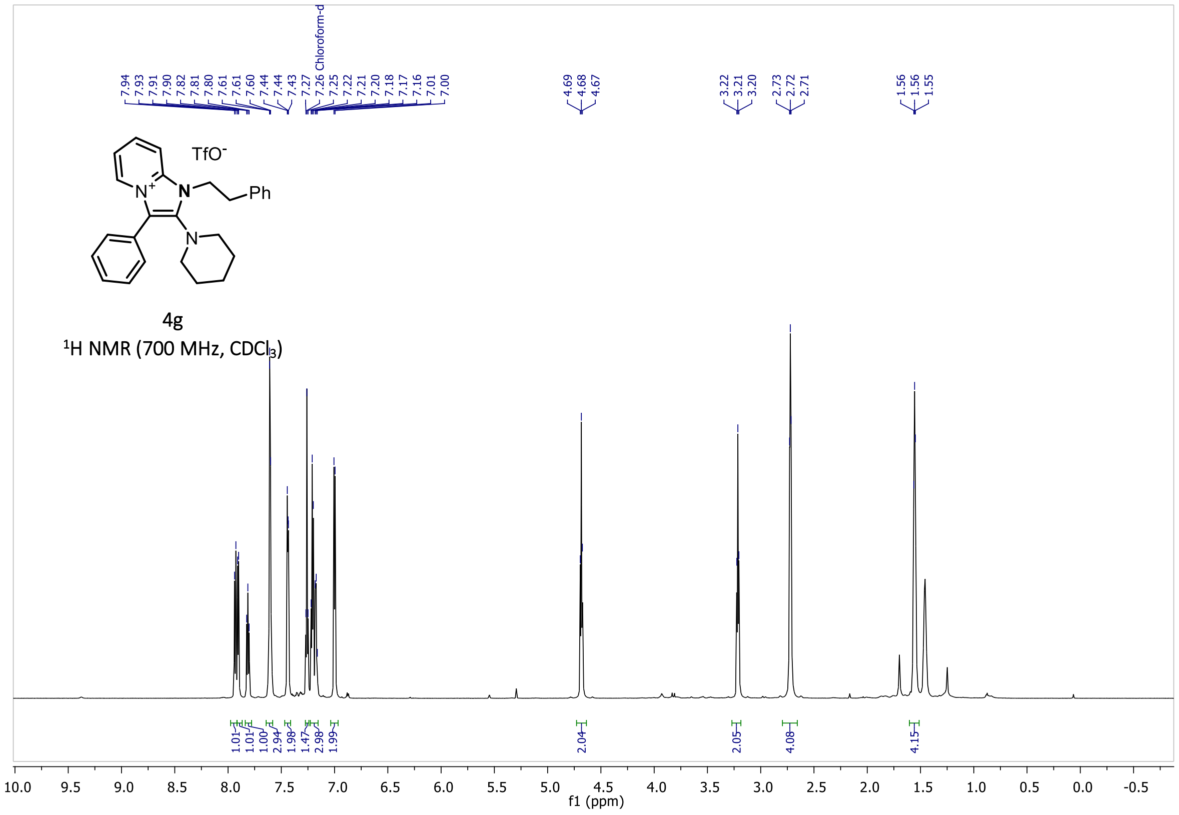

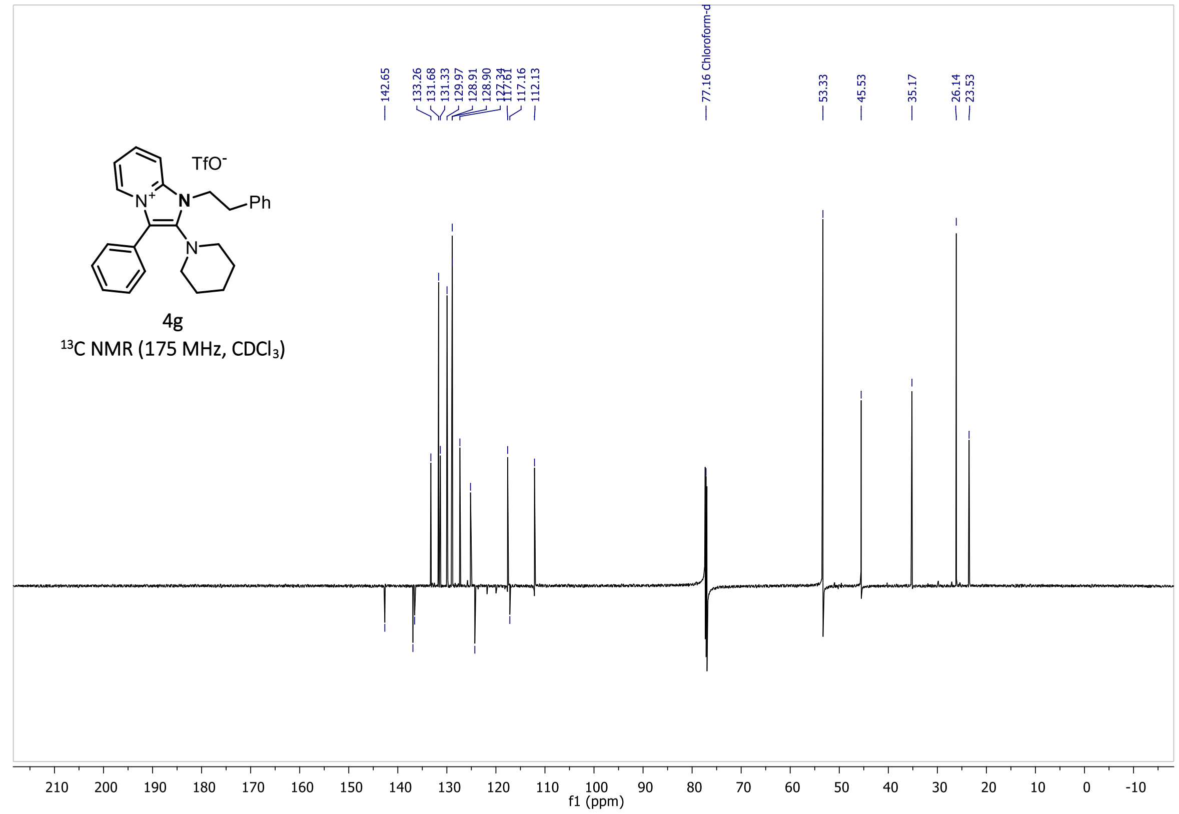

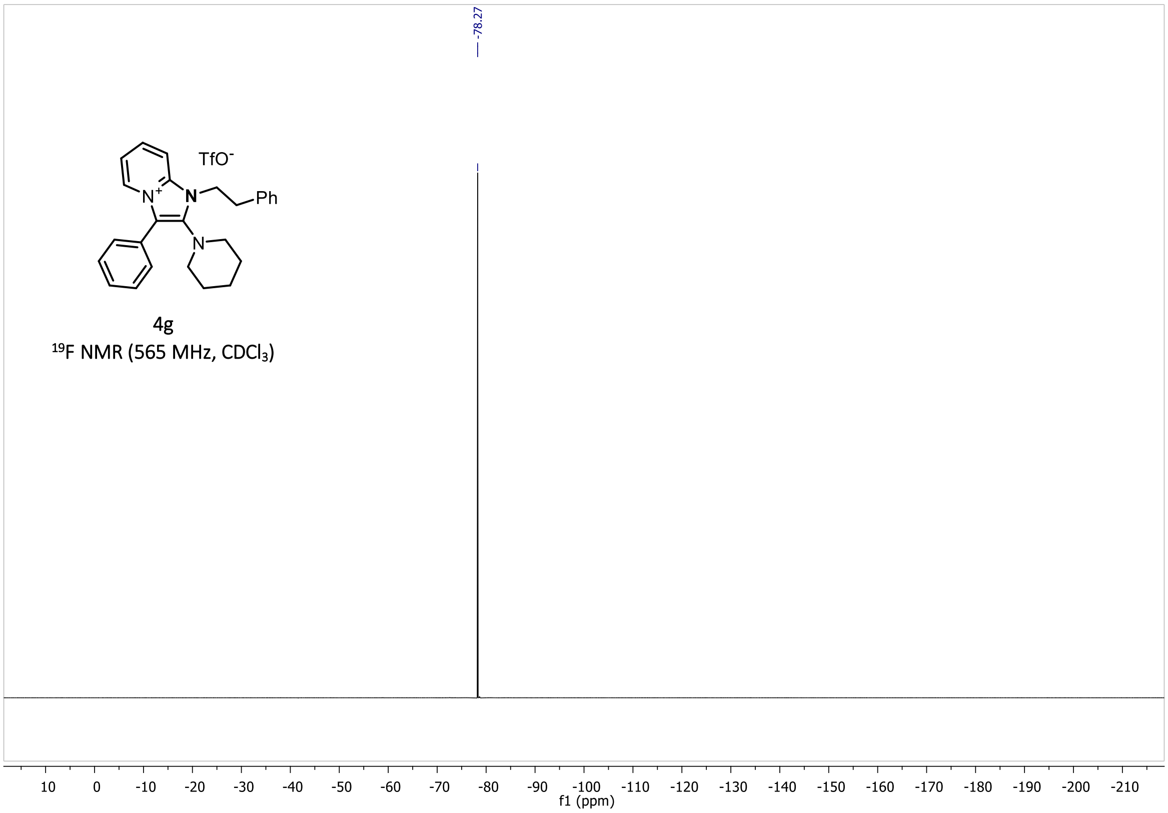

2-(Diethylamino)-1-phenethyl-3-phenyl-1H-imidazo[1,2-a]pyridin-4-ium trifluoromethanesulfonate (4h)

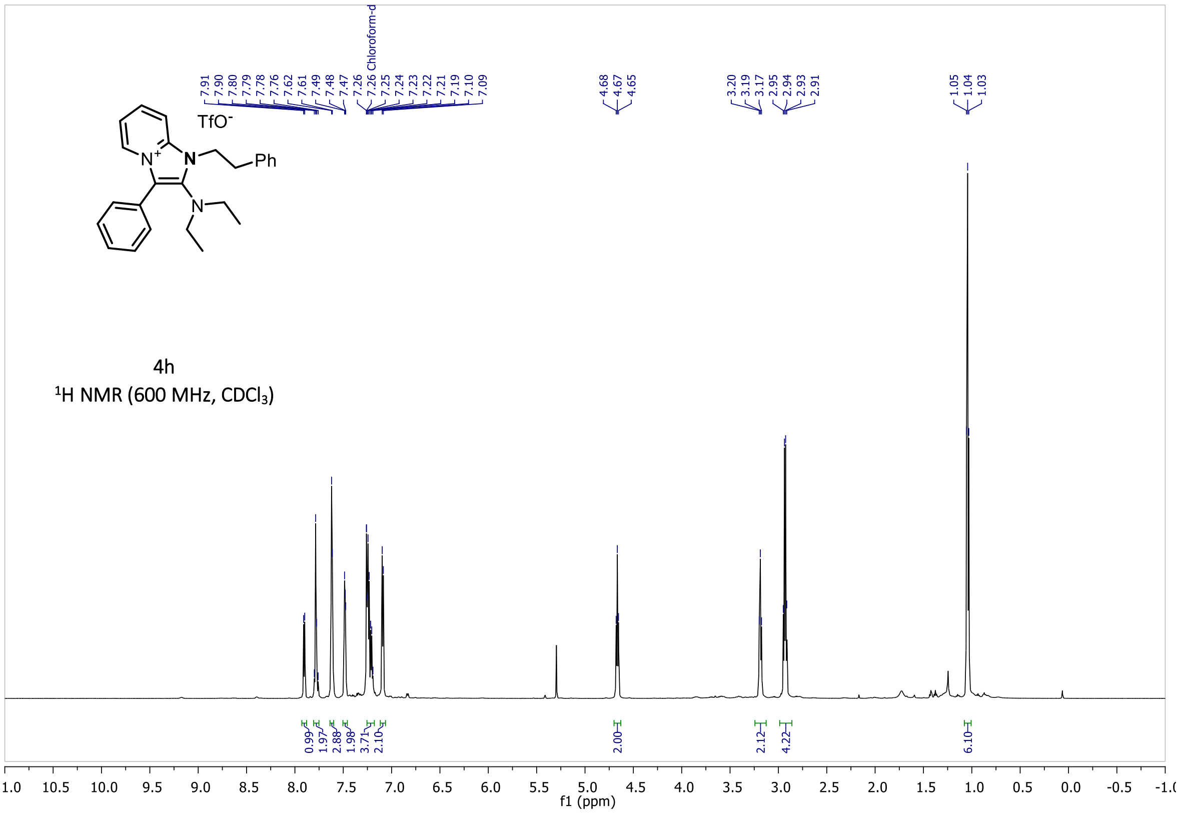

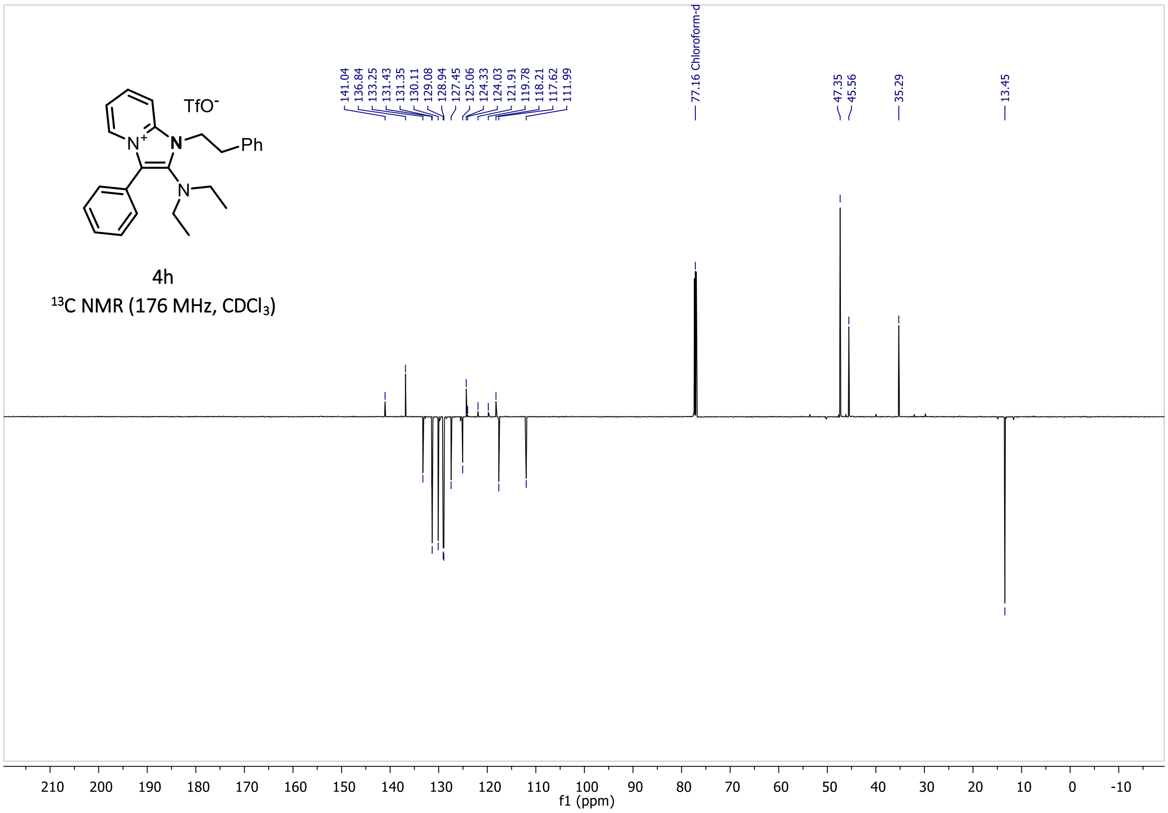

4h

^{13}C NMR (176 MHz, $CDCl_3$)

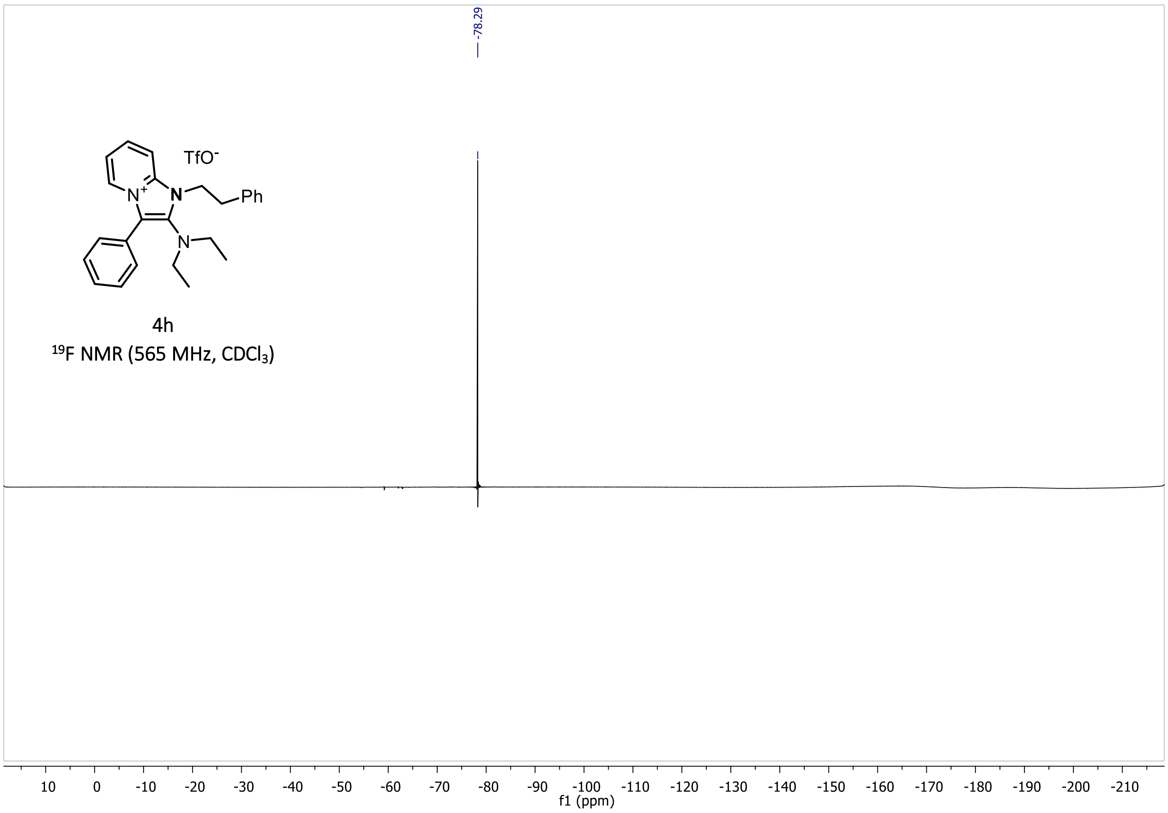

4h

^{19}F NMR (565 MHz, $CDCl_3$)

2-(Azetidin-1-yl)-1-phenethyl-3-phenyl-1H-imidazo[1,2-a]pyridin-4-ium trifluoroacetate (4i)

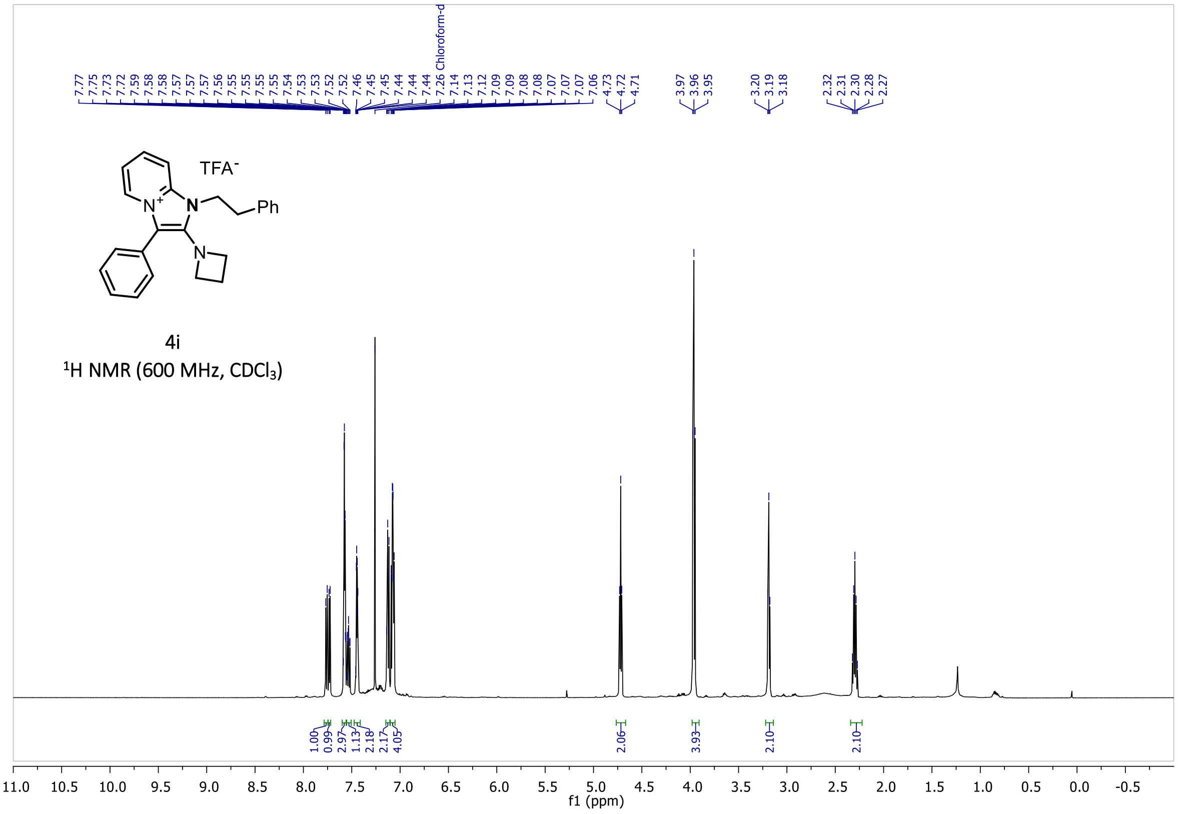

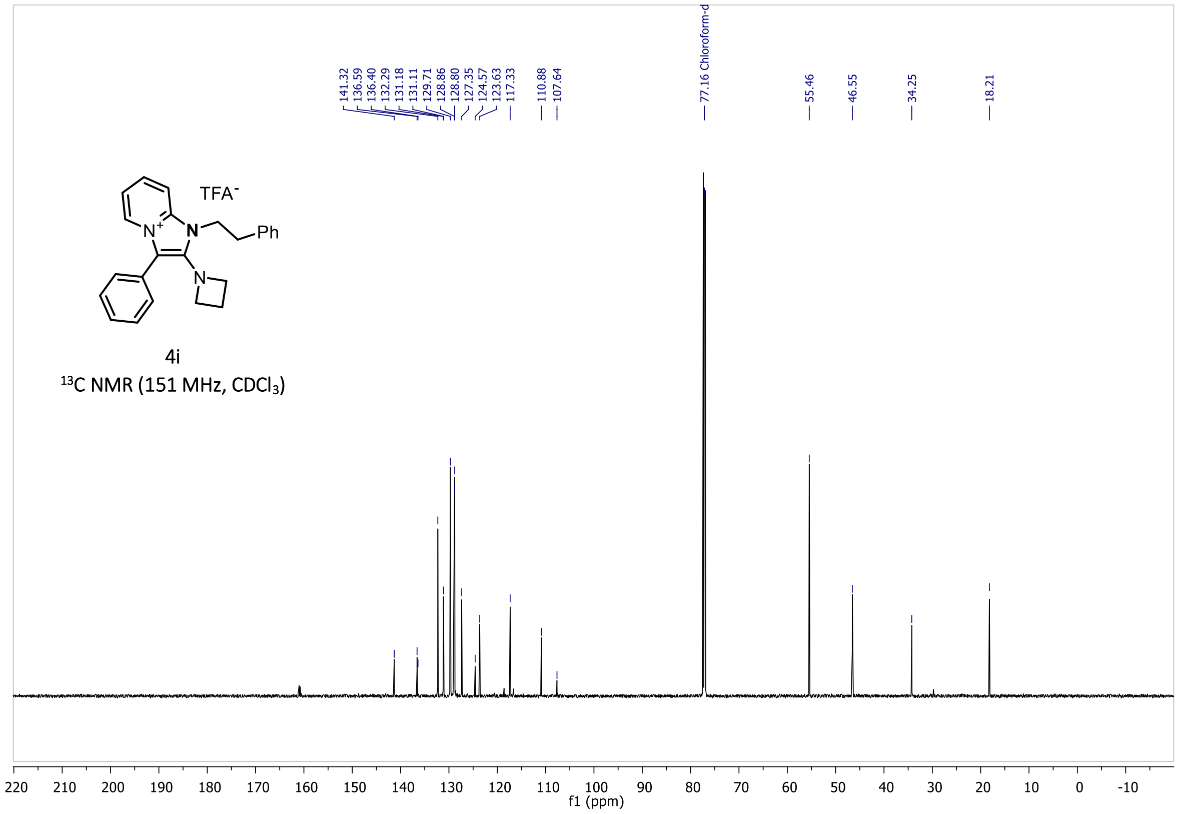

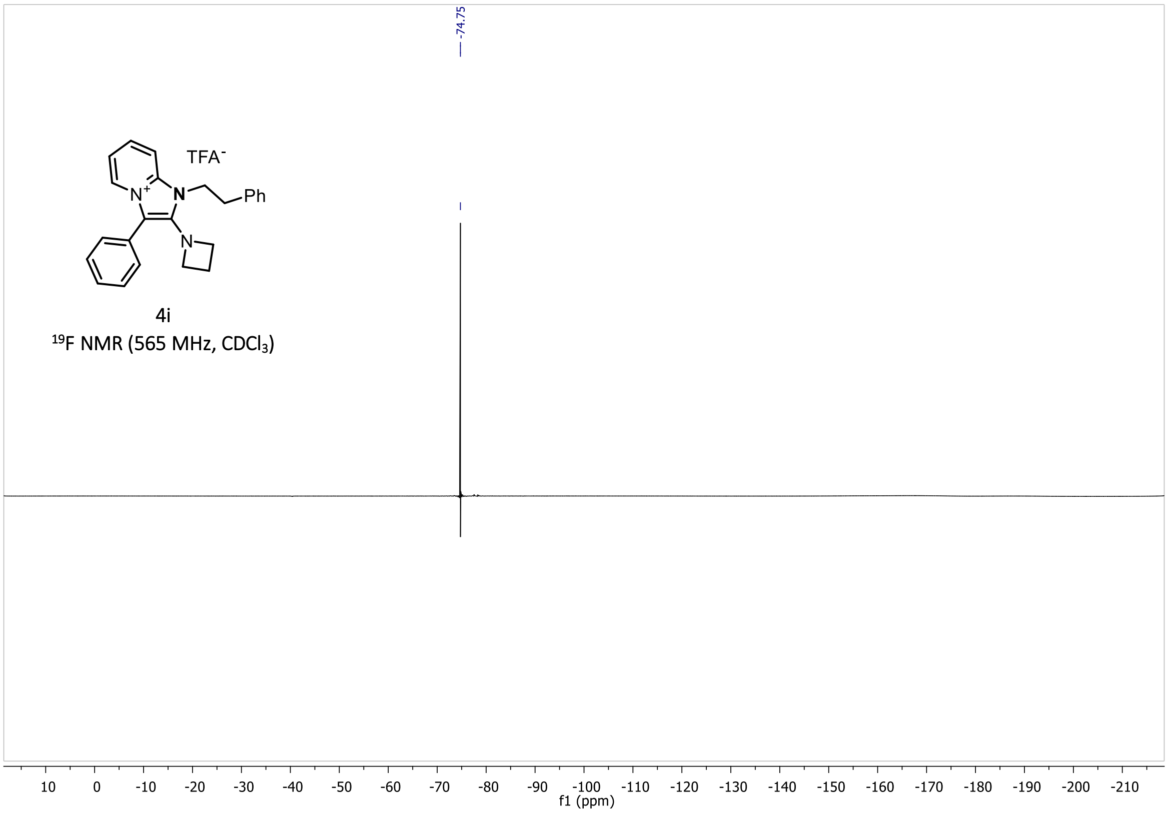

1-Heptyl-3-phenyl-2-(pyrrolidin-1-yl)-1H-imidazo[1,2-a]pyridin-4-ium trifluoromethanesulfonate (4j)

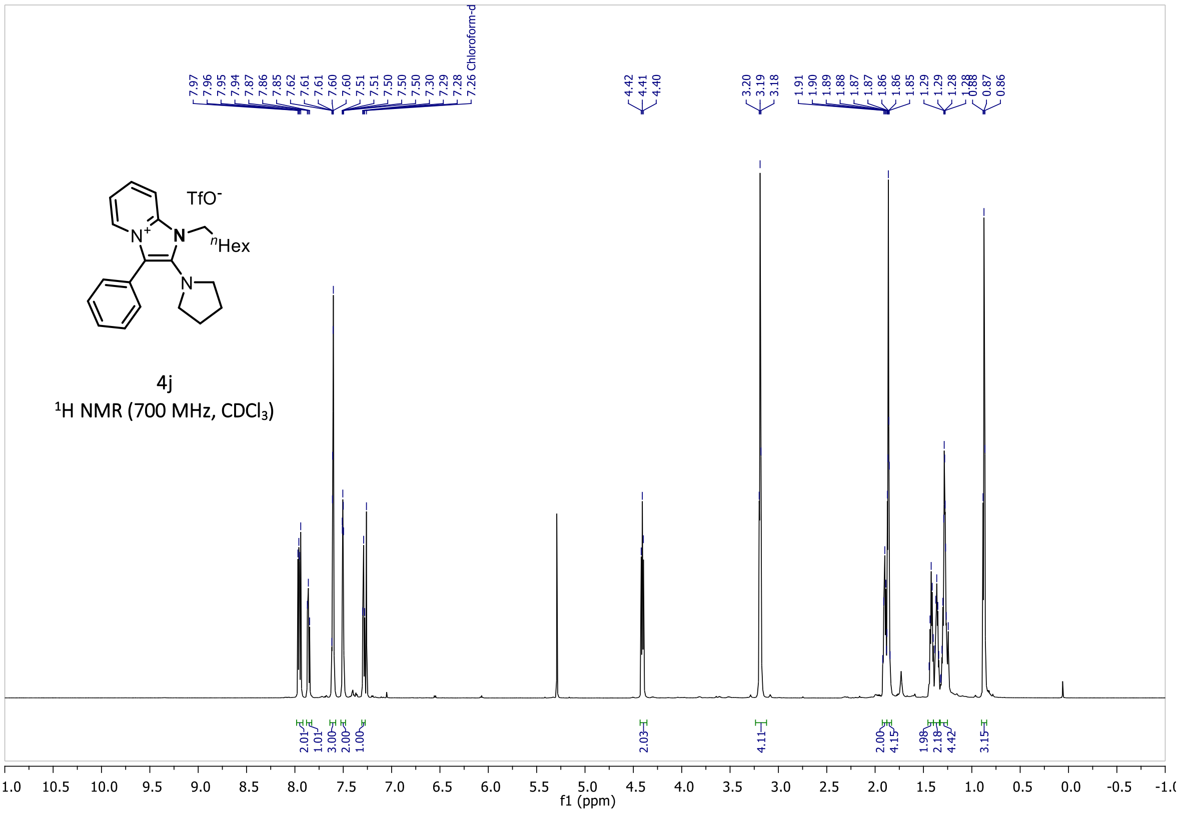

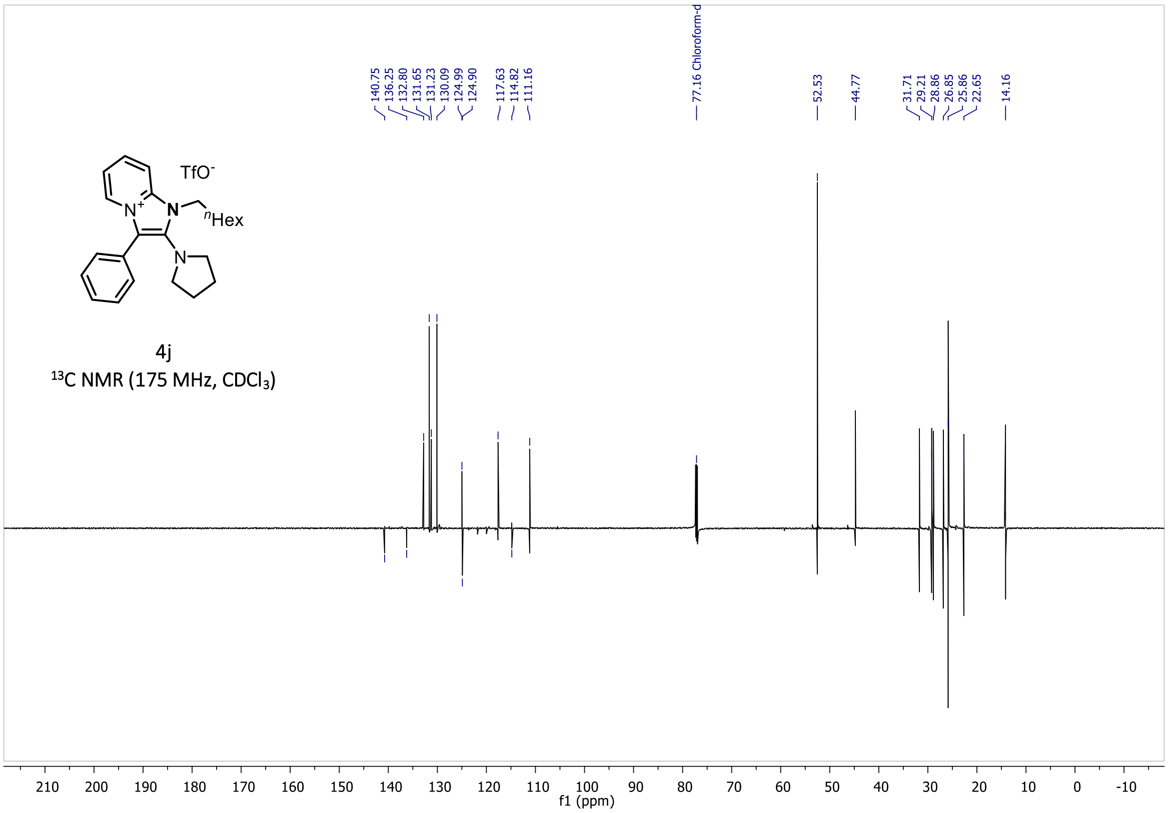

TfO-
nHex
4j
13C NMR (175 MHz, CDCl3)
140.75
136.25
132.80
131.65
131.23
130.09
124.99
124.90
117.63
114.82
111.16
77.16 Chloroform-d
52.53
44.77
31.71
29.21
28.86
26.85
25.86
22.65
14.16
f1 (ppm)

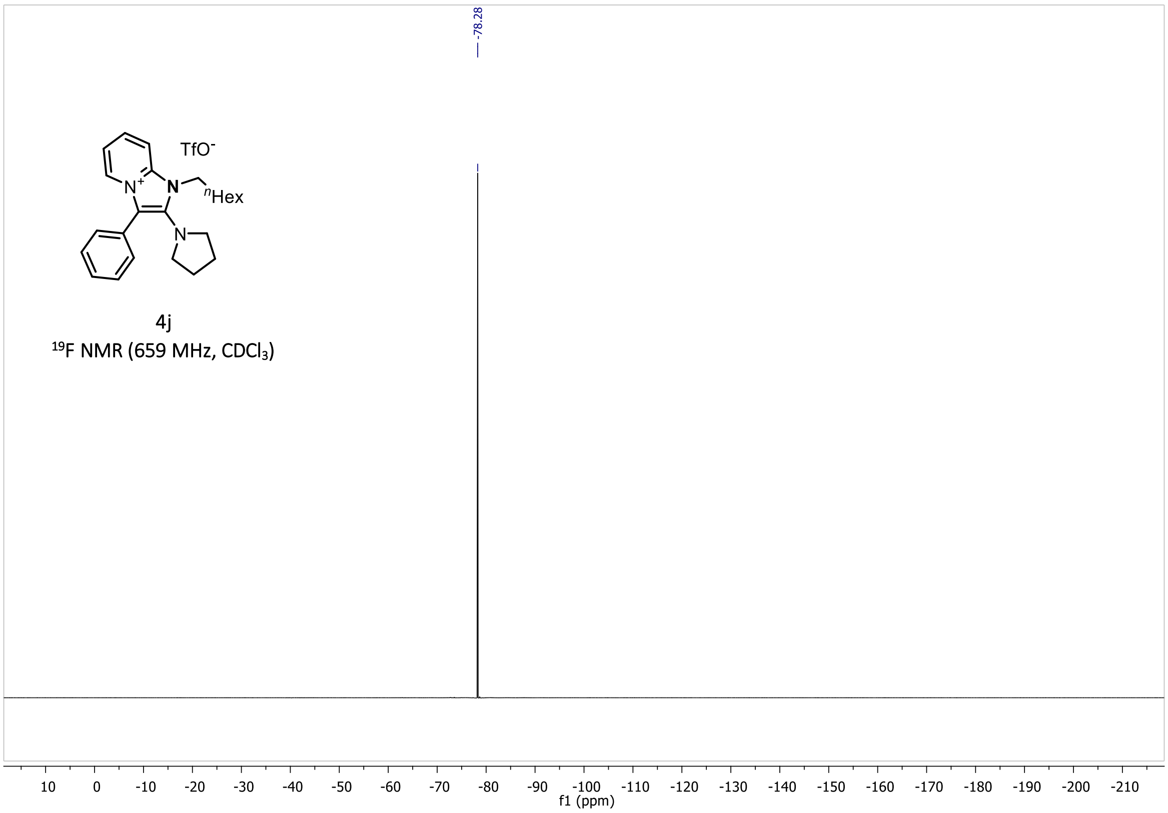

TfO-
nHex
4j
19F NMR (659 MHz, CDCl3)
-78.28
f1 (ppm)

1-(Cyclohexylmethyl)-3-phenyl-2-(pyrrolidin-1-yl)-1H-imidazo[1,2-a]pyridin-4-ium trifluoromethanesulfonate (4k)

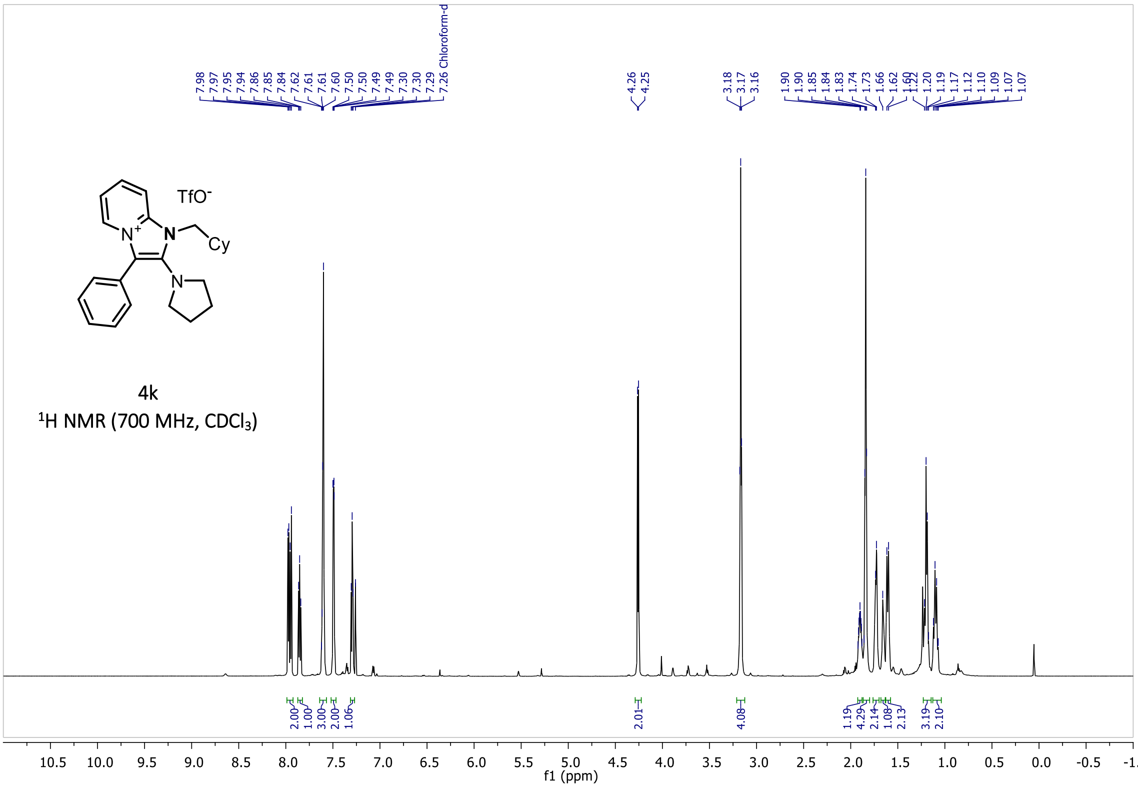

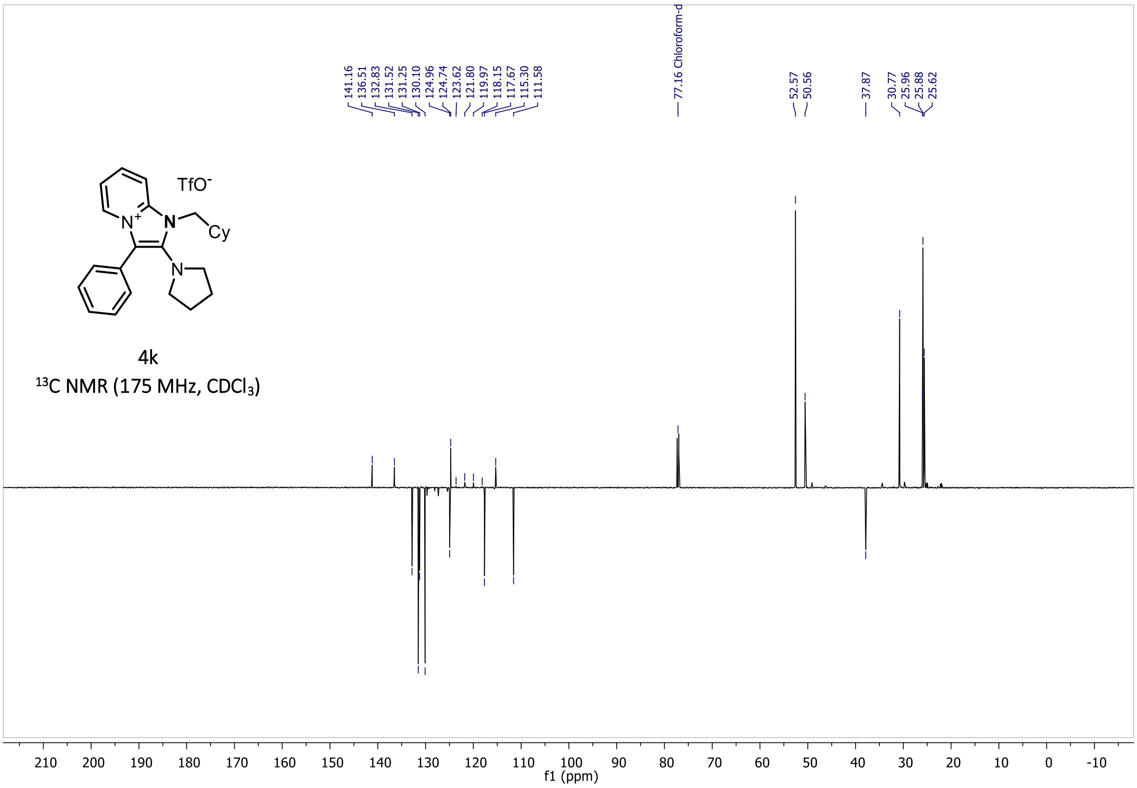

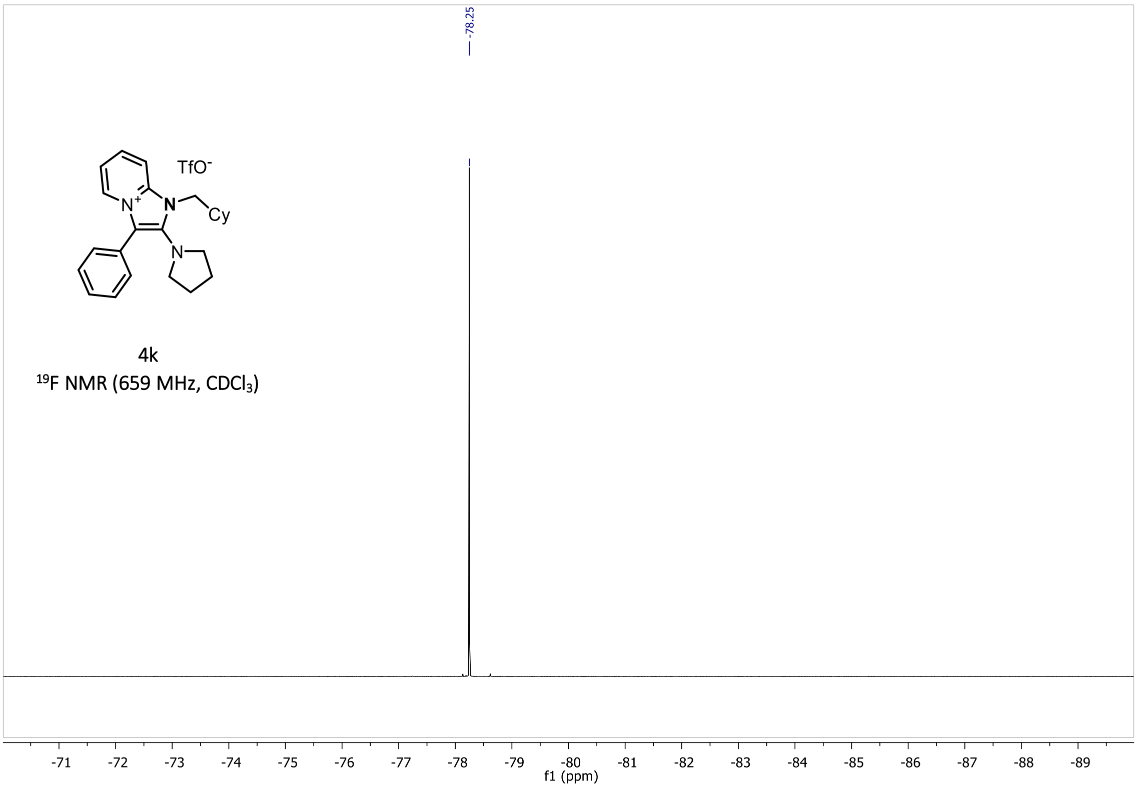

1-(Oct-7-en-1-yl)-3-phenyl-2-(pyrrolidin-1-yl)-1H-imidazo[1,2-a]pyridin-4-ium trifluoromethanesulfonate (4l)

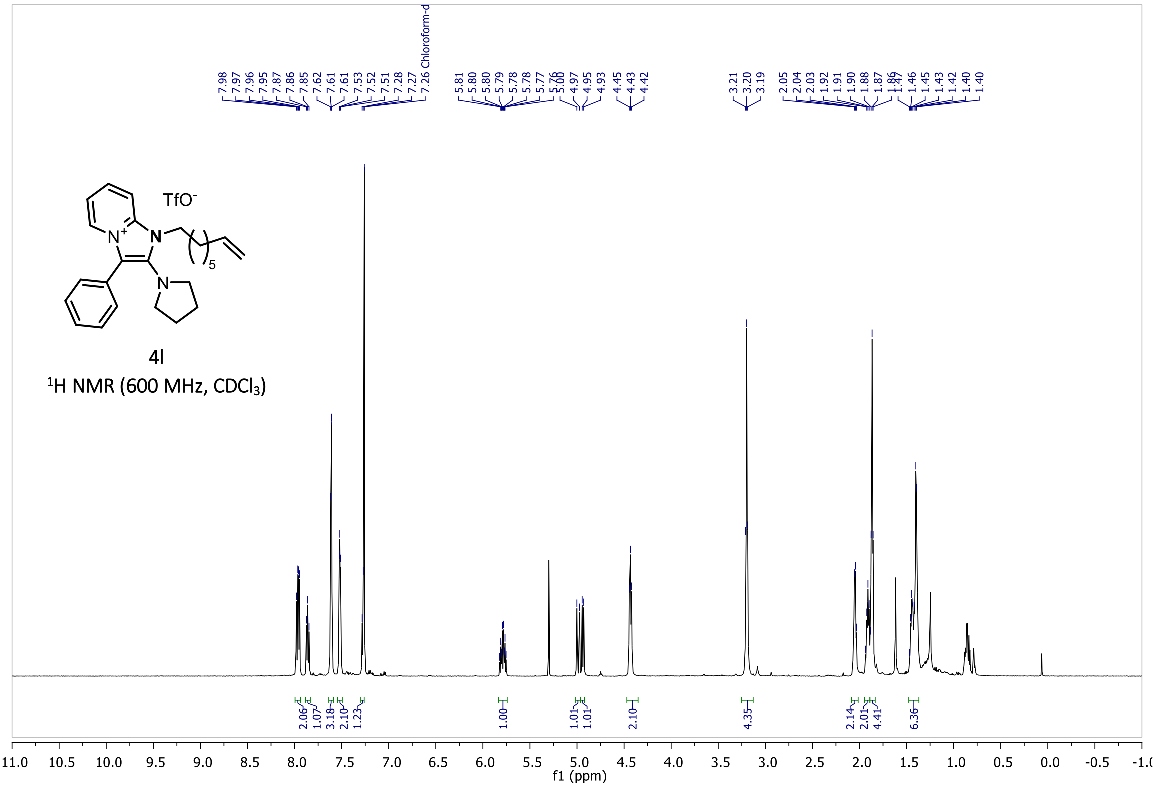

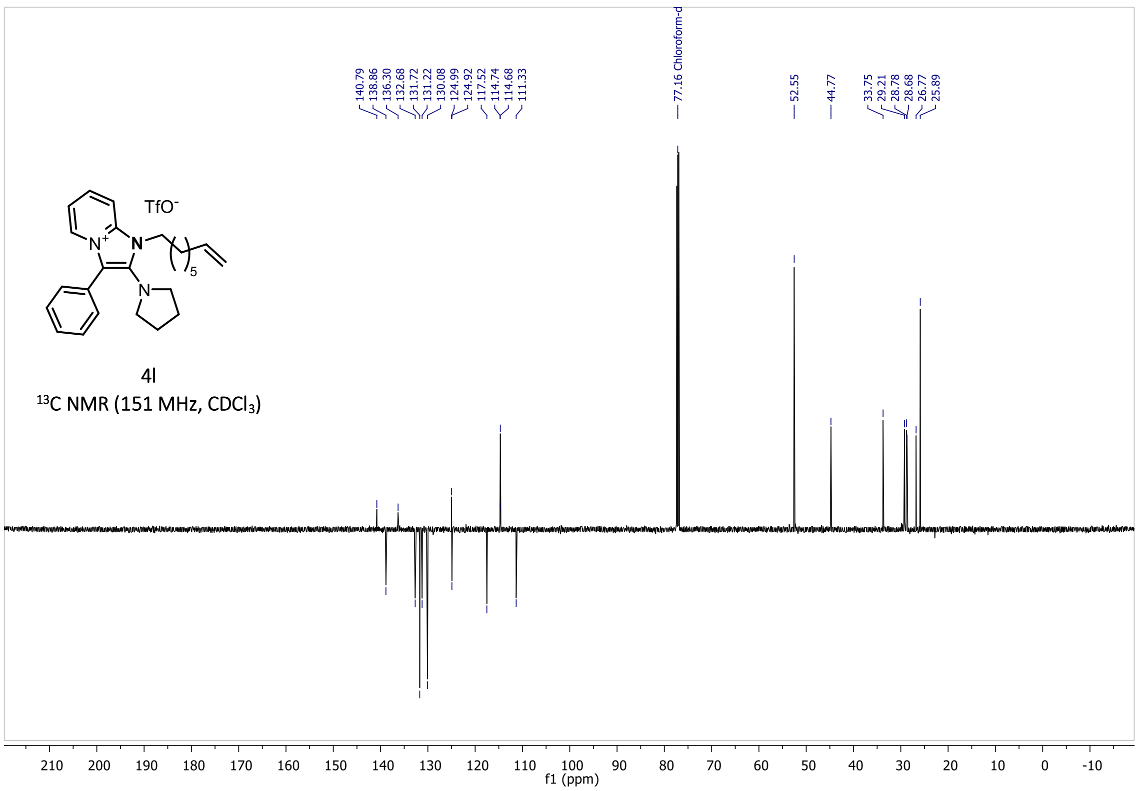

1-(3-Iodopropyl)-3-phenyl-2-(pyrrolidin-1-yl)-1H-imidazo[1,2-a]pyridin-4-ium trifluoromethanesulfonate (4m)

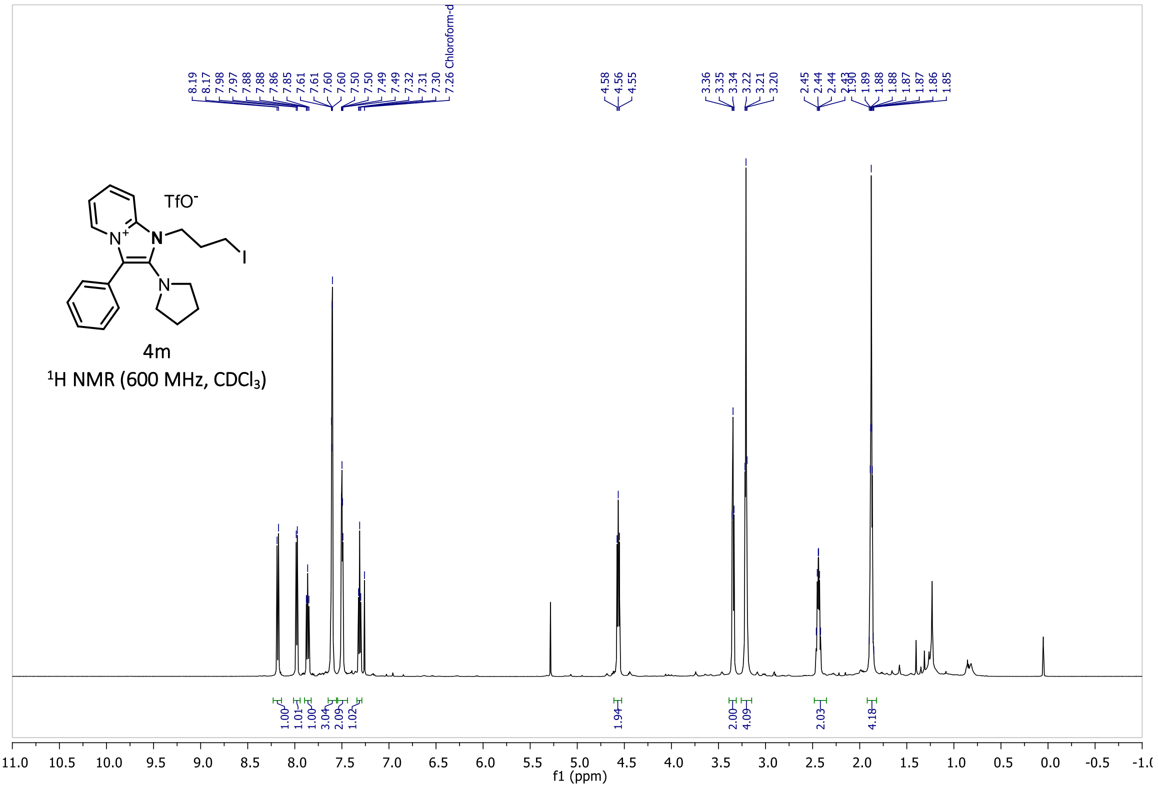

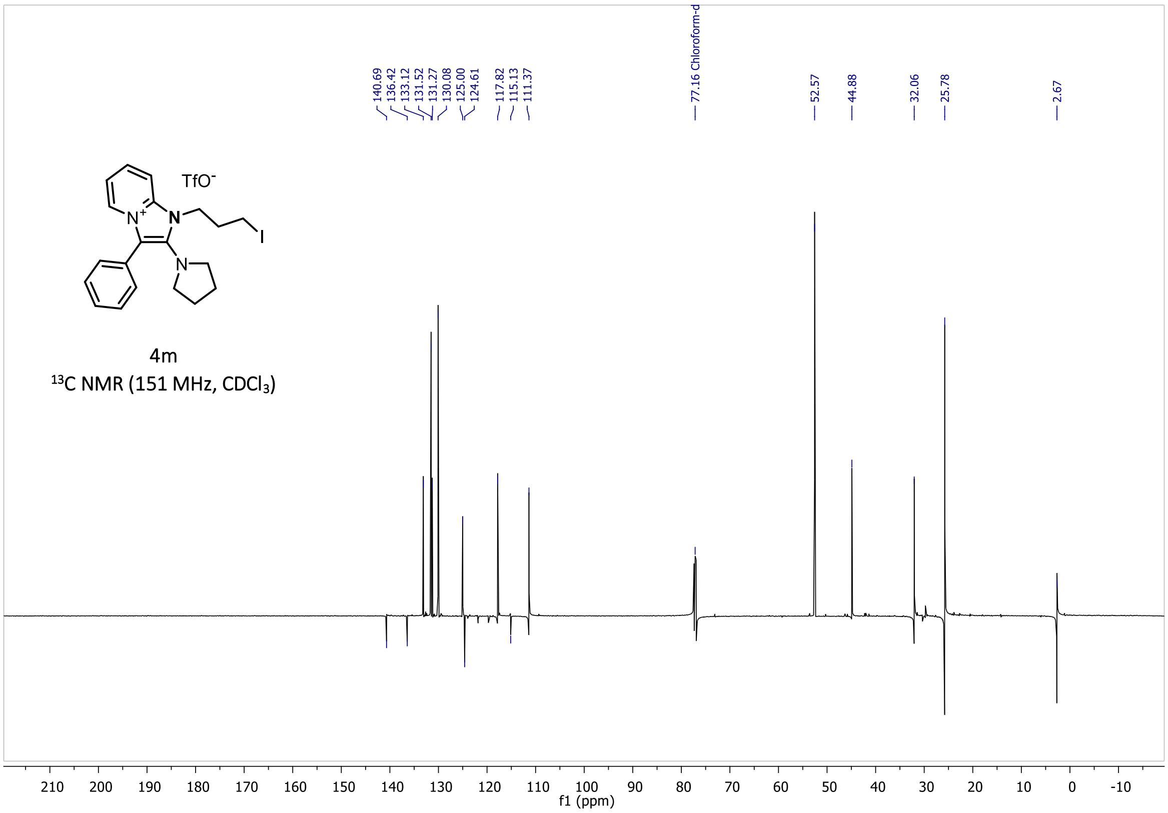

140.69
136.42
133.12
131.52
131.27
130.08
125.00
124.61
117.82
115.13
111.37
77.16 Chloroform-d
52.57
44.88
32.06
25.78
2.67
TfO⁻
N
N
N
I
4m
¹³C NMR (151 MHz, CDCl₃)
f1 (ppm)

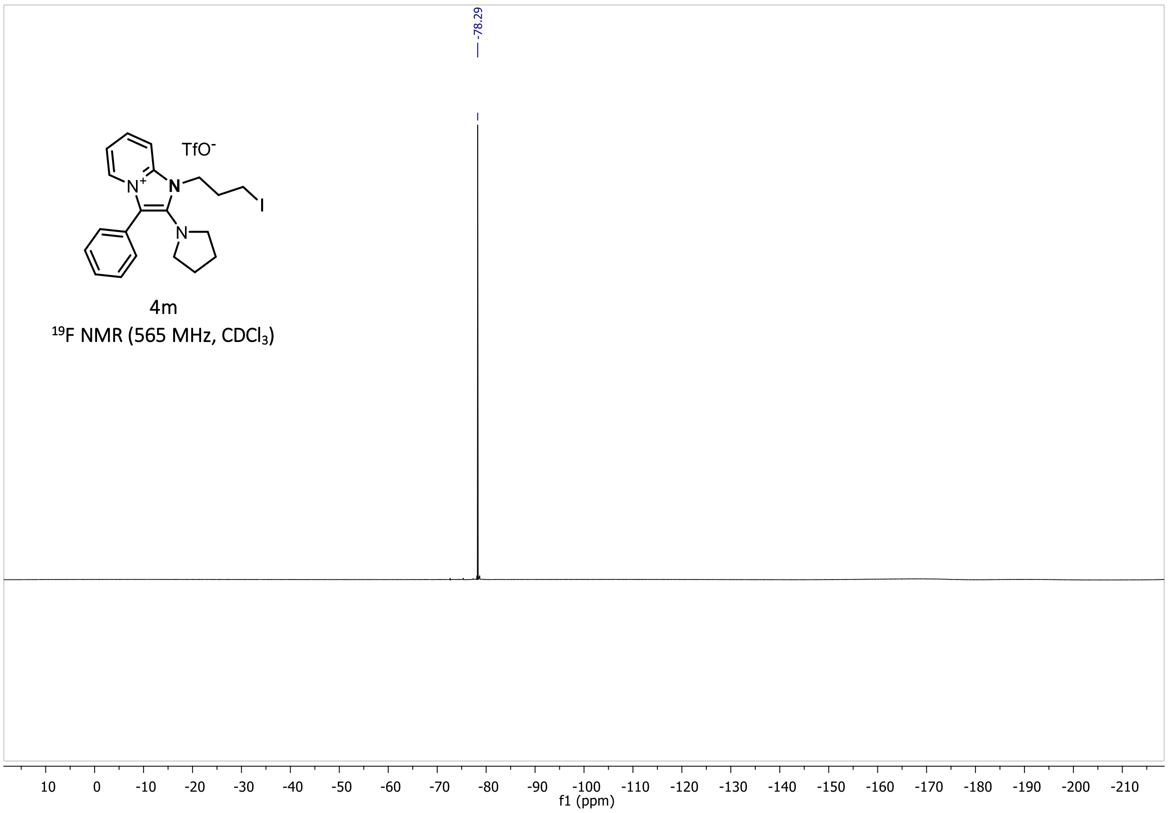

-78.29
TfO⁻
N
N
N
I
4m
¹⁹F NMR (565 MHz, CDCl₃)
f1 (ppm)

1-(3-Azidopropyl)-3-phenyl-2-(pyrrolidin-1-yl)-1H-imidazo[1,2-a]pyridin-4-ium trifluoromethanesulfonate (4n)

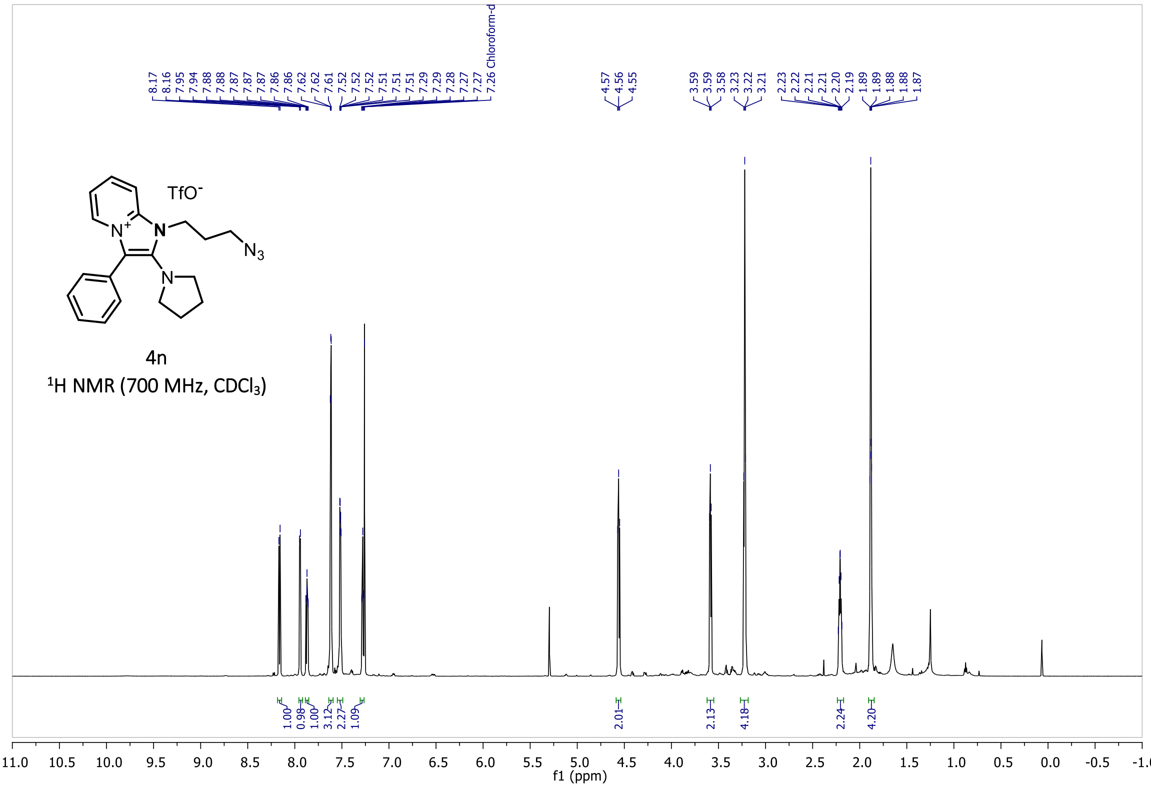

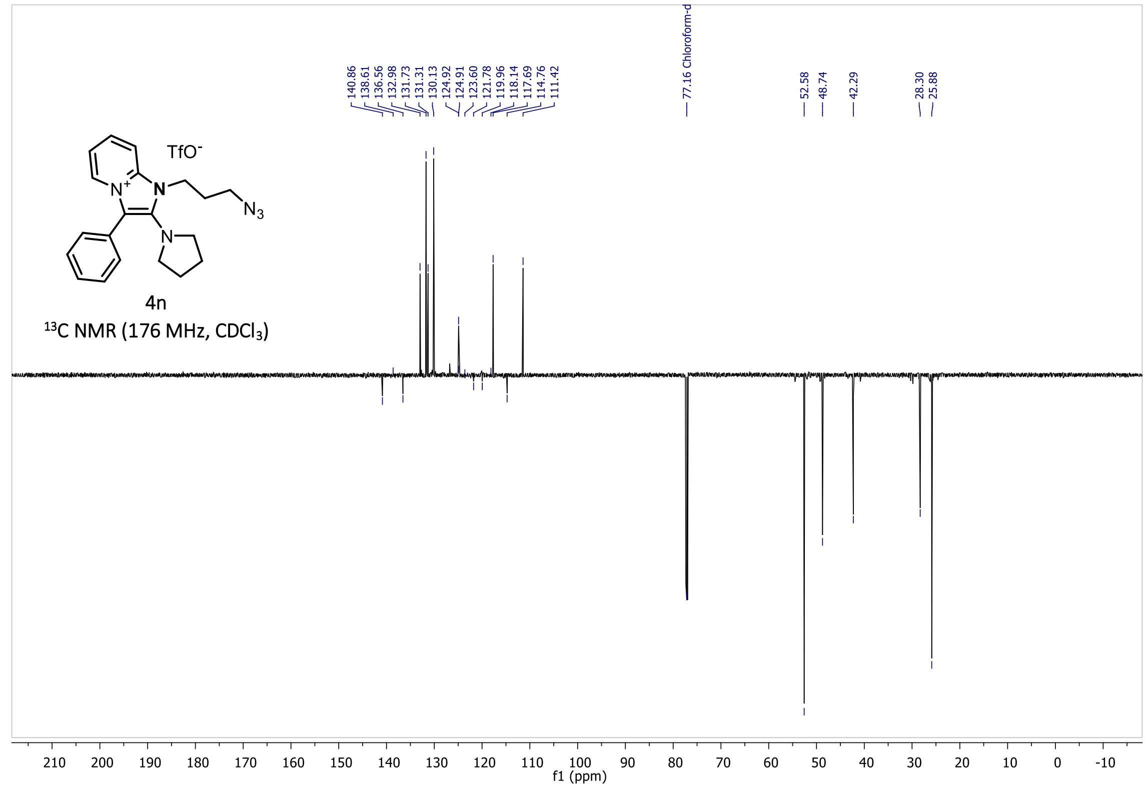

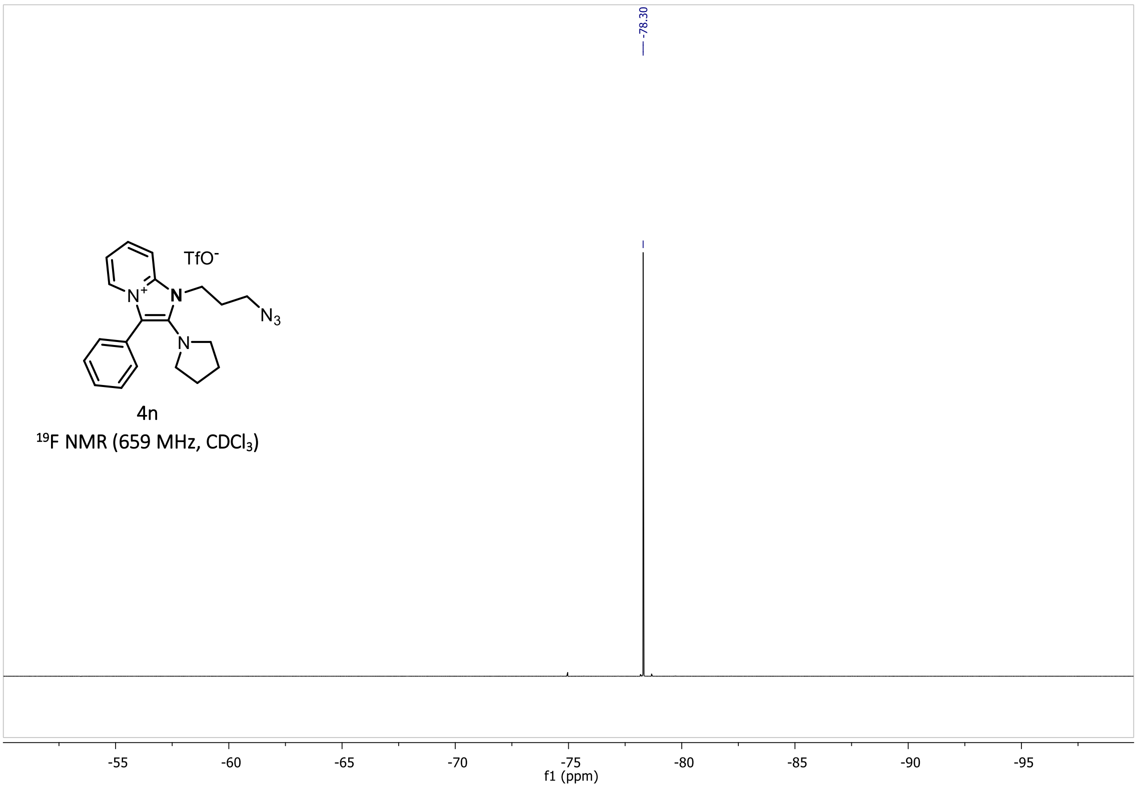

1-(4-Fluorophenethyl)-3-phenyl-2-(pyrrolidin-1-yl)-1H-imidazo[1,2-a]pyridin-4-ium trifluoromethanesulfonate (4o)

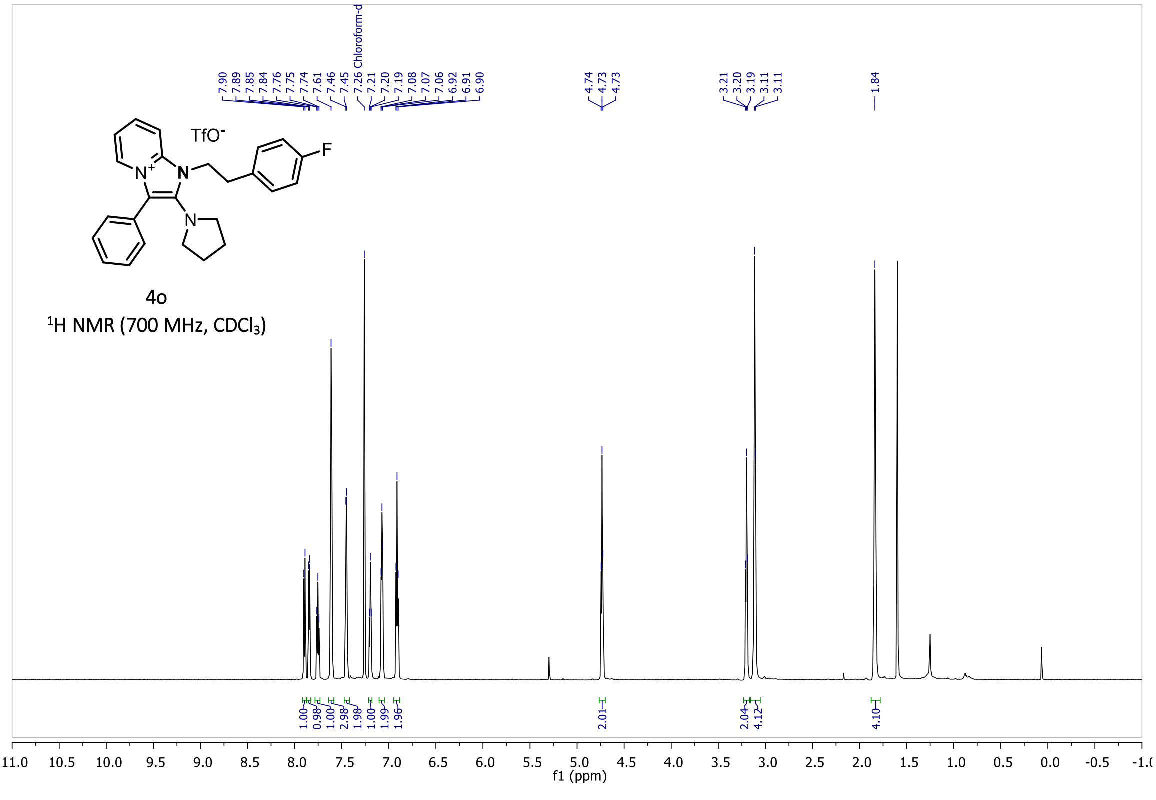

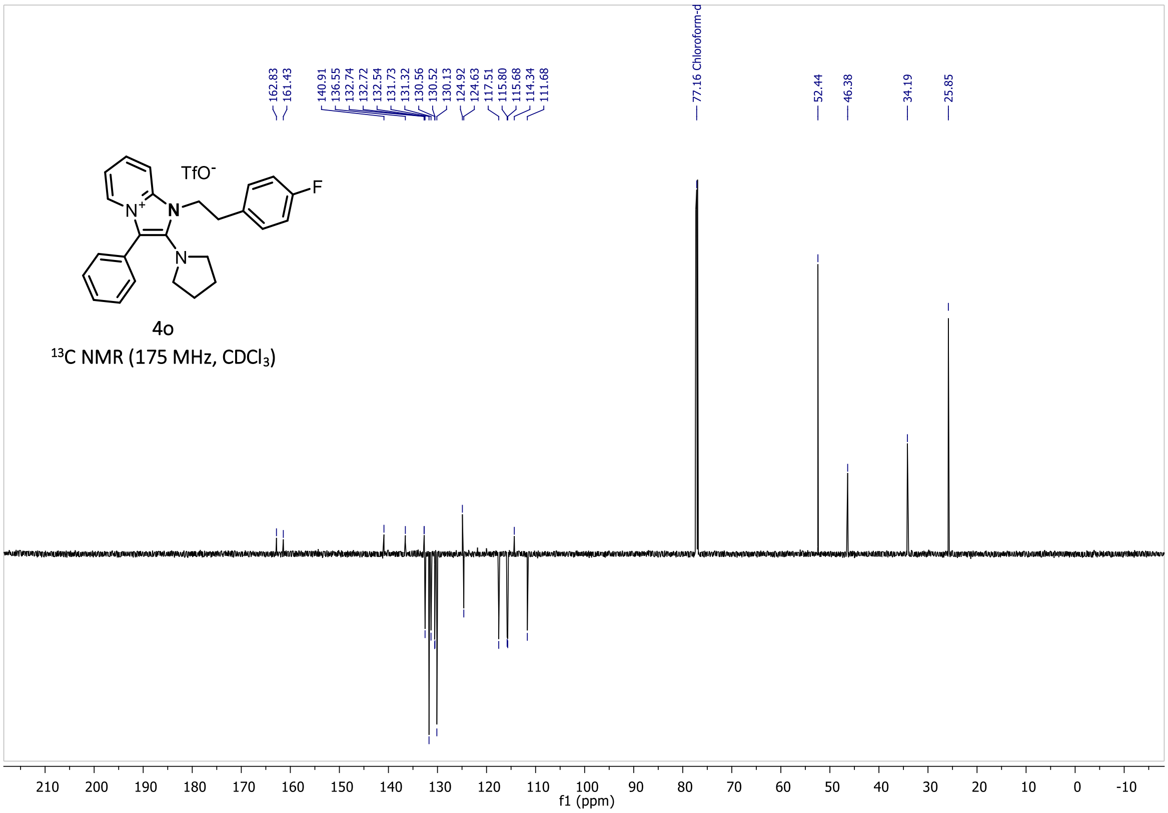
162.83
161.43
140.91
136.55
132.74
132.72
132.54
131.73
131.32
130.56
130.52
130.13
124.92
124.63
117.51
115.80
115.68
114.34
111.68
77.16 Chloroform-d
52.44
46.38
34.19
25.85
TfO-
F
N+
N
N
4o
^{13}C NMR (175 MHz, CDCl$_3$)
210 200 190 180 170 160 150 140 130 120 110 100 90 80 70 60 50 40 30 20 10 0 -10
f1 (ppm)

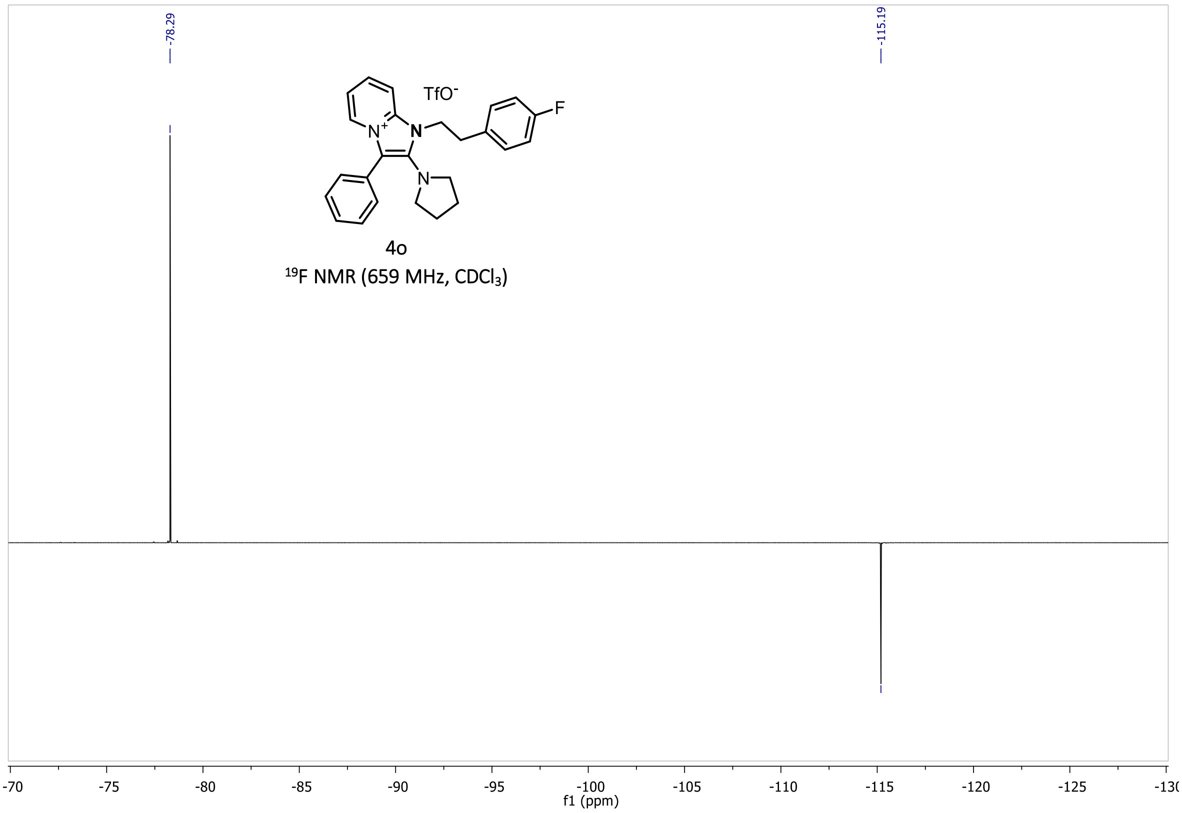
-78.29
-115.19
TfO-
F
N+
N
N
4o
^{19}F NMR (659 MHz, CDCl$_3$)
-70 -75 -80 -85 -90 -95 -100 -105 -110 -115 -120 -125 -130
f1 (ppm)

1-(4-Methylphenethyl)-3-phenyl-2-(pyrrolidin-1-yl)-1H-imidazo[1,2-a]pyridin-4-ium trifluoromethanesulfonate (4p)

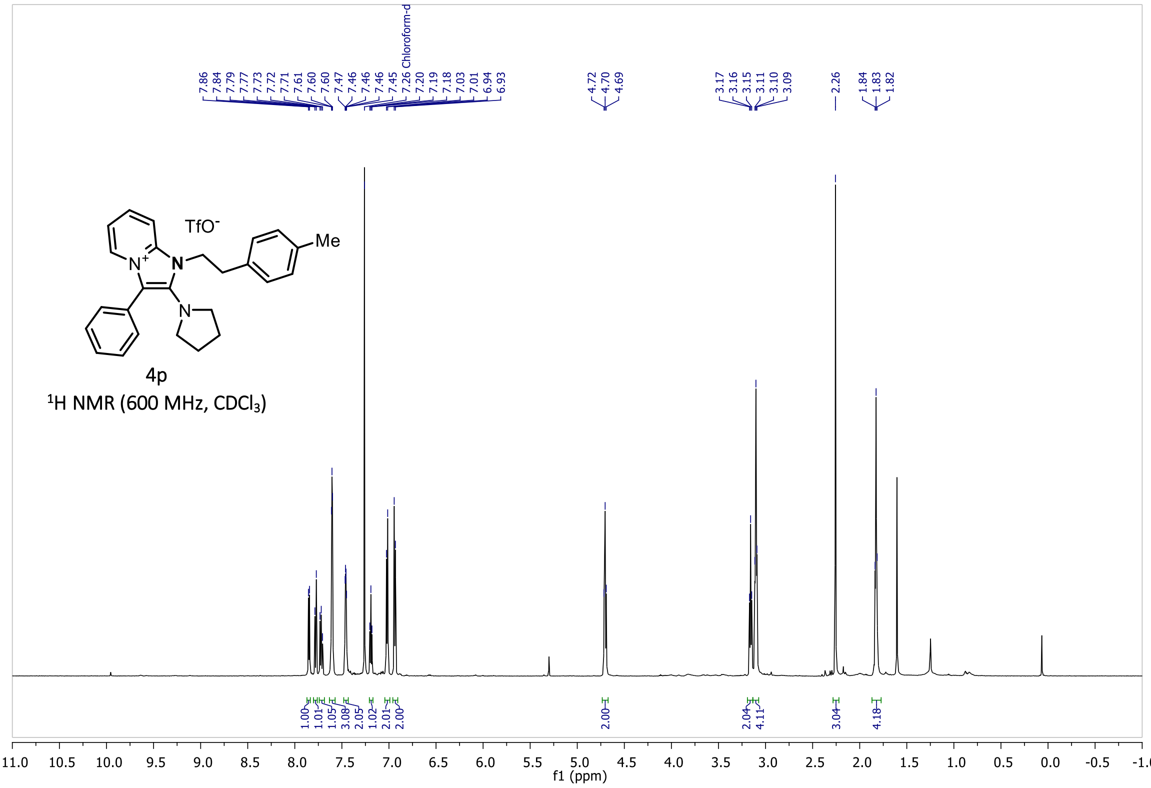

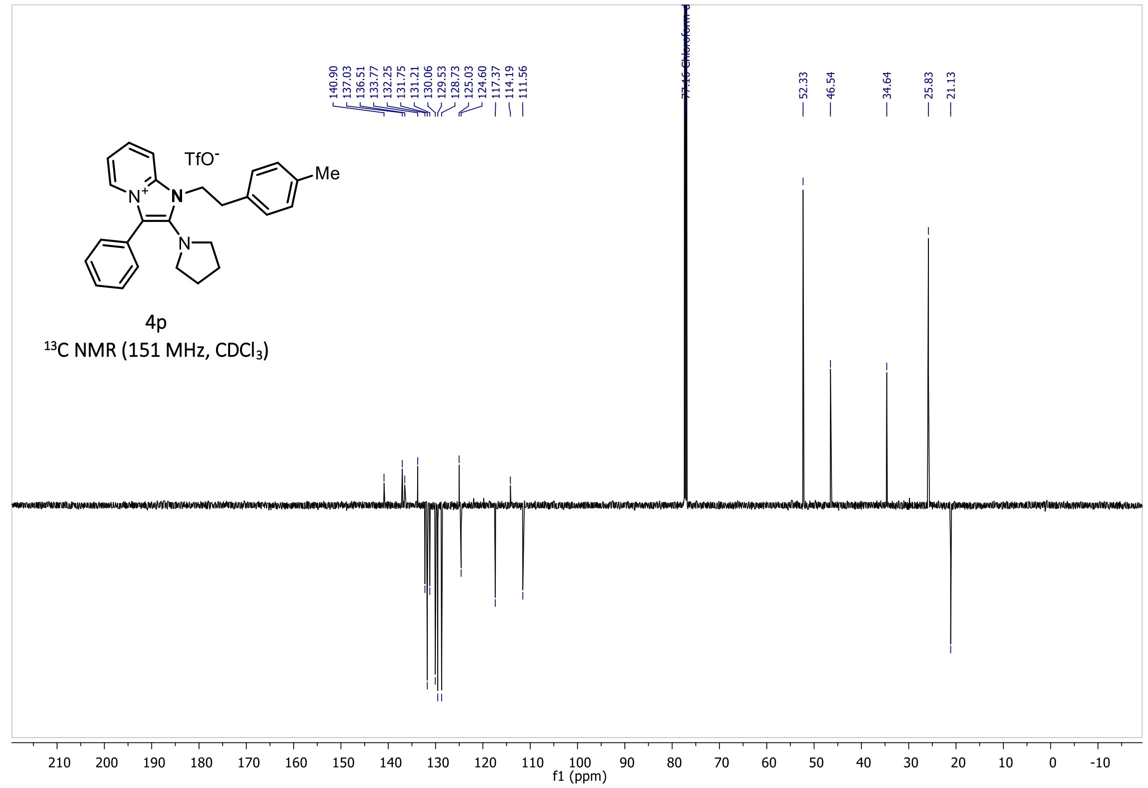

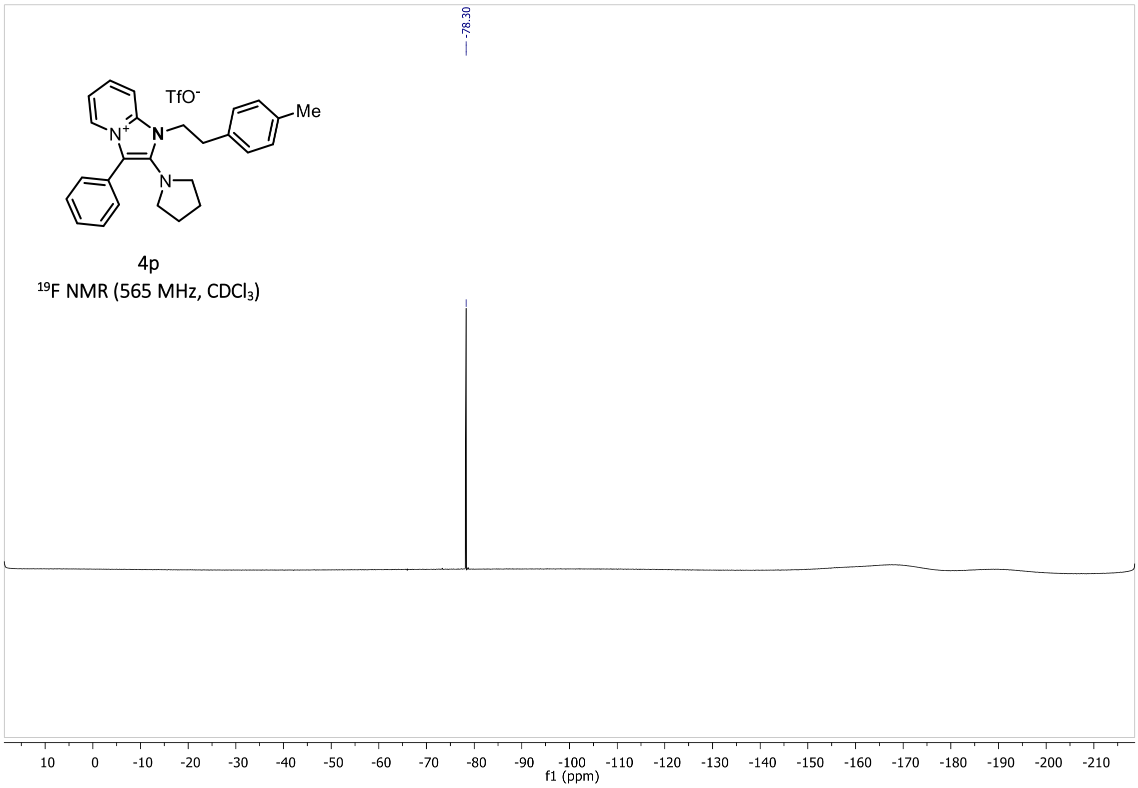

7-(Phenanthren-9-yl)-1-phenethyl-3-phenyl-2-(pyrrolidin-1-yl)-1H-imidazo[1,2-a]pyridin-4-ium trifluoromethanesulfonate (4q)

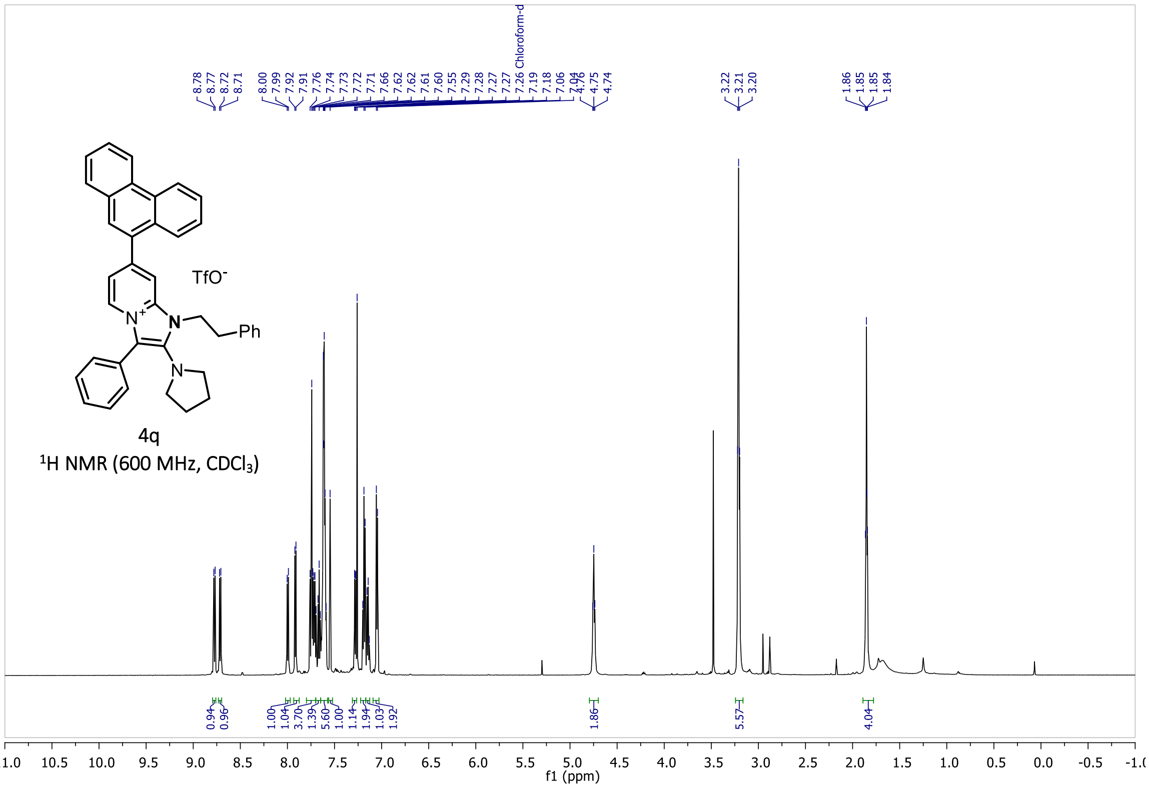

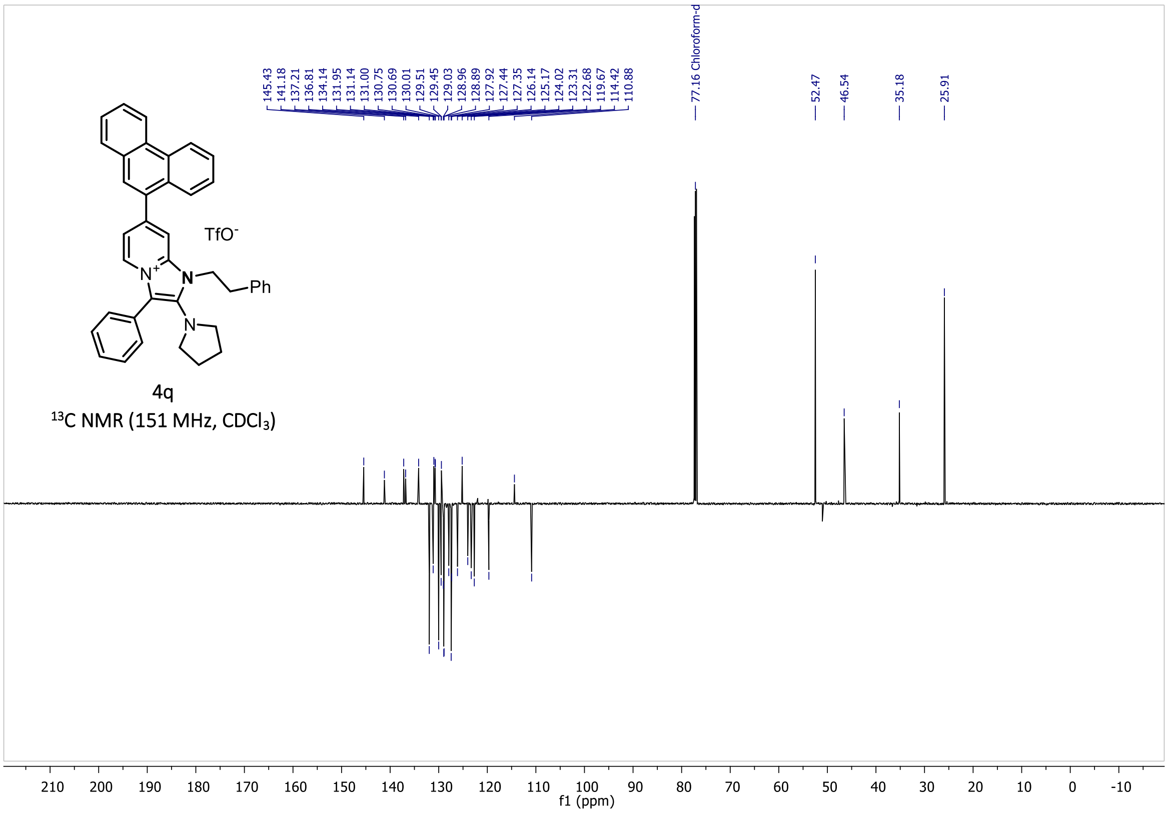
145.43
141.18
137.21
136.81
134.14
131.95
131.14
131.00
130.75
130.69
130.01
129.51
129.45
129.03
128.96
128.89
127.92
127.44
127.35
126.14
125.17
124.02
123.31
122.68
119.67
114.42
110.88
77.16 Chloroform-d
52.47
46.54
35.18
25.91
TfO-
N+
N
Ph
N
4q
13C NMR (151 MHz, CDCl3)
210
200
190
180
170
160
150
140
130
120
110
100
90
80
70
60
50
40
30
20
10
0
-10
f1 (ppm)

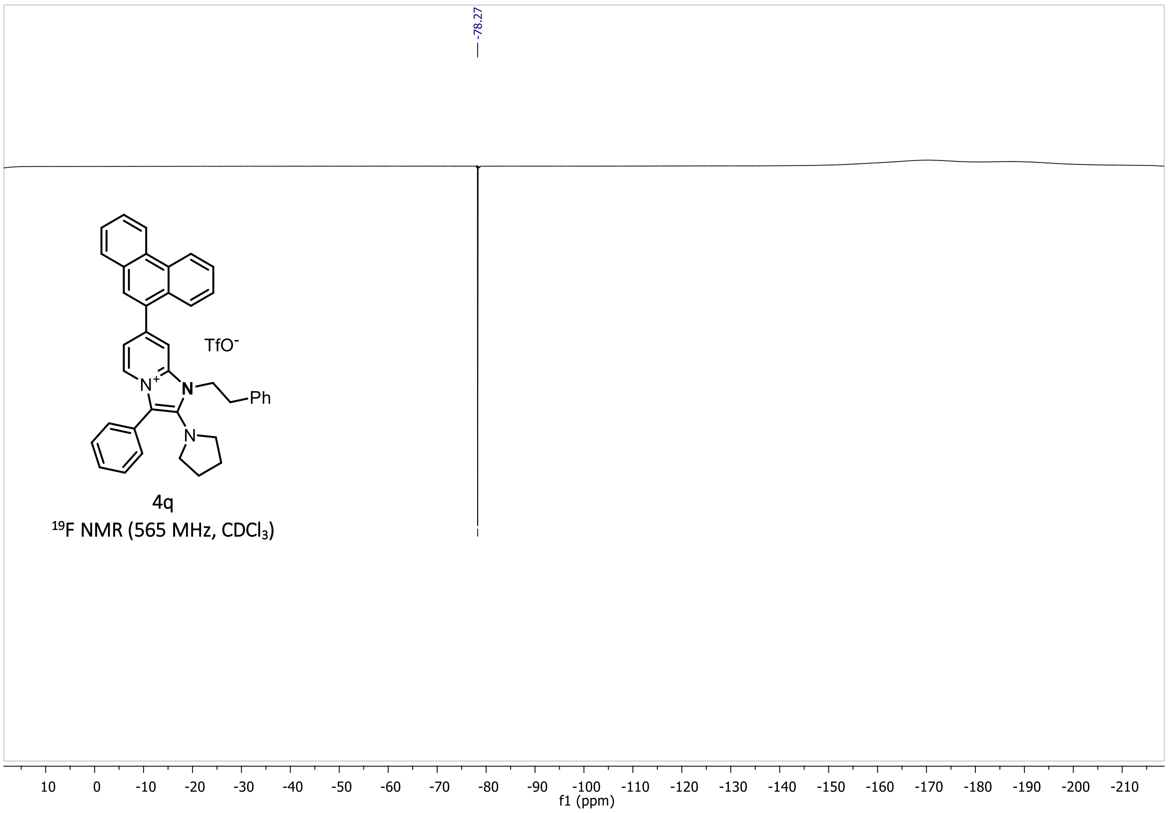
-78.27
TfO-
N+
N
Ph
N
4q
19F NMR (565 MHz, CDCl3)
10
0
-10
-20
-30
-40
-50
-60
-70
-80
-90
-100
-110
-120
-130
-140
-150
-160
-170
-180
-190
-200
-210
f1 (ppm)

1-Phenethyl-3,7-diphenyl-2-(pyrrolidin-1-yl)-1H-imidazo[1,2-a]pyridin-4-ium trifluoromethanesulfonate (4r)

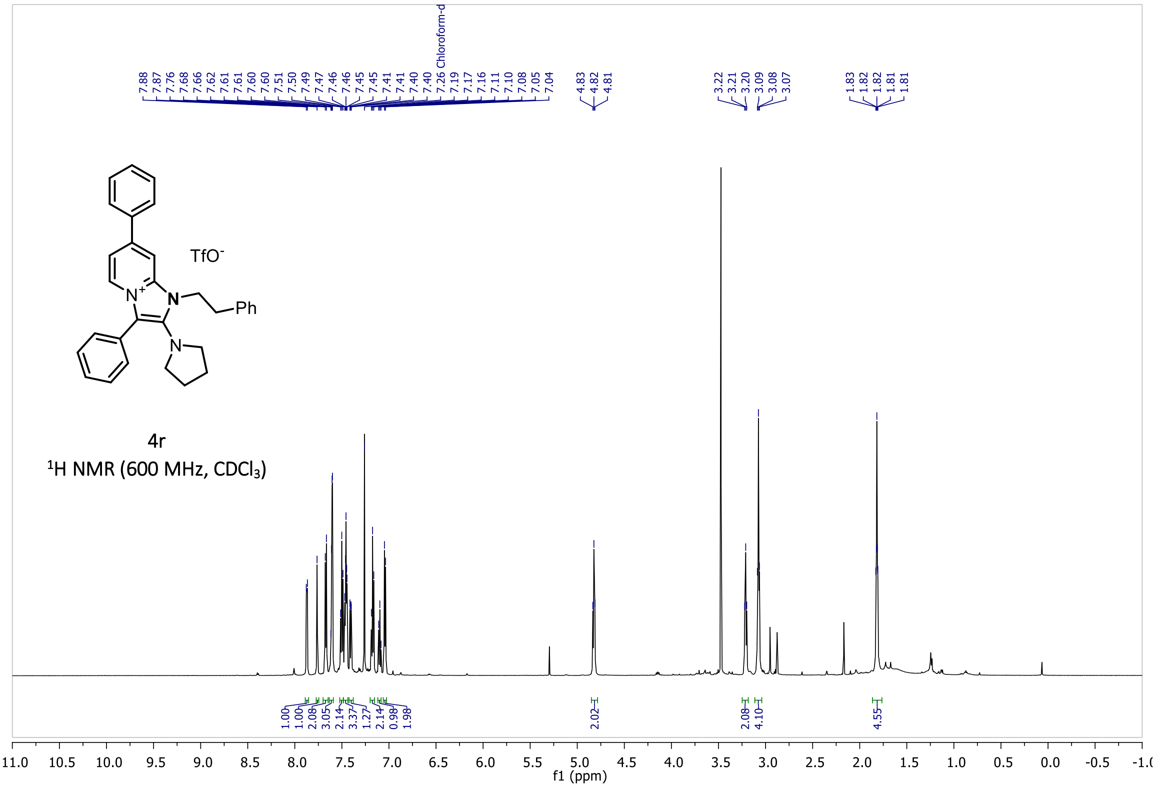

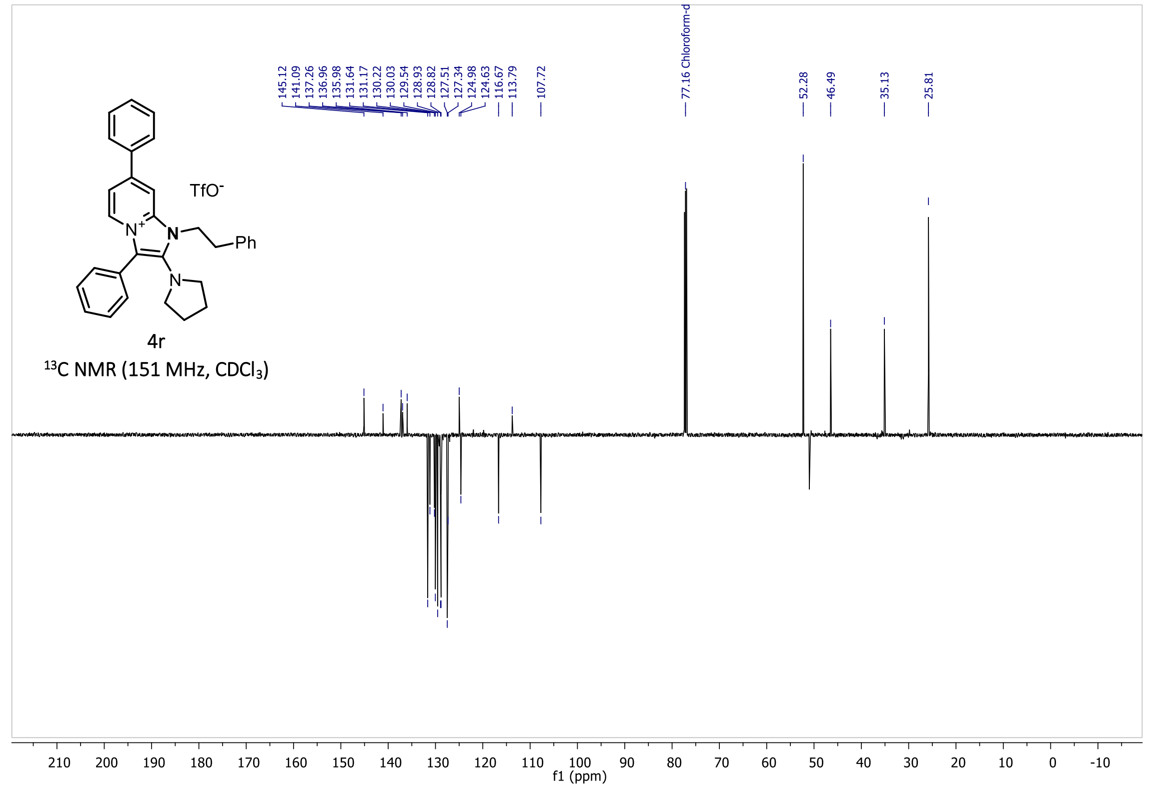

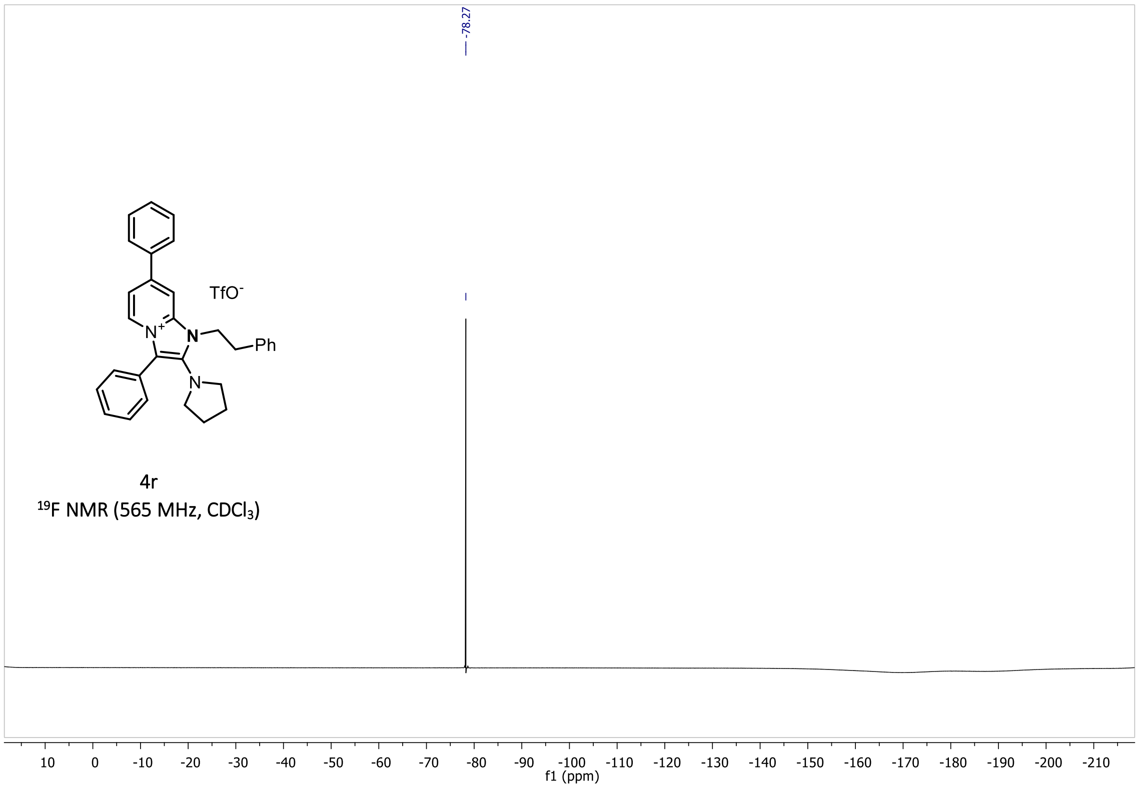

1-Phenethyl-3-phenyl-2-(pyrrolidin-1-yl)-7-(4-(trifluoromethyl)phenyl)-1H-imidazo[1,2-a]pyridin-4-ium trifluoromethanesulfonate (4s)

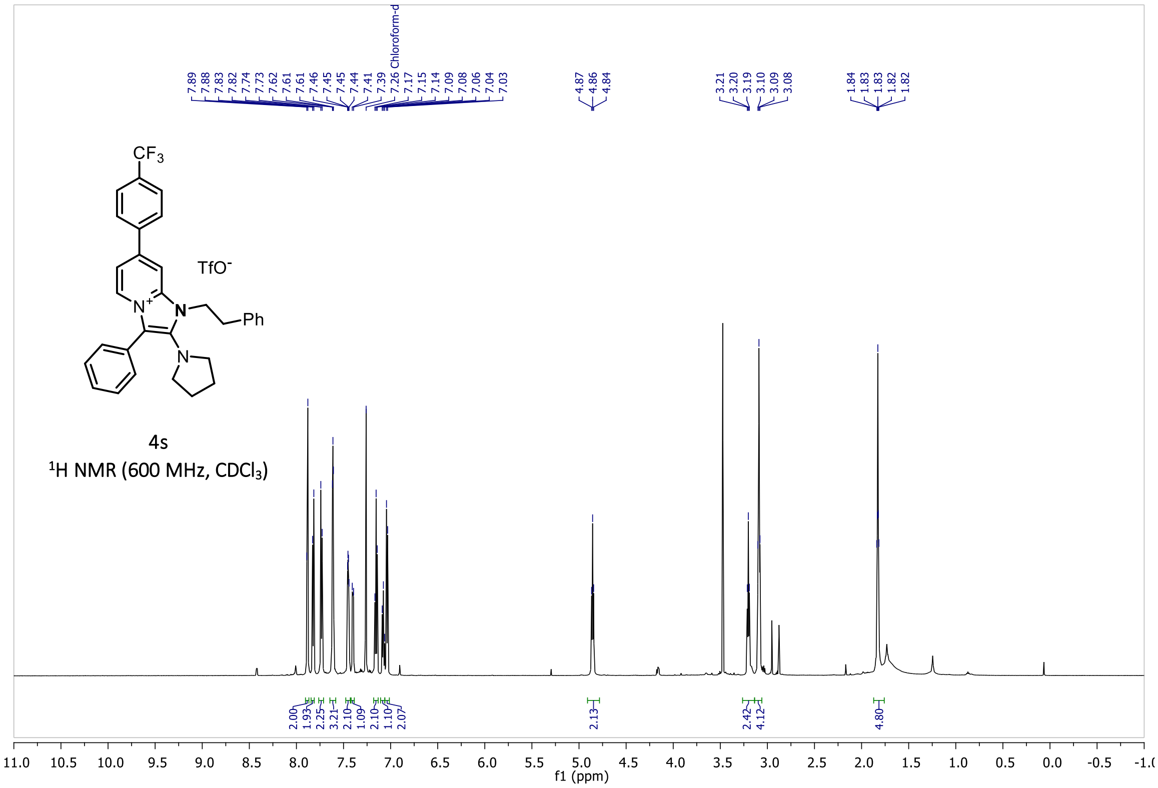

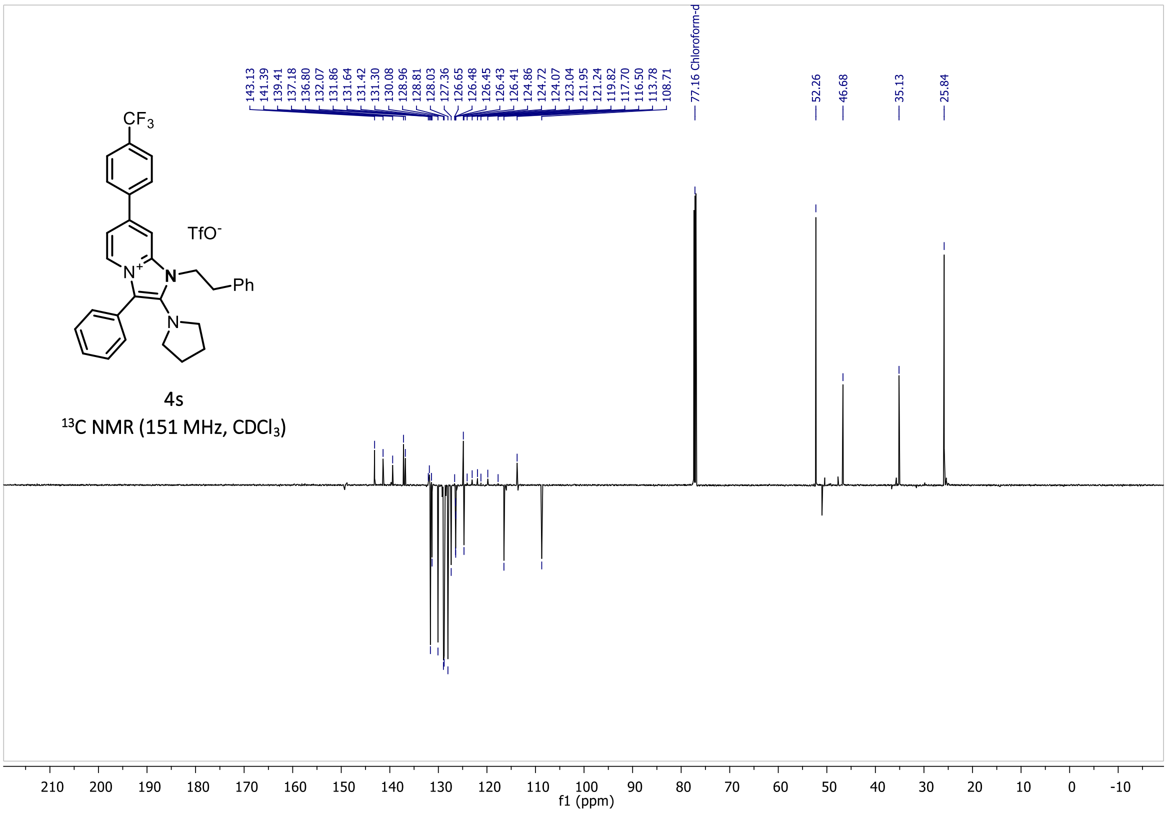

4s

^{13}C NMR (151 MHz, $CDCl_3$)

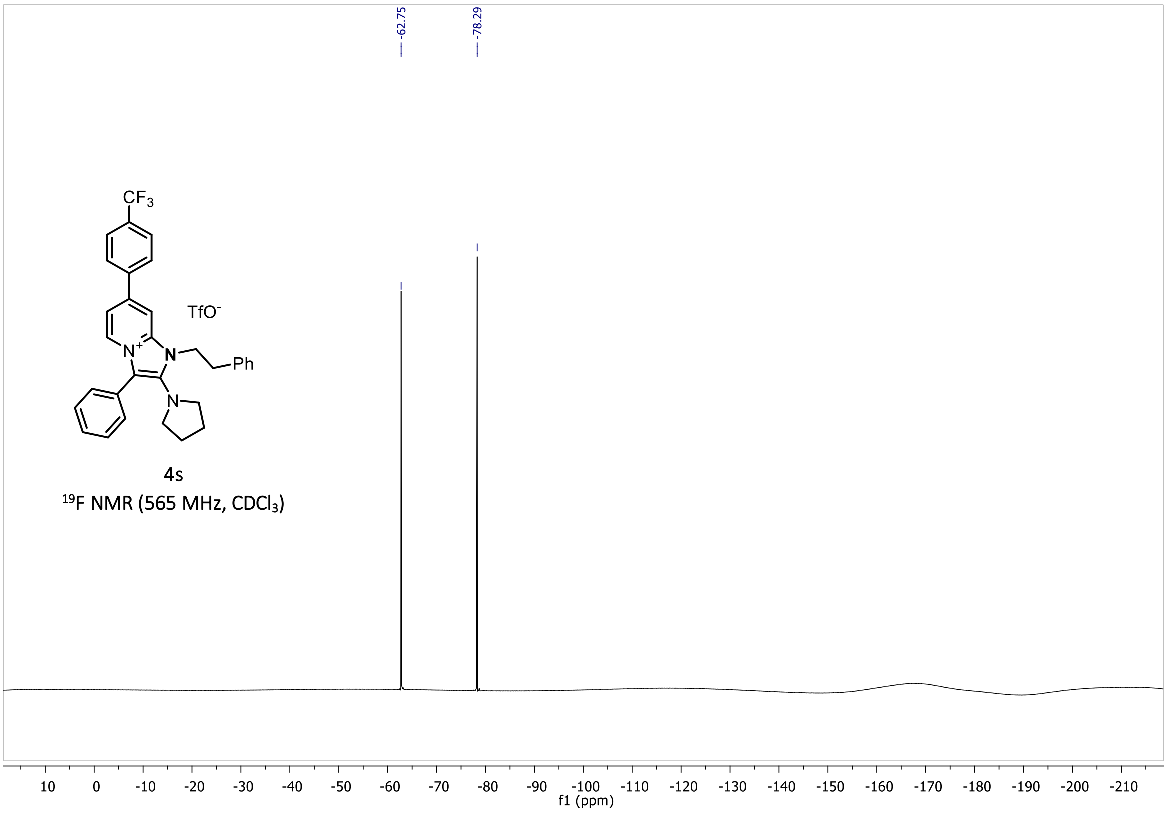

4s

^{19}F NMR (565 MHz, $CDCl_3$)

7-(4-Fluorophenyl)-1-phenethyl-3-phenyl-2-(pyrrolidin-1-yl)-1H-imidazo[1,2-a]pyridin-4-ium trifluoromethanesulfonate (4t)

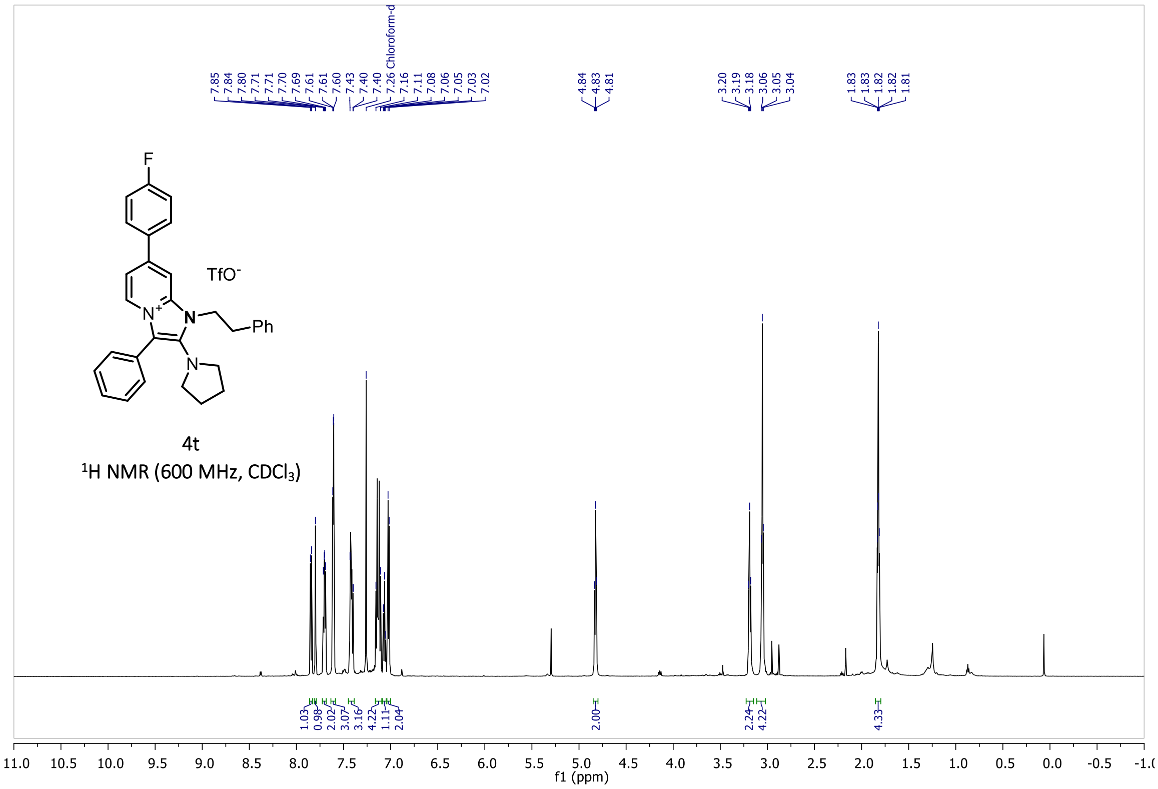

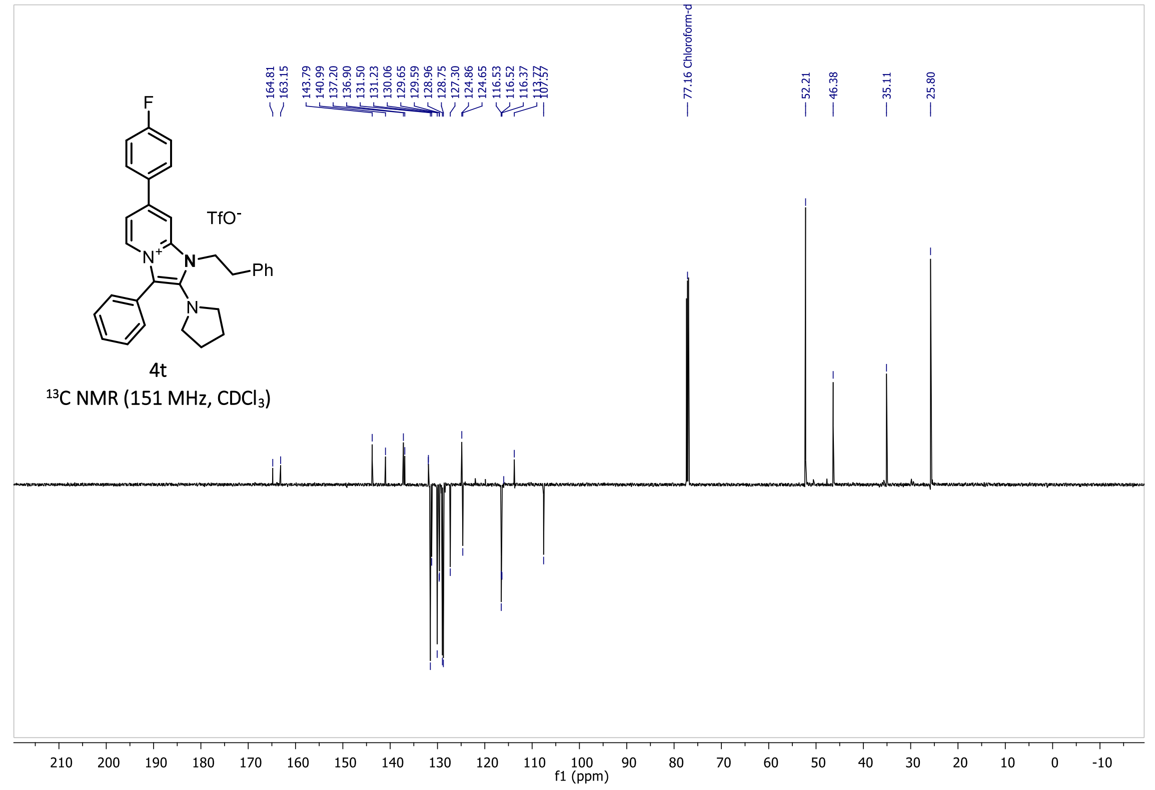

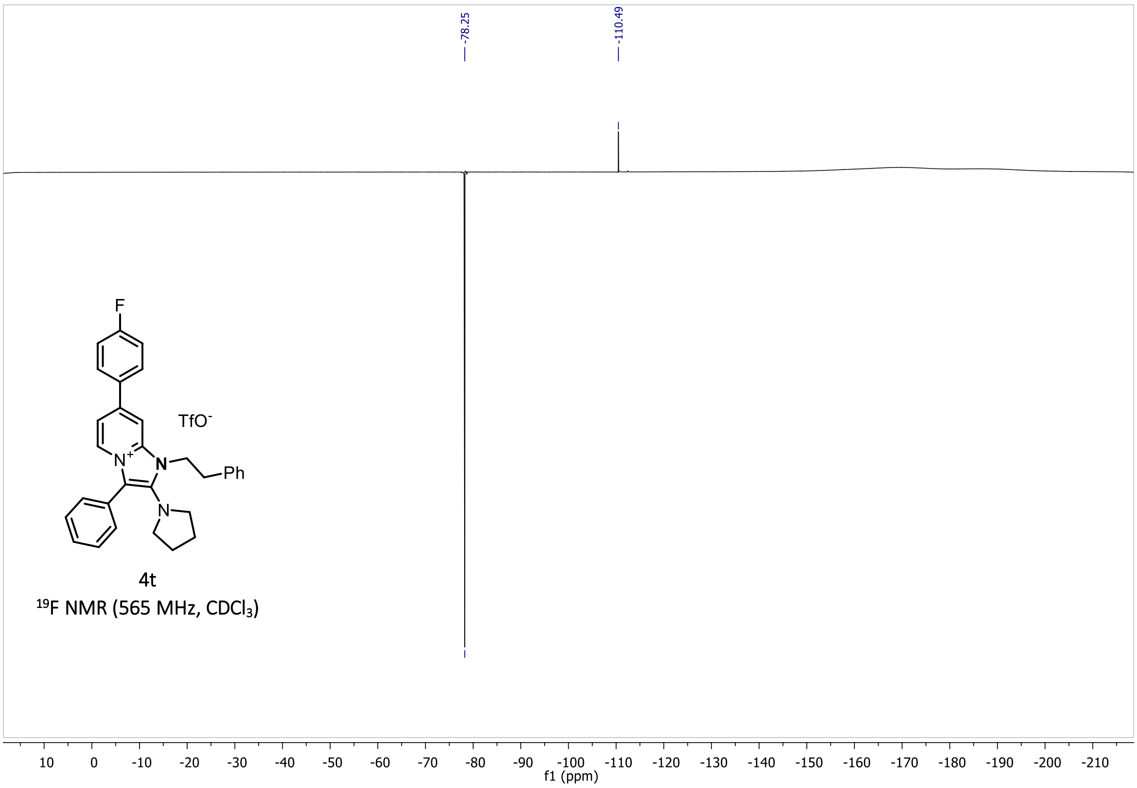

7-(4-Methoxyphenyl)-1-phenethyl-3-phenyl-2-(pyrrolidin-1-yl)-1H-imidazo[1,2-a]pyridin-4-ium trifluoromethanesulfonate (4u)

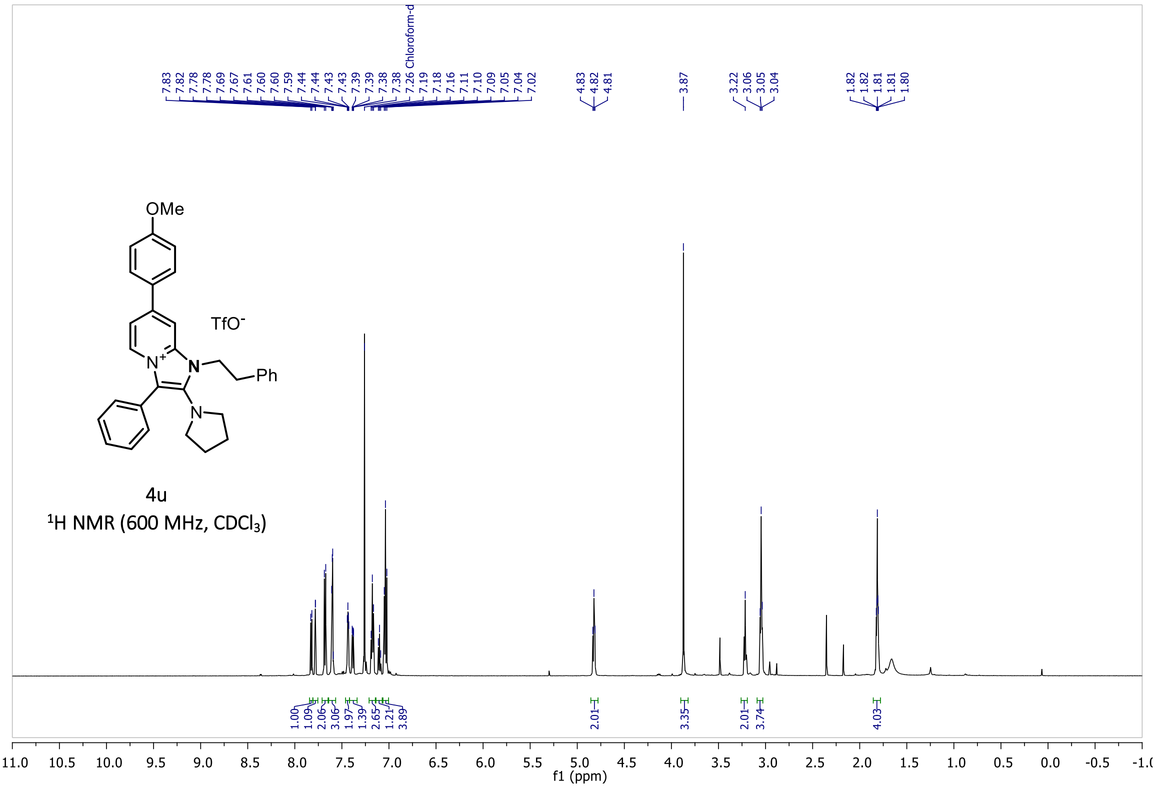

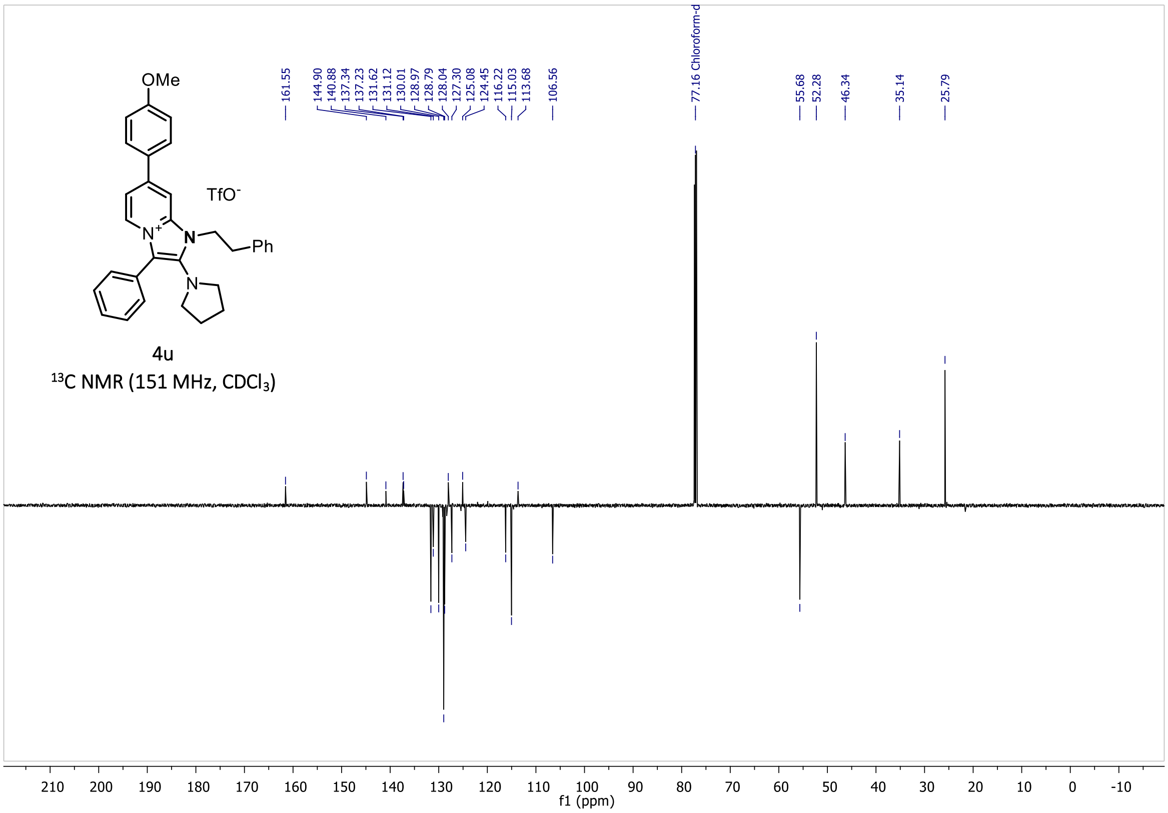
OMe
TfO⁻
Ph
4u
13C NMR (151 MHz, CDCl3)
161.55
144.90
140.88
137.34
137.23
131.62
131.12
130.01
128.97
128.79
128.04
127.30
125.08
124.45
116.22
115.03
113.68
106.56
77.16 Chloroform-d
55.68
52.28
46.34
35.14
25.79
f1 (ppm)

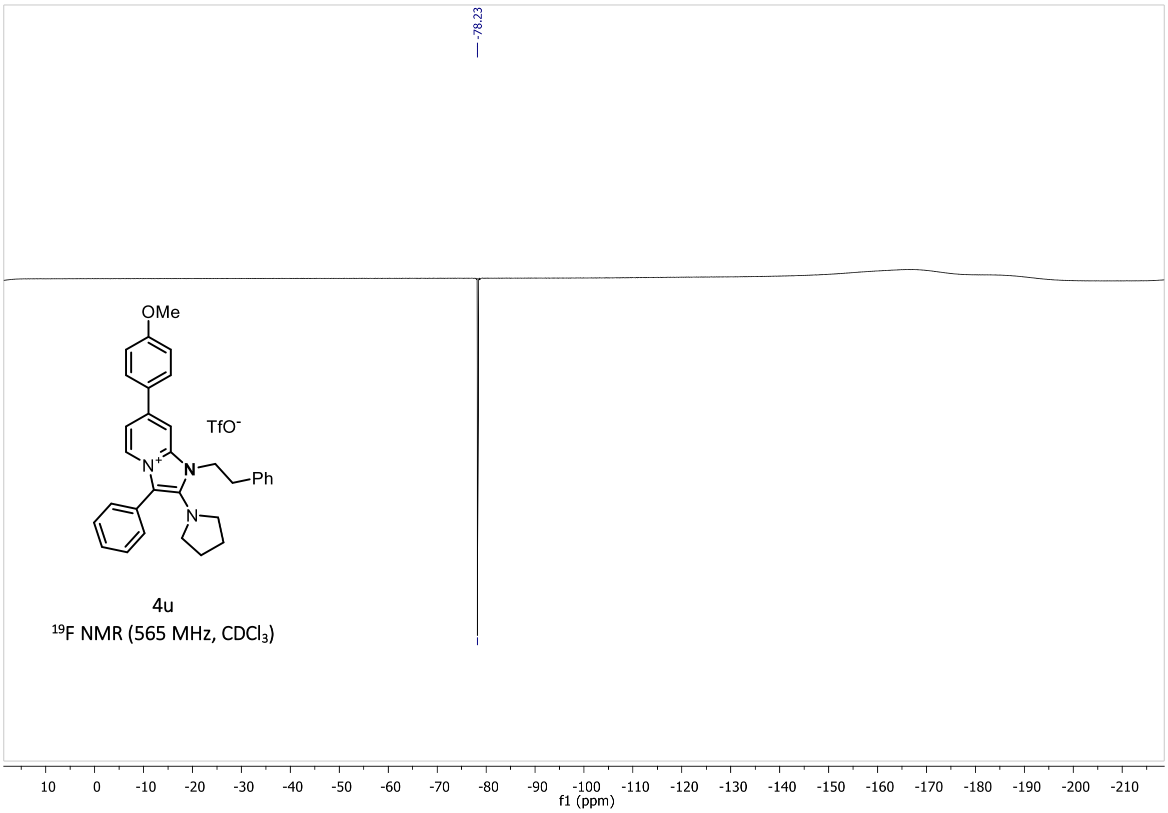
-78.23
OMe
TfO⁻
Ph
4u
19F NMR (565 MHz, CDCl3)
f1 (ppm)

7-Iodo-1-(7-methoxy-7-oxoheptyl)-3-phenyl-2-(pyrrolidin-1-yl)-1H-imidazo[1,2-a]pyridin-4-ium trifluoromethanesulfonate (4v)

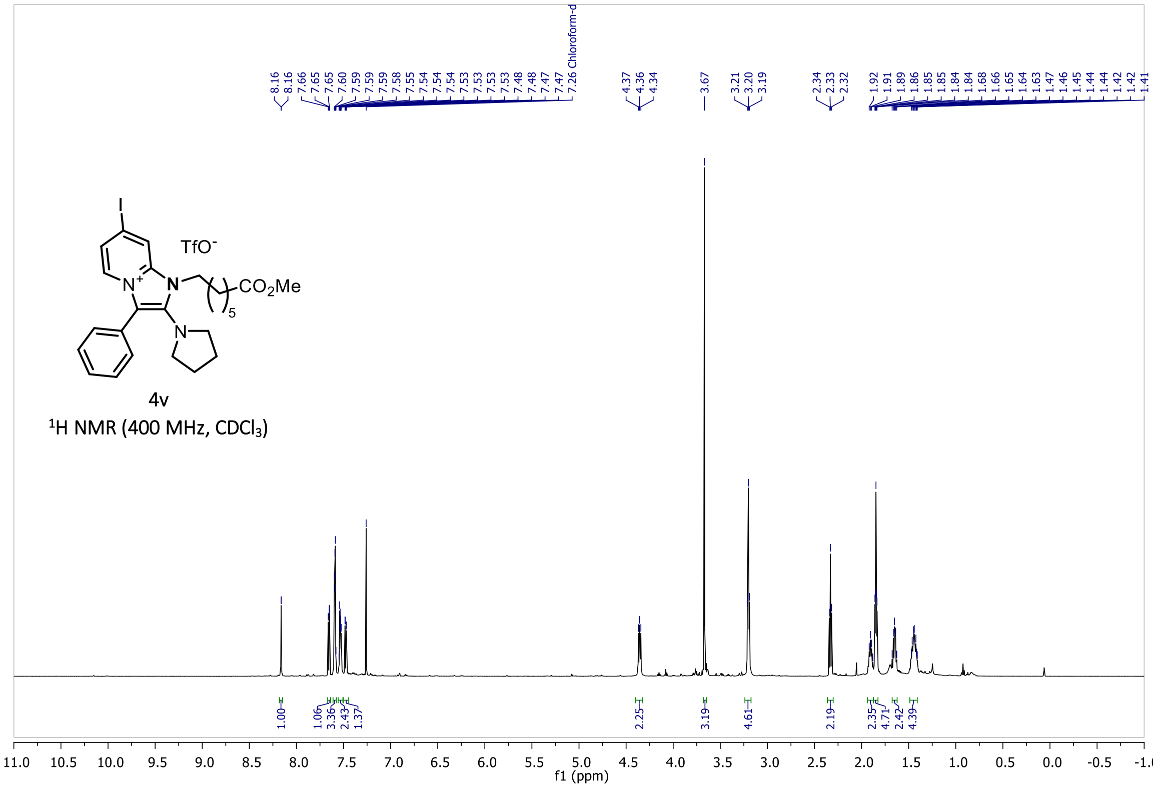

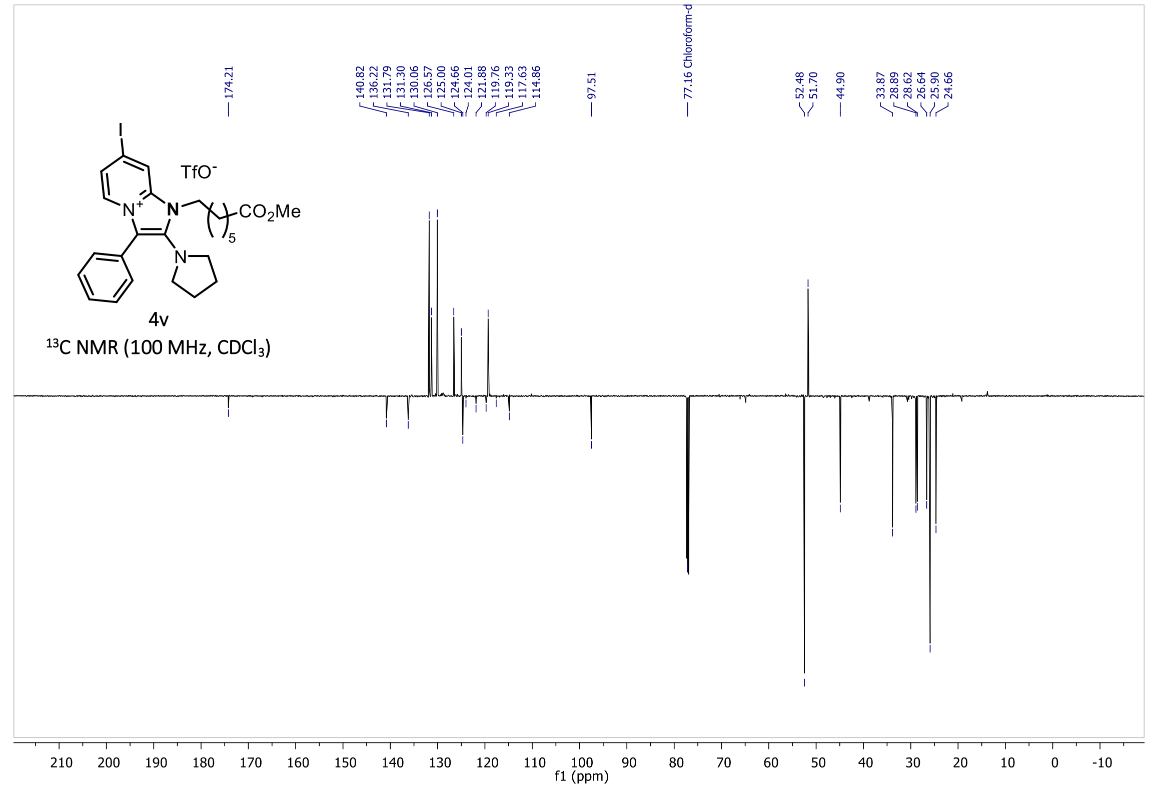

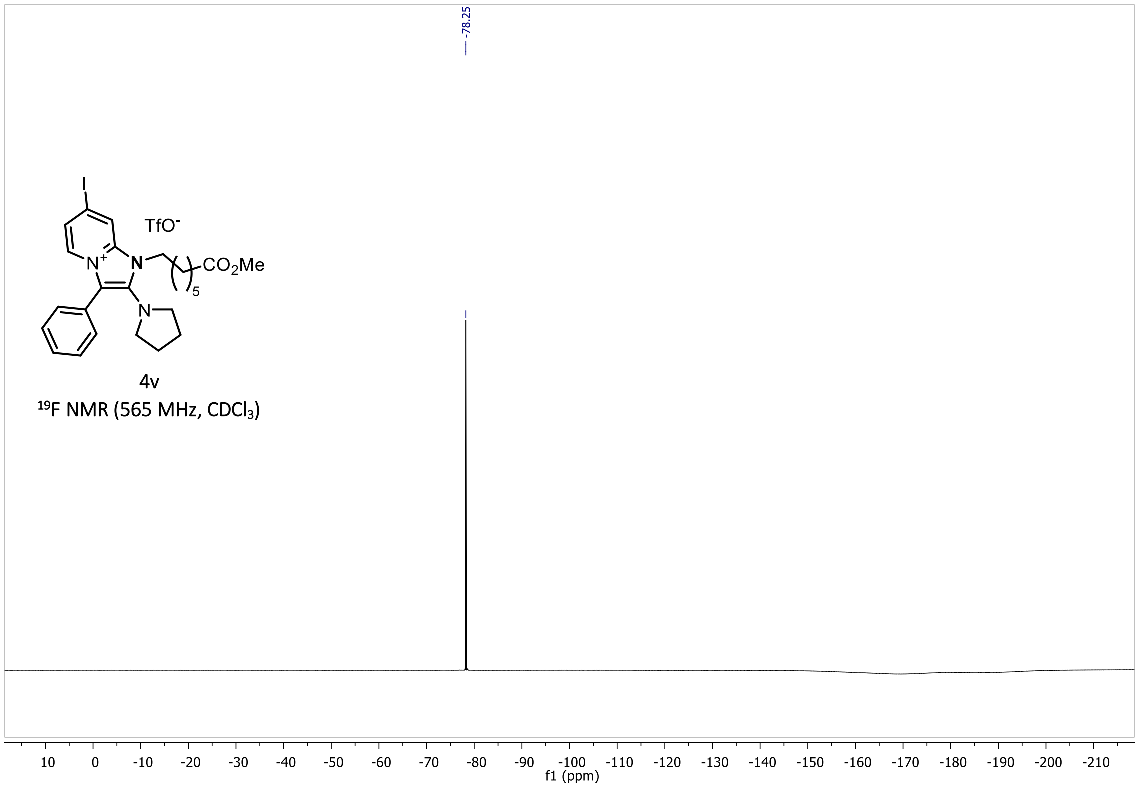

7-Iodo-1-(4-methoxy-4-oxobutyl)-3-phenyl-2-(pyrrolidin-1-yl)-1*H*-imidazo[1,2-a]pyridin-4-ium trifluoromethanesulfonate (4w)

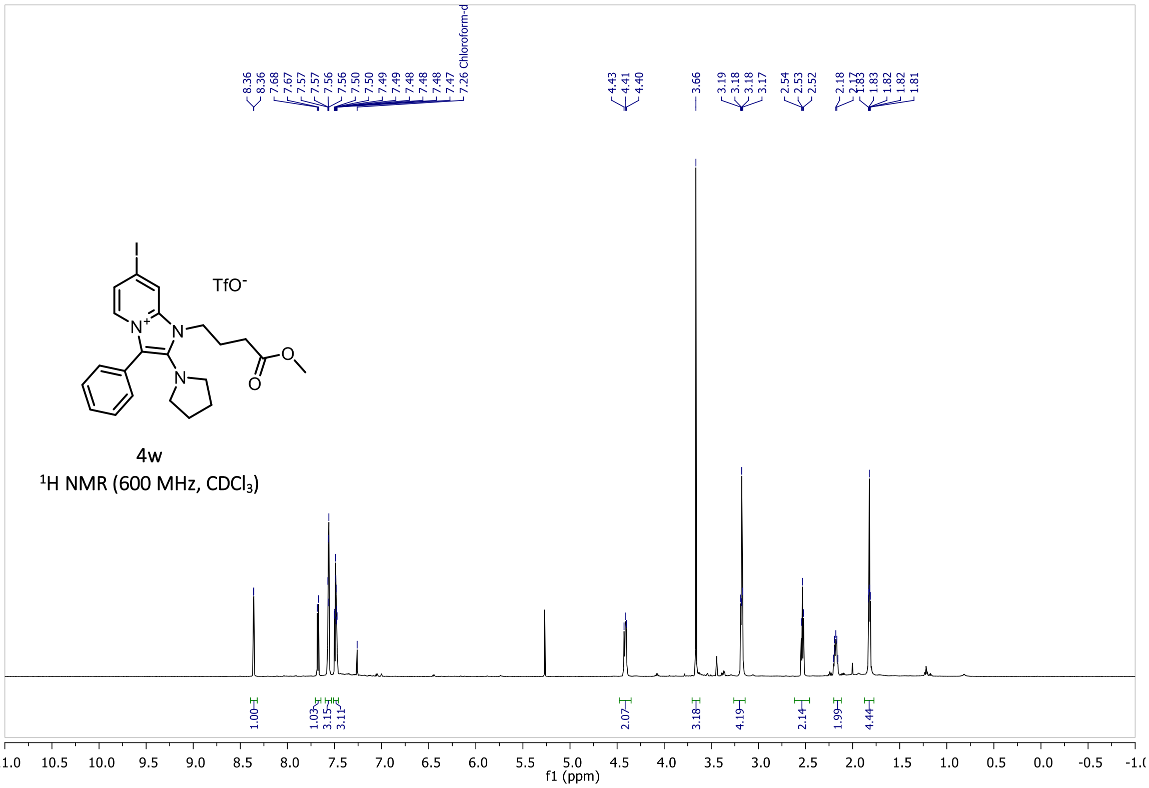

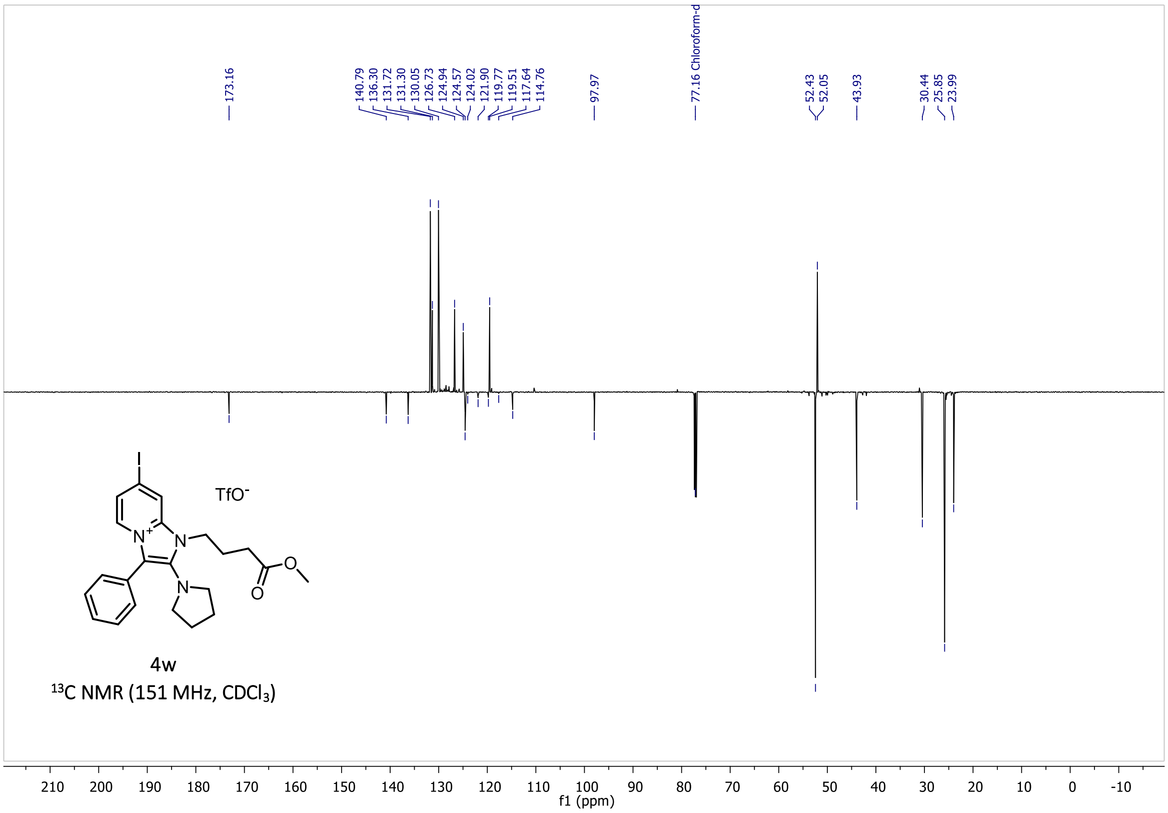
173.16
140.79
136.30
131.72
131.30
130.05
126.73
124.94
124.57
124.02
121.90
119.77
119.51
117.64
114.76
97.97
77.16 Chloroform-d
52.43
52.05
43.93
30.44
25.85
23.99
TfO-
4w
^{13}C NMR (151 MHz, CDCl$_3$)
f1 (ppm)

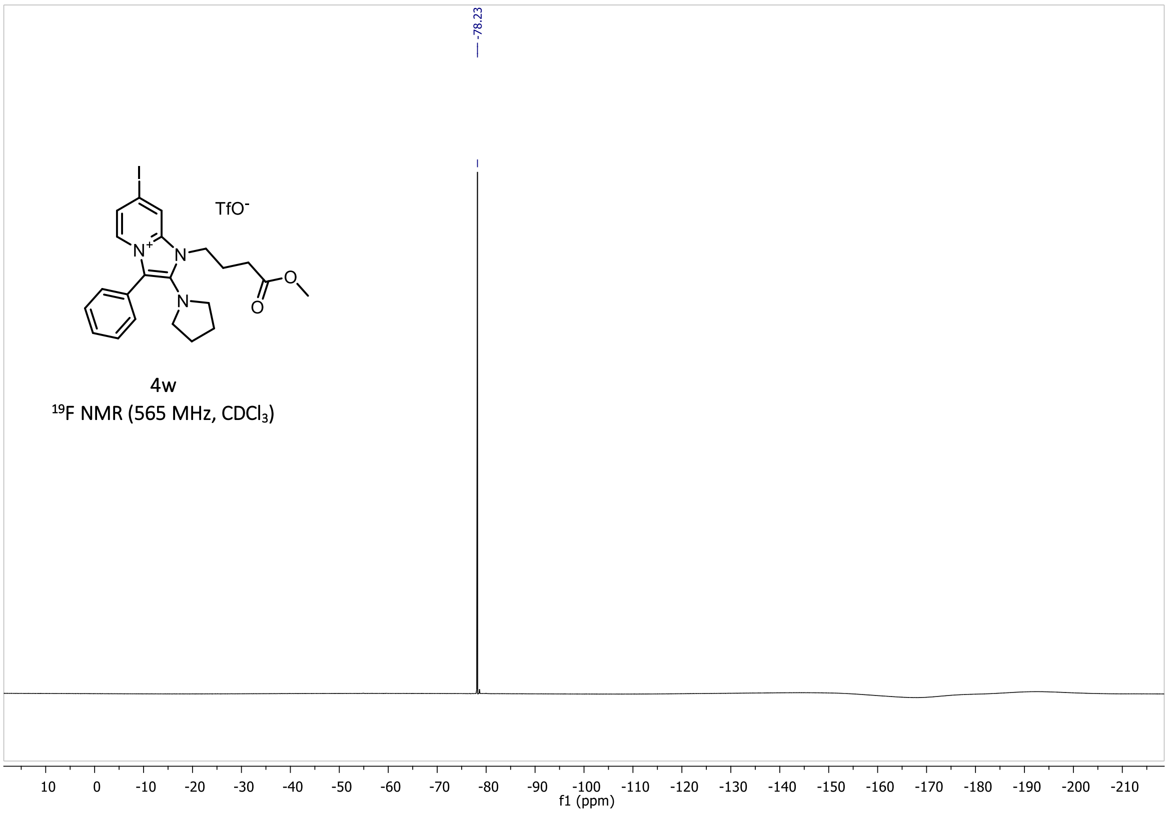
-78.23
TfO-
4w
^{19}F NMR (565 MHz, CDCl$_3$)
f1 (ppm)

1-(3-Azidopropyl)-7-iodo-3-phenyl-2-(pyrrolidin-1-yl)-1H-imidazo[1,2-a]pyridin-4-ium trifluoromethanesulfonate (4x)

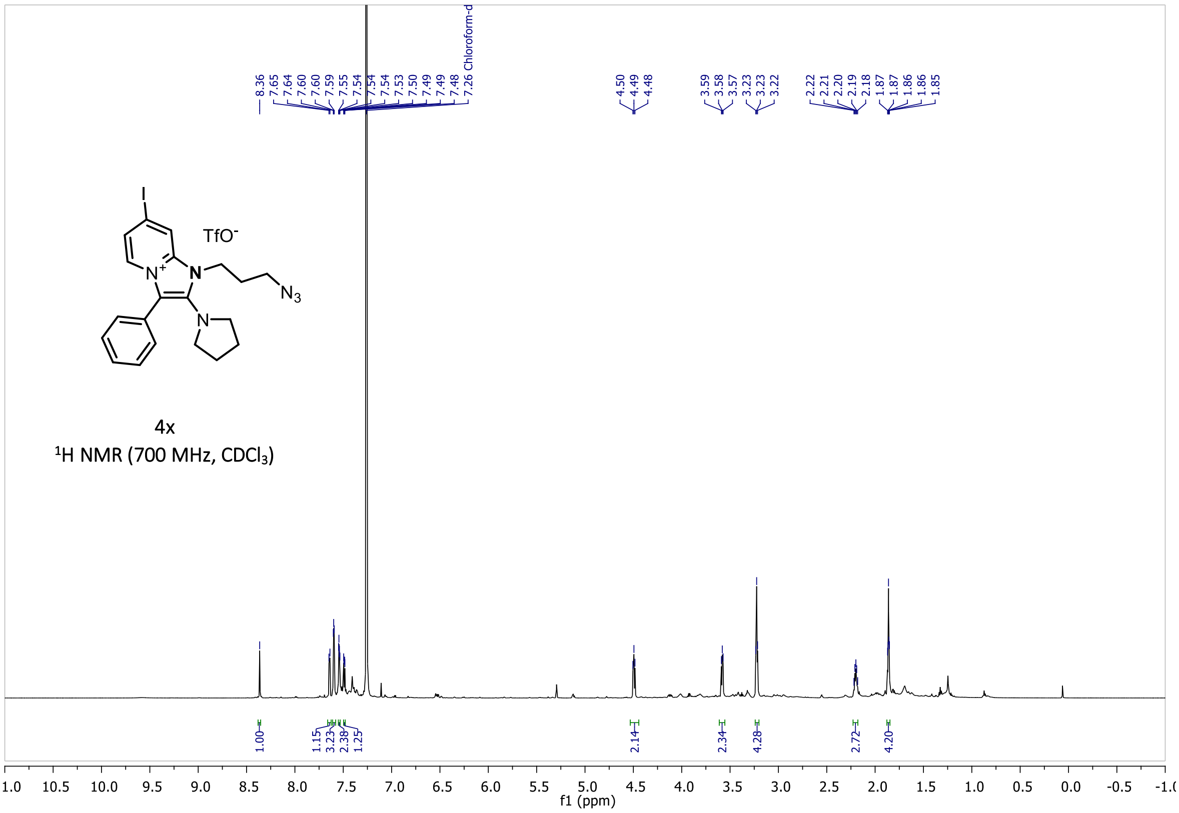

7-Iodo-1-phenethyl-3-phenyl-2-(pyrrolidin-1-yl)-1H-imidazo[1,2-a]pyridin-4-ium trifluoromethanesulfonate (4y)

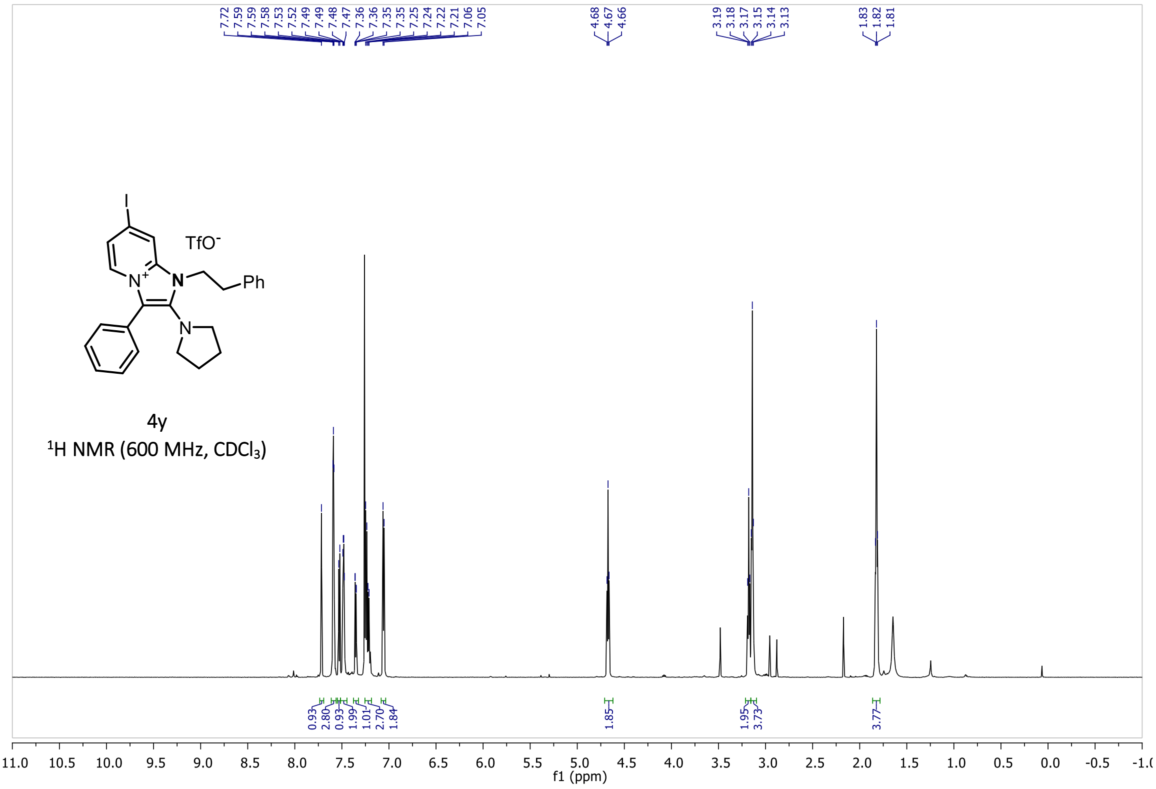

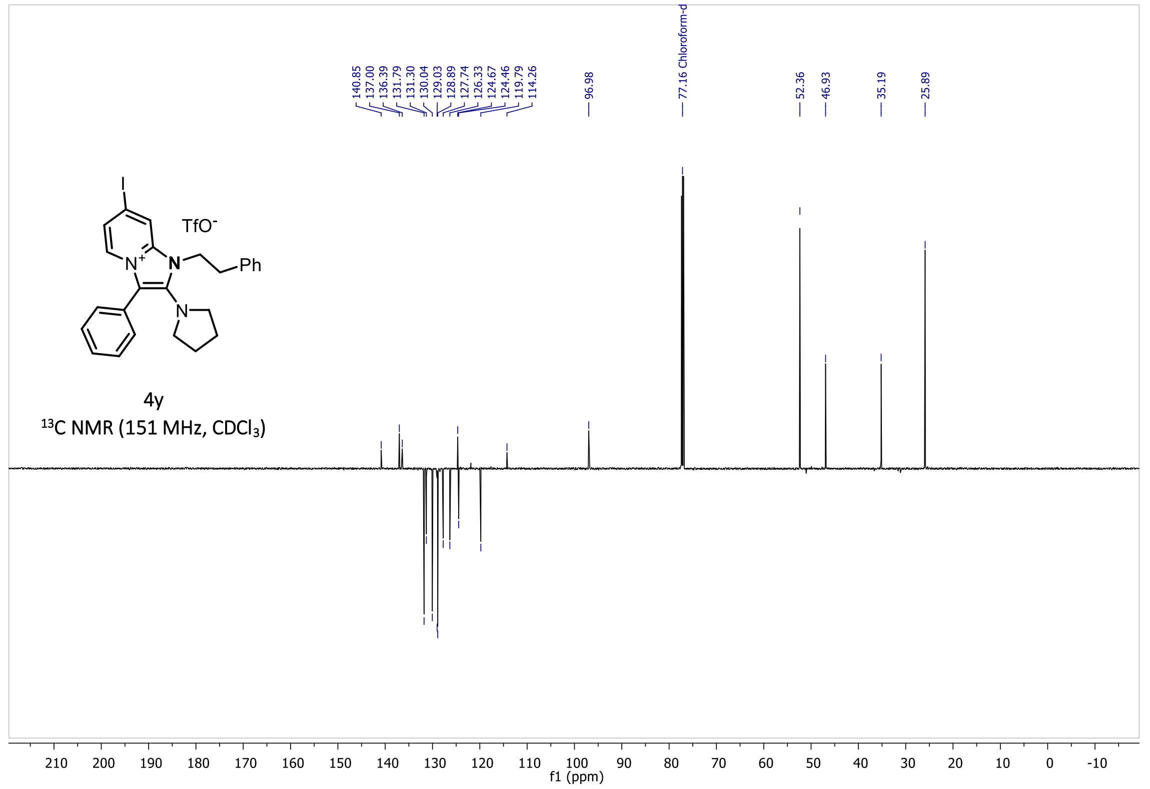

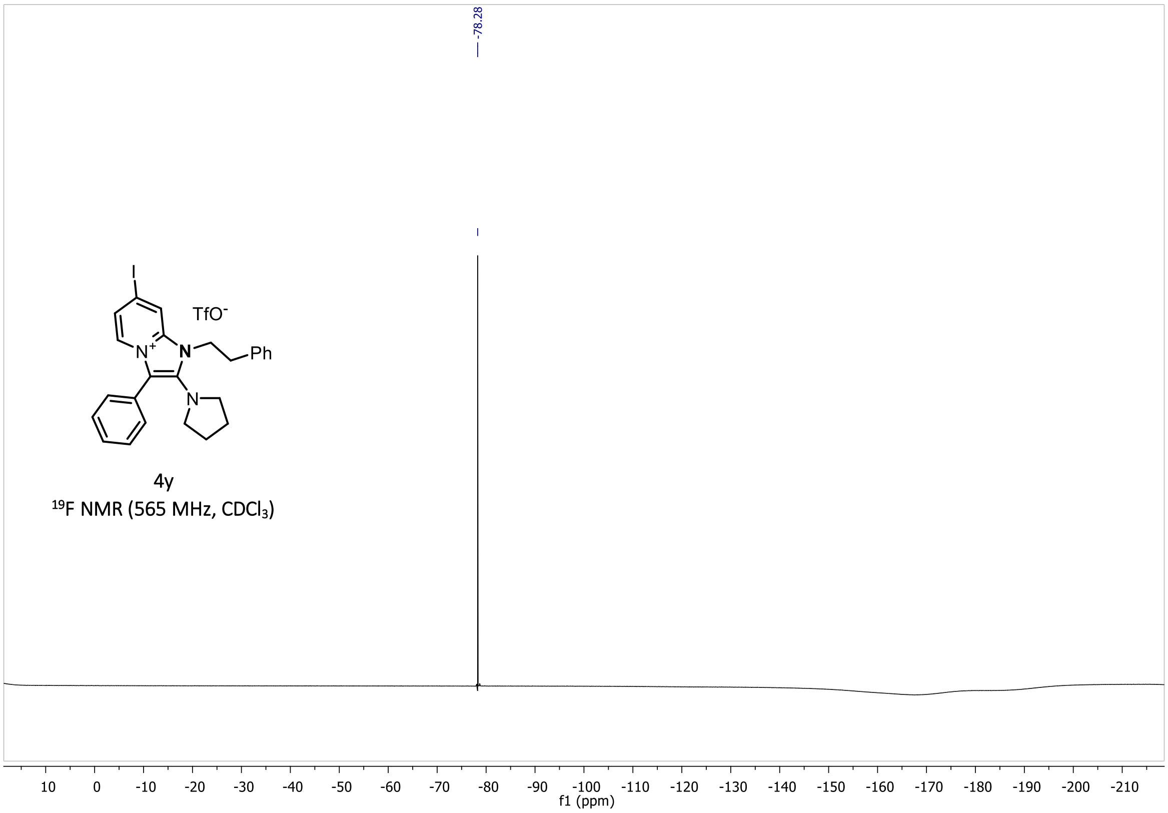

3-Heptyl-1-phenyl-2-(pyrrolidin-1-yl)-3H-imidazo[1,2-a]quinolin-10-ium trifluoromethanesulfonate (4z)

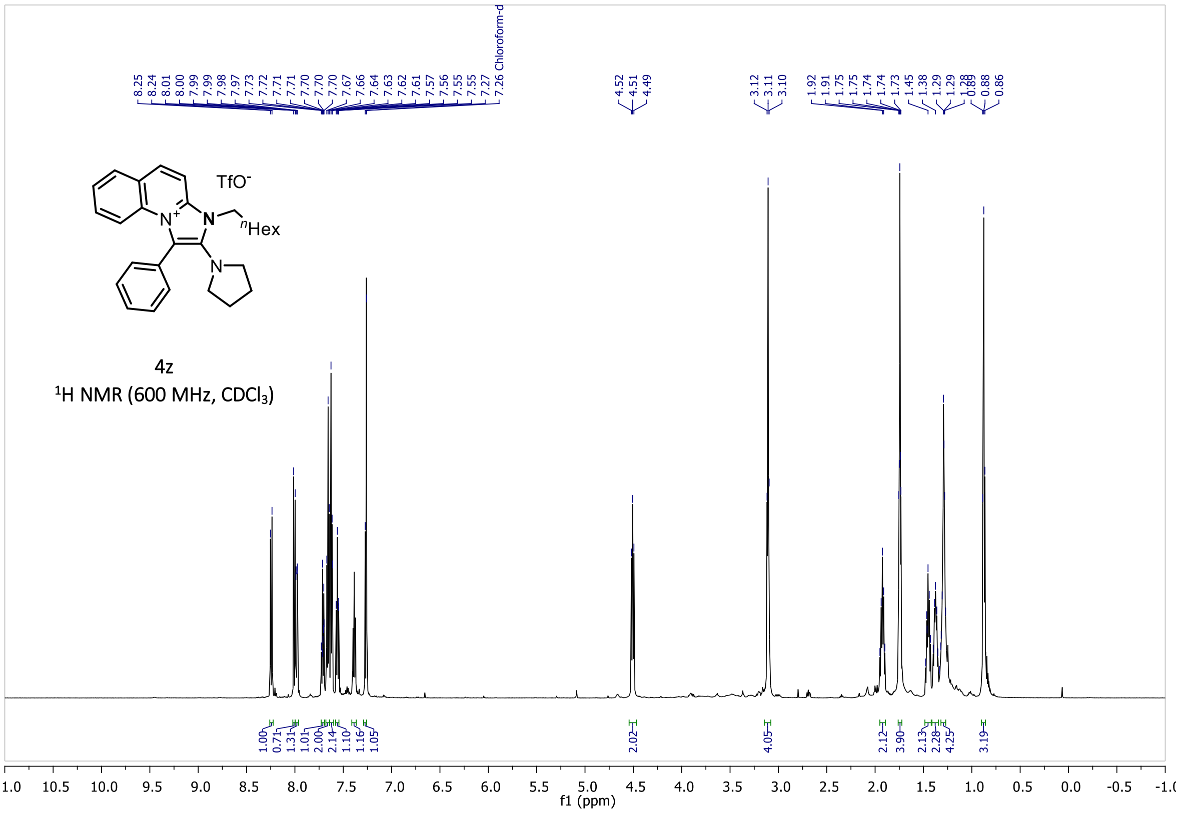

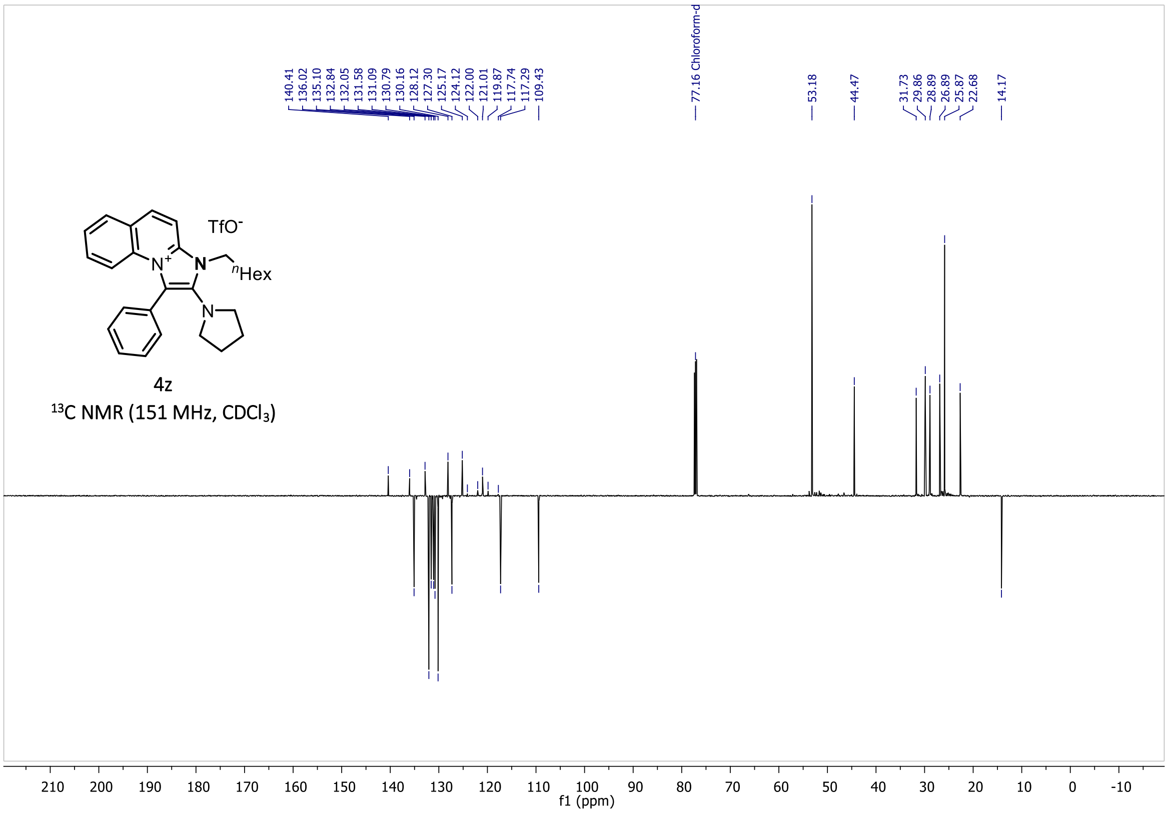
140.41
136.02
135.10
132.84
132.05
131.58
131.09
130.79
130.16
128.12
127.30
125.17
124.12
122.00
121.01
119.87
117.74
117.29
109.43
77.16 Chloroform-d
53.18
44.47
31.73
29.86
28.89
26.89
25.87
22.68
14.17
TfO⁻
N
N+
ⁿHex
N
4z
13C NMR (151 MHz, CDCl3)
210 200 190 180 170 160 150 140 130 120 110 100 90 80 70 60 50 40 30 20 10 0 -10
f1 (ppm)

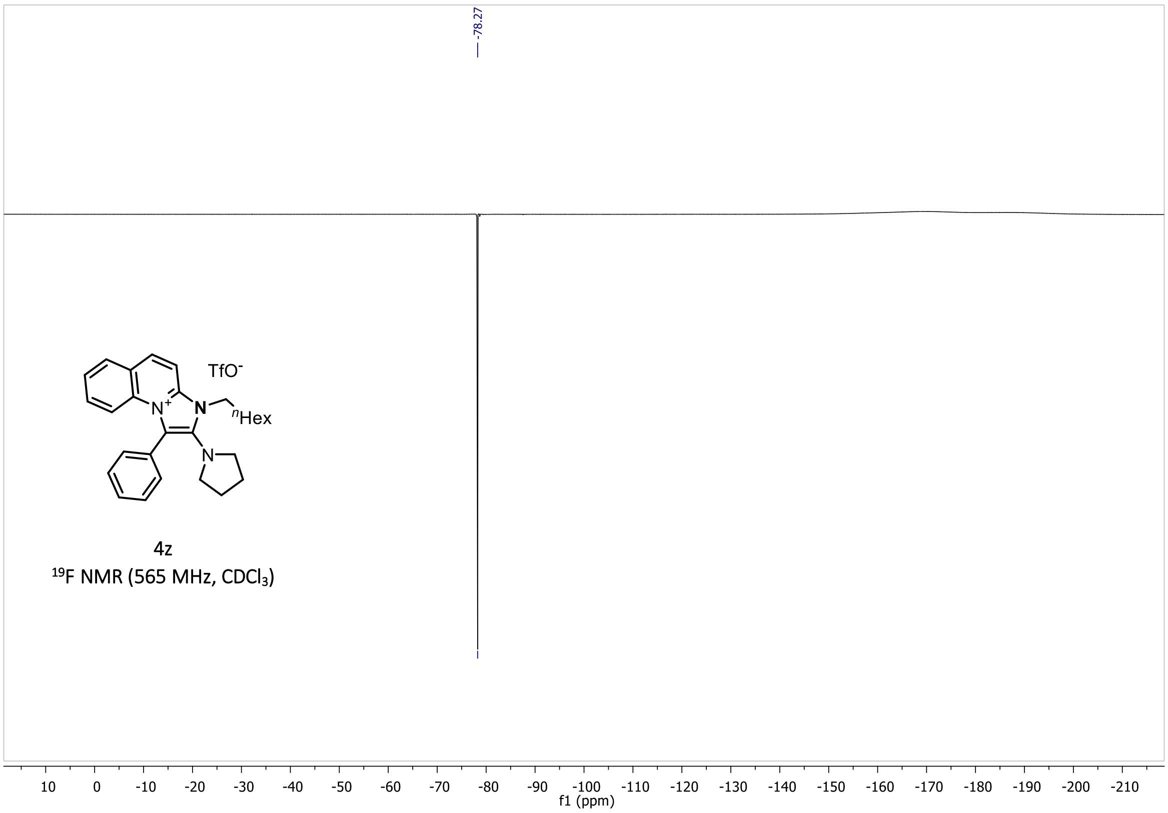
-78.27
TfO⁻
N
N+
ⁿHex
N
4z
19F NMR (565 MHz, CDCl3)
10 0 -10 -20 -30 -40 -50 -60 -70 -80 -90 -100 -110 -120 -130 -140 -150 -160 -170 -180 -190 -200 -210
f1 (ppm)

3-(8-(Benzyloxy)-8-oxooctyl)-1-phenyl-2-(pyrrolidin-1-yl)-3H-imidazo[1,2-a]quinolin-10-ium trifluoromethanesulfonate (4aa)

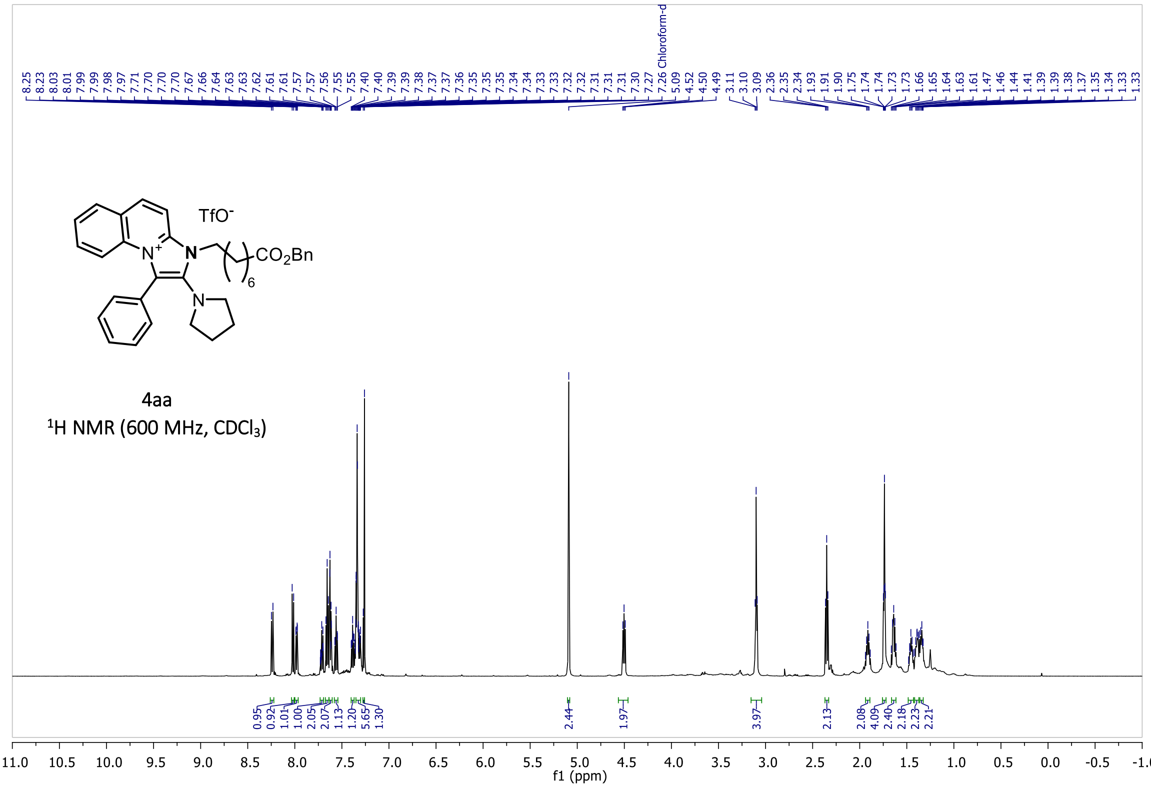

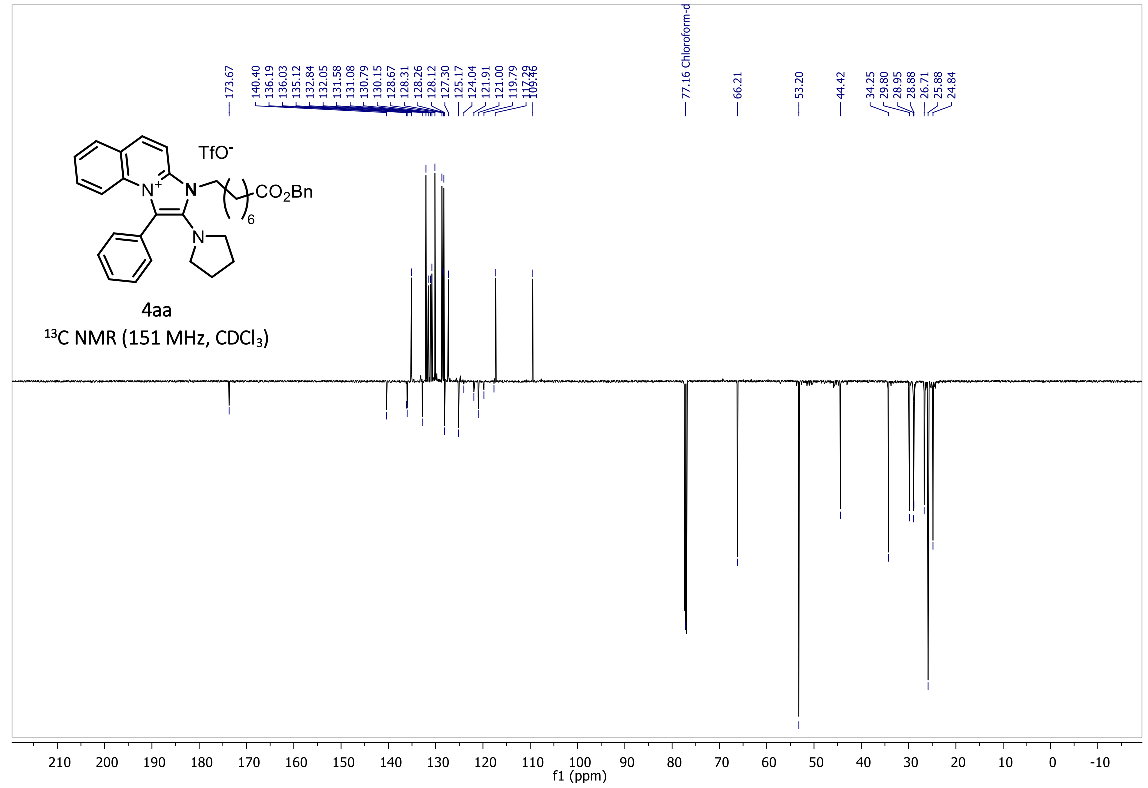

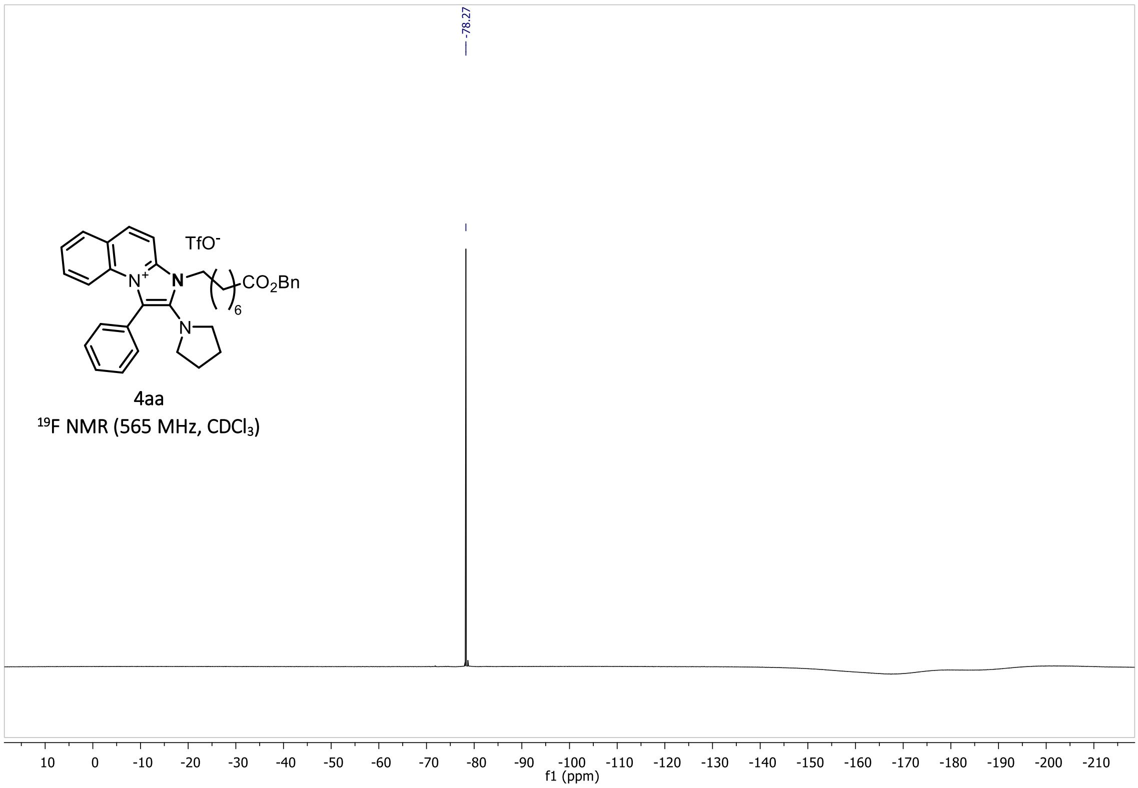

1-(Cyclohexylmethyl)-3-phenyl-2-(pyrrolidin-1-yl)-1H-imidazo[2,1-a]isoquinolin-4-ium trifluoromethanesulfonate (4ab)

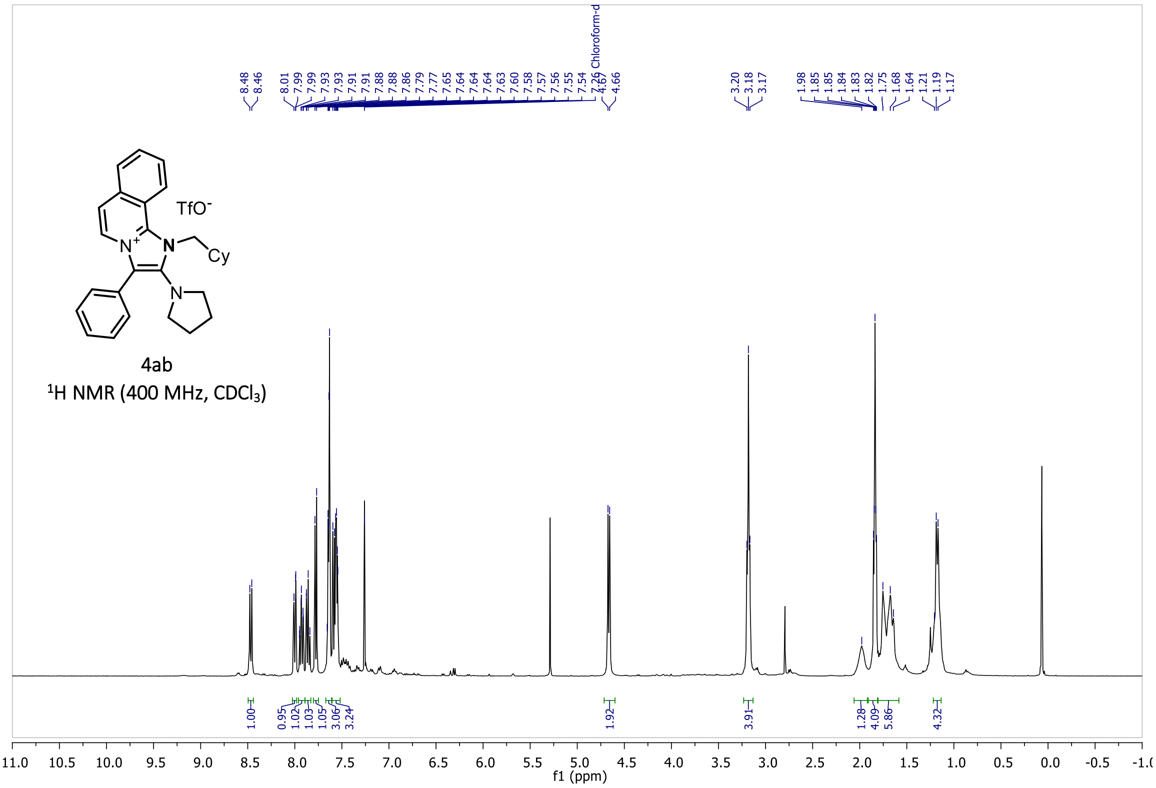

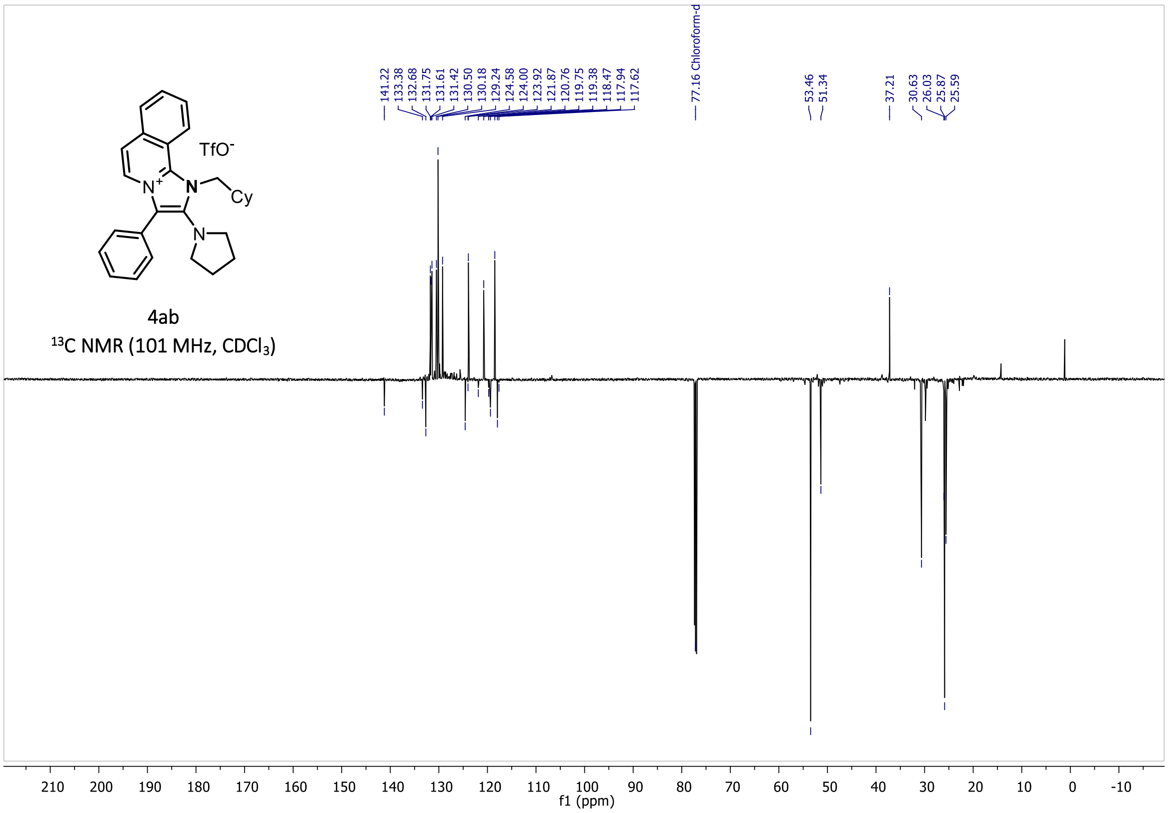
141.22
133.38
132.68
131.75
131.61
131.42
130.50
130.18
129.24
124.58
124.00
123.92
121.87
120.76
119.75
119.38
118.47
117.94
117.62
77.16 Chloroform-d
53.46
51.34
37.21
30.63
26.03
25.87
25.59
TfO⁻
Cy
4ab
^{13}C NMR (101 MHz, $CDCl_3$)
f1 (ppm)

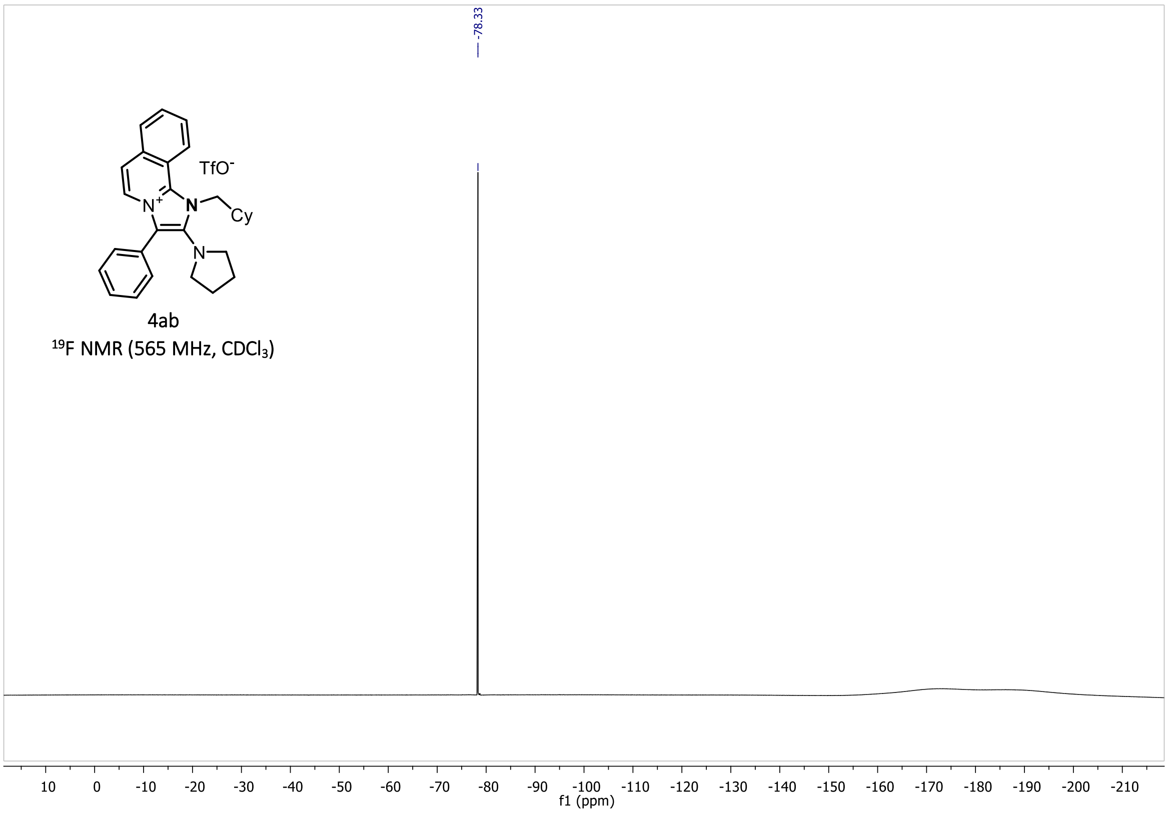
-78.33
TfO⁻
Cy
4ab
^{19}F NMR (565 MHz, $CDCl_3$)
f1 (ppm)

3-Phenyl-2-(pyrrolidin-1-yl)oxazolo[3,2-a]pyridin-4-ium trifluoromethanesulfonate (5a)

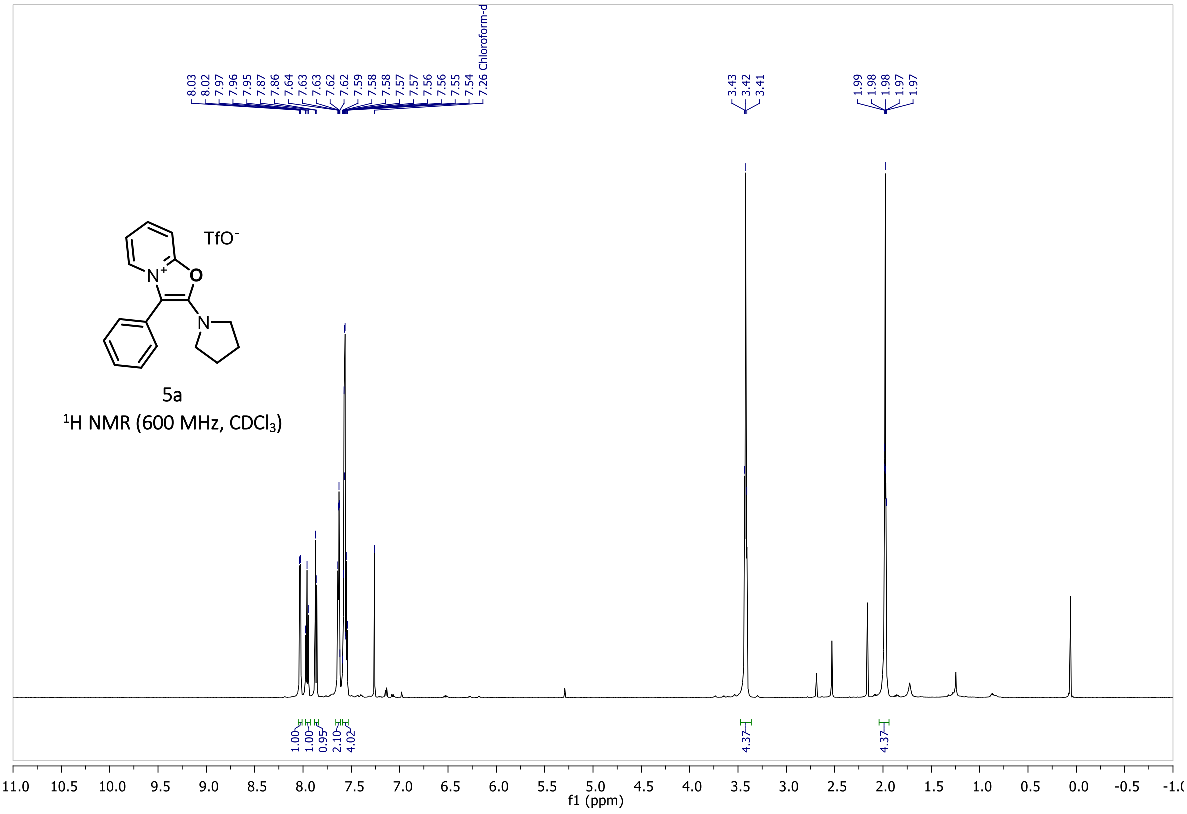

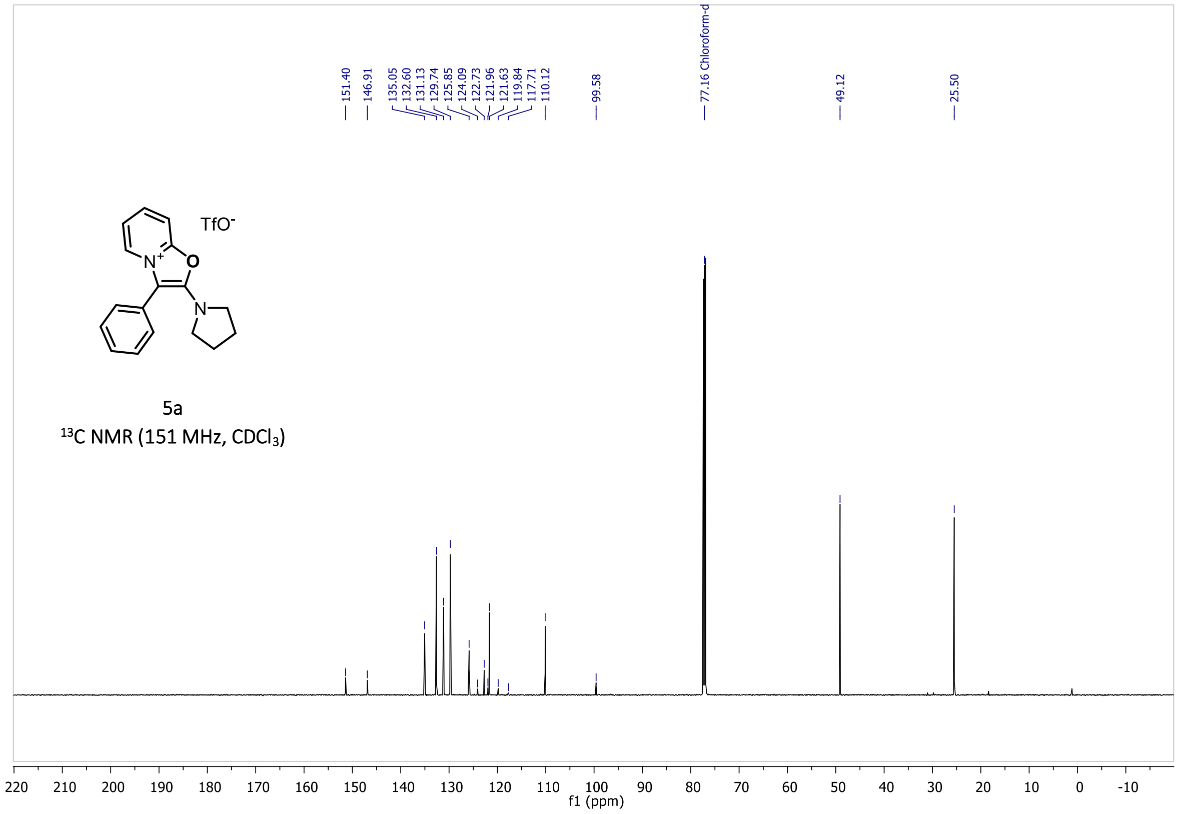

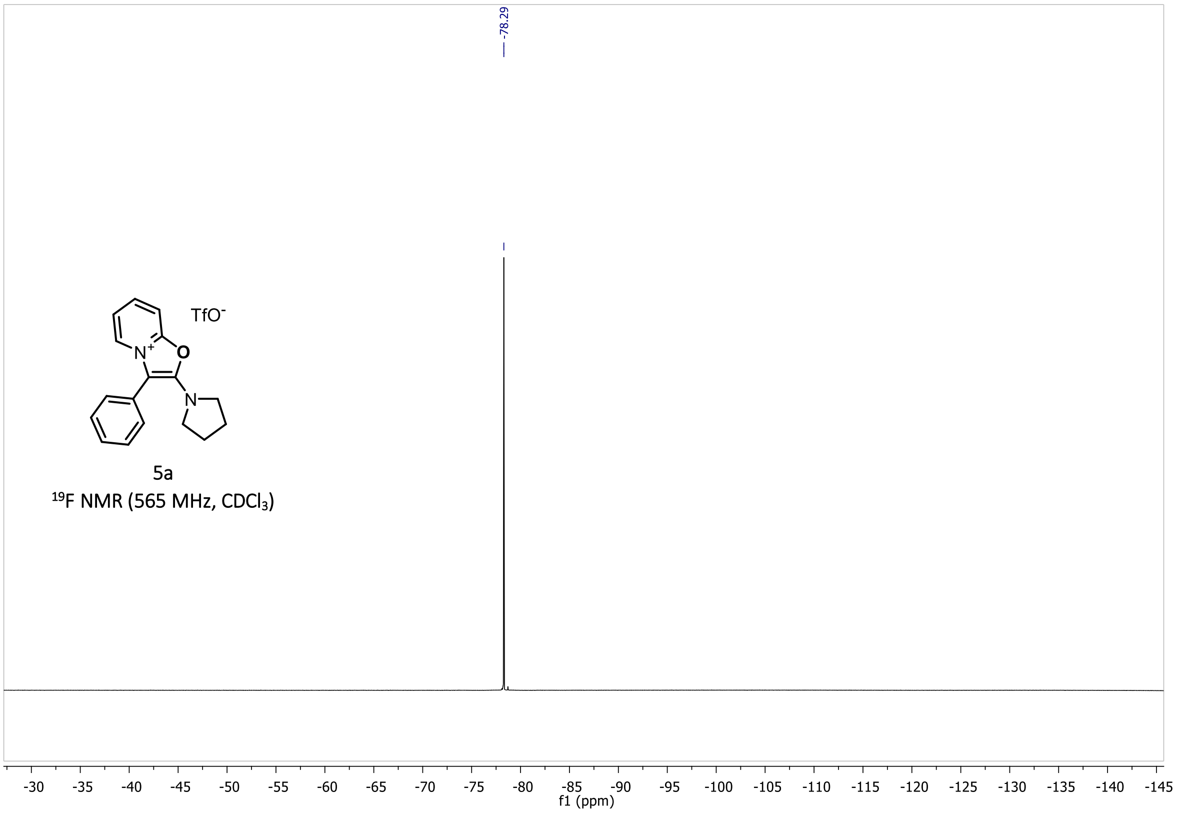

3-(4-Methoxyphenyl)-2-(pyrrolidin-1-yl)oxazolo[3,2-a]pyridin-4-ium trifluoromethanesulfonate (5b)

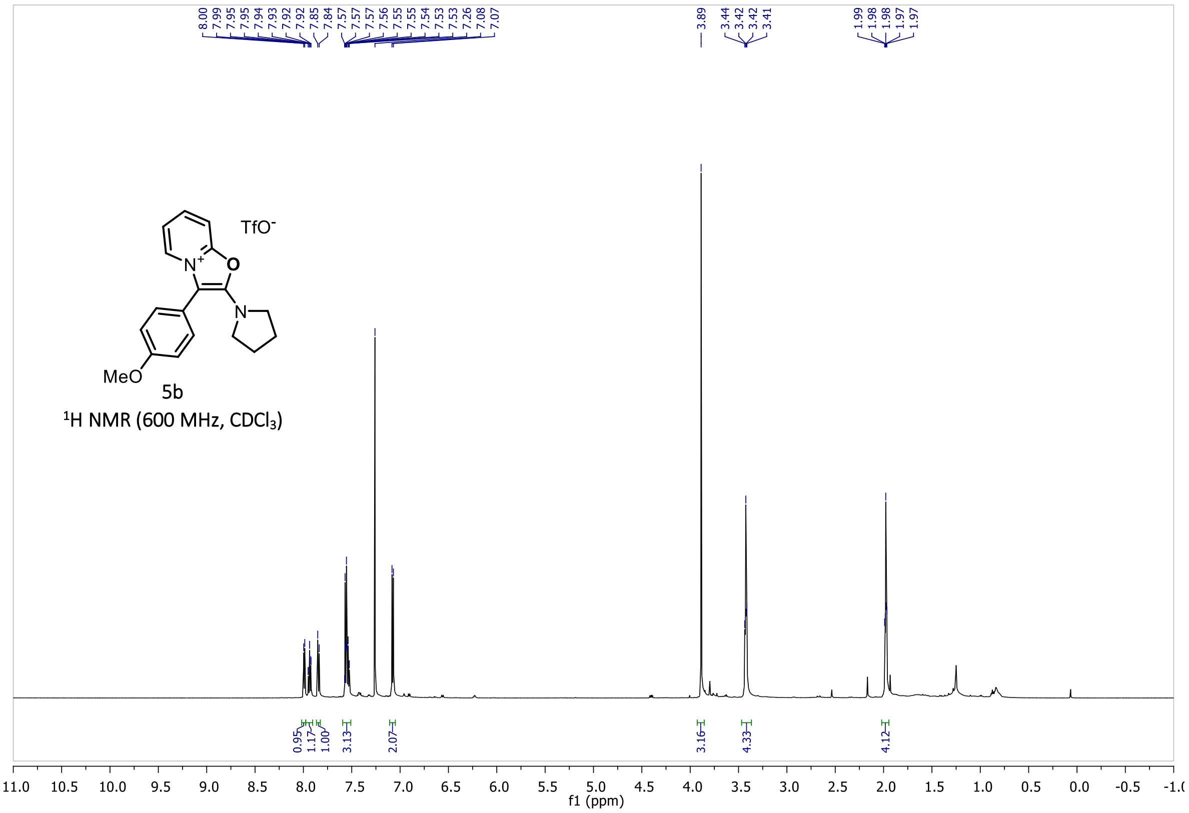

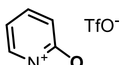

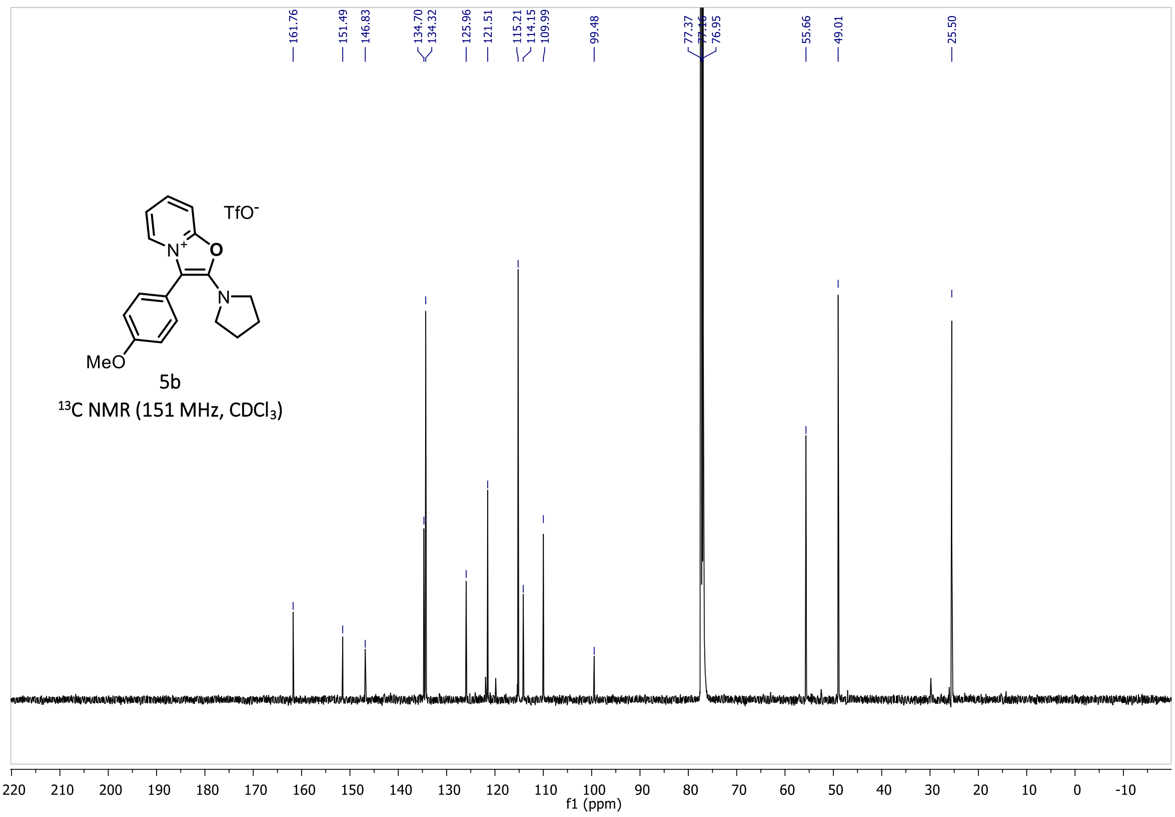

5b

^{13}C NMR (151 MHz, $CDCl_3$)

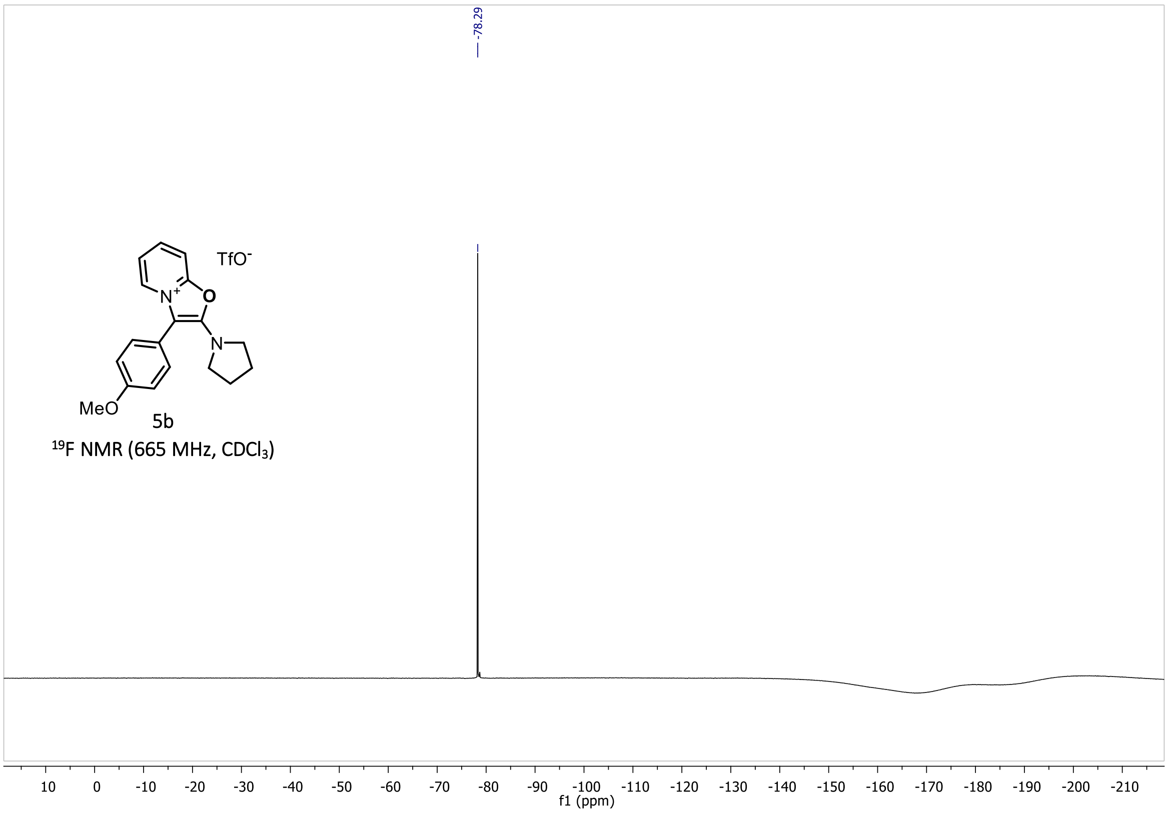

5b

^{19}F NMR (665 MHz, $CDCl_3$)

3-(4-Nitrophenyl)-2-(pyrrolidin-1-yl)oxazolo[3,2-a]pyridin-4-ium trifluoromethanesulfonate (5c)

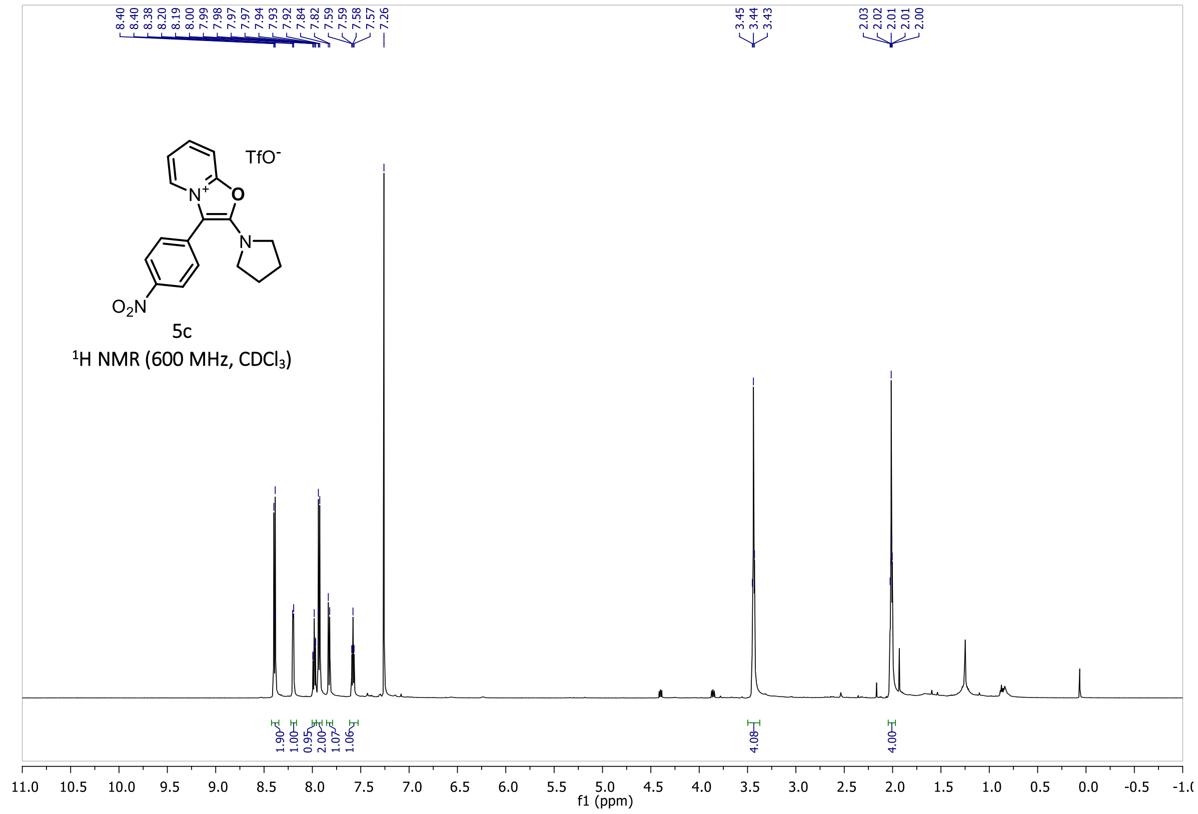

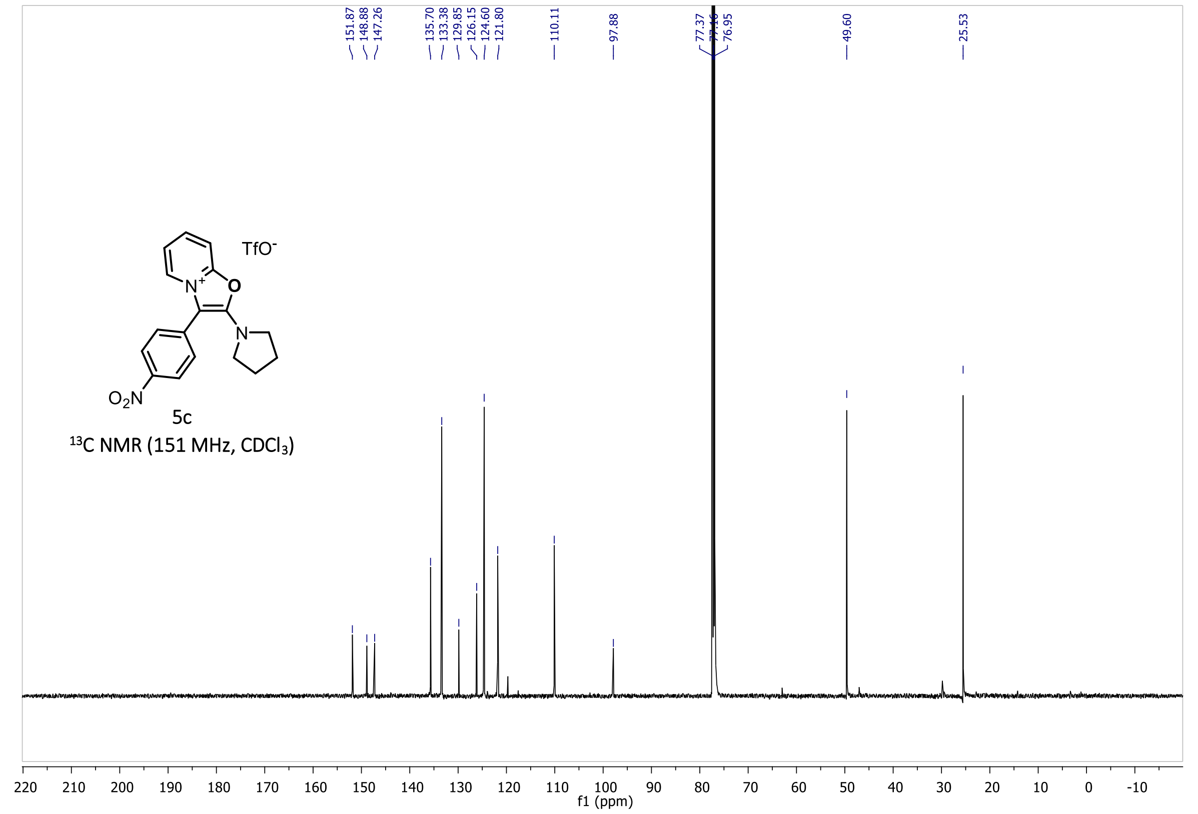

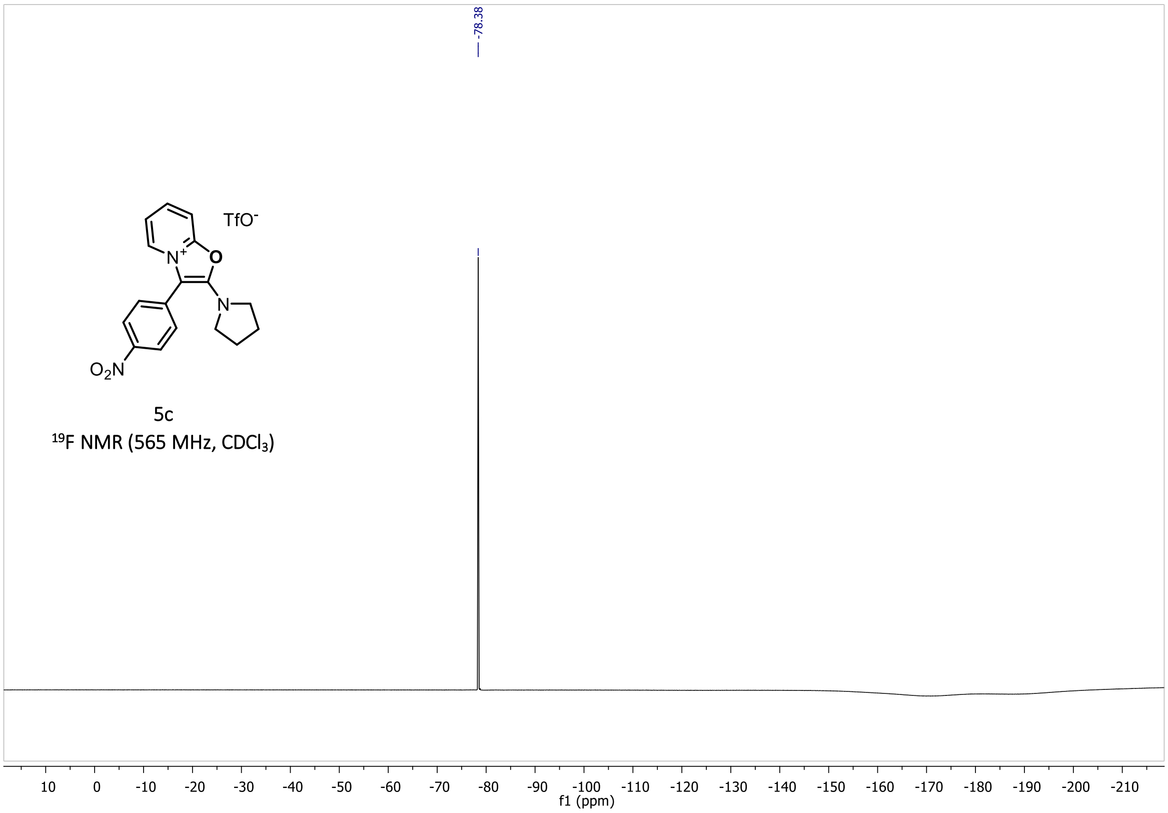

5c
^{19}F NMR (565 MHz, $CDCl_3$)

3-(4-Iodophenyl)-2-(pyrrolidin-1-yl)oxazolo[3,2-a]pyridin-4-ium trifluoromethanesulfonate (5d)

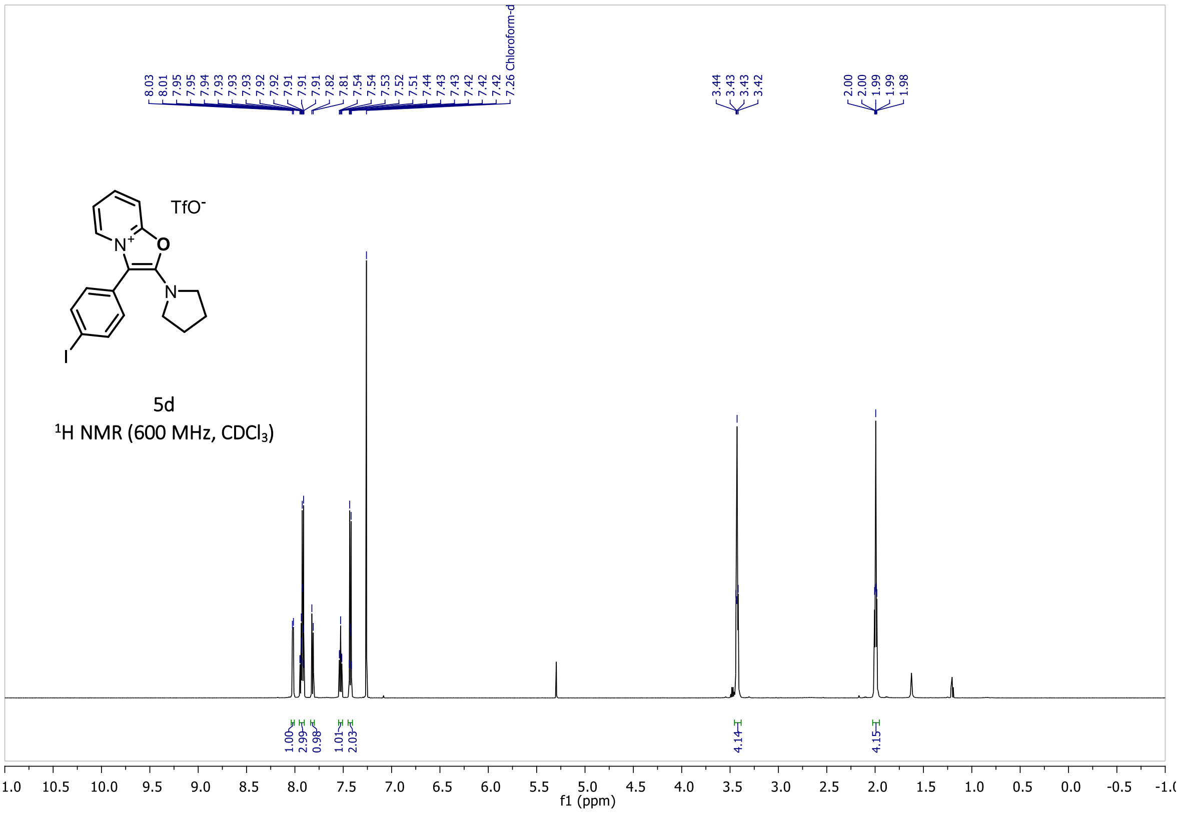

5d
^{1}H NMR (600 MHz, $CDCl_3$)

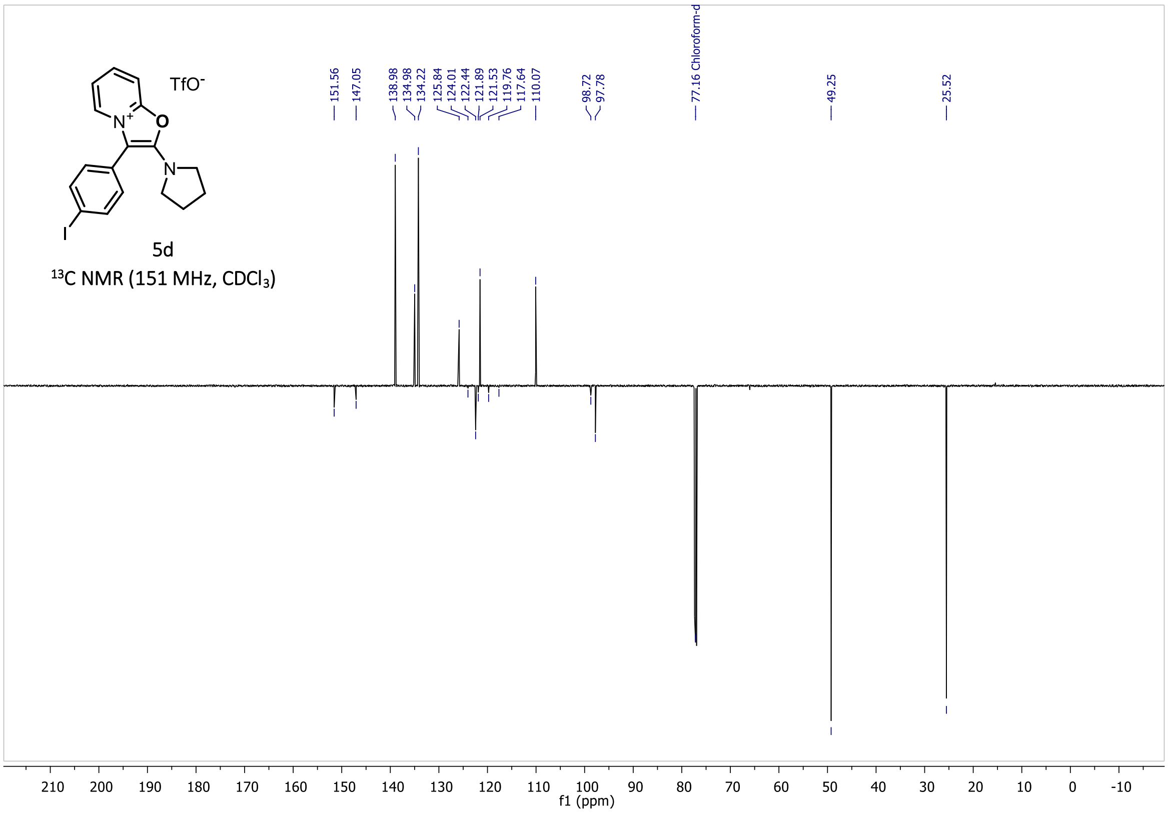
TfO-
N+
O
N
I
5d
^{13}C NMR (151 MHz, CDCl$_3$)
151.56
147.05
138.98
134.98
134.22
125.84
124.01
122.44
121.89
121.53
119.76
117.64
110.07
98.72
97.78
77.16 Chloroform-d
49.25
25.52
210 200 190 180 170 160 150 140 130 120 110 100 90 80 70 60 50 40 30 20 10 0 -10
f1 (ppm)

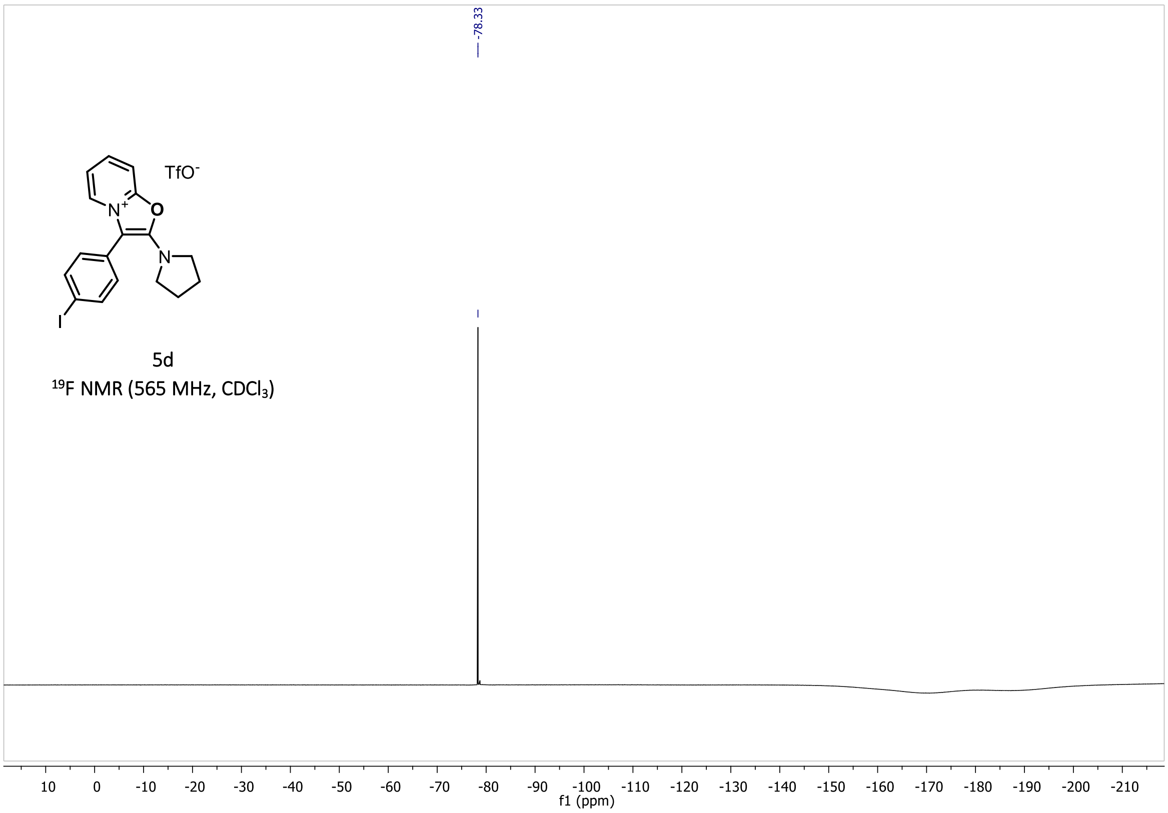
-78.33
TfO-
N+
O
N
I
5d
^{19}F NMR (565 MHz, CDCl$_3$)
10 0 -10 -20 -30 -40 -50 -60 -70 -80 -90 -100 -110 -120 -130 -140 -150 -160 -170 -180 -190 -200 -210
f1 (ppm)

3-(2-Chlorophenyl)-2-(pyrrolidin-1-yl)oxazolo[3,2-a]pyridin-4-ium trifluoromethanesulfonate (5e)

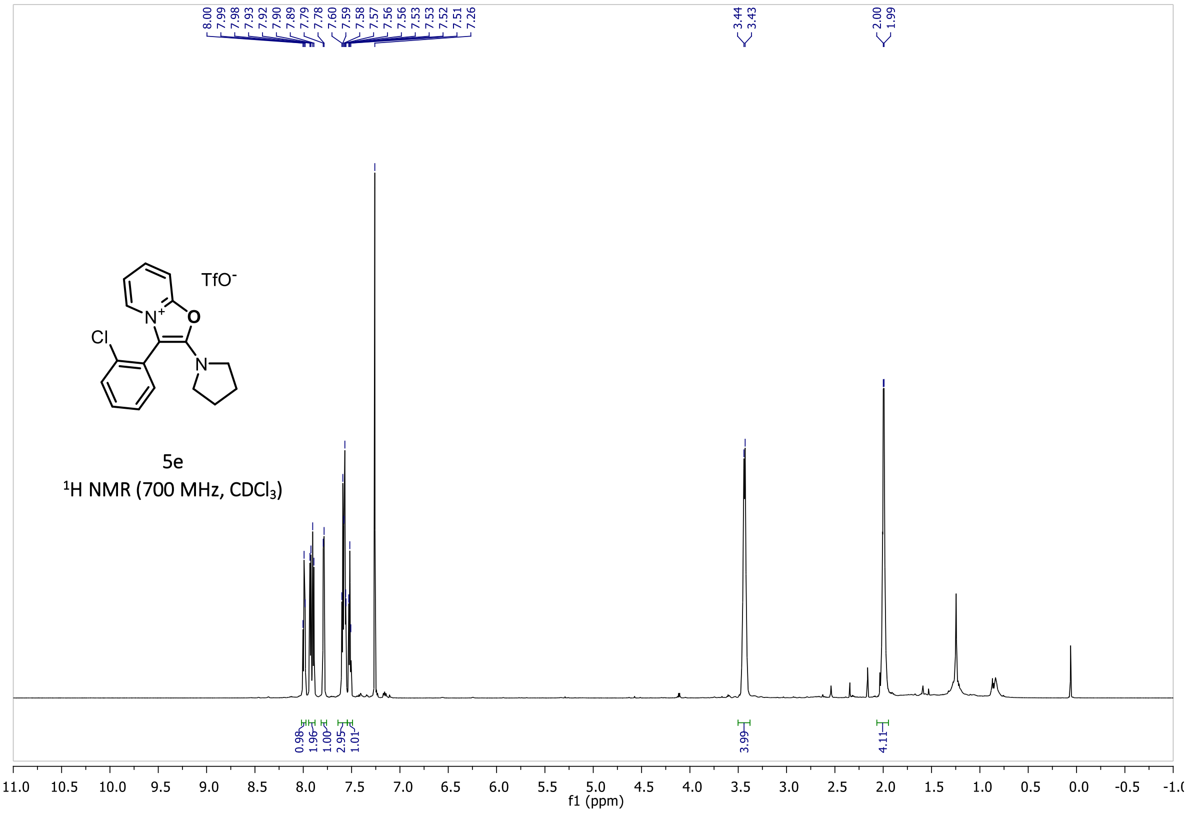

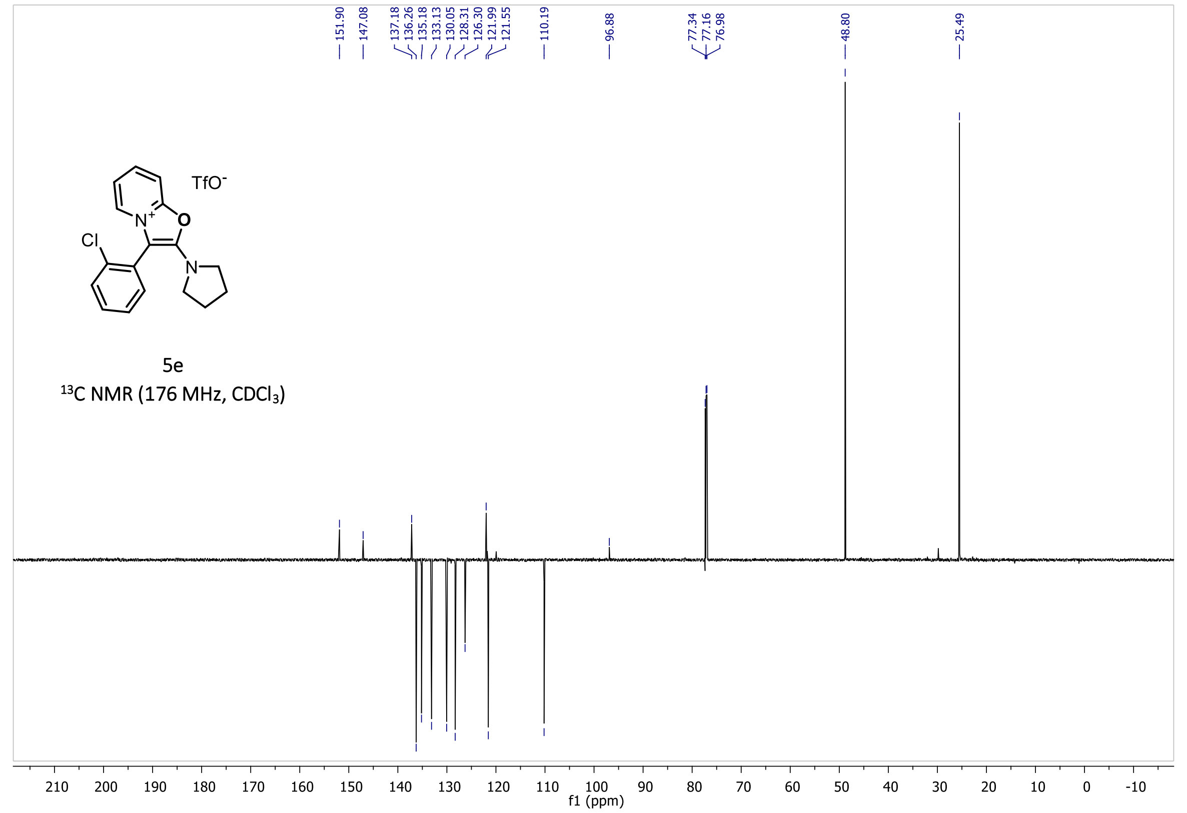

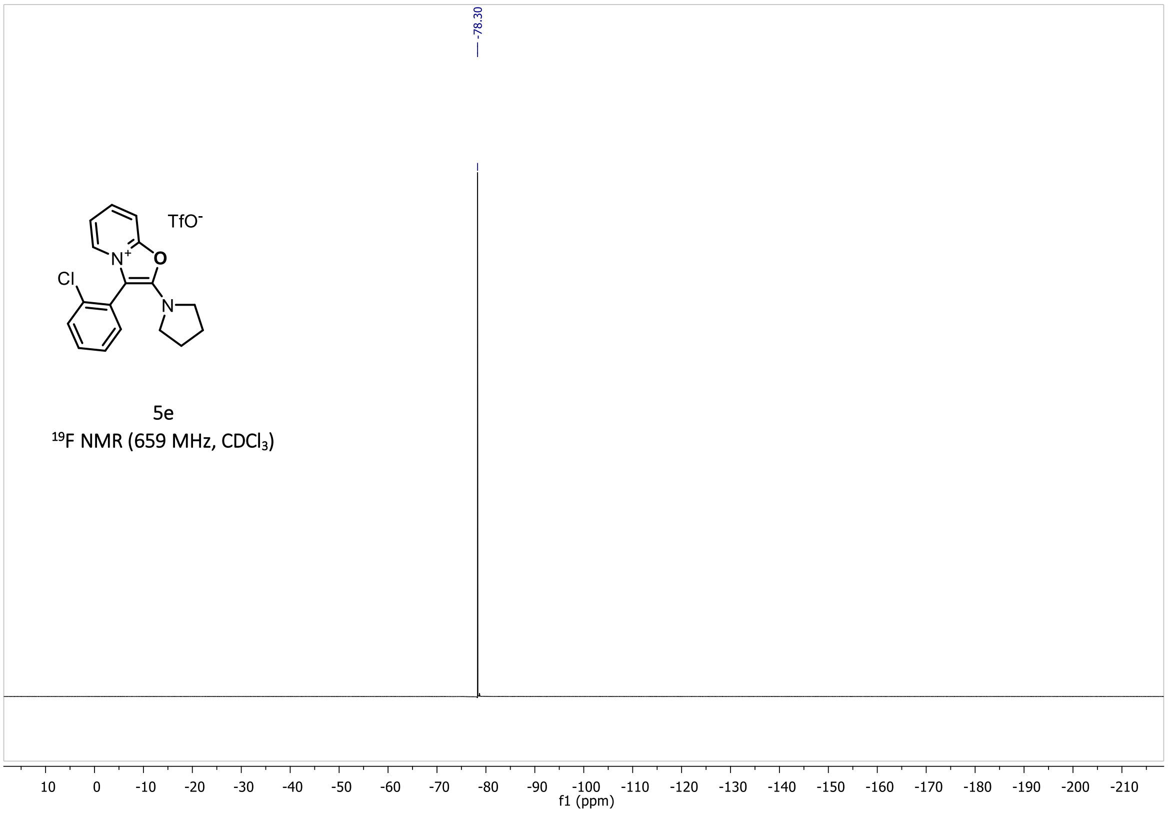

7-Iodo-3-phenyl-2-(pyrrolidin-1-yl)oxazolo[3,2-a]pyridin-4-ium trifluoromethanesulfonate (5f)

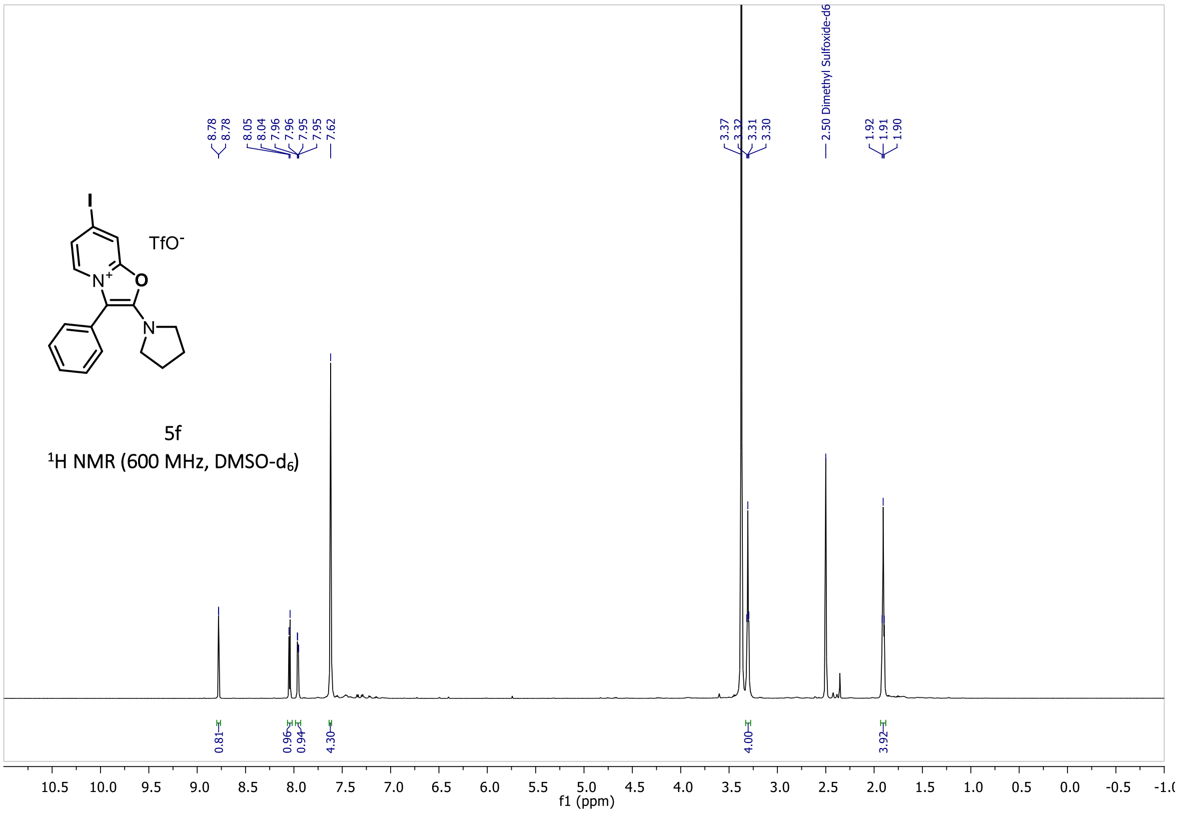

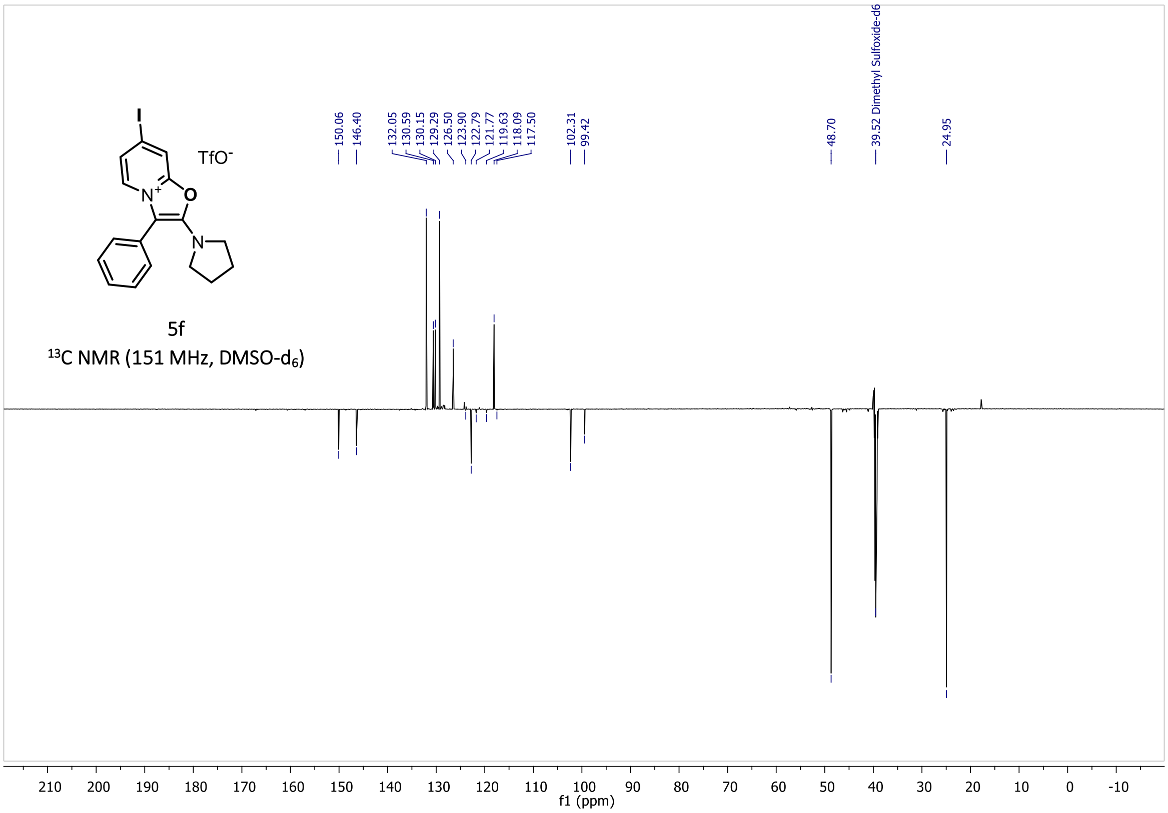

5f

^{13}C NMR (151 MHz, DMSO-d_6)

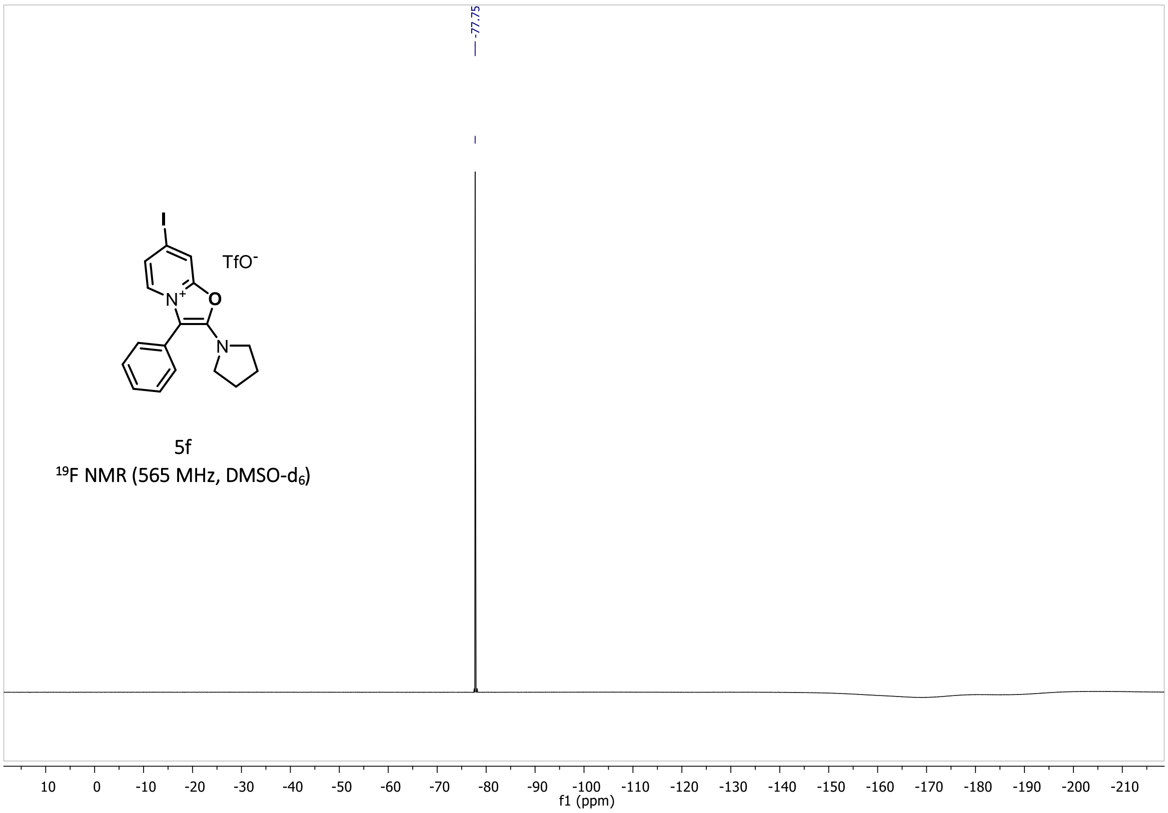

5f

^{19}F NMR (565 MHz, DMSO-d_6)

3-Phenyl-2-(pyrrolidin-1-yl)thiazolo[3,2-a]pyridin-4-ium trifluoromethanesulfonate (6a)

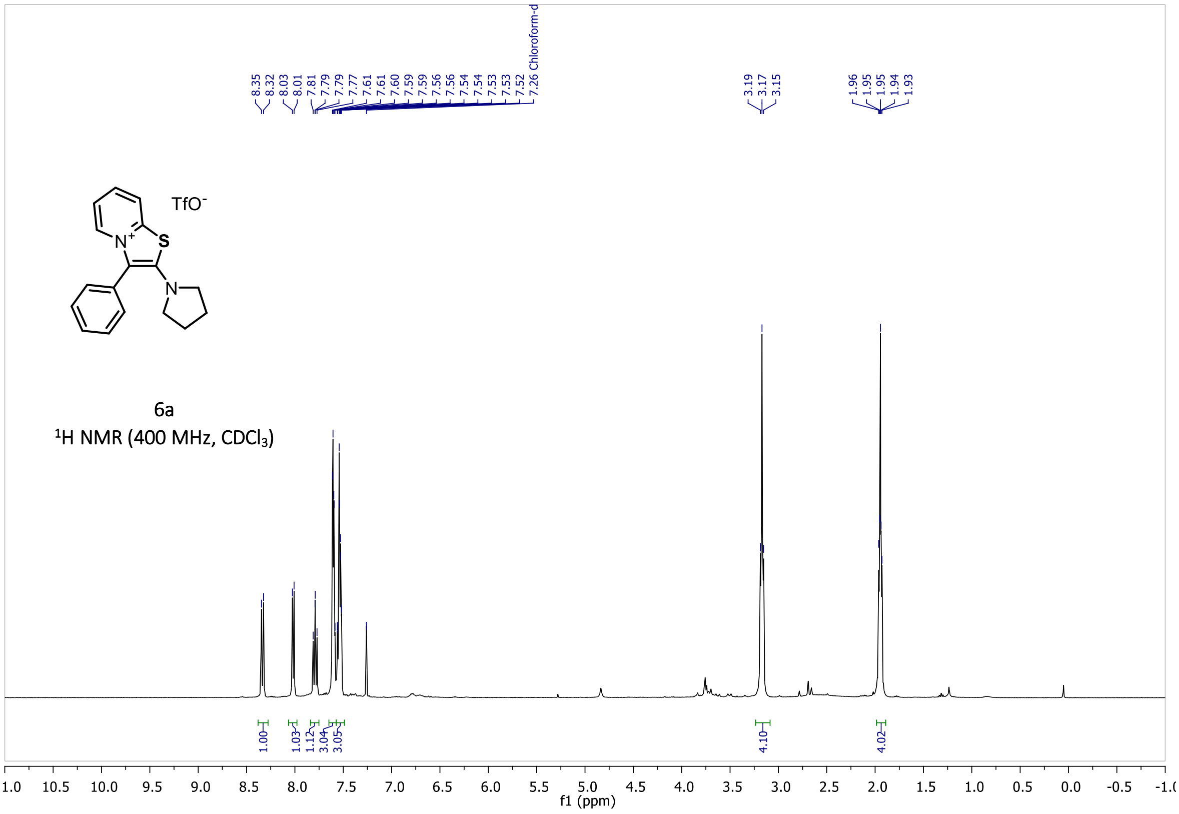

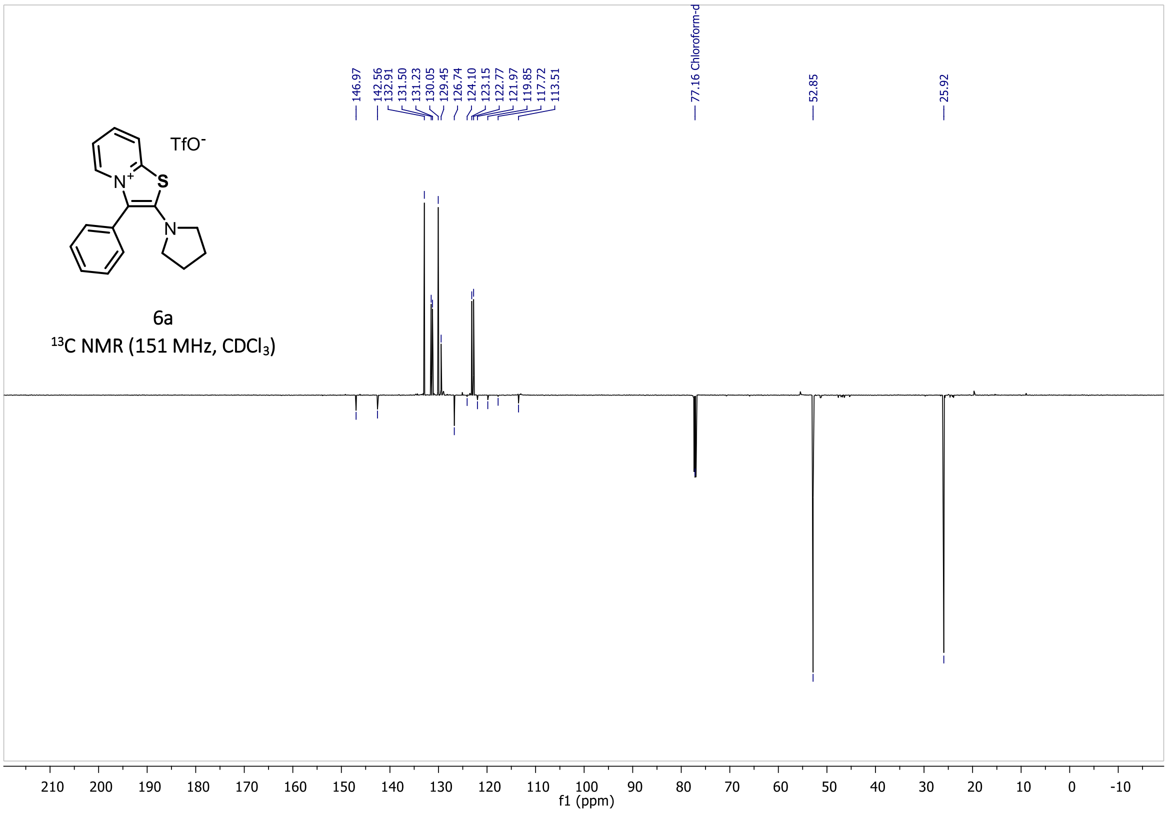

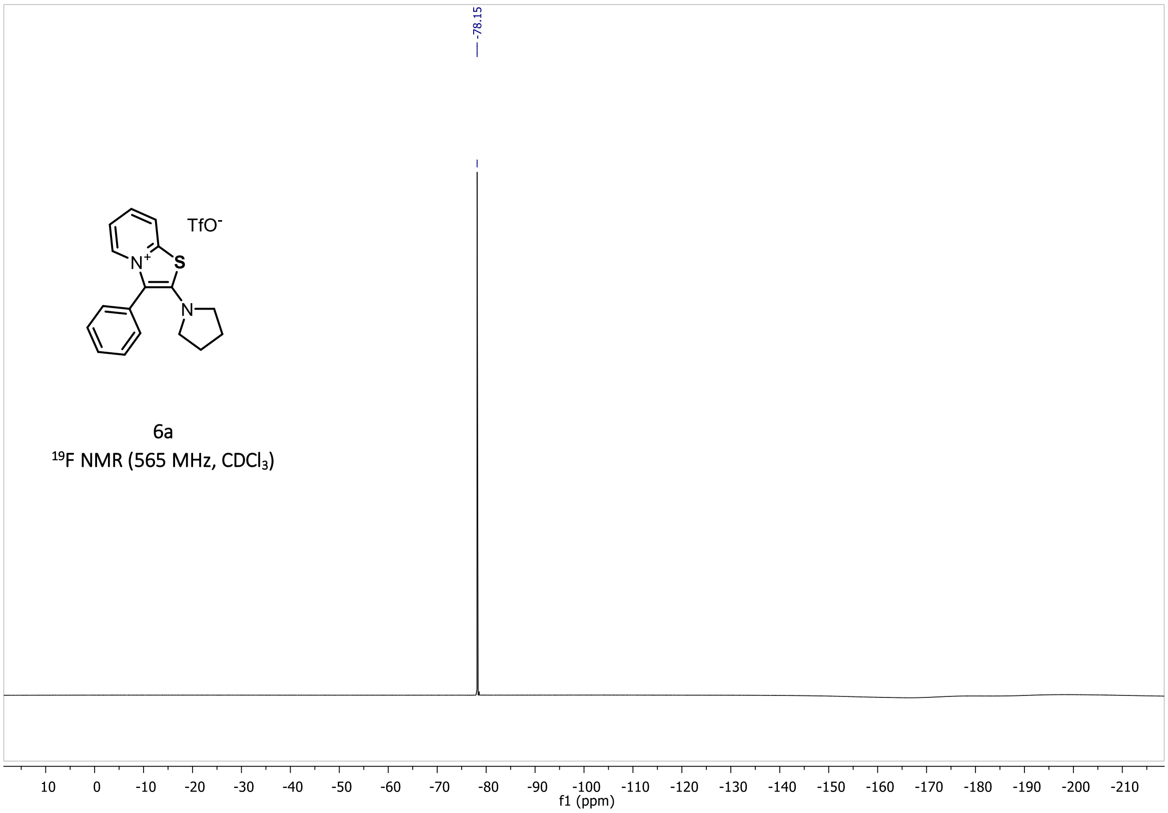

7-Iodo-3-phenyl-2-(pyrrolidin-1-yl)thiazolo[3,2-a]pyridin-4-ium trifluoromethanesulfonate (6b)

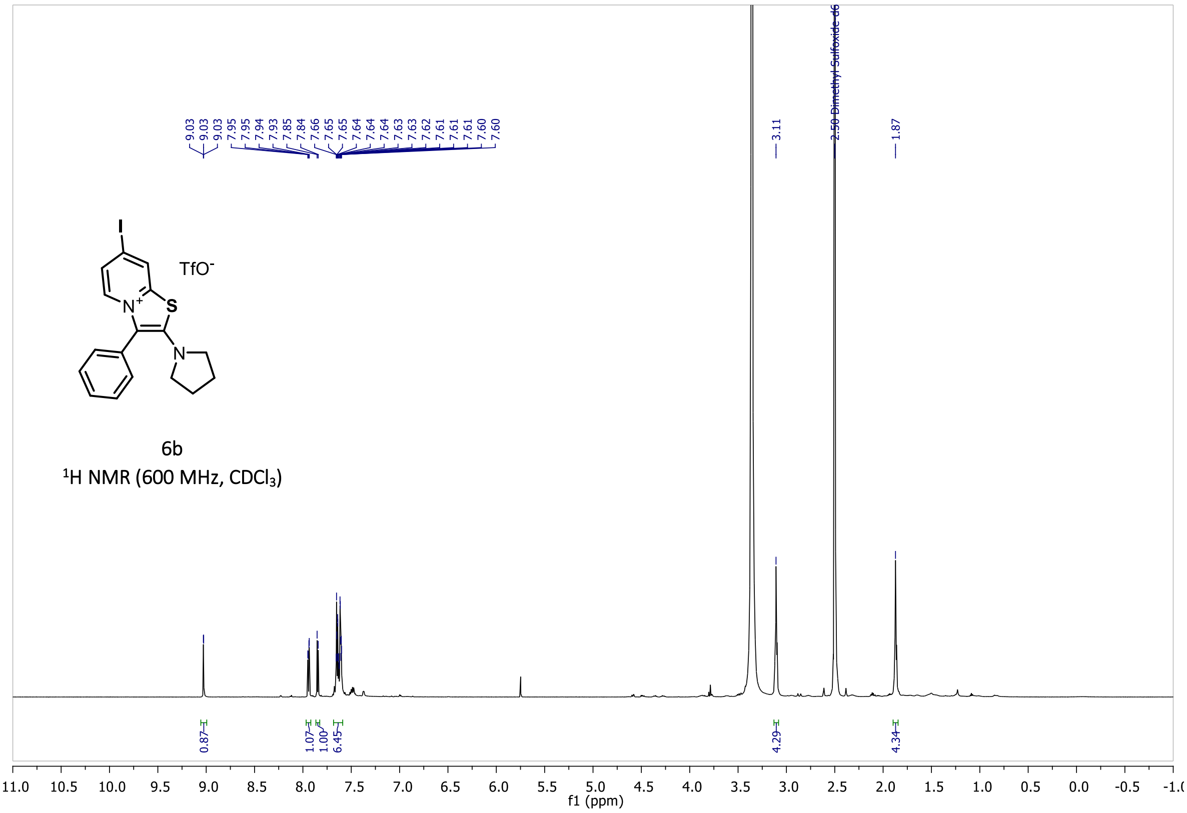

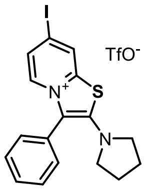

6b

^{13}C NMR (151 MHz, $CDCl_3$)

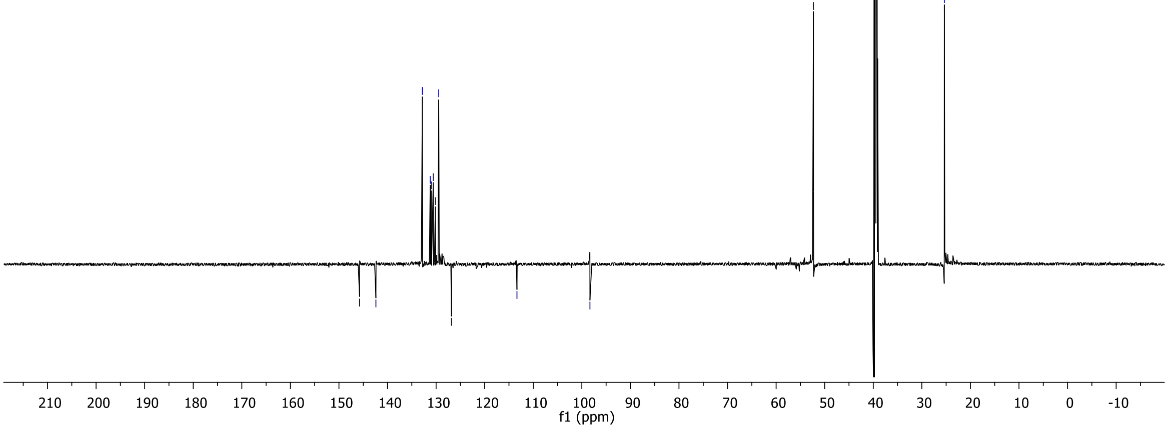

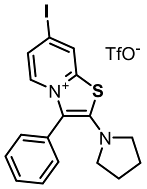

6b

^{19}F NMR (565 MHz, $CDCl_3$)

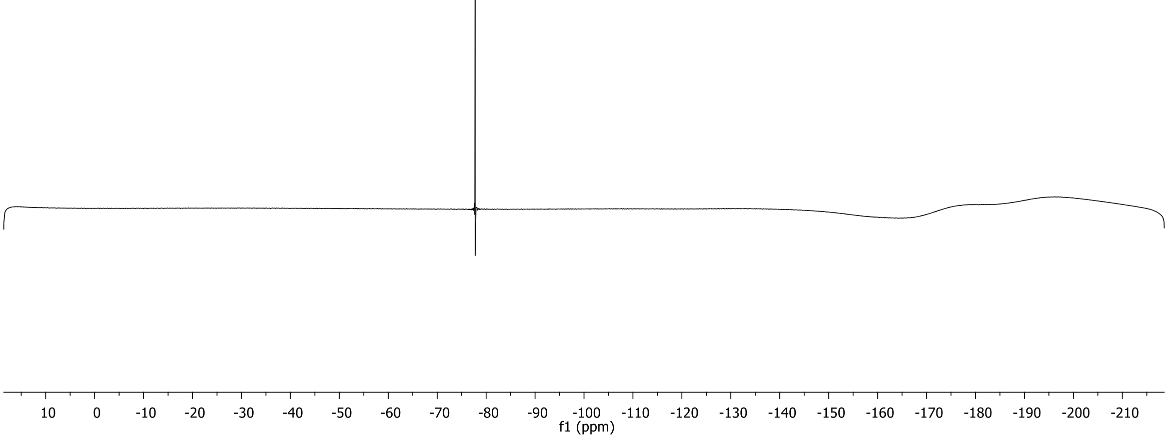

7-(4-(Diethylamino)phenyl)-1-phenethyl-3-phenyl-2-(pyrrolidin-1-yl)-1H-imidazo[1,2-a]pyridin-4-ium trifluoromethanesulfonate (7a)

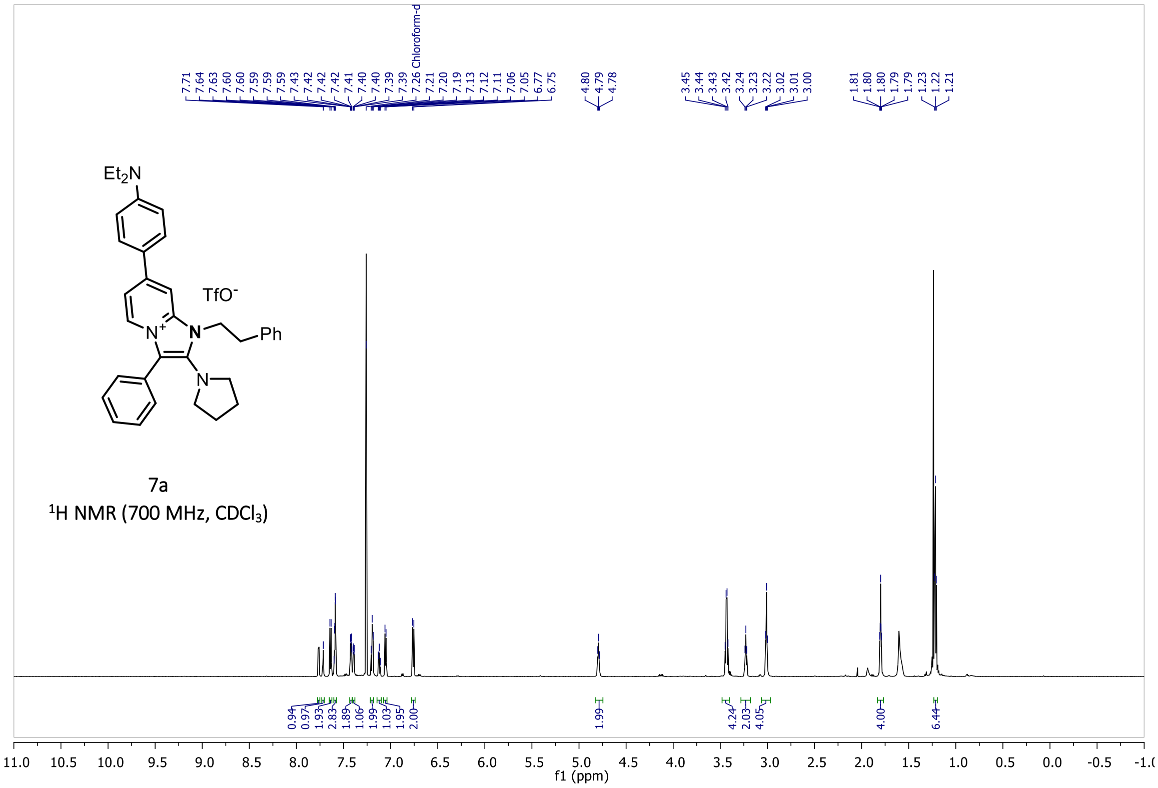

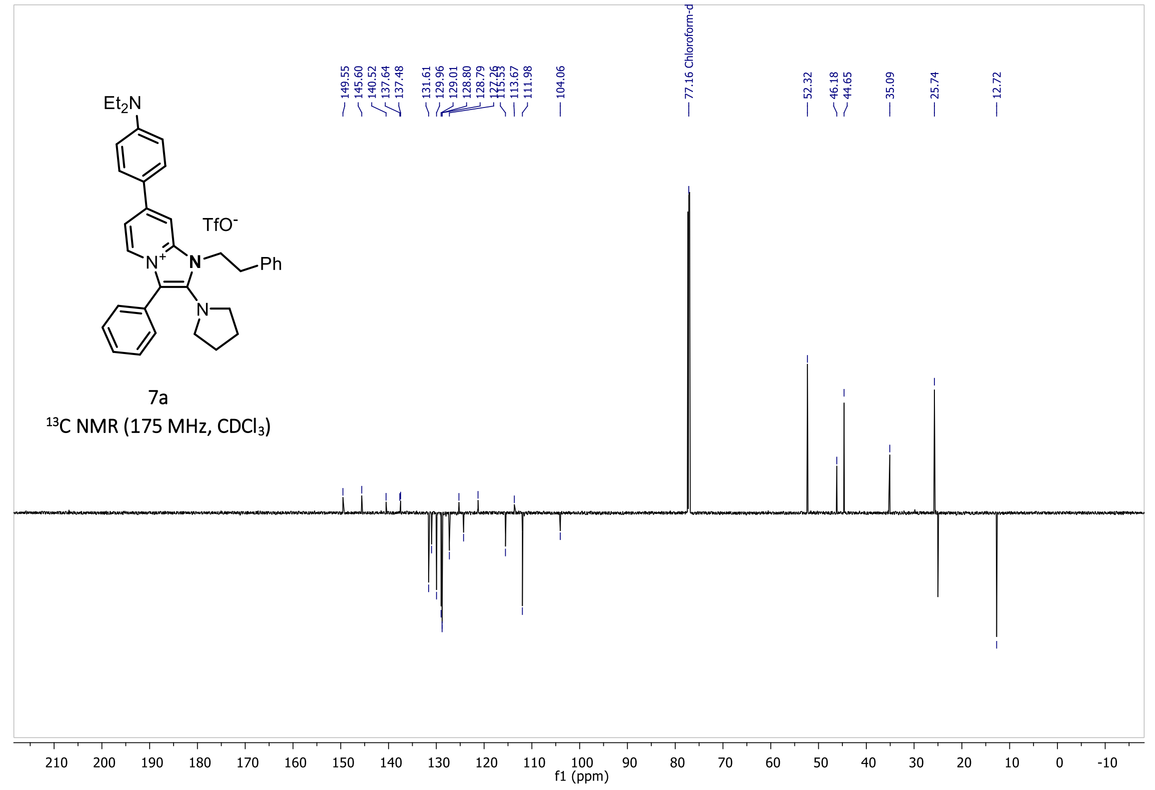

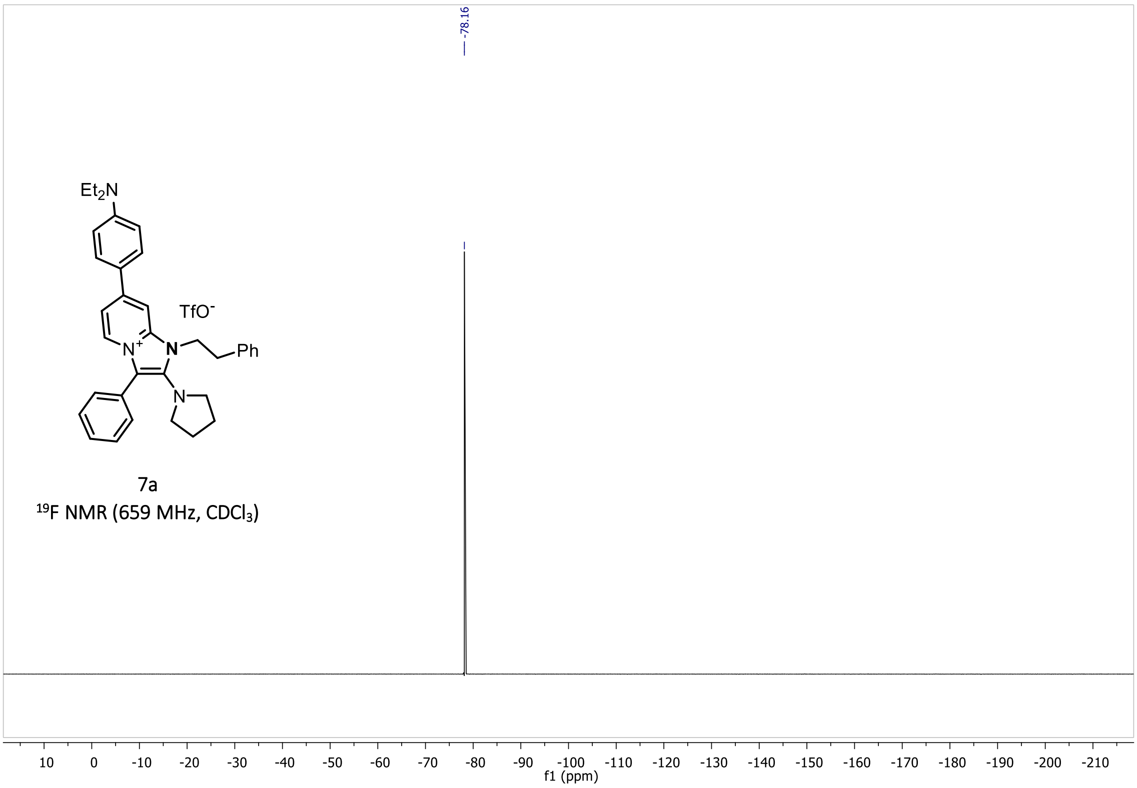

1-Phenethyl-3-phenyl-2-(pyrrolidin-1-yl)-7-(2,3,6,7-tetrahydro-1H,5H-pyrido[3,2,1-ij]quinolin-9-yl)-1H-imidazo[1,2-a]pyridin-4-ium trifluoromethanesulfonate (7b)

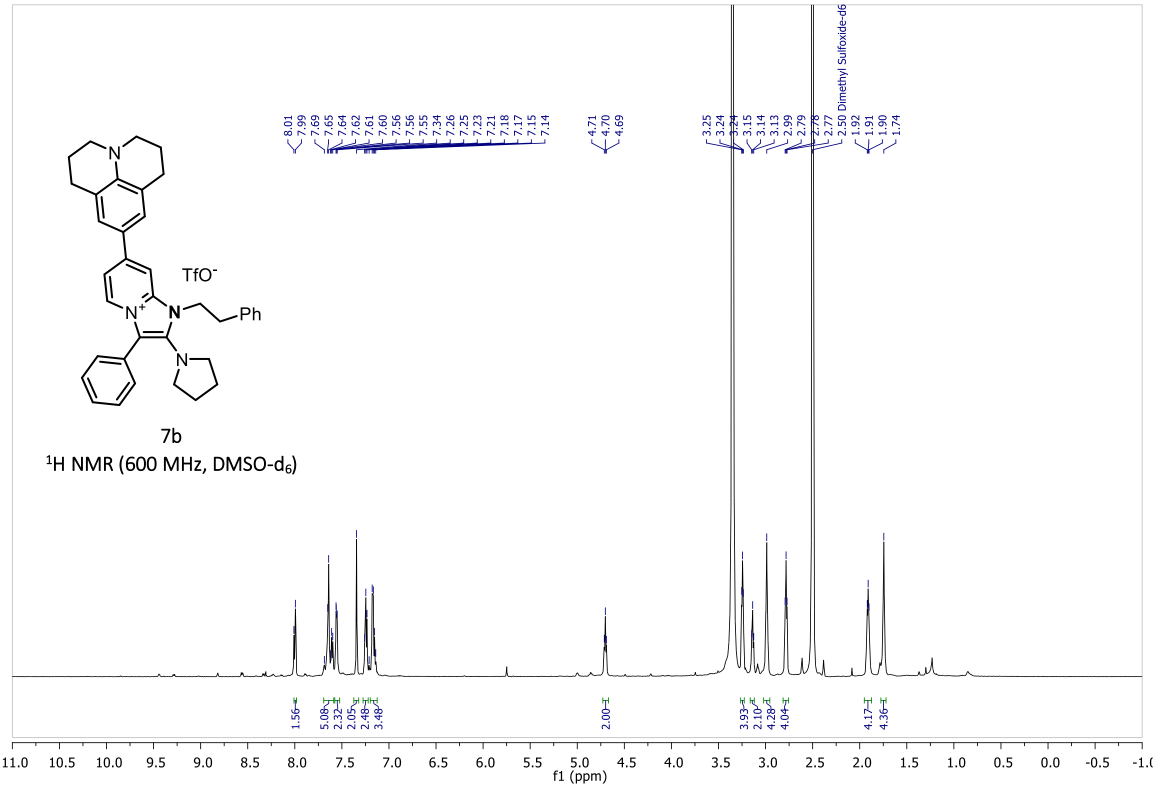

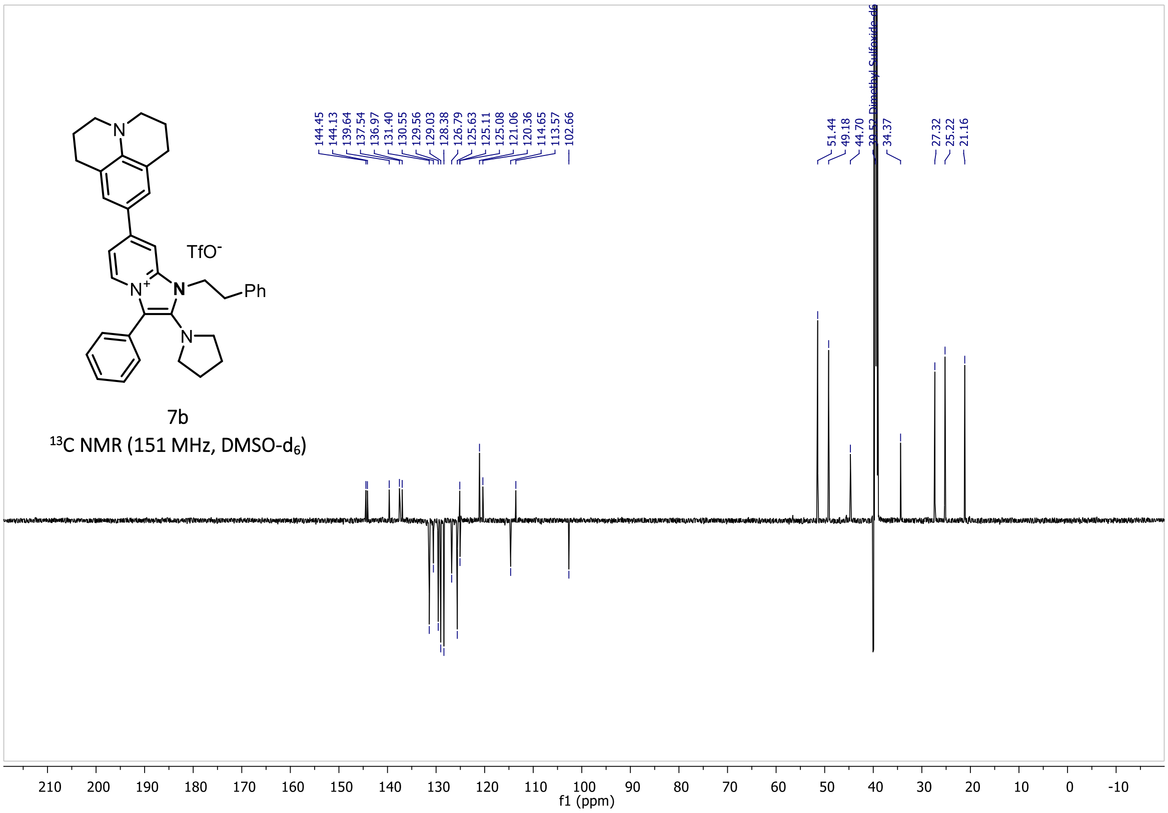
TfO-
Ph
N
N+
7b
13C NMR (151 MHz, DMSO-d6)
144.45
144.13
139.64
137.54
136.97
131.40
130.55
129.56
129.03
128.38
126.79
125.63
125.11
125.08
121.06
120.36
114.65
113.57
102.66
51.44
49.18
44.70
39.52 Dimethyl Sulfoxide-d6
34.37
27.32
25.22
21.16
f1 (ppm)

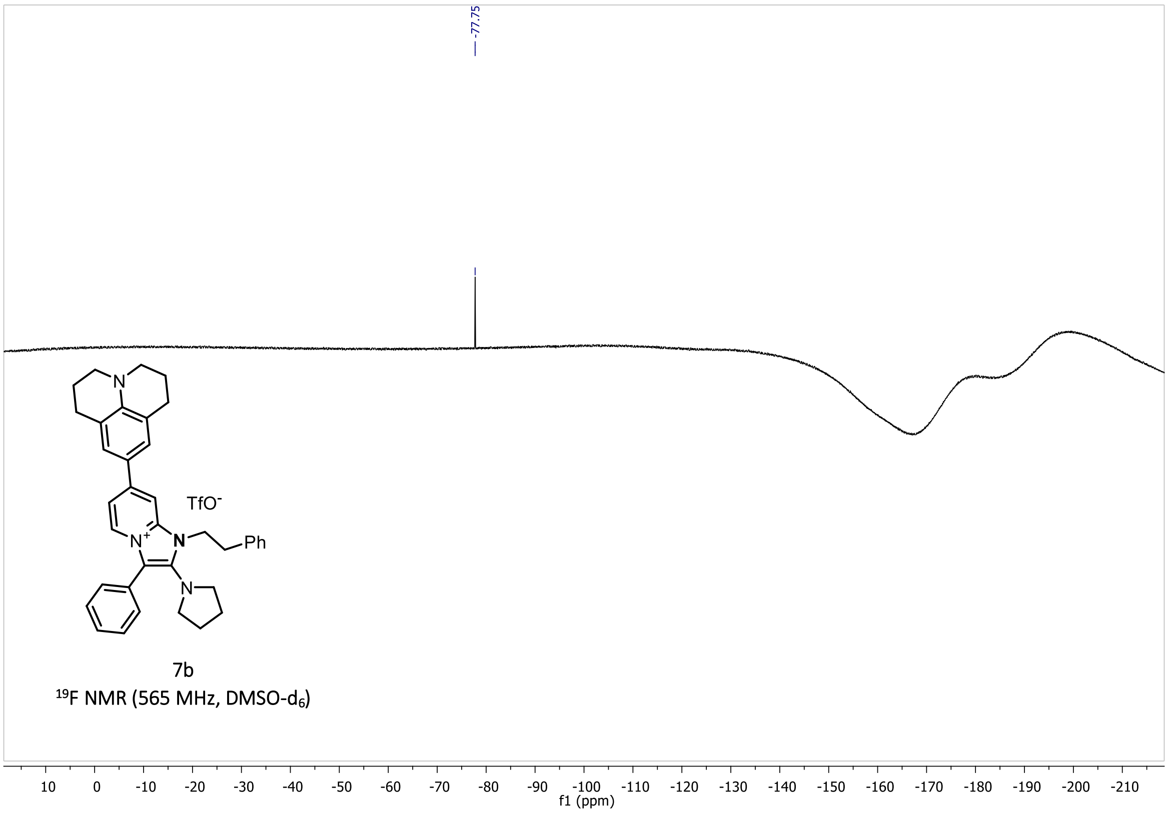
-77.75
TfO-
Ph
N
N+
7b
19F NMR (565 MHz, DMSO-d6)
f1 (ppm)

7-(4'-(Diethylamino)-[1,1'-biphenyl]-4-yl)-1-phenethyl-3-phenyl-2-(pyrrolidin-1-yl)-1H-imidazo[1,2-a]pyridin-4-ium trifluoromethanesulfonate (7c)

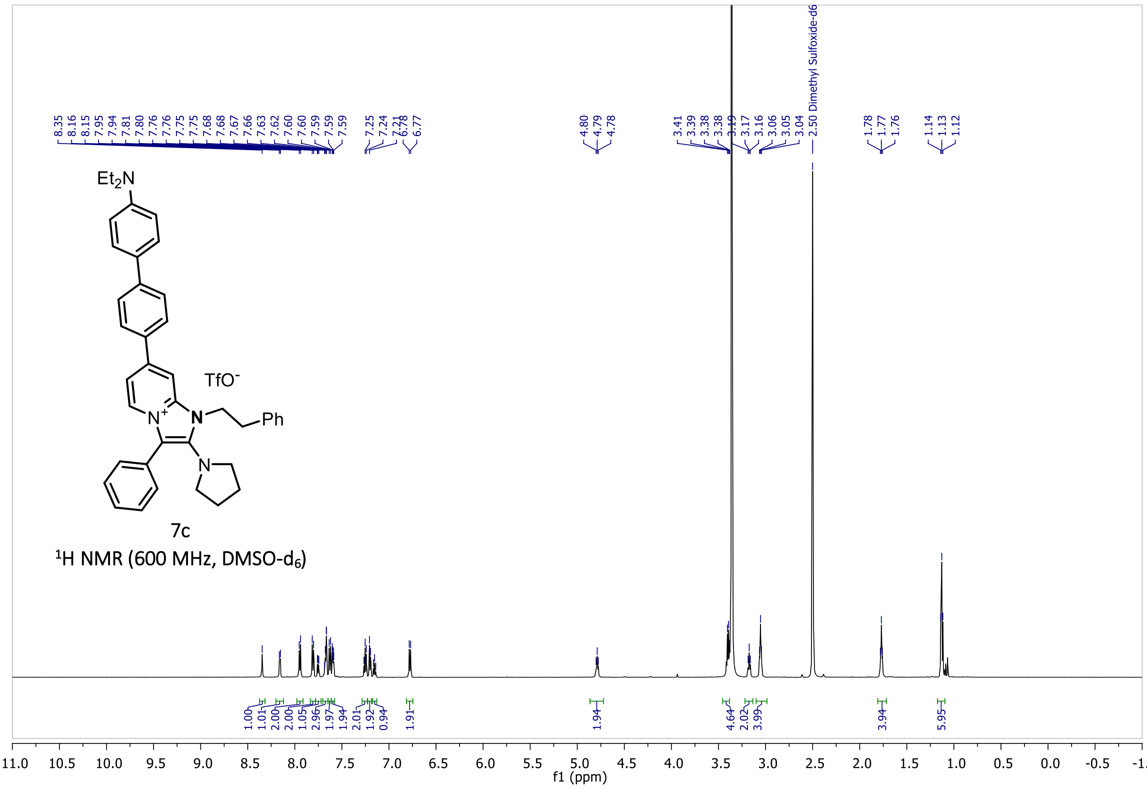

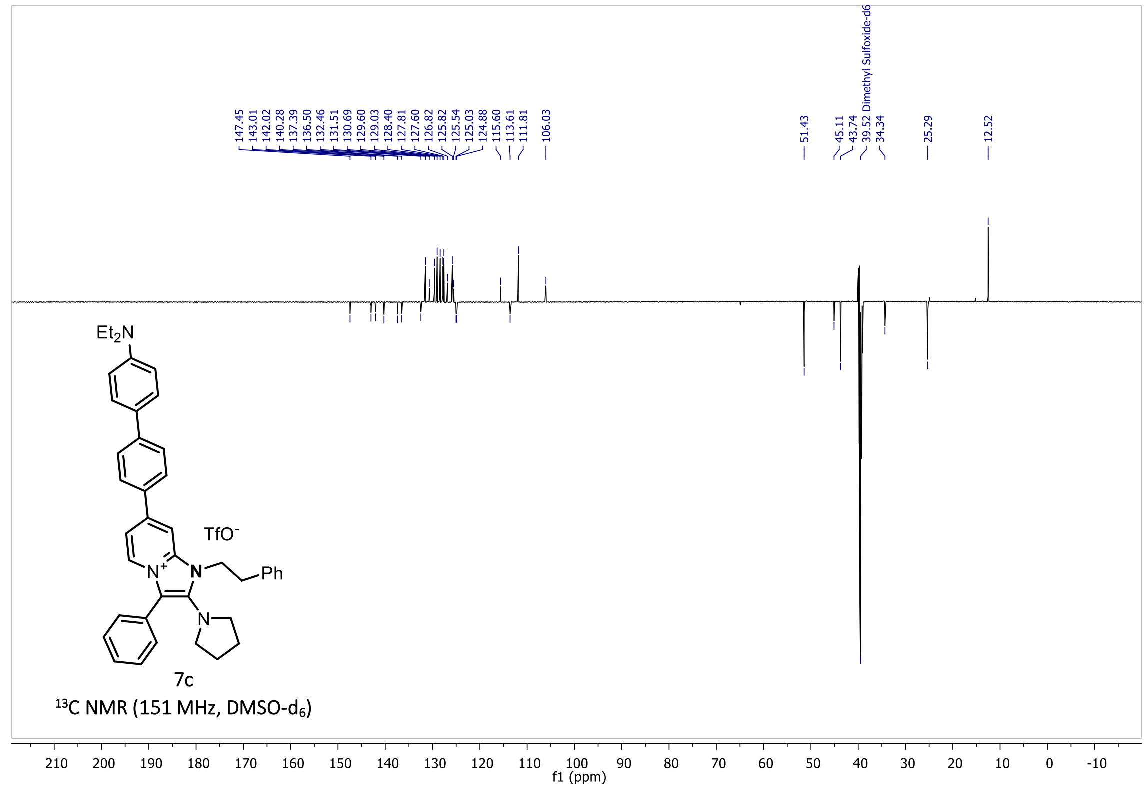

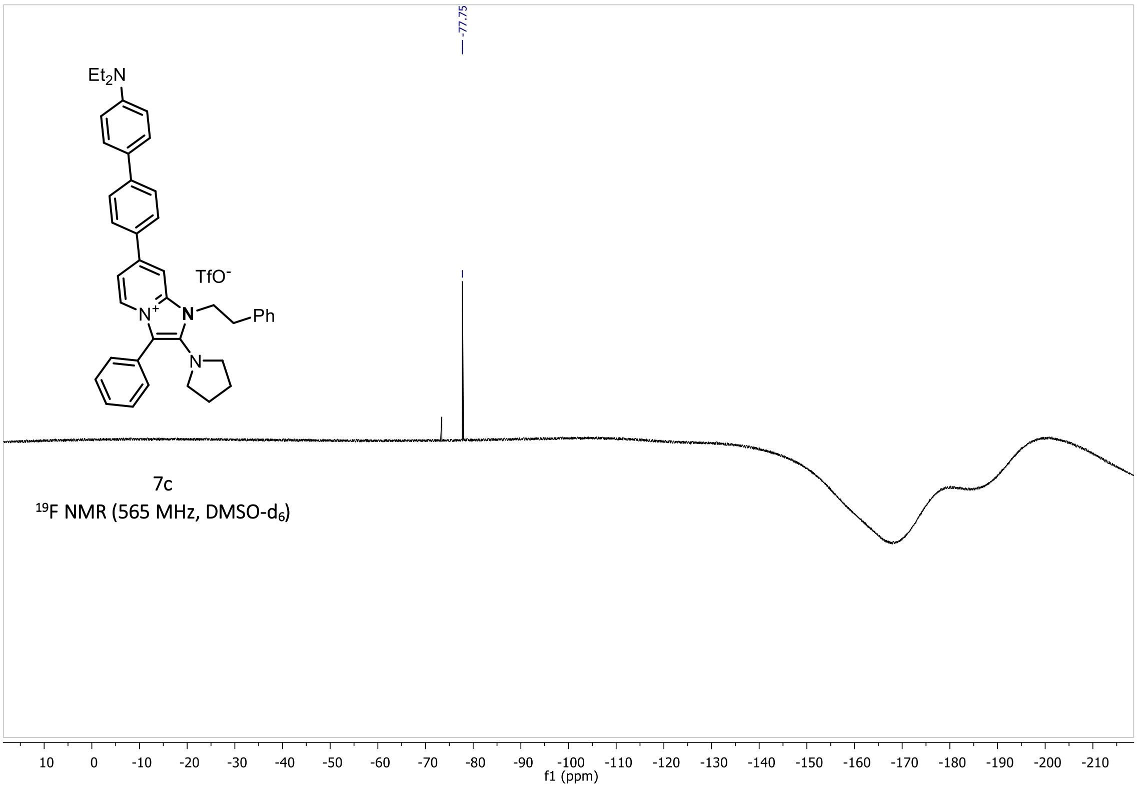

7-(4-(Methoxycarbonyl)phenyl)-1-phenethyl-3-phenyl-2-(pyrrolidin-1-yl)-1H-imidazo[1,2-a]pyridin-4-ium trifluoromethanesulfonate and trifluoroacetate (7d)

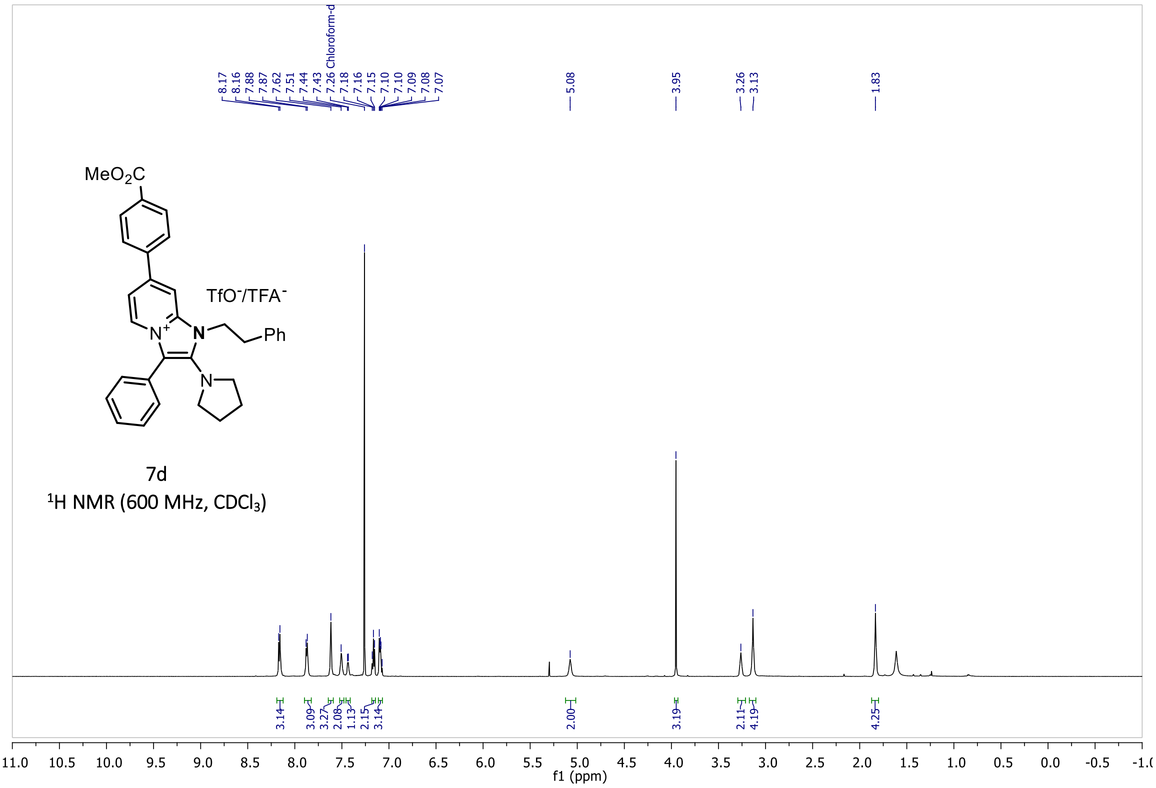

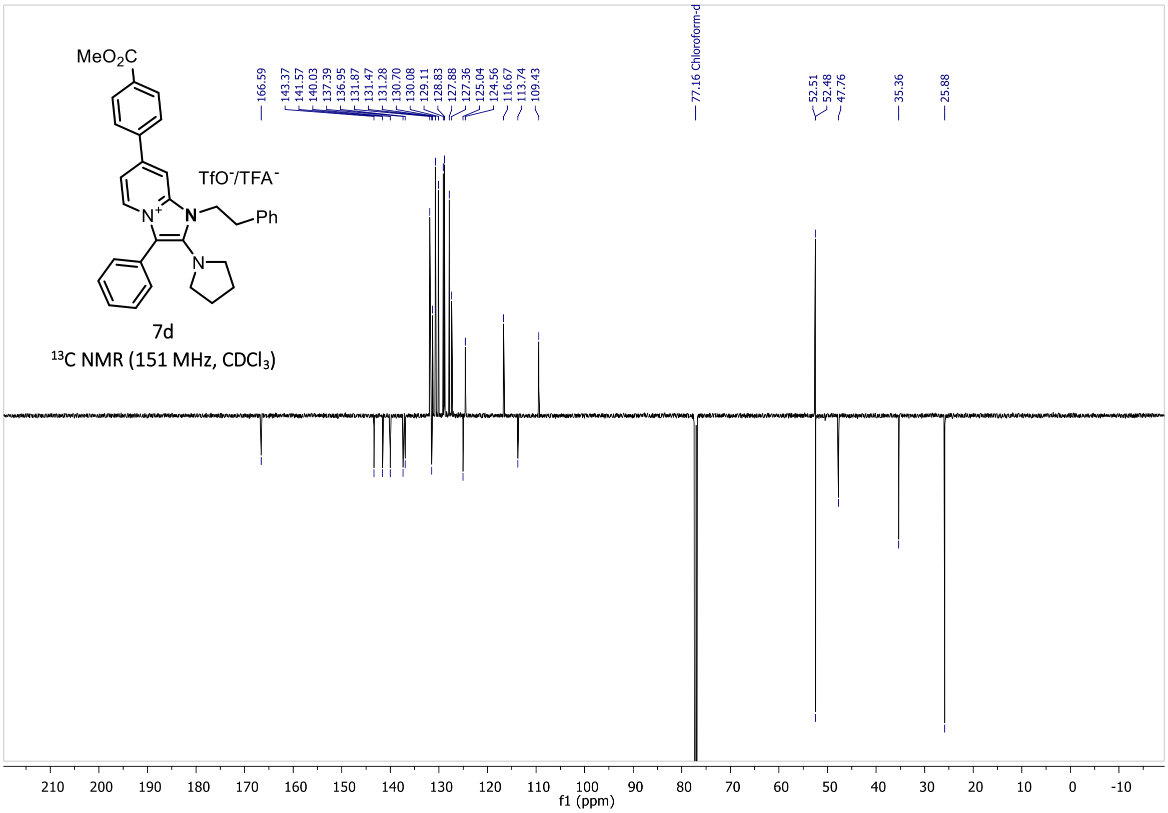
MeO2C
TfO-/TFA-
N+
N
Ph
N
7d
13C NMR (151 MHz, CDCl3)
166.59
143.37
141.57
140.03
137.39
136.95
131.87
131.47
131.28
130.70
130.08
129.11
128.83
127.88
127.36
125.04
124.56
116.67
113.74
109.43
77.16 Chloroform-d
52.51
52.48
47.76
35.36
25.88
210 200 190 180 170 160 150 140 130 120 110 100 90 80 70 60 50 40 30 20 10 0 -10
f1 (ppm)

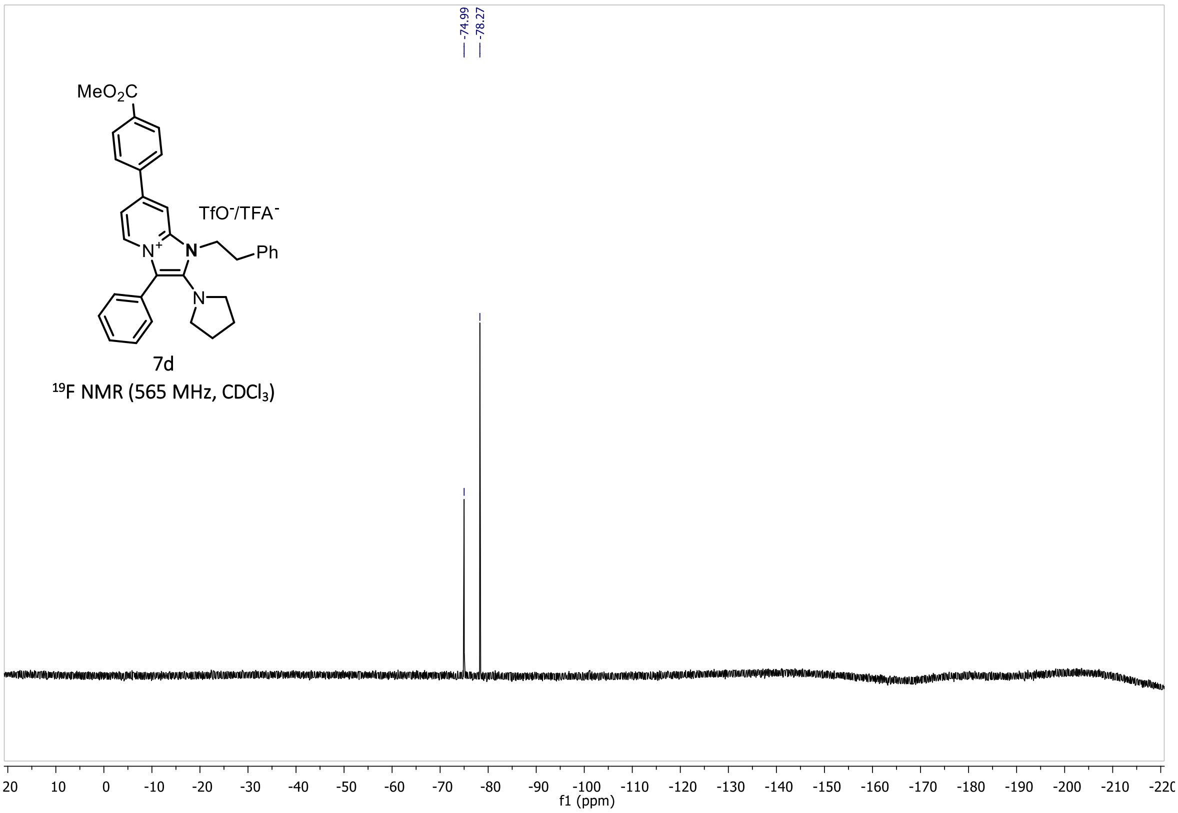
MeO2C
TfO-/TFA-
N+
N
Ph
N
7d
19F NMR (565 MHz, CDCl3)
-74.99
-78.27
20 10 0 -10 -20 -30 -40 -50 -60 -70 -80 -90 -100 -110 -120 -130 -140 -150 -160 -170 -180 -190 -200 -210 -220
f1 (ppm)

1-(3-Azidopropyl)-7-(4-(diethylamino)phenyl)-3-phenyl-2-(pyrrolidin-1-yl)-1H-imidazo[1,2-a]pyridin-4-ium trifluoromethanesulfonate (7e)

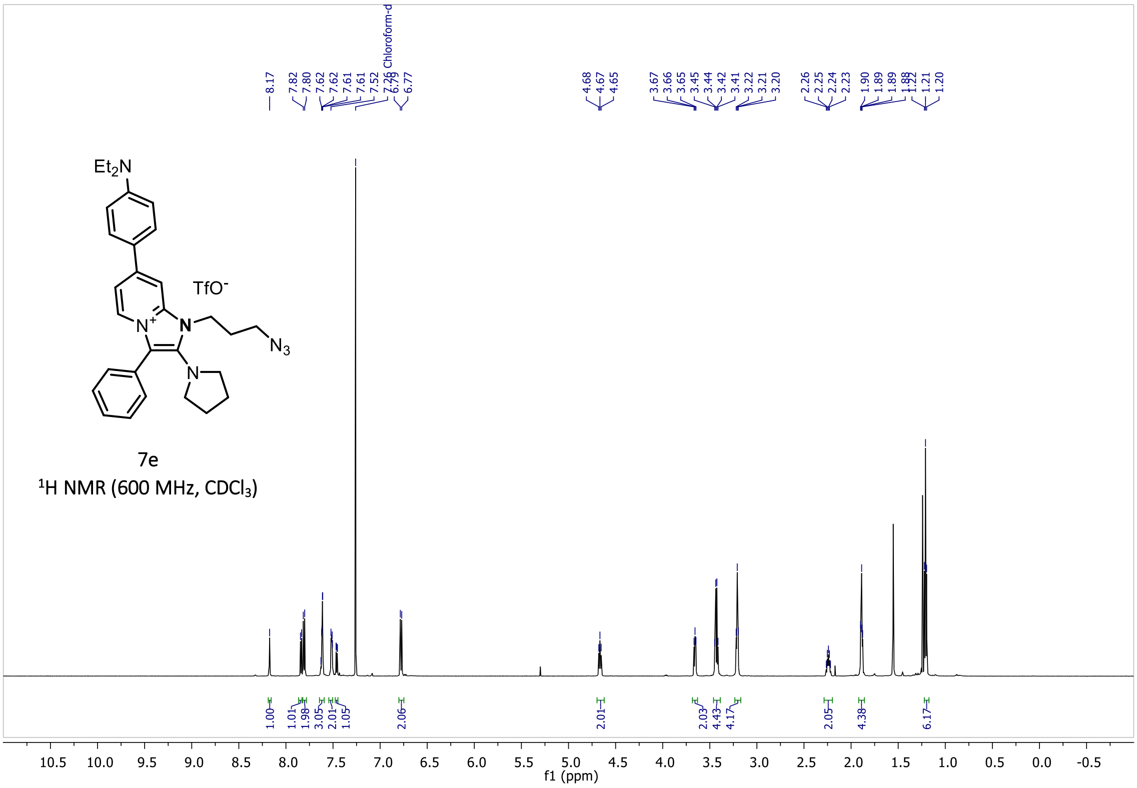

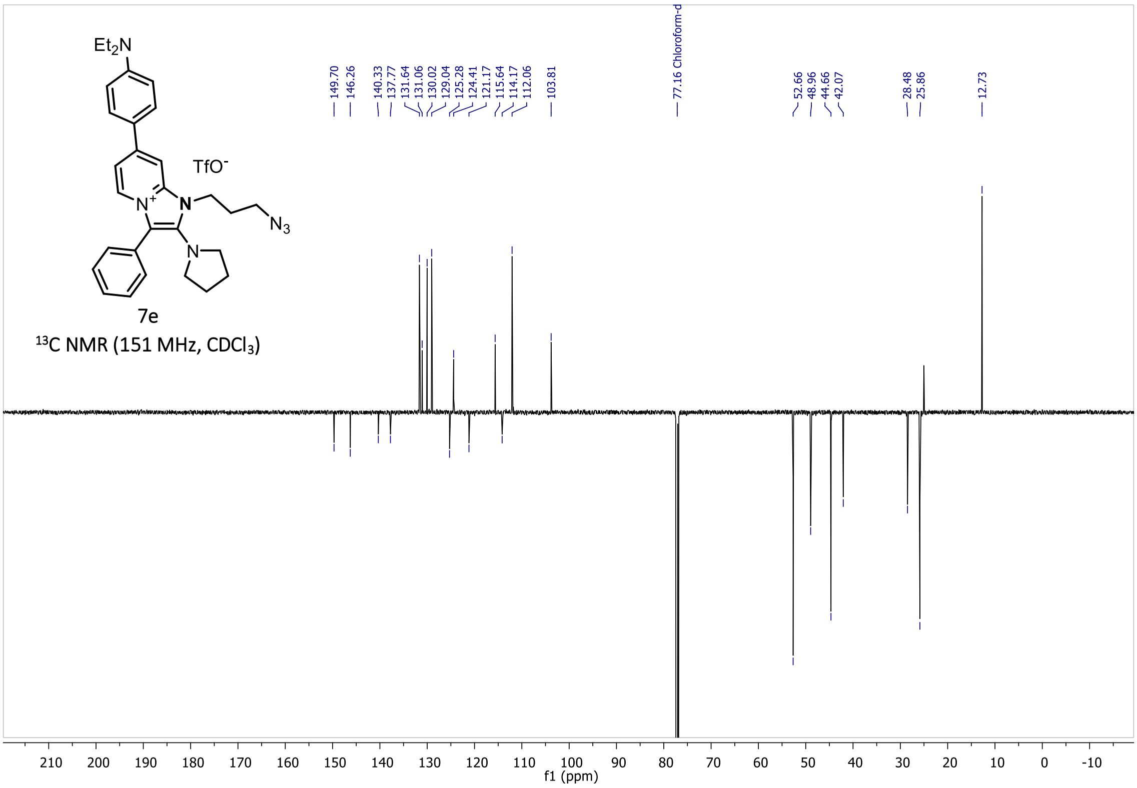

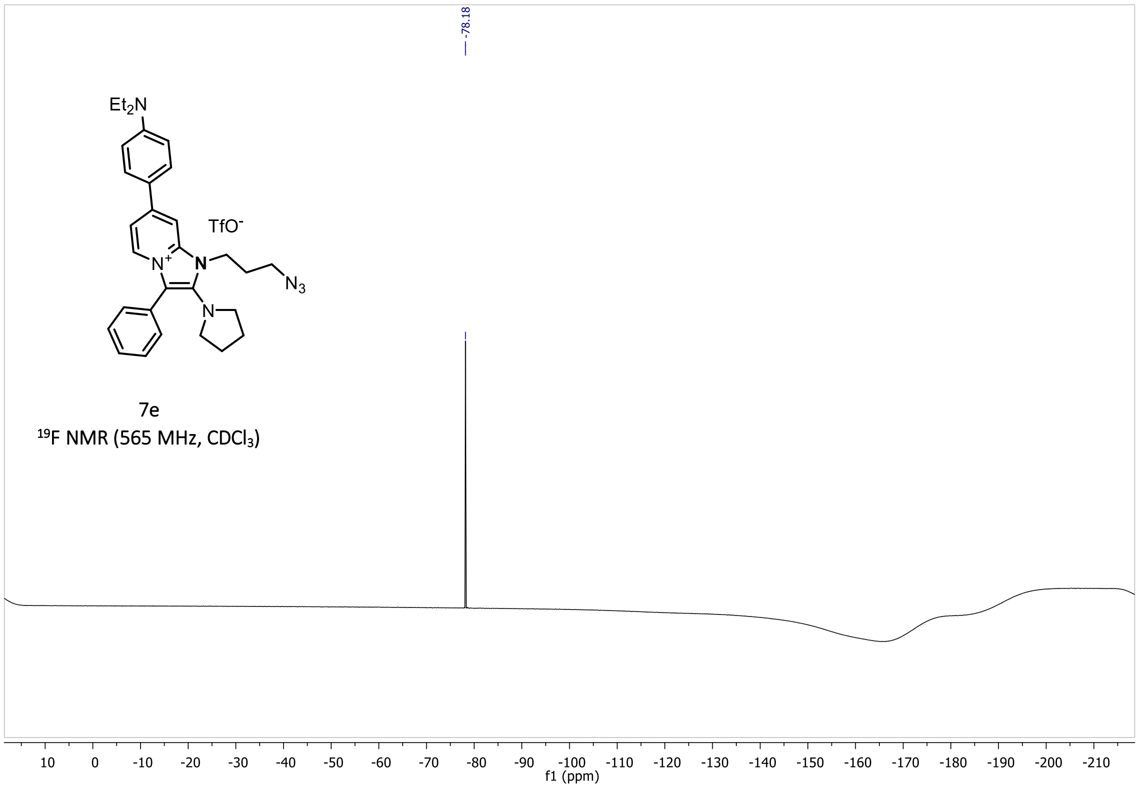

7-(4-(Diethylamino)phenyl)-1-(4-methoxy-4-oxobutyl)-3-phenyl-2-(pyrrolidin-1-yl)-1H-imidazo[1,2-a]pyridin-4-ium trifluoromethanesulfonate (7f)

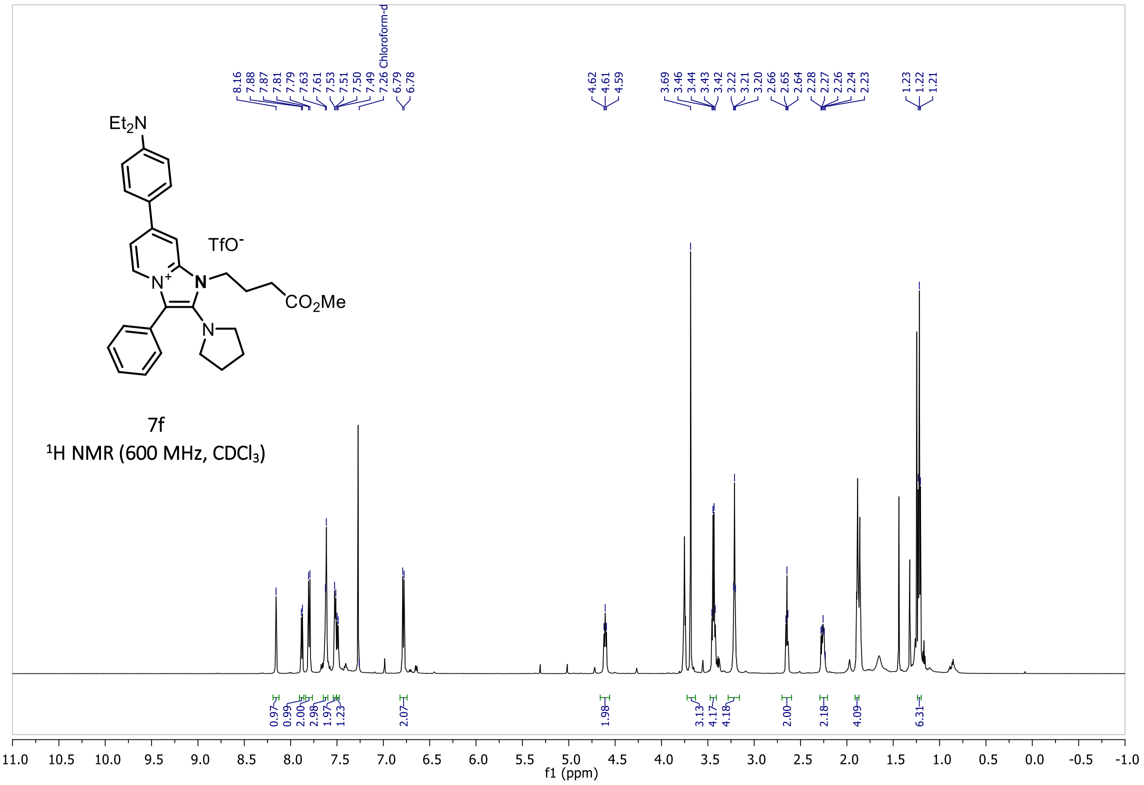

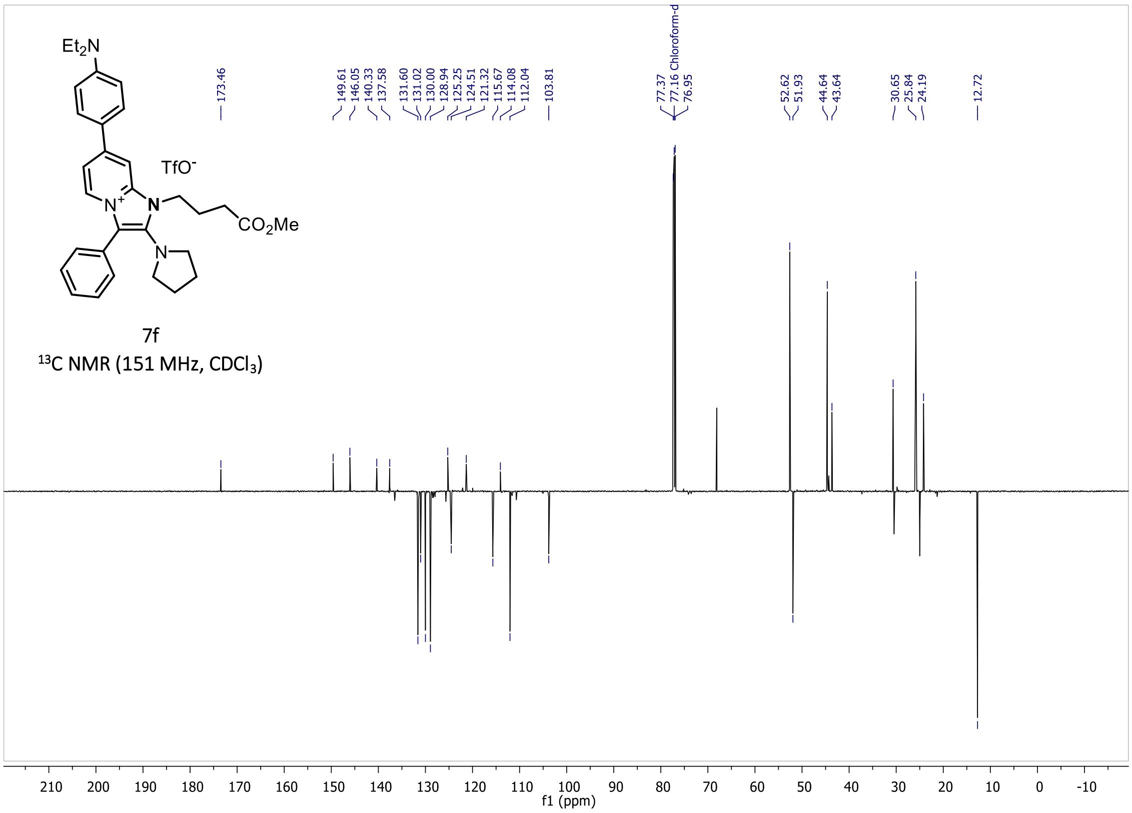
Et2N
TfO-
N+
N
CO2Me
N
7f
13C NMR (151 MHz, CDCl3)
173.46
149.61
146.05
140.33
137.58
131.60
131.02
130.00
128.94
125.25
124.51
121.32
115.67
114.08
112.04
103.81
77.37
77.16 Chloroform-d
76.95
52.62
51.93
44.64
43.64
30.65
25.84
24.19
12.72
210 200 190 180 170 160 150 140 130 120 110 100 90 80 70 60 50 40 30 20 10 0 -10
f1 (ppm)

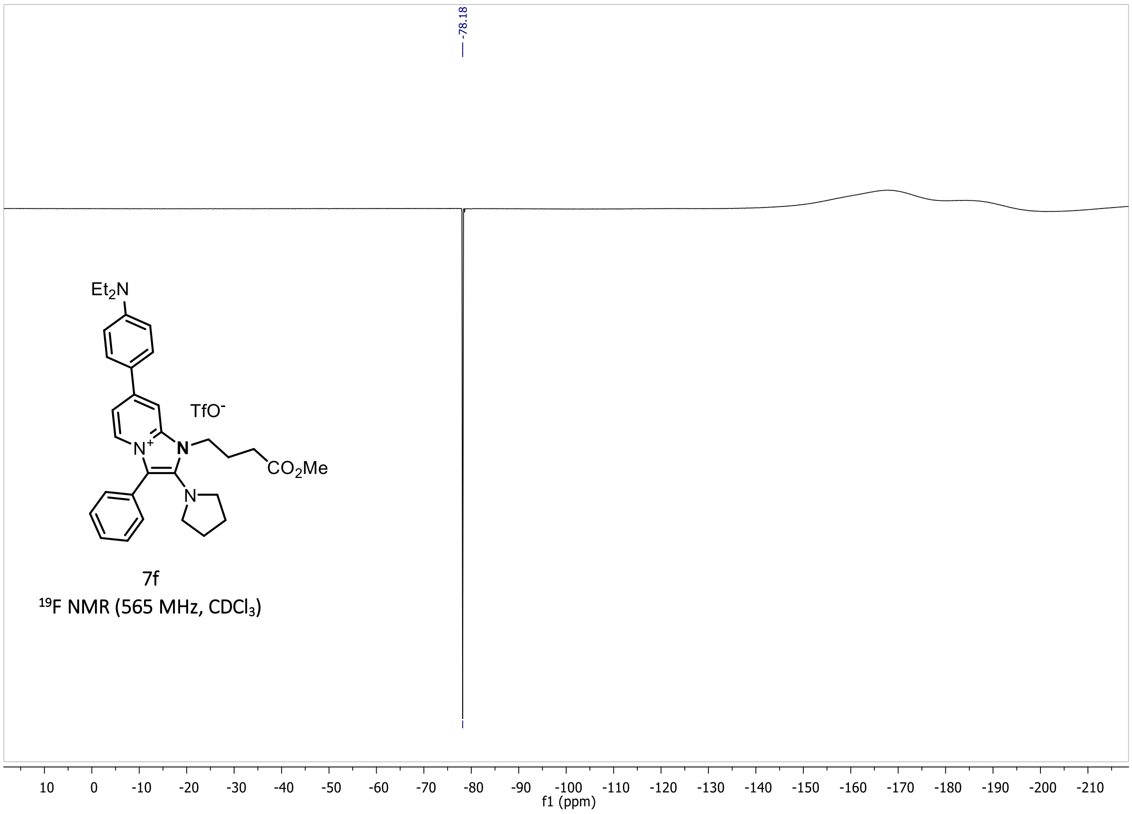
-78.18
Et2N
TfO-
N+
N
CO2Me
N
7f
19F NMR (565 MHz, CDCl3)
10 0 -10 -20 -30 -40 -50 -60 -70 -80 -90 -100 -110 -120 -130 -140 -150 -160 -170 -180 -190 -200 -210
f1 (ppm)

(*E*)-7-(4-(Diethylamino)styryl)-1-phenethyl-3-phenyl-2-(pyrrolidin-1-yl)-1H-imidazo[1,2-a]pyridin-4-ium trifluoromethanesulfonate (7g)

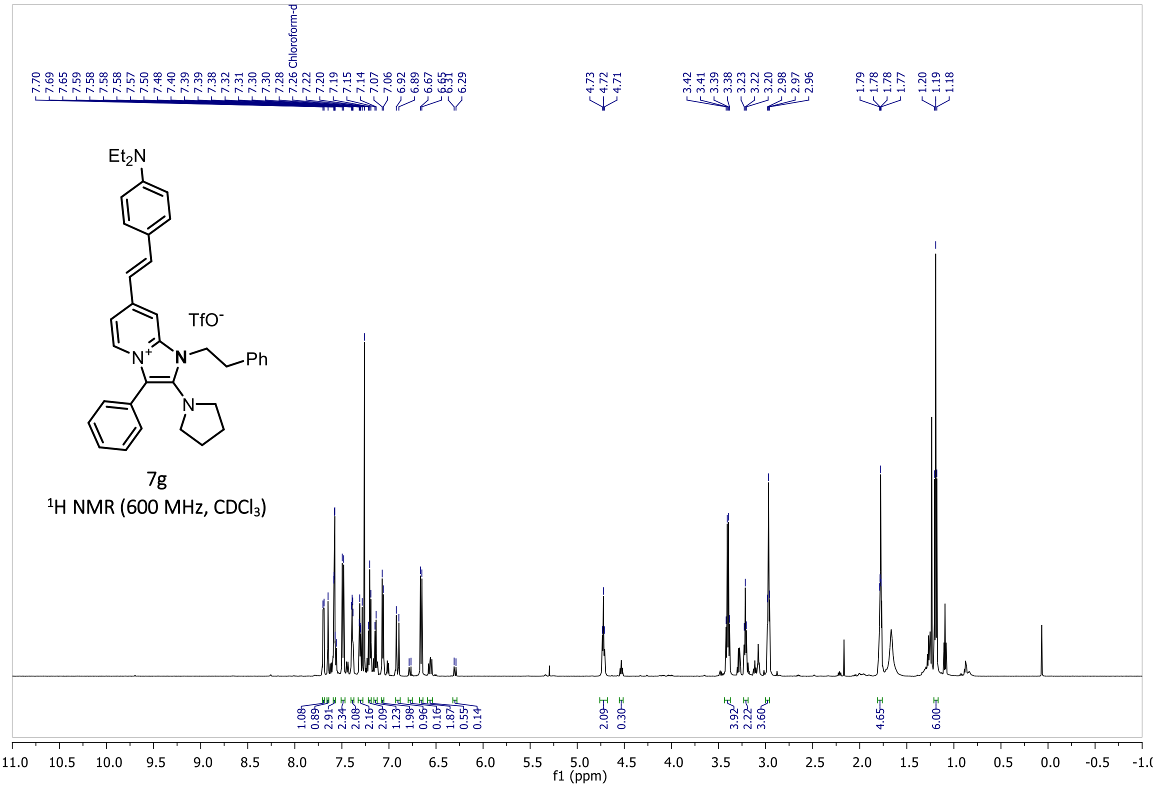

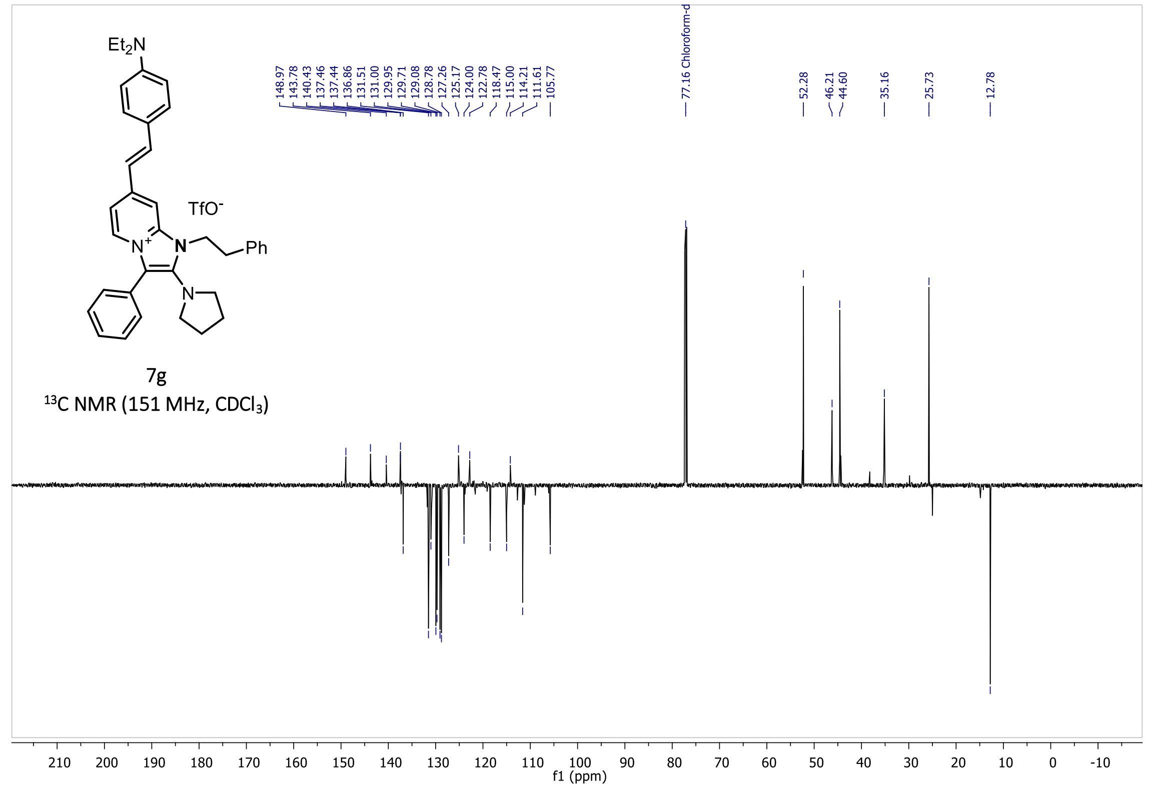

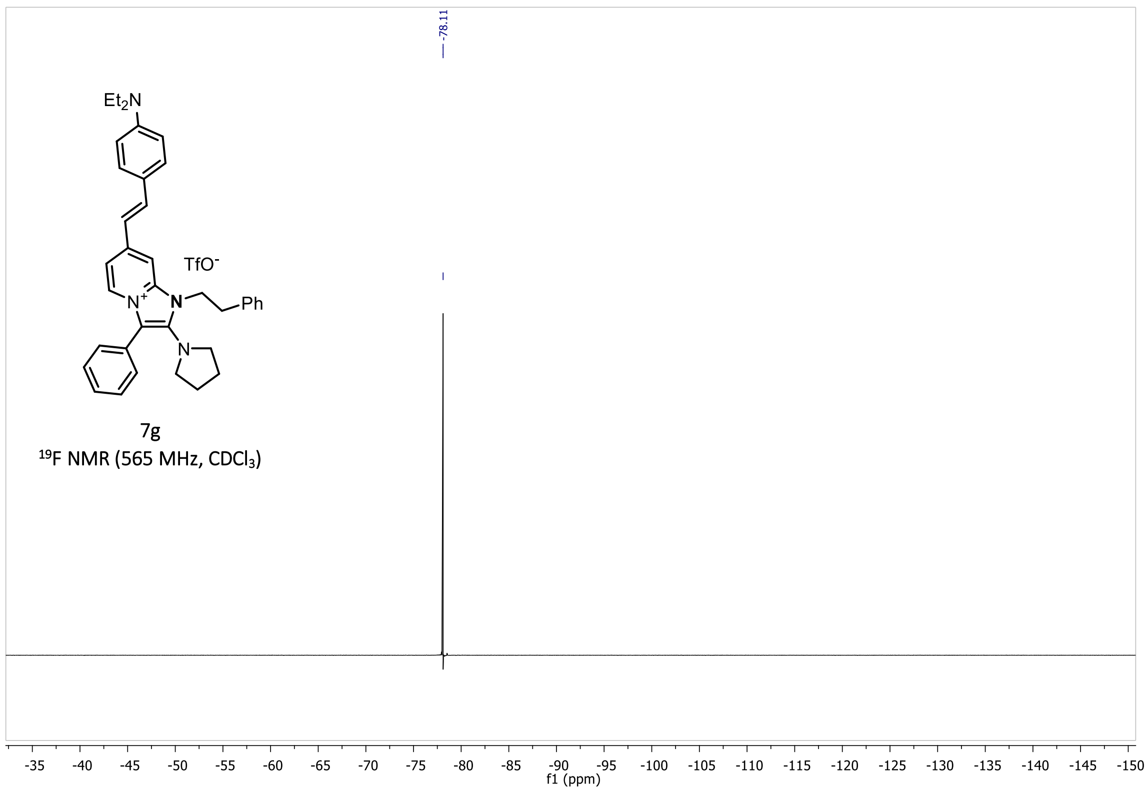

(*E*)-1-Phenethyl-3-phenyl-2-(pyrrolidin-1-yl)-7-(2-(2,3,6,7-tetrahydro-1H,5H-pyrido[3,2,1-ij]quinolin-9-yl)vinyl)-1H-imidazo[1,2-a]pyridin-4-ium trifluoromethanesulfonate (7h)

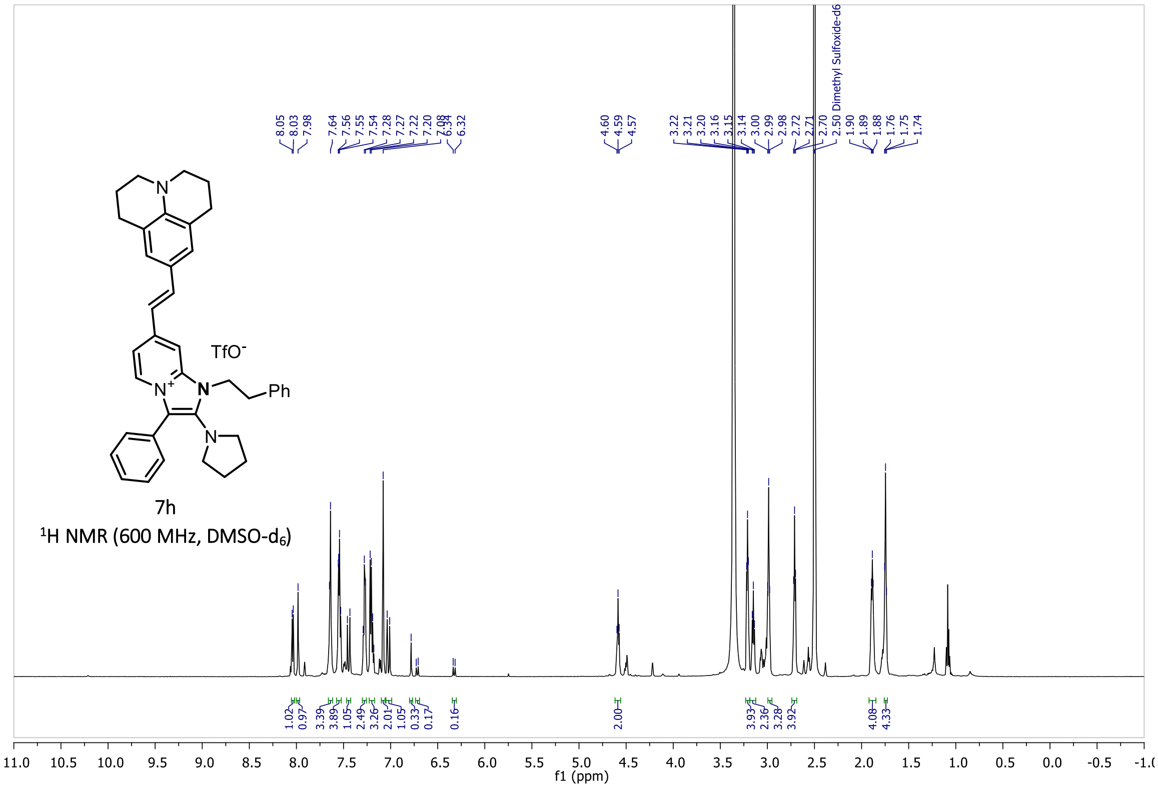

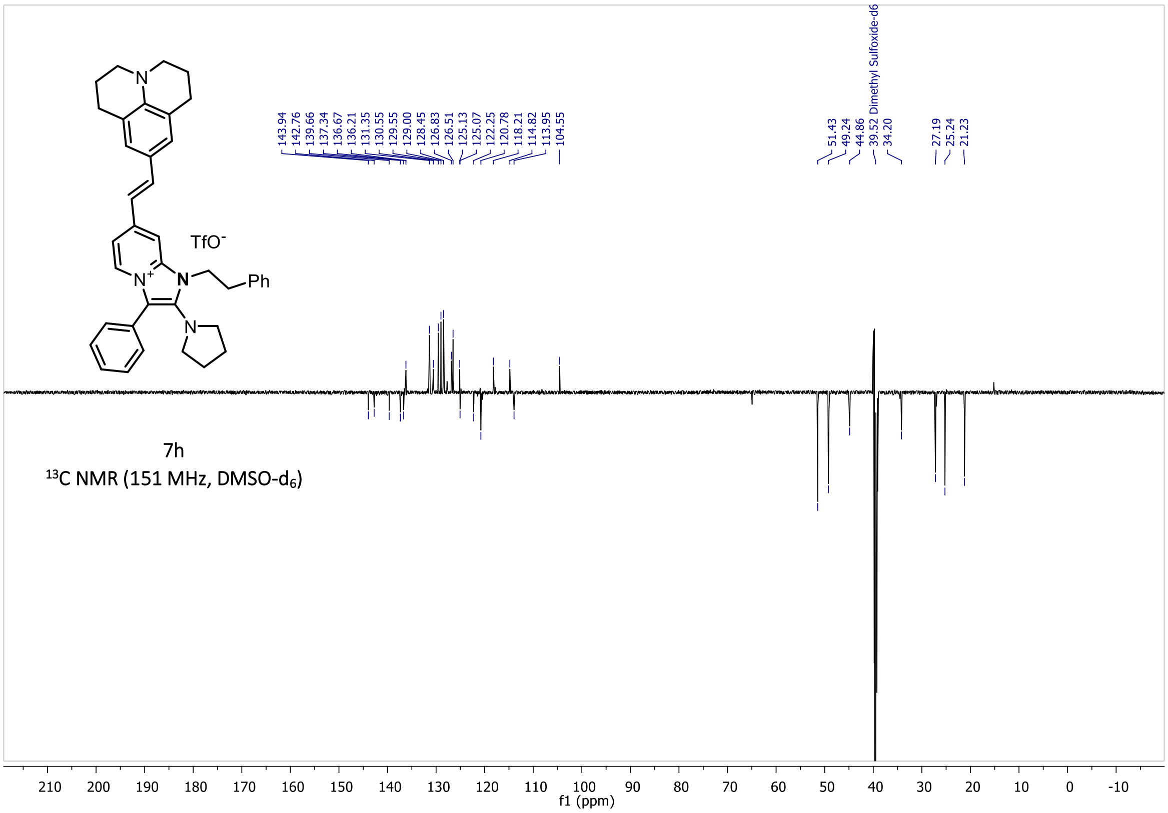

7h

^{13}C NMR (151 MHz, DMSO-d_6)

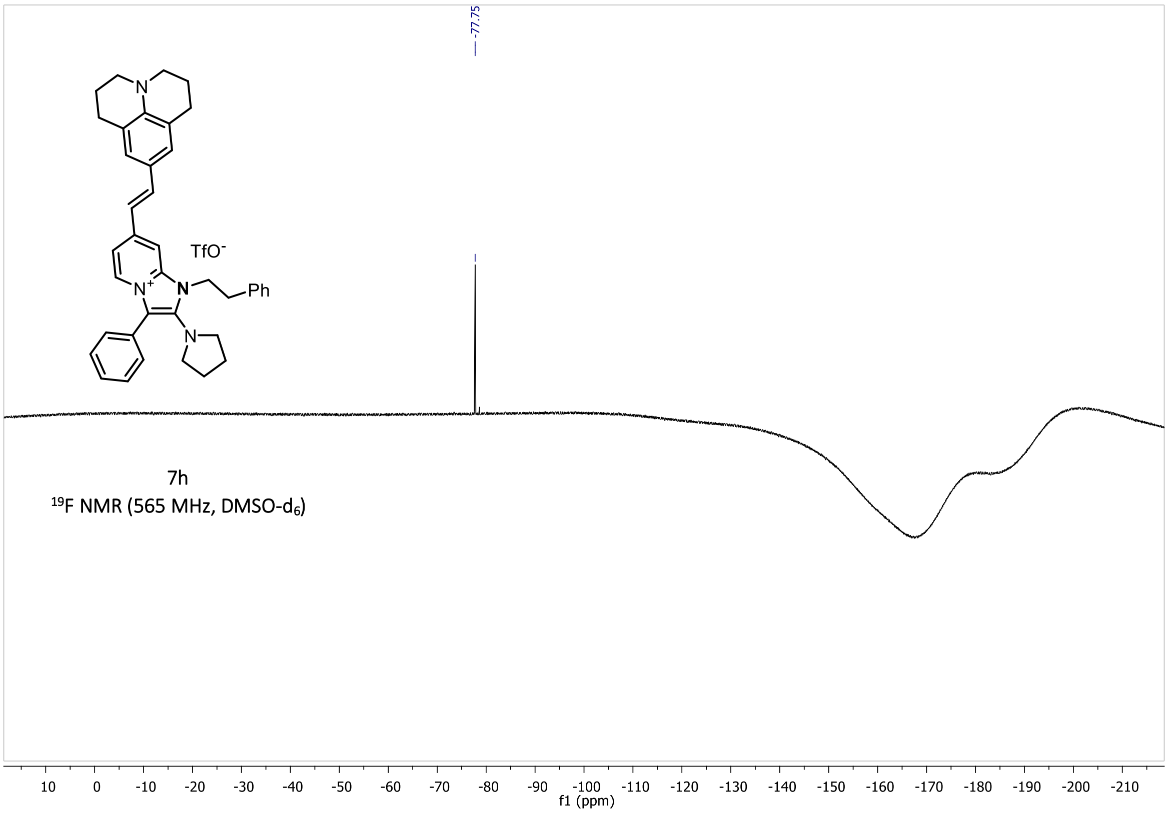

7h

^{19}F NMR (565 MHz, DMSO-d_6)

(*E*)-7-(4-Morpholinostyryl)-1-phenethyl-3-phenyl-2-(pyrrolidin-1-yl)-1H-imidazo[1,2-a]pyridin-4-ium trifluoromethanesulfonate (7i)

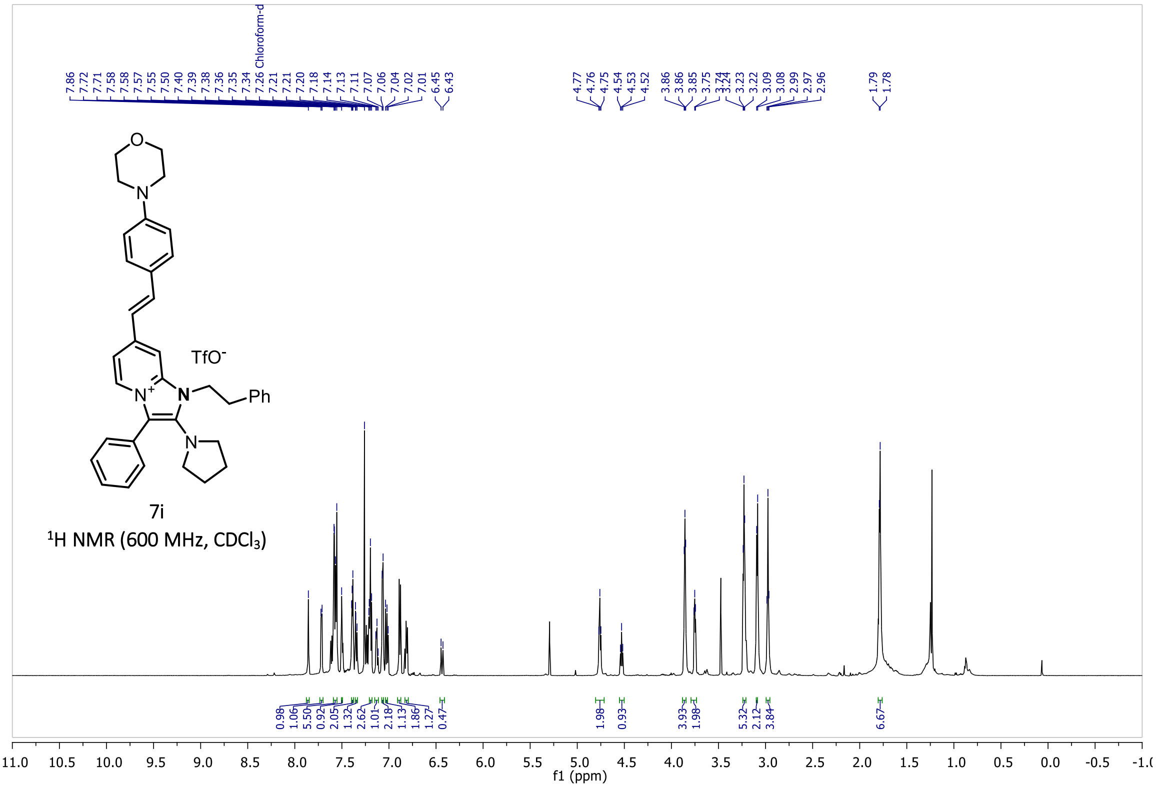

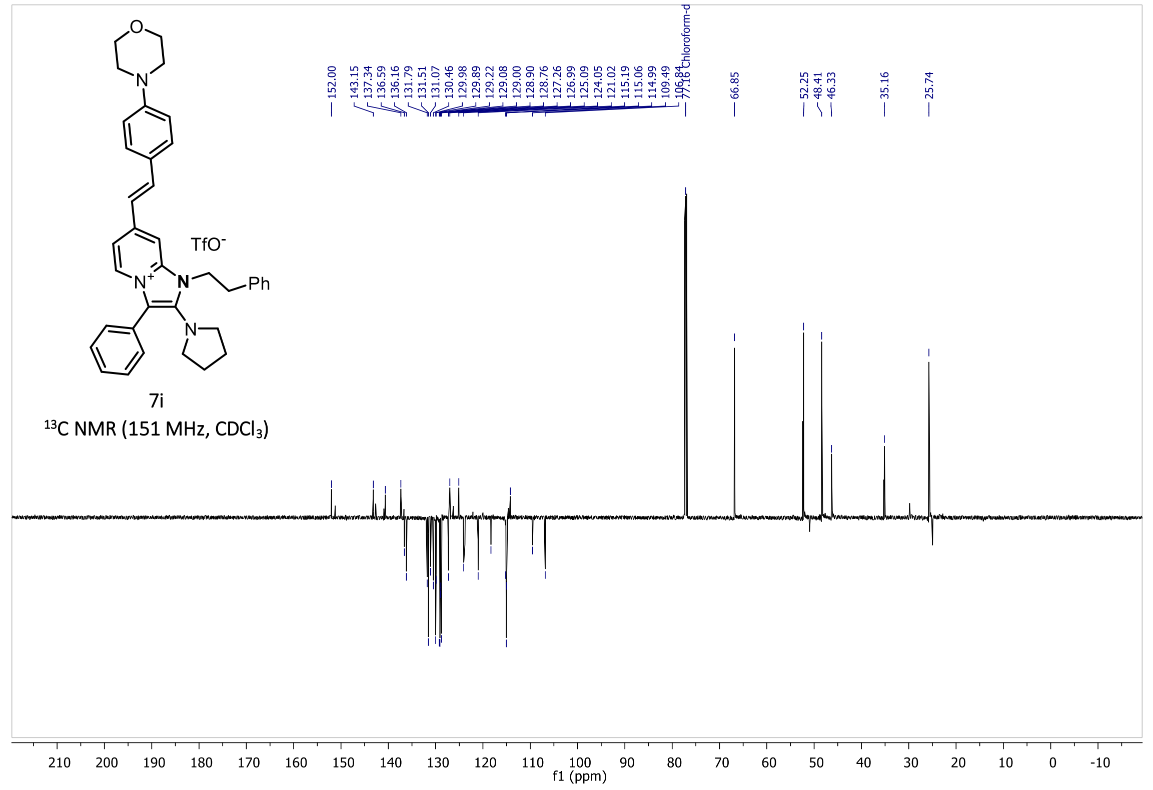

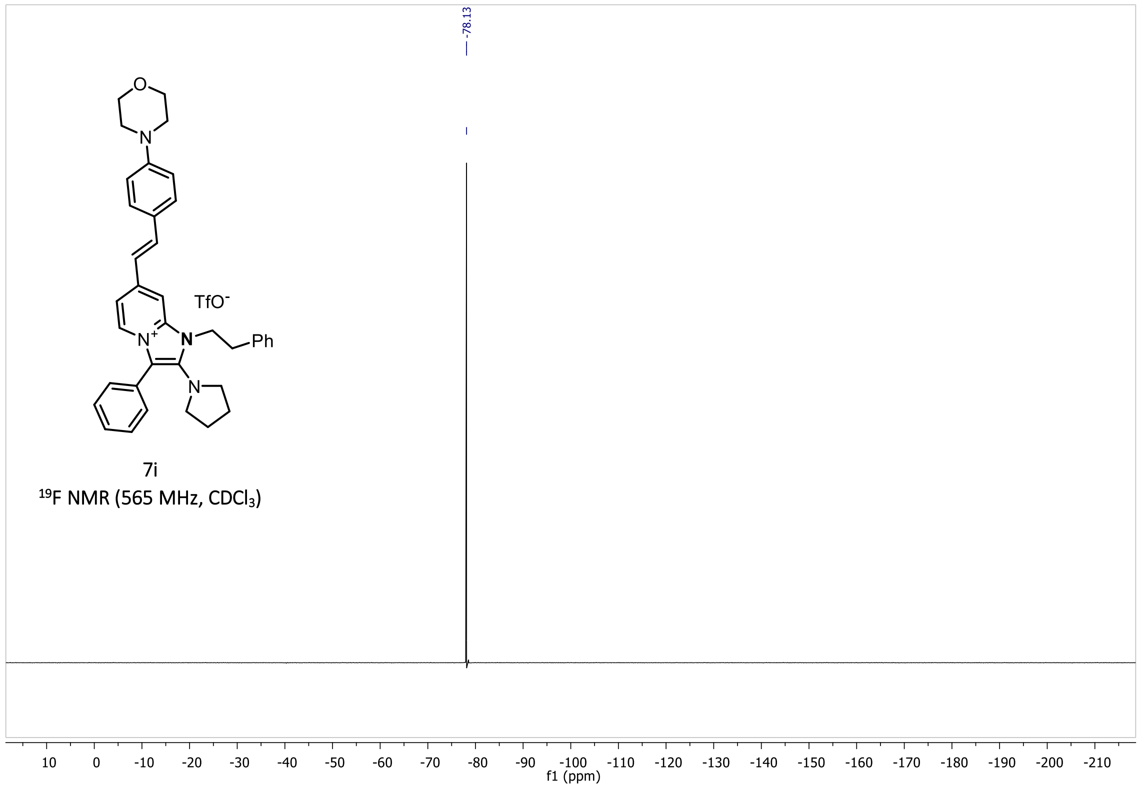

1-Phenethyl-3-phenyl-2-(pyrrolidin-1-yl)-7-((*E*)-4-((*E*)-3,4,5-trimethoxystyryl)styryl)-1H-imidazo[1,2-a]pyridin-4-ium trifluoromethanesulfonate (7j)

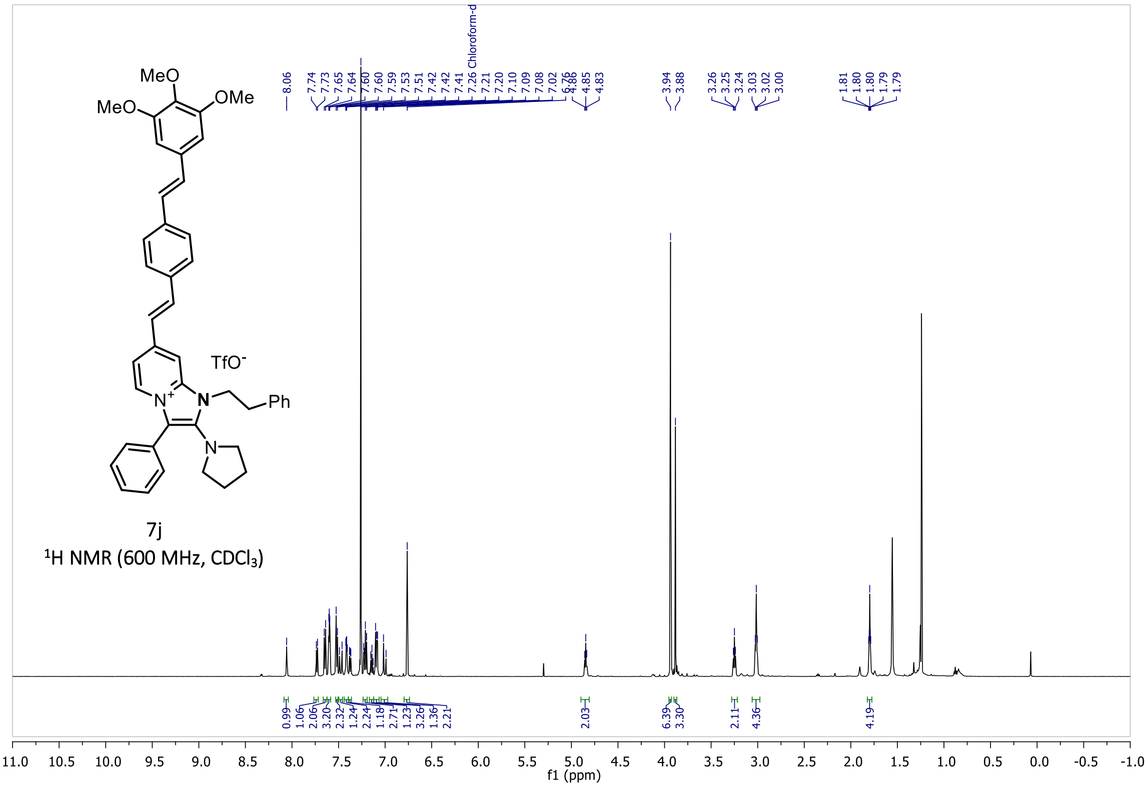

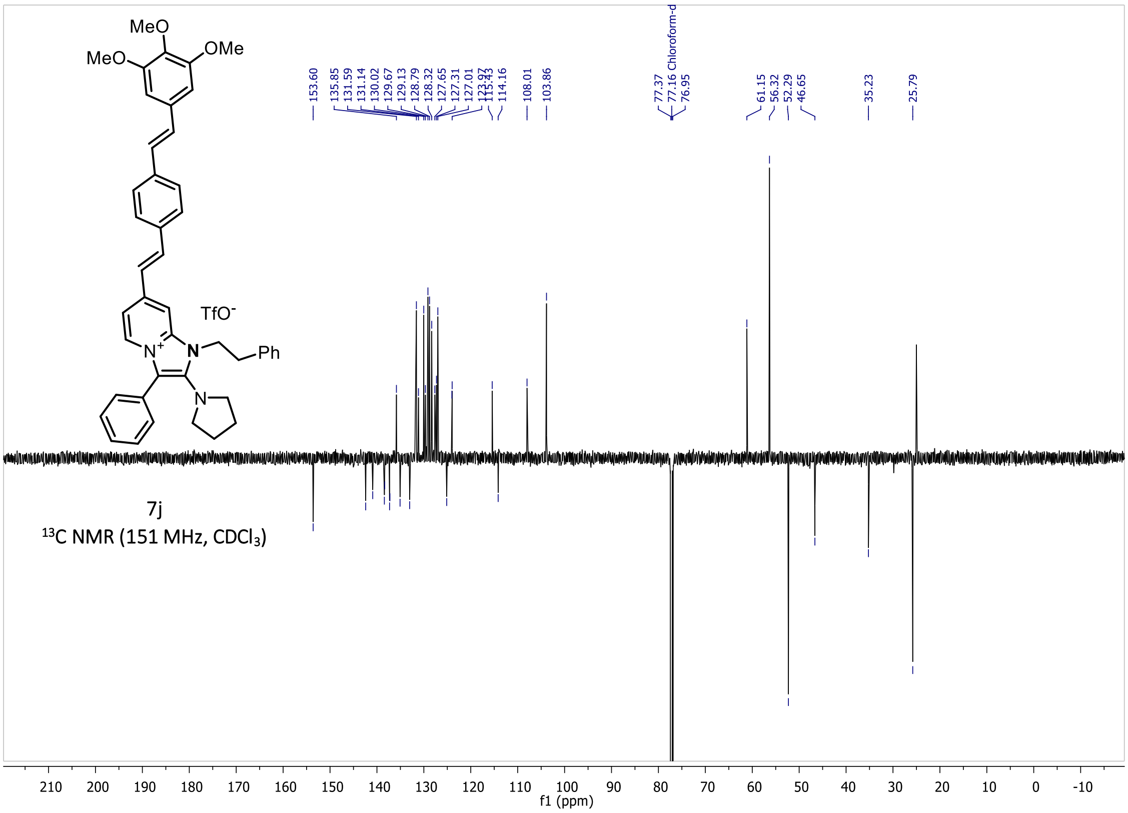

MeO
MeO
OMe
TfO-
N+
N
Ph
N
7j
13C NMR (151 MHz, CDCl3)
153.60
135.85
131.59
131.14
130.02
129.67
129.13
128.79
128.32
127.65
127.31
127.01
123.97
115.43
114.16
108.01
103.86
77.37
77.16 Chloroform-d
76.95
61.15
56.32
52.29
46.65
35.23
25.79
f1 (ppm)

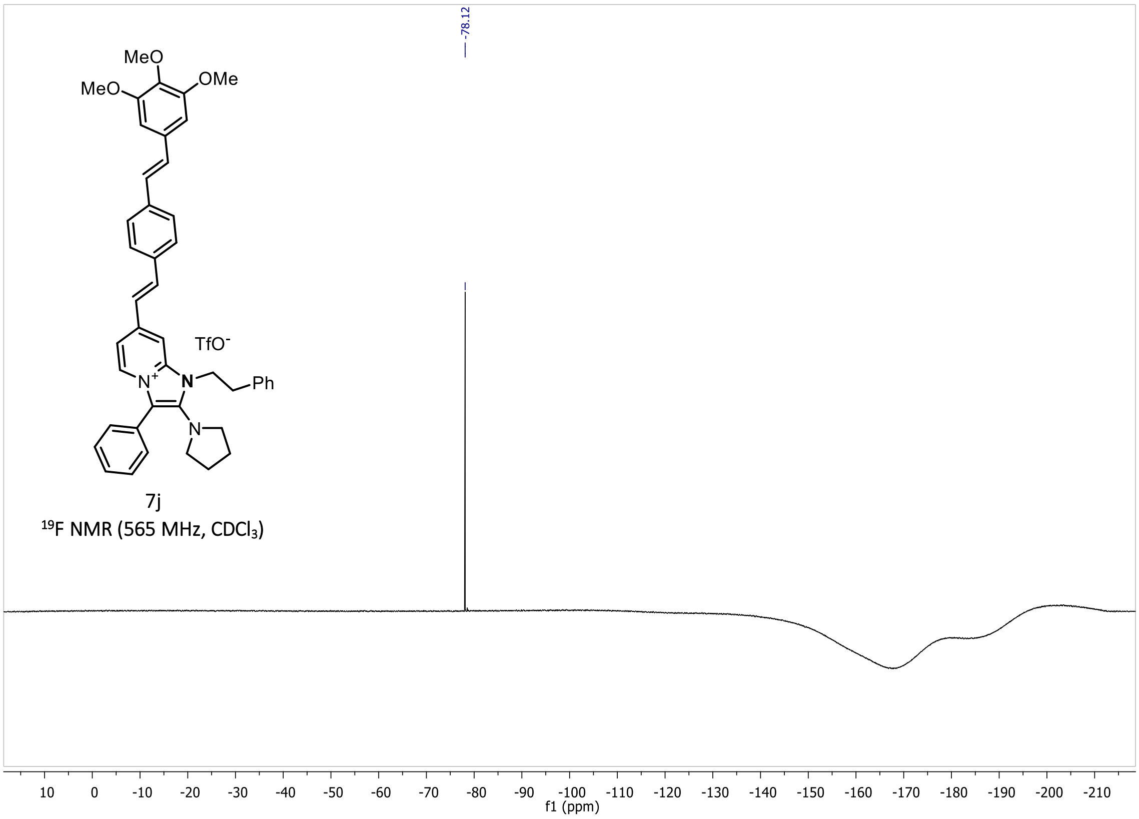

MeO
MeO
OMe
TfO-
N+
N
Ph
N
7j
19F NMR (565 MHz, CDCl3)
-78.12
f1 (ppm)

7-((1*E*,3*E*)-4-(4-(Diethylamino)phenyl)buta-1,3-dien-1-yl)-1-phenethyl-3-phenyl-2-(pyrrolidin-1-yl)-1H-imidazo[1,2-a]pyridin-4-ium trifluoromethanesulfonate (7k)

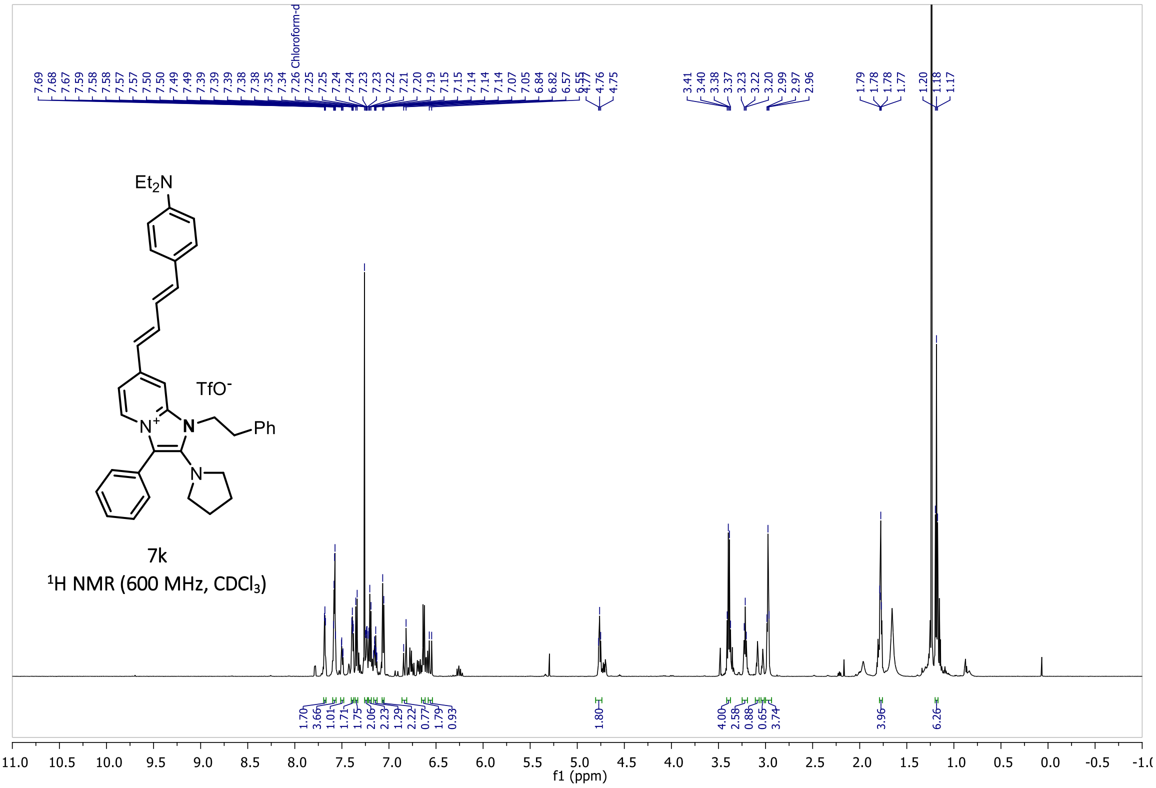

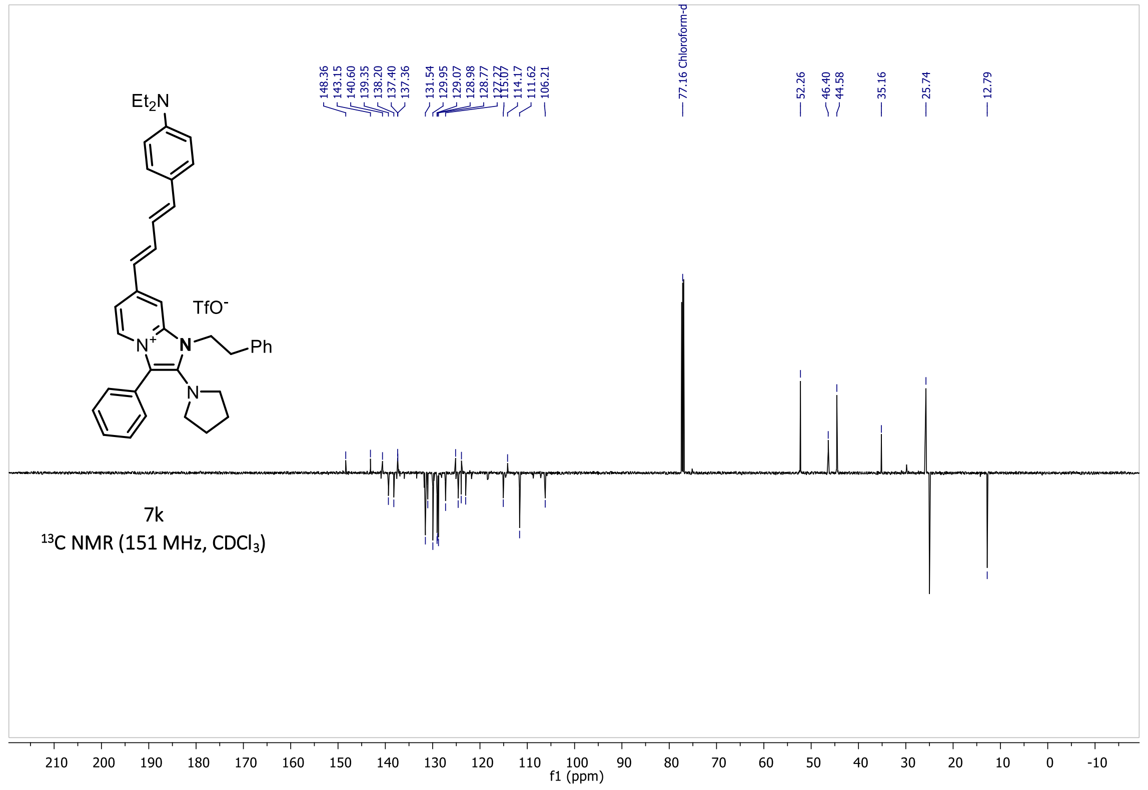

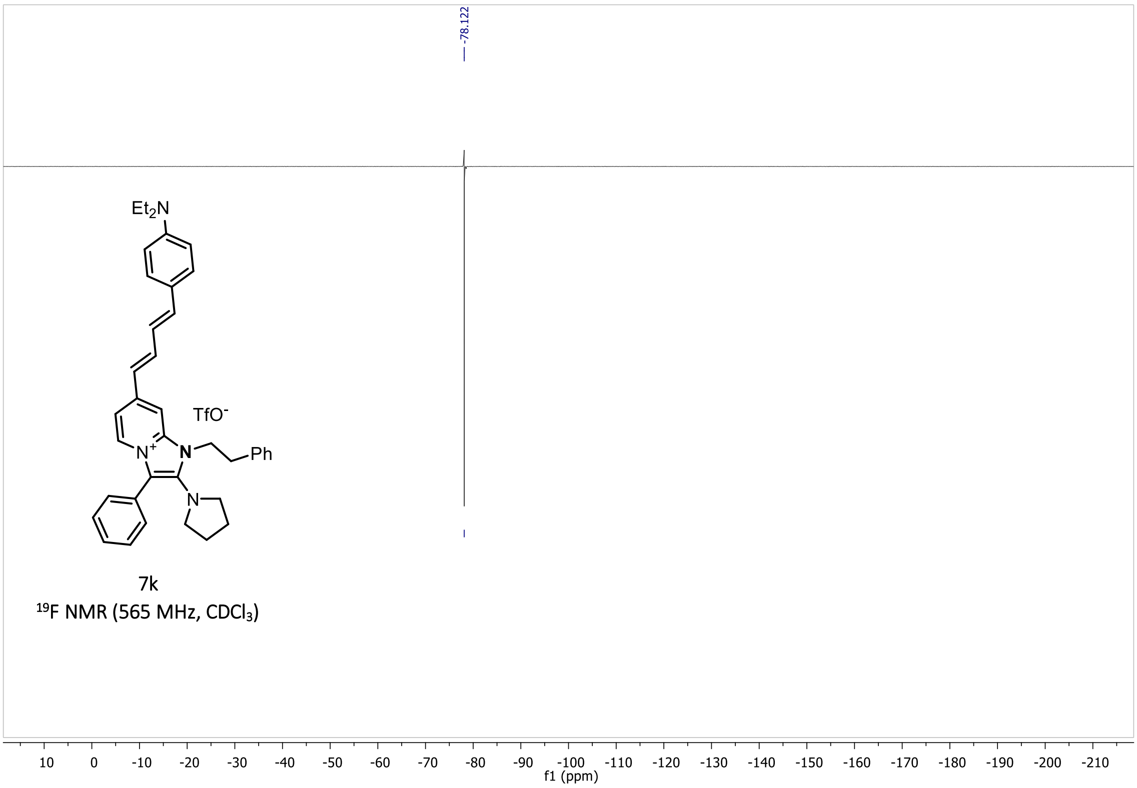

1-Phenethyl-3-phenyl-2-(pyrrolidin-1-yl)-7-((1*E*,3*E*)-4-(2,3,6,7-tetrahydro-1H,5H-pyrido[3,2,1-ij]quinolin-9-yl)buta-1,3-dien-1-yl)-1H-imidazo[1,2-a]pyridin-4-ium trifluoromethanesulfonate (7l)

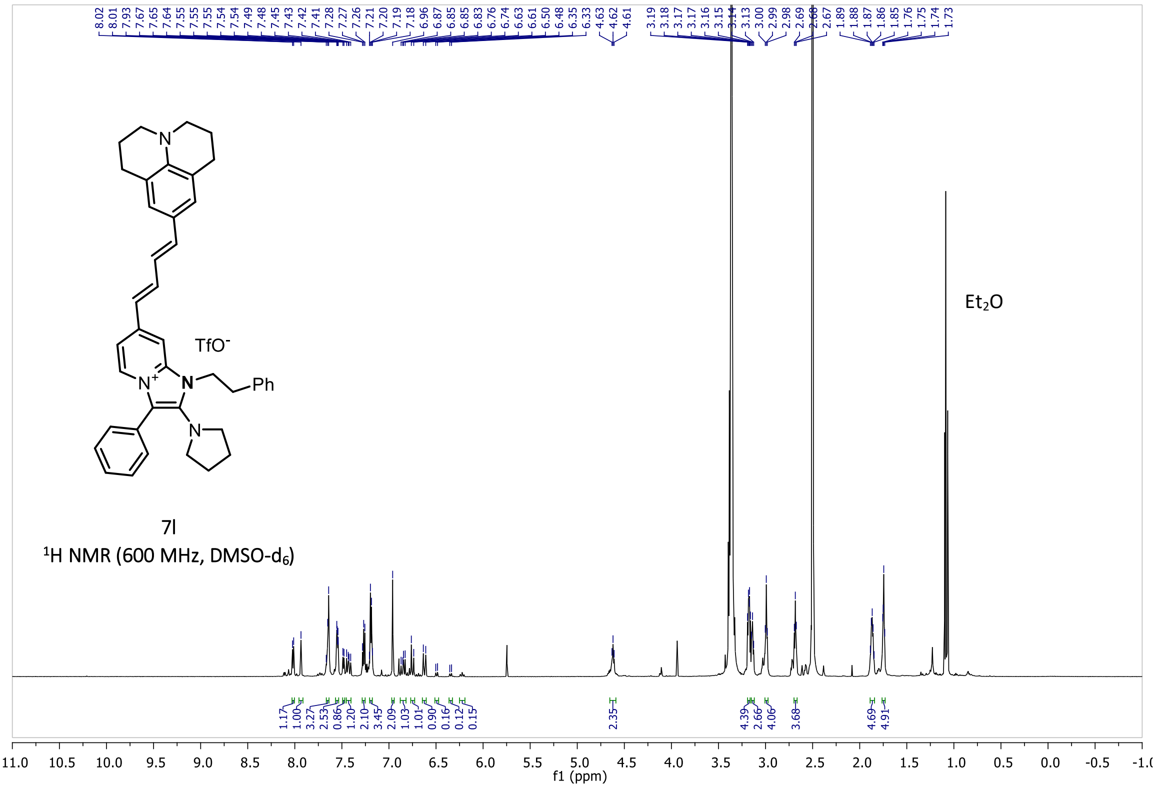

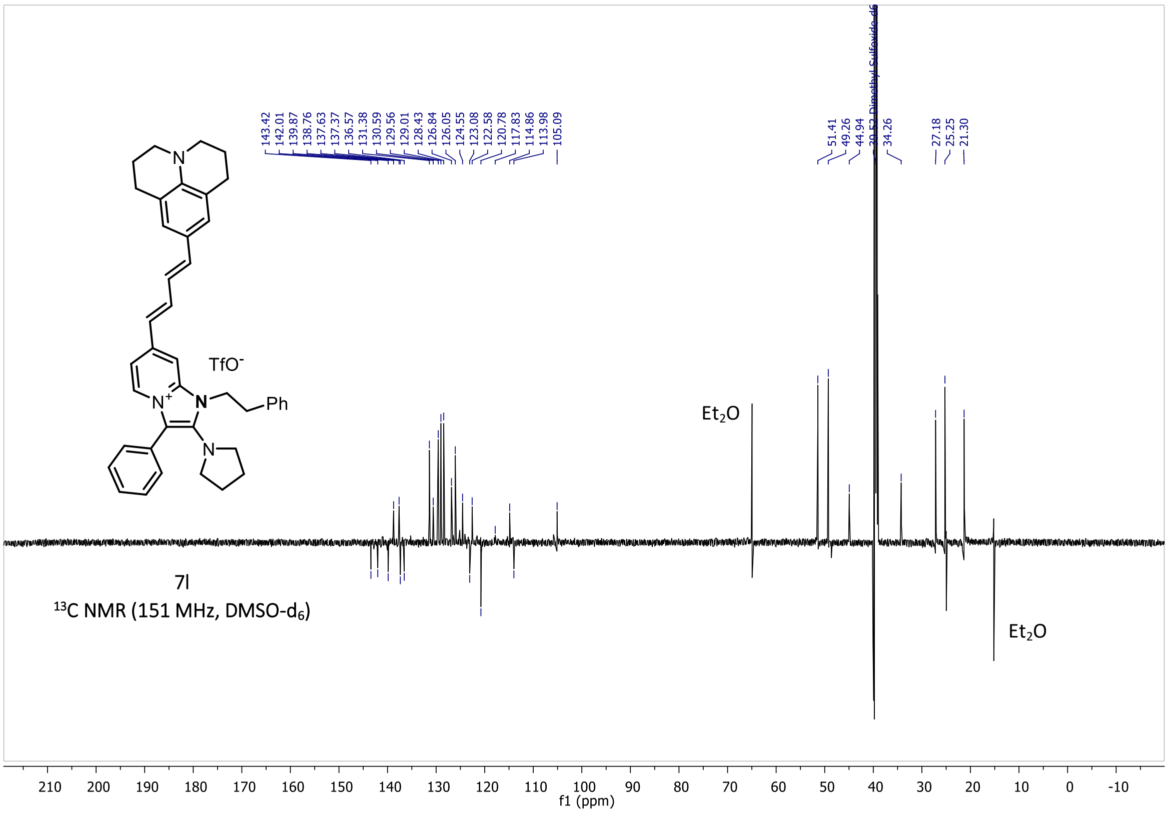
143.42
142.01
139.87
138.76
137.63
137.37
136.57
131.38
130.59
129.56
129.01
128.43
126.84
126.05
124.55
123.08
122.58
120.78
117.83
114.86
113.98
105.09
51.41
49.26
44.94
39.52 Dimethyl Sulfoxide-d6
34.26
27.18
25.25
21.30
TfO⁻
N⁺
N
Ph
N
Et2O
Et2O
7l
13C NMR (151 MHz, DMSO-d6)
f1 (ppm)

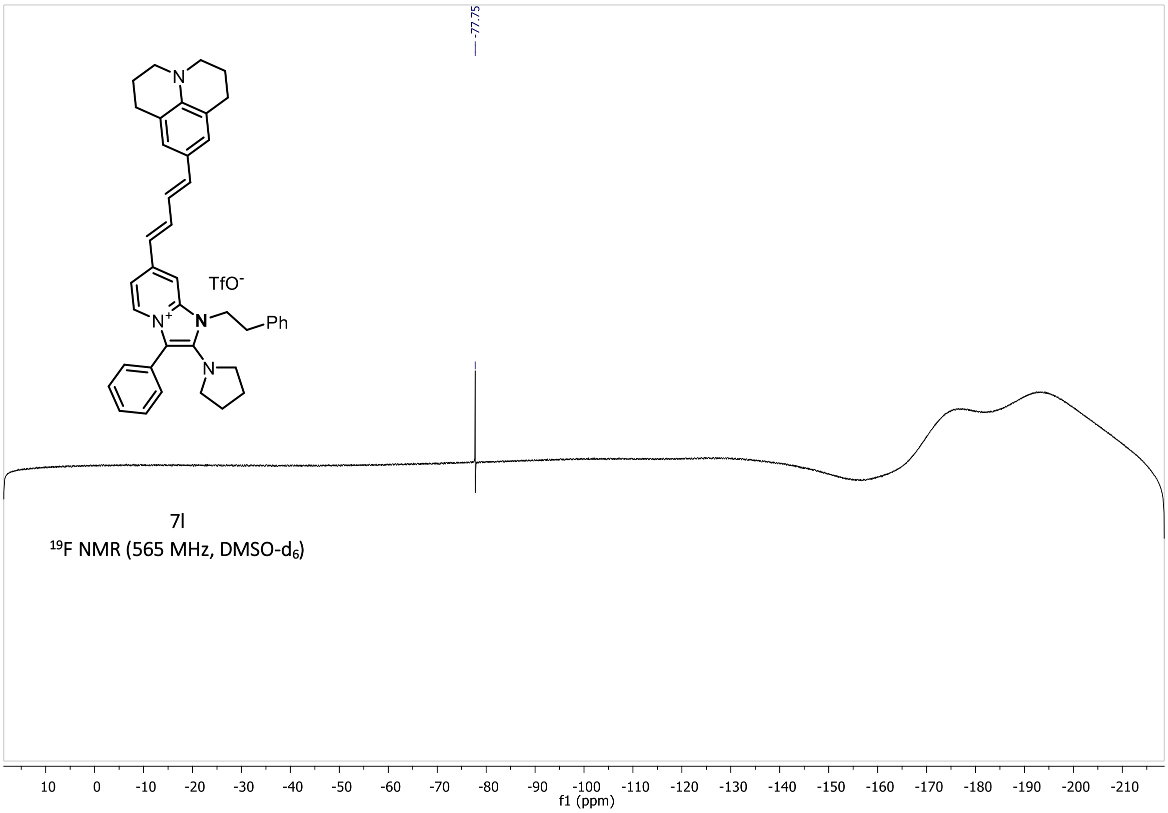
-77.75
TfO⁻
N⁺
N
Ph
N
7l
19F NMR (565 MHz, DMSO-d6)
f1 (ppm)

1-Phenethyl-3-phenyl-2-(pyrrolidin-1-yl)-7-((1*E*,3*E*)-4-(p-tolyl)buta-1,3-dien-1-yl)-1H-imidazo[1,2-a]pyridin-4-ium trifluoromethanesulfonate (7m)

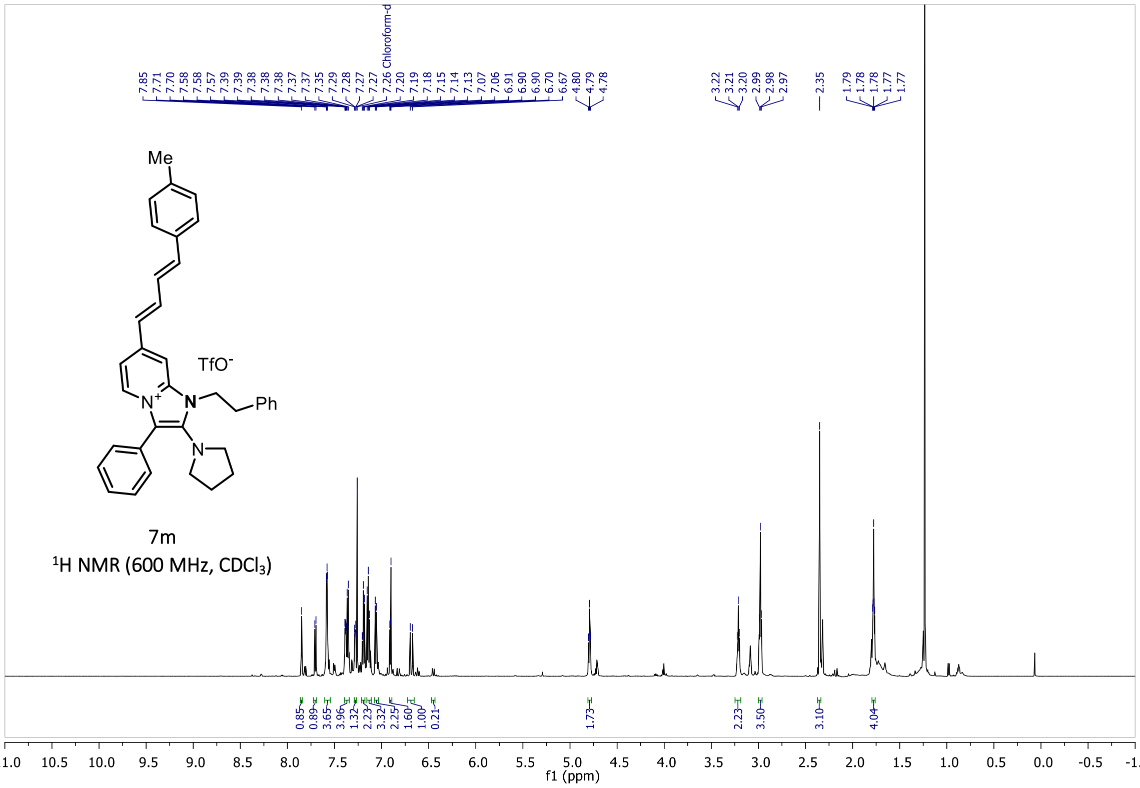

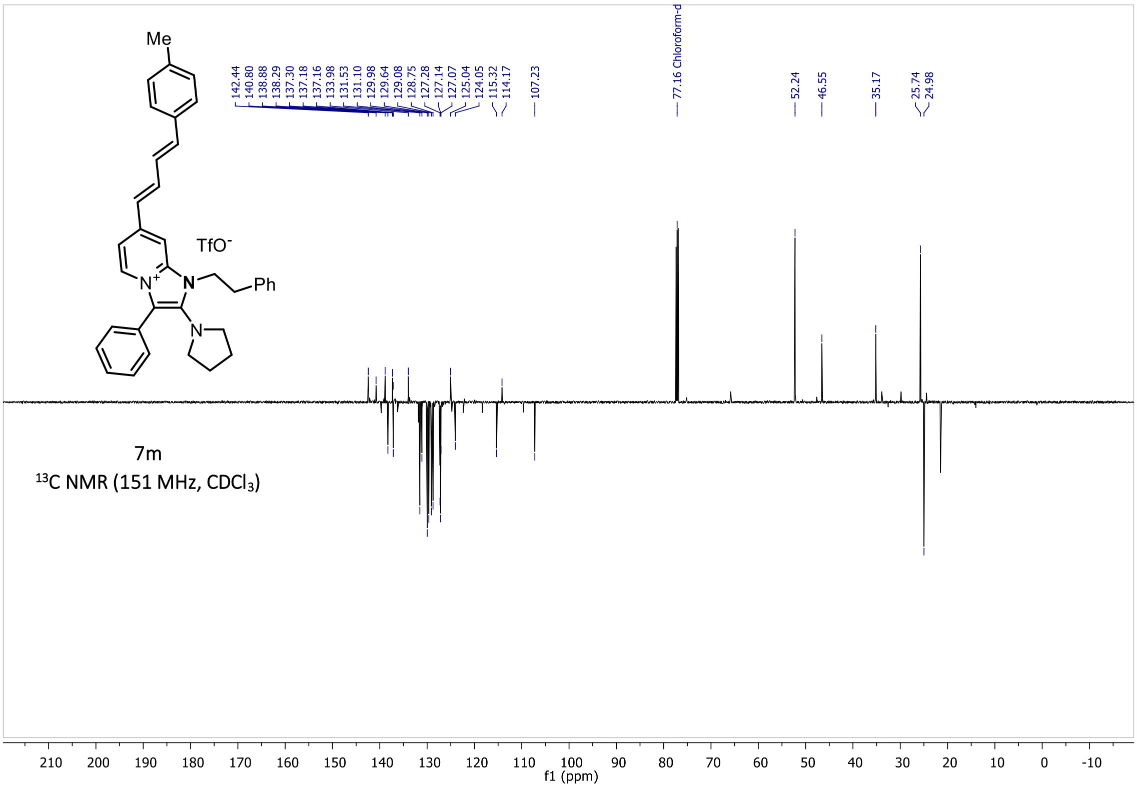

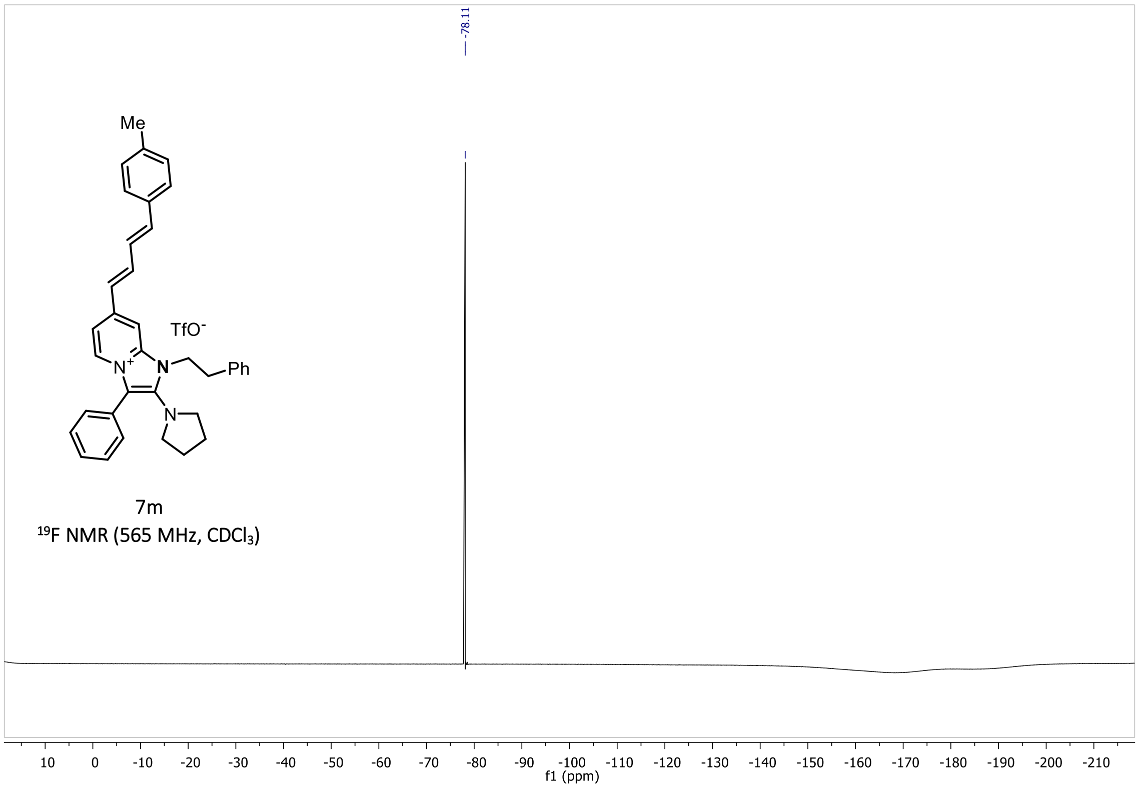

7-((1*E*,3*E*)-4-(4-Morpholinophenyl)buta-1,3-dien-1-yl)-1-phenethyl-3-phenyl-2-(pyrrolidin-1-yl)-1H-imidazo[1,2-a]pyridin-4-ium trifluoromethanesulfonate (7n)

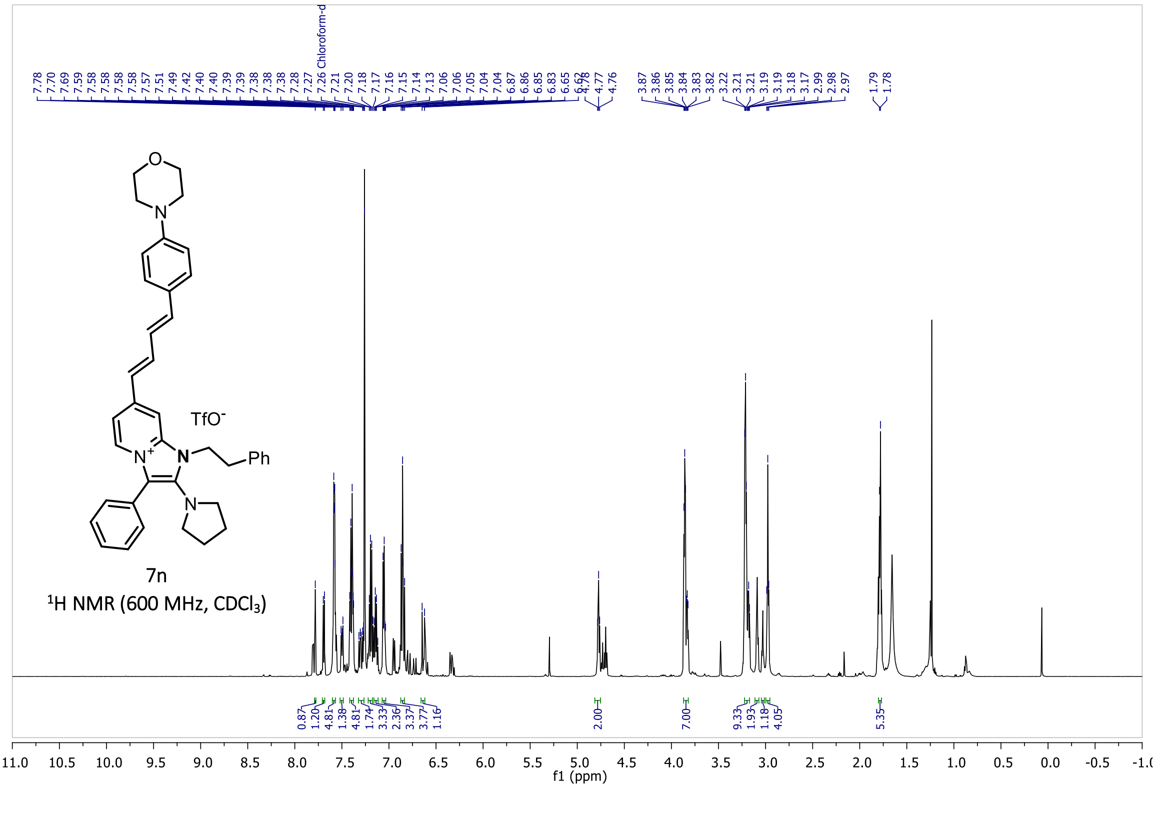

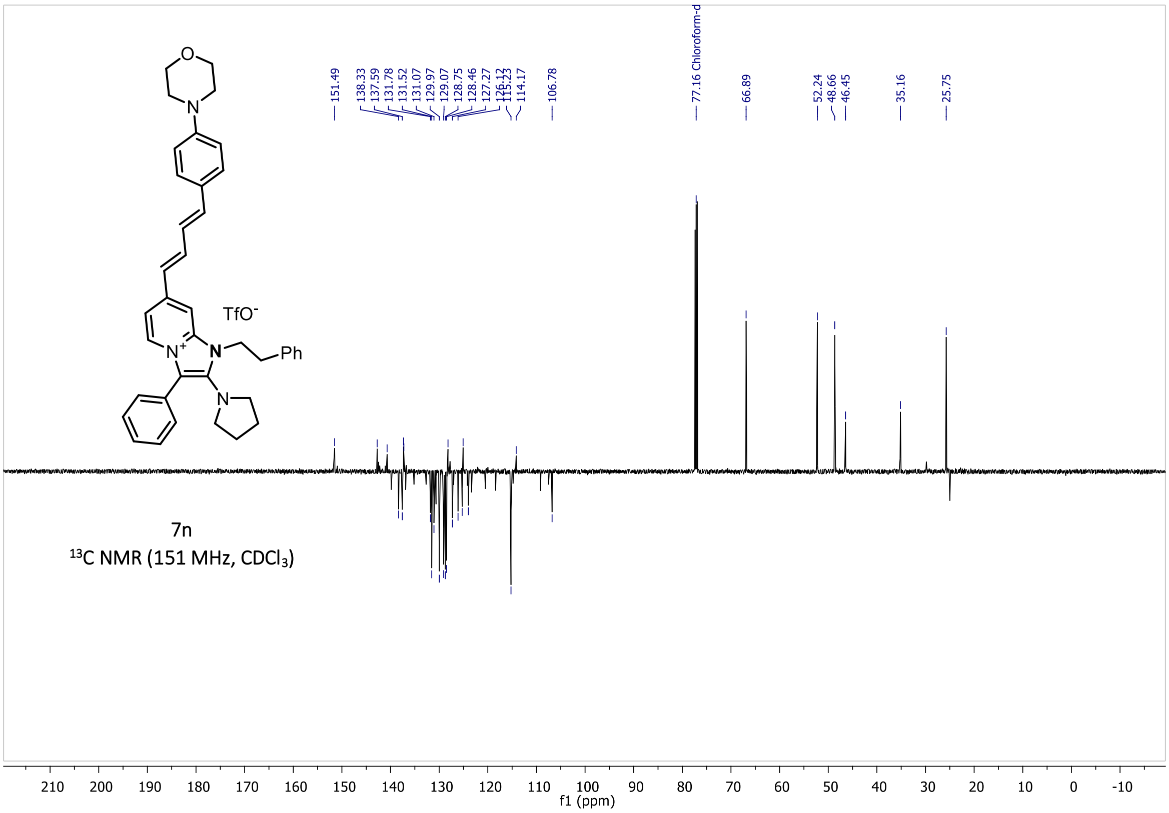
151.49
138.33
137.59
131.78
131.52
131.07
129.97
129.07
128.75
128.46
127.27
126.12
115.23
114.17
106.78
77.16 Chloroform-d
66.89
52.24
48.66
46.45
35.16
25.75
TfO⁻
N⁺
N
Ph
N
O
7n
^{13}C NMR (151 MHz, $CDCl_3$)
210 200 190 180 170 160 150 140 130 120 110 100 90 80 70 60 50 40 30 20 10 0 -10
f1 (ppm)

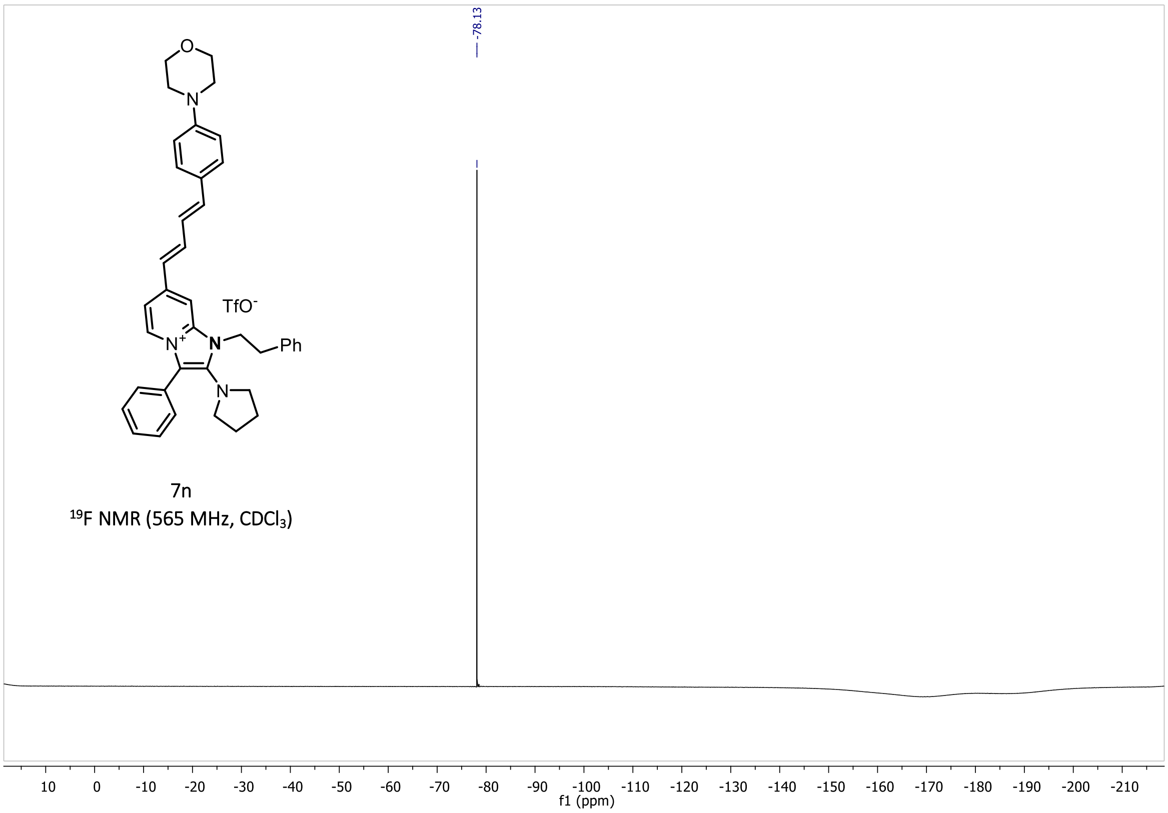
-78.13
TfO⁻
N⁺
N
Ph
N
O
7n
^{19}F NMR (565 MHz, $CDCl_3$)
10 0 -10 -20 -30 -40 -50 -60 -70 -80 -90 -100 -110 -120 -130 -140 -150 -160 -170 -180 -190 -200 -210
f1 (ppm)

1-Phenethyl-3-phenyl-2-(pyrrolidin-1-yl)-7-((1*E*,3*E*,5*E*)-6-(4-(trifluoromethyl)phenyl)hexa-1,3,5-trien-1-yl)-1H-imidazo[1,2-a]pyridin-4-ium trifluoromethanesulfonate (7o)

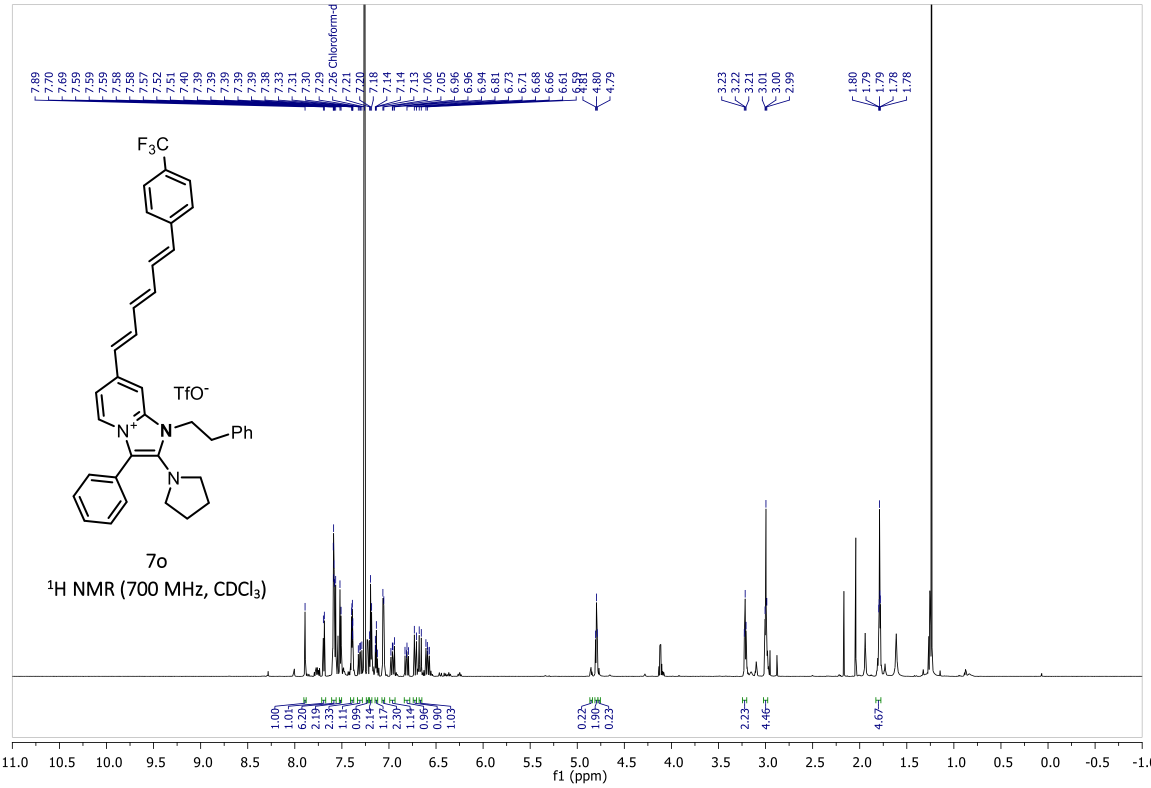

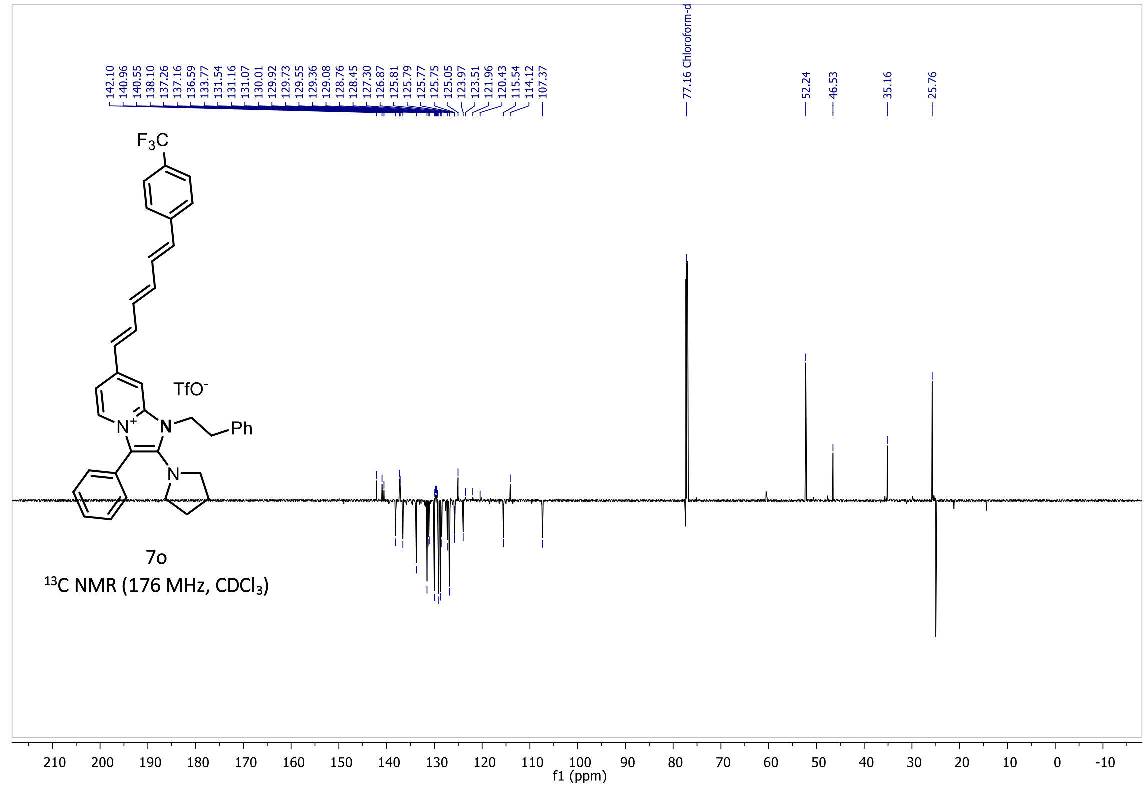

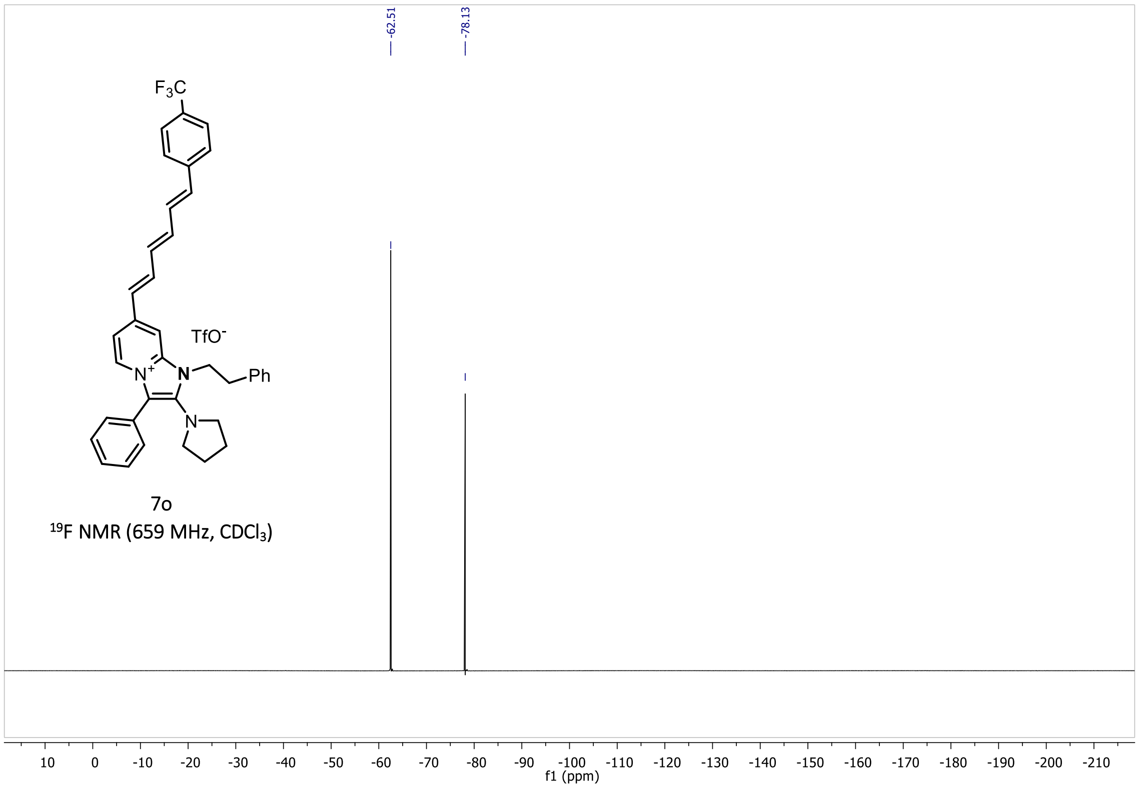

7-(4-(Diethylamino)phenyl)-3-phenyl-2-(pyrrolidin-1-yl)oxazolo[3,2-a]pyridin-4-ium trifluoromethanesulfonate (8a)

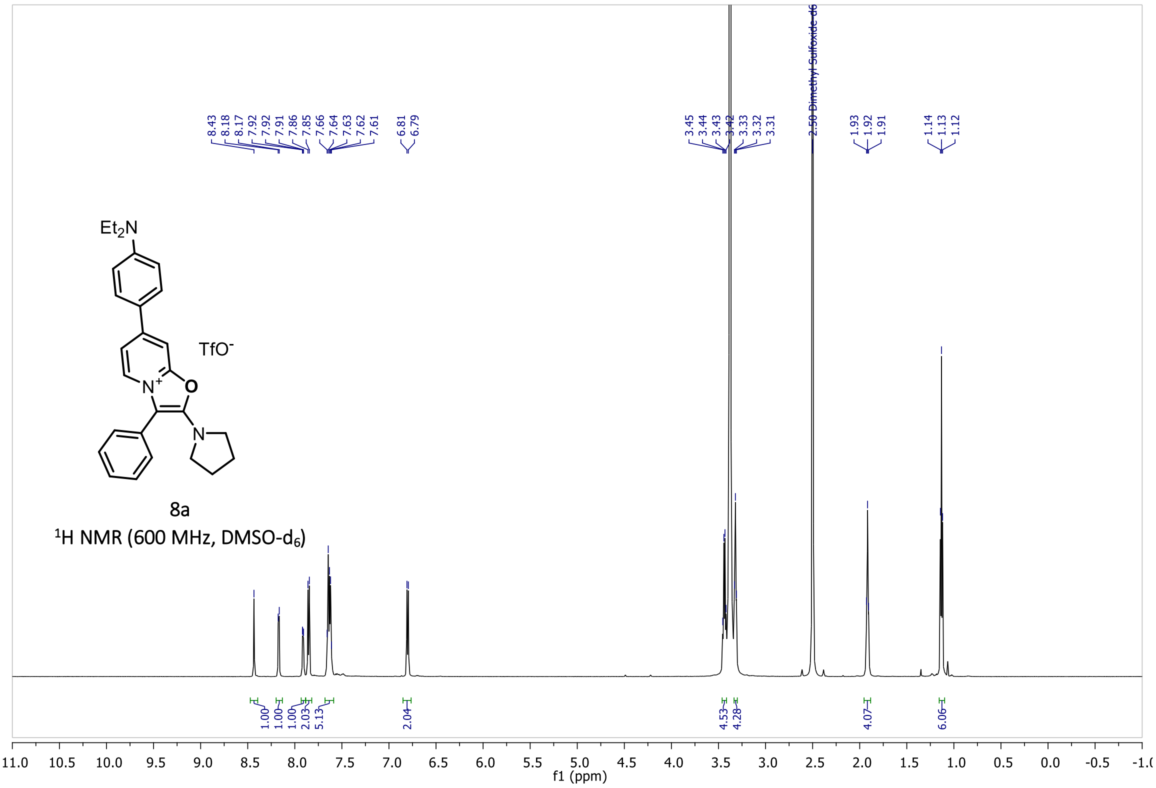

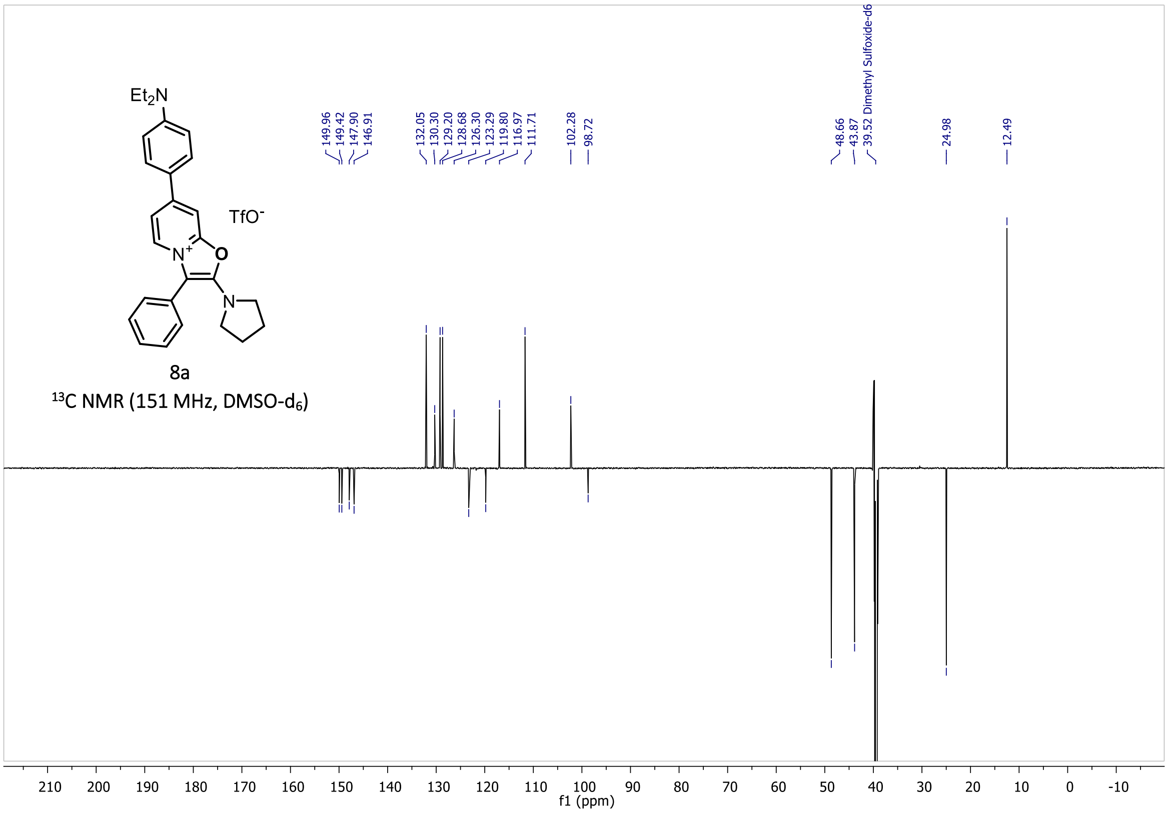

8a

^{13}C NMR (151 MHz, DMSO-d_6)

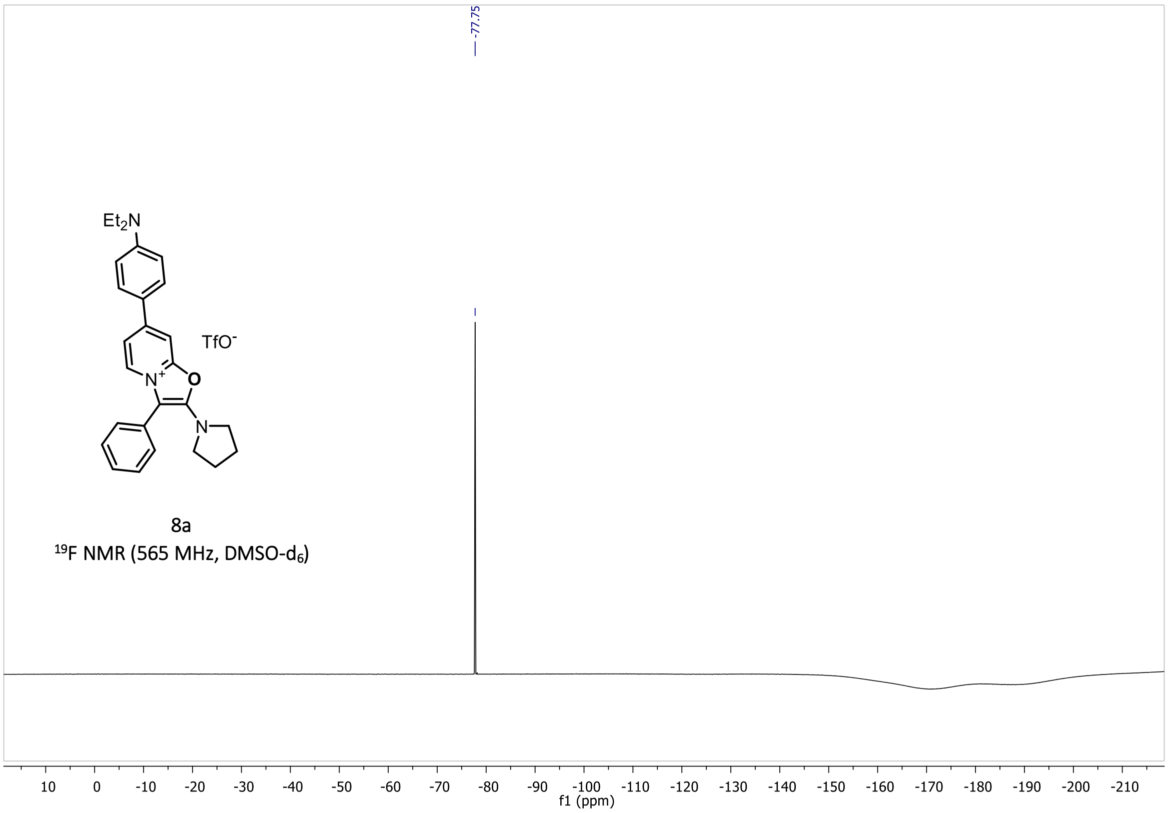

8a

^{19}F NMR (565 MHz, DMSO-d_6)

3-Phenyl-2-(pyrrolidin-1-yl)-7-(2,3,6,7-tetrahydro-1H,5H-pyrido[3,2,1-ij]quinolin-9-yl)oxazolo[3,2-a]pyridin-4-ium trifluoromethanesulfonate (8b)

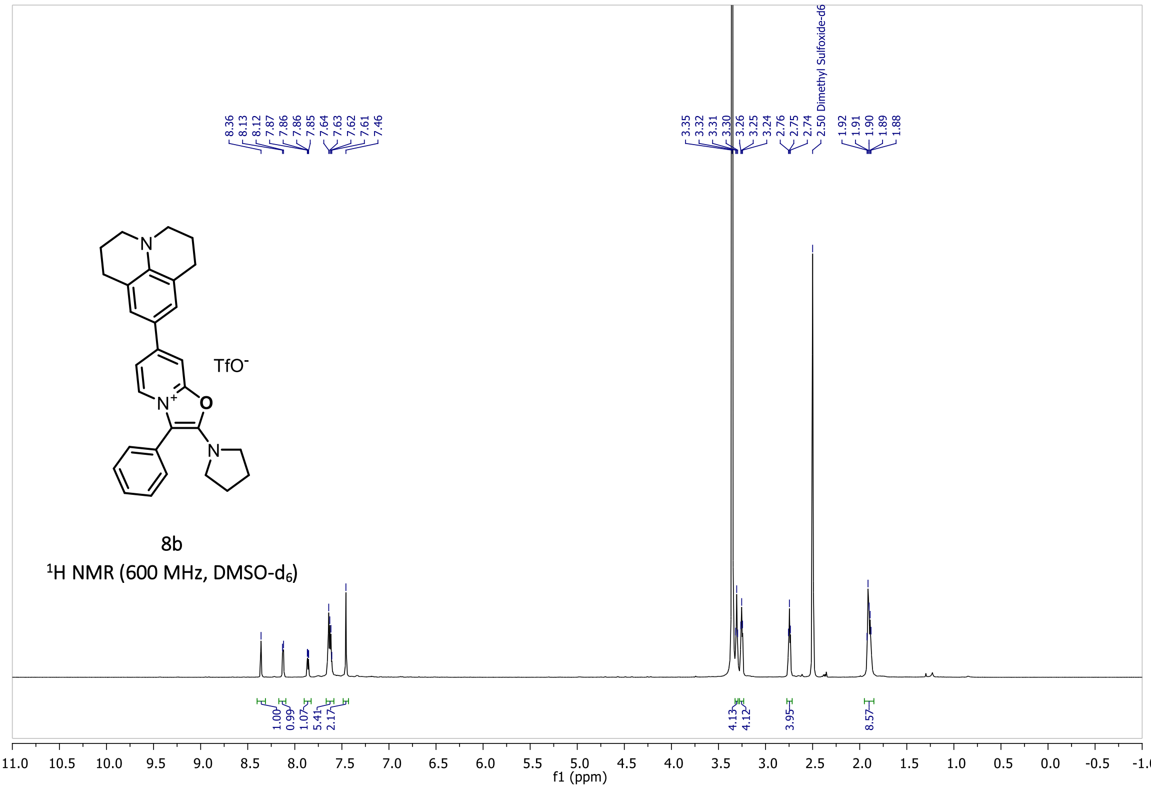

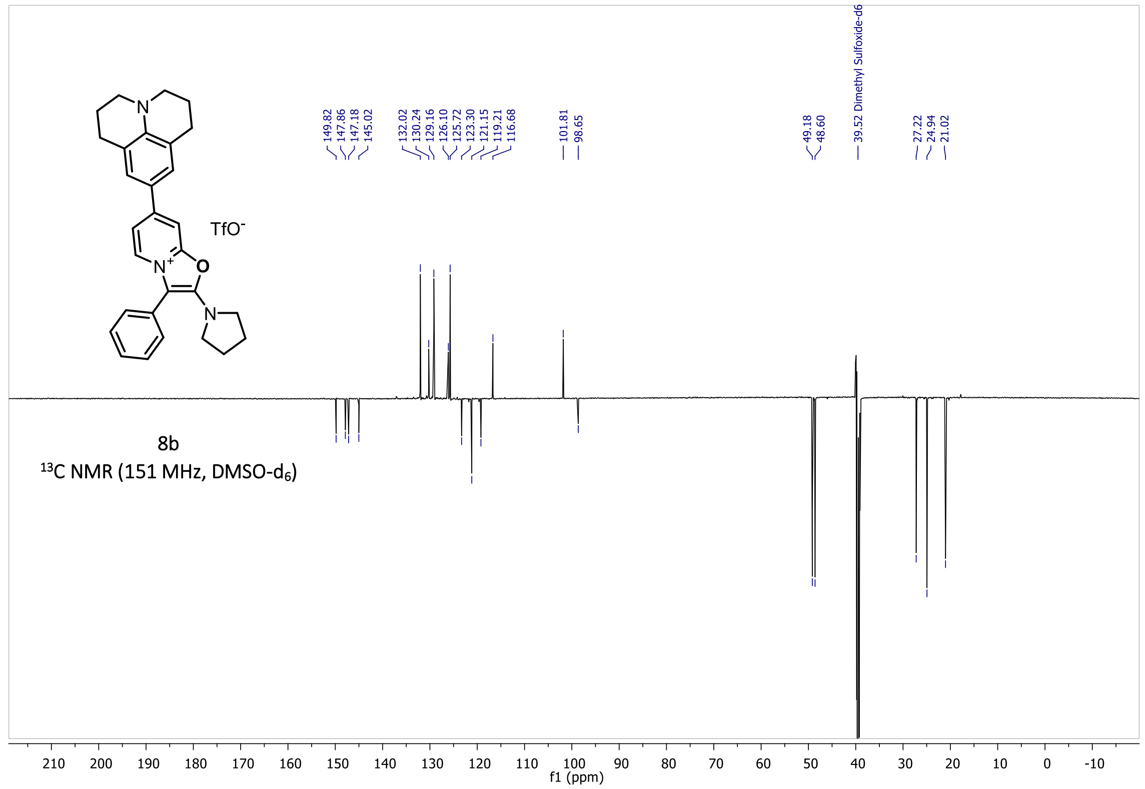

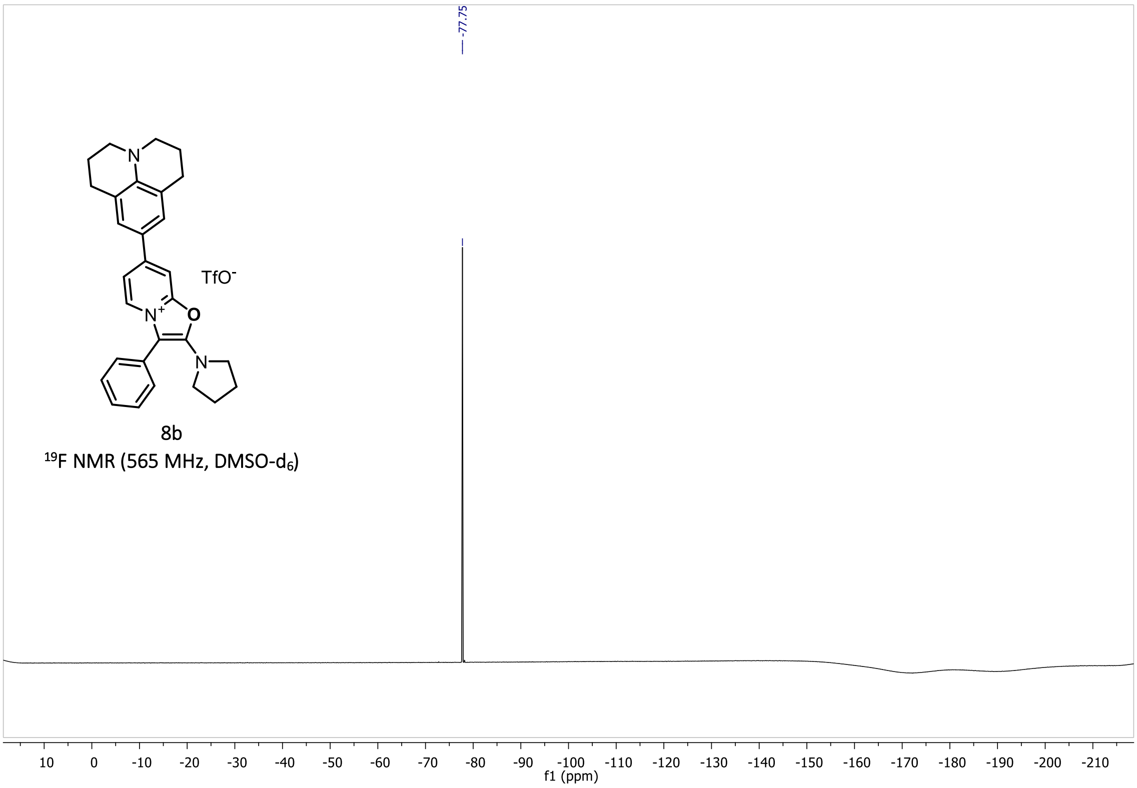

7-(4'-(Diethylamino)-[1,1'-biphenyl]-4-yl)-3-phenyl-2-(pyrrolidin-1-yl)oxazolo[3,2-a]pyridin-4-ium trifluoromethanesulfonate (8c)

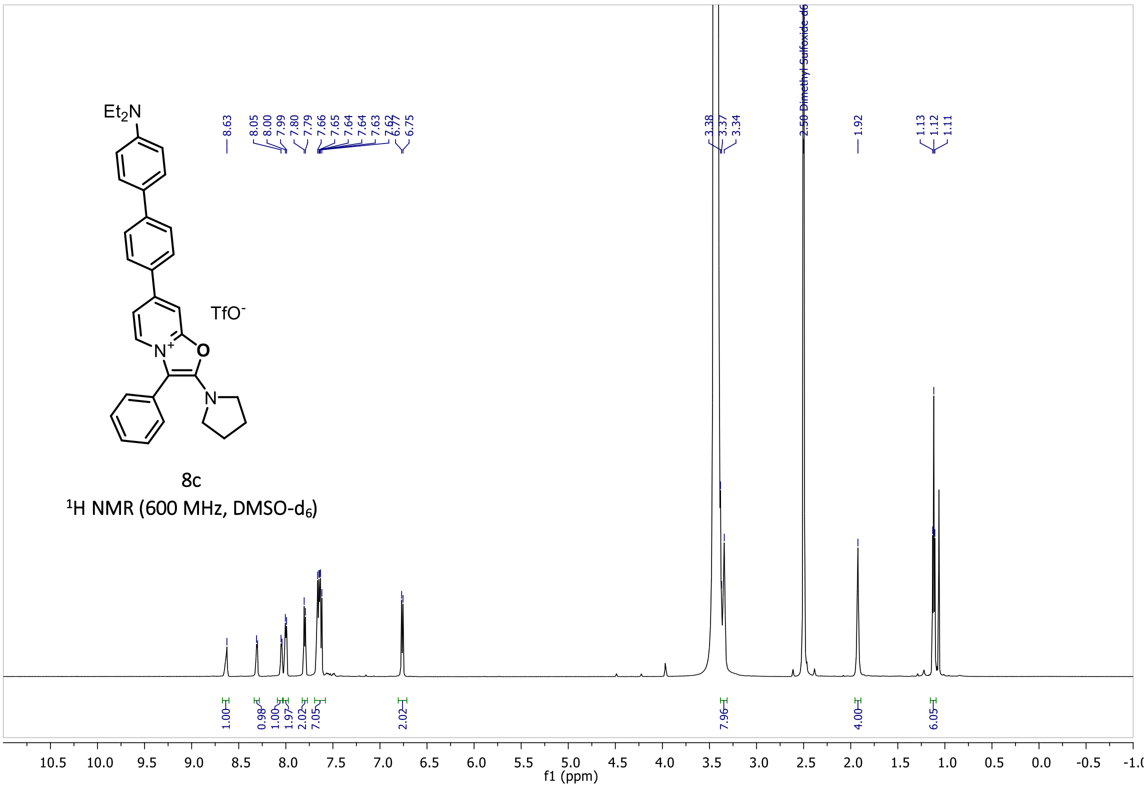

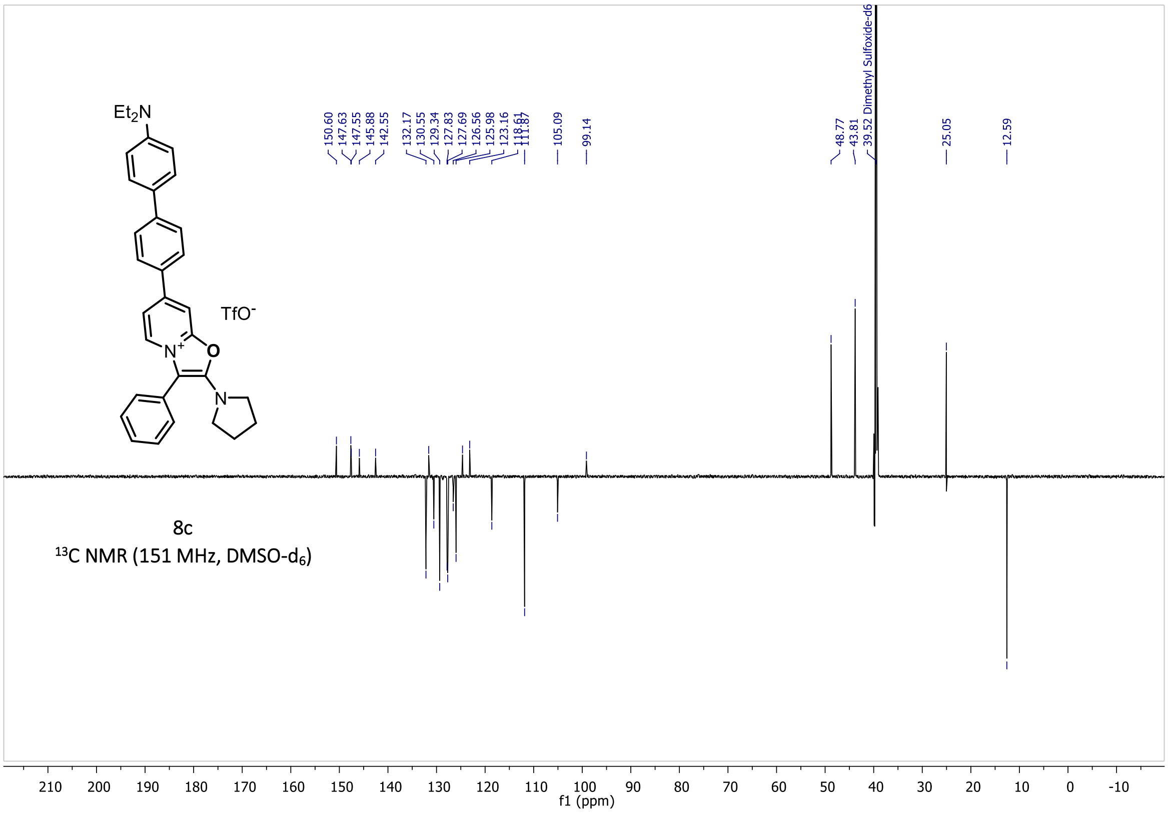

8c

^{13}C NMR (151 MHz, DMSO-d$_6$)

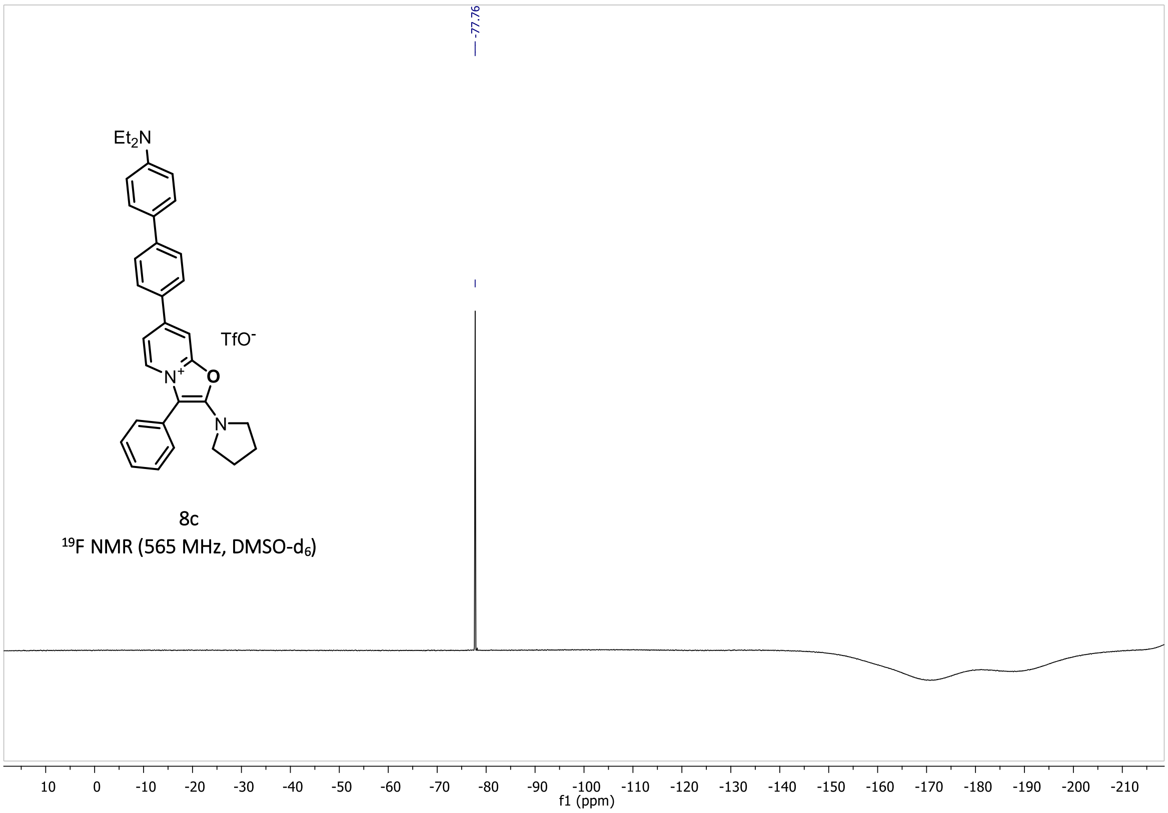

8c

^{19}F NMR (565 MHz, DMSO-d$_6$)

(*E*)-7-(4-(Diethylamino)styryl)-3-phenyl-2-(pyrrolidin-1-yl)oxazolo[3,2-a]pyridin-4-ium trifluoromethanesulfonate (8d)

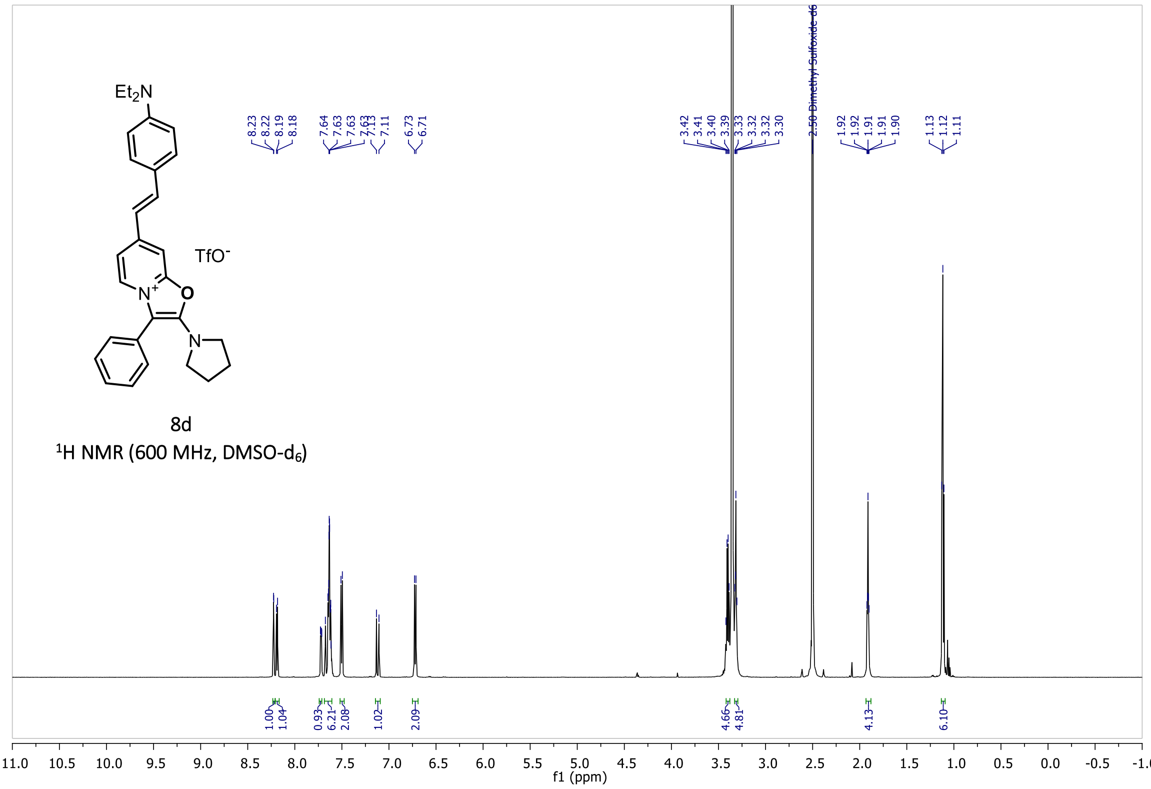

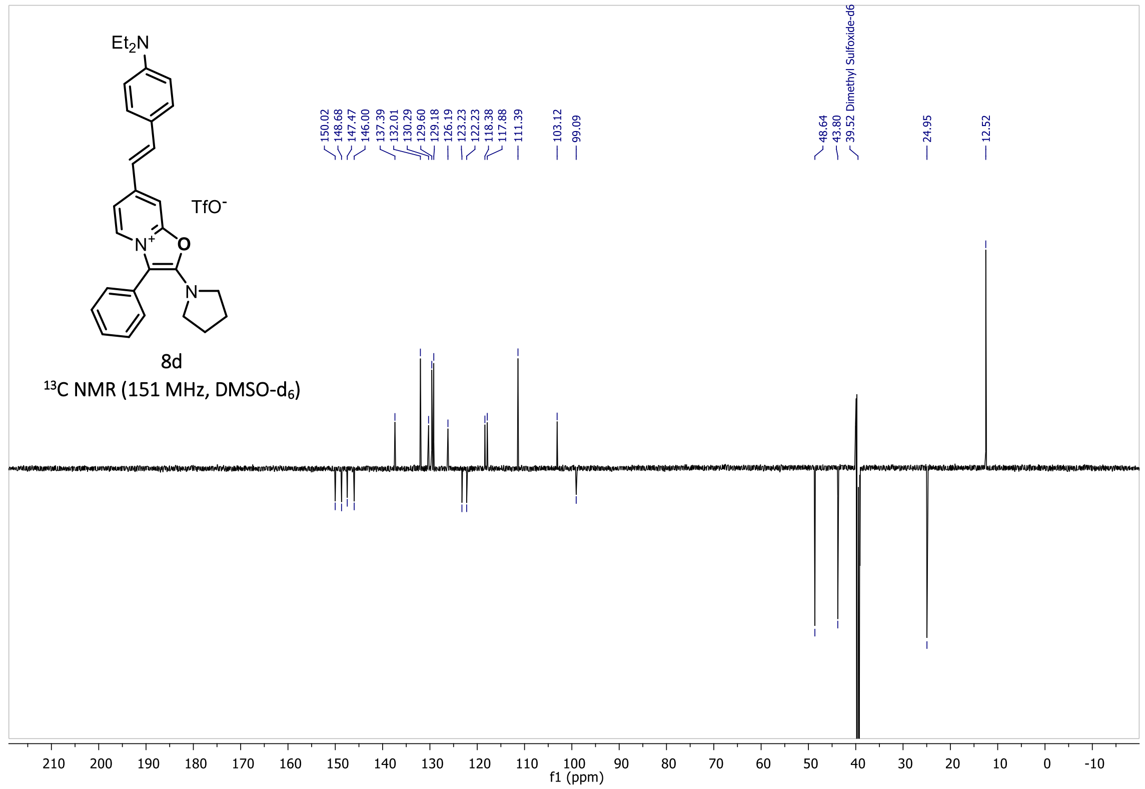

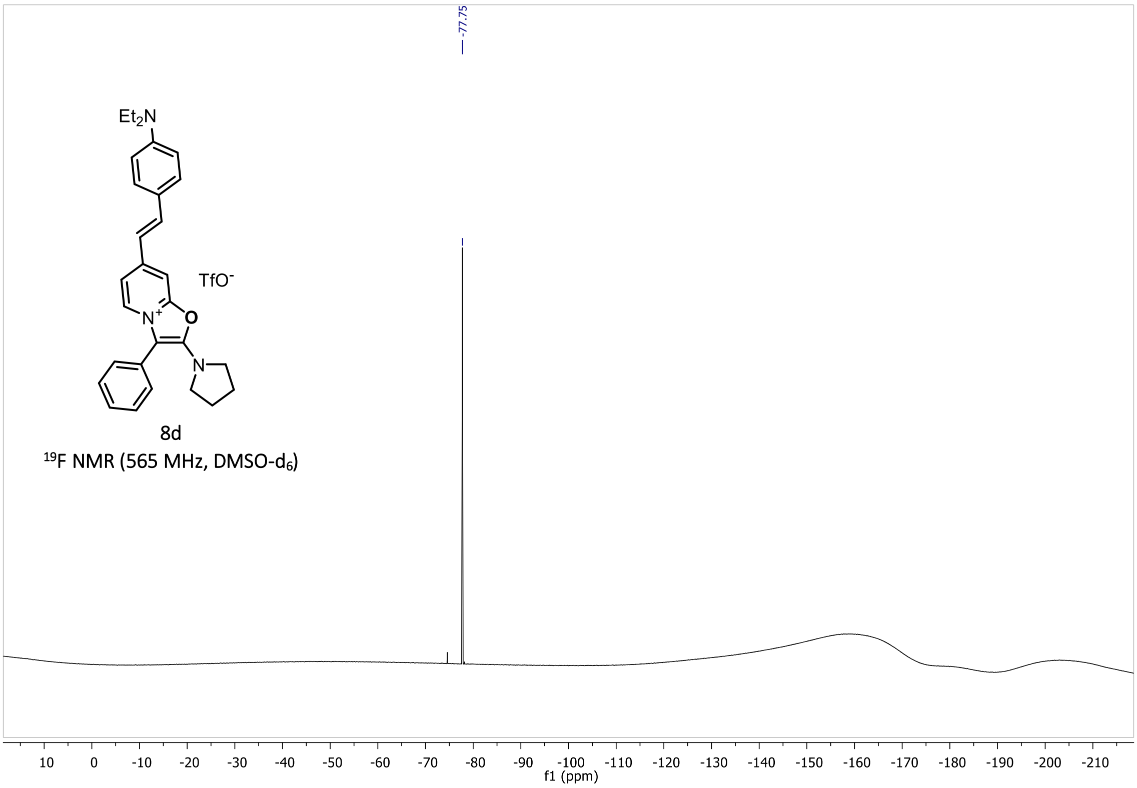

(*E*)-7-(4-(Dimethylamino)styryl)-3-phenyl-2-(pyrrolidin-1-yl)oxazolo[3,2-a]pyridin-4-ium trifluoromethanesulfonate (8e)

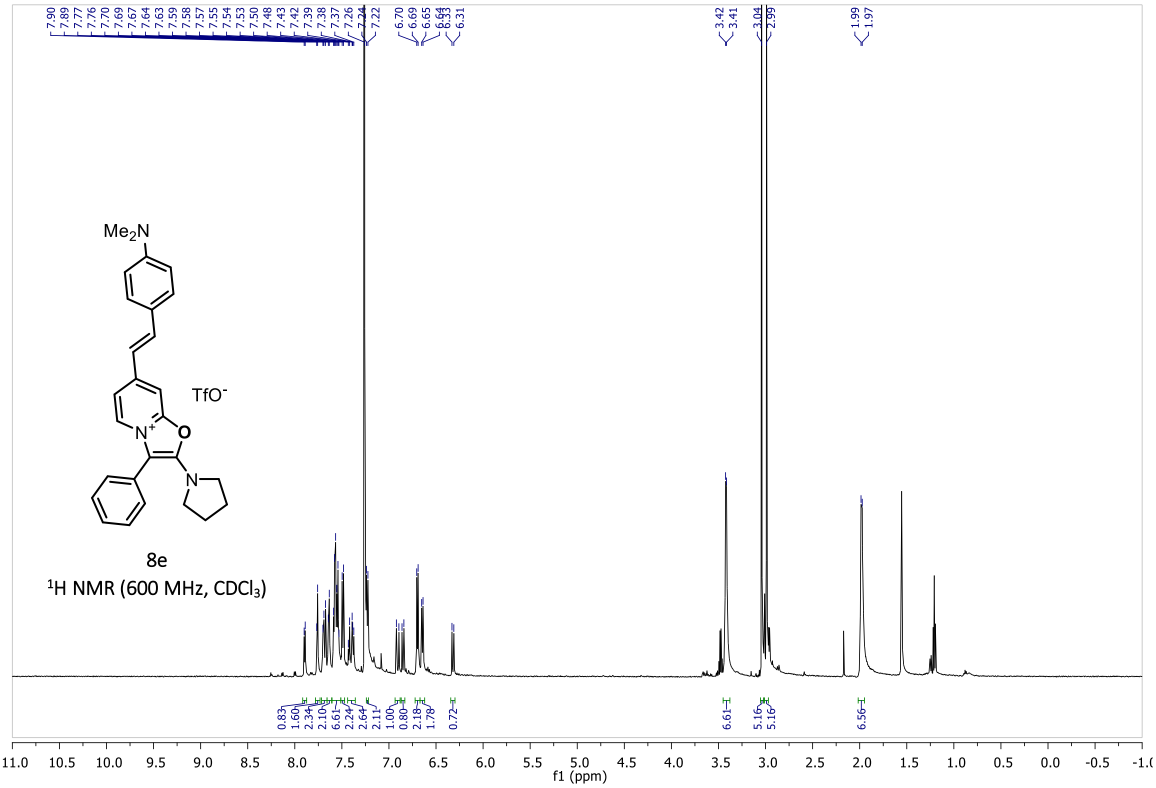

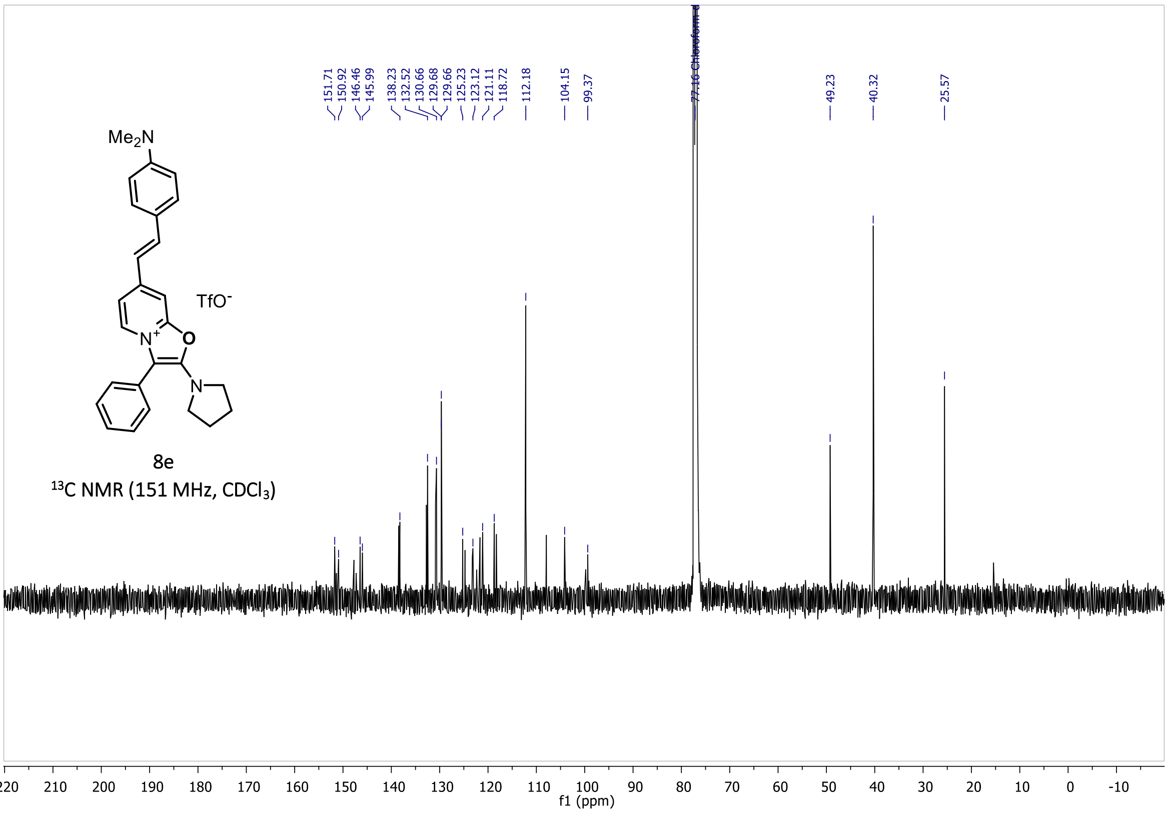
Me2N
TfO-
N+
O
N
8e
13C NMR (151 MHz, CDCl3)
151.71
150.92
146.46
145.99
138.23
132.52
130.66
129.68
129.66
125.23
123.12
121.11
118.72
112.18
104.15
99.37
77.16 Chloroform-d
49.23
40.32
25.57
220 210 200 190 180 170 160 150 140 130 120 110 100 90 80 70 60 50 40 30 20 10 0 -10
f1 (ppm)

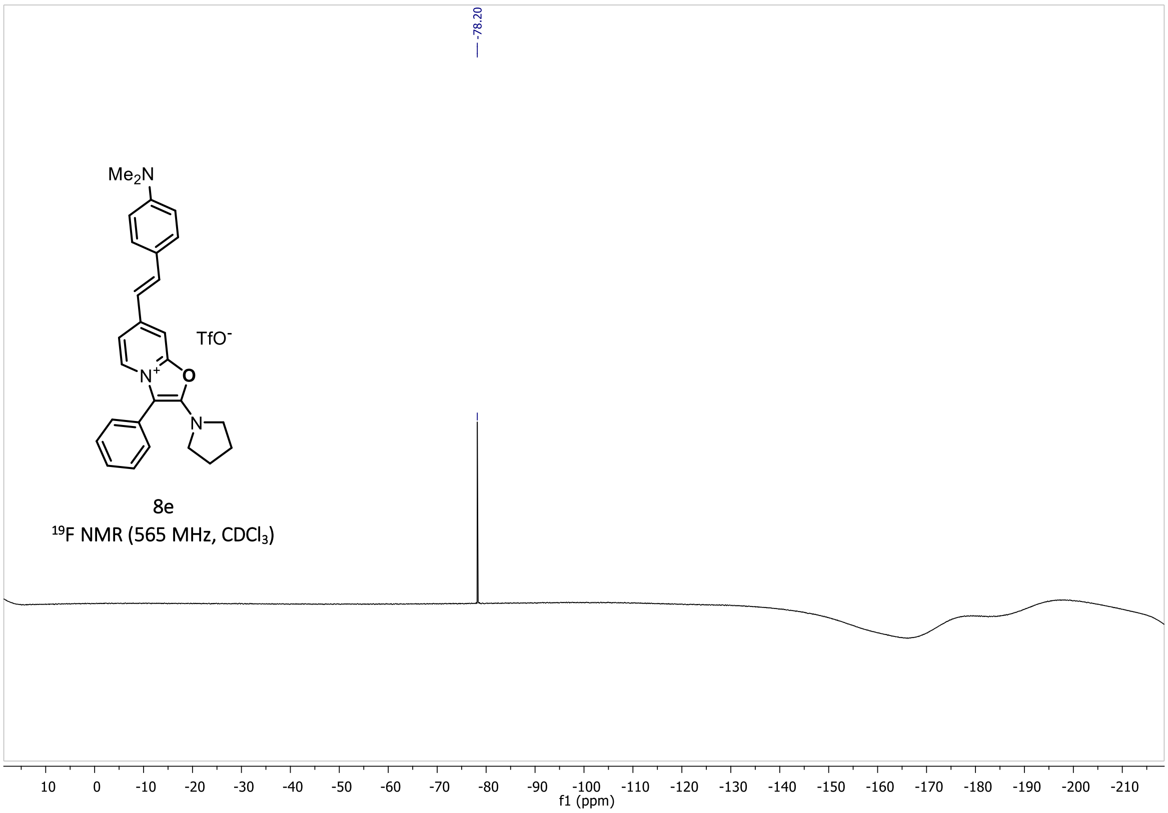
Me2N
TfO-
N+
O
N
8e
19F NMR (565 MHz, CDCl3)
-78.20
10 0 -10 -20 -30 -40 -50 -60 -70 -80 -90 -100 -110 -120 -130 -140 -150 -160 -170 -180 -190 -200 -210
f1 (ppm)

(*E*)-3-Phenyl-2-(pyrrolidin-1-yl)-7-(2-(2,3,6,7-tetrahydro-1H,5H-pyrido[3,2,1-ij]quinolin-9-yl)vinyl)oxazolo[3,2-a]pyridin-4-ium trifluoromethanesulfonate (8f)

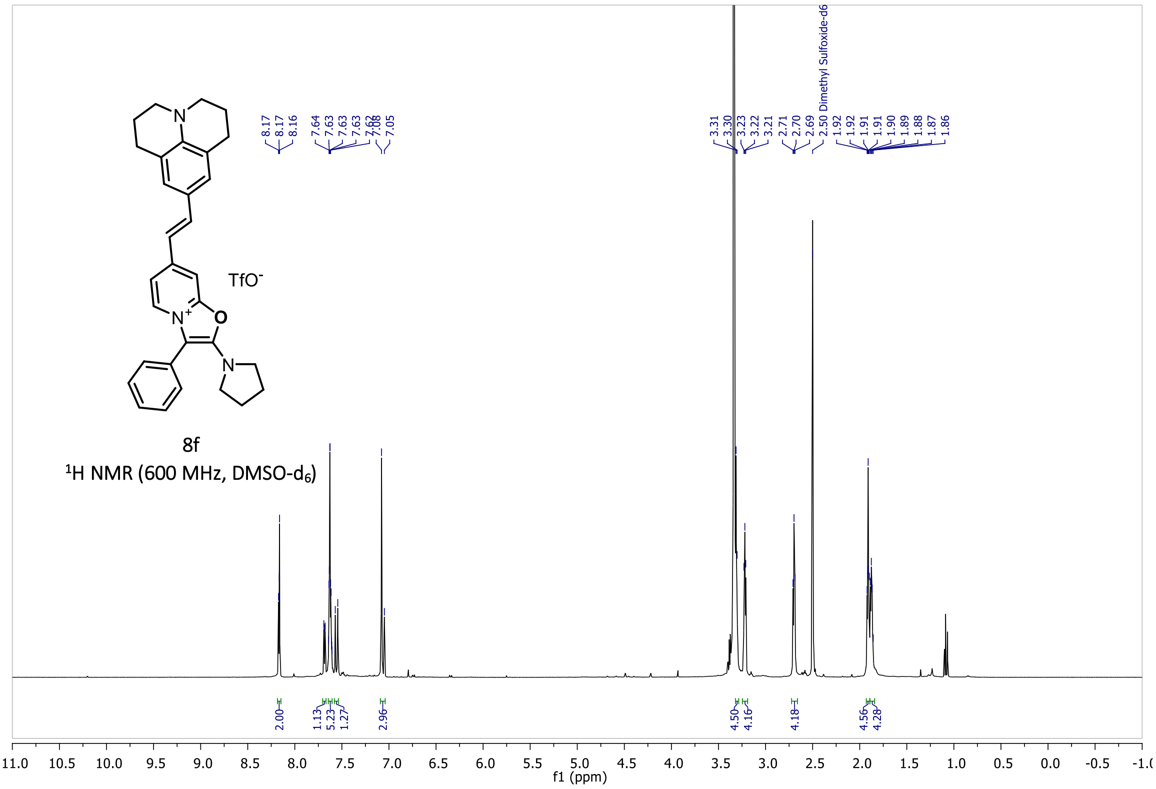

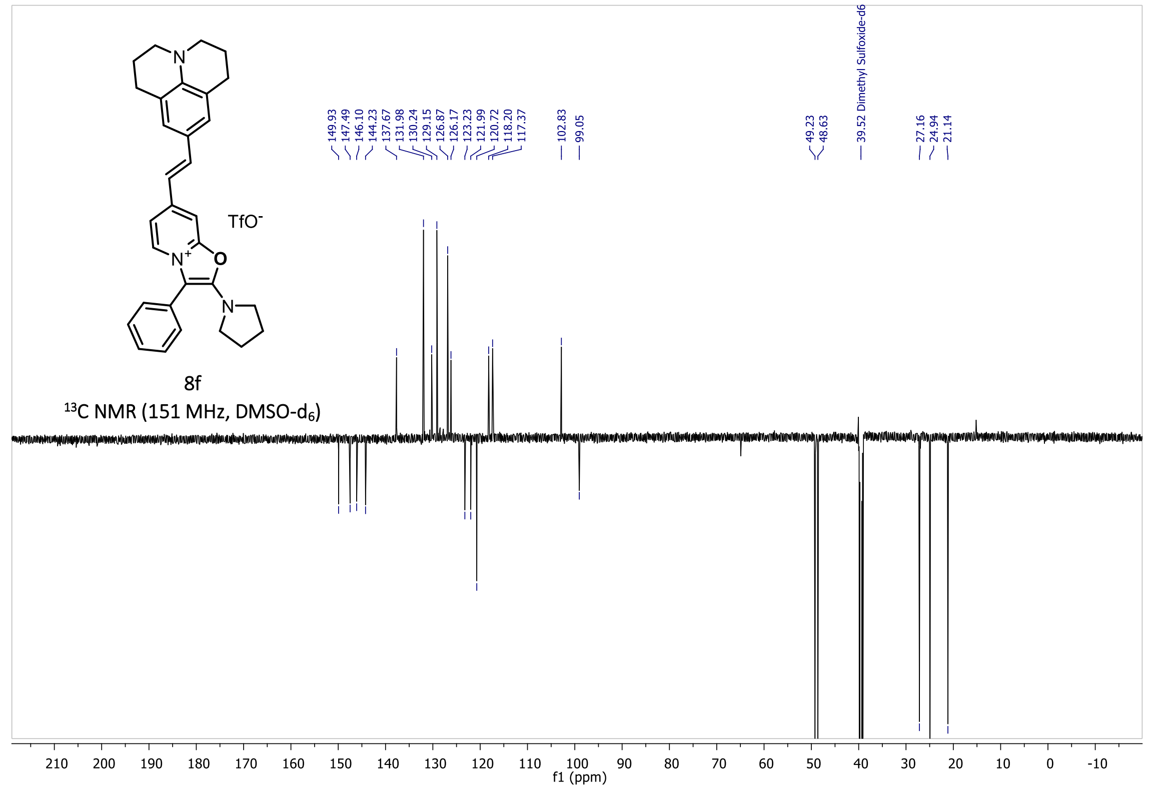

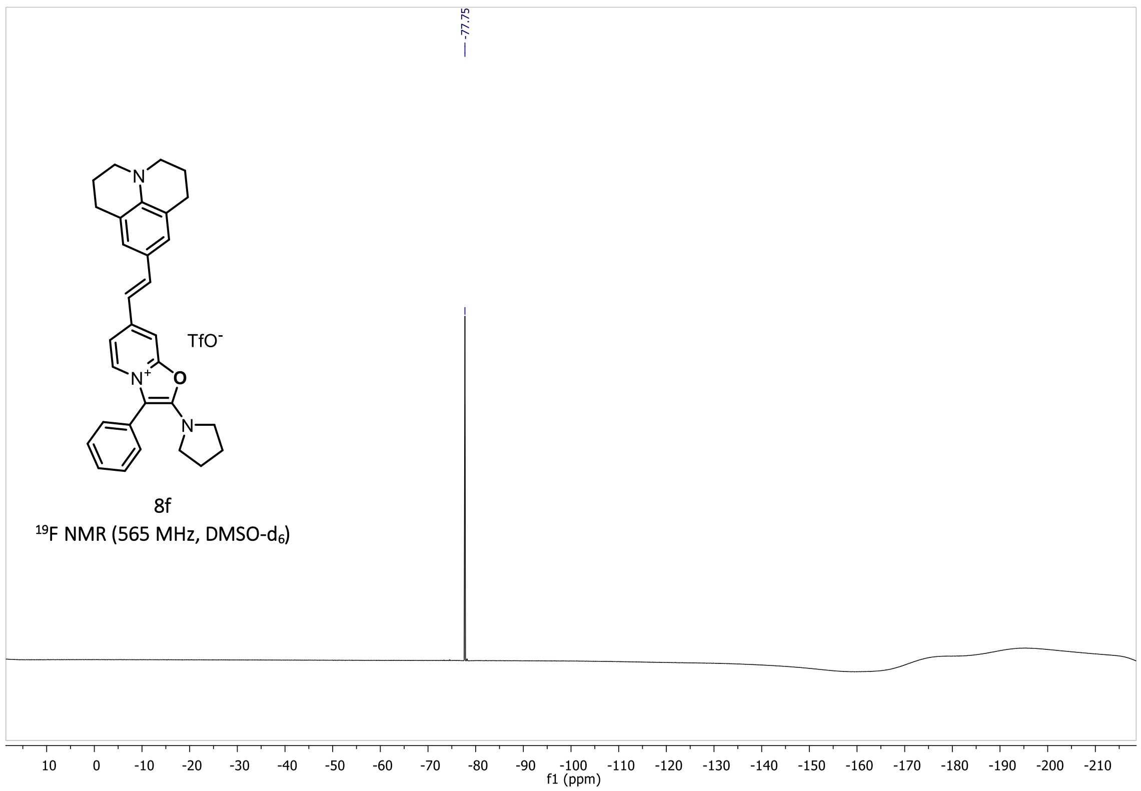

(*E*)-7-(4-Morpholinostyryl)-3-phenyl-2-(pyrrolidin-1-yl)oxazolo[3,2-a]pyridin-4-ium trifluoromethanesulfonate (8g)

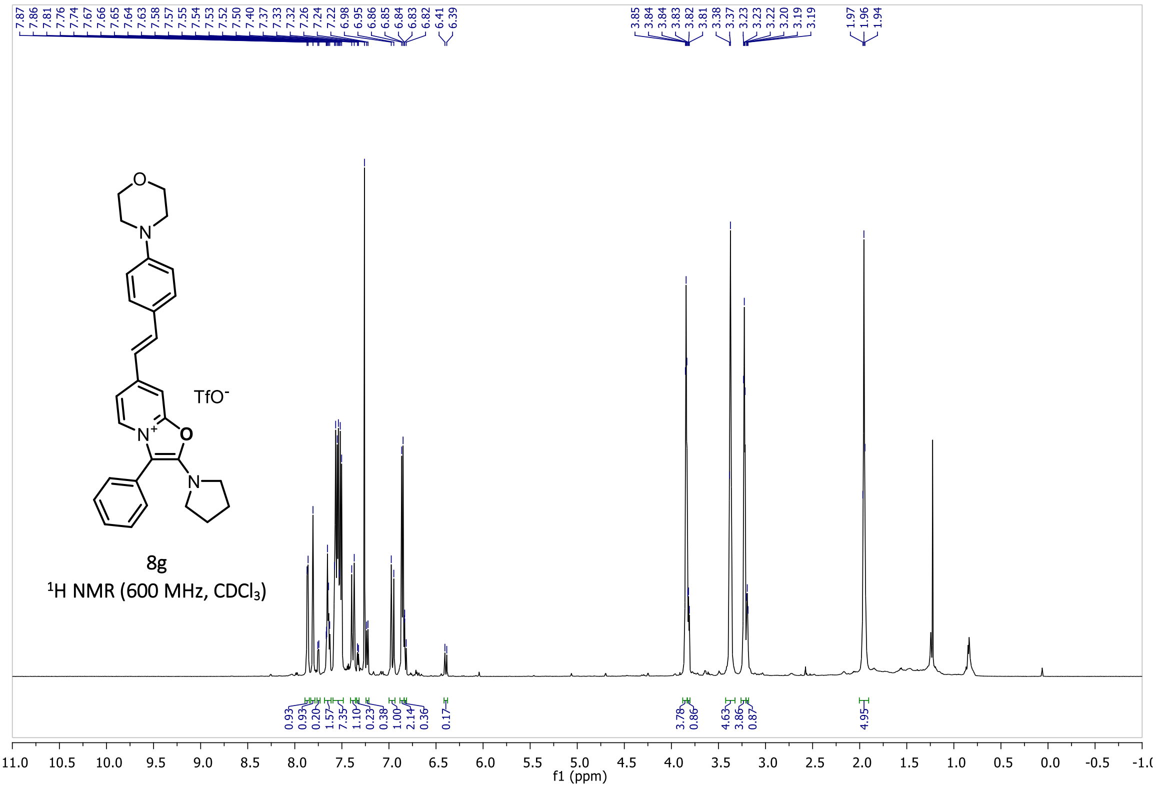

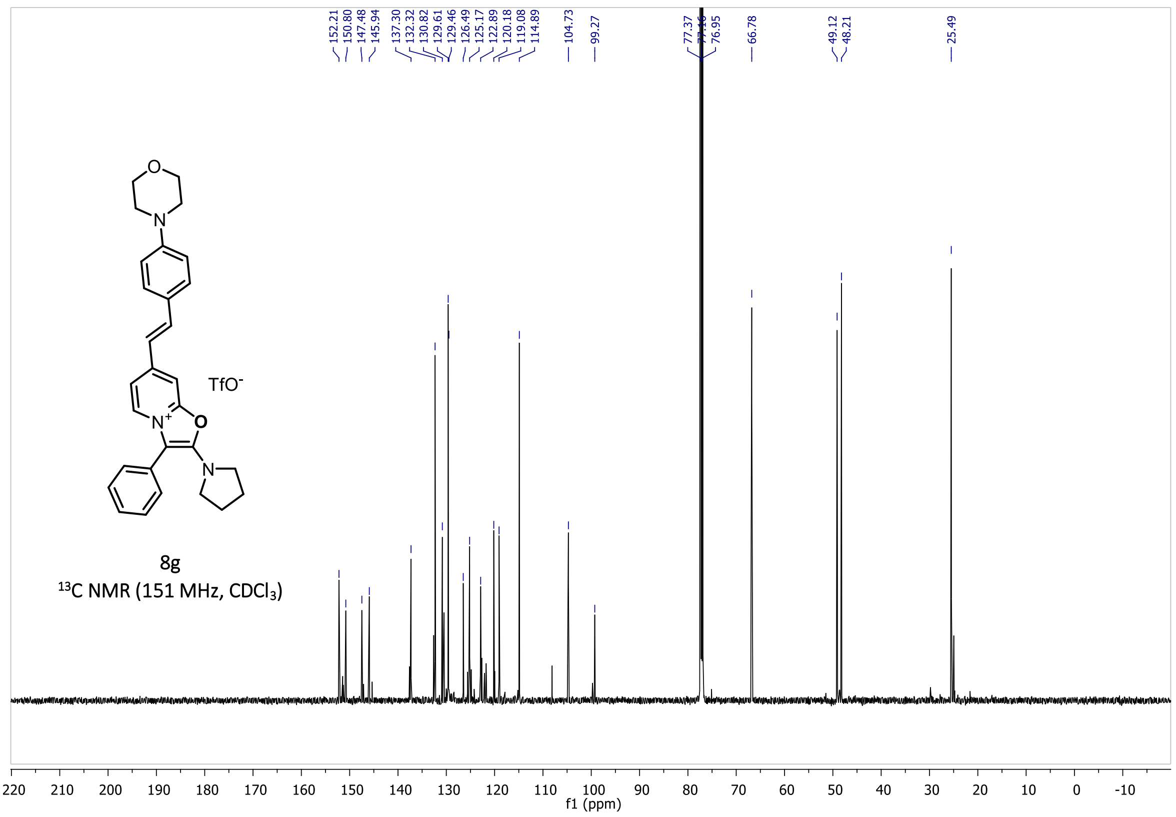
152.21
150.80
147.48
145.94
137.30
132.32
130.82
129.61
129.46
126.49
125.17
122.89
120.18
119.08
114.89
104.73
99.27
77.37
76.95
66.78
49.12
48.21
25.49
TfO-
8g
13C NMR (151 MHz, CDCl3)
f1 (ppm)

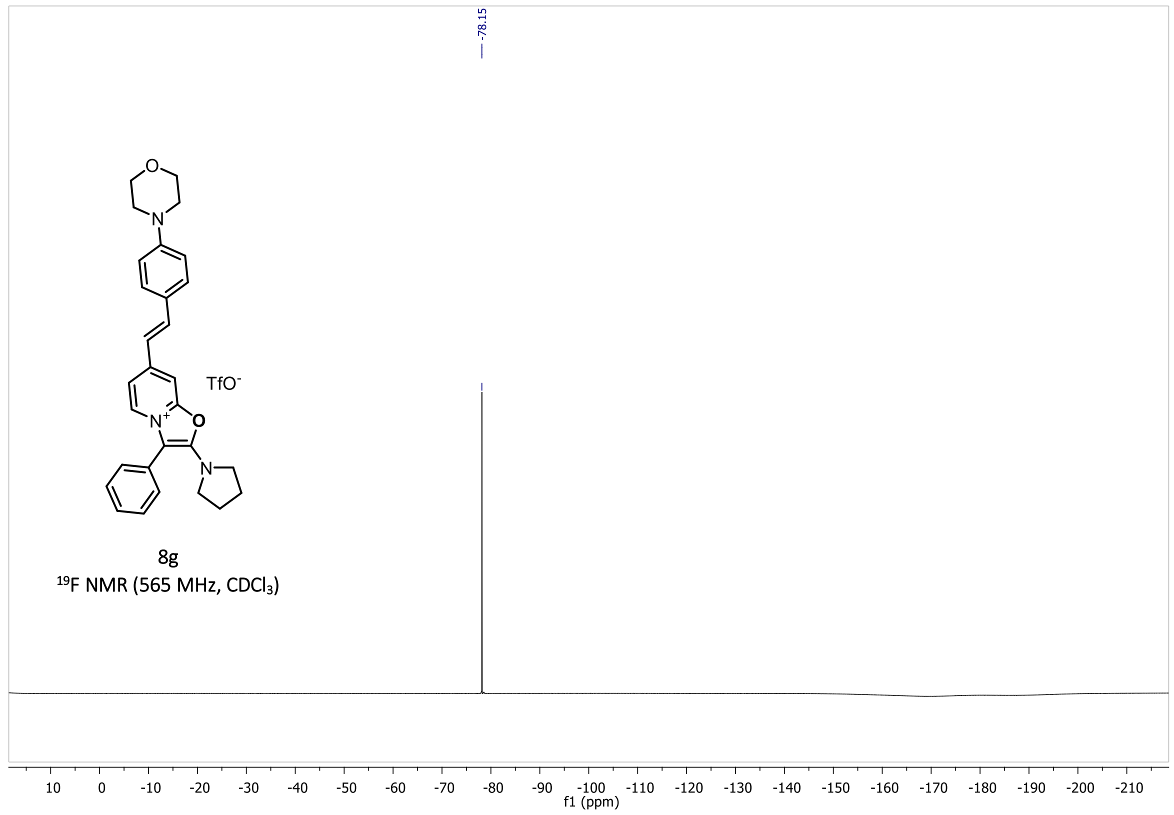
-78.15
TfO-
8g
19F NMR (565 MHz, CDCl3)
f1 (ppm)

7-((1*E*,3*E*)-4-(4-(Diethylamino)phenyl)buta-1,3-dien-1-yl)-3-phenyl-2-(pyrrolidin-1-yl)oxazolo[3,2-a]pyridin-4-ium trifluoromethanesulfonate (8h)

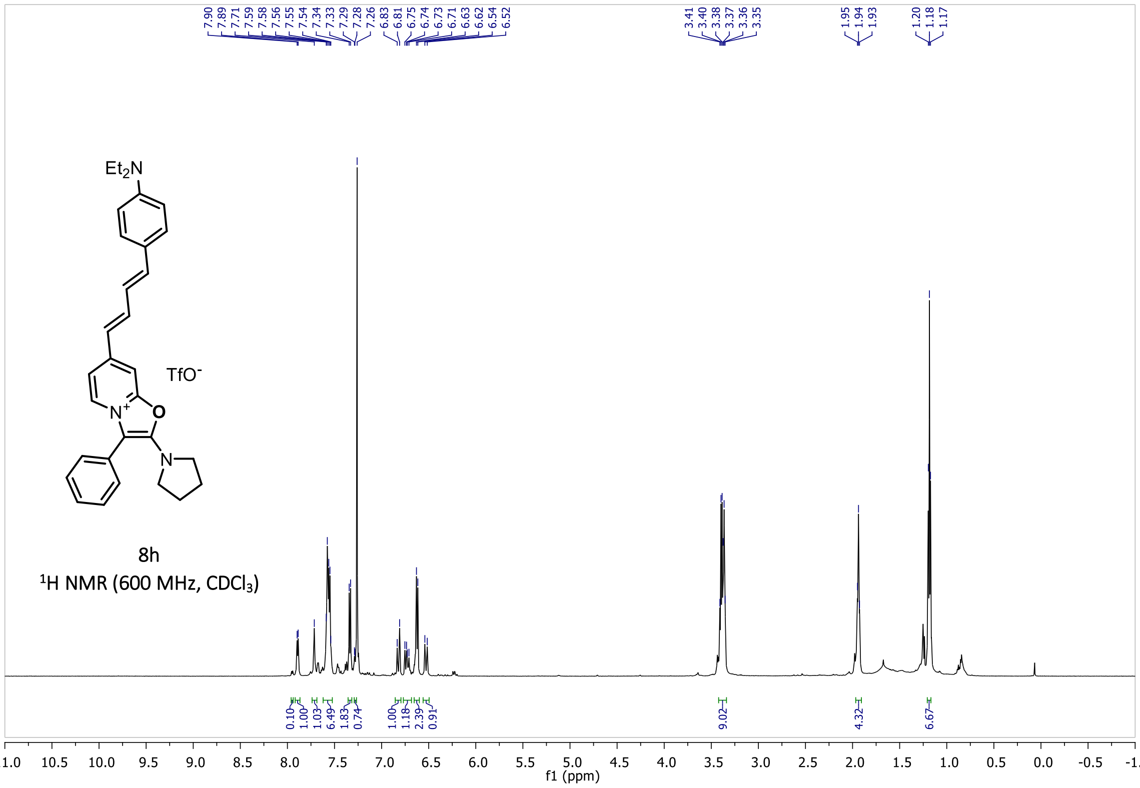

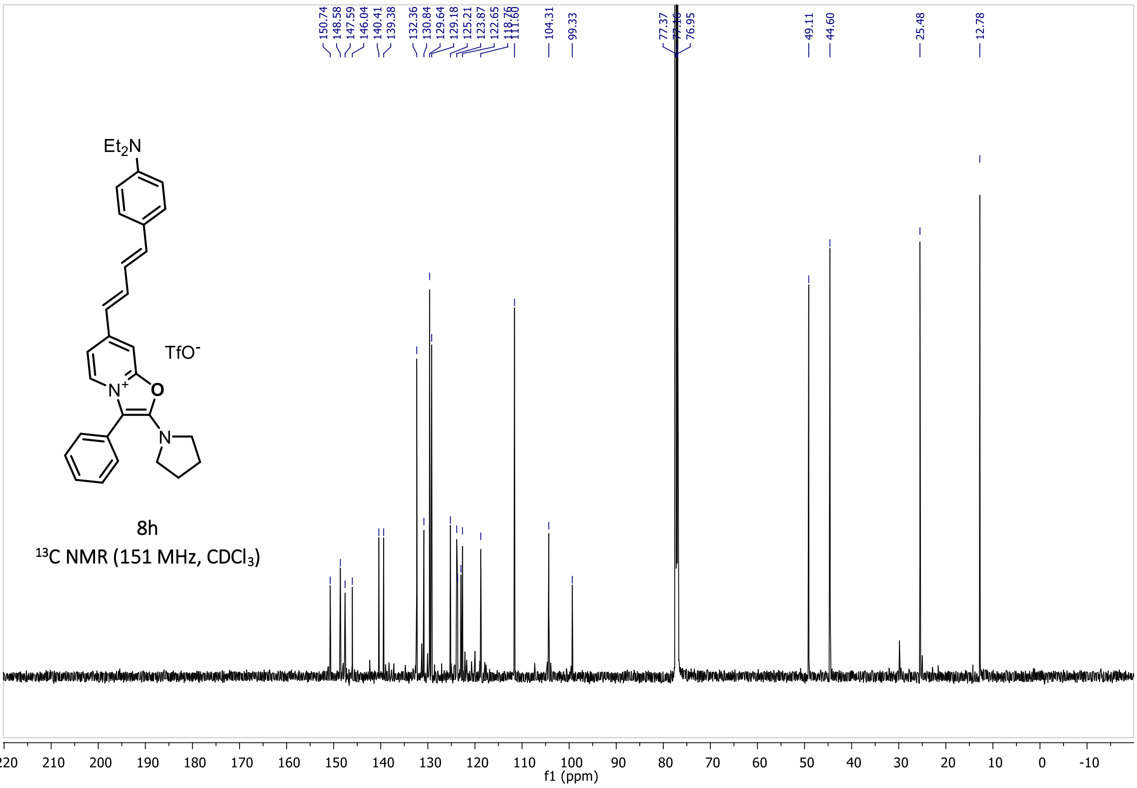

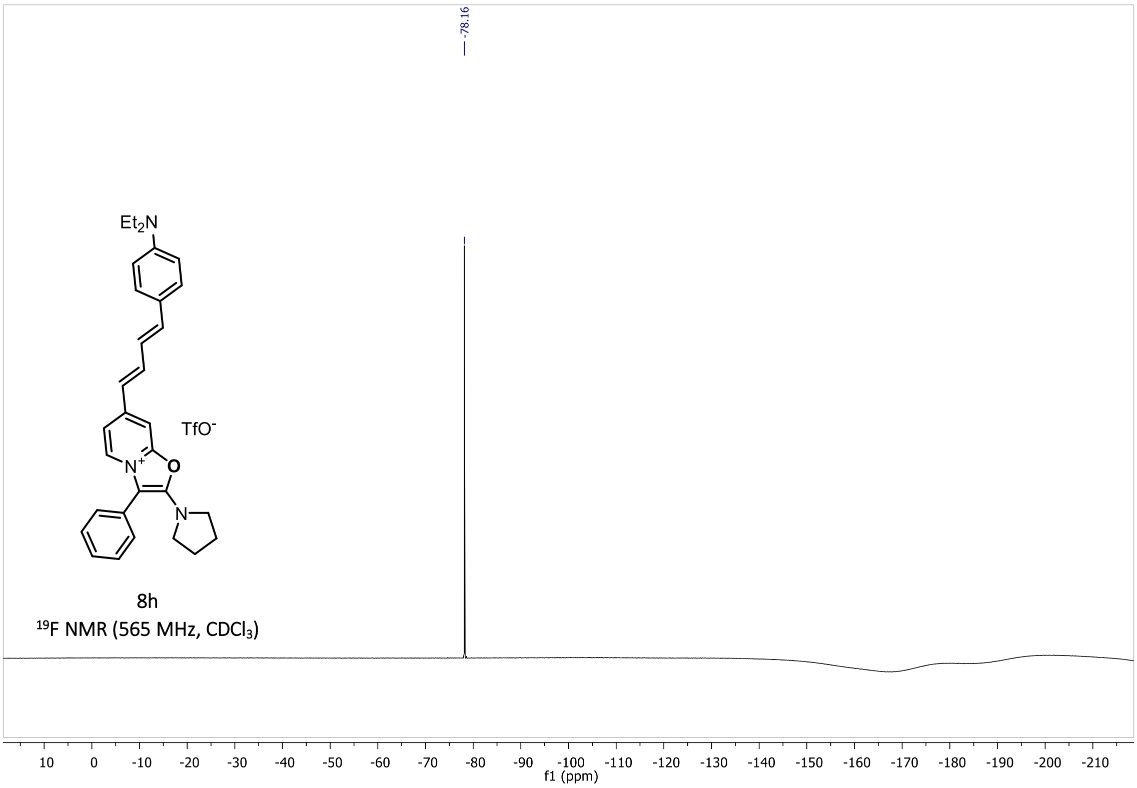

3-Phenyl-2-(pyrrolidin-1-yl)-7-((1*E*,3*E*)-4-(2,3,6,7-tetrahydro-1H,5H-pyrido[3,2,1-ij]quinolin-9-yl)buta-1,3-dien-1-yl)oxazolo[3,2-a]pyridin-4-ium trifluoromethanesulfonate (8i)

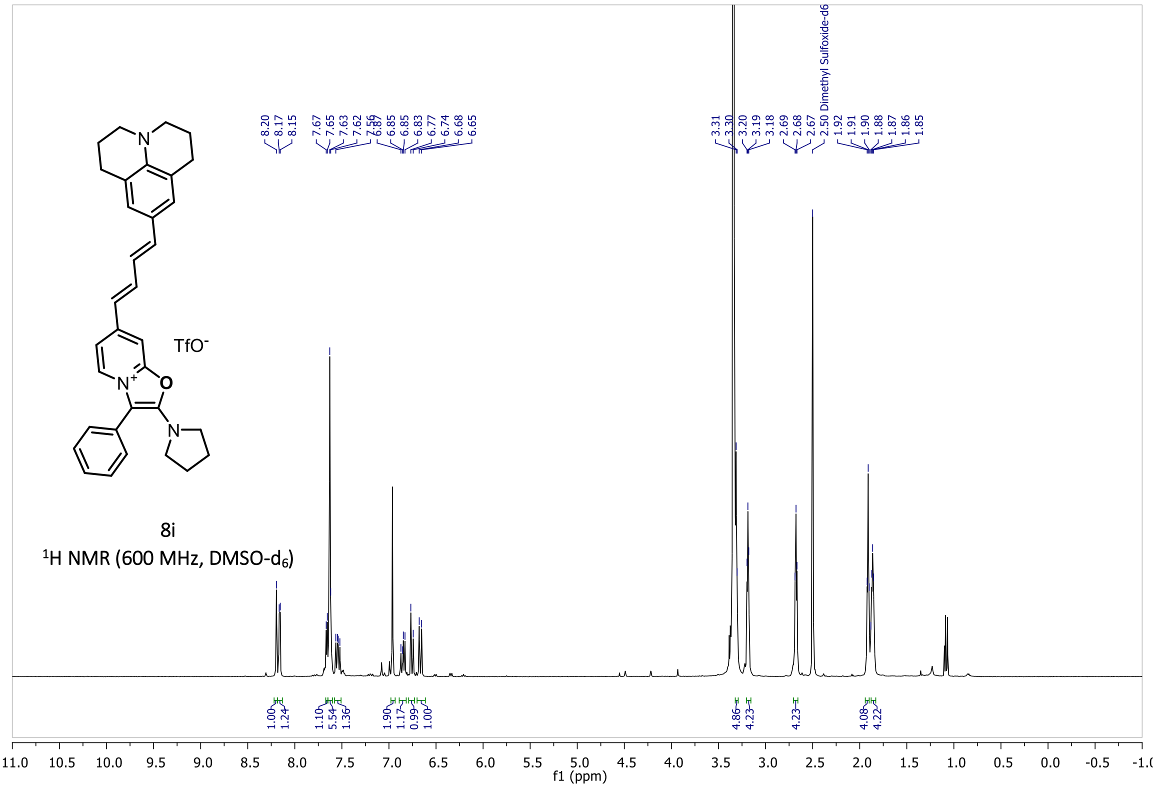

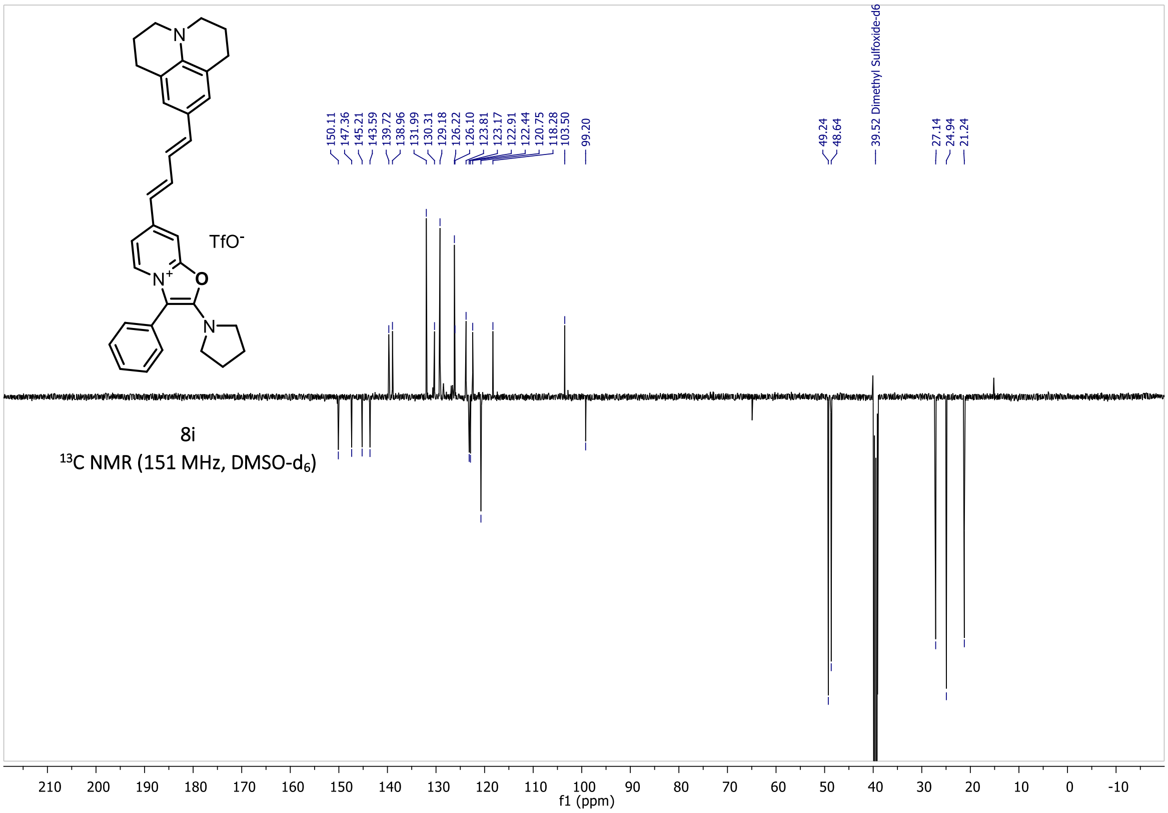

TfO-
8i
13C NMR (151 MHz, DMSO-d6)
150.11
147.36
145.21
143.59
139.72
138.96
131.99
130.31
129.18
126.22
126.10
123.81
123.17
122.91
122.44
120.75
118.28
103.50
99.20
49.24
48.64
39.52 Dimethyl Sulfoxide-d6
27.14
24.94
21.24
f1 (ppm)

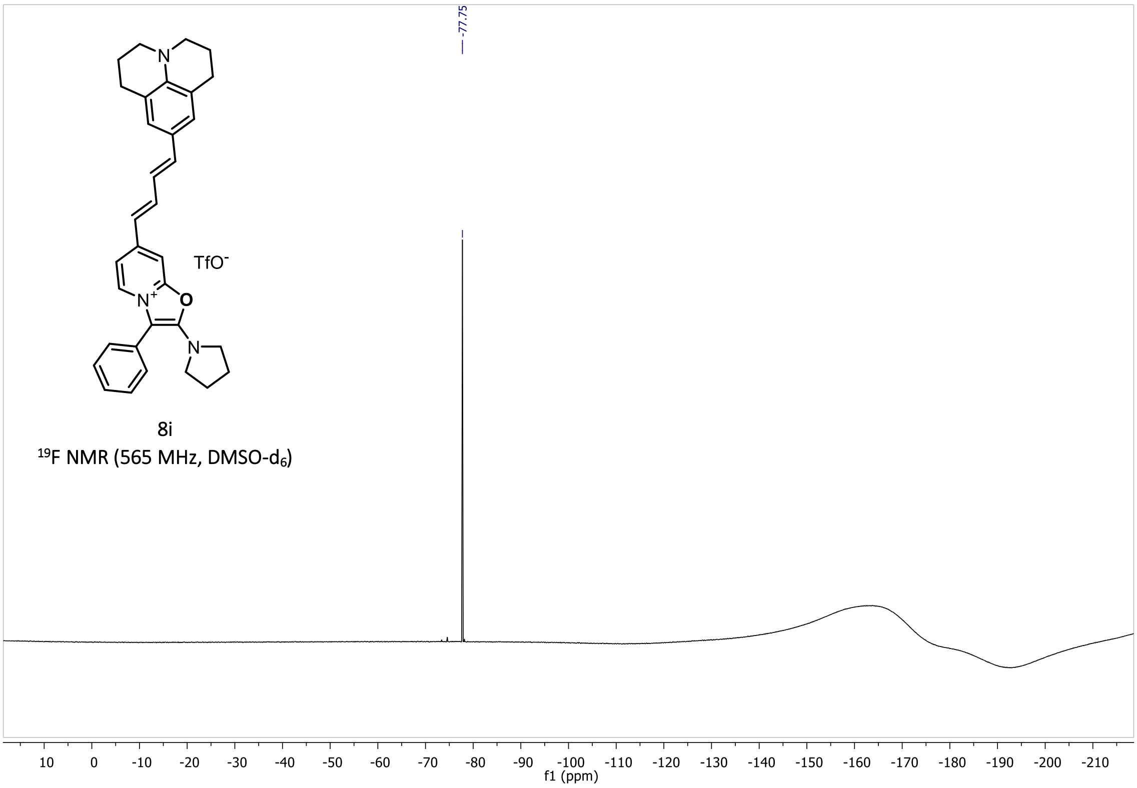

TfO-
8i
19F NMR (565 MHz, DMSO-d6)
-77.75
f1 (ppm)

7-(4-(Diethylamino)phenyl)-3-phenyl-2-(pyrrolidin-1-yl)thiazolo[3,2-a]pyridin-4-ium trifluoromethanesulfonate (9a)

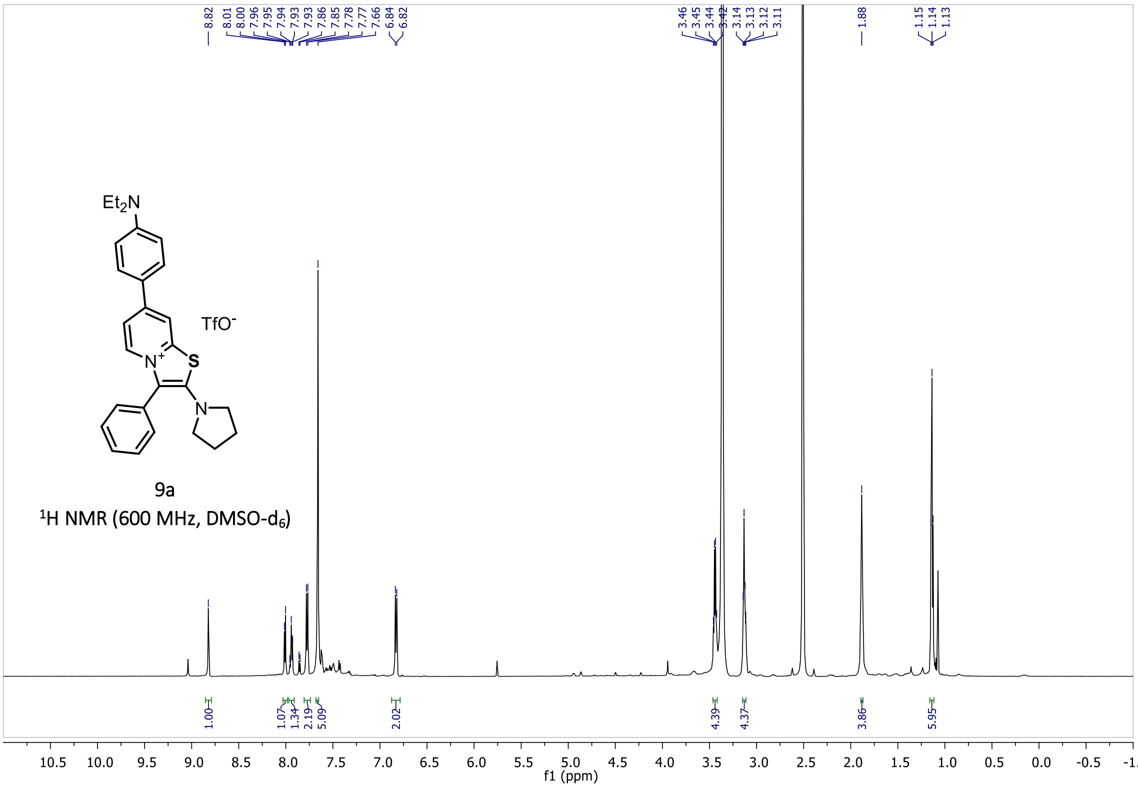

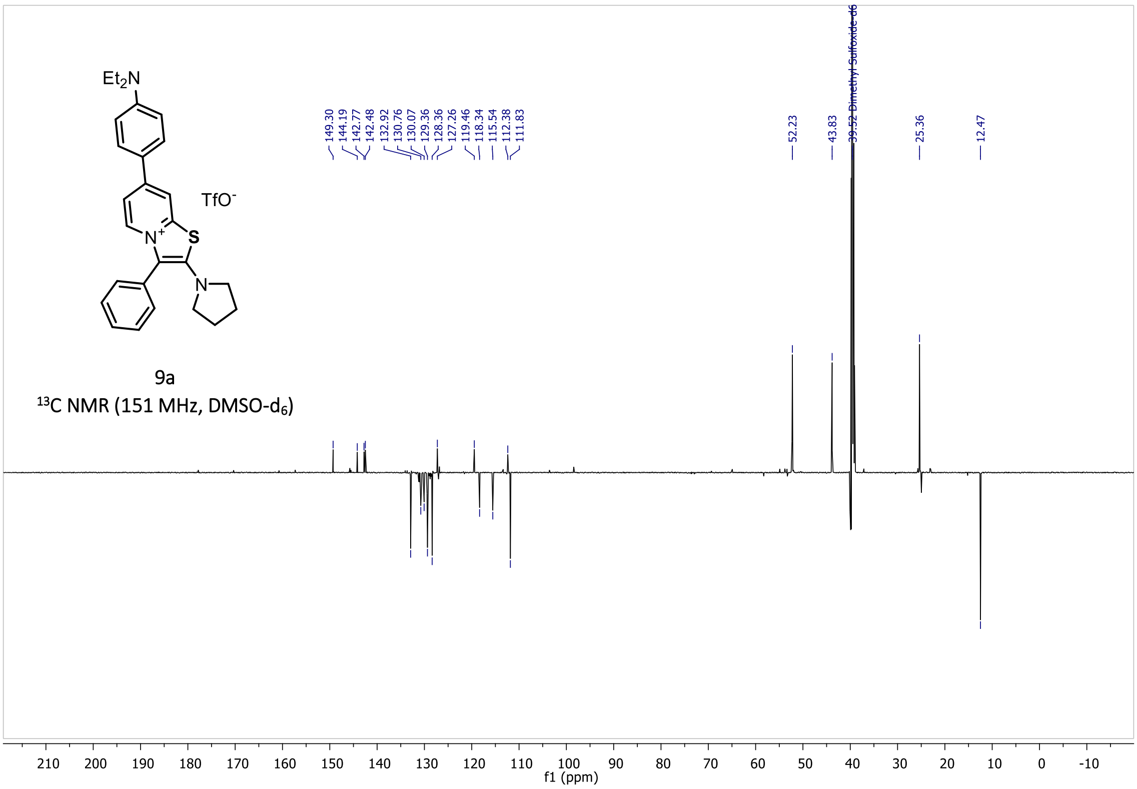

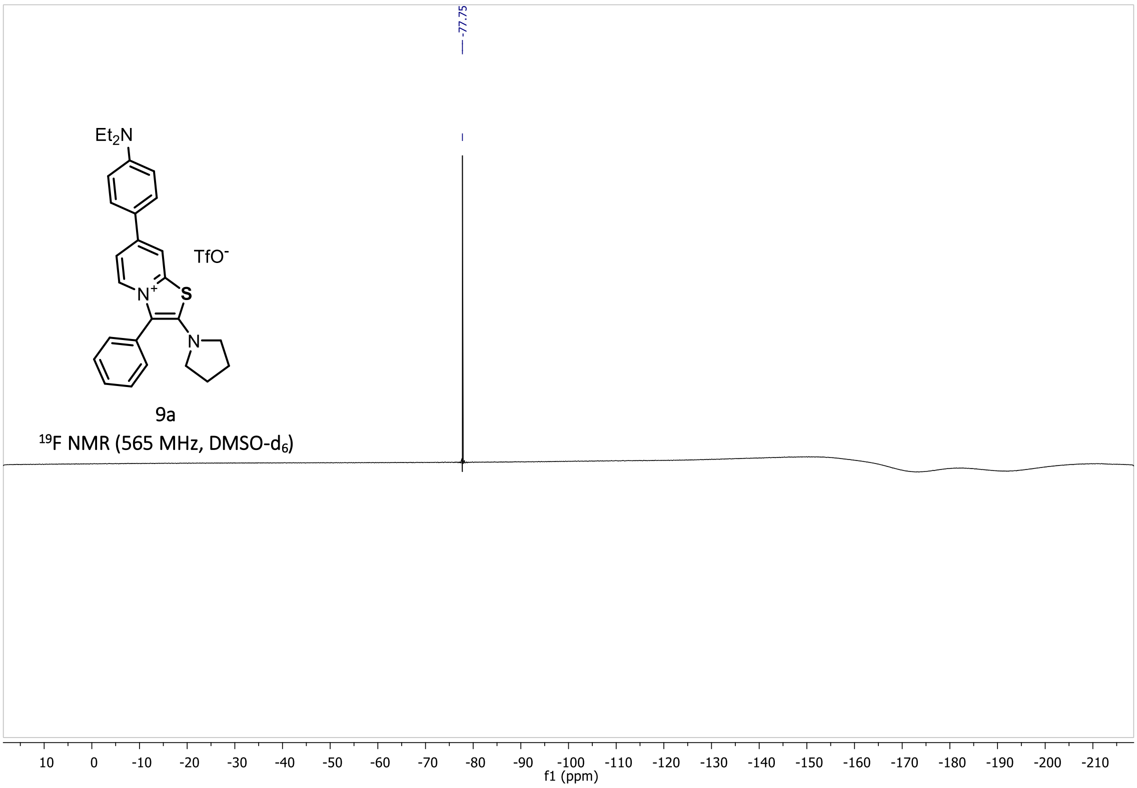

(*E*)-7-(4-(Diethylamino)styryl)-3-phenyl-2-(pyrrolidin-1-yl)thiazolo[3,2-a]pyridin-4-ium trifluoromethanesulfonate (9b)

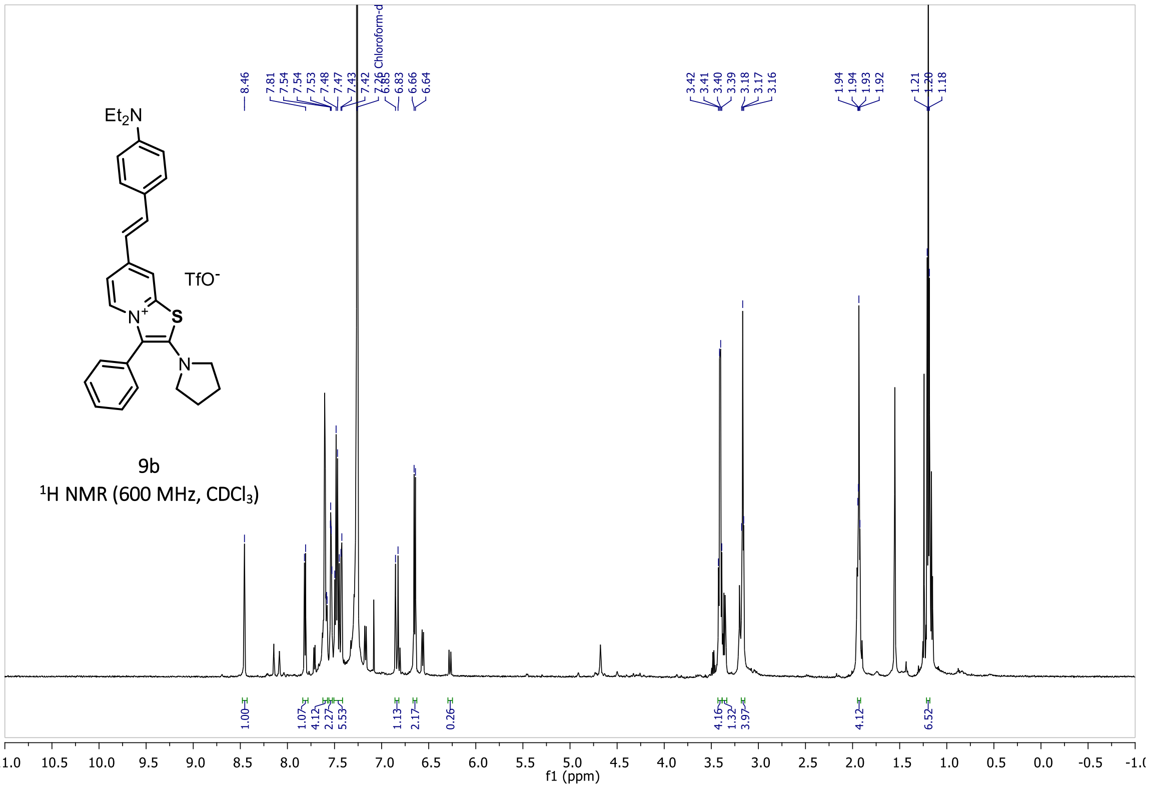

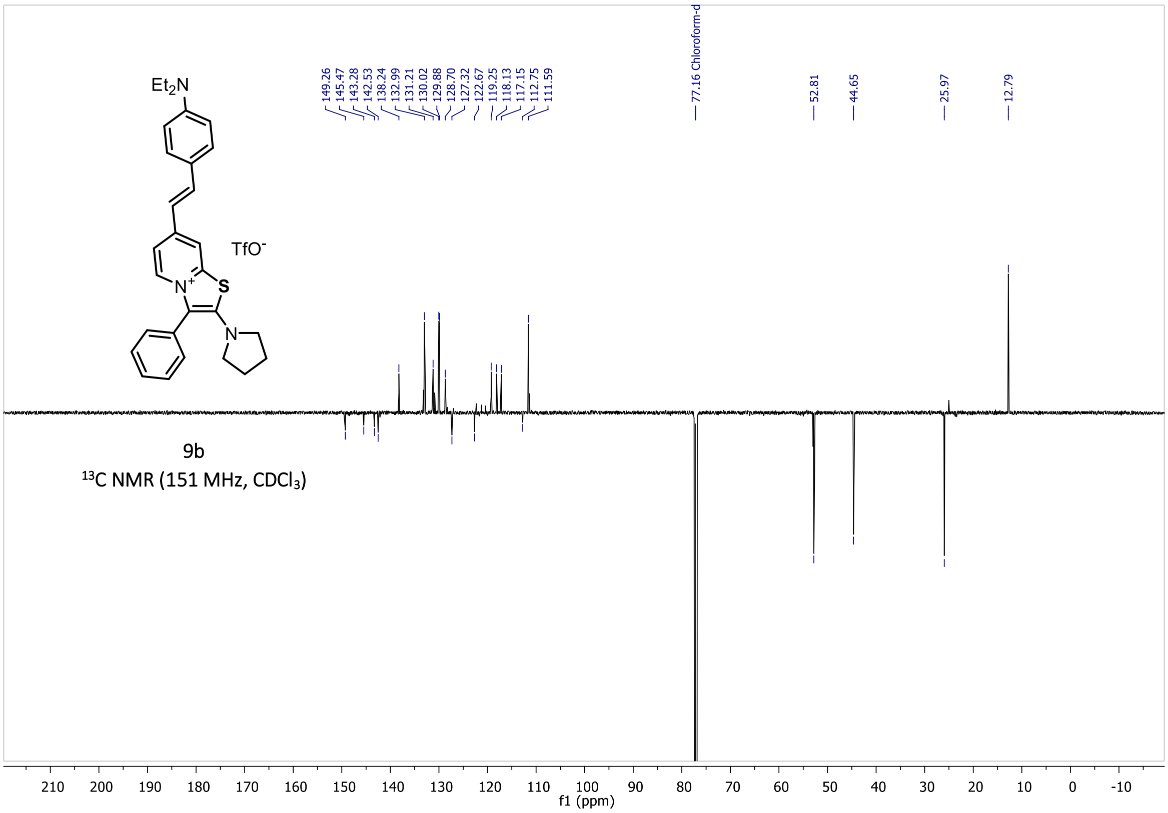

149.26
145.47
143.28
142.53
138.24
132.99
131.21
130.02
129.88
128.70
127.32
122.67
119.25
118.13
117.15
112.75
111.59
77.16 Chloroform-d
52.81
44.65
25.97
12.79
Et_2N
TfO⁻
N+
S
N
9b
^{13}C NMR (151 MHz, $CDCl_3$)
210 200 190 180 170 160 150 140 130 120 110 100 90 80 70 60 50 40 30 20 10 0 -10
f1 (ppm)

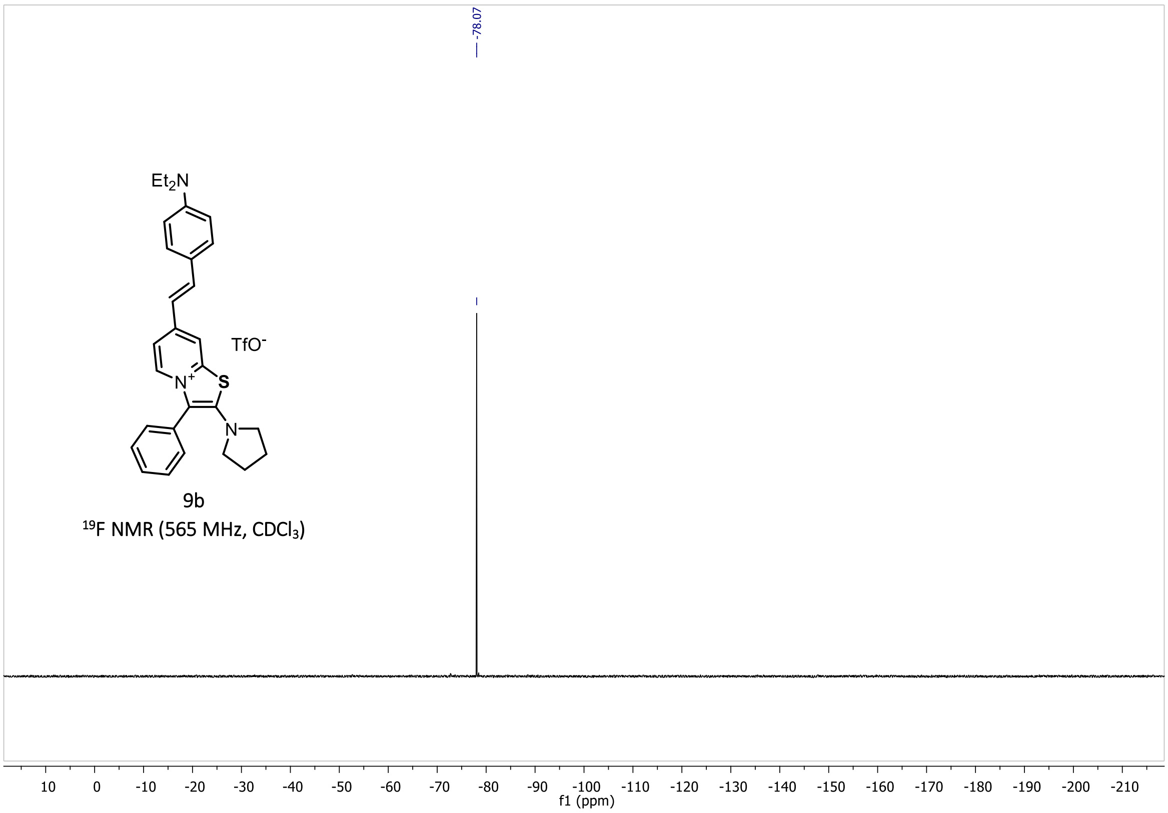

-78.07
Et_2N
TfO⁻
N+
S
N
9b
^{19}F NMR (565 MHz, $CDCl_3$)
10 0 -10 -20 -30 -40 -50 -60 -70 -80 -90 -100 -110 -120 -130 -140 -150 -160 -170 -180 -190 -200 -210
f1 (ppm)

13. References

(1) Würth, C.; Grabolle, M.; Pauli, J.; Spieles, M.; Resch-Genger, U. Relative and Absolute Determination of Fluorescence Quantum Yields of Transparent Samples. *Nat. Protoc.* **2013**, *8*, 1535–1550.

(2) Bolte, S.; Cordelières, F. P. A Guided Tour into Subcellular Colocalization Analysis in Light Microscopy. *J. Microsc.* **2006**, *224*, 213–232.

(3) Gonçalves, C. R.; Lemmerer, M.; Teskey, C. J.; Adler, P.; Kaiser, D.; Maryasin, B.; González, L.; Maulide, N. Unified Approach to the Chemoselective α-Functionalization of Amides with Heteroatom Nucleophiles. *J. Am. Chem. Soc.* **2019**, *141*, 18437–18443.

(4) de la Torre, A.; Kaiser, D.; Maulide, N. Flexible and Chemoselective Oxidation of Amides to α-Keto Amides and α-Hydroxy Amides. *J. Am. Chem. Soc.* **2017**, *139*, 6578–6581.

(5) Huh, D. H.; Jeong, J. S.; Lee, H. B.; Ryu, H.; Kim, Y. G. An Efficient Method for One-Carbon Elongation of Aryl Aldehydes via Their Dibromoalkene Derivatives. *Tetrahedron* **2002**, *58*, 9925–9932.

(6) Moeller, K. D.; Wang, P. W.; Tarazi, S.; Marzabadi, M. R.; Wong, P. L. Anodic Amide Oxidations in the Presence of Electron-Rich Phenyl Rings: Evidence for an Intramolecular Electron-Transfer Mechanism. *J. Org. Chem.* **1991**, *56*, 1058–1067.

(7) Jambu, S.; Sivasakthikumaran, R.; Jeganmohan, M. Aerobic Oxidative Alkenylation of Weak O-Coordinating Arylacetamides with Alkenes via a Rh(III)-Catalyzed C-H Activation. *Org. Lett.* **2019**, *21*, 1320–1324.

(8) Trillo, P.; Slagbrand, T.; Adolfsson, H. Straightforward α-Amino Nitrile Synthesis through Mo(CO)6 -Catalyzed Reductive Functionalization of Carboxamides. *Angew. Chem. Int. Ed.* **2018**, *57*, 12347–12351.

(9) Niu, Z.-J.; Li, L.-H.; Li, X.-S.; Liu, H.-C.; Shi, W.-Y.; Liang, Y.-M. Formation of O-Allyl- and Allenyl-Modified Amides via Intermolecular Claisen Rearrangement. *Org. Lett.* **2021**, *23*, 1315–1320.

(10) Kehner, R. A.; Zhang, G.; Bayeh-Romero, L. Mild Divergent Semireductive Transformations of Secondary and Tertiary Amides via Zirconocene Hydride Catalysis. *J. Am. Chem. Soc.* **2023**, *145*, 4921–4927.

(11) Zhang, H.; Riomet, M.; Roller, A.; Maulide, N. Synthesis of Novel Heterocycles by Amide Activation and Umpolung Cyclization. *Org. Lett.* **2020**, *22*, 2376–2380.

(12) Norman, J. P.; Larson, N. G.; Entz, E. D.; Neufeldt, S. R. Unconventional Site Selectivity in Palladium-Catalyzed Cross-Couplings of Dichloroheteroarenes under Ligand-Controlled and Ligand-Free Systems. *J. Org. Chem.* **2022**, *87*, 7414–7421.

(13) Yang, M.; Chen, J.; He, C.; Hu, X.; Ding, Y.; Kuang, Y.; Liu, J.; Huang, Q. Palladium-Catalyzed C-4 Selective Coupling of 2,4-Dichloropyridines and Synthesis of Pyridine-Based Dyes for Live-Cell Imaging. *J. Org. Chem.* **2020**, *85*, 6498–6508.

(14) Ruffoni, A.; Hampton, C.; Simonetti, M.; Leonori, D. Photoexcited Nitroarenes for the Oxidative Cleavage of Alkenes. *Nature* **2022**, *610*, 81–86.

(15) Lococo, M. D.; Zhang, X.; Jordan, R. F. Chelate-Controlled Synthesis of Racemic Ansa-Zirconocenes. *J. Am. Chem. Soc.* **2004**, *126*, 15231–15244.

(16) Suzuki, T.; Ota, Y.; Ri, M.; Bando, M.; Gotoh, A.; Itoh, Y.; Tsumoto, H.; Tatum, P. R.; Mizukami, T.; Nakagawa, H.; Iida, S.; Ueda, R.; Shirahige, K.; Miyata, N. Rapid Discovery of Highly Potent and Selective Inhibitors of Histone Deacetylase 8 Using Click Chemistry to Generate Candidate Libraries. *J. Med. Chem.* **2012**, *55*, 9562–9575.

(17) Meng, G.; Guo, T.; Ma, T.; Zhang, J.; Shen, Y.; Sharpless, K. B.; Dong, J. Modular Click Chemistry Libraries for Functional Screens Using a Diazotizing Reagent. *Nature* **2019**, *574*, 86–89.

(18) Yu, T.-B.; Bai, J. Z.; Guan, Z. Cycloaddition-Promoted Self-Assembly of a Polymer into Well-Defined Beta Sheets and Hierarchical Nanofibrils. *Angew. Chem. Int. Ed.* **2009**, *48*, 1097–1101.

(19) Das, S.; Mathur, A.; Sakhare, N.; Mallia, M. B.; Sarma, H. D.; Sachdev, S. S.; Dash, A. Synthesis and Biodistribution Studies of 99m Tc Labeled Fatty Acid Derivatives Prepared via “Click Approach” for Potential Use in Cardiac Imaging. *J. Labelled Comp. Radiopharm.* **2018**, *61*, 1048–1057.

(20) Stephan, M.; Stute, B.; von Lieres, E.; Müller, T. J. J. Consecutive Three-component Synthesis of Phenothiazine-based Merocyanines – Bayesian Optimization, Electronic Properties, and DSSC Characteristics. *Eur. J. Org. Chem.* **2022**, e202200163.

(21) Kim, E.; Koh, M.; Lim, B. J.; Park, S. B. Emission Wavelength Prediction of a Full-Color-Tunable Fluorescent Core Skeleton, 9-Aryl-1,2-Dihydropyrrolo[3,4-b]Indolizin-3-One. *J. Am. Chem. Soc.* **2011**, *133*, 6642–6649.

(22) Pietrzak, M.; Jędrzejewska, B. Aromatic Amines in Organic Synthesis. Part II. P-Aminocinnamaldehydes. *Molecules* **2021**, *26*, 4360.

(23) Liu, X.; Liu, S.; Wang, Q.; Zhou, G.; Yao, L.; Ouyang, Q.; Jiang, R.; Lan, Y.; Chen, W. Highly Regio- and Enantioselective Hydrogenation of Conjugated α-Substituted Dienoic Acids. *Org. Lett.* **2020**, *22*, 3149–3154.

(24) Zhao, J.; Zheng, X.; Tao, S.; Zhu, Y.; Yi, J.; Tang, S.; Li, R.; Chen, H.; Fu, H.; Yuan, M. Selective Rhodium-Catalyzed Hydroformylation of Terminal Arylalkynes and Conjugated Enynes to (Poly)Enals Enabled by a π-Acceptor Biphosphoramidite Ligand. *Org. Lett.* **2021**, *23*, 6067–6072.

(25) Liu, J.; Wu, C.; Hu, T.; Yang, W.; Xie, Y.; Shi, Y.; Liu, Q.; Shao, Y.; Zhang, F. Hexamethyldisilazane Lithium (LiHMDS)-Promoted Hydroboration of Alkynes and Alkenes with Pinacolborane. *J. Org. Chem.* **2022**, *87*, 3442–3452.

(26) Dolomanov, O. V.; Bourhis, L. J.; Gildea, R. J.; Howard, J. A. K.; Puschmann, H. *OLEX2*: A Complete Structure Solution, Refinement and Analysis Program. *J. Appl. Crystallogr.* **2009**, *42*, 339–341.

(27) Hübschle, C. B.; Sheldrick, G. M.; Dittrich, B. *ShelXle*: A Qt Graphical User Interface for*SHELXL*. *J. Appl. Crystallogr.* **2011**, *44*, 1281–1284.

(28) Spek, A. L. Structure Validation in Chemical Crystallography. *Acta Crystallogr. D Biol. Crystallogr.* **2009**, *65*, 148–155.

(29) Schrödinger Release 2019-2: MacroModel, Schrödinger, LLC, New York, NY, **2021**.

(30) Lee, C.; Yang, W.; Parr, R. G. Development of the Colle-Salvetti Correlation-Energy Formula into a Functional of the Electron Density. *Phys. Rev. B Condens. Matter* **1988**, *37*, 785–789.

(31) Grimme, S.; Antony, J.; Ehrlich, S.; Krieg, H. A Consistent and Accurate Ab Initio Parametrization of Density Functional Dispersion Correction (DFT-D) for the 94 Elements H-Pu. *J. Chem. Phys.* **2010**, *132*, 154104.

(32) Schäfer, A.; Horn, H.; Ahlrichs, R. Fully Optimized Contracted Gaussian Basis Sets for Atoms Li to Kr. *J. Chem. Phys.* **1992**, *97*, 2571–2577.

(33) Schäfer, A.; Huber, C.; Ahlrichs, R. Fully Optimized Contracted Gaussian Basis Sets of Triple Zeta Valence Quality for Atoms Li to Kr. *J. Chem. Phys.* **1994**, *100*, 5829–5835.

(34) Miertuš, S.; Scrocco, E.; Tomasi, J. Electrostatic Interaction of a Solute with a Continuum. A Direct Utilizaion of AB Initio Molecular Potentials for the Prevision of Solvent Effects. *Chem. Phys.* **1981**, *55*, 117–129.

(35) Miertuš̃, S.; Tomasi, J. Approximate Evaluations of the Electrostatic Free Energy and Internal Energy Changes in Solution Processes. *Chem. Phys.* **1982**, *65*, 239–245.

(36) Frisch, M. J.; Trucks, G. W.; Schlegel, H. B.; Scuseria, G. E.; Robb, M. A.; Cheeseman, J. R.; Scalmani, G.; Barone, V.; Petersson, G. A.; Nakatsuji, H.; Li, X.; Caricato, M.; Marenich, A. V.; Bloino, J.; Janesko, B. G.; Gomperts, R.; Mennucci, B.; Hratchian, H. P.; Ortiz, J. V.; Izmaylov, A. F.; Sonnenberg, J. L.; Williams-Young, D.; Ding, F.; Lipparini, F.; Egidi, F.; Goings, J.; Peng, B.; Petrone, A.; Henderson, T.; Ranasinghe, D.; Zakrzewski, V. G.; Gao, J.; Rega, N.; Zheng, G.; Liang, W.; Hada, M.; Ehara, M.; Toyota, K.; Fukuda, R.; Hasegawa, J.; Ishida, M.; Nakajima, T.; Honda, Y.; Kitao, O.; Nakai, H.; Vreven, T.; Throssell, K.; Montgomery, J. A., Jr.; Peralta, J. E.; Ogliaro, F.; Bearpark, M. J.; Heyd, J. J.; Brothers, E. N.; Kudin, K. N.; Staroverov, V. N.; Keith, T. A.; Kobayashi, R.; Normand, J.; Raghavachari, K.; Rendell, A. P.; Burant, J. C.; Iyengar, S. S.; Tomasi,

J.; Cossi, M.; Millam, J. M.; Klene, M.; Adamo, C.; Cammi, R.; Ochterski, J. W.; Martin, R. L.; Morokuma, K.; Farkas, O.; Foresman, J. B.; Fox, D. J. Gaussian 16, Revision A.03, Gaussian, Inc., Wallingford CT, **2016**.

(37) Schirmer, J. Beyond the Random-Phase Approximation: A New Approximation Scheme for the Polarization Propagator. *Phys. Rev. A Gen. Phys.* **1982**, *26*, 2395–2416.

(38) Trofimov, A. B.; Schirmer, J. An Efficient Polarization Propagator Approach to Valence Electron Excitation Spectra. *J. Phys. B: At. Mol. Opt. Phys.* **1995**, *28*, 2299–2324.

(39) Klamt, A.; Schüürmann, G. COSMO: A New Approach to Dielectric Screening in Solvents with Explicit Expressions for the Screening Energy and Its Gradient. *J. Chem. Soc., Perkin Trans. 2* **1993**, No. 5, 799–805.

(40) Balasubramani, S. G.; Chen, G. P.; Coriani, S.; Diedenhofen, M.; Frank, M. S.; Franzke, Y. J.; Furche, F.; Grotjahn, R.; Harding, M. E.; Hättig, C.; Hellweg, A.; Helmich-Paris, B.; Holzer, C.; Huniar, U.; Kaupp, M.; Marefat Khah, A.; Karbalaei Khani, S.; Müller, T.; Mack, F.; Nguyen, B. D.; Parker, S. M.; Perlt, E.; Rappoport, D.; Reiter, K.; Roy, S.; Rückert, M.; Schmitz, G.; Sierka, M.; Tapavicza, E.; Tew, D. P.; van Wüllen, C.; Voora, V. K.; Weigend, F.; Wodyński, A.; Yu, J. M. TURBOMOLE: Modular Program Suite for Ab Initio Quantum-Chemical and Condensed-Matter Simulations. *J. Chem. Phys.* **2020**, *152*, 184107.

(41) Luzanov, A. V.; Sukhorukov, A. A.; Umanskii, V. É. Application of Transition Density Matrix for Analysis of Excited States. *Theor. Exp. Chem.* **1976**, *10*, 354–361.

(42) Martin, R. L. Natural Transition Orbitals. *J. Chem. Phys.* **2003**, *118*, 4775–4777.

(43) Plasser, F.; Lischka, H. Analysis of Excitonic and Charge Transfer Interactions from Quantum Chemical Calculations. *J. Chem. Theory Comput.* **2012**, *8*, 2777–2789.

(44) Plasser, F.; Wormit, M.; Dreuw, A. New Tools for the Systematic Analysis and Visualization of Electronic Excitations. I. Formalism. *J. Chem. Phys.* **2014**, *141*, 024106.

(45) Bäppler, S. A.; Plasser, F.; Wormit, M.; Dreuw, A. Exciton Analysis of Many-Body Wave Functions: Bridging the Gap between the Quasiparticle and Molecular Orbital Pictures. *Phys. Rev. A* **2014**, *90*, 052521.

(46) Mewes, S. A.; Plasser, F.; Dreuw, A. Communication: Exciton Analysis in Time-Dependent Density Functional Theory: How Functionals Shape Excited-State Characters. *J. Chem. Phys.* **2015**, *143*, 171101.

(47) Plasser, F. TheoDORE: A Toolbox for a Detailed and Automated Analysis of Electronic Excited State Computations. *J. Chem. Phys.* **2020**, *152*, 084108.
